# Soil microbial community succession and physicochemical property changes affect *Ganoderma leucocontextum* growth in the Dadu river basin

**DOI:** 10.3389/fmicb.2025.1666459

**Published:** 2026-01-07

**Authors:** Bo Zhang, Xuezhen Yang, Qing Tian, Lei Ye, Zhenzhu Huang, Wei Tan, Lei Zhou, Hang Chen, Xiaolin Li

**Affiliations:** 1 Sichuan Institute of Edible Fungi, Sichuan Academy of Agricultural Sciences, Chengdu, China; 2 Chengdu Science & Innovation Fungi Industry Co., Ltd., Chengdu, China; 3 Agricultural Science Institute of Ganzi Tibetan Autonomous Prefecture, Ganzi, China

**Keywords:** *Ganoderma leucocontextum*, casing soil, continuous cropping obstacles (CCOs), microbial communities, physicochemical properties, bioactive compounds

## Abstract

*Ganoderma leucocontextum* is rich in bioactive compounds, including triterpenes and polysaccharides, and exhibits significant pharmacological effects. Its cultivation requires casing soil, crucial for achieving high productivity and superior quality. In this study, soil physicochemical properties and microbial communities were analyzed across four growth stages: casing (GCK), primordial (G1p), cap (G1c), and maturity (G1m) of *G. leucocontextum*. Results indicated that the soil pH significantly increased after cultivation, ranging from 6.78 to 7.11. The control soil contained the highest concentrations of total nitrogen (2.44 g/kg), available nitrogen (259.48 mg/kg) and organic matter (54.35 g/kg), significantly exceeding those in *G. leucocontextum*-cultivated soils. Soil available phosphorus and potassium gradually increased, peaking at maturity (42.01 mg/kg and 86.36 mg/kg, respectively). Microbial communities also shifted from bacterial to fungal dominance over time. Among bacteria, Acidobacteriota was the most prevalent phylum, averaging 28.46%, with a marked upward trend. *Arthrobacter* emerged as the most dominant genus, averaging 9.00%, with higher abundance at maturity. A Vicinamibacterales-order genus continuously increased in abundance, wheras *Novocardioides, Sphingomonas*, and an Intrasporangiaceae-family genus decreased during of *G. leucocontextum* growth. For fungi, Ascomycota was the most prevalent phylum, averaging 65.56%, followed by Basidiomycota at 21.60%, which dominated at maturity. *Ganoderma* was the most predominant genus, averaging 16.34%, and increased substantially with growth. The study revealed correlations between soil microbial communities and physicochemical properties, and demonstrated decreasing polysaccharide content but increasing triterpenoid acid content during growth. This research explores soil microbial community succession and physicochemical changes for *G. leucocontextum* cultivation, offering theoretical support for overcoming continuous cropping obstacles (CCOs) and insights for sustainable yield management.

## Introduction

Identified in 2015 as a novel species of the *Ganoderma* genus, *G. leucocontextum* exhibits greater suitability for low-temperature environments. Predominantly found in the Tibetan Plateau region (Liu Y. L. et al., 2021; Li et al., 2015), this fungus is rich in bioactive compounds such as triterpenes and polysaccharides. These substances demonstrate pharmacological effects, including anti-tumor, hypoglycemic, lipid-lowering, and immune-regulating properties. Notably, triterpenes and polysaccharides concentrations in *G. leucocontextum* are significantly higher than in *G. lucidum* (Baby et al., 2015; Liu Y. F. et al., 2021). In Southwest China, log cultivation is the primary method for growing *G. leucocontextum*, with logs providing essential nutrients and casing soil ensuring stable humidity and ventilation. However, continuous cropping in the same soil leads to CCOs, resulting in reduced yield and quality, increased pest infestations, and soil-borne pathogen infections (Yao et al., 2022, 2023).

Continuous cropping challenges for edible fungi include soil nutrient imbalances, shifts in microbial communities, and autotoxicity of cultivation strains (Jiang et al., 2021; Lu et al., 2022). Potential solutions such as liquid ammonia fumigation and lime-nitrogen treatment have been reported as effective interventions (Zhao C. Y. et al., 2023; Peruzzi et al., 2011). Meanwhile, substance accumulation from mushroom cultivation contributes to CCOs (Zhu et al., 2021). As a macrofungus, *G. leucocontextum* plays a key role in organic matter decomposition and synthesis, influencing soil carbon metabolism and nitrogen cycling. Soil properties, substrate characteristics and *Ganoderma* spp. interact during growth, altering environmental factors like temperature, enzyme activity, and microbial communities (Yao et al., 2022). Therefore, investigating microbial succession, physicochemical changes, and the soil-microorganism-fungus relationship is essential. Bacterial communities particularly affect substrate properties at *G. lucidum* elongation stage (Zhang et al., 2018), and CCOs in morel cultivation are linked to soil microbial shifts (Liu et al., 2022).

Generally, continuous cropping issues for fungi like *G. lucidum* are connected to soil microbial and physicochemical alterations. Few studies have examined the soil-microorganism-fungus dynamic during *G. leucocontextum* growth. And the mechanism by which microbial communities and soil properties progressively cause CCOs remains unreported. In this study, next-generation sequencing characterized soil microbial composition and diversity. We investigated soil properties and *G. leucocontextum* growth indicators across four stages. The findings offer theoretical support for overcoming CCOs and developing better cultivation strategies to enhance yield and quality.

## Materials and methods

### Cultivation of *G. leucocontextum*

The *G. leucocontextum* cultivar, Kangding Lingzhi, was sourced from the Ganzi Agricultural Sciences Institute in Sichuan, China, and officially recognized by the Sichuan Provincial Crop Variety Approval Committee in December 2016. *G. leucocontextum* cultivation utilized *Fagus sylvatica* log substrates, with lengths of 15–18 cm and diameters of 13–14 cm. These logs were placed into polypropylene cultivation bags measuring 30 cm × 44 cm × 0.005 cm. with each bag secured using a tie at one end. The log-filled bags were then autoclaved at 121°C for 2.5 h to eliminate most microorganisms and establish a relatively sterile environment conducive to mycelial germination.

Following sterilization, the logs were transferred to a shed and cooled at room temperature for inoculation. A liquid inoculation method employed approximately 25 mL of *G. leucocontextum* liquid spawn per log-filled bag. Subsequently, the bags were placed on pre-ventilated shelves in a greenhouse for incubation. During this phase, mycelia germinated and grew at temperature ranging from 21 to 23°C. After 55 to 60 days, the mycelia fully colonized the logs. The bags were then removed and transported to the cultivation site in Yanzigou, Luding, China (N 29°26′53″, E 102°26′56″), which featured an arched shed covered with nets. The mycelia-colonized logs were positioned in soil plots at a depth of 25 cm and covered with a 5–8 cm soil layer. This soil had been cleaned and disinfected with lime treatment. The optimal growth period for *G. leucocontextum* spans April to September, with temperatures maintained between 21 and 26°C and humidity controlled at 90% to 95%.

### Sample collection

**Figure 1:**
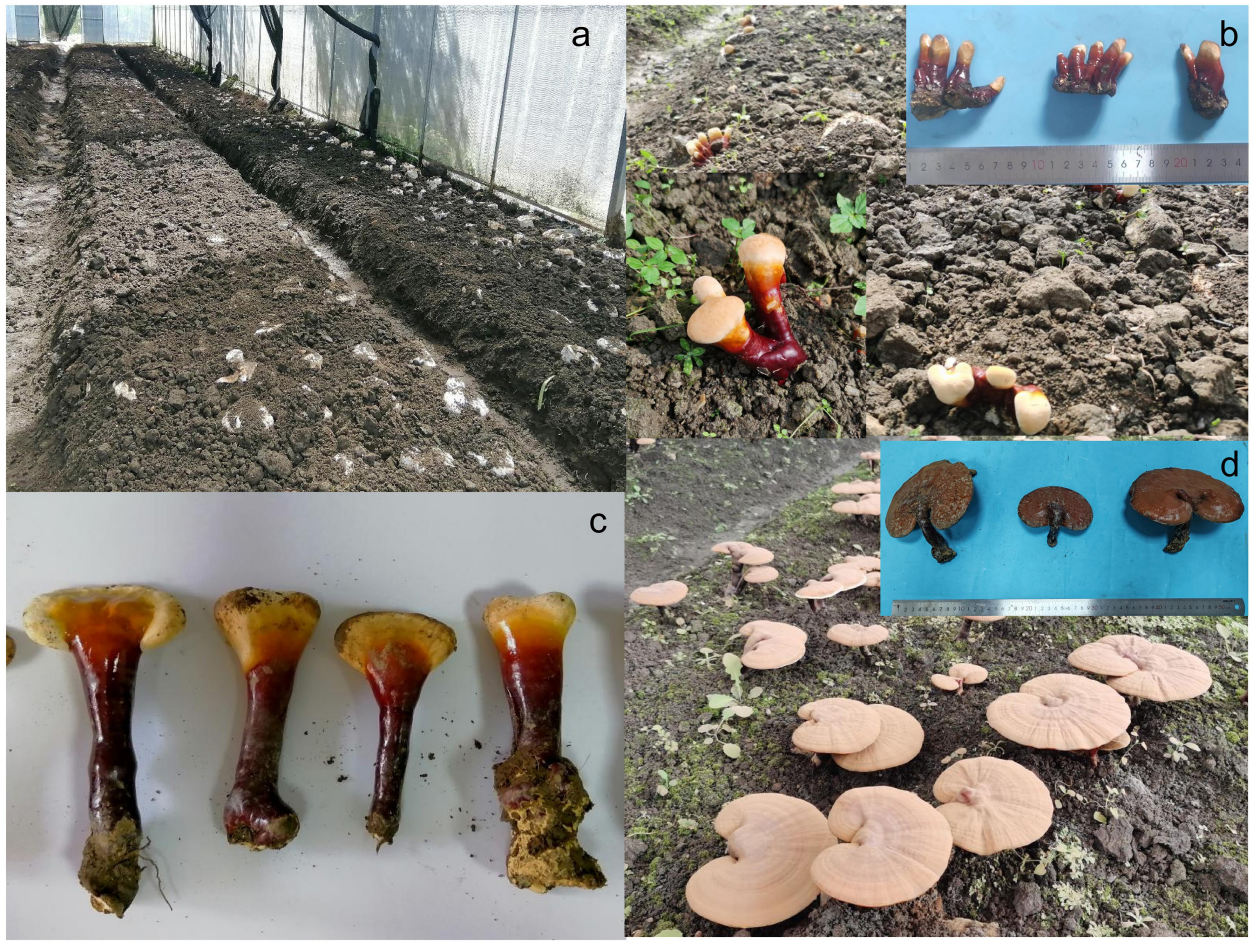
*G. leucocontextum* status at different growth stages. **(a)**, Soil covering of *G. leucocontextum* logs on April 24th; **(b)**, Primordium emerging on June 26th; **(c)**, Cap formation on August 10th; **(d)**, The fruiting body maturity on September 8th.

Soil samples were collected at four stages of *G. leucocontextum* growth: casing soil (GCK), primordium emergence (G1p), cap formation (G1c), and maturity (G1m) (Figure 1). The initial sampling occurred on April 24^th^ before covering the mycelia-colonized logs with casing soil, serving as the control group. After soil covering, the mycelia spread and intertwined. The second sampling took place at the primordial stage on June 26^th^, when primordia formed and began differentiation. As *G. leucocontextum* grew, the promordia elongated and stipe formed. Cap differentiation commenced after elongation, prompting the third sampling on August 10^th^. The final sampling occurred at the maturity stage on September 8^th^ (approximately 137 days after casing soil), when spores appeared on the pileus surface and gradually covered the yellow edges. At each stage, soil was collected from three random points at depths of 5–10 cm using disposable gloves and a sterilized scraper. The bulk soil samples were combined, mixed, and stored at −80°C in 2 mL Eppendorf tubes for DNA extraction, yielding a total of twelve samples.

### Soil physicochemical property determination

Soil samples were air-dried to facilitate physicochemical property assessment. Soil pH was measured via potentiometric method with a soil/water ratio of 1:2.5 (Yang et al., 2020). Total nitrogen content was quantified using an FIAstar 5,000 Analyzer (Foss Tecator, Denmark) to determine NH^4+^-N and NO^3−^-N amounts (Huang et al., 2015). Organic matter content was analyzed via the K_2_Cr_2_O_7_ oxidation method (Xie et al., 2023). Available nitrogen, phosphorus, and potassium contents were determined using alkali hydrolysis diffusion, hydrochloric acid ammonium chloride, and flame spectrometry methods, respectively (Zhao L. et al., 2023; Song et al., 2020; Ieggli et al., 2011).

### DNA extraction and PCR amplification and sequencing

Total microbial genomic DNA was extracted from soil samples using the E.Z.N.A.^®^ Soil DNA Kit (Omega Bio-tek, Norcross, GA, USA) according to the manufacturer's protocol. DNA quality and concentration were assessed via 1.0% agarose gel electrophoresis and quantified using a NanoDrop 2000 spectrophotometer (Thermo Scientific, USA). DNA samples were stored at −80°C for further analysis. PCR amplification was performed by Majorbio Bio-Pharm Technology Co. Ltd. (Shanghai, China). Bacterial amplification utilized primers 338F (5′-ACTCCTACGGGAGGCAGCA-3′) and 806R (5′-GGACTACHVGGGTWTCTAAT-3′), while fungal amplification employed ITS1F (5′-CTTGGTCATTTAGAGGAAGTAA-3′) and ITS2R (5′-GCTGCGTTCTTCATCGATGC-3′) (Li et al., 2025). Thermal cycling conditions included initial denaturation at 95°C for 3 min, followed by 27 bacterial or 35 fungal cycles of denaturation (95°C, 30 s), annealing (55°C, 30 s), and extension (72°C, 45 s), with a final extension at 72°C for 10 min, ending at 4°C (Liu et al., 2016). The PCR reaction mixture comprised 4 μL of 5 × Fast Pfu buffer, 2 μL of 2.5 mM dNTPs, 0.8 μL of each primer (5 μM), 0.4 μL of FastPfu polymerase, 0.2 μL of BSA, 10 ng of template DNA, and ddH_2_O to a final volume of 20 μL. All raw data were submitted to the Sequence Read Archive (SRA) database with the number SRA:SRP637349.

### Bioinformatics analysis

Sequences were clustered into operational taxonomic units (OTUs) at a 97% sequence identity threshold using UPARSE (version 7.0.1090) (Edgar, 2013). Representative OTU sequences were taxonomically annotated with the RDP Classifier at a 70% confidence threshold (Schubert et al., 2020). The relative abundances of OTU were computed, excluding those below 0.001% of total sequences across all samples by the methodology of (Bokulich and Mills 2013). Multivariate analyses used OTU relative abundance data within the R environment (R Core Team, 2016). A Venn diagram was generated with jvenn (Bardou et al., 2014) to illustrate shared and unique OTUs among soil microbial communities. Bacterial alpha and fungal diversity indices (e.g., Pielou's evenness, Chao1, ACE, Shannon, and Simpson indices) were calculated after rarefaction to the smallest library size. LEfSe analysis was performed to identify differentially abundant taxa (LDA score>4, *P* < 0.05) (Segata et al., 2011). Enzyme function was predicted by PICRUSt2 based on OTU representative sequences (Douglas et al., 2020). Additionally, Redundancy analysis (RDA) analysis explored soil-microorganism relationships at the OTU level using the vegan package in R (v3.3.1) (Wang et al., 2020).

### Nutritional component determination

Nutritional components of *G. leucocontextum* at three growth stages were analyzed, focusing on polysaccharide and triterpenoid contents. Polysaccharide quantification followed the method of (Ye et al. 2018), while triterpenoid acid levels were measured via ultraviolet-visible spectrophotometry (Yan et al., 2017; Chen et al., 2021).

### Statistical analysis

Data were statistically analyzed using Excel. Comparative analysis between soil and fruiting body samples employed t-tests, with the least significant difference method applied, with significance set at *P* < 0.05.

## Results

### Significant alterations in soil physicochemical properties

The physicochemical properties of the soil underwent significant changes during the growth of *G. leucocontextum* (Table 1). Findings indicated that soil without *G. leucocontextum* cultivation was slightly acidic, with a pH value of 6.48. After cultivation, the soil became neutral, with a pH range of 6.78 to 7.11, significantly higher than that of the control soil. Concentrations of total nitrogen (TN), available nitrogen (AN), and organic matter (OM) were highest in the control soil, surpassing those in *G. leucocontextum-*cultivated soils significantly. Specifically, TN and OM levels in the soil at the cap stage decreased to their lowest points, with reductions of 37.30% and 35.80% compared to the control. All AN values in *G. leucocontextum-*cultivated soils remained abundant, ranging from 1.5 to 2.0 g/kg, with the lowest content observed at the primordium stage. Available phosphorus (AP) and available potassium (AK) contents demonstrated an increasing trend during *G. leucocontextum* growth, peaking at maturity and significantly exceeding levels at other growth stages and the control.

**Table 1:** Investigation of soil physicochemical properties.

**TR**	**pH value**	**Total nitrogen content g/kg**	**Available phosphorus content mg/kg**	**Available potassium content mg/kg**	**Available nitrogen content mg/kg**	**Organic matter content g/kg**
GCK	6.48 ± 0.01 c	2.44 ± 0.03 a	16.46 ± 0.74 c	74.98 ± 3.96 b	259.48 ± 0.47 a	54.35 ± 0.83 a
G1p	7.11 ± 0.01 a	1.57 ± 0.03 c	15.55 ± 0.43 c	65.76 ± 3.93 b	187.64 ± 0.36 d	34.93 ± 0.40 c
G1c	6.78 ± 0.02 b	1.53 ± 0.04 c	33.15 ± 0.16 b	65.78 ± 4.02 b	235.46 ± 1.23 b	34.89 ± 0.36 c
G1m	7.10 ± 0.01 a	1.94 ± 0.02 b	42.01 ± 0.35 a	86.36 ± 4.05 a	193.96 ± 1.14 c	36.48 ± 0.26 b

GCK, the soil samples before *G. leucocontextum* cultivation; G1p, the soil samples at primordium emergence stage of *G. leucocontextum*; G1c, the soil samples at cap formation stage of *G. leucocontextum*; G1m, the soil samples at maturity stage of *G. leucocontextum*.

Mean ± standard deviation. Different lower-case letters showed significant difference (*P* < 0.05).

### Microbial community changes in abundance

**Figure 2:**
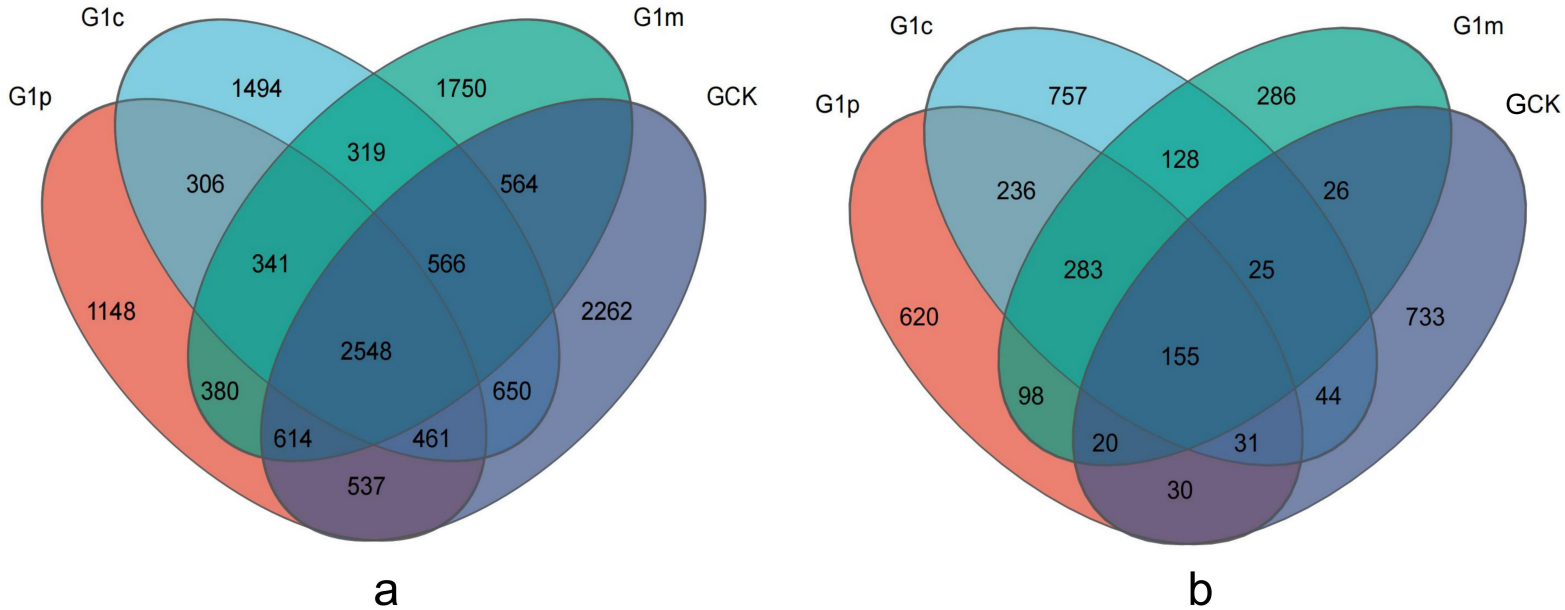
Venn diagram showing the number of shared OTUs between different growth stages of *G. leucocontextum*. A Venn diagram was generated with jvenn to illustrate shared and unique OTUs among soil microbial communities. **(a)**, bacterial Venn diagram; **(b)**, fungal Venn diagram.

A total of 38,105 and 44,417 sequences from 12 samples were assigned to an average of 4,223 bacterial and 840 fungal OTUs at a 97% similarity threshold (Table 2). The control soil exhibited the highest number of bacterial OTUs (4,902), significantly greater than others. Bacterial OTU counts showed a continuous upward trend with *G. leucocontextum* growth. Additionally, the greatest number of fungal OTUs occurred at cap stage (1,026), followed by control soil (899). Fungal OTU counts initially increased and then decreased during *G. leucocontextum* growth, reaching.the lowest level at the mature stage, which differed significantly from others. Across the four soil samples, 2,548 shared bacterial OTUs and 155 shared fungal OTUs were identified (Figure 2). The highest and lowest numbers of unique bacterial OTUs were found in the control (2,262) and primordial (1,148) soils, respectively. The cap soil contained the greatest number of unique fungal OTUs (757), while the soil at the maturity stage had the smallest.

**Table 2:** OTU number investigation.

**Growth stage**	**Bacterial OTU amount**	**Fungal OTU amount**
GCK	4,902 ± 134 a	899 ± 41 ab
G1p	3,767 ± 267 b	827 ± 9 b
G1c	4,016 ± 175 b	1,026 ± 88 a
G1m	4,204 ± 54 b	609 ± 44 c

Mean ± standard deviation. Different lower-case letters showed significant difference (*P* < 0.05) in the OTU number between different treatments.

**Figure 3:**
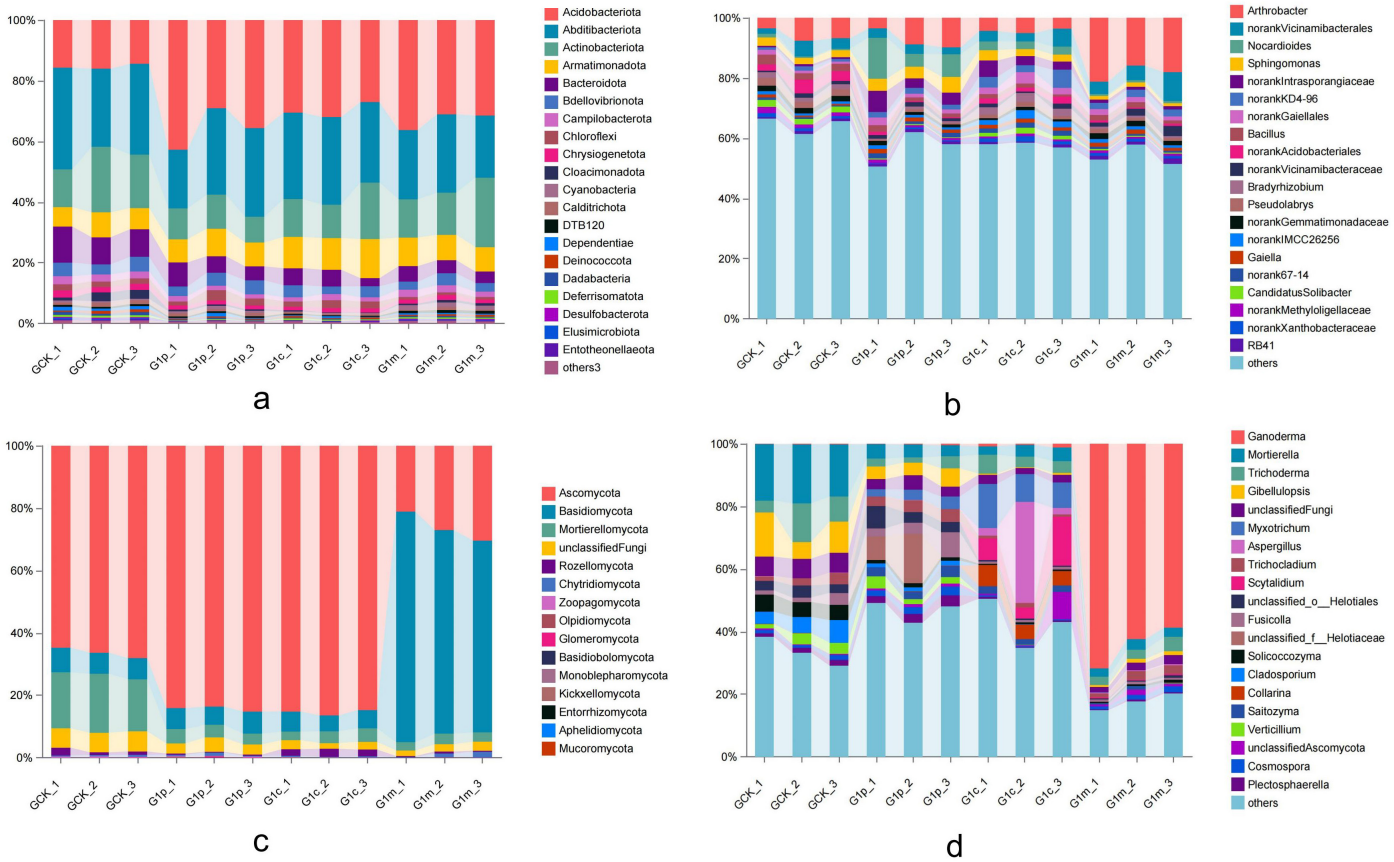
OTU average relative abundances of the microbial phyla and genera in the soil of *G. leucocontextum* during all growth stages. The relative abundances of OTU were computed, excluding those below 0.001% of total sequences across all samples by the methodology of Bokulich and Mills. Multivariate analyses used OTU relative abundance data within the R environment. **(a)**, bacterial phylum abundance; **(b)**, bacterial genus abundance; **(c)**, fungal phylum abundance; **(d)**, fungal genus abundance.

In the study of bacterial communities, 47 phyla and 1,308 genera were identified (Supplementary Tables S1, S2). The top 20 with the highest abundance were presented in Figures 3a, b. Acidobacteriota was the most prevalent phylum, comprising 15.36% to 35.75% (averaging 28.46%) of all bacterial sequences. Abditibacteriota and Actinobacteriota followed, accounting for 26.62% and 14.42% on average, respectively. Among the 20 most abundant phyla, Acidobacteriota and Armatimonadota showed increased abundance during *G. leucocontextum* growth, with Acidobacteriota exhibiting a marked upward trend. However, Bacteroidota, Chrysigenetota, Cloacimonadota, Calditrichota, and Dependentiae displayed significant declines compared to the control sample. At the genus level, *Arthrobacter* was the most prevalent, followed by a Vicinamibacterales-order genus and *Nocardioides*, accounting for average relative abundances of 9.00%, 4.13%, and 3.14%, respectively. Among the top 20 bacterial genera, 35% belonged to Actinobacteriota. Notably, a genus from the Acidobacteriales order exhibited high relative abundance at the cap stage, while *Arthrobacter* and two genera from the Vicinamibacteraceae and Gemmatimonadaceae families showed higher relative abundances at maturity. Furthermore, the Vicinamibacterales-order genus demonstrated a continuous increase in abundance, whereas *Novocardioides, Sphingomonas*, and an Intrasporangiaceae-family genus decreased during *G. leucocontextum* growth.

Significant alterations were also observed in soil fungal communities across different growth stages of *G. leucocontextum* (Supplementary Tables S3, S4). A total of 15 phyla (Figure 3c) and 661 genera were identified, with the top 20 genera depicted in Figure 3d. Ascomycota was the most prevalent phylum, comprising 26.15% to 85.43% (averaging 65.56%). Basidiomycota ranked second with an average abundance of 21.60%. The other three phyla exceeding 1% average abundance were Mortierellomycota, Rozellomycota and an unclassified phylum. Basidiomycota was predominantly abundant at the maturity stage compared to other stages, while Ascomycota abundance decreased and Mortierellomycota and the unclassified phylum significantly declined following *G. leucocontextum* growth. Notably, Basidiobolomycota and Kickxlolomycota were undetected in the control soil and at the maturity stage, respectively. *Ganoderma, Mortierella* and *Trichoderma* were the three most prevalent genera, averaging 16.34%, 7.14%, and 4.61% relative abundance, respectively. *Ganoderma* abundance increased substantially during *G. leucocontextum* growth, rising from 0.13% to 64.26%. However, *Mortierella, Trichoderma, Gibellulopsis* and an unclassified genus progressively decreased in abundance. Specifically, *Mortierella* maintained an abundance six times greater in the control soil than at the maturity stage. Additionally, *Solicocozyma* and *Cladosporium* were more abundant in the control soil, while *Fusicolla*, a Helotiales-order genus, and a Helotiaceae-family genus were more prevalent at the primordium stage. *Aspergillus, Collarina* and *Saitozyma* exhibited higher abundances at the maturity stage. *Myxotrichum* and *Cytalidium* were undetected prior to *G. leucocontextum* cultivation and emerged during its growth.

### Stage-dependent alpha diversity of microbial communities

**Figure 4:**
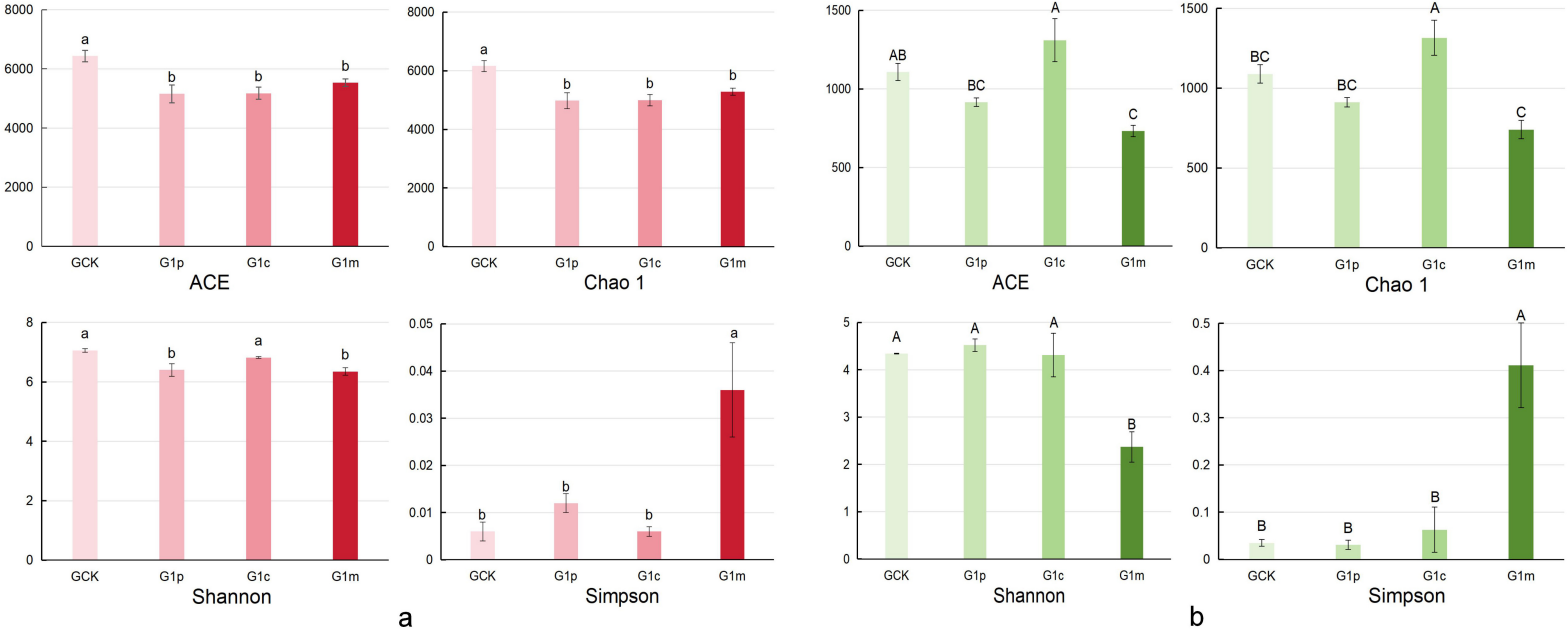
Microbial alpha diversity indices. Bacterial and fungal alpha diversity indices (e.g., Pielou's evenness, Chao1, ACE, Shannon, and Simpson indices) were calculated after rarefaction to the smallest library size. **(a)**, bacterial alpha diversity indices; **(b)**, fungal alpha diversity indices.

Microbial alpha diversity indices varied significantly across different growth stages of *G. leucocontextum* (Supplementary Tables S5, S6). Bacterial richness indices (e.g., ACE and Chao1) were significantly higher in the control soil and increased progressively across the three growth stages (Figure 4a), while fungal richness indices peaked at the cap stage. Indices of ACE and Chao1 were lowest at the maturity stage, differing significantly from the cap stage and the control soil (Figure 4b). Regarding bacterial diversity indices, the Shannon index was significantly elevated in the control soil and at the cap stages but lowest at the maturity stage. The fungal Shannon index peaked at the primordium stage and was notably lower at the maturity stage. The bacterial Pielou's evenness index (Pielou_e) was significantly higher in the control soil compared to the primordium and maturity stages. In contrast, the fungal Pielou_e index was highest at the primordium stage and decreased with *G. leucocontextum* growth.

### Microbial biomarkers significantly enriched at distinct growth stages

**Figure 5:**
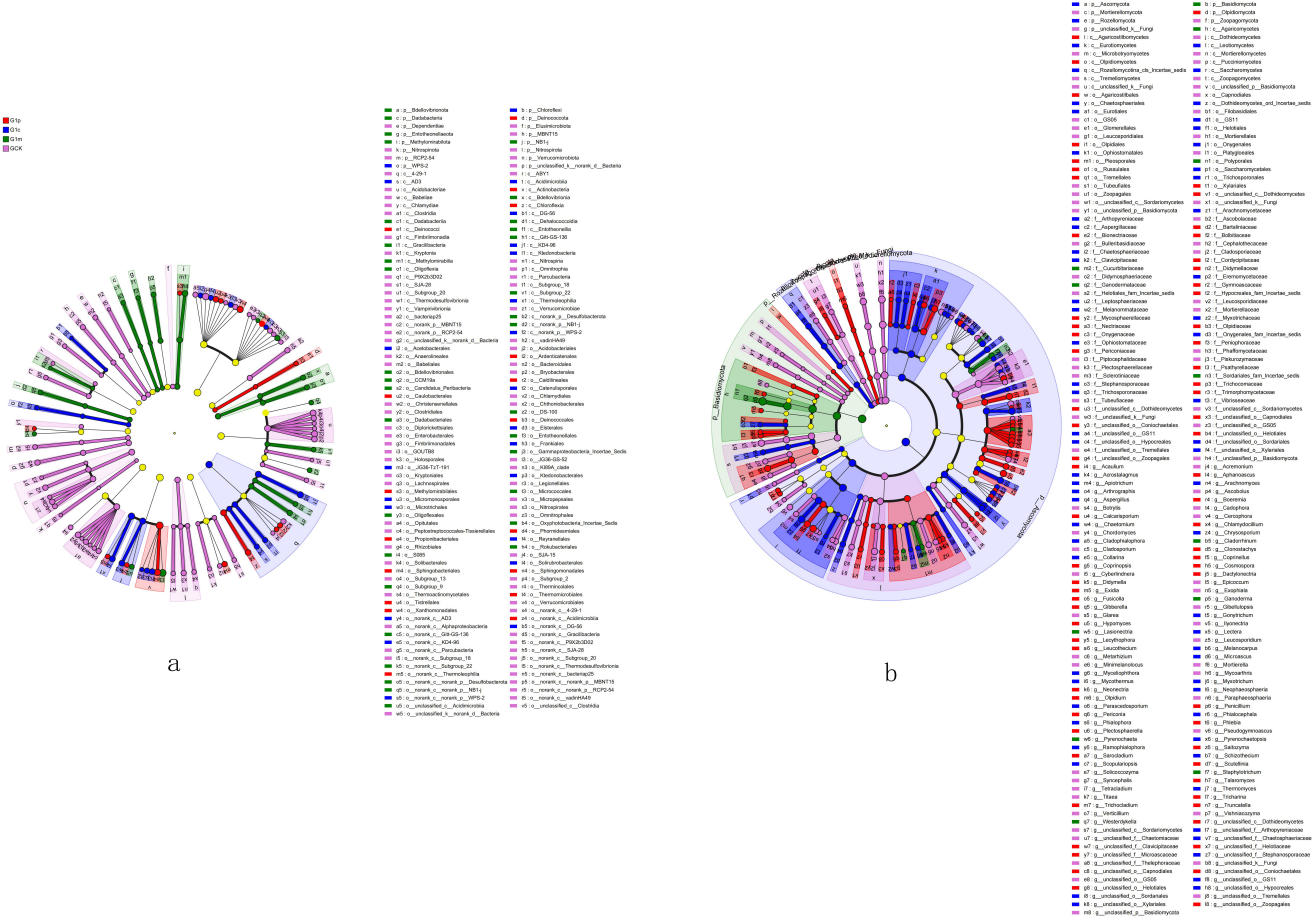
LEfSe analysis. A cladogram was showing the differentially abundant bacterial taxa (**a**, to order level) and fungal taxa (**b**, to genus level) at each of the four growing stages of *G. leucocontextum* based on LEfSe analysis (*P* < 0.05, LDA score > 2).

LEfSe analysis identified differentially abundant bacterial taxa (up to order level) and fungal taxa (up to genus level) across the four growth stages of *G. leucocontextum*. For bacterial taxa (Figure 5a), eight phyla, including Verrucomicrobiota and Nitrospirota, exhibited markedly elevated abundances in control soil relative to other treatments. Five phyla, such as Bdellovibrionota, displayed significantly higher abundance at maturity, while only one phylum showed significantly higher abundance at both primordium and cap stages. Control soil also featured 23 classes with notably increased abundances, including Clostridia and Acidobacteriae. At maturity, 11 classes showed pronounced enrichment. Additionally, control soil demonstrated significantly higher abundances in 47 orders, such as Babeliales and Anaerolineales. Maturity stage displayed elevated abundances in 18 orders, including Bdellovibrionales and Entotheonellales, with 14 orders each enriched at primordium and cap stage. Regarding fungi (Figure 5b), control soil revealed significantly higher abundances of 3 phyla, 12 orders (e.g., Glomerellales), and 33 genera (e.g., *Mortierella*). At the primordium stage, abundances were elevated for 1 phylum, 9 orders, and 38 genera (e.g., *Fusicolla*). Cap stage exhibited increased abundances of 2 phyla (e.g., Ascomycota), 7 orders, and 32 genera (e.g., *Aspergillus*). Abundance declined at maturity, with only 1 phylum (e.g., Basidiomycota), 1 order, and 6 genera (e.g., *Ganoderma*) showing significant enrichment.

### Profiles of microbial enzyme pathways across growth stages

**Figure 6:**
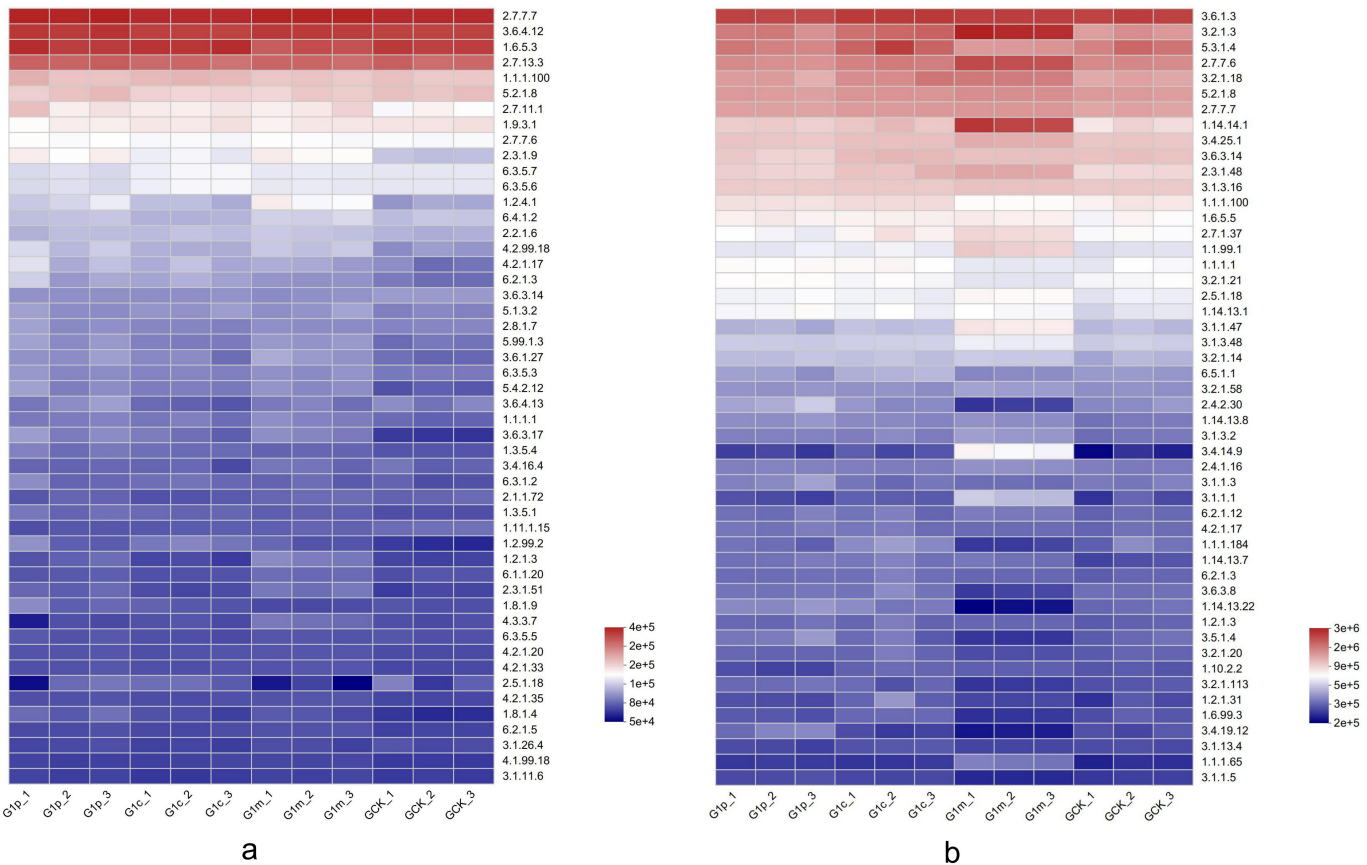
Heatmap of Enzyme. Enzyme function was predicted by PICRUSt2 based on OTU representative sequences. **(a)**, bacteria; **(b)**, fungi.

This study identified 2,442 bacterial and 902 fungal enzyme pathways. DNA-directed DNA polymerase and adenosine triphosphatase were the most prevalent bacterial and fungal enzyme pathways, respectively, across all samples. Bacterial pathways for glutaminyl-tRNA synthase (glutamine-hydrolyzing) and asparaginyl-tRNA synthase (glutamine-hydrolyzing) predominated at the cap stage. Conversely, pyruvate dehydrogenase (acetyl-transferring) dominated at maturity (Figure 6a). Fungal enzyme pathways showed minimal variation among control, primordium, and cap stages. However, maturity stage revealed substantial divergence, with significantly elevated pathways including glucan 1,4-alpha-glucosidase, unspecific monooxygenase, choline dehydrogenase, and tripeptidyl-peptidase I (Figure 6b). Abundance decreased for three fungal enzyme pathways: NAD(+) ADP-ribosyltransferase, cyclohexanone monooxygenase, and ubiquitinyl hydrolase 1, during *G. leucocontextum* growth.

### Stage-specific linkages between soil properties and microbial communities

**Figure 7:**
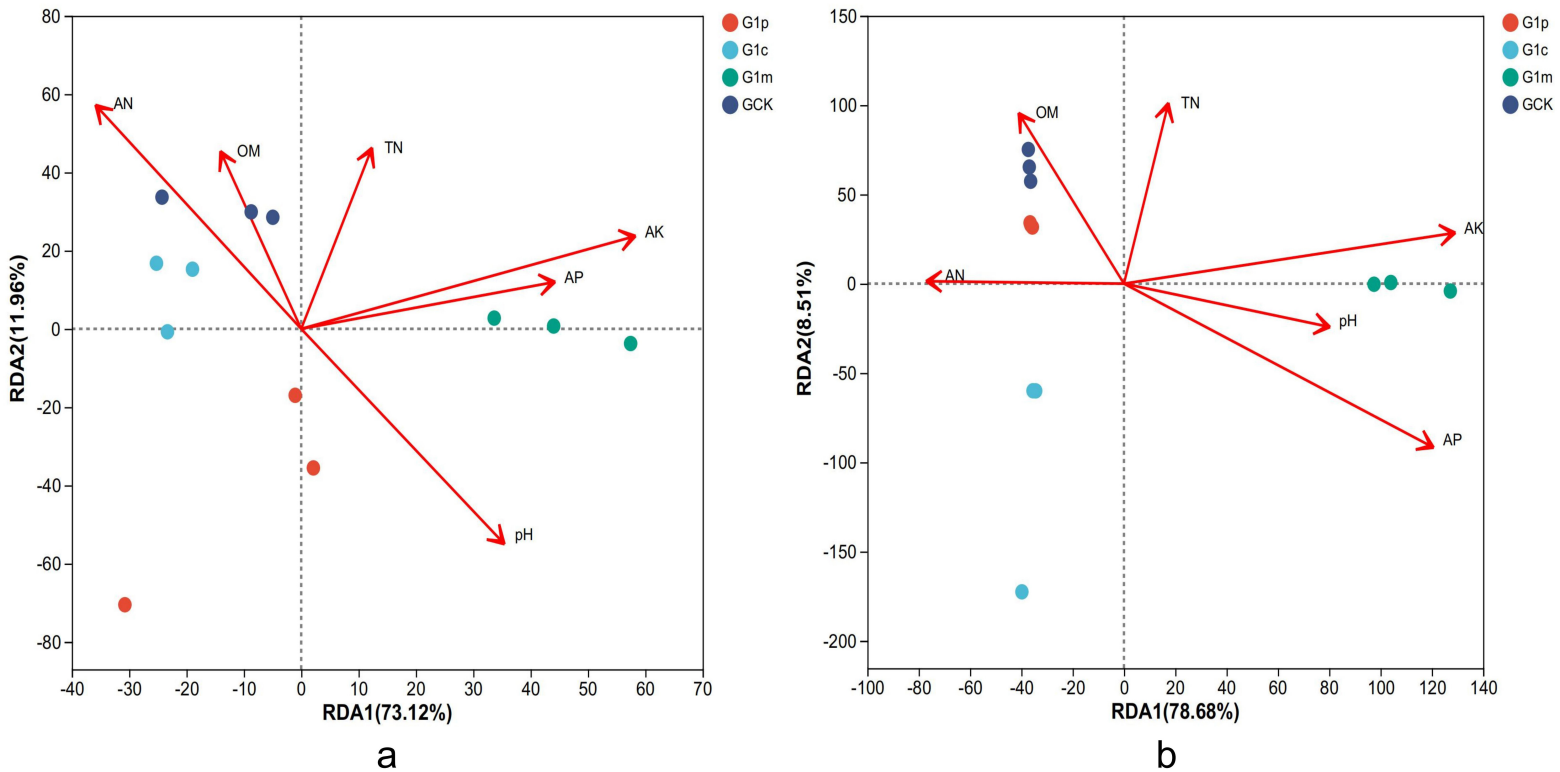
RDA on OTU level. Redundancy analysis (RDA) analysis explored soil-microorganism relationships at the OTU level using the vegan package in R (v3.3.1). **(a)**, bacteria; **(b)**, fungi.

Redundancy analysis elucidated relationship between soil microbial communities and physicochemical properties. In control soil, bacterial communities were predominantly influenced by organic matter (OM), with ammonium nitrogen (AN) and pH as secondary factors (Figure 7a). At primordium stage, OM, pH, and nitrogen contents exerted significant impacts. Soil acidity and AN emerged as primary influencers at cap stage, while available phosphorus (AP) dominated at maturity. Control soil fungal communities were chiefly affected by OM, followed by pH and AP (Figure 7b). AP and pH significantly influenced fungal communities at primordium stage. Total nitrogen (TN) was the main factor at cap stage, and ammonium nitrogen (AN) dominated at maturity, with pH and available potassium (AK) as secondary influences.

### Trends in polysaccharide and triterpenoid acid contents

**Figure 8:**
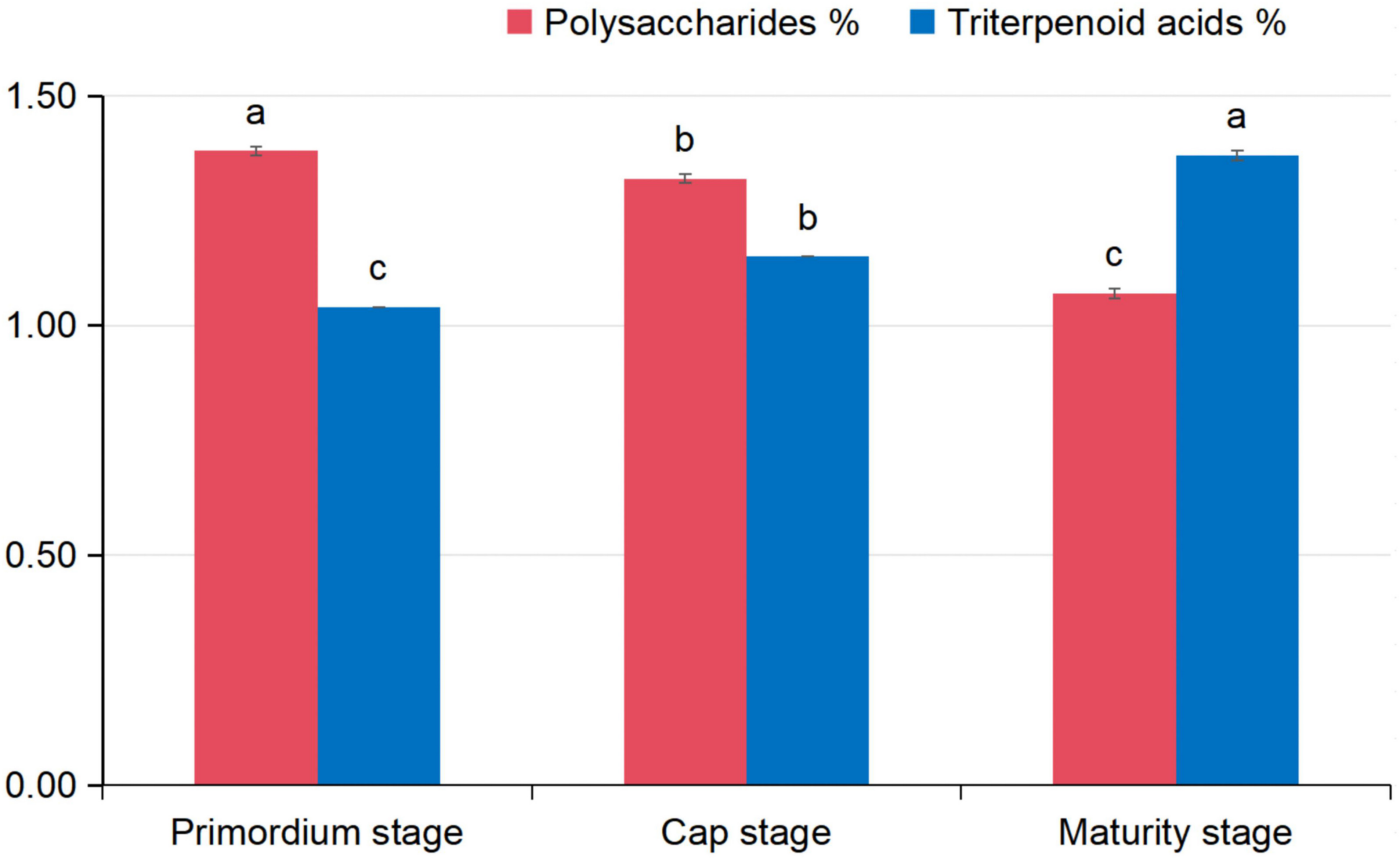
Contents of polysaccharides and triterpenoid acids in *G. leucocontextum* fruiting bodies at different growth stages.

The study documented a declining trend in polysaccharide content (PC) of *G. leucocontextum* fruiting bodies during growth (Figure 8). It peaked at primordium stage (1.38%), substantially exceeding levels at other stages. Conversely, triterpenoid acids content (TAC) increased progressively, reaching a maximum of 1.37% at maturity, significantly higher than at primordium or cap stages.

## Discussion

Soil plays a crucial role in the growth of soil-covered edible fungi, as its physical and chemical properties significantly influence fungal yield and quality. Thoughout the growth cycle, edible fungi and soil interact, dynamically altering the growth environment. Previous research documented reduced humus content alongside elevated pH levels and increased concentrations of carbon (C), nitrogen (N), and trace elements in *G. lucidum* cultivation (Ren et al., 2020). Consistent with these findings, the present study observed a significant pH increase in soil following *G. leucocontextum* cultivation, primarily attributed to lime disinfection. Similarly, (Han et al. 2023) detected heightened rhizosphere soil pH during tea interplanting with *G. lucidum*, which benefited crop growth by mitigating soil acidification in tea gardens. While *G. lucidum* growth typically decomposes substrates and secretes organic acids, decreasing environmental pH by 0.5-0.8 units. And while (Li et al. 2019) reported significant soil pH reduction after interplanting *G. lucidum* in tea gardens. Thus, the difference of pH changes is likely due to added lime amount.

The present study also noted significant alterations in soil nutrient content. Available phosphorus and potassium levels progressively rose, peaking at the maturity stage. Related studies indicate that available phosphorus in deeper soil layer increases during continuous *G. lucidum* cultivation. Likewise, *Oudemansiella radicata* cultivation markedly enhanced available nitrogen, phosphorus, and potassium levels. Additionally, interplanting *G. lucidum* in tea gardens increased organic matter, total nitrogen, available nitrogen, and available phosphorus (Ji et al., 2024; Jiang et al., 2018; Li et al., 2019). Conversely, (Lu et al. 2022) observed declines in most soil properties, including pH, organic matter, available phosphorus, and available potassium after 2 years of *G. lucidum* cultivation. Furthermore, total nitrogen and organic matter significantly decreased during *G. leucocontextum* cultivation, particularly at primordium and cap stages. These findings suggest that mushroom cultivation exerts substantial short-term effects on soil nutrient availability, with impacts varying by cultivated species. Thus, both cultivated species and duration must be considered when evaluating soil health under long-term edible fungi cultivation.

The medicinal value of *G. leucocontextum* stems largely from active ingredients like polysaccharides and triterpenoid acids, which exhibit distinct dynamic patterns during growth. (Baby et al. 2015) noted decreasing polysaccharides and increasing triterpenoid acids during *Ganoderma* maturation, consistent with the present results. Triterpenoids constitute primary bioactive constituents, with composition and content heavily influenced by environmental factors and harvest timing (Dong et al., 2023). Continuous cropping obstacles critically regulate triterpenoid biosynthesis. Previous work demonstrated that continuous cropping reduces total triterpene content in *G. leucocontextum* fruiting bodies (Xie et al., 2021). Cut-log cultivation with casing soil is a standard method for *G. leucocontextum*, ensuring nutrient supply, humidity maintenance, and adequate ventilation. However, prolonged continuous cropping heightens pest and disease incidence, triggers abnormal fruiting body development, and diminishes yield and quality (Chen et al., 2023). These issues correlate strongly with reduced triterpenoid acid levels (Xie et al., 2014; Jin et al., 2016). Continuous cropping obstacles arise from altered soil microbial populations, accumulation of root-derived toxic metabolites, and pathogenic bacteria proliferation (Jiang et al., 2021; Carrasco and Preston, 2020; Yong-Sup et al., 2010). Soil microorganisms are pivotal to ecosystem function and edible fungi development. Microbial community imbalance frequently links to CCOs, driving significant biological, physical, and chemical soil changes. Therefore, investigating soil microbial succession is essential to understanding active ingredients dynamics during *G. leucocontextum* cultivation.

Prior studies confirm strong correlations between CCOs and casing soil microbial shifts during *Ganoderma* cultivation (Wu et al., 2018). Continuously cropped soil exhibits significant bacterial and actinomycete declines alongside progressive fungal increases (Yao et al., 2022). Successive cultivation of edible fungi like *Dictyophora* and *Ganoderma* transitions soil from “bacteria-dominant” to a “fungi-dominant”. Conversely, *Poria cocos* cultivation increases bacterial but reduces actinomycetes and fungi by >50% (Lv, 2012). *G. lucidum* continuous cropping also altered soil bacterial community composition (Yuan et al., 2021), with richness and diversity showing initial increases followed by declines, mirroring current results. Here, control soil (uncultivated) exhibited highest bacterial diversity, while bacterial richness increased with cultivation, and fungal communities peaked at cap stage. Cultivation duration and cultivated fungal species crucially shape microbial dynamics.

Specifically, *G. leucocontextum* cultivation significantly increased *Acidobacteriota* phylum abundance while reducing most other bacterial phyla. (Yuan et al. 2021) reported Proteobacteria and Actinobacteria increases but consistent Chloroflexi declines over successive *G. lucidum* cropping years, with marked divergence between Proteobacteria and Actinobacteria (Ren et al., 2020). At finer taxonomic levels, *Vicinamibacterales* order abundance rose continuously, whereas genera like *Nocardioides* and *Sphingomonas* declined during *G. leucocontextum* growth. *Sphingomonas, Anaeromyxobacter*, and *Bradyrhizobium* genera also decreased annually in *G. lucidum* systems (Yuan et al., 2021), though *Sphingomonas* increased during *G. lucidum*-tea intercropping (Li et al., 2019). As a beneficial bacterium, *Sphingomonas* improves soil physicochemical properties. Dominant genera shifted across growth stages: an *Acidobacteriales*-order genus prevailed at cap stage, whreas *Arthrobacter* and others dominated maturity. According to (Wang et al. 2022), DA101 genus depletion after 2-year *G. lucidum* replanting. Different bacterial genera serve distinct ecological roles during fungal growth (Orlofsky et al., 2021). *Sphingomonas* degrades aromatic compounds, enabling environmental remediation applications. *Arthrobacter* adapts robustly to environments while decomposing complex organics. As a wood-rot fungus, *G. leucocontextum* secretes extracellular enzymes for lignin degradation at maturity, explaining increased *Arthrobacter* abundance linked to substrate breakdown and nutrient cycling (Guan et al., 2023; Ren et al., 2020).

Moreover, fungal communities exhibited significant shifts across various growth stages of *G. leucocontextum*. This study revealed the predominance of Basidiomycota at maturity, whereas the initially dominant phylum Ascomycota declined substantially in abundance. Similarly, Basidiomycota supplanted Ascomycota as the dominant fungi in wood segments following cultivation (Ren et al., 2020). Furthermore, *Ganoderma* emerged as the predominant genus in fungal communities at maturity, while genera such as *Mortierella* and *Trichoderma* showed reduced abundance. As a member of Basidiomycota, the dominant *Ganoderma* colonized the soil surrounding *G. leucocontextum* mycelia, gaining competitive advantages over other fungal communities. Notably, the decline in *Ascomycota* abundance may benefit *G. leucocontextum*'s growth environment. Ascomycota members like *Trichoderma* and *Penicillium* are known pathogenic genera detrimental to *Ganoderma* development (Solomon et al., 2025). Additionally, *Trichoderma* has been implicated in causing brown rot disease in continuous *Ganoderma* cropping systems (Kang et al., 2011). As a macrofungus, *G. leucocontextum* plays a vital role in decomposing and synthesizing organic materials, significantly contributing to soil carbon metabolism and nitrogen cycling. Throughout its growth cycle, microorganisms interact closely with the fungus. Non-*Ganoderma* microbes compete for nutrients and produce secondary metabolites that inhibit mycelial growth, ultimately reducing yield and quality. Thus, isolating and identifying these detrimental microbes is crucial for developing targeted fungicides and effective control measures against CCOs (Lin et al., 2021; Ning et al., 2022).

This study demonstrated that soil microbial communities are influenced by physicochemical properties during different growth stages of *G. leucocontextum*. Specifically, bacterial communities were primarily affected by soil organic matter, pH, and nitrogen content at primordium and cap stages, while available phosphorus became dominant at maturity. Fungal communities were influenced by soil available phosphorus and pH at primordium, but nitrogen content significantly impacted them at cap and maturity stages. Redundancy analysis confirmed significant correlations between fungal/bacterial community structures and physicochemical properties, including pH, nitrogen, carbon, and trace elements (Ren et al., 2020). The interaction between soil microorganisms and physicochemical properties shapes *G. leucocontextum* growth and elucidates underlying mechanisms of CCOs.

As the most diverse soil microbial group, bacteria are highly sensitive to environmental changes. Acidophilic genera like *Acidobacteria* thrive in acidic conditions (Kalam et al., 2020), likely explaining *Acidobacteriota* abundance variations observed here. As oligotrophic bacteria, *Acidobacteriota* adapt well to nutrient-poor environments, highlighting their ecological role in such soils (McReynolds et al., 2024). Conversely, copiotrophic Proteobacteria increase with nitrogen availability (Fierer et al., 2012). Nitrogen critically affects microbial community composition, and its decline here may suppress copiotrophic microorganisms, underscoring nitrogen's complex role in microbial dynamics. Rhizosphere soil properties, especially pH, significantly influence fungal β-diversity (Zhang et al., 2016), indicating pH's crucial role in structuring fungal populations and affecting soil ecosystems during cultivation. Interestingly, *G. leucocontextum* itself influences the soil environment. *Ganoderma* species (e.g., *G. tsugae, G. sinense*, and *G. lucidum*) reportedly enhance soil physical properties by reducing capillary porosity and increasing water retention (Gao, 2018). Thus, bidirectional mushroom-soil interactions mutually improve growth conditions.

## Conclusions

The casing soil environment was systematically analyzed across four *G. leucocontextum* growth stages, revealing dynamic successional patterns in physicochemical properties and microbial communities. Cultivation caused significant soil alkalization (pH 6.78–7.11), while key fertility indicators (e.g., total nitrogen, organic matter) decreased markedly at maturity. Conversely, available phosphorus and potassium peaked at maturity, indicating high demand during late fruiting body development. Soil-dominant microbial groups shifted from bacteria to fungi as cultivation progressed, with bacterial genera like *Arthrobacter* becoming predominant at maturity. Critically, Basidiomycota dominated at maturity, and *Ganoderma* abundance increased substantially throughout growth, confirming successful colonization. Redundancy analysis indicated significant correlations between physicochemical factors and microbial community structure, collectively shaping the microecological environment. Notably, increasing triterpenoid acids and decreasing polysaccharides suggest prolonged cultivation may alter medicinal quality. Collectively, these findings elucidate soil dynamics during *G. leucocontextum* cultivation, providing scientific insights for mitigating CCOs and establishing a theoretical foundation for high-quality, high-yield cultivation through soil microenvironment regulation.

## Data Availability

The data presented in the study are deposited in the NCBI repository, accession number SRA:SRP637349.
