# Supplementary material for: Soil microbial community succession and physicochemical property changes affect Ganoderma leucocontextum growth in the Dadu river basin
Source: Front Microbiol. 2026 Jan 7;16:1666459. doi: 10.3389/fmicb.2025.1666459 (PMC12819783; doi:10.3389/fmicb.2025.1666459)
Supplement: Supplementary file 1 [file Data_Sheet_1.doc]

Supplementary Table 1 Bacterial abundance at phylum level

| #OTU ID | GCK_1 | GCK_2 | GCK_3 | G1p_1 | G1p_2 | G1p_3 |
| --- | --- | --- | --- | --- | --- | --- |
| d__Bacteria;k__norank_d__Bacteria;p__Acidobacteriota | 0.156462407 | 0.16027812 | 0.143922117 | 0.427052063 | 0.289904934 | 0.355519696 |
| d__Bacteria;k__norank_d__Bacteria;p__Abditibacteriota | 0.335651489 | 0.25692734 | 0.298879325 | 0.193884553 | 0.285694885 | 0.292983775 |
| d__Bacteria;k__norank_d__Bacteria;p__Actinobacteriota | 0.124471854 | 0.216620484 | 0.176635033 | 0.101359465 | 0.111747397 | 0.084685441 |
| d__Bacteria;k__norank_d__Bacteria;p__Armatimonadota | 0.064217294 | 0.081768268 | 0.07006057 | 0.076550304 | 0.090765052 | 0.078694903 |
| d__Bacteria;k__norank_d__Bacteria;p__Bacteroidota | 0.118698334 | 0.089285714 | 0.090855587 | 0.079337851 | 0.054753282 | 0.045757991 |
| d__Bacteria;k__norank_d__Bacteria;p__Bdellovibrionota | 0.044823514 | 0.033841338 | 0.048162044 | 0.031220516 | 0.042485287 | 0.046310624 |
| d__Bacteria;k__norank_d__Bacteria;p__Campilobacterota | 0.026112059 | 0.023014163 | 0.022952991 | 0.01852646 | 0.015301041 | 0.013683187 |
| d__Bacteria;k__norank_d__Bacteria;p__Chloroflexi | 0.020679701 | 0.018729475 | 0.017631624 | 0.012801269 | 0.03320507 | 0.023122154 |
| d__Bacteria;k__norank_d__Bacteria;p__Chrysiogenetota | 0.023540218 | 0.016959154 | 0.020083867 | 0.01353032 | 0.013150747 | 0.012268447 |
| d__Bacteria;k__norank_d__Bacteria;p__Cloacimonadota | 0.009316363 | 0.029120484 | 0.029353344 | 0.005575092 | 0.005341784 | 0.005194748 |
| d__Bacteria;k__norank_d__Bacteria;p__Cyanobacteria | 0.008030442 | 0.005695813 | 0.004659261 | 0.006647225 | 0.009913988 | 0.007626332 |
| d__Bacteria;k__norank_d__Bacteria;p__Calditrichota | 0.006797008 | 0.012854064 | 0.012972363 | 0.010485462 | 0.007288366 | 0.009969495 |
| d__Bacteria;k__norank_d__Bacteria;p__DTB120 | 0.006193413 | 0.005567529 | 0.006792712 | 0.004159876 | 0.005749208 | 0.005902118 |
| d__Bacteria;k__norank_d__Bacteria;p__Dependentiae | 0.012360583 | 0.007722701 | 0.010422031 | 0.001994168 | 0.011951109 | 0.002652637 |
| d__Bacteria;k__norank_d__Bacteria;p__Deinococcota | 0.004303897 | 0.009108169 | 0.009048775 | 0.003173514 | 0.003893164 | 0.002652637 |
| d__Bacteria;k__norank_d__Bacteria;p__Dadabacteria | 0.010156147 | 0.006747742 | 0.010029672 | 0.003902565 | 0.004323223 | 0.003426323 |
| d__Bacteria;k__norank_d__Bacteria;p__Deferrisomatota | 0.007059441 | 0.006619458 | 0.007136026 | 0.001586757 | 0.003236759 | 0.001525266 |
| d__Bacteria;k__norank_d__Bacteria;p__Desulfobacterota | 0.002755544 | 0.004438629 | 0.002574855 | 0.000900592 | 0.001901313 | 0.000862107 |
| d__Bacteria;k__norank_d__Bacteria;p__Elusimicrobiota | 0.005196168 | 0.002693966 | 0.003776454 | 0.000922034 | 0.000837483 | 0.000530527 |
| d__Bacteria;k__norank_d__Bacteria;p__Entotheonellaeota | 0.002571841 | 0.002719622 | 0.00333505 | 0.000750493 | 0.00101856 | 0.00070737 |
| d__Bacteria;k__norank_d__Bacteria;p__Fibrobacterota | 0.001180947 | 0.000872332 | 0.002256063 | 0.001458101 | 0.000995926 | 0.001105265 |
| d__Bacteria;k__norank_d__Bacteria;p__FCPU426 | 0.002099462 | 0.001642036 | 0.00152039 | 0.000793379 | 0.001177003 | 0.000596843 |
| d__Bacteria;k__norank_d__Bacteria;p__Fusobacteriota | 0.001732056 | 0.001231527 | 0.00152039 | 0.000300197 | 0.000362155 | 0.000243158 |
| d__Bacteria;k__norank_d__Bacteria;p__Gemmatimonadota | 0.000498622 | 0.00169335 | 0.001177076 | 0.000235869 | 0.000271616 | 8.84E-05 |
| d__Bacteria;k__norank_d__Bacteria;p__Firmicutes | 0.000944758 | 0.000718391 | 0.000490449 | 0.000257312 | 0.000701675 | 0.00037579 |
| d__Bacteria;k__norank_d__Bacteria;p__Methylomirabilota | 0.000183703 | 0.000205255 | 0.000343314 | 0 | 4.53E-05 | 4.42E-05 |
| d__Bacteria;k__norank_d__Bacteria;p__Hydrogenedentes | 0.000446136 | 0.00064142 | 0.000588538 | 0.000471739 | 0.000611136 | 0.000486317 |
| d__Bacteria;k__norank_d__Bacteria;p__Latescibacterota | 0.000314919 | 5.13E-05 | 4.90E-05 | 0.000235869 | 0.000384789 | 0.000309474 |
| d__Bacteria;k__norank_d__Bacteria;p__GAL15 | 0.00015746 | 0.000359195 | 0.000171657 | 0.000385968 | 0.000588502 | 0.000552633 |
| d__Bacteria;k__norank_d__Bacteria;p__Myxococcota | 7.87E-05 | 7.70E-05 | 0 | 0.000879149 | 0.00067904 | 0.001304213 |
| d__Bacteria;k__norank_d__Bacteria;p__Halanaerobiaeota | 0.000367406 | 0.000436166 | 0.000465926 | 0.000150099 | 0.000407424 | 0.000198948 |
| d__Bacteria;k__norank_d__Bacteria;p__Margulisbacteria | 0.00015746 | 0.000307882 | 0.000245224 | 2.14E-05 | 0.000248981 | 0.000110527 |
| d__Bacteria;k__norank_d__Bacteria;p__MBNT15 | 0.000446136 | 0.000282225 | 0.000441404 | 6.43E-05 | 0.000158443 | 0.000287369 |
| d__Bacteria;k__norank_d__Bacteria;p__Nitrospirota | 0.000734812 | 0.000128284 | 0.000367836 | 0 | 0 | 2.21E-05 |
| d__Bacteria;k__norank_d__Bacteria;p__NB1-j | 0.00015746 | 0.000102627 | 0.000171657 | 4.29E-05 | 6.79E-05 | 0 |
| d__Bacteria;k__norank_d__Bacteria;p__Poribacteria | 0.000183703 | 7.70E-05 | 4.90E-05 | 6.43E-05 | 0.000158443 | 2.21E-05 |
| d__Bacteria;k__norank_d__Bacteria;p__RCP2-54 | 0.000183703 | 0.000128284 | 2.45E-05 | 4.29E-05 | 0.000248981 | 2.21E-05 |
| d__Bacteria;k__norank_d__Bacteria;p__Planctomycetota | 2.62E-05 | 7.70E-05 | 0.000343314 | 2.14E-05 | 0 | 0 |
| d__Bacteria;k__norank_d__Bacteria;p__Proteobacteria | 0.000131216 | 0 | 4.90E-05 | 6.43E-05 | 0.000113173 | 6.63E-05 |
| d__Bacteria;k__norank_d__Bacteria;p__Spirochaetota | 0.00015746 | 0.000102627 | 4.90E-05 | 8.58E-05 | 6.79E-05 | 8.84E-05 |
| d__Bacteria;k__norank_d__Bacteria;p__SAR324_cladeMarine_group_B | 0 | 5.13E-05 | 0.000122612 | 0 | 9.05E-05 | 0 |
| d__Bacteria;k__norank_d__Bacteria;p__Nitrospinota | 5.25E-05 | 0 | 2.45E-05 | 2.14E-05 | 6.79E-05 | 0 |
| d__Bacteria;k__norank_d__Bacteria;p__Sumerlaeota | 7.87E-05 | 2.57E-05 | 4.90E-05 | 0 | 9.05E-05 | 0 |
| d__Bacteria;k__norank_d__Bacteria;p__Schekmanbacteria | 0.000131216 | 5.13E-05 | 0 | 4.29E-05 | 0 | 0 |
| d__Bacteria;k__norank_d__Bacteria;p__Sva0485 | 0 | 2.57E-05 | 0.000122612 | 0 | 0 | 0 |
| d__Bacteria;k__norank_d__Bacteria;p__Patescibacteria | 2.62E-05 | 0 | 7.36E-05 | 0 | 0 | 0 |
| d__Bacteria;k__norank_d__Bacteria;p__Synergistota | 0.000131216 | 0 | 0 | 0 | 0 | 0 |
| d__Bacteria;k__norank_d__Bacteria;p__TX1A-33 | 0 | 0 | 0 | 0 | 0 | 0 |
| d__Bacteria;k__norank_d__Bacteria;p__Verrucomicrobiota | 0 | 0 | 0 | 0 | 0 | 0 |
| d__Bacteria;k__norank_d__Bacteria;p__WPS-2 | 0 | 0 | 0 | 0 | 0 | 0 |
| d__Bacteria;k__norank_d__Bacteria;p__WS2 | 0 | 0 | 0 | 0 | 0 | 0 |
| d__Bacteria;k__norank_d__Bacteria;p__WS4 | 0 | 0 | 0 | 0 | 0 | 0 |
| d__Bacteria;k__norank_d__Bacteria;p__Zixibacteria | 0 | 0 | 0 | 0 | 0 | 0 |
| d__Bacteria;k__norank_d__Bacteria;p__unclassified_k__norank_d__Bacteria | 0 | 0 | 0 | 0 | 0 | 0 |

| #OTU ID | G1c_1 | G1c_2 | G1c_3 | G1m_1 | G1m_2 | G1m_3 |
| --- | --- | --- | --- | --- | --- | --- |
| d__Bacteria;k__norank_d__Bacteria;p__Acidobacteriota | 0.304996603 | 0.319616232 | 0.270371982 | 0.36217058 | 0.310262825 | 0.314476901 |
| d__Bacteria;k__norank_d__Bacteria;p__Abditibacteriota | 0.284874301 | 0.288318423 | 0.265533396 | 0.228390034 | 0.258614283 | 0.204889976 |
| d__Bacteria;k__norank_d__Bacteria;p__Actinobacteriota | 0.124941201 | 0.110592506 | 0.185914339 | 0.126137997 | 0.138893706 | 0.228979539 |
| d__Bacteria;k__norank_d__Bacteria;p__Armatimonadota | 0.102911201 | 0.104187735 | 0.128824147 | 0.094226162 | 0.083529441 | 0.080684597 |
| d__Bacteria;k__norank_d__Bacteria;p__Bacteroidota | 0.056107249 | 0.055257358 | 0.026701825 | 0.05110206 | 0.043077609 | 0.037343971 |
| d__Bacteria;k__norank_d__Bacteria;p__Bdellovibrionota | 0.040140072 | 0.025463503 | 0.035918179 | 0.026449449 | 0.039510515 | 0.028593489 |
| d__Bacteria;k__norank_d__Bacteria;p__Campilobacterota | 0.013118695 | 0.019706988 | 0.014157344 | 0.024652611 | 0.023309966 | 0.018221593 |
| d__Bacteria;k__norank_d__Bacteria;p__Chloroflexi | 0.017927142 | 0.026008038 | 0.022887279 | 0.007930043 | 0.009214992 | 0.007618067 |
| d__Bacteria;k__norank_d__Bacteria;p__Chrysiogenetota | 0.011812052 | 0.014313497 | 0.013466117 | 0.01360805 | 0.016151007 | 0.012405096 |
| d__Bacteria;k__norank_d__Bacteria;p__Cloacimonadota | 0.003946062 | 0.001322443 | 0.001587261 | 0.004719693 | 0.007951646 | 0.007540857 |
| d__Bacteria;k__norank_d__Bacteria;p__Cyanobacteria | 0.002273559 | 0.003345002 | 0.003174522 | 0.015740297 | 0.015952835 | 0.01299704 |
| d__Bacteria;k__norank_d__Bacteria;p__Calditrichota | 0.00512204 | 0.004771166 | 0.005939428 | 0.003186392 | 0.008992048 | 0.004838502 |
| d__Bacteria;k__norank_d__Bacteria;p__DTB120 | 0.004128992 | 0.003007909 | 0.00363534 | 0.007426929 | 0.009982908 | 0.00893064 |
| d__Bacteria;k__norank_d__Bacteria;p__Dependentiae | 0.002665552 | 0.006041748 | 0.002406492 | 0.00553426 | 0.004136838 | 0.003268563 |
| d__Bacteria;k__norank_d__Bacteria;p__Deinococcota | 0.006088956 | 0.00207442 | 0.004121758 | 0.003593675 | 0.005474498 | 0.006485652 |
| d__Bacteria;k__norank_d__Bacteria;p__Dadabacteria | 0.002169027 | 0.003267211 | 0.001971276 | 0.004432199 | 0.005375412 | 0.00344872 |
| d__Bacteria;k__norank_d__Bacteria;p__Deferrisomatota | 0.005409502 | 0.004434072 | 0.002790507 | 0.005007187 | 0.004756125 | 0.004658345 |
| d__Bacteria;k__norank_d__Bacteria;p__Desulfobacterota | 0.00146344 | 0.001555815 | 0.003584138 | 0.002683277 | 0.002353291 | 0.00360314 |
| d__Bacteria;k__norank_d__Bacteria;p__Elusimicrobiota | 0.000418126 | 0.000959419 | 0.001177645 | 0.002563488 | 0.001956947 | 0.002547935 |
| d__Bacteria;k__norank_d__Bacteria;p__Entotheonellaeota | 0.001385042 | 0.001348373 | 0.001203246 | 0.001724964 | 0.001089945 | 0.001003732 |
| d__Bacteria;k__norank_d__Bacteria;p__Fibrobacterota | 0.002717817 | 0.000881628 | 0.000486419 | 0.001006229 | 0.001610146 | 0.000926522 |
| d__Bacteria;k__norank_d__Bacteria;p__FCPU426 | 0.00036586 | 0.000129651 | 0.000460818 | 0.002060374 | 0.001783547 | 0.001415519 |
| d__Bacteria;k__norank_d__Bacteria;p__Fusobacteriota | 0.000470391 | 0.000518605 | 0.000537621 | 0.000598946 | 0.000718373 | 0.000566208 |
| d__Bacteria;k__norank_d__Bacteria;p__Gemmatimonadota | 0.000627189 | 0.000466744 | 0.00079363 | 0.000311452 | 0.000470658 | 0.000488998 |
| d__Bacteria;k__norank_d__Bacteria;p__Firmicutes | 0.000130664 | 0.000129651 | 5.12E-05 | 0.001485386 | 0.000569744 | 0.000720628 |
| d__Bacteria;k__norank_d__Bacteria;p__Methylomirabilota | 0.001620237 | 0.001244652 | 0.001254448 | 0.000383325 | 0.000371572 | 0.000386051 |
| d__Bacteria;k__norank_d__Bacteria;p__Hydrogenedentes | 0.000574923 | 0.000285233 | 0.000204808 | 0.000191663 | 0.000322029 | 0.000566208 |
| d__Bacteria;k__norank_d__Bacteria;p__Latescibacterota | 0.000496524 | 0.000103721 | 2.56E-05 | 0.000814566 | 0.001114717 | 0.000488998 |
| d__Bacteria;k__norank_d__Bacteria;p__GAL15 | 0.000104531 | 0.000233372 | 0.000460818 | 0.000167705 | 0.000371572 | 0.000643418 |
| d__Bacteria;k__norank_d__Bacteria;p__Myxococcota | 0.000209063 | 0.000103721 | 0.000102404 | 7.19E-05 | 2.48E-05 | 0.000102947 |
| d__Bacteria;k__norank_d__Bacteria;p__Halanaerobiaeota | 0.00018293 | 2.59E-05 | 0 | 0.000311452 | 0.000544973 | 0.000205894 |
| d__Bacteria;k__norank_d__Bacteria;p__Margulisbacteria | 0.000209063 | 0 | 2.56E-05 | 0.000694777 | 0.000569744 | 0.000411787 |
| d__Bacteria;k__norank_d__Bacteria;p__MBNT15 | 0.00018293 | 0.000181512 | 0.000179207 | 0.00033541 | 0.000222943 | 0.000205894 |
| d__Bacteria;k__norank_d__Bacteria;p__Nitrospirota | 2.61E-05 | 0 | 0 | 9.58E-05 | 0 | 0 |
| d__Bacteria;k__norank_d__Bacteria;p__NB1-j | 2.61E-05 | 0 | 0 | 2.40E-05 | 0.000470658 | 0.00015442 |
| d__Bacteria;k__norank_d__Bacteria;p__Poribacteria | 0 | 0 | 2.56E-05 | 2.40E-05 | 4.95E-05 | 0 |
| d__Bacteria;k__norank_d__Bacteria;p__RCP2-54 | 0 | 0 | 0 | 0 | 0 | 0 |
| d__Bacteria;k__norank_d__Bacteria;p__Planctomycetota | 0 | 2.59E-05 | 0 | 0 | 7.43E-05 | 2.57E-05 |
| d__Bacteria;k__norank_d__Bacteria;p__Proteobacteria | 0 | 5.19E-05 | 0 | 0 | 0 | 0.000102947 |
| d__Bacteria;k__norank_d__Bacteria;p__Spirochaetota | 0 | 0 | 0 | 2.40E-05 | 0 | 0 |
| d__Bacteria;k__norank_d__Bacteria;p__SAR324_cladeMarine_group_B | 7.84E-05 | 0 | 2.56E-05 | 0 | 7.43E-05 | 0 |
| d__Bacteria;k__norank_d__Bacteria;p__Nitrospinota | 2.61E-05 | 0 | 0 | 9.58E-05 | 2.48E-05 | 0 |
| d__Bacteria;k__norank_d__Bacteria;p__Sumerlaeota | 0 | 0 | 0 | 0 | 0 | 5.15E-05 |
| d__Bacteria;k__norank_d__Bacteria;p__Schekmanbacteria | 0 | 0 | 0 | 2.40E-05 | 2.48E-05 | 0 |
| d__Bacteria;k__norank_d__Bacteria;p__Sva0485 | 0 | 2.59E-05 | 0 | 0 | 0 | 0 |
| d__Bacteria;k__norank_d__Bacteria;p__Patescibacteria | 5.23E-05 | 0 | 0 | 0 | 0 | 0 |
| d__Bacteria;k__norank_d__Bacteria;p__Synergistota | 0 | 0 | 0 | 0 | 0 | 0 |
| d__Bacteria;k__norank_d__Bacteria;p__TX1A-33 | 0 | 0 | 0 | 0 | 0 | 0 |
| d__Bacteria;k__norank_d__Bacteria;p__Verrucomicrobiota | 0 | 0 | 0 | 0 | 0 | 0 |
| d__Bacteria;k__norank_d__Bacteria;p__WPS-2 | 0 | 0 | 0 | 0 | 0 | 0 |
| d__Bacteria;k__norank_d__Bacteria;p__WS2 | 0 | 0 | 0 | 0 | 0 | 0 |
| d__Bacteria;k__norank_d__Bacteria;p__WS4 | 0 | 0 | 0 | 0 | 0 | 0 |
| d__Bacteria;k__norank_d__Bacteria;p__Zixibacteria | 0 | 0 | 0 | 0 | 0 | 0 |
| d__Bacteria;k__norank_d__Bacteria;p__unclassified_k__norank_d__Bacteria | 0 | 0 | 0 | 0 | 0 | 0 |
