# Supplementary material for: Soil microbial community succession and physicochemical property changes affect Ganoderma leucocontextum growth in the Dadu river basin
Source: Front Microbiol. 2026 Jan 7;16:1666459. doi: 10.3389/fmicb.2025.1666459 (PMC12819783; doi:10.3389/fmicb.2025.1666459)
Supplement: Supplementary file 2 [file Data_Sheet_2.doc]

Supplementary Table 2 Bacterial abundance at phylum level

| #OTU ID | GCK_1 | GCK_2 | GCK_3 | G1p_1 | G1p_2 | G1p_3 | G1c_1 | G1c_2 | G1c_3 | G1m_1 | G1m_2 | G1m_3 |
| --- | --- | --- | --- | --- | --- | --- | --- | --- | --- | --- | --- | --- |
| d__Bacteria;k__norank_d__Bacteria;p__Actinobacteriota;c__Actinobacteria;o__Micrococcales;f__Micrococcaceae;g__Arthrobacter | 0.034404934 | 0.07635468 | 0.067951642 | 0.034822884 | 0.088388411 | 0.097860206 | 0.043432812 | 0.050719564 | 0.035508564 | 0.212146622 | 0.158240234 | 0.180748938 |
| d__Bacteria;k__norank_d__Bacteria;p__Acidobacteriota;c__Vicinamibacteria;o__Vicinamibacterales;f__norank_o__Vicinamibacterales;g__norank_f__norank_o__Vicinamibacterales | 0.017766697 | 0.051980706 | 0.034723755 | 0.030641564 | 0.030986872 | 0.022657942 | 0.034286312 | 0.027408272 | 0.059573488 | 0.041015812 | 0.049196165 | 0.095869257 |
| d__Bacteria;k__norank_d__Bacteria;p__Actinobacteriota;c__Actinobacteria;o__Propionibacteriales;f__Nocardioidaceae;g__Nocardioides | 0.013147881 | 0.004515599 | 0.003899066 | 0.137125826 | 0.042598461 | 0.076086476 | 0.029451733 | 0.025359782 | 0.027009037 | 0.005414471 | 0.006093785 | 0.006125338 |
| d__Bacteria;k__norank_d__Bacteria;p__Proteobacteria;c__Alphaproteobacteria;o__Sphingomonadales;f__Sphingomonadaceae;g__Sphingomonas | 0.026873114 | 0.020576765 | 0.02395841 | 0.039668925 | 0.03881847 | 0.051925372 | 0.03454764 | 0.022870478 | 0.022605668 | 0.012362242 | 0.014664718 | 0.009831425 |
| d__Bacteria;k__norank_d__Bacteria;p__Actinobacteriota;c__Actinobacteria;o__Micrococcales;f__Intrasporangiaceae;g__unclassified_f__Intrasporangiaceae | 0.005406115 | 0.007543103 | 0.006277741 | 0.070010292 | 0.031507469 | 0.039745347 | 0.053572362 | 0.030623622 | 0.027469855 | 0.011859128 | 0.010205851 | 0.011143997 |
| d__Bacteria;k__norank_d__Bacteria;p__Chloroflexi;c__KD4-96;o__norank_c__KD4-96;f__norank_o__norank_c__KD4-96;g__norank_f__norank_o__norank_c__KD4-96 | 0.008450335 | 0.013546798 | 0.00757743 | 0.018740887 | 0.019714803 | 0.016335824 | 0.036063346 | 0.023648386 | 0.059650291 | 0.019932918 | 0.024771483 | 0.023343199 |
| d__Bacteria;k__norank_d__Bacteria;p__Actinobacteriota;c__Thermoleophilia;o__Gaiellales;f__norank_o__Gaiellales;g__norank_f__norank_o__Gaiellales | 0.015903425 | 0.00869766 | 0.008043356 | 0.025173686 | 0.011226799 | 0.012644237 | 0.021768672 | 0.037158045 | 0.022605668 | 0.01914231 | 0.016175778 | 0.01299704 |
| d__Bacteria;k__norank_d__Bacteria;p__Firmicutes;c__Bacilli;o__Bacillales;f__Bacillaceae;g__Bacillus | 0.032856581 | 0.020935961 | 0.023762231 | 0.022643451 | 0.013354459 | 0.012644237 | 0.014242408 | 0.013016984 | 0.007065871 | 0.018926689 | 0.019148356 | 0.011916098 |
| d__Bacteria;k__norank_d__Bacteria;p__Acidobacteriota;c__Acidobacteriae;o__Acidobacteriales;f__norank_o__Acidobacteriales;g__norank_f__norank_o__Acidobacteriales | 0.020469755 | 0.046900657 | 0.033988082 | 0.010399691 | 0.003372567 | 0.003138954 | 0.017927142 | 0.014313497 | 0.022631269 | 0.006181121 | 0.003517551 | 0.006151075 |
| d__Bacteria;k__norank_d__Bacteria;p__Acidobacteriota;c__Vicinamibacteria;o__Vicinamibacterales;f__Vicinamibacteraceae;g__norank_f__Vicinamibacteraceae | 0.005458601 | 0.014059934 | 0.010152284 | 0.011321726 | 0.014440923 | 0.009947389 | 0.011263262 | 0.004278491 | 0.017613476 | 0.013919502 | 0.022665907 | 0.034023935 |
| d__Bacteria;k__norank_d__Bacteria;p__Proteobacteria;c__Alphaproteobacteria;o__Rhizobiales;f__Xanthobacteraceae;g__Bradyrhizobium | 0.018973888 | 0.013136289 | 0.015228426 | 0.003066301 | 0.008691716 | 0.005570538 | 0.024904615 | 0.029560482 | 0.023757712 | 0.00773838 | 0.007456216 | 0.007103333 |
| d__Bacteria;k__norank_d__Bacteria;p__Proteobacteria;c__Alphaproteobacteria;o__Rhizobiales;f__Xanthobacteraceae;g__Pseudolabrys | 0.025665923 | 0.021833949 | 0.024252679 | 0.005060468 | 0.008850158 | 0.005305274 | 0.011001934 | 0.016465707 | 0.011853255 | 0.014326785 | 0.009958136 | 0.009522584 |
| d__Bacteria;k__norank_d__Bacteria;p__Gemmatimonadota;c__Gemmatimonadetes;o__Gemmatimonadales;f__Gemmatimonadaceae;g__norank_f__Gemmatimonadaceae | 0.017294318 | 0.017036125 | 0.017435445 | 0.014345141 | 0.010819375 | 0.009394757 | 0.006899075 | 0.011616751 | 0.006937866 | 0.019597508 | 0.017959325 | 0.013717668 |
| d__Bacteria;k__norank_d__Bacteria;p__Actinobacteriota;c__Acidimicrobiia;o__IMCC26256;f__norank_o__IMCC26256;g__norank_f__norank_o__IMCC26256 | 0.011599528 | 0.007543103 | 0.008460237 | 0.012007891 | 0.008488004 | 0.007338963 | 0.016228506 | 0.027330481 | 0.018816723 | 0.012026833 | 0.011122396 | 0.008827693 |
| d__Bacteria;k__norank_d__Bacteria;p__Actinobacteriota;c__Thermoleophilia;o__Gaiellales;f__Gaiellaceae;g__Gaiella | 0.00913266 | 0.004695197 | 0.004585694 | 0.015374389 | 0.012222725 | 0.012069499 | 0.011001934 | 0.013743031 | 0.011904457 | 0.013751797 | 0.013550001 | 0.010449106 |
| d__Bacteria;k__norank_d__Bacteria;p__Actinobacteriota;c__Thermoleophilia;o__Solirubrobacterales;f__67-14;g__norank_f__67-14 | 0.008922714 | 0.006234606 | 0.004634738 | 0.016382194 | 0.009121775 | 0.013395818 | 0.01562745 | 0.016595358 | 0.01587261 | 0.00697173 | 0.007059873 | 0.00571355 |
| d__Bacteria;k__norank_d__Bacteria;p__Acidobacteriota;c__Acidobacteriae;o__Solibacterales;f__Solibacteraceae;g__Candidatus_Solibacter | 0.022831649 | 0.018344622 | 0.019348194 | 0.003194957 | 0.004051607 | 0.002608427 | 0.011289395 | 0.019758849 | 0.013056501 | 0.004216579 | 0.002625777 | 0.003809034 |
| d__Bacteria;k__norank_d__Bacteria;p__Proteobacteria;c__Alphaproteobacteria;o__Rhizobiales;f__Methyloligellaceae;g__norank_f__Methyloligellaceae | 0.021703189 | 0.012546182 | 0.011672675 | 0.012672613 | 0.006722499 | 0.00711791 | 0.006088956 | 0.008531051 | 0.005376206 | 0.009032103 | 0.00718373 | 0.00712907 |
| d__Bacteria;k__norank_d__Bacteria;p__Proteobacteria;c__Alphaproteobacteria;o__Rhizobiales;f__Xanthobacteraceae;g__norank_f__Xanthobacteraceae | 0.012727988 | 0.00862069 | 0.008803551 | 0.002444464 | 0.006179267 | 0.004708431 | 0.012961898 | 0.018177104 | 0.012134866 | 0.010325827 | 0.008719562 | 0.008261485 |
| d__Bacteria;k__norank_d__Bacteria;p__Acidobacteriota;c__Blastocatellia;o__Pyrinomonadales;f__Pyrinomonadaceae;g__RB41 | 0.005143682 | 0.010647578 | 0.008018833 | 0.008898705 | 0.009868719 | 0.009041072 | 0.006611613 | 0.002981978 | 0.009062748 | 0.011164351 | 0.010651738 | 0.018015699 |
| d__Bacteria;k__norank_d__Bacteria;p__Chloroflexi;c__Chloroflexia;o__Thermomicrobiales;f__JG30-KF-CM45;g__norank_f__JG30-KF-CM45 | 0.003569085 | 0.003591954 | 0.003482184 | 0.013187237 | 0.012788592 | 0.012754764 | 0.011472325 | 0.009127447 | 0.00990758 | 0.009056061 | 0.007381902 | 0.006048128 |
| d__Bacteria;k__norank_d__Bacteria;p__Proteobacteria;c__Gammaproteobacteria;o__Burkholderiales;f__SC-I-84;g__norank_f__SC-I-84 | 0.008555308 | 0.007620074 | 0.009073298 | 0.004545844 | 0.007333635 | 0.005835802 | 0.008048921 | 0.011409309 | 0.010957221 | 0.009343555 | 0.009561792 | 0.008518852 |
| d__Bacteria;k__norank_d__Bacteria;p__Acidobacteriota;c__Holophagae;o__Subgroup_7;f__norank_o__Subgroup_7;g__norank_f__norank_o__Subgroup_7 | 0.011468311 | 0.008723317 | 0.009465656 | 0.009563427 | 0.009846084 | 0.007935806 | 0.006846809 | 0.007779074 | 0.00675866 | 0.008840441 | 0.006638757 | 0.006125338 |
| d__Bacteria;k__norank_d__Bacteria;p__Actinobacteriota;c__MB-A2-108;o__norank_c__MB-A2-108;f__norank_o__norank_c__MB-A2-108;g__norank_f__norank_o__norank_c__MB-A2-108 | 0.00456633 | 0.00572147 | 0.003948111 | 0.010935758 | 0.006201901 | 0.006476856 | 0.010845137 | 0.004745235 | 0.009600369 | 0.010158122 | 0.00968565 | 0.011684468 |
| d__Bacteria;k__norank_d__Bacteria;p__Patescibacteria;c__Saccharimonadia;o__Saccharimonadales;f__norank_o__Saccharimonadales;g__norank_f__norank_o__Saccharimonadales | 0.0107335 | 0.009056856 | 0.007258638 | 0.006132601 | 0.0230421 | 0.013307396 | 0.003815397 | 0.005834306 | 0.004633778 | 0.001844753 | 0.002749635 | 0.002342041 |
| d__Bacteria;k__norank_d__Bacteria;p__Proteobacteria;c__Gammaproteobacteria;o__Xanthomonadales;f__Xanthomonadaceae;g__Lysobacter | 0.004592573 | 0.004079433 | 0.003825498 | 0.007697916 | 0.013716614 | 0.02926743 | 0.007865991 | 0.003837677 | 0.00442897 | 0.004000958 | 0.003715722 | 0.003062669 |
| d__Bacteria;k__norank_d__Bacteria;p__Acidobacteriota;c__Acidobacteriae;o__Bryobacterales;f__Bryobacteraceae;g__Bryobacter | 0.011678257 | 0.012956691 | 0.013487334 | 0.003387941 | 0.003938434 | 0.00282948 | 0.007212669 | 0.012031635 | 0.008269118 | 0.00486344 | 0.004979068 | 0.003937717 |
| d__Bacteria;k__norank_d__Bacteria;p__Firmicutes;c__Bacilli;o__Paenibacillales;f__Paenibacillaceae;g__Paenibacillus | 0.011022176 | 0.010314039 | 0.008926163 | 0.013766189 | 0.005477592 | 0.00537159 | 0.004886845 | 0.005108259 | 0.002688103 | 0.005510302 | 0.004632267 | 0.004503925 |
| d__Bacteria;k__norank_d__Bacteria;p__Acidobacteriota;c__Vicinamibacteria;o__Subgroup_17;f__norank_o__Subgroup_17;g__norank_f__norank_o__Subgroup_17 | 0.004303897 | 0.006003695 | 0.003751931 | 0.00458873 | 0.010185604 | 0.00641054 | 0.002796216 | 0.001944769 | 0.007885103 | 0.009918543 | 0.010428794 | 0.013151461 |
| d__Bacteria;k__norank_d__Bacteria;p__Chloroflexi;c__Chloroflexia;o__Chloroflexales;f__Roseiflexaceae;g__norank_f__Roseiflexaceae | 0.005169925 | 0.00643986 | 0.005100665 | 0.004760271 | 0.007990041 | 0.005106327 | 0.004912978 | 0.011953844 | 0.006784261 | 0.007954001 | 0.005945156 | 0.006691545 |
| d__Bacteria;k__norank_d__Bacteria;p__Proteobacteria;c__Alphaproteobacteria;o__Rhizobiales;f__Devosiaceae;g__Devosia | 0.006219656 | 0.003130131 | 0.004830918 | 0.004653058 | 0.009665007 | 0.006366329 | 0.011812052 | 0.007130818 | 0.00962597 | 0.004767609 | 0.005499269 | 0.003165616 |
| d__Bacteria;k__norank_d__Bacteria;p__Proteobacteria;c__Gammaproteobacteria;o__Burkholderiales;f__Oxalobacteraceae;g__Massilia | 0.002834274 | 0.002309113 | 0.001593958 | 0.003366498 | 0.009913988 | 0.020204253 | 0.009564627 | 0.009127447 | 0.006451448 | 0.002084332 | 0.005870841 | 0.001286836 |
| d__Bacteria;k__norank_d__Bacteria;p__Chloroflexi;c__Anaerolineae;o__SBR1031;f__A4b;g__norank_f__A4b | 0.003464112 | 0.006183292 | 0.007013414 | 0.003473711 | 0.010298778 | 0.009018966 | 0.003527936 | 0.003448723 | 0.006349044 | 0.006756109 | 0.00735713 | 0.0053275 |
| d__Bacteria;k__norank_d__Bacteria;p__Methylomirabilota;c__Methylomirabilia;o__Rokubacteriales;f__norank_o__Rokubacteriales;g__norank_f__norank_o__Rokubacteriales | 0.006377116 | 0.004515599 | 0.00343314 | 0.005746634 | 0.007582617 | 0.005592643 | 0.001724769 | 0.002826397 | 0.002739305 | 0.011068519 | 0.011345339 | 0.009188007 |
| d__Bacteria;k__norank_d__Bacteria;p__Proteobacteria;c__Gammaproteobacteria;o__Burkholderiales;f__Nitrosomonadaceae;g__mle1-7 | 0.008292875 | 0.006696429 | 0.00728316 | 0.003109186 | 0.004232684 | 0.003161059 | 0.004050593 | 0.007078958 | 0.004992192 | 0.008025874 | 0.006564443 | 0.006974649 |
| d__Bacteria;k__norank_d__Bacteria;p__Chloroflexi;c__Ktedonobacteria;o__C0119;f__norank_o__C0119;g__norank_f__norank_o__C0119 | 0.003857761 | 0.00749179 | 0.004683783 | 0.006411356 | 0.002376641 | 0.002254742 | 0.010714472 | 0.010916634 | 0.008678733 | 0.005989459 | 0.001758775 | 0.003165616 |
| d__Bacteria;k__norank_d__Bacteria;p__Proteobacteria;c__Alphaproteobacteria;o__Caulobacterales;f__Caulobacteraceae;g__Brevundimonas | 0.002493111 | 0.001103243 | 0.001863704 | 0.008062441 | 0.015187868 | 0.026194792 | 0.003266607 | 0.001737327 | 0.002713704 | 0.001317681 | 0.002576234 | 0.000875048 |
| d__Bacteria;k__norank_d__Bacteria;p__Chloroflexi;c__JG30-KF-CM66;o__norank_c__JG30-KF-CM66;f__norank_o__norank_c__JG30-KF-CM66;g__norank_f__norank_o__norank_c__JG30-KF-CM66 | 0.003254166 | 0.004336002 | 0.003261483 | 0.004395746 | 0.006315075 | 0.004863168 | 0.003135943 | 0.006612213 | 0.005913827 | 0.010589363 | 0.006911244 | 0.006948913 |
| d__Bacteria;k__norank_d__Bacteria;p__Firmicutes;c__Bacilli;o__Bacillales;f__Planococcaceae;g__Paenisporosarcina | 0.00913266 | 0.007260878 | 0.007896221 | 0.004760271 | 0.008850158 | 0.003094743 | 0.003998327 | 0.003967328 | 0.002099281 | 0.006037374 | 0.002774406 | 0.003217089 |
| d__Bacteria;k__norank_d__Bacteria;p__Actinobacteriota;c__Actinobacteria;o__Corynebacteriales;f__Mycobacteriaceae;g__Mycobacterium | 0.005222412 | 0.004336002 | 0.002893646 | 0.005339223 | 0.007175192 | 0.005990539 | 0.005566299 | 0.007727214 | 0.005145798 | 0.004815525 | 0.004087295 | 0.004426715 |
| d__Bacteria;k__norank_d__Bacteria;p__Bacteroidota;c__Bacteroidia;o__Flavobacteriales;f__Flavobacteriaceae;g__Flavobacterium | 0.006849495 | 0.003694581 | 0.005100665 | 0.001844069 | 0.003078316 | 0.003735797 | 0.007656928 | 0.004537793 | 0.007193876 | 0.005869669 | 0.006192871 | 0.005842234 |
| d__Bacteria;k__norank_d__Bacteria;p__Proteobacteria;c__Gammaproteobacteria;o__Pseudomonadales;f__Pseudomonadaceae;g__Pseudomonas | 0.007400604 | 0.005747126 | 0.008754506 | 0.001072133 | 0.003508375 | 0.001945267 | 0.000993049 | 0.000363023 | 0.002688103 | 0.002994729 | 0.016720751 | 0.007746751 |
| d__Bacteria;k__norank_d__Bacteria;p__Actinobacteriota;c__Actinobacteria;o__Micrococcales;f__Microbacteriaceae;g__Leifsonia | 0.002335651 | 0.003053161 | 0.001765615 | 0.001844069 | 0.003598914 | 0.00570317 | 0.007317201 | 0.007597563 | 0.00806431 | 0.004480115 | 0.007456216 | 0.005842234 |
| d__Bacteria;k__norank_d__Bacteria;p__Cyanobacteria;c__Cyanobacteriia;o__Chloroplast;f__norank_o__Chloroplast;g__norank_f__norank_o__Chloroplast | 0.004986222 | 0.008184524 | 0.009392089 | 0.007590702 | 0.003689452 | 0.004973695 | 0.003449537 | 0.003241281 | 0.002790507 | 0.001677048 | 0.005375412 | 0.002959722 |
| d__Bacteria;k__norank_d__Bacteria;p__Chloroflexi;c__Dehalococcoidia;o__S085;f__norank_o__S085;g__norank_f__norank_o__S085 | 0.004540087 | 0.00466954 | 0.004536649 | 0.002959087 | 0.003734722 | 0.003360007 | 0.003162076 | 0.004408142 | 0.004915389 | 0.00862482 | 0.005226783 | 0.005173079 |
| d__Bacteria;k__norank_d__Bacteria;p__unclassified_k__norank_d__Bacteria;c__unclassified_k__norank_d__Bacteria;o__unclassified_k__norank_d__Bacteria;f__unclassified_k__norank_d__Bacteria;g__unclassified_k__norank_d__Bacteria | 0.007059441 | 0.006619458 | 0.007136026 | 0.001586757 | 0.003236759 | 0.001525266 | 0.005409502 | 0.004434072 | 0.002790507 | 0.005007187 | 0.004756125 | 0.004658345 |
| d__Bacteria;k__norank_d__Bacteria;p__Proteobacteria;c__Alphaproteobacteria;o__Rhizobiales;f__Hyphomicrobiaceae;g__Hyphomicrobium | 0.007033198 | 0.006131979 | 0.00566468 | 0.004846042 | 0.004255319 | 0.004001061 | 0.004442586 | 0.005575003 | 0.003532936 | 0.003258265 | 0.003022121 | 0.002342041 |
| d__Bacteria;k__norank_d__Bacteria;p__Proteobacteria;c__Alphaproteobacteria;o__Rhizobiales;f__Rhizobiaceae;g__Ensifer | 0.003726545 | 0.003361043 | 0.004561171 | 0.001951282 | 0.004866455 | 0.003271586 | 0.004625516 | 0.002748606 | 0.004480172 | 0.005031145 | 0.006663529 | 0.006871702 |
| d__Bacteria;k__norank_d__Bacteria;p__Actinobacteriota;c__Actinobacteria;o__Streptomycetales;f__Streptomycetaceae;g__Streptomyces | 0.007977956 | 0.00297619 | 0.003310527 | 0.002766103 | 0.002965143 | 0.002962111 | 0.006533215 | 0.005782445 | 0.004198561 | 0.004360326 | 0.003269836 | 0.003500193 |
| d__Bacteria;k__norank_d__Bacteria;p__Chloroflexi;c__Anaerolineae;o__RBG-13-54-9;f__norank_o__RBG-13-54-9;g__norank_f__norank_o__RBG-13-54-9 | 0.007059441 | 0.006773399 | 0.006498443 | 0.004009778 | 0.003938434 | 0.003094743 | 0.002717817 | 0.004434072 | 0.002969714 | 0.002946814 | 0.002749635 | 0.003268563 |
| d__Bacteria;k__norank_d__Bacteria;p__Nitrospirota;c__Nitrospiria;o__Nitrospirales;f__Nitrospiraceae;g__Nitrospira | 0.007138171 | 0.005464901 | 0.008092401 | 0.003044858 | 0.003259393 | 0.003050533 | 0.001907699 | 0.003007909 | 0.001740867 | 0.004288452 | 0.005251554 | 0.003371509 |
| d__Bacteria;k__norank_d__Bacteria;p__Proteobacteria;c__Alphaproteobacteria;o__Rhizobiales;f__Rhizobiaceae;g__Allorhizobium-Neorhizobium-Pararhizobium-Rhizobium | 0.005694791 | 0.004079433 | 0.006057039 | 0.002508791 | 0.004028972 | 0.002940006 | 0.005252705 | 0.002489304 | 0.003532936 | 0.004216579 | 0.004359781 | 0.003860507 |
| d__Bacteria;k__norank_d__Bacteria;p__Chloroflexi;c__Anaerolineae;o__SBR1031;f__norank_o__SBR1031;g__norank_f__norank_o__SBR1031 | 0.003437869 | 0.005849754 | 0.005419456 | 0.00233725 | 0.004436397 | 0.003227375 | 0.003214342 | 0.004641514 | 0.005581014 | 0.003066603 | 0.003864351 | 0.00375756 |
| d__Bacteria;k__norank_d__Bacteria;p__Gemmatimonadota;c__Gemmatimonadetes;o__Gemmatimonadales;f__Gemmatimonadaceae;g__Gemmatimonas | 0.006875738 | 0.004412972 | 0.004315947 | 0.002508791 | 0.002784065 | 0.002254742 | 0.00475618 | 0.007053027 | 0.005837024 | 0.00287494 | 0.002254205 | 0.002573671 |
| d__Bacteria;k__norank_d__Bacteria;p__Proteobacteria;c__Alphaproteobacteria;o__Rhizobiales;f__Rhizobiales_Incertae_Sedis;g__Bauldia | 0.004382627 | 0.004130747 | 0.004021678 | 0.001308002 | 0.004775917 | 0.002741058 | 0.003057545 | 0.004356282 | 0.003763345 | 0.005390513 | 0.005573584 | 0.004014927 |
| d__Bacteria;k__norank_d__Bacteria;p__Proteobacteria;c__Gammaproteobacteria;o__Burkholderiales;f__Comamonadaceae;g__unclassified_f__Comamonadaceae | 0.005064952 | 0.004823481 | 0.005615635 | 0.001072133 | 0.002829335 | 0.002298952 | 0.004599383 | 0.004615584 | 0.006221039 | 0.002012458 | 0.004483638 | 0.003268563 |
| d__Bacteria;k__norank_d__Bacteria;p__Actinobacteriota;c__Actinobacteria;o__Corynebacteriales;f__Nocardiaceae;g__Rhodococcus | 0.001784543 | 0.001231527 | 0.002574855 | 0.001736856 | 0.005319149 | 0.0049958 | 0.00329274 | 0.004096979 | 0.002764906 | 0.004935314 | 0.00500384 | 0.005198816 |
| d__Bacteria;k__norank_d__Bacteria;p__Proteobacteria;c__Gammaproteobacteria;o__Burkholderiales;f__Nitrosomonadaceae;g__Ellin6067 | 0.003831518 | 0.003566297 | 0.004389514 | 0.000943477 | 0.002105025 | 0.00141474 | 0.003998327 | 0.004797096 | 0.003993753 | 0.004024916 | 0.005994699 | 0.003320036 |
| d__Bacteria;k__norank_d__Bacteria;p__Actinobacteriota;c__Acidimicrobiia;o__Microtrichales;f__Ilumatobacteraceae;g__norank_f__Ilumatobacteraceae | 0.002834274 | 0.002026888 | 0.001937272 | 0.002894759 | 0.003078316 | 0.002210531 | 0.003893796 | 0.005367561 | 0.005043394 | 0.004887398 | 0.003988209 | 0.003809034 |
| d__Bacteria;k__norank_d__Bacteria;p__Firmicutes;c__Clostridia;o__Clostridiales;f__Clostridiaceae;g__Clostridium_sensu_stricto_1 | 0.009342606 | 0.00628592 | 0.006817234 | 0.004031221 | 0.002851969 | 0.002232636 | 0.003580202 | 0.003241281 | 0.00130565 | 0.001126018 | 0.000520201 | 0.000566208 |
| d__Bacteria;k__norank_d__Bacteria;p__Proteobacteria;c__Alphaproteobacteria;o__Rhizobiales;f__Hyphomicrobiaceae;g__Pedomicrobium | 0.002703057 | 0.001821634 | 0.002501287 | 0.00201561 | 0.003666818 | 0.002144215 | 0.002090629 | 0.003241281 | 0.002892911 | 0.005270724 | 0.006366271 | 0.004555398 |
| d__Bacteria;k__norank_d__Bacteria;p__Proteobacteria;c__Gammaproteobacteria;o__Xanthomonadales;f__Xanthomonadaceae;g__Arenimonas | 0.003805275 | 0.004438629 | 0.004634738 | 0.00096492 | 0.002693526 | 0.005813696 | 0.00347567 | 0.002178141 | 0.002380891 | 0.002347868 | 0.003294607 | 0.002599408 |
| d__Bacteria;k__norank_d__Bacteria;p__Myxococcota;c__bacteriap25;o__norank_c__bacteriap25;f__norank_o__norank_c__bacteriap25;g__norank_f__norank_o__norank_c__bacteriap25 | 0.006403359 | 0.004541256 | 0.005100665 | 0.001886954 | 0.002557718 | 0.002475795 | 0.002639419 | 0.001789187 | 0.001331251 | 0.003521802 | 0.003492779 | 0.002650881 |
| d__Bacteria;k__norank_d__Bacteria;p__Acidobacteriota;c__Blastocatellia;o__11-24;f__norank_o__11-24;g__norank_f__norank_o__11-24 | 0.001233434 | 0.003078818 | 0.002305108 | 0.00128656 | 0.002625622 | 0.00216632 | 0.001489573 | 0.000829768 | 0.002176084 | 0.005582175 | 0.004780896 | 0.010603526 |
| d__Bacteria;k__norank_d__Bacteria;p__Actinobacteriota;c__Actinobacteria;o__Frankiales;f__Geodermatophilaceae;g__Blastococcus | 0.000918515 | 0.000538793 | 0.000514971 | 0.012758384 | 0.00174287 | 0.002696848 | 0.003763132 | 0.00632698 | 0.003865748 | 0.00143747 | 0.001461518 | 0.001698623 |
| d__Bacteria;k__norank_d__Bacteria;p__Proteobacteria;c__Gammaproteobacteria;o__Gammaproteobacteria_Incertae_Sedis;f__unclassified_o__Gammaproteobacteria_Incertae_Sedis;g__Acidibacter | 0.003464112 | 0.004643883 | 0.004487604 | 0.001265117 | 0.002105025 | 0.002144215 | 0.002874615 | 0.003682095 | 0.00337933 | 0.002275994 | 0.003567094 | 0.003011195 |
| d__Bacteria;k__norank_d__Bacteria;p__Proteobacteria;c__Alphaproteobacteria;o__Rhizobiales;f__Amb-16S-1323;g__norank_f__Amb-16S-1323 | 0.002781787 | 0.002309113 | 0.0026239 | 0.001393773 | 0.00174287 | 0.000950528 | 0.002822349 | 0.004485933 | 0.003865748 | 0.005055103 | 0.004805668 | 0.003242826 |
| d__Bacteria;k__norank_d__Bacteria;p__Actinobacteriota;c__Acidimicrobiia;o__Microtrichales;f__norank_o__Microtrichales;g__norank_f__norank_o__Microtrichales | 0.002440625 | 0.001872947 | 0.001888227 | 0.002894759 | 0.003372567 | 0.003846324 | 0.002796216 | 0.003993258 | 0.003737744 | 0.003497844 | 0.002724863 | 0.002985459 |
| d__Bacteria;k__norank_d__Bacteria;p__Acidobacteriota;c__Blastocatellia;o__Blastocatellales;f__Blastocatellaceae;g__JGI_0001001-H03 | 0.001574597 | 0.004720854 | 0.003531229 | 0.001779741 | 0.001425985 | 0.001657898 | 0.003527936 | 0.002722676 | 0.005094596 | 0.001677048 | 0.002848721 | 0.00548192 |
| d__Bacteria;k__norank_d__Bacteria;p__Actinobacteriota;c__Acidimicrobiia;o__Microtrichales;f__Ilumatobacteraceae;g__CL500-29_marine_group | 0.001942002 | 0.002463054 | 0.002721989 | 0.000900592 | 0.002557718 | 0.002387373 | 0.003005279 | 0.003941398 | 0.003430532 | 0.004000958 | 0.004409324 | 0.003886244 |
| d__Bacteria;k__norank_d__Bacteria;p__Acidobacteriota;c__Blastocatellia;o__Blastocatellales;f__Blastocatellaceae;g__norank_f__Blastocatellaceae | 0.001023488 | 0.003130131 | 0.00333505 | 0.002551677 | 0.003598914 | 0.004222114 | 0.003397272 | 0.001140931 | 0.00337933 | 0.001653091 | 0.003245064 | 0.004864239 |
| d__Bacteria;k__norank_d__Bacteria;p__Myxococcota;c__Polyangia;o__Haliangiales;f__Haliangiaceae;g__Haliangium | 0.004435114 | 0.003822865 | 0.00313887 | 0.002401578 | 0.002874604 | 0.001613688 | 0.002325824 | 0.004589654 | 0.003200123 | 0.002779109 | 0.002204662 | 0.00164715 |
| d__Bacteria;k__norank_d__Bacteria;p__Proteobacteria;c__Alphaproteobacteria;o__Sphingomonadales;f__Sphingomonadaceae;g__unclassified_f__Sphingomonadaceae | 0.002519354 | 0.002052545 | 0.001912749 | 0.003323613 | 0.003779991 | 0.005526327 | 0.003946062 | 0.001918838 | 0.00363534 | 0.002012458 | 0.002254205 | 0.001904517 |
| d__Bacteria;k__norank_d__Bacteria;p__Proteobacteria;c__Alphaproteobacteria;o__Sphingomonadales;f__Sphingomonadaceae;g__Qipengyuania | 0.00167957 | 0.00056445 | 0.000735673 | 0.007204735 | 0.010321412 | 0.007957911 | 0.001594104 | 0.000933489 | 0.000947236 | 0.001054145 | 0.000767916 | 0.001003732 |
| d__Bacteria;k__norank_d__Bacteria;p__Actinobacteriota;c__Actinobacteria;o__Propionibacteriales;f__Nocardioidaceae;g__Marmoricola | 0.001180947 | 0.000307882 | 0.000269747 | 0.017668754 | 0.003757356 | 0.004730536 | 0.002874615 | 0.001789187 | 0.000972837 | 0.000455199 | 0.000421115 | 0.000257367 |
| d__Bacteria;k__norank_d__Bacteria;p__Proteobacteria;c__Alphaproteobacteria;o__norank_c__Alphaproteobacteria;f__norank_o__norank_c__Alphaproteobacteria;g__norank_f__norank_o__norank_c__Alphaproteobacteria | 0.004802519 | 0.004207718 | 0.003163393 | 0.001265117 | 0.00212766 | 0.001149476 | 0.00219516 | 0.00318942 | 0.002278487 | 0.003521802 | 0.003765265 | 0.003011195 |
| d__Bacteria;k__norank_d__Bacteria;p__Patescibacteria;c__Saccharimonadia;o__Saccharimonadales;f__LWQ8;g__norank_f__LWQ8 | 0.001915759 | 0.001488095 | 0.00161848 | 0.001736856 | 0.003395201 | 0.002321058 | 0.005461768 | 0.007053027 | 0.006067433 | 0.00088644 | 0.00116426 | 0.001492729 |
| d__Bacteria;k__norank_d__Bacteria;p__Bacteroidota;c__Bacteroidia;o__Sphingobacteriales;f__Sphingobacteriaceae;g__Pedobacter | 0.003175436 | 0.0011289 | 0.002550332 | 0.004760271 | 0.007265731 | 0.006366329 | 0.002038363 | 0.00103721 | 0.000947236 | 0.001245807 | 0.002526691 | 0.001209626 |
| d__Bacteria;k__norank_d__Bacteria;p__Proteobacteria;c__Alphaproteobacteria;o__Micropepsales;f__Micropepsaceae;g__norank_f__Micropepsaceae | 0.006429602 | 0.005362274 | 0.006547488 | 4.29E-05 | 0.000113173 | 0.000243158 | 0.003057545 | 0.00526384 | 0.003968152 | 0.001006229 | 0.000916545 | 0.001080942 |
| d__Bacteria;k__norank_d__Bacteria;p__Bacteroidota;c__Bacteroidia;o__Cytophagales;f__Hymenobacteraceae;g__Adhaeribacter | 0.00167957 | 0.001334154 | 0.002256063 | 0.003773909 | 0.006835672 | 0.011428445 | 0.002221293 | 0.000881628 | 0.000947236 | 0.000503115 | 0.001238574 | 0.000694891 |
| d__Bacteria;k__norank_d__Bacteria;p__Proteobacteria;c__Alphaproteobacteria;o__Rhizobiales;f__Rhizobiaceae;g__Mesorhizobium | 0.002703057 | 0.002770936 | 0.002697467 | 0.001500986 | 0.002851969 | 0.001547372 | 0.003188209 | 0.003396862 | 0.003302527 | 0.003114518 | 0.003071664 | 0.002496461 |
| d__Bacteria;k__norank_d__Bacteria;p__Proteobacteria;c__Gammaproteobacteria;o__Burkholderiales;f__A21b;g__norank_f__A21b | 0.005327385 | 0.003694581 | 0.003604797 | 0.001029248 | 0.001290177 | 0.001171581 | 0.00237809 | 0.003604304 | 0.002406492 | 0.003641591 | 0.00183309 | 0.002573671 |
| d__Bacteria;k__norank_d__Bacteria;p__Acidobacteriota;c__Thermoanaerobaculia;o__Thermoanaerobaculales;f__Thermoanaerobaculaceae;g__Subgroup_10 | 0.001968246 | 0.003027504 | 0.004757351 | 0.002122824 | 0.004096876 | 0.002851585 | 0.001202112 | 0.001503954 | 0.004275364 | 0.002012458 | 0.00267532 | 0.00195599 |
| d__Bacteria;k__norank_d__Bacteria;p__Proteobacteria;c__Alphaproteobacteria;o__Rhizobiales;f__Rhizobiales_Incertae_Sedis;g__norank_f__Rhizobiales_Incertae_Sedis | 0.003332896 | 0.003771552 | 0.004879963 | 0.001629642 | 0.004028972 | 0.002276847 | 0.001254377 | 0.00106314 | 0.000972837 | 0.003378055 | 0.003294607 | 0.002367778 |
| d__Bacteria;k__norank_d__Bacteria;p__Chloroflexi;c__TK10;o__norank_c__TK10;f__norank_o__norank_c__TK10;g__norank_f__norank_o__norank_c__TK10 | 0.00288676 | 0.003617611 | 0.003212438 | 0.002187152 | 0.001810774 | 0.00141474 | 0.002770083 | 0.004382212 | 0.002585699 | 0.003665549 | 0.00133766 | 0.002161884 |
| d__Bacteria;k__norank_d__Bacteria;p__Actinobacteriota;c__Acidimicrobiia;o__Microtrichales;f__Ilumatobacteraceae;g__Ilumatobacter | 0.000839785 | 0.000923645 | 0.001226121 | 0.001972725 | 0.003508375 | 0.003625271 | 0.002430356 | 0.002644885 | 0.004044955 | 0.003330139 | 0.003542322 | 0.002110411 |
| d__Bacteria;k__norank_d__Bacteria;p__Desulfobacterota;c__norank_p__Desulfobacterota;o__norank_c__norank_p__Desulfobacterota;f__norank_o__norank_c__norank_p__Desulfobacterota;g__norank_f__norank_o__norank_c__norank_p__Desulfobacterota | 0.005038709 | 0.002206486 | 0.003212438 | 0.001200789 | 0.001425985 | 0.00103895 | 0.001515706 | 0.002411513 | 0.001024039 | 0.004480115 | 0.003542322 | 0.002908249 |
| d__Bacteria;k__norank_d__Bacteria;p__Proteobacteria;c__Gammaproteobacteria;o__Burkholderiales;f__Burkholderiaceae;g__Burkholderia-Caballeronia-Paraburkholderia | 0.00312295 | 0.004823481 | 0.002893646 | 0.000192984 | 0.000226347 | 0.000132632 | 0.004834579 | 0.003759886 | 0.002636901 | 0.001820795 | 0.002576234 | 0.002856775 |
| d__Bacteria;k__norank_d__Bacteria;p__Proteobacteria;c__Alphaproteobacteria;o__Rhizobiales;f__Xanthobacteraceae;g__unclassified_f__Xanthobacteraceae | 0.003385382 | 0.001590722 | 0.002207018 | 0.000793379 | 0.001946582 | 0.00103895 | 0.003710866 | 0.00313756 | 0.003532936 | 0.002779109 | 0.002130348 | 0.002393514 |
| d__Bacteria;k__norank_d__Bacteria;p__Actinobacteriota;c__Thermoleophilia;o__Solirubrobacterales;f__Solirubrobacteraceae;g__Conexibacter | 0.00280803 | 0.001616379 | 0.001078987 | 0.00362381 | 0.001720235 | 0.002475795 | 0.003005279 | 0.003578374 | 0.003302527 | 0.002012458 | 0.001907404 | 0.001235362 |
| d__Bacteria;k__norank_d__Bacteria;p__Proteobacteria;c__Alphaproteobacteria;o__Elsterales;f__norank_o__Elsterales;g__norank_f__norank_o__Elsterales | 0.003752788 | 0.00233477 | 0.003089826 | 0.000771936 | 0.000792214 | 0.000530527 | 0.002482622 | 0.004874887 | 0.002816108 | 0.00232391 | 0.001709232 | 0.001672886 |
| d__Bacteria;k__norank_d__Bacteria;p__Bacteroidota;c__Bacteroidia;o__Chitinophagales;f__Chitinophagaceae;g__Terrimonas | 0.001942002 | 0.002411741 | 0.003580274 | 0.000814821 | 0.002987777 | 0.001989478 | 0.001385042 | 0.000648256 | 0.001510458 | 0.00232391 | 0.003542322 | 0.003783297 |
| d__Bacteria;k__norank_d__Bacteria;p__Bacteroidota;c__Bacteroidia;o__Chitinophagales;f__Saprospiraceae;g__norank_f__Saprospiraceae | 0.002571841 | 0.003745895 | 0.004364992 | 0.000729051 | 0.002331372 | 0.001326319 | 0.000627189 | 0.000570465 | 0.000870433 | 0.001916627 | 0.004657039 | 0.003036932 |
| d__Bacteria;k__norank_d__Bacteria;p__Latescibacterota;c__norank_p__Latescibacterota;o__norank_c__norank_p__Latescibacterota;f__norank_o__norank_c__norank_p__Latescibacterota;g__norank_f__norank_o__norank_c__norank_p__Latescibacterota | 0.002388138 | 0.003976806 | 0.00242772 | 0.000771936 | 0.001833409 | 0.000862107 | 0.001437307 | 0.001374303 | 0.003558537 | 0.002419741 | 0.002229433 | 0.003242826 |
| d__Bacteria;k__norank_d__Bacteria;p__Acidobacteriota;c__Acidobacteriae;o__Subgroup_2;f__norank_o__Subgroup_2;g__norank_f__norank_o__Subgroup_2 | 0.00320168 | 0.00636289 | 0.007356728 | 0.000214427 | 0.000407424 | 0.000176842 | 0.001907699 | 0.002178141 | 0.002969714 | 0.000718735 | 0.000396344 | 0.000437524 |
| d__Bacteria;k__norank_d__Bacteria;p__Bacteroidota;c__Bacteroidia;o__Chitinophagales;f__Chitinophagaceae;g__norank_f__Chitinophagaceae | 0.002335651 | 0.00282225 | 0.003114348 | 0.000493181 | 0.001788139 | 0.001481056 | 0.002953013 | 0.002515234 | 0.002816108 | 0.001557259 | 0.002254205 | 0.002084674 |
| d__Bacteria;k__norank_d__Bacteria;p__Actinobacteriota;c__Actinobacteria;o__Micromonosporales;f__Micromonosporaceae;g__Micromonospora | 0.002151949 | 0.000846675 | 0.000735673 | 0.002144266 | 0.001222273 | 0.002144215 | 0.00347567 | 0.003552444 | 0.003584138 | 0.002419741 | 0.002105576 | 0.001775833 |
| d__Bacteria;k__norank_d__Bacteria;p__Actinobacteriota;c__Actinobacteria;o__Micrococcales;f__Microbacteriaceae;g__Microbacterium | 0.000183703 | 0.000384852 | 0.000196179 | 0.001308002 | 0.003689452 | 0.004752642 | 0.002848482 | 0.00207442 | 0.002329689 | 0.001677048 | 0.002328519 | 0.003191352 |
| d__Bacteria;k__norank_d__Bacteria;p__Chloroflexi;c__Anaerolineae;o__Caldilineales;f__Caldilineaceae;g__norank_f__Caldilineaceae | 0.001627083 | 0.002386084 | 0.002133451 | 0.002487349 | 0.002738796 | 0.003315796 | 0.001698636 | 0.00101128 | 0.001024039 | 0.0019885 | 0.002080805 | 0.002264831 |
| d__Bacteria;k__norank_d__Bacteria;p__Firmicutes;c__Bacilli;o__Bacillales;f__Planococcaceae;g__Sporosarcina | 0.005589818 | 0.00410509 | 0.004095245 | 0.002465906 | 0.001222273 | 0.000420001 | 0.001202112 | 0.002255932 | 0.000665626 | 0.000910398 | 0.000718373 | 0.000952258 |
| d__Bacteria;k__norank_d__Bacteria;p__Actinobacteriota;c__Actinobacteria;o__Frankiales;f__Nakamurellaceae;g__Nakamurella | 0.000971001 | 0.000821018 | 0.000514971 | 0.000771936 | 0.001946582 | 0.001812635 | 0.004703915 | 0.004252561 | 0.00312332 | 0.00177288 | 0.002278976 | 0.00156994 |
| d__Bacteria;k__norank_d__Bacteria;p__Patescibacteria;c__Saccharimonadia;o__Saccharimonadales;f__unclassified_o__Saccharimonadales;g__unclassified_o__Saccharimonadales | 0.001417137 | 0.001642036 | 0.001177076 | 0.001436658 | 0.002218198 | 0.002409479 | 0.002848482 | 0.004071049 | 0.003891349 | 0.001173934 | 0.001089945 | 0.001106679 |
| d__Bacteria;k__norank_d__Bacteria;p__Proteobacteria;c__Alphaproteobacteria;o__Caulobacterales;f__Caulobacteraceae;g__Phenylobacterium | 0.000997244 | 0.000436166 | 0.000956375 | 0.000707608 | 0.002784065 | 0.0024979 | 0.002953013 | 0.003682095 | 0.002534497 | 0.001964542 | 0.001758775 | 0.002367778 |
| d__Bacteria;k__norank_d__Bacteria;p__Proteobacteria;c__Alphaproteobacteria;o__Rhizobiales;f__Beijerinckiaceae;g__Bosea | 0.00144338 | 0.001488095 | 0.001667525 | 0.000707608 | 0.003259393 | 0.002674742 | 0.001881566 | 0.001581745 | 0.001587261 | 0.001916627 | 0.003121207 | 0.002290567 |
| d__Bacteria;k__norank_d__Bacteria;p__MBNT15;c__norank_p__MBNT15;o__norank_c__norank_p__MBNT15;f__norank_o__norank_c__norank_p__MBNT15;g__norank_f__norank_o__norank_c__norank_p__MBNT15 | 0.005196168 | 0.002693966 | 0.003776454 | 0.000922034 | 0.000837483 | 0.000530527 | 0.000418126 | 0.000959419 | 0.001177645 | 0.002563488 | 0.001956947 | 0.002547935 |
| d__Bacteria;k__norank_d__Bacteria;p__Proteobacteria;c__Alphaproteobacteria;o__Reyranellales;f__Reyranellaceae;g__Reyranella | 0.002493111 | 0.002309113 | 0.00252581 | 0.000428853 | 0.00174287 | 0.000641054 | 0.002351957 | 0.003059769 | 0.002508896 | 0.001892669 | 0.002056033 | 0.00156994 |
| d__Bacteria;k__norank_d__Bacteria;p__Chloroflexi;c__Anaerolineae;o__Anaerolineales;f__Anaerolineaceae;g__norank_f__Anaerolineaceae | 0.002388138 | 0.003899836 | 0.003261483 | 0.001136461 | 0.001312811 | 0.001967373 | 0.000601056 | 0.00108907 | 0.001792069 | 0.001724964 | 0.002378062 | 0.001930254 |
| d__Bacteria;k__norank_d__Bacteria;p__Actinobacteriota;c__Actinobacteria;o__Propionibacteriales;f__Nocardioidaceae;g__unclassified_f__Nocardioidaceae | 0.000524866 | 0.000410509 | 0.000147135 | 0.013401664 | 0.001901313 | 0.002895796 | 0.001881566 | 0.00108907 | 0.000486419 | 0.000215621 | 0.0001734 | 0.000205894 |
| d__Bacteria;k__norank_d__Bacteria;p__Firmicutes;c__Bacilli;o__Bacillales;f__Bacillaceae;g__Fictibacillus | 0.001338407 | 0.001077586 | 0.00152039 | 0.001651085 | 0.001674966 | 0.006587382 | 0.004416453 | 0.000674186 | 0.000665626 | 0.001126018 | 0.001015631 | 0.001364046 |
| d__Bacteria;k__norank_d__Bacteria;p__Actinobacteriota;c__Thermoleophilia;o__Solirubrobacterales;f__Solirubrobacteraceae;g__Solirubrobacter | 0.000813542 | 0.00064142 | 0.00071115 | 0.001844069 | 0.002308737 | 0.001945267 | 0.002979146 | 0.003345002 | 0.003737744 | 0.001820795 | 0.001312889 | 0.001415519 |
| d__Bacteria;k__norank_d__Bacteria;p__Verrucomicrobiota;c__Verrucomicrobiae;o__Chthoniobacterales;f__Chthoniobacteraceae;g__Candidatus_Udaeobacter | 0.002650571 | 0.005516215 | 0.006449398 | 0.002208594 | 0.000701675 | 0.000353685 | 0.000810119 | 0.000388954 | 0.000307212 | 0.001126018 | 0.000916545 | 0.001132415 |
| d__Bacteria;k__norank_d__Bacteria;p__Actinobacteriota;c__Actinobacteria;o__Micrococcales;f__Intrasporangiaceae;g__Oryzihumus | 0.000656082 | 0.000949302 | 0.000735673 | 0.004438631 | 0.001561793 | 0.001392635 | 0.003527936 | 0.003396862 | 0.002816108 | 0.001054145 | 0.000767916 | 0.001003732 |
| d__Bacteria;k__norank_d__Bacteria;p__Planctomycetota;c__Planctomycetes;o__Gemmatales;f__Gemmataceae;g__norank_f__Gemmataceae | 0.001469623 | 0.003591954 | 0.002157973 | 0.001265117 | 0.001516523 | 0.000994739 | 0.002090629 | 0.000985349 | 0.001510458 | 0.001964542 | 0.001659689 | 0.002882512 |
| d__Bacteria;k__norank_d__Bacteria;p__Chloroflexi;c__Chloroflexia;o__Thermomicrobiales;f__AKYG1722;g__norank_f__AKYG1722 | 0.000524866 | 0.000692734 | 0.00080924 | 0.002508791 | 0.003282028 | 0.003691587 | 0.001437307 | 0.002022559 | 0.001689665 | 0.001533301 | 0.002353291 | 0.001441256 |
| d__Bacteria;k__norank_d__Bacteria;p__Proteobacteria;c__Gammaproteobacteria;o__Xanthomonadales;f__Rhodanobacteraceae;g__Dokdonella | 0.001968246 | 0.001257184 | 0.001422301 | 0.000192984 | 0.000475328 | 0.000132632 | 0.002979146 | 0.005575003 | 0.004403369 | 0.001126018 | 0.00116426 | 0.001183889 |
| d__Bacteria;k__norank_d__Bacteria;p__Proteobacteria;c__Alphaproteobacteria;o__Rhizobiales;f__Xanthobacteraceae;g__Rhodoplanes | 0.00136465 | 0.001000616 | 0.00071115 | 0.000428853 | 0.001448619 | 0.001149476 | 0.002064496 | 0.002359653 | 0.002048079 | 0.002994729 | 0.003542322 | 0.002573671 |
| d__Bacteria;k__norank_d__Bacteria;p__Proteobacteria;c__Gammaproteobacteria;o__Xanthomonadales;f__Xanthomonadaceae;g__Thermomonas | 0.000813542 | 0.000282225 | 0.000416881 | 0.003602367 | 0.006201901 | 0.00853265 | 0.000679454 | 0.000440814 | 0.000358414 | 0.000167705 | 4.95E-05 | 0.000128684 |
| d__Bacteria;k__norank_d__Bacteria;p__Chloroflexi;c__Ktedonobacteria;o__Ktedonobacterales;f__JG30-KF-AS9;g__norank_f__JG30-KF-AS9 | 0.003516599 | 0.001411125 | 0.001961794 | 0.000729051 | 0.000226347 | 0.000132632 | 0.008362515 | 0.00204849 | 0.000998438 | 0.00110206 | 0.000619287 | 0.000540471 |
| d__Bacteria;k__norank_d__Bacteria;p__Chloroflexi;c__AD3;o__norank_c__AD3;f__norank_o__norank_c__AD3;g__norank_f__norank_o__norank_c__AD3 | 0.002493111 | 0.002180829 | 0.001348733 | 0.000514624 | 0.000226347 | 0.00037579 | 0.001385042 | 0.007182679 | 0.002995315 | 0.001173934 | 0.000594516 | 0.000977995 |
| d__Bacteria;k__norank_d__Bacteria;p__Proteobacteria;c__Alphaproteobacteria;o__Rhizobiales;f__Beijerinckiaceae;g__Microvirga | 0.00128592 | 0.001000616 | 0.001054464 | 0.003087743 | 0.00246718 | 0.003824219 | 0.000810119 | 0.00106314 | 0.000947236 | 0.002467657 | 0.001709232 | 0.001492729 |
| d__Bacteria;k__norank_d__Bacteria;p__Proteobacteria;c__Alphaproteobacteria;o__Rhizobiales;f__Rhizobiaceae;g__Aminobacter | 0.002545598 | 0.001359811 | 0.001667525 | 0.000986362 | 0.003214124 | 0.00179053 | 0.00201223 | 0.00106314 | 0.001587261 | 0.001078103 | 0.002700092 | 0.001029469 |
| d__Bacteria;k__norank_d__Bacteria;p__Bacteroidota;c__Bacteroidia;o__Chitinophagales;f__Chitinophagaceae;g__Ferruginibacter | 0.00144338 | 0.001334154 | 0.002403198 | 0.000729051 | 0.001923947 | 0.001392635 | 0.002979146 | 0.001892908 | 0.002304088 | 0.000862482 | 0.001436746 | 0.001518466 |
| d__Bacteria;k__norank_d__Bacteria;p__Bacteroidota;c__Bacteroidia;o__Cytophagales;f__Microscillaceae;g__Chryseolinea | 0.001810786 | 0.001949918 | 0.003310527 | 0.001093576 | 0.001425985 | 0.001304213 | 0.00146344 | 0.001192791 | 0.001638463 | 0.000910398 | 0.002724863 | 0.001235362 |
| d__Bacteria;k__norank_d__Bacteria;p__Proteobacteria;c__Alphaproteobacteria;o__Azospirillales;f__norank_o__Azospirillales;g__norank_f__norank_o__Azospirillales | 0.002099462 | 0.001847291 | 0.001593958 | 0.003795351 | 0.001448619 | 0.000972634 | 0.00091465 | 0.001166861 | 0.000998438 | 0.002204121 | 0.001734004 | 0.001261099 |
| d__Bacteria;k__norank_d__Bacteria;p__Methylomirabilota;c__Methylomirabilia;o__Rokubacteriales;f__WX65;g__norank_f__WX65 | 0.001259677 | 0.000846675 | 0.00080924 | 0.000514624 | 0.001629697 | 0.001436845 | 0.000391993 | 0.000466744 | 0.000409616 | 0.004408241 | 0.004310238 | 0.00344872 |
| d__Bacteria;k__norank_d__Bacteria;p__Patescibacteria;c__Saccharimonadia;o__Saccharimonadales;f__WWH38;g__norank_f__WWH38 | 0.001417137 | 0.001359811 | 0.001299689 | 0.000900592 | 0.00033952 | 0.00070737 | 0.002796216 | 0.005808375 | 0.003712143 | 0.000670819 | 0.000470658 | 0.000360314 |
| d__Bacteria;k__norank_d__Bacteria;p__Actinobacteriota;c__Thermoleophilia;o__Solirubrobacterales;f__Solirubrobacteraceae;g__norank_f__Solirubrobacteraceae | 0.001233434 | 0.000692734 | 0.000662105 | 0.000686165 | 0.004979629 | 0.003492639 | 0.001777034 | 0.001996629 | 0.00130565 | 0.000790609 | 0.000990859 | 0.000797838 |
| d__Bacteria;k__norank_d__Bacteria;p__Bdellovibrionota;c__Oligoflexia;o__0319-6G20;f__norank_o__0319-6G20;g__norank_f__norank_o__0319-6G20 | 0.002256922 | 0.001744663 | 0.002207018 | 0.001715413 | 0.001290177 | 0.001503161 | 0.00164637 | 0.001244652 | 0.000972837 | 0.001892669 | 0.00133766 | 0.001338309 |
| d__Bacteria;k__norank_d__Bacteria;p__Acidobacteriota;c__Subgroup_5;o__norank_c__Subgroup_5;f__norank_o__norank_c__Subgroup_5;g__norank_f__norank_o__norank_c__Subgroup_5 | 0.000892271 | 0.003104475 | 0.002721989 | 0.001436658 | 0.001358081 | 0.000729475 | 0.001515706 | 0.000440814 | 0.001177645 | 0.001629133 | 0.000941316 | 0.002187621 |
| d__Bacteria;k__norank_d__Bacteria;p__Acidobacteriota;c__Acidobacteriae;o__Acidobacteriales;f__Koribacteraceae;g__Candidatus_Koribacter | 0.001942002 | 0.004695197 | 0.003457662 | 0.000922034 | 0.000520598 | 0.000950528 | 0.001411174 | 0.001374303 | 0.002176084 | 9.58E-05 | 0.000198172 | 0.00015442 |
| d__Bacteria;k__norank_d__Bacteria;p__Firmicutes;c__Bacilli;o__Alicyclobacillales;f__Alicyclobacillaceae;g__Tumebacillus | 0.001994489 | 0.00177032 | 0.00232963 | 0.001736856 | 0.00101856 | 0.000596843 | 0.000810119 | 0.00106314 | 0.000588823 | 0.002299952 | 0.00133766 | 0.002342041 |
| d__Bacteria;k__norank_d__Bacteria;p__Proteobacteria;c__Alphaproteobacteria;o__Rhizobiales;f__Rhizobiaceae;g__unclassified_f__Rhizobiaceae | 0.00167957 | 0.001385468 | 0.00161848 | 0.001308002 | 0.001946582 | 0.001503161 | 0.001620237 | 0.001711396 | 0.001587261 | 0.00110206 | 0.001040402 | 0.001235362 |
| d__Bacteria;k__norank_d__Bacteria;p__Proteobacteria;c__Gammaproteobacteria;o__PLTA13;f__norank_o__PLTA13;g__norank_f__norank_o__PLTA13 | 0.002230678 | 0.000795361 | 0.001741092 | 0.001651085 | 0.001380715 | 0.00103895 | 0.000574923 | 0.000388954 | 0.00051202 | 0.002108289 | 0.003443236 | 0.001621413 |
| d__Bacteria;k__norank_d__Bacteria;p__Actinobacteriota;c__Actinobacteria;o__Micrococcales;f__Cellulomonadaceae;g__Cellulomonas | 0.000472379 | 0.00056445 | 0.000367836 | 0.000364525 | 0.000769579 | 0.000972634 | 0.003083677 | 0.001944769 | 0.004556975 | 0.001245807 | 0.001461518 | 0.00164715 |
| d__Bacteria;k__norank_d__Bacteria;p__Proteobacteria;c__Gammaproteobacteria;o__Burkholderiales;f__TRA3-20;g__norank_f__TRA3-20 | 0.002283165 | 0.002565681 | 0.003065303 | 0.000600395 | 0.001109099 | 0.000618949 | 0.001149846 | 0.000985349 | 0.001612862 | 0.001197892 | 0.001263346 | 0.000900785 |
| d__Bacteria;k__norank_d__Bacteria;p__Proteobacteria;c__Alphaproteobacteria;o__Sphingomonadales;f__Sphingomonadaceae;g__Altererythrobacter | 0.000682325 | 0.000384852 | 0.000220702 | 0.000793379 | 0.001380715 | 0.00145895 | 0.003998327 | 0.001815117 | 0.004633778 | 0.000646862 | 0.00049543 | 0.000669155 |
| d__Bacteria;k__norank_d__Bacteria;p__Proteobacteria;c__Alphaproteobacteria;o__Dongiales;f__Dongiaceae;g__Dongia | 0.00160084 | 0.0011289 | 0.002157973 | 0.000514624 | 0.001674966 | 0.001061055 | 0.000862384 | 0.00101128 | 0.00156166 | 0.002012458 | 0.001932176 | 0.00156994 |
| d__Bacteria;k__norank_d__Bacteria;p__Proteobacteria;c__Alphaproteobacteria;o__Caulobacterales;f__Hyphomonadaceae;g__SWB02 | 0.001784543 | 0.001924261 | 0.002844601 | 0.000364525 | 0.001154368 | 0.000817896 | 0.000601056 | 0.001140931 | 0.001536059 | 0.001724964 | 0.00183309 | 0.001158152 |
| d__Bacteria;k__norank_d__Bacteria;p__Proteobacteria;c__Gammaproteobacteria;o__Burkholderiales;f__Oxalobacteraceae;g__unclassified_f__Oxalobacteraceae | 0.002440625 | 0.001436782 | 0.002771034 | 0.001136461 | 0.001086464 | 0.001503161 | 0.000522657 | 0.000777907 | 0.00051202 | 0.001653091 | 0.001560603 | 0.001286836 |
| d__Bacteria;k__norank_d__Bacteria;p__Firmicutes;c__Bacilli;o__Bacillales;f__Planococcaceae;g__Lysinibacillus | 0.003149193 | 0.00282225 | 0.002231541 | 0.001308002 | 0.001674966 | 0.000729475 | 0.001045314 | 0.000829768 | 0.000332813 | 0.001054145 | 0.000941316 | 0.000411787 |
| d__Bacteria;k__norank_d__Bacteria;p__Proteobacteria;c__Gammaproteobacteria;o__Xanthomonadales;f__Xanthomonadaceae;g__Luteimonas | 0.005301142 | 0.000538793 | 0.000564016 | 0.003194957 | 0.001154368 | 0.001680004 | 0.00201223 | 0.000466744 | 0.000384015 | 0.000455199 | 0.000148629 | 0.000128684 |
| d__Bacteria;k__norank_d__Bacteria;p__Proteobacteria;c__Alphaproteobacteria;o__Rhizobiales;f__Labraceae;g__Labrys | 0.001653326 | 0.000974959 | 0.001397778 | 0.000235869 | 0.000950656 | 0.000353685 | 0.002587153 | 0.001970699 | 0.001920074 | 0.001389554 | 0.001362432 | 0.001106679 |
| d__Bacteria;k__norank_d__Bacteria;p__Acidobacteriota;c__Subgroup_11;o__norank_c__Subgroup_11;f__norank_o__norank_c__Subgroup_11;g__norank_f__norank_o__norank_c__Subgroup_11 | 0.001417137 | 0.002719622 | 0.002035361 | 0.000407411 | 0.000565867 | 0.000397896 | 0.000653321 | 0.000363023 | 0.002534497 | 0.001844753 | 0.001213803 | 0.00172436 |
| d__Bacteria;k__norank_d__Bacteria;p__Bdellovibrionota;c__Bdellovibrionia;o__Bdellovibrionales;f__Bdellovibrionaceae;g__Bdellovibrio | 0.001312164 | 0.001077586 | 0.001054464 | 0.000364525 | 0.000882752 | 0.000420001 | 0.000862384 | 0.000570465 | 0.00130565 | 0.001485386 | 0.003864351 | 0.002625145 |
| d__Bacteria;k__norank_d__Bacteria;p__Actinobacteriota;c__Actinobacteria;o__Pseudonocardiales;f__Pseudonocardiaceae;g__Pseudonocardia | 0.000734812 | 0.000513136 | 0.00071115 | 0.001157904 | 0.001516523 | 0.001348424 | 0.001933832 | 0.002437443 | 0.001868872 | 0.001054145 | 0.001189031 | 0.001286836 |
| d__Bacteria;k__norank_d__Bacteria;p__Acidobacteriota;c__Vicinamibacteria;o__Vicinamibacterales;f__Vicinamibacteraceae;g__Luteitalea | 0.000209946 | 0.000307882 | 0.000465926 | 0.00169397 | 0.003078316 | 0.001525266 | 0.001202112 | 0.000803838 | 0.001331251 | 0.000862482 | 0.001139488 | 0.002573671 |
| d__Bacteria;k__norank_d__Bacteria;p__Acidobacteriota;c__Subgroup_25;o__norank_c__Subgroup_25;f__norank_o__norank_c__Subgroup_25;g__norank_f__norank_o__norank_c__Subgroup_25 | 0.000341163 | 0.001590722 | 0.000956375 | 0.001651085 | 0.001516523 | 0.000906318 | 0.001071447 | 7.78E-05 | 0.000281611 | 0.001629133 | 0.001758775 | 0.003294299 |
| d__Bacteria;k__norank_d__Bacteria;p__Proteobacteria;c__Gammaproteobacteria;o__Burkholderiales;f__Nitrosomonadaceae;g__MND1 | 0.00280803 | 0.001949918 | 0.002599377 | 0.000986362 | 0.001222273 | 0.000795791 | 0.000339727 | 0.001192791 | 0.000281611 | 0.000910398 | 0.000867002 | 0.000849312 |
| d__Bacteria;k__norank_d__Bacteria;p__Myxococcota;c__Polyangia;o__Polyangiales;f__BIrii41;g__norank_f__BIrii41 | 0.001128461 | 0.00056445 | 0.001961794 | 0.000536067 | 0.000543232 | 0.000862107 | 0.001385042 | 0.001478024 | 0.003481734 | 0.000431241 | 0.001535832 | 0.000643418 |
| d__Bacteria;k__norank_d__Bacteria;p__Firmicutes;c__Limnochordia;o__norank_c__Limnochordia;f__norank_o__norank_c__Limnochordia;g__Hydrogenispora | 0.001233434 | 0.00177032 | 0.00161848 | 0.002187152 | 0.000769579 | 0.000972634 | 0.001567972 | 0.000777907 | 0.000819231 | 0.000958313 | 0.000644059 | 0.001209626 |
| d__Bacteria;k__norank_d__Bacteria;p__Bdellovibrionota;c__Oligoflexia;o__Oligoflexales;f__norank_o__Oligoflexales;g__Oligoflexus | 0.000577352 | 0.001000616 | 0.001324211 | 0.000300197 | 0.001041195 | 0.00141474 | 0.000705587 | 0.000363023 | 0.000460818 | 0.002228079 | 0.002179891 | 0.002882512 |
| d__Bacteria;k__norank_d__Bacteria;p__NB1-j;c__norank_p__NB1-j;o__norank_c__norank_p__NB1-j;f__norank_o__norank_c__norank_p__NB1-j;g__norank_f__norank_o__norank_c__norank_p__NB1-j | 0.002099462 | 0.001642036 | 0.00152039 | 0.000793379 | 0.001177003 | 0.000596843 | 0.00036586 | 0.000129651 | 0.000460818 | 0.002060374 | 0.001783547 | 0.001415519 |
| d__Bacteria;k__norank_d__Bacteria;p__Patescibacteria;c__Saccharimonadia;o__Saccharimonadales;f__Saccharimonadaceae;g__TM7a | 0.000603595 | 0.000872332 | 0.000686628 | 0.001029248 | 0.002172929 | 0.003337902 | 0.001254377 | 0.000881628 | 0.001536059 | 0.000383325 | 0.000520201 | 0.000463261 |
| d__Bacteria;k__norank_d__Bacteria;p__Myxococcota;c__Polyangia;o__Polyangiales;f__Sandaracinaceae;g__norank_f__Sandaracinaceae | 0.001627083 | 0.001488095 | 0.001961794 | 0.000557509 | 0.00067904 | 0.000840002 | 0.000810119 | 0.001244652 | 0.001075241 | 0.000838524 | 0.001288117 | 0.001055205 |
| d__Bacteria;k__norank_d__Bacteria;p__Proteobacteria;c__Alphaproteobacteria;o__Rhodobacterales;f__Rhodobacteraceae;g__Amaricoccus | 0.000524866 | 0.000769704 | 0.000564016 | 0.000986362 | 0.001788139 | 0.001635793 | 0.001019182 | 0.001348373 | 0.001024039 | 0.000814566 | 0.001461518 | 0.001209626 |
| d__Bacteria;k__norank_d__Bacteria;p__Actinobacteriota;c__Acidimicrobiia;o__Microtrichales;f__Iamiaceae;g__Iamia | 0.000341163 | 0.000256568 | 0.000196179 | 0.00105069 | 0.001878678 | 0.002100004 | 0.001489573 | 0.001296512 | 0.001766468 | 0.000646862 | 0.001387203 | 0.000617681 |
| d__Bacteria;k__norank_d__Bacteria;p__Chloroflexi;c__OLB14;o__norank_c__OLB14;f__norank_o__norank_c__OLB14;g__norank_f__norank_o__norank_c__OLB14 | 0.000944758 | 0.000974959 | 0.001397778 | 0.001007805 | 0.001335446 | 0.000994739 | 0.000627189 | 0.00106314 | 0.001177645 | 0.001197892 | 0.001411975 | 0.000875048 |
| d__Bacteria;k__norank_d__Bacteria;p__Proteobacteria;c__Alphaproteobacteria;o__Sphingomonadales;f__Sphingomonadaceae;g__Novosphingobium | 0.002624328 | 0.000667077 | 0.000980897 | 0.003152071 | 0.001471254 | 0.001105265 | 0.000810119 | 0.000414884 | 0.00025601 | 0.000431241 | 0.000619287 | 0.000308841 |
| d__Bacteria;k__norank_d__Bacteria;p__Verrucomicrobiota;c__Verrucomicrobiae;o__Chthoniobacterales;f__Chthoniobacteraceae;g__Chthoniobacter | 0.000734812 | 0.001975575 | 0.003163393 | 0.000750493 | 0.000995926 | 0.001746319 | 0.000470391 | 5.19E-05 | 0.000102404 | 0.000383325 | 0.001411975 | 0.000900785 |
| d__Bacteria;k__norank_d__Bacteria;p__Bacteroidota;c__Bacteroidia;o__Chitinophagales;f__Chitinophagaceae;g__Puia | 0.00160084 | 0.00120587 | 0.00171657 | 8.58E-05 | 0.000158443 | 2.21E-05 | 0.00256102 | 0.001659536 | 0.002278487 | 0.000455199 | 0.000396344 | 0.000488998 |
| d__Bacteria;k__norank_d__Bacteria;p__Proteobacteria;c__Alphaproteobacteria;o__Acetobacterales;f__Acetobacteraceae;g__norank_f__Acetobacteraceae | 0.00144338 | 0.002001232 | 0.001348733 | 4.29E-05 | 0.000248981 | 0.000287369 | 0.00128051 | 0.002800467 | 0.001740867 | 0.000383325 | 0.000594516 | 0.000437524 |
| d__Bacteria;k__norank_d__Bacteria;p__Proteobacteria;c__Alphaproteobacteria;o__unclassified_c__Alphaproteobacteria;f__unclassified_c__Alphaproteobacteria;g__unclassified_c__Alphaproteobacteria | 0.00160084 | 0.001282841 | 0.00171657 | 0.000278755 | 0.001131734 | 0.000618949 | 0.000679454 | 0.00106314 | 0.000896034 | 0.001006229 | 0.001486289 | 0.000823575 |
| d__Bacteria;k__norank_d__Bacteria;p__Actinobacteriota;c__Actinobacteria;o__Pseudonocardiales;f__Pseudonocardiaceae;g__Lechevalieria | 0.002230678 | 0.000128284 | 0.000588538 | 0.001222232 | 0.000973291 | 0.000773686 | 0.000156797 | 0.000155581 | 0.00025601 | 0.000215621 | 0.005474498 | 0.00015442 |
| d__Bacteria;k__norank_d__Bacteria;p__Firmicutes;c__Clostridia;o__Lachnospirales;f__Lachnospiraceae;g__unclassified_f__Lachnospiraceae | 0.002256922 | 0.002052545 | 0.00161848 | 0.000793379 | 0.000995926 | 0.000442106 | 0.000888517 | 0.000959419 | 0.000537621 | 0.000622904 | 0.000569744 | 0.000566208 |
| d__Bacteria;k__norank_d__Bacteria;p__Proteobacteria;c__Gammaproteobacteria;o__Diplorickettsiales;f__Diplorickettsiaceae;g__norank_f__Diplorickettsiaceae | 0.001627083 | 0.002206486 | 0.001961794 | 0.000128656 | 0.000588502 | 0.000486317 | 0.000810119 | 0.00108907 | 0.000742429 | 0.000814566 | 0.000966088 | 0.000746365 |
| d__Bacteria;k__norank_d__Bacteria;p__Proteobacteria;c__Gammaproteobacteria;o__Burkholderiales;f__Burkholderiaceae;g__Cupriavidus | 0.001233434 | 0.001051929 | 0.001005419 | 8.58E-05 | 0.000203712 | 0.000198948 | 0.000601056 | 0.000596396 | 0.001715266 | 0.001245807 | 0.001808318 | 0.002290567 |
| d__Bacteria;k__norank_d__Bacteria;p__Proteobacteria;c__Gammaproteobacteria;o__Burkholderiales;f__Nitrosomonadaceae;g__Nitrosospira | 0.000944758 | 0.001667693 | 0.001005419 | 0.000664723 | 0.000882752 | 0.000663159 | 0.000888517 | 0.001244652 | 0.001203246 | 0.001173934 | 0.001015631 | 0.000617681 |
| d__Bacteria;k__norank_d__Bacteria;p__Acidobacteriota;c__Subgroup_22;o__norank_c__Subgroup_22;f__norank_o__norank_c__Subgroup_22;g__norank_f__norank_o__norank_c__Subgroup_22 | 0.00128592 | 0.001436782 | 0.00152039 | 0.000171541 | 0.000452694 | 0.000287369 | 0.00036586 | 0.000570465 | 0.00079363 | 0.001653091 | 0.001288117 | 0.002136147 |
| d__Bacteria;k__norank_d__Bacteria;p__Proteobacteria;c__Alphaproteobacteria;o__Tistrellales;f__Geminicoccaceae;g__Candidatus_Alysiosphaera | 0.000603595 | 0.000692734 | 0.000564016 | 0.000986362 | 0.001878678 | 0.001127371 | 0.000653321 | 0.000907559 | 0.000921635 | 0.001006229 | 0.001312889 | 0.001183889 |
| d__Bacteria;k__norank_d__Bacteria;p__Actinobacteriota;c__Actinobacteria;o__Propionibacteriales;f__Propionibacteriaceae;g__Microlunatus | 5.25E-05 | 0 | 0.000122612 | 0.002058496 | 0.000701675 | 0.001503161 | 0.000862384 | 0.001452094 | 0.001331251 | 0.000838524 | 0.001535832 | 0.001338309 |
| d__Bacteria;k__norank_d__Bacteria;p__Chloroflexi;c__Gitt-GS-136;o__norank_c__Gitt-GS-136;f__norank_o__norank_c__Gitt-GS-136;g__norank_f__norank_o__norank_c__Gitt-GS-136 | 0.000393649 | 0.000205255 | 7.36E-05 | 0.000836264 | 0.000973291 | 0.001149476 | 0.000940783 | 7.78E-05 | 0.001177645 | 0.001844753 | 0.001684461 | 0.002419251 |
| d__Bacteria;k__norank_d__Bacteria;p__Proteobacteria;c__Alphaproteobacteria;o__Rhizobiales;f__Rhizobiales_Incertae_Sedis;g__Nordella | 0.000866028 | 0.001334154 | 0.001250644 | 0.000664723 | 0.000973291 | 0.000486317 | 0.000391993 | 0.000363023 | 0.00025601 | 0.001629133 | 0.001684461 | 0.001853043 |
| d__Bacteria;k__norank_d__Bacteria;p__Proteobacteria;c__Gammaproteobacteria;o__Diplorickettsiales;f__Diplorickettsiaceae;g__Aquicella | 0.001653326 | 0.001513752 | 0.002280586 | 0.000107213 | 0.000950656 | 0.000309474 | 0.000391993 | 0.000881628 | 0.000537621 | 0.000958313 | 0.000916545 | 0.001209626 |
| d__Bacteria;k__norank_d__Bacteria;p__Proteobacteria;c__Gammaproteobacteria;o__Burkholderiales;f__Comamonadaceae;g__Methylibium | 0.000971001 | 0.000487479 | 0.00071115 | 0.000428853 | 0.000633771 | 0.000773686 | 0.001202112 | 0.001685466 | 0.001868872 | 0.000646862 | 0.001263346 | 0.001029469 |
| d__Bacteria;k__norank_d__Bacteria;p__Dependentiae;c__Babeliae;o__Babeliales;f__Vermiphilaceae;g__norank_f__Vermiphilaceae | 0.001758299 | 0.001924261 | 0.002010839 | 0.000536067 | 0.000430059 | 0.00037579 | 0.000836252 | 0.000855698 | 0.000691227 | 0.000958313 | 0.000544973 | 0.000591944 |
| d__Bacteria;k__norank_d__Bacteria;p__Bacteroidota;c__Bacteroidia;o__Chitinophagales;f__Chitinophagaceae;g__Flavisolibacter | 0.000892271 | 0.000436166 | 0.000760195 | 0.001265117 | 0.001199638 | 0.001923162 | 0.00201223 | 0.001296512 | 0.000614424 | 0.000431241 | 0.000272486 | 0.000360314 |
| d__Bacteria;k__norank_d__Bacteria;p__Firmicutes;c__Clostridia;o__Peptostreptococcales-Tissierellales;f__Peptostreptococcaceae;g__Romboutsia | 0.002466868 | 0.001436782 | 0.001839182 | 0.001758298 | 0.000497963 | 0.00070737 | 0.00091465 | 0.000596396 | 0.000486419 | 0.000143747 | 0.000148629 | 0.000411787 |
| d__Bacteria;k__norank_d__Bacteria;p__Bacteroidota;c__Bacteroidia;o__Chitinophagales;f__Chitinophagaceae;g__Edaphobaculum | 0.000524866 | 0.000692734 | 0.000613061 | 0.000278755 | 0.000407424 | 0.001635793 | 0.002351957 | 0.001166861 | 0.001945675 | 0.000790609 | 0.000619287 | 0.000360314 |
| d__Bacteria;k__norank_d__Bacteria;p__Proteobacteria;c__Alphaproteobacteria;o__Rhizobiales;f__A0839;g__norank_f__A0839 | 0.001233434 | 0.001308498 | 0.001765615 | 0.000300197 | 0.000814848 | 0.000508422 | 0.000496524 | 0.000492675 | 0.000896034 | 0.001269765 | 0.001288117 | 0.000875048 |
| d__Bacteria;k__norank_d__Bacteria;p__Chloroflexi;c__unclassified_p__Chloroflexi;o__unclassified_p__Chloroflexi;f__unclassified_p__Chloroflexi;g__unclassified_p__Chloroflexi | 0.000656082 | 0.000615764 | 0.000490449 | 0.000686165 | 0.00067904 | 0.000464212 | 0.001228244 | 0.002333722 | 0.000972837 | 0.001533301 | 0.000520201 | 0.000977995 |
| d__Bacteria;k__norank_d__Bacteria;p__Verrucomicrobiota;c__Chlamydiae;o__Chlamydiales;f__Parachlamydiaceae;g__Neochlamydia | 0.000708568 | 0.004336002 | 0.002305108 | 0.000793379 | 0.000497963 | 0.000530527 | 0.000209063 | 5.19E-05 | 0.000153606 | 0.000383325 | 0.000594516 | 0.000514734 |
| d__Bacteria;k__norank_d__Bacteria;p__Actinobacteriota;c__Actinobacteria;o__Micrococcales;f__Microbacteriaceae;g__Agromyces | 0.000262433 | 0.000128284 | 0.000122612 | 0.000707608 | 0.00106383 | 0.001967373 | 0.000783986 | 0.00106314 | 0.001203246 | 0.000814566 | 0.001263346 | 0.00164715 |
| d__Bacteria;k__norank_d__Bacteria;p__Desulfobacterota;c__Desulfuromonadia;o__Geobacterales;f__Geobacteraceae;g__Citrifermentans | 0.002125705 | 0.001385468 | 0.002108929 | 0.000214427 | 0.001674966 | 0.00033158 | 0.000391993 | 0.001400233 | 0.000896034 | 0.000383325 | 2.48E-05 | 7.72E-05 |
| d__Bacteria;k__norank_d__Bacteria;p__Bacteroidota;c__Bacteroidia;o__Cytophagales;f__Microscillaceae;g__norank_f__Microscillaceae | 0.001207191 | 0.000744048 | 0.001201599 | 0.000578952 | 0.000792214 | 0.000773686 | 0.001123713 | 0.000414884 | 0.001100842 | 0.000646862 | 0.00151106 | 0.000900785 |
| d__Bacteria;k__norank_d__Bacteria;p__Acidobacteriota;c__Blastocatellia;o__Blastocatellales;f__Blastocatellaceae;g__Tellurimicrobium | 0.000419892 | 0.001590722 | 0.001177076 | 0.000192984 | 0.000316885 | 0.000397896 | 0.001411174 | 0.001322443 | 0.00286731 | 0.000191663 | 0.000371572 | 0.000720628 |
| d__Bacteria;k__norank_d__Bacteria;p__Proteobacteria;c__Gammaproteobacteria;o__unclassified_c__Gammaproteobacteria;f__unclassified_c__Gammaproteobacteria;g__unclassified_c__Gammaproteobacteria | 0.002309408 | 0.001077586 | 0.001397778 | 0.000771936 | 0.000430059 | 0.000442106 | 0.00054879 | 0.000596396 | 0.000409616 | 0.001006229 | 0.001486289 | 0.000463261 |
| d__Bacteria;k__norank_d__Bacteria;p__Firmicutes;c__Bacilli;o__Erysipelotrichales;f__Erysipelotrichaceae;g__Turicibacter | 0.001705813 | 0.001411125 | 0.002157973 | 0.001543872 | 0.000633771 | 0.000221053 | 0.000757853 | 0.000907559 | 0.000358414 | 0.000503115 | 0.00049543 | 0.00015442 |
| d__Bacteria;k__norank_d__Bacteria;p__Proteobacteria;c__Gammaproteobacteria;o__Burkholderiales;f__Nitrosomonadaceae;g__IS-44 | 0.001102218 | 0.001051929 | 0.001324211 | 0.000257312 | 0.000565867 | 0.000751581 | 0.000653321 | 0.001503954 | 0.001152044 | 0.001006229 | 0.000693602 | 0.000720628 |
| d__Bacteria;k__norank_d__Bacteria;p__Actinobacteriota;c__Acidimicrobiia;o__unclassified_c__Acidimicrobiia;f__unclassified_c__Acidimicrobiia;g__unclassified_c__Acidimicrobiia | 0.000341163 | 0.000307882 | 0.000318792 | 0.000600395 | 0.000543232 | 0.001105265 | 0.00054879 | 0.000855698 | 0.000563222 | 0.001916627 | 0.001956947 | 0.001698623 |
| d__Bacteria;k__norank_d__Bacteria;p__Proteobacteria;c__Alphaproteobacteria;o__Rhizobiales;f__Xanthobacteraceae;g__Pseudorhodoplanes | 0.000787298 | 0.000359195 | 0.000490449 | 0.000300197 | 0.000792214 | 0.000840002 | 0.0018293 | 0.001607675 | 0.001868872 | 0.000383325 | 0.00066883 | 0.000643418 |
| d__Bacteria;k__norank_d__Bacteria;p__Actinobacteriota;c__Actinobacteria;o__Frankiales;f__norank_o__Frankiales;g__norank_f__norank_o__Frankiales | 0.000708568 | 0.00064142 | 0.000980897 | 0.000814821 | 0.001471254 | 0.001702109 | 0.000705587 | 0.001478024 | 0.001331251 | 0.000143747 | 0.000346801 | 0.000180157 |
| d__Bacteria;k__norank_d__Bacteria;p__Myxococcota;c__Polyangia;o__Polyangiales;f__Polyangiaceae;g__Pajaroellobacter | 0.001233434 | 0.000974959 | 0.001103509 | 0.001029248 | 0.000837483 | 0.00070737 | 0.000757853 | 0.000648256 | 0.001126443 | 0.000670819 | 0.000594516 | 0.000617681 |
| d__Bacteria;k__norank_d__Bacteria;p__Proteobacteria;c__Alphaproteobacteria;o__Rhizobiales;f__Beijerinckiaceae;g__alphaI_cluster | 0.001075974 | 0.000795361 | 0.000833762 | 0.000493181 | 0.000633771 | 0.000287369 | 0.001228244 | 0.001633606 | 0.00181767 | 0.000359368 | 0.000470658 | 0.000643418 |
| d__Bacteria;k__norank_d__Bacteria;p__Proteobacteria;c__Gammaproteobacteria;o__Steroidobacterales;f__Steroidobacteraceae;g__Steroidobacter | 0.001180947 | 0.001462438 | 0.001275166 | 0.00105069 | 0.000928022 | 0.000464212 | 0.00036586 | 0.000233372 | 0.000230409 | 0.00088644 | 0.001362432 | 0.000772101 |
| d__Bacteria;k__norank_d__Bacteria;p__Actinobacteriota;c__Actinobacteria;o__Frankiales;f__Frankiaceae;g__Jatrophihabitans | 0.000603595 | 0.000461823 | 0.000171657 | 0.000278755 | 4.53E-05 | 0.000132632 | 0.001907699 | 0.004434072 | 0.001843271 | 9.58E-05 | 9.91E-05 | 7.72E-05 |
| d__Bacteria;k__norank_d__Bacteria;p__Proteobacteria;c__Gammaproteobacteria;o__Burkholderiales;f__Comamonadaceae;g__Ramlibacter | 0.000866028 | 0.000744048 | 0.001029942 | 6.43E-05 | 0.000746944 | 0.000641054 | 0.001149846 | 0.001218722 | 0.001126443 | 0.000670819 | 0.001089945 | 0.000669155 |
| d__Bacteria;k__norank_d__Bacteria;p__Proteobacteria;c__Alphaproteobacteria;o__Rhizobiales;f__Kaistiaceae;g__Kaistia | 0.000656082 | 0.000897989 | 0.001152554 | 0.000107213 | 0.000837483 | 0.000685265 | 0.000757853 | 0.000570465 | 0.00104964 | 0.000862482 | 0.000817459 | 0.001441256 |
| d__Bacteria;k__norank_d__Bacteria;p__Proteobacteria;c__Alphaproteobacteria;o__Rhizobiales;f__Hyphomicrobiaceae;g__unclassified_f__Hyphomicrobiaceae | 0.000787298 | 0.001154557 | 0.000735673 | 0.000621837 | 0.000860118 | 0.000574738 | 0.00073172 | 0.000544535 | 0.00104964 | 0.001509344 | 0.000594516 | 0.000617681 |
| d__Bacteria;k__norank_d__Bacteria;p__Proteobacteria;c__Alphaproteobacteria;o__Rhizobiales;f__norank_o__Rhizobiales;g__norank_f__norank_o__Rhizobiales | 0.000682325 | 0.000410509 | 0.000882807 | 0.000385968 | 0.001154368 | 0.000420001 | 0.00036586 | 0.000466744 | 0.000179207 | 0.002012458 | 0.001634918 | 0.001158152 |
| d__Bacteria;k__norank_d__Bacteria;p__Firmicutes;c__Clostridia;o__norank_c__Clostridia;f__Hungateiclostridiaceae;g__Ruminiclostridium | 0.00128592 | 0.001154557 | 0.001226121 | 0.000729051 | 0.001335446 | 0.000884212 | 0.00073172 | 0.001296512 | 0.000358414 | 0.000143747 | 0.000198172 | 0.000283104 |
| d__Bacteria;k__norank_d__Bacteria;p__Verrucomicrobiota;c__Verrucomicrobiae;o__Chthoniobacterales;f__Xiphinematobacteraceae;g__Candidatus_Xiphinematobacter | 0.000472379 | 0.001488095 | 0.001888227 | 0.000257312 | 0.000905387 | 0.00070737 | 0.000104531 | 2.59E-05 | 0.000102404 | 0.000742693 | 0.001263346 | 0.001466993 |
| d__Bacteria;k__norank_d__Bacteria;p__Myxococcota;c__Myxococcia;o__Myxococcales;f__Myxococcaceae;g__P3OB-42 | 0.001312164 | 0.000897989 | 0.001029942 | 0.000729051 | 0.000475328 | 0.000574738 | 0.000653321 | 0.000907559 | 0.000230409 | 0.000694777 | 0.001288117 | 0.000591944 |
| d__Bacteria;k__norank_d__Bacteria;p__Desulfobacterota;c__Desulfuromonadia;o__Geobacterales;f__Geobacteraceae;g__norank_f__Geobacteraceae | 0.00144338 | 0.001513752 | 0.001471346 | 0.000214427 | 0.003191489 | 0.000353685 | 0.000261329 | 0.000648256 | 0.000153606 | 2.40E-05 | 0 | 0.000102947 |
| d__Bacteria;k__norank_d__Bacteria;p__Bacteroidota;c__Bacteroidia;o__Sphingobacteriales;f__AKYH767;g__norank_f__AKYH767 | 0.000892271 | 0.001282841 | 0.001765615 | 0.000150099 | 0.000203712 | 0.000442106 | 0.000783986 | 0.000959419 | 0.001100842 | 0.000407283 | 0.000792687 | 0.000540471 |
| d__Bacteria;k__norank_d__Bacteria;p__Actinobacteriota;c__Acidimicrobiia;o__norank_c__Acidimicrobiia;f__norank_o__norank_c__Acidimicrobiia;g__norank_f__norank_o__norank_c__Acidimicrobiia | 0.000183703 | 0.000282225 | 9.81E-05 | 0.000686165 | 0.001471254 | 0.001657898 | 0.000601056 | 0.001322443 | 0.001331251 | 0.00033541 | 0.000867002 | 0.000437524 |
| d__Bacteria;k__norank_d__Bacteria;p__Myxococcota;c__Myxococcia;o__Myxococcales;f__Myxococcaceae;g__norank_f__Myxococcaceae | 0.001180947 | 0.00064142 | 0.000956375 | 0.0016082 | 0.000769579 | 0.000950528 | 0.000418126 | 0.000544535 | 0.000307212 | 0.000646862 | 0.000619287 | 0.000617681 |
| d__Bacteria;k__norank_d__Bacteria;p__Firmicutes;c__Clostridia;o__Lachnospirales;f__Lachnospiraceae;g__Cellulosilyticum | 0.001653326 | 0.002129516 | 0.001373256 | 0.000836264 | 0.00174287 | 0.00033158 | 0.000156797 | 0.000414884 | 0.000179207 | 0.000167705 | 7.43E-05 | 0.000102947 |
| d__Bacteria;k__norank_d__Bacteria;p__Proteobacteria;c__Alphaproteobacteria;o__Rhizobiales;f__KF-JG30-B3;g__norank_f__KF-JG30-B3 | 0.001810786 | 0.001077586 | 0.001495868 | 0.000385968 | 0.000633771 | 0.000287369 | 0.000444259 | 0.000803838 | 0.000384015 | 0.000742693 | 0.000718373 | 0.000360314 |
| d__Bacteria;k__norank_d__Bacteria;p__Proteobacteria;c__Gammaproteobacteria;o__Xanthomonadales;f__Xanthomonadaceae;g__Pseudoxanthomonas | 0.000288676 | 0 | 0.000735673 | 0.000707608 | 0.000769579 | 0.000994739 | 0.001332776 | 0.000596396 | 0.00079363 | 0.000646862 | 0.00133766 | 0.000849312 |
| d__Bacteria;k__norank_d__Bacteria;p__Proteobacteria;c__Alphaproteobacteria;o__Caulobacterales;f__Caulobacteraceae;g__Caulobacter | 0.000787298 | 0.000256568 | 0.000662105 | 0.000364525 | 0.000746944 | 0.001193687 | 0.001411174 | 0.000674186 | 0.001228847 | 0.000503115 | 0.00066883 | 0.000540471 |
| d__Bacteria;k__norank_d__Bacteria;p__Chloroflexi;c__Anaerolineae;o__Ardenticatenales;f__norank_o__Ardenticatenales;g__norank_f__norank_o__Ardenticatenales | 0.000419892 | 0.000333539 | 0.000490449 | 0.000922034 | 0.002218198 | 0.000884212 | 0.000653321 | 0.000103721 | 0.000281611 | 0.001054145 | 0.000817459 | 0.000849312 |
| d__Bacteria;k__norank_d__Bacteria;p__Bdellovibrionota;c__Bdellovibrionia;o__Bacteriovoracales;f__Bacteriovoracaceae;g__Peredibacter | 0.000813542 | 0.000795361 | 0.000662105 | 0.000621837 | 0.001041195 | 0.001436845 | 0.000261329 | 0.000207442 | 0.000332813 | 0.000790609 | 0.001436746 | 0.000514734 |
| d__Bacteria;k__norank_d__Bacteria;p__Proteobacteria;c__Gammaproteobacteria;o__Gammaproteobacteria_Incertae_Sedis;f__unclassified_o__Gammaproteobacteria_Incertae_Sedis;g__norank_f__unclassified | 0.000131216 | 0.000538793 | 0.000637583 | 8.58E-05 | 2.26E-05 | 6.63E-05 | 0.000261329 | 5.19E-05 | 0 | 0.002275994 | 0.00200649 | 0.002779565 |
| d__Bacteria;k__norank_d__Bacteria;p__Firmicutes;c__Clostridia;o__Clostridiales;f__Clostridiaceae;g__Clostridium_sensu_stricto_12 | 0.001049731 | 0.001000616 | 0.000833762 | 0.000814821 | 0.000611136 | 0.000751581 | 0.001306643 | 0.001503954 | 0.000307212 | 0.000359368 | 9.91E-05 | 0.000205894 |
| d__Bacteria;k__norank_d__Bacteria;p__Verrucomicrobiota;c__Verrucomicrobiae;o__Pedosphaerales;f__Pedosphaeraceae;g__norank_f__Pedosphaeraceae | 0.000892271 | 0.002026888 | 0.002869124 | 0.000128656 | 0.000362155 | 0.000353685 | 0.00036586 | 2.59E-05 | 0.000102404 | 9.58E-05 | 0.000792687 | 0.000823575 |
| d__Bacteria;k__norank_d__Bacteria;p__RCP2-54;c__norank_p__RCP2-54;o__norank_c__norank_p__RCP2-54;f__norank_o__norank_c__norank_p__RCP2-54;g__norank_f__norank_o__norank_c__norank_p__RCP2-54 | 0.001732056 | 0.001231527 | 0.00152039 | 0.000300197 | 0.000362155 | 0.000243158 | 0.000470391 | 0.000518605 | 0.000537621 | 0.000598946 | 0.000718373 | 0.000566208 |
| d__Bacteria;k__norank_d__Bacteria;p__Proteobacteria;c__Alphaproteobacteria;o__Sphingomonadales;f__Sphingomonadaceae;g__Sphingobium | 0.000603595 | 0.000102627 | 0.000490449 | 0.000536067 | 0.000701675 | 0.00070737 | 0.001515706 | 0.000544535 | 0.000768029 | 0.001269765 | 0.000644059 | 0.000849312 |
| d__Bacteria;k__norank_d__Bacteria;p__Acidobacteriota;c__Blastocatellia;o__DS-100;f__norank_o__DS-100;g__norank_f__norank_o__DS-100 | 0.000524866 | 0.000692734 | 0.00071115 | 0.000450296 | 0.000520598 | 0.000486317 | 2.61E-05 | 2.59E-05 | 0.000332813 | 0.000934356 | 0.001387203 | 0.002547935 |
| d__Bacteria;k__norank_d__Bacteria;p__Proteobacteria;c__Gammaproteobacteria;o__Xanthomonadales;f__Rhodanobacteraceae;g__Rhodanobacter | 0.001574597 | 0.001488095 | 0.002918169 | 6.43E-05 | 0 | 4.42E-05 | 0.000757853 | 0.00108907 | 0.000640025 | 2.40E-05 | 2.48E-05 | 0 |
| d__Bacteria;k__norank_d__Bacteria;p__Patescibacteria;c__Parcubacteria;o__norank_c__Parcubacteria;f__norank_o__norank_c__Parcubacteria;g__norank_f__norank_o__norank_c__Parcubacteria | 0.000839785 | 0.000974959 | 0.001201599 | 0.000300197 | 0.000543232 | 0.000265264 | 0.000470391 | 0.000596396 | 0.000768029 | 0.000958313 | 0.000966088 | 0.000720628 |
| d__Bacteria;k__norank_d__Bacteria;p__Proteobacteria;c__Alphaproteobacteria;o__Rhizobiales;f__unclassified_o__Rhizobiales;g__unclassified_o__Rhizobiales | 0.000577352 | 0.000487479 | 0.000882807 | 0.000192984 | 0.000882752 | 0.000397896 | 0.000862384 | 0.000570465 | 0.000409616 | 0.000790609 | 0.001461518 | 0.000720628 |
| d__Bacteria;k__norank_d__Bacteria;p__Proteobacteria;c__Gammaproteobacteria;o__Burkholderiales;f__Comamonadaceae;g__Polaromonas | 0.000419892 | 0.000667077 | 0.00090733 | 0.000192984 | 0.000633771 | 6.63E-05 | 2.61E-05 | 2.59E-05 | 0 | 0.002036416 | 0.00183309 | 0.001415519 |
| d__Bacteria;k__norank_d__Bacteria;p__Firmicutes;c__Clostridia;o__Clostridiales;f__Clostridiaceae;g__Clostridium_sensu_stricto_8 | 0.001548353 | 0.00064142 | 0.000931852 | 0.000707608 | 0.000430059 | 0.000464212 | 0.000940783 | 0.001478024 | 0.000384015 | 0.00033541 | 9.91E-05 | 0.00023163 |
| d__Bacteria;k__norank_d__Bacteria;p__Firmicutes;c__Clostridia;o__norank_c__Clostridia;f__Hungateiclostridiaceae;g__norank_f__Hungateiclostridiaceae | 0.00152211 | 0.001000616 | 0.001078987 | 0.000707608 | 0.00067904 | 0.000420001 | 0.000757853 | 0.000907559 | 0.000204808 | 0.000215621 | 0.000247715 | 0.000283104 |
| d__Bacteria;k__norank_d__Bacteria;p__Actinobacteriota;c__Actinobacteria;o__Micrococcales;f__Micrococcaceae;g__Paenarthrobacter | 0.000236189 | 0.000949302 | 0.000735673 | 6.43E-05 | 0.00033952 | 0.000596843 | 0.000966916 | 0.00108907 | 0.000691227 | 0.00122185 | 0.000445887 | 0.000669155 |
| d__Bacteria;k__norank_d__Bacteria;p__Firmicutes;c__Bacilli;o__Paenibacillales;f__Paenibacillaceae;g__Cohnella | 0.000971001 | 0.001000616 | 0.000735673 | 0.000514624 | 0.000520598 | 0.00033158 | 0.000679454 | 0.000803838 | 0.000614424 | 0.00055103 | 0.000569744 | 0.000669155 |
| d__Bacteria;k__norank_d__Bacteria;p__Proteobacteria;c__Alphaproteobacteria;o__Azospirillales;f__Azospirillaceae;g__Skermanella | 0.00015746 | 7.70E-05 | 0.000465926 | 0.001886954 | 0.000362155 | 0.000685265 | 0.00036586 | 0.000388954 | 0.000204808 | 0.000862482 | 0.001288117 | 0.001209626 |
| d__Bacteria;k__norank_d__Bacteria;p__Firmicutes;c__Bacilli;o__Brevibacillales;f__Brevibacillaceae;g__Brevibacillus | 0.000577352 | 0.000615764 | 0.000490449 | 0.000557509 | 0.000203712 | 0.000309474 | 0.00091465 | 0.000829768 | 0.000281611 | 0.001126018 | 0.001139488 | 0.000900785 |
| d__Bacteria;k__norank_d__Bacteria;p__Bacteroidota;c__Bacteroidia;o__Cytophagales;f__Spirosomaceae;g__Dyadobacter | 0.000892271 | 0.00056445 | 0.001103509 | 0.00128656 | 0.001561793 | 0.001348424 | 0.000339727 | 0.000103721 | 0.000102404 | 0.000143747 | 0.000247715 | 0.00023163 |
| d__Bacteria;k__norank_d__Bacteria;p__Proteobacteria;c__Gammaproteobacteria;o__Burkholderiales;f__B1-7BS;g__norank_f__B1-7BS | 0.000682325 | 0.000513136 | 0.001005419 | 0.001157904 | 0.000837483 | 0.000287369 | 0.000339727 | 0.000207442 | 0.000204808 | 0.000694777 | 0.001288117 | 0.000694891 |
| d__Bacteria;k__norank_d__Bacteria;p__Bacteroidota;c__Bacteroidia;o__Sphingobacteriales;f__Sphingobacteriaceae;g__unclassified_f__Sphingobacteriaceae | 0.000236189 | 0.000153941 | 0.000147135 | 0.005360666 | 0.000543232 | 0.001105265 | 0.000104531 | 2.59E-05 | 5.12E-05 | 4.79E-05 | 9.91E-05 | 2.57E-05 |
| d__Bacteria;k__norank_d__Bacteria;p__Proteobacteria;c__Gammaproteobacteria;o__CCD24;f__norank_o__CCD24;g__norank_f__norank_o__CCD24 | 0.001075974 | 0.000590107 | 0.000539493 | 0.000578952 | 0.00067904 | 0.000530527 | 0.000601056 | 0.000674186 | 0.000742429 | 0.000359368 | 0.000817459 | 0.000566208 |
| d__Bacteria;k__norank_d__Bacteria;p__Acidobacteriota;c__Acidobacteriae;o__Acidobacteriales;f__Acidobacteriaceae_Subgroup_1;g__norank_f__Acidobacteriaceae_Subgroup_1 | 0.000787298 | 0.001436782 | 0.001078987 | 0.000536067 | 0.000294251 | 0.000353685 | 0.00091465 | 0.000803838 | 0.000998438 | 0.000119789 | 0.000123857 | 0.000205894 |
| d__Bacteria;k__norank_d__Bacteria;p__Acidobacteriota;c__Acidobacteriae;o__norank_c__Acidobacteriae;f__norank_o__norank_c__Acidobacteriae;g__Paludibaculum | 0.000866028 | 0.000974959 | 0.001275166 | 0.000364525 | 0.000611136 | 0.000243158 | 0.000391993 | 0.000492675 | 0.001177645 | 0.000215621 | 0.000544973 | 0.000386051 |
| d__Bacteria;k__norank_d__Bacteria;p__Proteobacteria;c__Alphaproteobacteria;o__Rhizobiales;f__D05-2;g__norank_f__D05-2 | 0.000367406 | 0.000230911 | 0.000564016 | 0.000407411 | 0.000248981 | 0.000751581 | 0.001358909 | 0.000855698 | 0.001715266 | 0.000191663 | 0.000371572 | 0.000334577 |
| d__Bacteria;k__norank_d__Bacteria;p__Desulfobacterota;c__Desulfuromonadia;o__Geobacterales;f__Geobacteraceae;g__Geobacter | 0.001390894 | 0.0011289 | 0.001593958 | 4.29E-05 | 0.001380715 | 0.000198948 | 0.000313594 | 0.000648256 | 0.000204808 | 0.000215621 | 7.43E-05 | 0.000102947 |
| d__Bacteria;k__norank_d__Bacteria;p__Proteobacteria;c__Gammaproteobacteria;o__Steroidobacterales;f__Steroidobacteraceae;g__norank_f__Steroidobacteraceae | 0.000918515 | 0.000384852 | 0.001177076 | 0.00064328 | 0.000746944 | 0.00037579 | 0.000235196 | 7.78E-05 | 0.000588823 | 0.00055103 | 0.000792687 | 0.000643418 |
| d__Bacteria;k__norank_d__Bacteria;p__Proteobacteria;c__Alphaproteobacteria;o__Caulobacterales;f__Hyphomonadaceae;g__Hirschia | 0.000918515 | 0.000538793 | 0.000760195 | 0.00032164 | 0.000384789 | 0.000552633 | 0.00073172 | 0.000492675 | 0.000768029 | 0.000431241 | 0.000941316 | 0.000283104 |
| d__Bacteria;k__norank_d__Bacteria;p__Proteobacteria;c__Alphaproteobacteria;o__Sphingomonadales;f__Sphingomonadaceae;g__Sphingopyxis | 0.000787298 | 0.000538793 | 0.000784718 | 0.000471739 | 0.001493889 | 0.000751581 | 0.000339727 | 0.000103721 | 0.000435217 | 0.000503115 | 0.000693602 | 0.000205894 |
| d__Bacteria;k__norank_d__Bacteria;p__Proteobacteria;c__Gammaproteobacteria;o__R7C24;f__norank_o__R7C24;g__norank_f__norank_o__R7C24 | 0.000551109 | 0.000692734 | 0.000613061 | 8.58E-05 | 0.000248981 | 0.000353685 | 0.00109758 | 0.000596396 | 0.000537621 | 0.00110206 | 0.000222943 | 0.000926522 |
| d__Bacteria;k__norank_d__Bacteria;p__Proteobacteria;c__Alphaproteobacteria;o__Defluviicoccales;f__Defluviicoccaceae;g__Defluviicoccus | 0.001154704 | 0.000590107 | 0.000367836 | 0.001200789 | 0.000203712 | 0.000751581 | 0.000601056 | 0.000155581 | 0.000307212 | 0.000622904 | 0.000346801 | 0.000643418 |
| d__Bacteria;k__norank_d__Bacteria;p__Bacteroidota;c__SJA-28;o__norank_c__SJA-28;f__norank_o__norank_c__SJA-28;g__norank_f__norank_o__norank_c__SJA-28 | 0.001154704 | 0.001334154 | 0.001029942 | 4.29E-05 | 0.000316885 | 0.000198948 | 0.000261329 | 0.000285233 | 0.000460818 | 0.000718735 | 0.000767916 | 0.000360314 |
| d__Bacteria;k__norank_d__Bacteria;p__Proteobacteria;c__Alphaproteobacteria;o__Caulobacterales;f__Caulobacteraceae;g__norank_f__Caulobacteraceae | 0.000288676 | 0.000230911 | 0.000392359 | 0.000171541 | 0.000407424 | 0.000552633 | 0.001202112 | 0.000907559 | 0.001868872 | 0.000239578 | 0.000247715 | 0.000334577 |
| d__Bacteria;k__norank_d__Bacteria;p__Proteobacteria;c__Alphaproteobacteria;o__Rhizobiales;f__Xanthobacteraceae;g__Rhodopseudomonas | 0.001023488 | 0.000718391 | 0.000956375 | 0.000493181 | 0.000520598 | 0.000486317 | 0.000287461 | 0.000544535 | 0.000409616 | 0.000359368 | 0.00049543 | 0.000540471 |
| d__Bacteria;k__norank_d__Bacteria;p__Acidobacteriota;c__AT-s3-28;o__norank_c__AT-s3-28;f__norank_o__norank_c__AT-s3-28;g__norank_f__norank_o__norank_c__AT-s3-28 | 0.000341163 | 0.000461823 | 0.000490449 | 0.00032164 | 0.000452694 | 0.000132632 | 0.000391993 | 0.000388954 | 0.000460818 | 0.001293723 | 0.000619287 | 0.001466993 |
| d__Bacteria;k__norank_d__Bacteria;p__Dadabacteria;c__Dadabacteriia;o__Dadabacteriales;f__norank_o__Dadabacteriales;g__norank_f__norank_o__Dadabacteriales | 0.000944758 | 0.000718391 | 0.000490449 | 0.000257312 | 0.000701675 | 0.00037579 | 0.000130664 | 0.000129651 | 5.12E-05 | 0.001485386 | 0.000569744 | 0.000720628 |
| d__Bacteria;k__norank_d__Bacteria;p__Actinobacteriota;c__Actinobacteria;o__Frankiales;f__unclassified_o__Frankiales;g__unclassified_o__Frankiales | 7.87E-05 | 0.000256568 | 0.000171657 | 0.000450296 | 0.000226347 | 0.000596843 | 0.00091465 | 0.001244652 | 0.001100842 | 0.000239578 | 0.000619287 | 0.000669155 |
| d__Bacteria;k__norank_d__Bacteria;p__Bacteroidota;c__Bacteroidia;o__Chitinophagales;f__Chitinophagaceae;g__unclassified_f__Chitinophagaceae | 0.000761055 | 0.000692734 | 0.00080924 | 0.000471739 | 0.000475328 | 0.00103895 | 0.000391993 | 0.000311163 | 0.000435217 | 0.000383325 | 0.000371572 | 0.000386051 |
| d__Bacteria;k__norank_d__Bacteria;p__Actinobacteriota;c__Actinobacteria;o__Micrococcales;f__Intrasporangiaceae;g__Terrabacter | 7.87E-05 | 0.000128284 | 0.000269747 | 0.000814821 | 0.000475328 | 0.000530527 | 0.001123713 | 0.000907559 | 0.000896034 | 0.000311452 | 0.000272486 | 0.000694891 |
| d__Bacteria;k__norank_d__Bacteria;p__Proteobacteria;c__Gammaproteobacteria;o__Burkholderiales;f__Comamonadaceae;g__Rhizobacter | 0.000629839 | 0.000487479 | 0.000686628 | 0.000214427 | 0.000316885 | 0.000464212 | 0.000574923 | 0.000959419 | 0.001100842 | 0.00033541 | 0.000346801 | 0.000386051 |
| d__Bacteria;k__norank_d__Bacteria;p__Firmicutes;c__Clostridia;o__Clostridiales;f__Clostridiaceae;g__Clostridium_sensu_stricto_13 | 0.001627083 | 0.000718391 | 0.000637583 | 0.000343083 | 0.000565867 | 0.000221053 | 0.001123713 | 0.000311163 | 0.000281611 | 0.000263536 | 0.000148629 | 0.000180157 |
| d__Bacteria;k__norank_d__Bacteria;p__Actinobacteriota;c__Actinobacteria;o__Frankiales;f__Acidothermaceae;g__Acidothermus | 0.000997244 | 0.000487479 | 0.000564016 | 0.000150099 | 4.53E-05 | 0.000110527 | 0.001202112 | 0.001633606 | 0.000998438 | 0.000119789 | 2.48E-05 | 5.15E-05 |
| d__Bacteria;k__norank_d__Bacteria;p__Bacteroidota;c__Bacteroidia;o__Chitinophagales;f__Chitinophagaceae;g__Aurantisolimonas | 0.000603595 | 0.000795361 | 0.000539493 | 0.000343083 | 0.001199638 | 0.000397896 | 0.00036586 | 0.000363023 | 0.000614424 | 0.000407283 | 0.00049543 | 0.000257367 |
| d__Bacteria;k__norank_d__Bacteria;p__Actinobacteriota;c__Actinobacteria;o__Frankiales;f__Sporichthyaceae;g__norank_f__Sporichthyaceae | 0.000236189 | 0.000205255 | 0.000269747 | 0.000943477 | 0.000430059 | 0.000420001 | 0.000313594 | 0.00108907 | 0.001152044 | 0.000598946 | 0.000346801 | 0.000308841 |
| d__Bacteria;k__norank_d__Bacteria;p__Proteobacteria;c__Alphaproteobacteria;o__Rhizobiales;f__Devosiaceae;g__norank_f__Devosiaceae | 0.000288676 | 0.000128284 | 0.000416881 | 0.00064328 | 0.000746944 | 0.00037579 | 0.001567972 | 0.000518605 | 0.000665626 | 0.000383325 | 0.000322029 | 0.000180157 |
| d__Bacteria;k__norank_d__Bacteria;p__Myxococcota;c__Myxococcia;o__Myxococcales;f__Myxococcaceae;g__Archangium | 0.000551109 | 0.000205255 | 0.000318792 | 0.000578952 | 0.000633771 | 0.000773686 | 0.00018293 | 0.000233372 | 0.000460818 | 0.000287494 | 0.001288117 | 0.000694891 |
| d__Bacteria;k__norank_d__Bacteria;p__Proteobacteria;c__Gammaproteobacteria;o__Burkholderiales;f__Sutterellaceae;g__norank_f__Sutterellaceae | 0.000892271 | 0.000436166 | 0.000760195 | 0.00032164 | 0.000928022 | 0.000552633 | 0.000156797 | 0.000466744 | 0.000204808 | 0.00055103 | 0.000520201 | 0.000411787 |
| d__Bacteria;k__norank_d__Bacteria;p__Bacteroidota;c__Bacteroidia;o__Chitinophagales;f__Chitinophagaceae;g__Dinghuibacter | 0.000656082 | 0.000821018 | 0.001324211 | 0.000257312 | 0.000430059 | 0.000442106 | 0.000287461 | 0.000311163 | 0.000588823 | 0.000287494 | 0.000322029 | 0.000411787 |
| d__Bacteria;k__norank_d__Bacteria;p__Actinobacteriota;c__Thermoleophilia;o__norank_c__Thermoleophilia;f__norank_o__norank_c__Thermoleophilia;g__norank_f__norank_o__norank_c__Thermoleophilia | 0.000236189 | 2.57E-05 | 0.000122612 | 0.001350888 | 0.000633771 | 0.000596843 | 0.000287461 | 0.000233372 | 0.000128005 | 0.000838524 | 0.000990859 | 0.000669155 |
| d__Bacteria;k__norank_d__Bacteria;p__WPS-2;c__norank_p__WPS-2;o__norank_c__norank_p__WPS-2;f__norank_o__norank_c__norank_p__WPS-2;g__norank_f__norank_o__norank_c__norank_p__WPS-2 | 0.000183703 | 0.000205255 | 0.000343314 | 0 | 4.53E-05 | 4.42E-05 | 0.001620237 | 0.001244652 | 0.001254448 | 0.000383325 | 0.000371572 | 0.000386051 |
| d__Bacteria;k__norank_d__Bacteria;p__Cyanobacteria;c__Sericytochromatia;o__norank_c__Sericytochromatia;f__norank_o__norank_c__Sericytochromatia;g__norank_f__norank_o__norank_c__Sericytochromatia | 7.87E-05 | 0.000307882 | 0.000343314 | 0.000471739 | 0.000995926 | 0.001702109 | 0.000209063 | 0.000311163 | 0.00051202 | 0.00033541 | 0.000569744 | 0.000180157 |
| d__Bacteria;k__norank_d__Bacteria;p__Acidobacteriota;c__Acidobacteriae;o__Acidobacteriales;f__Acidobacteriaceae_Subgroup_1;g__Edaphobacter | 0.000498622 | 0.000410509 | 0.000514971 | 0.000107213 | 4.53E-05 | 4.42E-05 | 0.001332776 | 0.000959419 | 0.001766468 | 4.79E-05 | 2.48E-05 | 0.000257367 |
| d__Bacteria;k__norank_d__Bacteria;p__Proteobacteria;c__Alphaproteobacteria;o__Rhizobiales;f__Rhizobiaceae;g__Aliihoeflea | 0.000656082 | 0.000205255 | 0.000245224 | 0.000793379 | 0.001652331 | 0.001127371 | 0.000313594 | 0.000207442 | 0.000332813 | 4.79E-05 | 0.000297258 | 0.000128684 |
| d__Bacteria;k__norank_d__Bacteria;p__Proteobacteria;c__Alphaproteobacteria;o__Sphingomonadales;f__Sphingomonadaceae;g__Sphingorhabdus | 0.000393649 | 0.000256568 | 0.000220702 | 0.000407411 | 0.001222273 | 0.001149476 | 5.23E-05 | 0.000181512 | 0.000102404 | 0.000311452 | 0.001411975 | 0.000257367 |
| d__Bacteria;k__norank_d__Bacteria;p__Bdellovibrionota;c__Bdellovibrionia;o__Bdellovibrionales;f__Bdellovibrionaceae;g__OM27_clade | 0.000551109 | 0.000513136 | 0.000490449 | 0.000557509 | 0.000973291 | 0.000530527 | 0.000261329 | 0.000311163 | 0.000102404 | 0.000598946 | 0.000346801 | 0.000720628 |
| d__Bacteria;k__norank_d__Bacteria;p__Firmicutes;c__Bacilli;o__Bacillales;f__Planococcaceae;g__Rummeliibacillus | 0.000393649 | 0.000461823 | 0.000686628 | 0.002551677 | 0.000520598 | 0.000287369 | 0.000261329 | 0.000103721 | 5.12E-05 | 0.000263536 | 0.000322029 | 5.15E-05 |
| d__Bacteria;k__norank_d__Bacteria;p__Verrucomicrobiota;c__Chlamydiae;o__Chlamydiales;f__Parachlamydiaceae;g__unclassified_f__Parachlamydiaceae | 0.000472379 | 0.002565681 | 0.001078987 | 0.000428853 | 0.000158443 | 0.000176842 | 0.000444259 | 7.78E-05 | 0 | 0.000191663 | 9.91E-05 | 0.00023163 |
| d__Bacteria;k__norank_d__Bacteria;p__Proteobacteria;c__Gammaproteobacteria;o__JG36-TzT-191;f__norank_o__JG36-TzT-191;g__norank_f__norank_o__JG36-TzT-191 | 0.000944758 | 0.000718391 | 0.000318792 | 0.000235869 | 2.26E-05 | 0.000132632 | 0.000705587 | 0.001244652 | 0.00079363 | 0.000263536 | 0.000123857 | 0.000334577 |
| d__Bacteria;k__norank_d__Bacteria;p__Myxococcota;c__Polyangia;o__Polyangiales;f__Phaselicystidaceae;g__Phaselicystis | 0.000656082 | 0.00056445 | 0.000662105 | 0.000428853 | 0.000430059 | 0.000397896 | 0.000574923 | 0.000544535 | 0.000537621 | 0.000383325 | 0.000272486 | 0.000386051 |
| d__Bacteria;k__norank_d__Bacteria;p__Proteobacteria;c__Gammaproteobacteria;o__Legionellales;f__Legionellaceae;g__Legionella | 0.001338407 | 0.000846675 | 0.000735673 | 0.000214427 | 0.00033952 | 0.000221053 | 0.000391993 | 0.000518605 | 0.000307212 | 0.000311452 | 0.000247715 | 0.000360314 |
| d__Bacteria;k__norank_d__Bacteria;p__Proteobacteria;c__Gammaproteobacteria;o__Xanthomonadales;f__Rhodanobacteraceae;g__norank_f__Rhodanobacteraceae | 0 | 5.13E-05 | 0.000147135 | 0.000986362 | 0.001425985 | 0.001680004 | 5.23E-05 | 5.19E-05 | 7.68E-05 | 0.000431241 | 0.000544973 | 0.000334577 |
| d__Bacteria;k__norank_d__Bacteria;p__Actinobacteriota;c__Actinobacteria;o__Micrococcales;f__Intrasporangiaceae;g__norank_f__Intrasporangiaceae | 0.000131216 | 0.000179598 | 0.000220702 | 0.000471739 | 0.000792214 | 0.000928423 | 0.000810119 | 0.000726047 | 0.000716828 | 0.00033541 | 0.000148629 | 0.00023163 |
| d__Bacteria;k__norank_d__Bacteria;p__Verrucomicrobiota;c__Verrucomicrobiae;o__Verrucomicrobiales;f__Rubritaleaceae;g__Luteolibacter | 0.000524866 | 0.000923645 | 0.000956375 | 0.000150099 | 0.000384789 | 0.000309474 | 0.00036586 | 7.78E-05 | 0.000179207 | 0.000431241 | 0.00084223 | 0.000463261 |
| d__Bacteria;k__norank_d__Bacteria;p__Acidobacteriota;c__Subgroup_18;o__norank_c__Subgroup_18;f__norank_o__norank_c__Subgroup_18;g__norank_f__norank_o__norank_c__Subgroup_18 | 0.000761055 | 0.000923645 | 0.00080924 | 0.000750493 | 0.000430059 | 0.000530527 | 0.000156797 | 5.19E-05 | 0.000435217 | 0.000263536 | 0.000198172 | 0.000283104 |
| d__Bacteria;k__norank_d__Bacteria;p__Firmicutes;c__Desulfitobacteriia;o__Desulfitobacteriales;f__Desulfitobacteriaceae;g__Desulfosporosinus | 0.001653326 | 0.00064142 | 0.001275166 | 0.000471739 | 0.000452694 | 0.000221053 | 0.000156797 | 0.000259302 | 0.000179207 | 2.40E-05 | 0.000222943 | 2.57E-05 |
| d__Bacteria;k__norank_d__Bacteria;p__Actinobacteriota;c__Actinobacteria;o__Micromonosporales;f__Micromonosporaceae;g__Luedemannella | 0.000498622 | 0.000359195 | 0.000294269 | 0.000493181 | 0.00033952 | 0.000397896 | 0.000496524 | 0.001296512 | 0.000486419 | 0.000383325 | 0.000297258 | 0.00023163 |
| d__Bacteria;k__norank_d__Bacteria;p__Myxococcota;c__Myxococcia;o__Myxococcales;f__Anaeromyxobacteraceae;g__Anaeromyxobacter | 0.000839785 | 0.000461823 | 0.000367836 | 0.000771936 | 0.000565867 | 0.000442106 | 0.000444259 | 0.000363023 | 0.000204808 | 0.000215621 | 0.000445887 | 0.000437524 |
| d__Bacteria;k__norank_d__Bacteria;p__Actinobacteriota;c__Actinobacteria;o__Streptosporangiales;f__Streptosporangiaceae;g__Streptosporangium | 0.000446136 | 0.000384852 | 0.000514971 | 0.000578952 | 0.000520598 | 0.000574738 | 0.000339727 | 0.000518605 | 0.000486419 | 0.000527072 | 0.00049543 | 0.00015442 |
| d__Bacteria;k__norank_d__Bacteria;p__Proteobacteria;c__Gammaproteobacteria;o__KI89A_clade;f__norank_o__KI89A_clade;g__norank_f__norank_o__KI89A_clade | 0.000892271 | 0.000692734 | 0.001005419 | 0.000214427 | 0.000362155 | 0.000221053 | 0.000156797 | 0.000311163 | 0.000358414 | 0.000431241 | 0.000371572 | 0.000386051 |
| d__Bacteria;k__norank_d__Bacteria;p__Proteobacteria;c__Alphaproteobacteria;o__Acetobacterales;f__Acetobacteraceae;g__Roseomonas | 0.000341163 | 0.000307882 | 0.000220702 | 0.00105069 | 0.000769579 | 0.000442106 | 0.00036586 | 0.000440814 | 0.000409616 | 0.000311452 | 0.000371572 | 0.000360314 |
| d__Bacteria;k__norank_d__Bacteria;p__Bacteroidota;c__Bacteroidia;o__Cytophagales;f__Microscillaceae;g__Ohtaekwangia | 0.000288676 | 0.000333539 | 0.000416881 | 2.14E-05 | 0.000452694 | 0.00037579 | 0.000705587 | 0.000103721 | 0.00051202 | 0.000287494 | 0.001486289 | 0.000386051 |
| d__Bacteria;k__norank_d__Bacteria;p__Actinobacteriota;c__Actinobacteria;o__Propionibacteriales;f__Nocardioidaceae;g__Kribbella | 0.000918515 | 0.000436166 | 0.000588538 | 0.000364525 | 0.000860118 | 0.000596843 | 0.000444259 | 0.000207442 | 0.000691227 | 9.58E-05 | 9.91E-05 | 5.15E-05 |
| d__Bacteria;k__norank_d__Bacteria;p__Acidobacteriota;c__Blastocatellia;o__Blastocatellales;f__Blastocatellaceae;g__Stenotrophobacter | 0.000131216 | 0.000282225 | 0.000269747 | 0.000600395 | 0.000294251 | 0.000265264 | 0.00018293 | 0.000207442 | 0.000409616 | 0.000167705 | 0.000867002 | 0.001672886 |
| d__Bacteria;k__norank_d__Bacteria;p__Nitrospirota;c__4-29-1;o__norank_c__4-29-1;f__norank_o__norank_c__4-29-1;g__norank_f__norank_o__norank_c__4-29-1 | 0.00167957 | 0.000897989 | 0.000956375 | 0.000364525 | 0.000497963 | 0.000287369 | 0.00018293 | 0.000207442 | 2.56E-05 | 7.19E-05 | 0.000123857 | 5.15E-05 |
| d__Bacteria;k__norank_d__Bacteria;p__Armatimonadota;c__norank_p__Armatimonadota;o__norank_c__norank_p__Armatimonadota;f__norank_o__norank_c__norank_p__Armatimonadota;g__norank_f__norank_o__norank_c__norank_p__Armatimonadota | 0.000419892 | 7.70E-05 | 0.000441404 | 0.000557509 | 0.000294251 | 0.000508422 | 0.000601056 | 0.000518605 | 0.00025601 | 0.000383325 | 0.000767916 | 0.000488998 |
| d__Bacteria;k__norank_d__Bacteria;p__Proteobacteria;c__Gammaproteobacteria;o__Cellvibrionales;f__Halieaceae;g__OM60NOR5_clade | 0.000498622 | 0.00064142 | 0.001103509 | 0.000300197 | 0.000520598 | 0.00033158 | 0.000156797 | 0.000440814 | 0.000102404 | 0.000383325 | 0.000445887 | 0.000360314 |
| d__Bacteria;k__norank_d__Bacteria;p__Verrucomicrobiota;c__Chlamydiae;o__Chlamydiales;f__cvE6;g__norank_f__cvE6 | 0.000446136 | 0.001744663 | 0.001741092 | 4.29E-05 | 0.000113173 | 0.000154737 | 0.000104531 | 0.000129651 | 0.000102404 | 0.000191663 | 0.000396344 | 0.000102947 |
| d__Bacteria;k__norank_d__Bacteria;p__Gemmatimonadota;c__Gemmatimonadetes;o__Gemmatimonadales;f__Gemmatimonadaceae;g__unclassified_f__Gemmatimonadaceae | 0.001023488 | 0.000718391 | 0.000514971 | 0.000536067 | 0.000271616 | 0.00033158 | 7.84E-05 | 0.000207442 | 0.000230409 | 0.000574988 | 0.000421115 | 0.000334577 |
| d__Bacteria;k__norank_d__Bacteria;p__Planctomycetota;c__OM190;o__norank_c__OM190;f__norank_o__norank_c__OM190;g__norank_f__norank_o__norank_c__OM190 | 0.000367406 | 0.000744048 | 0.00080924 | 0.000300197 | 0.000407424 | 8.84E-05 | 0.000130664 | 0.000103721 | 0.000358414 | 0.000455199 | 0.000767916 | 0.000694891 |
| d__Bacteria;k__norank_d__Bacteria;p__Proteobacteria;c__Gammaproteobacteria;o__Burkholderiales;f__Alcaligenaceae;g__Achromobacter | 0.000209946 | 0.000205255 | 0.000416881 | 0.000150099 | 0.000497963 | 0.000552633 | 0.000496524 | 0.000207442 | 0.000230409 | 0.000503115 | 0.000817459 | 0.000849312 |
| d__Bacteria;k__norank_d__Bacteria;p__Acidobacteriota;c__Vicinamibacteria;o__Subgroup_9;f__norank_o__Subgroup_9;g__norank_f__norank_o__Subgroup_9 | 0.000209946 | 0.000256568 | 0.000367836 | 0.000235869 | 0.000475328 | 0.000110527 | 0.000156797 | 0 | 2.56E-05 | 0.001149976 | 0.000743144 | 0.001338309 |
| d__Bacteria;k__norank_d__Bacteria;p__Acidobacteriota;c__Acidobacteriae;o__Acidobacteriales;f__Acidobacteriaceae_Subgroup_1;g__unclassified_f__Acidobacteriaceae_Subgroup_1 | 0.00015746 | 0.001051929 | 0.000514971 | 0 | 0 | 0 | 0.000940783 | 0.001140931 | 0.001177645 | 2.40E-05 | 0 | 0 |
| d__Bacteria;k__norank_d__Bacteria;p__Sumerlaeota;c__Sumerlaeia;o__Sumerlaeales;f__Sumerlaeaceae;g__Sumerlaea | 0.000393649 | 0.00056445 | 0.000588538 | 0.000471739 | 0.000565867 | 0.000486317 | 0.000574923 | 0.000285233 | 0.000204808 | 0.000119789 | 0.000272486 | 0.000463261 |
| d__Bacteria;k__norank_d__Bacteria;p__Actinobacteriota;c__Actinobacteria;o__Kineosporiales;f__Kineosporiaceae;g__Quadrisphaera | 0 | 7.70E-05 | 4.90E-05 | 0.002701775 | 6.79E-05 | 0.000198948 | 0.000757853 | 0.000440814 | 0.000332813 | 0.000239578 | 7.43E-05 | 2.57E-05 |
| d__Bacteria;k__norank_d__Bacteria;p__Bacteroidota;c__Bacteroidia;o__Flavobacteriales;f__NS9_marine_group;g__norank_f__NS9_marine_group | 0.000419892 | 0.000102627 | 0.000613061 | 6.43E-05 | 0.000294251 | 0.000110527 | 0.000287461 | 0.000337093 | 0.000844832 | 0.000359368 | 0.000792687 | 0.000720628 |
| d__Bacteria;k__norank_d__Bacteria;p__Actinobacteriota;c__Actinobacteria;o__Micrococcales;f__Micrococcaceae;g__unclassified_f__Micrococcaceae | 0.000262433 | 0.000230911 | 0.000196179 | 0.000192984 | 0.000181077 | 0.000198948 | 0.000235196 | 0.000259302 | 5.12E-05 | 0.000934356 | 0.000743144 | 0.001441256 |
| d__Bacteria;k__norank_d__Bacteria;p__Firmicutes;c__Clostridia;o__Peptostreptococcales-Tissierellales;f__Peptostreptococcaceae;g__Terrisporobacter | 0.000787298 | 0.000897989 | 0.00080924 | 0.000664723 | 0.000475328 | 0.000198948 | 0.00018293 | 0.000337093 | 5.12E-05 | 0.000263536 | 9.91E-05 | 0.00015442 |
| d__Bacteria;k__norank_d__Bacteria;p__Cyanobacteria;c__Cyanobacteriia;o__Cyanobacteriales;f__Nostocaceae;g__Nostoc_PCC-73102 | 0.000104973 | 0.000615764 | 0.000833762 | 0.000150099 | 0.000203712 | 0.000618949 | 0.000757853 | 0.000544535 | 0.000972837 | 2.40E-05 | 0 | 0 |
| d__Bacteria;k__norank_d__Bacteria;p__Bacteroidota;c__Bacteroidia;o__Chitinophagales;f__Chitinophagaceae;g__Parafilimonas | 0.000367406 | 0.000307882 | 0.000490449 | 2.14E-05 | 0.000226347 | 0.000265264 | 0.00109758 | 0.000440814 | 0.000614424 | 0.000407283 | 0.000421115 | 0.000128684 |
| d__Bacteria;k__norank_d__Bacteria;p__Bacteroidota;c__Bacteroidia;o__Chitinophagales;f__Chitinophagaceae;g__Flavihumibacter | 0.000393649 | 0.000128284 | 0.000514971 | 6.43E-05 | 0.000181077 | 0.000574738 | 0.000601056 | 0.000466744 | 0.000281611 | 0.000646862 | 0.000470658 | 0.000437524 |
| d__Bacteria;k__norank_d__Bacteria;p__Planctomycetota;c__Phycisphaerae;o__Phycisphaerales;f__Phycisphaeraceae;g__SM1A02 | 0.000367406 | 0.000359195 | 0.000784718 | 0.000150099 | 0.000271616 | 0.000198948 | 0.000287461 | 0.000207442 | 0.000486419 | 0.000167705 | 0.001114717 | 0.000360314 |
| d__Bacteria;k__norank_d__Bacteria;p__Actinobacteriota;c__Actinobacteria;o__Micrococcales;f__Demequinaceae;g__Demequina | 0 | 0.000205255 | 9.81E-05 | 2.14E-05 | 4.53E-05 | 0.000265264 | 0.001019182 | 0.000959419 | 0.001894473 | 7.19E-05 | 0.000123857 | 5.15E-05 |
| d__Bacteria;k__norank_d__Bacteria;p__Gemmatimonadota;c__S0134_terrestrial_group;o__norank_c__S0134_terrestrial_group;f__norank_o__norank_c__S0134_terrestrial_group;g__norank_f__norank_o__norank_c__S0134_terrestrial_group | 0.000551109 | 0.000333539 | 0.000539493 | 0.000471739 | 0.000384789 | 0.000530527 | 0.000470391 | 0.000414884 | 0.000230409 | 0.000263536 | 0.000322029 | 0.00023163 |
| d__Bacteria;k__norank_d__Bacteria;p__Actinobacteriota;c__Acidimicrobiia;o__Microtrichales;f__Microtrichaceae;g__norank_f__Microtrichaceae | 7.87E-05 | 0.000102627 | 0.000122612 | 0.000128656 | 0.000271616 | 0.000552633 | 0.00073172 | 0.000907559 | 0.001100842 | 0.00033541 | 0.000123857 | 0.000283104 |
| d__Bacteria;k__norank_d__Bacteria;p__Proteobacteria;c__Alphaproteobacteria;o__Rhizobiales;f__Rhizobiaceae;g__Pseudaminobacter | 0.000498622 | 0.000615764 | 0.000416881 | 0.000128656 | 0.000520598 | 0.00033158 | 0.000444259 | 0.000155581 | 0.000204808 | 0.000407283 | 0.000421115 | 0.000591944 |
| d__Bacteria;k__norank_d__Bacteria;p__Proteobacteria;c__Alphaproteobacteria;o__Rhizobiales;f__Beijerinckiaceae;g__Roseiarcus | 0.000524866 | 0.000436166 | 0.000613061 | 4.29E-05 | 0.000135808 | 0.000132632 | 0.000705587 | 0.000959419 | 0.000691227 | 0.000191663 | 0.000148629 | 0.00015442 |
| d__Bacteria;k__norank_d__Bacteria;p__Actinobacteriota;c__Actinobacteria;o__Micrococcales;f__Promicromonosporaceae;g__Promicromonospora | 0.00015746 | 0.000153941 | 0.000220702 | 0.000707608 | 0.00072431 | 0.000994739 | 0.000418126 | 0.000259302 | 0.000896034 | 0 | 2.48E-05 | 0.000128684 |
| d__Bacteria;k__norank_d__Bacteria;p__Chloroflexi;c__Chloroflexia;o__Chloroflexales;f__Herpetosiphonaceae;g__Herpetosiphon | 0.000131216 | 0.000282225 | 0.000514971 | 0.000150099 | 0.000837483 | 0.000994739 | 2.61E-05 | 0 | 0 | 0.000694777 | 0.000644059 | 0.000386051 |
| d__Bacteria;k__norank_d__Bacteria;p__Verrucomicrobiota;c__Chlamydiae;o__Chlamydiales;f__Simkaniaceae;g__norank_f__Simkaniaceae | 0.000419892 | 0.001975575 | 0.00090733 | 0.000235869 | 0.000135808 | 4.42E-05 | 0.000130664 | 0.000103721 | 5.12E-05 | 9.58E-05 | 0.000148629 | 0.000411787 |
| d__Bacteria;k__norank_d__Bacteria;p__Bacteroidota;c__Bacteroidia;o__Cytophagales;f__Cytophagaceae;g__Sporocytophaga | 0.000656082 | 0.000359195 | 0.000465926 | 0.000428853 | 0.000316885 | 0.00037579 | 0.000287461 | 0.000103721 | 0.00025601 | 0.00055103 | 0.00049543 | 0.000360314 |
| d__Bacteria;k__norank_d__Bacteria;p__Proteobacteria;c__Alphaproteobacteria;o__Rhizobiales;f__Rhodomicrobiaceae;g__Rhodomicrobium | 0.000551109 | 0.000436166 | 0.000490449 | 0.000343083 | 0.000452694 | 0.000176842 | 0.000679454 | 0.000829768 | 0.000230409 | 9.58E-05 | 9.91E-05 | 0.000257367 |
| d__Bacteria;k__norank_d__Bacteria;p__Proteobacteria;c__Gammaproteobacteria;o__Burkholderiales;f__unclassified_o__Burkholderiales;g__unclassified_o__Burkholderiales | 0.000971001 | 0.000461823 | 0.000662105 | 0.000107213 | 0.000407424 | 4.42E-05 | 0.000287461 | 0.000285233 | 0.000230409 | 0.000215621 | 0.000544973 | 0.000360314 |
| d__Bacteria;k__norank_d__Bacteria;p__Gemmatimonadota;c__AKAU4049;o__norank_c__AKAU4049;f__norank_o__norank_c__AKAU4049;g__norank_f__norank_o__norank_c__AKAU4049 | 0 | 0.000128284 | 2.45E-05 | 0.000278755 | 0.000271616 | 0.000110527 | 0.000444259 | 2.59E-05 | 7.68E-05 | 0.00088644 | 0.001560603 | 0.000720628 |
| d__Bacteria;k__norank_d__Bacteria;p__Bacteroidota;c__Bacteroidia;o__Chitinophagales;f__Chitinophagaceae;g__Parasegetibacter | 0.000472379 | 0.000256568 | 0.000514971 | 0.000235869 | 0.00072431 | 0.000751581 | 0.000261329 | 0.000207442 | 0.00025601 | 0.000431241 | 0.000322029 | 7.72E-05 |
| d__Bacteria;k__norank_d__Bacteria;p__Acidobacteriota;c__Blastocatellia;o__Blastocatellales;f__Blastocatellaceae;g__Aridibacter | 0.000183703 | 0.000410509 | 0.000392359 | 0.000171541 | 0.000430059 | 0.000530527 | 0.000418126 | 0.000181512 | 0.000588823 | 0.000239578 | 0.000544973 | 0.000411787 |
| d__Bacteria;k__norank_d__Bacteria;p__Bacteroidota;c__Bacteroidia;o__Sphingobacteriales;f__Sphingobacteriaceae;g__Solitalea | 0.000472379 | 0.000410509 | 0.000465926 | 0.000278755 | 0.000384789 | 0.000420001 | 0.000156797 | 0.000129651 | 0.00051202 | 0.000455199 | 0.000619287 | 0.000180157 |
| d__Bacteria;k__norank_d__Bacteria;p__Proteobacteria;c__Alphaproteobacteria;o__Reyranellales;f__Reyranellaceae;g__norank_f__Reyranellaceae | 0.000262433 | 0.000102627 | 0.000465926 | 0.000128656 | 0.000656406 | 0.00033158 | 0.00036586 | 0.000259302 | 0.000384015 | 0.000527072 | 0.000693602 | 0.000283104 |
| d__Bacteria;k__norank_d__Bacteria;p__Proteobacteria;c__Gammaproteobacteria;o__Coxiellales;f__Coxiellaceae;g__Coxiella | 0.000918515 | 0.00056445 | 0.000858285 | 0.000214427 | 0.000135808 | 0.000243158 | 0.000130664 | 0.000363023 | 5.12E-05 | 0.000359368 | 0.000247715 | 0.000334577 |
| d__Bacteria;k__norank_d__Bacteria;p__Bacteroidota;c__Bacteroidia;o__Flavobacteriales;f__Weeksellaceae;g__Chryseobacterium | 0.000183703 | 0.000179598 | 9.81E-05 | 4.29E-05 | 0.000497963 | 0.000265264 | 0.00073172 | 0.000285233 | 0.00051202 | 0.000742693 | 0.000743144 | 0.000128684 |
| d__Bacteria;k__norank_d__Bacteria;p__Planctomycetota;c__Planctomycetes;o__Isosphaerales;f__Isosphaeraceae;g__Singulisphaera | 0.000183703 | 0.000410509 | 0.000613061 | 0.000278755 | 0.00033952 | 0.000176842 | 0.001071447 | 0.000181512 | 0.000588823 | 2.40E-05 | 7.43E-05 | 0.000463261 |
| d__Bacteria;k__norank_d__Bacteria;p__GAL15;c__norank_p__GAL15;o__norank_c__norank_p__GAL15;f__norank_o__norank_c__norank_p__GAL15;g__norank_f__norank_o__norank_c__norank_p__GAL15 | 0.000314919 | 5.13E-05 | 4.90E-05 | 0.000235869 | 0.000384789 | 0.000309474 | 0.000496524 | 0.000103721 | 2.56E-05 | 0.000814566 | 0.001114717 | 0.000488998 |
| d__Bacteria;k__norank_d__Bacteria;p__Actinobacteriota;c__Actinobacteria;o__Micrococcales;f__Microbacteriaceae;g__Clavibacter | 0.000131216 | 0.000384852 | 0.000196179 | 8.58E-05 | 0.000181077 | 0.000353685 | 0.00036586 | 0.000181512 | 0.000230409 | 0.000407283 | 0.001040402 | 0.000823575 |
| d__Bacteria;k__norank_d__Bacteria;p__Actinobacteriota;c__Actinobacteria;o__Micrococcales;f__Microbacteriaceae;g__unclassified_f__Microbacteriaceae | 0.000209946 | 5.13E-05 | 0.000171657 | 0.000278755 | 0.000407424 | 0.000353685 | 0.000783986 | 0.000388954 | 0.00051202 | 0.000287494 | 0.000421115 | 0.000488998 |
| d__Bacteria;k__norank_d__Bacteria;p__Chloroflexi;c__Ktedonobacteria;o__Ktedonobacterales;f__Ktedonobacteraceae;g__norank_f__Ktedonobacteraceae | 0.000262433 | 0.001385468 | 0.00071115 | 0.00032164 | 0.000113173 | 8.84E-05 | 0.001045314 | 0.000129651 | 0.000179207 | 0 | 0 | 0.000102947 |
| d__Bacteria;k__norank_d__Bacteria;p__Proteobacteria;c__Gammaproteobacteria;o__JG36-GS-52;f__norank_o__JG36-GS-52;g__norank_f__norank_o__JG36-GS-52 | 0.000734812 | 0.000692734 | 0.000637583 | 0.000192984 | 0.000475328 | 6.63E-05 | 2.61E-05 | 0.000155581 | 0.000153606 | 0.00033541 | 0.000445887 | 0.000386051 |
| d__Bacteria;k__norank_d__Bacteria;p__Actinobacteriota;c__Thermoleophilia;o__Gaiellales;f__unclassified_o__Gaiellales;g__unclassified_o__Gaiellales | 0.000183703 | 0.000179598 | 0.000147135 | 0.000128656 | 0.000203712 | 6.63E-05 | 0.000653321 | 0.000777907 | 0.000358414 | 0.000670819 | 0.000619287 | 0.000257367 |
| d__Bacteria;k__norank_d__Bacteria;p__Proteobacteria;c__Gammaproteobacteria;o__Burkholderiales;f__Methylophilaceae;g__Methylotenera | 0.000419892 | 0.000513136 | 0.00071115 | 0.000107213 | 0.000135808 | 0.000287369 | 0.000444259 | 0.000129651 | 2.56E-05 | 0.000263536 | 0.000421115 | 0.000772101 |
| d__Bacteria;k__norank_d__Bacteria;p__Proteobacteria;c__Gammaproteobacteria;o__Burkholderiales;f__Comamonadaceae;g__Comamonas | 0.000708568 | 0.000718391 | 0.000980897 | 2.14E-05 | 4.53E-05 | 0.000132632 | 0.00018293 | 0.000363023 | 0.000435217 | 0.000287494 | 0.0001734 | 0.000180157 |
| d__Bacteria;k__norank_d__Bacteria;p__Acidobacteriota;c__Acidobacteriae;o__Acidobacteriales;f__unclassified_o__Acidobacteriales;g__unclassified_o__Acidobacteriales | 0.000393649 | 0.000974959 | 0.000367836 | 0.000214427 | 6.79E-05 | 2.21E-05 | 0.000574923 | 0.000414884 | 0.00104964 | 0 | 4.95E-05 | 7.72E-05 |
| d__Bacteria;k__norank_d__Bacteria;p__Firmicutes;c__Bacilli;o__Thermoactinomycetales;f__Thermoactinomycetaceae;g__Thermoflavimicrobium | 0.000971001 | 0.000333539 | 0.000662105 | 0.000128656 | 0.000271616 | 0.000221053 | 0.000391993 | 0.000181512 | 7.68E-05 | 0.000383325 | 0.000322029 | 0.000257367 |
| d__Bacteria;k__norank_d__Bacteria;p__WS2;c__norank_p__WS2;o__norank_c__norank_p__WS2;f__norank_o__norank_c__norank_p__WS2;g__norank_f__norank_o__norank_c__norank_p__WS2 | 0.00015746 | 0.000359195 | 0.000171657 | 0.000385968 | 0.000588502 | 0.000552633 | 0.000104531 | 0.000233372 | 0.000460818 | 0.000167705 | 0.000371572 | 0.000643418 |
| d__Bacteria;k__norank_d__Bacteria;p__Nitrospirota;c__Thermodesulfovibrionia;o__norank_c__Thermodesulfovibrionia;f__norank_o__norank_c__Thermodesulfovibrionia;g__norank_f__norank_o__norank_c__Thermodesulfovibrionia | 0.001338407 | 0.000384852 | 0.000931852 | 0.000493181 | 0.000543232 | 8.84E-05 | 7.84E-05 | 5.19E-05 | 0.000204808 | 4.79E-05 | 0 | 2.57E-05 |
| d__Bacteria;k__norank_d__Bacteria;p__Planctomycetota;c__Phycisphaerae;o__Phycisphaerales;f__Phycisphaeraceae;g__AKYG587 | 0.000236189 | 0.000513136 | 0.000539493 | 0.000171541 | 0.000158443 | 0.00037579 | 7.84E-05 | 2.59E-05 | 2.56E-05 | 0.000287494 | 0.000792687 | 0.000952258 |
| d__Bacteria;k__norank_d__Bacteria;p__Firmicutes;c__Clostridia;o__Clostridiales;f__Clostridiaceae;g__Clostridium_sensu_stricto_3 | 0.000656082 | 0.000615764 | 0.001078987 | 0.000235869 | 0.000294251 | 0.000287369 | 7.84E-05 | 0.000648256 | 5.12E-05 | 4.79E-05 | 0.000123857 | 0 |
| d__Bacteria;k__norank_d__Bacteria;p__Bacteroidota;c__Bacteroidia;o__Chitinophagales;f__Chitinophagaceae;g__Flavitalea | 0.000419892 | 7.70E-05 | 0.000171657 | 0.000450296 | 0.000362155 | 0.000950528 | 0.00036586 | 7.78E-05 | 0.000665626 | 7.19E-05 | 0.000222943 | 0.000205894 |
| d__Bacteria;k__norank_d__Bacteria;p__Actinobacteriota;c__Actinobacteria;o__Micromonosporales;f__Micromonosporaceae;g__Actinoplanes | 5.25E-05 | 0.000102627 | 0.000122612 | 4.29E-05 | 0.000135808 | 0.00033158 | 0.000418126 | 0.000388954 | 0.000691227 | 0.000838524 | 0.000520201 | 0.000360314 |
| d__Bacteria;k__norank_d__Bacteria;p__Bdellovibrionota;c__Oligoflexia;o__Oligoflexales;f__norank_o__Oligoflexales;g__norank_f__norank_o__Oligoflexales | 0.000393649 | 0.000128284 | 0.000269747 | 0.000385968 | 0.000384789 | 0.000486317 | 0.000235196 | 0.000207442 | 0.000307212 | 0.000191663 | 0.000470658 | 0.000540471 |
| d__Bacteria;k__norank_d__Bacteria;p__Myxococcota;c__Myxococcia;o__Myxococcales;f__Myxococcaceae;g__unclassified_f__Myxococcaceae | 5.25E-05 | 0.000282225 | 0.000416881 | 0.000343083 | 0.000294251 | 0.000420001 | 0.000130664 | 0.000259302 | 0.000204808 | 0.00033541 | 0.000743144 | 0.000514734 |
| d__Bacteria;k__norank_d__Bacteria;p__Firmicutes;c__Clostridia;o__Clostridiales;f__Caloramatoraceae;g__Fonticella | 0.000787298 | 0.000487479 | 0.000490449 | 0.000407411 | 0.000226347 | 0.000154737 | 0.000313594 | 0.000570465 | 0.00025601 | 9.58E-05 | 9.91E-05 | 0.000102947 |
| d__Bacteria;k__norank_d__Bacteria;p__Elusimicrobiota;c__Lineage_IIa;o__norank_c__Lineage_IIa;f__norank_o__norank_c__Lineage_IIa;g__norank_f__norank_o__norank_c__Lineage_IIa | 0.000183703 | 0.000897989 | 0.000564016 | 0.000192984 | 0.000113173 | 2.21E-05 | 0.000522657 | 0.000311163 | 0.000486419 | 0.000167705 | 0.000198172 | 0.000308841 |
| d__Bacteria;k__norank_d__Bacteria;p__Bacteroidota;c__Kapabacteria;o__Kapabacteriales;f__norank_o__Kapabacteriales;g__norank_f__norank_o__Kapabacteriales | 0.000262433 | 0.000128284 | 0.000245224 | 0.000879149 | 0.00067904 | 0.000552633 | 0.000156797 | 5.19E-05 | 0.00025601 | 0.000119789 | 0.000445887 | 0.000180157 |
| d__Bacteria;k__norank_d__Bacteria;p__Firmicutes;c__Bacilli;o__Bacillales;f__Planococcaceae;g__Psychrobacillus | 0.000866028 | 0.000410509 | 0.000514971 | 0.000364525 | 0.000248981 | 0.000243158 | 7.84E-05 | 0.000337093 | 2.56E-05 | 0.00033541 | 0.000322029 | 0.000205894 |
| d__Bacteria;k__norank_d__Bacteria;p__Bacteroidota;c__Bacteroidia;o__Sphingobacteriales;f__Sphingobacteriaceae;g__Mucilaginibacter | 0.000236189 | 0.000205255 | 0.000564016 | 8.58E-05 | 0.000158443 | 8.84E-05 | 0.001045314 | 0.000492675 | 0.00079363 | 7.19E-05 | 9.91E-05 | 0.000102947 |
| d__Bacteria;k__norank_d__Bacteria;p__Planctomycetota;c__Phycisphaerae;o__Tepidisphaerales;f__WD2101_soil_group;g__norank_f__WD2101_soil_group | 0.000761055 | 0.000974959 | 0.001839182 | 2.14E-05 | 0 | 2.21E-05 | 5.23E-05 | 0 | 7.68E-05 | 0 | 9.91E-05 | 0 |
| d__Bacteria;k__norank_d__Bacteria;p__Chloroflexi;c__Chloroflexia;o__Thermomicrobiales;f__Thermomicrobiaceae;g__Nitrolancea | 0.000367406 | 0.000205255 | 7.36E-05 | 0.000278755 | 0.000113173 | 0.000132632 | 0.000209063 | 0.000751977 | 0.000691227 | 0.00055103 | 0.000148629 | 0.000180157 |
| d__Bacteria;k__norank_d__Bacteria;p__Myxococcota;c__Polyangia;o__Blfdi19;f__norank_o__Blfdi19;g__norank_f__norank_o__Blfdi19 | 0.000419892 | 0.000333539 | 0.000564016 | 0.000385968 | 0.000294251 | 0.00033158 | 0.000104531 | 0.000155581 | 5.12E-05 | 0.000503115 | 0.000222943 | 0.000283104 |
| d__Bacteria;k__norank_d__Bacteria;p__Chloroflexi;c__Anaerolineae;o__Caldilineales;f__Caldilineaceae;g__Litorilinea | 0.000209946 | 0.000128284 | 9.81E-05 | 0.000364525 | 0.000565867 | 0.000530527 | 0.000601056 | 0.000129651 | 7.68E-05 | 0.000263536 | 0.000346801 | 0.000334577 |
| d__Bacteria;k__norank_d__Bacteria;p__Actinobacteriota;c__Actinobacteria;o__Frankiales;f__Geodermatophilaceae;g__Geodermatophilus | 5.25E-05 | 0.000128284 | 2.45E-05 | 0.00096492 | 0.000271616 | 0.000552633 | 0.000313594 | 0.000337093 | 0.000332813 | 0.000311452 | 0.000198172 | 0.00015442 |
| d__Bacteria;k__norank_d__Bacteria;p__Desulfobacterota;c__Desulfuromonadia;o__norank_c__Desulfuromonadia;f__Desulfuromonadaceae;g__Desulfuromonas | 0.000236189 | 0.000128284 | 0.000416881 | 6.43E-05 | 0.002263468 | 0.000287369 | 0 | 0 | 0 | 0 | 0.000198172 | 2.57E-05 |
| d__Bacteria;k__norank_d__Bacteria;p__Actinobacteriota;c__Actinobacteria;o__PeM15;f__norank_o__PeM15;g__norank_f__norank_o__PeM15 | 2.62E-05 | 0.000179598 | 9.81E-05 | 8.58E-05 | 0.000248981 | 0.000353685 | 0.000601056 | 0.000700117 | 0.000844832 | 0 | 0.000198172 | 0.000205894 |
| d__Bacteria;k__norank_d__Bacteria;p__Proteobacteria;c__Gammaproteobacteria;o__Xanthomonadales;f__Xanthomonadaceae;g__Stenotrophomonas | 0.000708568 | 0.000128284 | 9.81E-05 | 0.000171541 | 0.000656406 | 0.000243158 | 0.000993049 | 0.000181512 | 0.000128005 | 0 | 0.000148629 | 7.72E-05 |
| d__Bacteria;k__norank_d__Bacteria;p__Deinococcota;c__Deinococci;o__Deinococcales;f__Trueperaceae;g__Truepera | 7.87E-05 | 7.70E-05 | 0 | 0.000879149 | 0.000656406 | 0.001304213 | 0.000156797 | 0.000103721 | 0.000102404 | 4.79E-05 | 2.48E-05 | 0.000102947 |
| d__Bacteria;k__norank_d__Bacteria;p__Firmicutes;c__Bacilli;o__Bacillales;f__Bacillaceae;g__unclassified_f__Bacillaceae | 0.000367406 | 0.000667077 | 0.000490449 | 0.000407411 | 0.000135808 | 0.00033158 | 7.84E-05 | 0.000233372 | 0.00025601 | 0.000383325 | 2.48E-05 | 0.00015442 |
| d__Bacteria;k__norank_d__Bacteria;p__Firmicutes;c__Clostridia;o__Lachnospirales;f__Lachnospiraceae;g__Anaerosporobacter | 0.000708568 | 0.000538793 | 0.000465926 | 0.000235869 | 0.000452694 | 0.000176842 | 0.00018293 | 0.000311163 | 0.00025601 | 9.58E-05 | 9.91E-05 | 0 |
| d__Bacteria;k__norank_d__Bacteria;p__Bacteroidota;c__Bacteroidia;o__Chitinophagales;f__norank_o__Chitinophagales;g__norank_f__norank_o__Chitinophagales | 0.000314919 | 0.000384852 | 0.000245224 | 0.00032164 | 0.000611136 | 0.000353685 | 5.23E-05 | 0.000285233 | 0.000153606 | 0.000311452 | 0.000148629 | 0.000308841 |
| d__Bacteria;k__norank_d__Bacteria;p__Proteobacteria;c__Alphaproteobacteria;o__Acetobacterales;f__Acetobacteraceae;g__unclassified_f__Acetobacteraceae | 0.000393649 | 0.000256568 | 0.000465926 | 0.000107213 | 4.53E-05 | 6.63E-05 | 0.000783986 | 0.000492675 | 0.000384015 | 0.000191663 | 7.43E-05 | 0.000205894 |
| d__Bacteria;k__norank_d__Bacteria;p__Firmicutes;c__Negativicutes;o__Veillonellales-Selenomonadales;f__Sporomusaceae;g__Sporomusa | 7.87E-05 | 0.000230911 | 2.45E-05 | 0.000514624 | 0.000113173 | 0.000309474 | 0.000313594 | 0.000388954 | 0.000460818 | 0.000215621 | 0.000247715 | 0.000514734 |
| d__Bacteria;k__norank_d__Bacteria;p__Firmicutes;c__Clostridia;o__Thermincolales;f__Thermincolaceae;g__Thermincola | 0.000971001 | 0.000333539 | 0.000490449 | 0.000192984 | 0.000135808 | 0.00033158 | 0.000235196 | 0.000492675 | 0.000102404 | 2.40E-05 | 7.43E-05 | 2.57E-05 |
| d__Bacteria;k__norank_d__Bacteria;p__Acidobacteriota;c__Acidobacteriae;o__Subgroup_15;f__norank_o__Subgroup_15;g__norank_f__norank_o__Subgroup_15 | 0.000524866 | 0.000333539 | 0.000686628 | 0.000300197 | 0.000113173 | 0.000154737 | 0.000235196 | 0.000285233 | 0.000204808 | 0.000191663 | 0.000272486 | 7.72E-05 |
| d__Bacteria;k__norank_d__Bacteria;p__Firmicutes;c__Clostridia;o__Clostridiales;f__Clostridiaceae;g__Clostridium_sensu_stricto_10 | 0.000472379 | 0.000410509 | 0.000416881 | 0.000300197 | 6.79E-05 | 0.000154737 | 0.000287461 | 0.000622326 | 0.000204808 | 0.000143747 | 0.000198172 | 5.15E-05 |
| d__Bacteria;k__norank_d__Bacteria;p__Patescibacteria;c__WWE3;o__norank_c__WWE3;f__norank_o__norank_c__WWE3;g__norank_f__norank_o__norank_c__WWE3 | 0.000472379 | 0.000461823 | 0.000637583 | 8.58E-05 | 2.26E-05 | 0 | 0.00018293 | 0.000492675 | 0.000691227 | 0.000215621 | 0 | 5.15E-05 |
| d__Bacteria;k__norank_d__Bacteria;p__Firmicutes;c__Bacilli;o__Bacillales;f__Planococcaceae;g__unclassified_f__Planococcaceae | 0.000551109 | 0.000333539 | 0.000465926 | 0.000278755 | 0.000158443 | 0.000176842 | 0.000339727 | 0.000466744 | 2.56E-05 | 0.000239578 | 4.95E-05 | 0.000205894 |
| d__Bacteria;k__norank_d__Bacteria;p__Planctomycetota;c__Planctomycetes;o__Isosphaerales;f__Isosphaeraceae;g__norank_f__Isosphaeraceae | 0.000131216 | 0.000769704 | 0.000367836 | 0.000214427 | 0.000362155 | 0.000110527 | 0.000418126 | 5.19E-05 | 0.000179207 | 0.000191663 | 0.0001734 | 0.000257367 |
| d__Bacteria;k__norank_d__Bacteria;p__Actinobacteriota;c__Actinobacteria;o__Streptosporangiales;f__Streptosporangiaceae;g__Nonomuraea | 0.000236189 | 0.000179598 | 0.000122612 | 0.000171541 | 0.000158443 | 0.000198948 | 0.000261329 | 0.000337093 | 0.000435217 | 0.000239578 | 0.000693602 | 0.000180157 |
| d__Bacteria;k__norank_d__Bacteria;p__Chloroflexi;c__Chloroflexia;o__Chloroflexales;f__Chloroflexaceae;g__FFCH7168 | 7.87E-05 | 0.000359195 | 0.000367836 | 8.58E-05 | 0.000271616 | 0.000397896 | 0.000156797 | 0.000259302 | 0.000153606 | 0.000287494 | 0.000371572 | 0.000411787 |
| d__Bacteria;k__norank_d__Bacteria;p__Proteobacteria;c__Gammaproteobacteria;o__Cellvibrionales;f__Cellvibrionaceae;g__Cellvibrio | 0.000236189 | 5.13E-05 | 7.36E-05 | 2.14E-05 | 0.002037121 | 0.000353685 | 5.23E-05 | 0 | 0.000102404 | 0.000143747 | 2.48E-05 | 0.000102947 |
| d__Bacteria;k__norank_d__Bacteria;p__Actinobacteriota;c__Actinobacteria;o__Streptosporangiales;f__Thermomonosporaceae;g__Thermobispora | 0 | 0 | 0 | 0 | 0 | 0 | 0.001071447 | 0.000985349 | 0.001126443 | 0 | 0 | 0 |
| d__Bacteria;k__norank_d__Bacteria;p__Bacteroidota;c__Bacteroidia;o__Chitinophagales;f__Chitinophagaceae;g__Chitinophaga | 0.001653326 | 0.000256568 | 0.000367836 | 0.000150099 | 0.000113173 | 4.42E-05 | 0.000235196 | 5.19E-05 | 0.000102404 | 2.40E-05 | 0.000148629 | 0 |
| d__Bacteria;k__norank_d__Bacteria;p__Cyanobacteria;c__Cyanobacteriia;o__Cyanobacteriales;f__Nostocaceae;g__Scytonema_UTEX_2349 | 0.00015746 | 0.000359195 | 0.000147135 | 0.000343083 | 0.000316885 | 0.000486317 | 0.000156797 | 0.000155581 | 0.00079363 | 2.40E-05 | 0.000123857 | 2.57E-05 |
| d__Bacteria;k__norank_d__Bacteria;p__Methylomirabilota;c__Methylomirabilia;o__Methylomirabilales;f__Methylomirabilaceae;g__Sh765B-TzT-35 | 0.000262433 | 0.000205255 | 0.000367836 | 0.000385968 | 0.000565867 | 0.000464212 | 0.000156797 | 5.19E-05 | 2.56E-05 | 0.000191663 | 0.000198172 | 0.000205894 |
| d__Bacteria;k__norank_d__Bacteria;p__Firmicutes;c__Bacilli;o__Paenibacillales;f__Paenibacillaceae;g__norank_f__Paenibacillaceae | 0.000524866 | 0.000538793 | 0.000465926 | 0.000214427 | 6.79E-05 | 6.63E-05 | 0.000287461 | 0.000233372 | 0.000230409 | 0.000191663 | 0.000123857 | 0.000128684 |
| d__Bacteria;k__norank_d__Bacteria;p__Chloroflexi;c__Anaerolineae;o__norank_c__Anaerolineae;f__norank_o__norank_c__Anaerolineae;g__norank_f__norank_o__norank_c__Anaerolineae | 0.000892271 | 0.000590107 | 0.000416881 | 4.29E-05 | 4.53E-05 | 6.63E-05 | 5.23E-05 | 0.000466744 | 0.000179207 | 4.79E-05 | 0.0001734 | 7.72E-05 |
| d__Bacteria;k__norank_d__Bacteria;p__Actinobacteriota;c__Actinobacteria;o__Corynebacteriales;f__Nocardiaceae;g__Smaragdicoccus | 0.000131216 | 0.000384852 | 0.000318792 | 6.43E-05 | 9.05E-05 | 0.000221053 | 0.000496524 | 0.000492675 | 0.000179207 | 0.000287494 | 0.000148629 | 0.000205894 |
| d__Bacteria;k__norank_d__Bacteria;p__Verrucomicrobiota;c__Verrucomicrobiae;o__Verrucomicrobiales;f__Verrucomicrobiaceae;g__Roseimicrobium | 0.000183703 | 0.00064142 | 0.000662105 | 4.29E-05 | 0.000135808 | 0.00033158 | 0.000156797 | 2.59E-05 | 7.68E-05 | 9.58E-05 | 0.000371572 | 0.000283104 |
| d__Bacteria;k__norank_d__Bacteria;p__SAR324_cladeMarine_group_B;c__norank_p__SAR324_cladeMarine_group_B;o__norank_c__norank_p__SAR324_cladeMarine_group_B;f__norank_o__norank_c__norank_p__SAR324_cladeMarine_group_B;g__norank_f__norank_o__norank_c__norank_p__SAR324_cladeMarine_group_B | 0.000446136 | 0.000282225 | 0.000441404 | 6.43E-05 | 0.000158443 | 0.000287369 | 0.00018293 | 0.000181512 | 0.000179207 | 0.00033541 | 0.000222943 | 0.000205894 |
| d__Bacteria;k__norank_d__Bacteria;p__Planctomycetota;c__Planctomycetes;o__Isosphaerales;f__Isosphaeraceae;g__Aquisphaera | 0.000183703 | 0.000461823 | 0.000269747 | 0.000128656 | 0.000226347 | 0.000198948 | 0.000679454 | 0.000259302 | 0.000281611 | 0.000119789 | 7.43E-05 | 7.72E-05 |
| d__Bacteria;k__norank_d__Bacteria;p__Acidobacteriota;c__unclassified_p__Acidobacteriota;o__unclassified_p__Acidobacteriota;f__unclassified_p__Acidobacteriota;g__unclassified_p__Acidobacteriota | 0.000551109 | 0.000359195 | 0 | 6.43E-05 | 4.53E-05 | 0 | 0.000261329 | 0.000181512 | 0.000307212 | 0.000119789 | 0.000297258 | 0.000772101 |
| d__Bacteria;k__norank_d__Bacteria;p__Entotheonellaeota;c__Entotheonellia;o__Entotheonellales;f__Entotheonellaceae;g__norank_f__Entotheonellaceae | 0.00015746 | 0.000307882 | 0.000245224 | 2.14E-05 | 0.000226347 | 0.000110527 | 0.00018293 | 0 | 2.56E-05 | 0.000694777 | 0.000569744 | 0.000411787 |
| d__Bacteria;k__norank_d__Bacteria;p__Proteobacteria;c__Gammaproteobacteria;o__Burkholderiales;f__Oxalobacteraceae;g__Noviherbaspirillum | 0.000288676 | 0.000333539 | 0.000318792 | 0.000364525 | 0.000543232 | 0.000198948 | 0.000130664 | 0.000259302 | 0.000102404 | 0.000143747 | 9.91E-05 | 0.00015442 |
| d__Bacteria;k__norank_d__Bacteria;p__Actinobacteriota;c__Thermoleophilia;o__unclassified_c__Thermoleophilia;f__unclassified_c__Thermoleophilia;g__unclassified_c__Thermoleophilia | 0.000682325 | 0.000102627 | 0 | 0.000814821 | 0.000316885 | 0.000309474 | 0.000339727 | 7.78E-05 | 2.56E-05 | 0.000191663 | 2.48E-05 | 2.57E-05 |
| d__Bacteria;k__norank_d__Bacteria;p__Firmicutes;c__Bacilli;o__Bacillales;f__Planococcaceae;g__Solibacillus | 0.000551109 | 0.00064142 | 0.000514971 | 0.000150099 | 0.000113173 | 8.84E-05 | 0.000209063 | 0.000103721 | 2.56E-05 | 0.000239578 | 4.95E-05 | 0.000205894 |
| d__Bacteria;k__norank_d__Bacteria;p__Proteobacteria;c__Gammaproteobacteria;o__Burkholderiales;f__Nitrosomonadaceae;g__GOUTA6 | 0.000603595 | 0.000436166 | 0.001299689 | 0.000107213 | 4.53E-05 | 8.84E-05 | 2.61E-05 | 5.19E-05 | 0.000102404 | 7.19E-05 | 2.48E-05 | 2.57E-05 |
| d__Bacteria;k__norank_d__Bacteria;p__Actinobacteriota;c__Actinobacteria;o__Streptosporangiales;f__Thermomonosporaceae;g__Actinocorallia | 0.000262433 | 0.000128284 | 0.000367836 | 0.000493181 | 0.000135808 | 0.00033158 | 5.23E-05 | 0.000337093 | 5.12E-05 | 0.000167705 | 0.000322029 | 0.00023163 |
| d__Bacteria;k__norank_d__Bacteria;p__Acidobacteriota;c__Acidobacteriae;o__unclassified_c__Acidobacteriae;f__unclassified_c__Acidobacteriae;g__unclassified_c__Acidobacteriae | 0.000839785 | 0.000256568 | 0.000269747 | 0.000171541 | 0.000181077 | 8.84E-05 | 0.000156797 | 0.000155581 | 0.000153606 | 0.000167705 | 0.000346801 | 7.72E-05 |
| d__Bacteria;k__norank_d__Bacteria;p__Armatimonadota;c__Chthonomonadetes;o__Chthonomonadales;f__norank_o__Chthonomonadales;g__norank_f__norank_o__Chthonomonadales | 0.000209946 | 0.000179598 | 0.000171657 | 0.000621837 | 0.00033952 | 0.000198948 | 0.000444259 | 0.000103721 | 5.12E-05 | 4.79E-05 | 0.000247715 | 0.00023163 |
| d__Bacteria;k__norank_d__Bacteria;p__Actinobacteriota;c__Acidimicrobiia;o__Actinomarinales;f__norank_o__Actinomarinales;g__norank_f__norank_o__Actinomarinales | 0.000104973 | 7.70E-05 | 9.81E-05 | 8.58E-05 | 0.000475328 | 0.000176842 | 0.000261329 | 0.000129651 | 5.12E-05 | 0.000455199 | 0.000743144 | 0.000180157 |
| d__Bacteria;k__norank_d__Bacteria;p__Verrucomicrobiota;c__Chlamydiae;o__Chlamydiales;f__unclassified_o__Chlamydiales;g__unclassified_o__Chlamydiales | 2.62E-05 | 0.001180213 | 0.000662105 | 0.000150099 | 0.000135808 | 0.000110527 | 5.23E-05 | 0.000103721 | 0 | 0.000167705 | 0.000123857 | 7.72E-05 |
| d__Bacteria;k__norank_d__Bacteria;p__Armatimonadota;c__Fimbriimonadia;o__Fimbriimonadales;f__Fimbriimonadaceae;g__norank_f__Fimbriimonadaceae | 0.000262433 | 0.000513136 | 0.001397778 | 0 | 6.79E-05 | 2.21E-05 | 2.61E-05 | 5.19E-05 | 2.56E-05 | 0.000167705 | 0.0001734 | 7.72E-05 |
| d__Bacteria;k__norank_d__Bacteria;p__Myxococcota;c__Polyangia;o__mle1-27;f__norank_o__mle1-27;g__norank_f__norank_o__mle1-27 | 0.000419892 | 0.000333539 | 0.000171657 | 0.000192984 | 0.000294251 | 0.000154737 | 0.000104531 | 0.000440814 | 0.000102404 | 0.000287494 | 0.0001734 | 0.000102947 |
| d__Bacteria;k__norank_d__Bacteria;p__Firmicutes;c__Clostridia;o__norank_c__Clostridia;f__Hungateiclostridiaceae;g__unclassified_f__Hungateiclostridiaceae | 0.000314919 | 0.000513136 | 0.000196179 | 0.000385968 | 0.000113173 | 0.000243158 | 0.000156797 | 0.000388954 | 0 | 0.000263536 | 9.91E-05 | 0.000102947 |
| d__Bacteria;k__norank_d__Bacteria;p__Actinobacteriota;c__Actinobacteria;o__Micrococcales;f__Micrococcaceae;g__Pseudarthrobacter | 0 | 0.000282225 | 0.000196179 | 0 | 0.000181077 | 0.000243158 | 0.000156797 | 0.000337093 | 5.12E-05 | 0.000407283 | 0.000247715 | 0.000669155 |
| d__Bacteria;k__norank_d__Bacteria;p__Gemmatimonadota;c__Longimicrobia;o__Longimicrobiales;f__Longimicrobiaceae;g__norank_f__Longimicrobiaceae | 7.87E-05 | 7.70E-05 | 0 | 0.000214427 | 0.000430059 | 0.000596843 | 0.000209063 | 5.19E-05 | 0.000588823 | 0.000119789 | 0.000247715 | 0.00015442 |
| d__Bacteria;k__norank_d__Bacteria;p__Actinobacteriota;c__Actinobacteria;o__Micrococcales;f__unclassified_o__Micrococcales;g__unclassified_o__Micrococcales | 2.62E-05 | 0.000128284 | 0 | 0.000235869 | 0.000158443 | 0.000464212 | 0.000966916 | 0.000259302 | 0.000128005 | 0.000191663 | 9.91E-05 | 0.000102947 |
| d__Bacteria;k__norank_d__Bacteria;p__Actinobacteriota;c__Actinobacteria;o__unclassified_c__Actinobacteria;f__unclassified_c__Actinobacteria;g__unclassified_c__Actinobacteria | 0.000104973 | 0.000256568 | 0.000122612 | 0.000235869 | 0.000113173 | 0.000287369 | 0.000627189 | 0.000285233 | 0.00025601 | 0.000167705 | 0.0001734 | 0.000128684 |
| d__Bacteria;k__norank_d__Bacteria;p__Planctomycetota;c__Planctomycetes;o__Isosphaerales;f__Isosphaeraceae;g__unclassified_f__Isosphaeraceae | 2.62E-05 | 0.000538793 | 0.000245224 | 8.58E-05 | 0.000135808 | 8.84E-05 | 0.000836252 | 7.78E-05 | 0.000358414 | 2.40E-05 | 9.91E-05 | 0.000205894 |
| d__Bacteria;k__norank_d__Bacteria;p__Actinobacteriota;c__Actinobacteria;o__Frankiales;f__Sporichthyaceae;g__Sporichthya | 0.000367406 | 0.000333539 | 0.000220702 | 6.43E-05 | 0.000362155 | 0.00033158 | 0.000235196 | 0.000129651 | 7.68E-05 | 0.000119789 | 0.000222943 | 0.000257367 |
| d__Bacteria;k__norank_d__Bacteria;p__Verrucomicrobiota;c__Verrucomicrobiae;o__Pedosphaerales;f__Pedosphaeraceae;g__ADurb.Bin063-1 | 0.000498622 | 0.000590107 | 0.001324211 | 0 | 2.26E-05 | 0 | 5.23E-05 | 5.19E-05 | 2.56E-05 | 0.000119789 | 2.48E-05 | 0 |
| d__Bacteria;k__norank_d__Bacteria;p__Proteobacteria;c__Alphaproteobacteria;o__Rhizobiales;f__Beijerinckiaceae;g__Rhodoblastus | 0.000393649 | 0.000410509 | 0.000171657 | 0 | 0 | 2.21E-05 | 0.000601056 | 0.000674186 | 0.000384015 | 0 | 4.95E-05 | 0 |
| d__Bacteria;k__norank_d__Bacteria;p__Proteobacteria;c__Alphaproteobacteria;o__Rhodobacterales;f__Rhodobacteraceae;g__unclassified_f__Rhodobacteraceae | 0.000209946 | 0.000205255 | 0.000416881 | 0.000235869 | 0.000384789 | 0.000486317 | 7.84E-05 | 7.78E-05 | 2.56E-05 | 0.000239578 | 0.000148629 | 0.000180157 |
| d__Bacteria;k__norank_d__Bacteria;p__Proteobacteria;c__Gammaproteobacteria;o__Burkholderiales;f__Comamonadaceae;g__Variovorax | 0.000367406 | 0.000359195 | 0.000490449 | 2.14E-05 | 0.000113173 | 6.63E-05 | 0.000156797 | 0.000129651 | 0.000332813 | 0.000167705 | 0.000247715 | 0.00023163 |
| d__Bacteria;k__norank_d__Bacteria;p__Acidobacteriota;c__Acidobacteriae;o__AKIW659;f__norank_o__AKIW659;g__norank_f__norank_o__AKIW659 | 0.000787298 | 0.000230911 | 0.000269747 | 0.000150099 | 0.000316885 | 0.000309474 | 7.84E-05 | 0.000259302 | 0.000102404 | 9.58E-05 | 4.95E-05 | 2.57E-05 |
| d__Bacteria;k__norank_d__Bacteria;p__Firmicutes;c__Bacilli;o__Paenibacillales;f__Paenibacillaceae;g__Ammoniphilus | 0.000262433 | 0.000307882 | 0.000220702 | 0.000278755 | 9.05E-05 | 0.000221053 | 0.000418126 | 0.000181512 | 0.000153606 | 0.000239578 | 0.000148629 | 0.000128684 |
| d__Bacteria;k__norank_d__Bacteria;p__Actinobacteriota;c__unclassified_p__Actinobacteriota;o__unclassified_p__Actinobacteriota;f__unclassified_p__Actinobacteriota;g__unclassified_p__Actinobacteriota | 0.000131216 | 0.000282225 | 0.000122612 | 0.000235869 | 0.000158443 | 0.000243158 | 0.00018293 | 0.000155581 | 0.000384015 | 0.000431241 | 0.0001734 | 0.000128684 |
| d__Bacteria;k__norank_d__Bacteria;p__Proteobacteria;c__Alphaproteobacteria;o__Caulobacterales;f__Caulobacteraceae;g__Asticcacaulis | 5.25E-05 | 0.000102627 | 0.000196179 | 0 | 4.53E-05 | 2.21E-05 | 0.00054879 | 0.000700117 | 0.000870433 | 0 | 7.43E-05 | 0 |
| d__Bacteria;k__norank_d__Bacteria;p__Bacteroidota;c__Bacteroidia;o__Chitinophagales;f__Chitinophagaceae;g__Niastella | 0.00015746 | 0.000282225 | 0.000564016 | 6.43E-05 | 9.05E-05 | 0.000353685 | 0.000235196 | 0.000207442 | 0.000230409 | 0.000239578 | 0.000123857 | 2.57E-05 |
| d__Bacteria;k__norank_d__Bacteria;p__Proteobacteria;c__Alphaproteobacteria;o__Holosporales;f__Holosporaceae;g__norank_f__Holosporaceae | 0.000472379 | 0.000538793 | 0.000441404 | 8.58E-05 | 0.000113173 | 0.000154737 | 0.000156797 | 0.000129651 | 0.000179207 | 9.58E-05 | 7.43E-05 | 0.000102947 |
| d__Bacteria;k__norank_d__Bacteria;p__Proteobacteria;c__Alphaproteobacteria;o__Rhodospirillales;f__norank_o__Rhodospirillales;g__norank_f__norank_o__Rhodospirillales | 0.000209946 | 0.000128284 | 0.000294269 | 8.58E-05 | 0.000135808 | 4.42E-05 | 0.000287461 | 0.000285233 | 0.000537621 | 0.000215621 | 4.95E-05 | 0.000257367 |
| d__Bacteria;k__norank_d__Bacteria;p__Firmicutes;c__Clostridia;o__Lachnospirales;f__Lachnospiraceae;g__Lachnospiraceae_UCG-007 | 0.000813542 | 0.000487479 | 0.000294269 | 0.000257312 | 0.000181077 | 0 | 0.00018293 | 0.000259302 | 0 | 0 | 0 | 5.15E-05 |
| d__Bacteria;k__norank_d__Bacteria;p__Chloroflexi;c__Anaerolineae;o__SJA-15;f__norank_o__SJA-15;g__norank_f__norank_o__SJA-15 | 0.000577352 | 0.000230911 | 0.000441404 | 0.000300197 | 0.000226347 | 0.000287369 | 2.61E-05 | 0.000155581 | 0.000153606 | 7.19E-05 | 4.95E-05 | 0 |
| d__Bacteria;k__norank_d__Bacteria;p__Bdellovibrionota;c__Bdellovibrionia;o__Bacteriovoracales;f__Bacteriovoracaceae;g__Bacteriovorax | 0.000288676 | 0.000230911 | 0.000564016 | 0.000107213 | 0.000135808 | 0.000110527 | 0.000130664 | 0.000103721 | 0.000102404 | 0.000167705 | 0.000222943 | 0.000308841 |
| d__Bacteria;k__norank_d__Bacteria;p__Proteobacteria;c__Alphaproteobacteria;o__Rhizobiales;f__Rhizobiaceae;g__norank_f__Rhizobiaceae | 0.00015746 | 0 | 0 | 0.001522429 | 0 | 8.84E-05 | 7.84E-05 | 0 | 2.56E-05 | 0.000383325 | 7.43E-05 | 0.000128684 |
| d__Bacteria;k__norank_d__Bacteria;p__Acidobacteriota;c__Acidobacteriae;o__Subgroup_13;f__norank_o__Subgroup_13;g__norank_f__norank_o__Subgroup_13 | 0.000236189 | 0.000590107 | 0.000539493 | 8.58E-05 | 0.000135808 | 4.42E-05 | 7.84E-05 | 0.000155581 | 0.000409616 | 7.19E-05 | 4.95E-05 | 2.57E-05 |
| d__Bacteria;k__norank_d__Bacteria;p__Actinobacteriota;c__Acidimicrobiia;o__Microtrichales;f__unclassified_o__Microtrichales;g__unclassified_o__Microtrichales | 0.000288676 | 0.000153941 | 9.81E-05 | 6.43E-05 | 0.000135808 | 8.84E-05 | 0.000235196 | 0.000363023 | 0.00025601 | 0.000407283 | 7.43E-05 | 0.00023163 |
| d__Bacteria;k__norank_d__Bacteria;p__Myxococcota;c__Polyangia;o__Nannocystales;f__Nannocystaceae;g__unclassified_f__Nannocystaceae | 0.000472379 | 0 | 0.000196179 | 0.000471739 | 0.000226347 | 0.000243158 | 5.23E-05 | 0.000103721 | 0.000128005 | 2.40E-05 | 0.000272486 | 0.000205894 |
| d__Bacteria;k__norank_d__Bacteria;p__Verrucomicrobiota;c__Chlamydiae;o__Chlamydiales;f__Parachlamydiaceae;g__norank_f__Parachlamydiaceae | 0.000209946 | 0.001000616 | 0.000784718 | 0.000107213 | 6.79E-05 | 6.63E-05 | 0 | 7.78E-05 | 0 | 0 | 0 | 5.15E-05 |
| d__Bacteria;k__norank_d__Bacteria;p__Proteobacteria;c__Gammaproteobacteria;o__Xanthomonadales;f__Rhodanobacteraceae;g__Dyella | 0.000209946 | 0.000256568 | 0.000171657 | 0.000514624 | 0 | 4.42E-05 | 0.000313594 | 0.000337093 | 0.000204808 | 4.79E-05 | 0.0001734 | 5.15E-05 |
| d__Bacteria;k__norank_d__Bacteria;p__Bacteroidota;c__Bacteroidia;o__Sphingobacteriales;f__env.OPS_17;g__norank_f__env.OPS_17 | 0.000446136 | 0.000384852 | 0.000392359 | 0.000150099 | 0.000135808 | 0.000198948 | 2.61E-05 | 0.000103721 | 0.000204808 | 9.58E-05 | 0 | 0.000180157 |
| d__Bacteria;k__norank_d__Bacteria;p__Bacteroidota;c__Bacteroidia;o__Chitinophagales;f__37-13;g__norank_f__37-13 | 0.000314919 | 0.000384852 | 0.000514971 | 4.29E-05 | 2.26E-05 | 2.21E-05 | 0.000104531 | 0.000207442 | 5.12E-05 | 0.000143747 | 0.000371572 | 0.000128684 |
| d__Bacteria;k__norank_d__Bacteria;p__Fibrobacterota;c__Fibrobacteria;o__Fibrobacterales;f__Fibrobacteraceae;g__possible_genus_04 | 0.00015746 | 0.000230911 | 0.000318792 | 4.29E-05 | 0.000181077 | 0.000176842 | 0.000156797 | 2.59E-05 | 0 | 0.000263536 | 0.000544973 | 0.000205894 |
| d__Bacteria;k__norank_d__Bacteria;p__Proteobacteria;c__Alphaproteobacteria;o__Caulobacterales;f__Caulobacteraceae;g__unclassified_f__Caulobacteraceae | 0.000183703 | 7.70E-05 | 0.000147135 | 6.43E-05 | 9.05E-05 | 0.000132632 | 0.000496524 | 0.000388954 | 0.000435217 | 0.000119789 | 7.43E-05 | 7.72E-05 |
| d__Bacteria;k__norank_d__Bacteria;p__Actinobacteriota;c__Actinobacteria;o__Frankiales;f__Geodermatophilaceae;g__unclassified_f__Geodermatophilaceae | 5.25E-05 | 7.70E-05 | 0.000122612 | 0.000514624 | 9.05E-05 | 4.42E-05 | 0.000418126 | 0.000492675 | 0.000128005 | 0.000167705 | 7.43E-05 | 0.000102947 |
| d__Bacteria;k__norank_d__Bacteria;p__Myxococcota;c__Polyangia;o__Nannocystales;f__Nannocystaceae;g__Nannocystis | 0.000104973 | 0.000307882 | 0.000147135 | 0.000171541 | 0.000158443 | 0.000309474 | 0.000156797 | 0 | 0.000179207 | 0.000143747 | 0.000396344 | 0.000205894 |
| d__Bacteria;k__norank_d__Bacteria;p__Acidobacteriota;c__Blastocatellia;o__Blastocatellales;f__Blastocatellaceae;g__unclassified_f__Blastocatellaceae | 0.000183703 | 0.000153941 | 0 | 0.000150099 | 2.26E-05 | 6.63E-05 | 0.000156797 | 0.000129651 | 0.000230409 | 0.000167705 | 0.000272486 | 0.000746365 |
| d__Bacteria;k__norank_d__Bacteria;p__Actinobacteriota;c__Actinobacteria;o__Micromonosporales;f__Micromonosporaceae;g__Dactylosporangium | 0.00015746 | 2.57E-05 | 0.000147135 | 0.000192984 | 0.000158443 | 8.84E-05 | 0.000261329 | 0.000388954 | 0.000230409 | 9.58E-05 | 0.000470658 | 5.15E-05 |
| d__Bacteria;k__norank_d__Bacteria;p__Firmicutes;c__Bacilli;o__Thermoactinomycetales;f__Thermoactinomycetaceae;g__Laceyella | 0.000498622 | 0.000436166 | 0.000171657 | 6.43E-05 | 0 | 0.000176842 | 0 | 0.000103721 | 5.12E-05 | 0.00033541 | 0.000222943 | 0.000205894 |
| d__Bacteria;k__norank_d__Bacteria;p__Actinobacteriota;c__Actinobacteria;o__Kineosporiales;f__Kineosporiaceae;g__Angustibacter | 0.000104973 | 5.13E-05 | 7.36E-05 | 0.000343083 | 9.05E-05 | 8.84E-05 | 0.000496524 | 0.000155581 | 0.000614424 | 9.58E-05 | 7.43E-05 | 7.72E-05 |
| d__Bacteria;k__norank_d__Bacteria;p__Dependentiae;c__Babeliae;o__Babeliales;f__unclassified_o__Babeliales;g__unclassified_o__Babeliales | 7.87E-05 | 0.000179598 | 0.000539493 | 6.43E-05 | 0.000203712 | 0.000154737 | 0.000209063 | 0.000129651 | 0.000230409 | 7.19E-05 | 0.000222943 | 0.000180157 |
| d__Bacteria;k__norank_d__Bacteria;p__Firmicutes;c__Clostridia;o__Clostridiales;f__Clostridiaceae;g__Clostridium_sensu_stricto_6 | 0.000472379 | 0.000384852 | 0.000416881 | 0.000343083 | 0 | 4.42E-05 | 7.84E-05 | 0.000103721 | 2.56E-05 | 0.000119789 | 2.48E-05 | 0.000205894 |
| d__Bacteria;k__norank_d__Bacteria;p__Verrucomicrobiota;c__Verrucomicrobiae;o__Verrucomicrobiales;f__Verrucomicrobiaceae;g__norank_f__Verrucomicrobiaceae | 0.000104973 | 0.000487479 | 0.000416881 | 2.14E-05 | 0.000203712 | 0.000132632 | 0.000104531 | 0 | 0 | 0.000263536 | 0.000272486 | 0.000205894 |
| d__Bacteria;k__norank_d__Bacteria;p__Bacteroidota;c__Bacteroidia;o__Cytophagales;f__Cyclobacteriaceae;g__norank_f__Cyclobacteriaceae | 0.000209946 | 0.000282225 | 2.45E-05 | 0.000150099 | 0.000271616 | 0.000729475 | 0.000156797 | 2.59E-05 | 0.000128005 | 4.79E-05 | 0.000148629 | 2.57E-05 |
| d__Bacteria;k__norank_d__Bacteria;p__Proteobacteria;c__Alphaproteobacteria;o__Rickettsiales;f__SM2D12;g__norank_f__SM2D12 | 0.000288676 | 0.000179598 | 0.000367836 | 0 | 2.26E-05 | 2.21E-05 | 0.000130664 | 0.000207442 | 0.000153606 | 0.000239578 | 0.000297258 | 0.000283104 |
| d__Bacteria;k__norank_d__Bacteria;p__Proteobacteria;c__Alphaproteobacteria;o__Sphingomonadales;f__Sphingomonadaceae;g__Ellin6055 | 0.000183703 | 0 | 7.36E-05 | 0.000235869 | 0.000701675 | 0.000751581 | 0 | 0.000129651 | 5.12E-05 | 2.40E-05 | 0 | 2.57E-05 |
| d__Bacteria;k__norank_d__Bacteria;p__Actinobacteriota;c__Actinobacteria;o__Micromonosporales;f__Micromonosporaceae;g__Allocatelliglobosispora | 0.000236189 | 0.000230911 | 0.000171657 | 6.43E-05 | 0.000203712 | 0.000198948 | 0.000104531 | 0.000103721 | 0.000204808 | 0.000191663 | 0.000322029 | 0.000128684 |
| d__Bacteria;k__norank_d__Bacteria;p__Myxococcota;c__Polyangia;o__Polyangiales;f__Polyangiaceae;g__Aetherobacter | 0.000551109 | 0.000153941 | 0.000245224 | 8.58E-05 | 0.000248981 | 0.000176842 | 7.84E-05 | 0.000259302 | 0.000179207 | 2.40E-05 | 0.000148629 | 0 |
| d__Bacteria;k__norank_d__Bacteria;p__Proteobacteria;c__Gammaproteobacteria;o__Gammaproteobacteria_Incertae_Sedis;f__unclassified_o__Gammaproteobacteria_Incertae_Sedis;g__Candidatus_Berkiella | 0.000314919 | 0.000256568 | 0.000588538 | 2.14E-05 | 0.000113173 | 6.63E-05 | 7.84E-05 | 0.000103721 | 0.000128005 | 0.000263536 | 0.000123857 | 7.72E-05 |
| d__Bacteria;k__norank_d__Bacteria;p__Firmicutes;c__Clostridia;o__Clostridiales;f__Clostridiaceae;g__Clostridium_sensu_stricto_9 | 0.000498622 | 0.000282225 | 0.000269747 | 0.000235869 | 0.000248981 | 8.84E-05 | 0.000104531 | 0.000155581 | 5.12E-05 | 7.19E-05 | 4.95E-05 | 7.72E-05 |
| d__Bacteria;k__norank_d__Bacteria;p__Chloroflexi;c__Anaerolineae;o__unclassified_c__Anaerolineae;f__unclassified_c__Anaerolineae;g__unclassified_c__Anaerolineae | 7.87E-05 | 0.000282225 | 0.000147135 | 0.000128656 | 0.000271616 | 0.000132632 | 2.61E-05 | 0.000181512 | 0.000179207 | 0.000119789 | 0.000198172 | 0.000386051 |
| d__Bacteria;k__norank_d__Bacteria;p__Proteobacteria;c__Gammaproteobacteria;o__Burkholderiales;f__Nitrosomonadaceae;g__966-1 | 0.000236189 | 0.000128284 | 7.36E-05 | 0 | 9.05E-05 | 0 | 0.000209063 | 0.000129651 | 0.000435217 | 0.000311452 | 0.0001734 | 0.000334577 |
| d__Bacteria;k__norank_d__Bacteria;p__Actinobacteriota;c__Actinobacteria;o__Micromonosporales;f__Micromonosporaceae;g__Salinispora | 0.000131216 | 0.000102627 | 7.36E-05 | 0.000300197 | 0.000135808 | 0.000176842 | 0.000261329 | 0.000155581 | 0.000179207 | 0.000239578 | 0.000123857 | 0.000205894 |
| d__Bacteria;k__norank_d__Bacteria;p__Cyanobacteria;c__Cyanobacteriia;o__Cyanobacteriales;f__Phormidiaceae;g__Tychonema_CCAP_1459-11B | 0.000209946 | 0.000153941 | 0.000147135 | 0.00032164 | 0.000271616 | 0.000397896 | 0 | 0 | 0 | 0.000239578 | 9.91E-05 | 0.00023163 |
| d__Bacteria;k__norank_d__Bacteria;p__Latescibacterota;c__Latescibacteria;o__Latescibacterales;f__Latescibacteraceae;g__norank_f__Latescibacteraceae | 0.000314919 | 0.000436166 | 9.81E-05 | 0.000128656 | 6.79E-05 | 0 | 2.61E-05 | 0.000181512 | 2.56E-05 | 0.000263536 | 0.000123857 | 0.000360314 |
| d__Bacteria;k__norank_d__Bacteria;p__Proteobacteria;c__Gammaproteobacteria;o__Burkholderiales;f__Comamonadaceae;g__Hydrogenophaga | 0.000131216 | 5.13E-05 | 0.000318792 | 0.000192984 | 0.000271616 | 0.000287369 | 0.000104531 | 2.59E-05 | 5.12E-05 | 0.000119789 | 0.000445887 | 2.57E-05 |
| d__Bacteria;k__norank_d__Bacteria;p__Proteobacteria;c__Alphaproteobacteria;o__Tistrellales;f__Geminicoccaceae;g__norank_f__Geminicoccaceae | 5.25E-05 | 0.000102627 | 0.000171657 | 0.000257312 | 0.000271616 | 0.00033158 | 0.000209063 | 0.000103721 | 0.000102404 | 0.000167705 | 7.43E-05 | 0.000180157 |
| d__Bacteria;k__norank_d__Bacteria;p__Dependentiae;c__Babeliae;o__Babeliales;f__UBA12409;g__norank_f__UBA12409 | 0.000183703 | 0.000436166 | 0.000196179 | 0 | 0.000113173 | 4.42E-05 | 0.000156797 | 0.000233372 | 0.000128005 | 0.000407283 | 9.91E-05 | 2.57E-05 |
| d__Bacteria;k__norank_d__Bacteria;p__Chloroflexi;c__Anaerolineae;o__SBR1031;f__A4b;g__OLB13 | 2.62E-05 | 5.13E-05 | 4.90E-05 | 0.000128656 | 0.000248981 | 0.00037579 | 0.000339727 | 2.59E-05 | 0.000716828 | 2.40E-05 | 0 | 2.57E-05 |
| d__Bacteria;k__norank_d__Bacteria;p__Bacteroidota;c__Bacteroidia;o__Bacteroidales;f__Prolixibacteraceae;g__BSV13 | 0.000787298 | 0.000256568 | 0.000392359 | 6.43E-05 | 0.000113173 | 2.21E-05 | 0.000104531 | 0.000181512 | 5.12E-05 | 2.40E-05 | 0 | 0 |
| d__Bacteria;k__norank_d__Bacteria;p__Proteobacteria;c__Gammaproteobacteria;o__Burkholderiales;f__norank_o__Burkholderiales;g__norank_f__norank_o__Burkholderiales | 0.000472379 | 0.000179598 | 0.000392359 | 8.58E-05 | 0.000203712 | 0.000397896 | 7.84E-05 | 0 | 0 | 7.19E-05 | 2.48E-05 | 7.72E-05 |
| d__Bacteria;k__norank_d__Bacteria;p__Patescibacteria;c__Parcubacteria;o__Candidatus_Kaiserbacteria;f__norank_o__Candidatus_Kaiserbacteria;g__norank_f__norank_o__Candidatus_Kaiserbacteria | 0.000314919 | 0.000436166 | 0.000441404 | 8.58E-05 | 4.53E-05 | 4.42E-05 | 0.00018293 | 2.59E-05 | 7.68E-05 | 4.79E-05 | 0.0001734 | 0.000102947 |
| d__Bacteria;k__norank_d__Bacteria;p__Actinobacteriota;c__Actinobacteria;o__Catenulisporales;f__Catenulisporaceae;g__Catenulispora | 0 | 7.70E-05 | 2.45E-05 | 0 | 0 | 0 | 0.000418126 | 0.000388954 | 0.000460818 | 0.000407283 | 9.91E-05 | 7.72E-05 |
| d__Bacteria;k__norank_d__Bacteria;p__Firmicutes;c__Clostridia;o__norank_c__Clostridia;f__Gracilibacteraceae;g__Lutispora | 0.000419892 | 0.000179598 | 0.000220702 | 8.58E-05 | 0.00033952 | 0.000176842 | 2.61E-05 | 0.000207442 | 0 | 0.000143747 | 9.91E-05 | 2.57E-05 |
| d__Bacteria;k__norank_d__Bacteria;p__Patescibacteria;c__Saccharimonadia;o__Saccharimonadales;f__S32;g__TM7 | 0.000104973 | 0.000333539 | 0.000220702 | 0.000128656 | 4.53E-05 | 2.21E-05 | 0.000209063 | 0.000155581 | 0.000230409 | 0.000215621 | 0.000123857 | 0.000102947 |
| d__Bacteria;k__norank_d__Bacteria;p__Proteobacteria;c__Gammaproteobacteria;o__Gammaproteobacteria_Incertae_Sedis;f__unclassified_o__Gammaproteobacteria_Incertae_Sedis;g__Candidatus_Ovatusbacter | 0.000104973 | 0.000256568 | 0.000245224 | 8.58E-05 | 6.79E-05 | 8.84E-05 | 0.000261329 | 0.000207442 | 0.000102404 | 0.000239578 | 0.0001734 | 5.15E-05 |
| d__Bacteria;k__norank_d__Bacteria;p__Proteobacteria;c__Alphaproteobacteria;o__Rhizobiales;f__Rhizobiaceae;g__Phyllobacterium | 0.000682325 | 0.000230911 | 0.000245224 | 4.29E-05 | 0.000384789 | 8.84E-05 | 7.84E-05 | 5.19E-05 | 5.12E-05 | 2.40E-05 | 0 | 0 |
| d__Bacteria;k__norank_d__Bacteria;p__Firmicutes;c__Bacilli;o__Thermoactinomycetales;f__Thermoactinomycetaceae;g__Thermoactinomyces | 0.000209946 | 0.000102627 | 0.000122612 | 0.000128656 | 4.53E-05 | 4.42E-05 | 0.000104531 | 2.59E-05 | 0 | 0.000479157 | 0.000272486 | 0.000334577 |
| d__Bacteria;k__norank_d__Bacteria;p__Chloroflexi;c__Anaerolineae;o__Anaerolineales;f__Anaerolineaceae;g__Anaerolinea | 0.000236189 | 0.000359195 | 0.00090733 | 4.29E-05 | 6.79E-05 | 0.000176842 | 5.23E-05 | 0 | 2.56E-05 | 0 | 0 | 0 |
| d__Bacteria;k__norank_d__Bacteria;p__Chloroflexi;c__Anaerolineae;o__Anaerolineales;f__Anaerolineaceae;g__unclassified_f__Anaerolineaceae | 0.000498622 | 0.000487479 | 0.000588538 | 0 | 9.05E-05 | 4.42E-05 | 7.84E-05 | 2.59E-05 | 0 | 0 | 0 | 5.15E-05 |
| d__Bacteria;k__norank_d__Bacteria;p__Actinobacteriota;c__Actinobacteria;o__Micromonosporales;f__Micromonosporaceae;g__unclassified_f__Micromonosporaceae | 7.87E-05 | 5.13E-05 | 7.36E-05 | 0.000107213 | 0.000113173 | 0.000176842 | 0.000287461 | 0.000103721 | 0.000179207 | 0.000263536 | 0.000272486 | 0.00015442 |
| d__Bacteria;k__norank_d__Bacteria;p__Myxococcota;c__Myxococcia;o__Myxococcales;f__Myxococcaceae;g__Cystobacter | 0 | 0 | 0 | 0 | 4.53E-05 | 0 | 0 | 0 | 0 | 0.000814566 | 0.0001734 | 0.000823575 |
| d__Bacteria;k__norank_d__Bacteria;p__Firmicutes;c__Desulfitobacteriia;o__Desulfitobacteriales;f__Desulfitobacteriaceae;g__Desulfitobacterium | 0.000288676 | 7.70E-05 | 9.81E-05 | 0.000171541 | 4.53E-05 | 4.42E-05 | 0.000313594 | 0.000726047 | 2.56E-05 | 2.40E-05 | 0 | 2.57E-05 |
| d__Bacteria;k__norank_d__Bacteria;p__Proteobacteria;c__Alphaproteobacteria;o__Rhizobiales;f__Pleomorphomonadaceae;g__Chthonobacter | 5.25E-05 | 0.000102627 | 4.90E-05 | 0.000171541 | 0.000203712 | 0.000309474 | 0.00018293 | 0.000259302 | 0.000179207 | 2.40E-05 | 0.000198172 | 0.000102947 |
| d__Bacteria;k__norank_d__Bacteria;p__Myxococcota;c__Polyangia;o__Polyangiales;f__Polyangiaceae;g__Sorangium | 0.000262433 | 0.000128284 | 0.000147135 | 8.58E-05 | 0.000248981 | 0.000110527 | 0.000287461 | 7.78E-05 | 0.000153606 | 0.000119789 | 2.48E-05 | 0.000180157 |
| d__Bacteria;k__norank_d__Bacteria;p__Proteobacteria;c__Alphaproteobacteria;o__Rhodobacterales;f__Rhodobacteraceae;g__Rhodobacter | 0.000288676 | 2.57E-05 | 0.000245224 | 0.000278755 | 0.000181077 | 0.000309474 | 0 | 0 | 2.56E-05 | 0.000119789 | 0.000272486 | 7.72E-05 |
| d__Bacteria;k__norank_d__Bacteria;p__Firmicutes;c__Desulfotomaculia;o__Desulfotomaculales;f__norank_o__Desulfotomaculales;g__Pelotomaculum | 0.000236189 | 0.000282225 | 0.000196179 | 0.000257312 | 6.79E-05 | 6.63E-05 | 0.00018293 | 7.78E-05 | 2.56E-05 | 4.79E-05 | 0.000123857 | 0.000257367 |
| d__Bacteria;k__norank_d__Bacteria;p__Firmicutes;c__Clostridia;o__Peptostreptococcales-Tissierellales;f__Peptostreptococcaceae;g__unclassified_f__Peptostreptococcaceae | 0.000314919 | 0.000230911 | 0.000171657 | 0.000471739 | 0.000181077 | 0.000110527 | 7.84E-05 | 0.000181512 | 2.56E-05 | 4.79E-05 | 0 | 0 |
| d__Bacteria;k__norank_d__Bacteria;p__Patescibacteria;c__norank_p__Patescibacteria;o__norank_c__norank_p__Patescibacteria;f__norank_o__norank_c__norank_p__Patescibacteria;g__norank_f__norank_o__norank_c__norank_p__Patescibacteria | 0.00015746 | 0.000230911 | 2.45E-05 | 0.000278755 | 2.26E-05 | 0.000132632 | 0.00018293 | 0.000363023 | 0.000409616 | 0 | 0 | 0 |
| d__Bacteria;k__norank_d__Bacteria;p__Bacteroidota;c__Bacteroidia;o__Chitinophagales;f__Chitinophagaceae;g__Taibaiella | 0.000472379 | 5.13E-05 | 0.000294269 | 0.000214427 | 0.000181077 | 6.63E-05 | 5.23E-05 | 7.78E-05 | 0.000332813 | 0 | 2.48E-05 | 2.57E-05 |
| d__Bacteria;k__norank_d__Bacteria;p__Firmicutes;c__Bacilli;o__Bacillales;f__Bacillaceae;g__Geobacillus | 0.000131216 | 2.57E-05 | 0.000196179 | 4.29E-05 | 0 | 2.21E-05 | 7.84E-05 | 5.19E-05 | 5.12E-05 | 0.000311452 | 0.00049543 | 0.000386051 |
| d__Bacteria;k__norank_d__Bacteria;p__Firmicutes;c__Bacilli;o__Paenibacillales;f__Paenibacillaceae;g__Thermobacillus | 5.25E-05 | 7.70E-05 | 2.45E-05 | 2.14E-05 | 6.79E-05 | 0 | 0.000313594 | 0.000207442 | 0.000204808 | 0.000239578 | 0.000346801 | 0.00023163 |
| d__Bacteria;k__norank_d__Bacteria;p__Bacteroidota;c__Kryptonia;o__Kryptoniales;f__BSV26;g__norank_f__BSV26 | 0.000419892 | 0.000205255 | 0.000588538 | 2.14E-05 | 0.000113173 | 4.42E-05 | 0 | 0 | 0 | 0.000167705 | 0.000123857 | 0.000102947 |
| d__Bacteria;k__norank_d__Bacteria;p__Planctomycetota;c__Planctomycetes;o__norank_c__Planctomycetes;f__norank_o__norank_c__Planctomycetes;g__norank_f__norank_o__norank_c__Planctomycetes | 0.000104973 | 0.000179598 | 0.000465926 | 2.14E-05 | 9.05E-05 | 0.000198948 | 0.000287461 | 0.000103721 | 2.56E-05 | 0 | 0.0001734 | 0.000128684 |
| d__Bacteria;k__norank_d__Bacteria;p__Proteobacteria;c__Gammaproteobacteria;o__Burkholderiales;f__Oxalobacteraceae;g__Undibacterium | 5.25E-05 | 2.57E-05 | 4.90E-05 | 0 | 0.001199638 | 0.000110527 | 0 | 0.000155581 | 0 | 9.58E-05 | 2.48E-05 | 2.57E-05 |
| d__Bacteria;k__norank_d__Bacteria;p__Gemmatimonadota;c__BD2-11_terrestrial_group;o__norank_c__BD2-11_terrestrial_group;f__norank_o__norank_c__BD2-11_terrestrial_group;g__norank_f__norank_o__norank_c__BD2-11_terrestrial_group | 0.000209946 | 0.000282225 | 7.36E-05 | 0.000107213 | 0.000203712 | 0.000154737 | 7.84E-05 | 0.000129651 | 5.12E-05 | 0.000143747 | 0.000123857 | 0.000180157 |
| d__Bacteria;k__norank_d__Bacteria;p__Desulfobacterota;c__Desulfuromonadia;o__Geobacterales;f__Geobacteraceae;g__unclassified_f__Geobacteraceae | 0.000131216 | 0.000205255 | 0.000122612 | 6.43E-05 | 0.000746944 | 0 | 7.84E-05 | 0.000207442 | 0 | 9.58E-05 | 4.95E-05 | 0 |
| d__Bacteria;k__norank_d__Bacteria;p__Bacteroidota;c__Bacteroidia;o__Sphingobacteriales;f__unclassified_o__Sphingobacteriales;g__unclassified_o__Sphingobacteriales | 0 | 0 | 0.000122612 | 0.001522429 | 0 | 0 | 0 | 5.19E-05 | 0 | 0 | 0 | 0 |
| d__Bacteria;k__norank_d__Bacteria;p__Bacteroidota;c__Bacteroidia;o__Sphingobacteriales;f__Sphingobacteriaceae;g__Sphingobacterium | 0.001259677 | 0.000102627 | 9.81E-05 | 2.14E-05 | 2.26E-05 | 0.000110527 | 0 | 0 | 0 | 0 | 7.43E-05 | 0 |
| d__Bacteria;k__norank_d__Bacteria;p__Cyanobacteria;c__Vampirivibrionia;o__Vampirovibrionales;f__Vampirovibrionaceae;g__Vampirovibrio | 0.000209946 | 0.000333539 | 0.000441404 | 0.000171541 | 6.79E-05 | 2.21E-05 | 2.61E-05 | 0 | 0 | 9.58E-05 | 9.91E-05 | 0.000180157 |
| d__Bacteria;k__norank_d__Bacteria;p__Firmicutes;c__Limnochordia;o__Limnochordales;f__Limnochordaceae;g__norank_f__Limnochordaceae | 0.000131216 | 0.000128284 | 0.000122612 | 0.000192984 | 0.000113173 | 8.84E-05 | 0.000313594 | 7.78E-05 | 0.000204808 | 7.19E-05 | 0.000148629 | 5.15E-05 |
| d__Bacteria;k__norank_d__Bacteria;p__Firmicutes;c__Bacilli;o__Alicyclobacillales;f__Alicyclobacillaceae;g__Alicyclobacillus | 0.000262433 | 0.000256568 | 0.000245224 | 0.000128656 | 6.79E-05 | 0 | 7.84E-05 | 0.000233372 | 2.56E-05 | 0.000143747 | 7.43E-05 | 0.000102947 |
| d__Bacteria;k__norank_d__Bacteria;p__Firmicutes;c__Bacilli;o__Bacillales;f__unclassified_o__Bacillales;g__unclassified_o__Bacillales | 0.000236189 | 0.000179598 | 0.000171657 | 4.29E-05 | 6.79E-05 | 0.000132632 | 0.000156797 | 7.78E-05 | 7.68E-05 | 0.000143747 | 9.91E-05 | 0.00023163 |
| d__Bacteria;k__norank_d__Bacteria;p__Verrucomicrobiota;c__Chlamydiae;o__Chlamydiales;f__Parachlamydiaceae;g__Candidatus_Protochlamydia | 2.62E-05 | 0.000513136 | 0.000662105 | 6.43E-05 | 4.53E-05 | 4.42E-05 | 0.000104531 | 2.59E-05 | 0 | 7.19E-05 | 0 | 5.15E-05 |
| d__Bacteria;k__norank_d__Bacteria;p__Proteobacteria;c__Gammaproteobacteria;o__Burkholderiales;f__Oxalobacteraceae;g__Herbaspirillum | 0.000813542 | 7.70E-05 | 7.36E-05 | 0.000364525 | 0 | 4.42E-05 | 7.84E-05 | 5.19E-05 | 0 | 0 | 9.91E-05 | 0 |
| d__Bacteria;k__norank_d__Bacteria;p__Proteobacteria;c__Gammaproteobacteria;o__CCM19a;f__norank_o__CCM19a;g__norank_f__norank_o__CCM19a | 0.000183703 | 7.70E-05 | 0.000220702 | 2.14E-05 | 0.000158443 | 0.000110527 | 0 | 0 | 5.12E-05 | 0.000263536 | 0.000272486 | 0.00023163 |
| d__Bacteria;k__norank_d__Bacteria;p__Armatimonadota;c__Chthonomonadetes;o__Chthonomonadales;f__Chthonomonadaceae;g__Chthonomonas | 0.00015746 | 0 | 4.90E-05 | 0 | 4.53E-05 | 4.42E-05 | 0.000940783 | 0.000103721 | 5.12E-05 | 4.79E-05 | 0.000123857 | 2.57E-05 |
| d__Bacteria;k__norank_d__Bacteria;p__Proteobacteria;c__Gammaproteobacteria;o__Pseudomonadales;f__Moraxellaceae;g__norank_f__Moraxellaceae | 0.000341163 | 0.000282225 | 0.000220702 | 4.29E-05 | 2.26E-05 | 4.42E-05 | 7.84E-05 | 7.78E-05 | 5.12E-05 | 4.79E-05 | 0.000222943 | 0.00015442 |
| d__Bacteria;k__norank_d__Bacteria;p__Proteobacteria;c__Gammaproteobacteria;o__Burkholderiales;f__Burkholderiaceae;g__Lautropia | 0.000131216 | 2.57E-05 | 7.36E-05 | 0.000150099 | 0.000407424 | 0.000353685 | 0.000130664 | 7.78E-05 | 5.12E-05 | 2.40E-05 | 7.43E-05 | 7.72E-05 |
| d__Bacteria;k__norank_d__Bacteria;p__Proteobacteria;c__Alphaproteobacteria;o__Rhizobiales;f__Beijerinckiaceae;g__FFCH5858 | 0.000104973 | 7.70E-05 | 0 | 4.29E-05 | 0.000158443 | 0 | 0.000235196 | 0.000103721 | 0.000153606 | 0.000239578 | 0.0001734 | 0.000283104 |
| d__Bacteria;k__norank_d__Bacteria;p__Dependentiae;c__Babeliae;o__Babeliales;f__norank_o__Babeliales;g__norank_f__norank_o__Babeliales | 0.000131216 | 5.13E-05 | 0.000392359 | 0.000150099 | 6.79E-05 | 4.42E-05 | 0.000130664 | 7.78E-05 | 0.000128005 | 7.19E-05 | 0.000198172 | 0.000102947 |
| d__Bacteria;k__norank_d__Bacteria;p__Actinobacteriota;c__Coriobacteriia;o__OPB41;f__norank_o__OPB41;g__norank_f__norank_o__OPB41 | 0.00015746 | 0.000256568 | 4.90E-05 | 0.000128656 | 0.000294251 | 0.000154737 | 0.00018293 | 0.000103721 | 0.000128005 | 0 | 0 | 7.72E-05 |
| d__Bacteria;k__norank_d__Bacteria;p__Proteobacteria;c__Alphaproteobacteria;o__Rhodobacterales;f__Rhodobacteraceae;g__Paracoccus | 0.000236189 | 2.57E-05 | 0.000122612 | 0.000128656 | 0.000475328 | 0.000221053 | 2.61E-05 | 2.59E-05 | 5.12E-05 | 4.79E-05 | 4.95E-05 | 7.72E-05 |
| d__Bacteria;k__norank_d__Bacteria;p__Myxococcota;c__Myxococcia;o__Myxococcales;f__Vulgatibacteraceae;g__Vulgatibacter | 5.25E-05 | 0.000102627 | 9.81E-05 | 0.000278755 | 6.79E-05 | 0.000132632 | 0.000156797 | 7.78E-05 | 5.12E-05 | 0.000215621 | 0.000148629 | 0.000102947 |
| d__Bacteria;k__norank_d__Bacteria;p__Dependentiae;c__Babeliae;o__Babeliales;f__Babeliaceae;g__norank_f__Babeliaceae | 0.000419892 | 0.000128284 | 0.000196179 | 0 | 0.000203712 | 8.84E-05 | 5.23E-05 | 5.19E-05 | 2.56E-05 | 0.000215621 | 2.48E-05 | 7.72E-05 |
| d__Bacteria;k__norank_d__Bacteria;p__Myxococcota;c__Polyangia;o__unclassified_c__Polyangia;f__unclassified_c__Polyangia;g__unclassified_c__Polyangia | 0.000341163 | 0.000153941 | 0.000613061 | 2.14E-05 | 6.79E-05 | 0 | 5.23E-05 | 0 | 5.12E-05 | 2.40E-05 | 0.000148629 | 0 |
| d__Bacteria;k__norank_d__Bacteria;p__Chloroflexi;c__Chloroflexia;o__Kallotenuales;f__AKIW781;g__norank_f__AKIW781 | 0.000104973 | 0.000153941 | 9.81E-05 | 0.000150099 | 0.000113173 | 0.000154737 | 5.23E-05 | 0.000129651 | 2.56E-05 | 0.000215621 | 0.000222943 | 5.15E-05 |
| d__Bacteria;k__norank_d__Bacteria;p__Patescibacteria;c__Microgenomatia;o__norank_c__Microgenomatia;f__norank_o__norank_c__Microgenomatia;g__norank_f__norank_o__norank_c__Microgenomatia | 2.62E-05 | 5.13E-05 | 0 | 8.58E-05 | 0.000203712 | 4.42E-05 | 0.000130664 | 0.000311163 | 0.000460818 | 9.58E-05 | 2.48E-05 | 2.57E-05 |
| d__Bacteria;k__norank_d__Bacteria;p__Bacteroidota;c__Bacteroidia;o__Chitinophagales;f__Chitinophagaceae;g__UTBCD1 | 0.000209946 | 0.000128284 | 0.000122612 | 0.000128656 | 0.000294251 | 0.000110527 | 2.61E-05 | 0.000181512 | 5.12E-05 | 4.79E-05 | 0.000123857 | 2.57E-05 |
| d__Bacteria;k__norank_d__Bacteria;p__Firmicutes;c__Clostridia;o__Peptostreptococcales-Tissierellales;f__Anaerovoracaceae;g__Anaerovorax | 0.000682325 | 0.000153941 | 0.000343314 | 0 | 2.26E-05 | 6.63E-05 | 0 | 7.78E-05 | 7.68E-05 | 2.40E-05 | 0 | 0 |
| d__Bacteria;k__norank_d__Bacteria;p__Firmicutes;c__Clostridia;o__norank_c__Clostridia;f__Hungateiclostridiaceae;g__Acetivibrio | 0.000314919 | 0.000205255 | 9.81E-05 | 6.43E-05 | 0.000135808 | 4.42E-05 | 0.000104531 | 0.000207442 | 0 | 9.58E-05 | 0.000148629 | 2.57E-05 |
| d__Bacteria;k__norank_d__Bacteria;p__Myxococcota;c__Polyangia;o__Polyangiales;f__Polyangiaceae;g__norank_f__Polyangiaceae | 0.000209946 | 0.000179598 | 0.000122612 | 0.000192984 | 4.53E-05 | 0.000154737 | 2.61E-05 | 0.000155581 | 5.12E-05 | 0.000119789 | 4.95E-05 | 0.000128684 |
| d__Bacteria;k__norank_d__Bacteria;p__Acidobacteriota;c__Acidobacteriae;o__Acidobacteriales;f__Acidobacteriaceae_Subgroup_1;g__Granulicella | 2.62E-05 | 0.000102627 | 0.000318792 | 0 | 0 | 0 | 0.000261329 | 0.000207442 | 0.000486419 | 0 | 2.48E-05 | 0 |
| d__Bacteria;k__norank_d__Bacteria;p__Proteobacteria;c__Gammaproteobacteria;o__Xanthomonadales;f__Xanthomonadaceae;g__norank_f__Xanthomonadaceae | 0.000183703 | 0.000230911 | 9.81E-05 | 0 | 0 | 0 | 0.000209063 | 0.000466744 | 0.000230409 | 0 | 0 | 0 |
| d__Bacteria;k__norank_d__Bacteria;p__Firmicutes;c__Clostridia;o__Peptococcales;f__Peptococcaceae;g__norank_f__Peptococcaceae | 0.000131216 | 0.000179598 | 2.45E-05 | 4.29E-05 | 0.000113173 | 0.000287369 | 0.000261329 | 0.000103721 | 0.000102404 | 7.19E-05 | 7.43E-05 | 2.57E-05 |
| d__Bacteria;k__norank_d__Bacteria;p__Proteobacteria;c__unclassified_p__Proteobacteria;o__unclassified_p__Proteobacteria;f__unclassified_p__Proteobacteria;g__unclassified_p__Proteobacteria | 0.000341163 | 5.13E-05 | 0.000122612 | 0 | 4.53E-05 | 2.21E-05 | 0.00036586 | 5.19E-05 | 0.000102404 | 0.000191663 | 0.000123857 | 0 |
| d__Bacteria;k__norank_d__Bacteria;p__Acidobacteriota;c__Acidobacteriae;o__GOUTB8;f__norank_o__GOUTB8;g__norank_f__norank_o__GOUTB8 | 0.000813542 | 0.000205255 | 0.000171657 | 0 | 0.000135808 | 0 | 2.61E-05 | 0 | 5.12E-05 | 0 | 0 | 0 |
| d__Bacteria;k__norank_d__Bacteria;p__Proteobacteria;c__Gammaproteobacteria;o__Burkholderiales;f__Alcaligenaceae;g__norank_f__Alcaligenaceae | 0.000314919 | 2.57E-05 | 0.000196179 | 0 | 0 | 0.000154737 | 5.23E-05 | 0.000181512 | 0.000281611 | 0.000119789 | 2.48E-05 | 5.15E-05 |
| d__Bacteria;k__norank_d__Bacteria;p__Firmicutes;c__Symbiobacteriia;o__Symbiobacteriales;f__Symbiobacteraceae;g__Symbiobacterium | 7.87E-05 | 0.000128284 | 9.81E-05 | 4.29E-05 | 0.000226347 | 6.63E-05 | 0.000235196 | 0 | 0.000128005 | 0.000119789 | 0.000247715 | 2.57E-05 |
| d__Bacteria;k__norank_d__Bacteria;p__Chloroflexi;c__Ktedonobacteria;o__Ktedonobacterales;f__Ktedonobacteraceae;g__1959-1 | 7.87E-05 | 0.000436166 | 0.000539493 | 8.58E-05 | 9.05E-05 | 0 | 0.000130664 | 2.59E-05 | 0 | 0 | 0 | 0 |
| d__Bacteria;k__norank_d__Bacteria;p__Cyanobacteria;c__Vampirivibrionia;o__Obscuribacterales;f__Obscuribacteraceae;g__norank_f__Obscuribacteraceae | 7.87E-05 | 0.000230911 | 0.000318792 | 0 | 0 | 0 | 7.84E-05 | 0.000103721 | 0.000102404 | 0.000119789 | 0.000148629 | 0.000205894 |
| d__Bacteria;k__norank_d__Bacteria;p__Proteobacteria;c__Alphaproteobacteria;o__Rhizobiales;f__Beijerinckiaceae;g__unclassified_f__Beijerinckiaceae | 7.87E-05 | 7.70E-05 | 2.45E-05 | 6.43E-05 | 0.000181077 | 0.000198948 | 0.000444259 | 0.000233372 | 7.68E-05 | 0 | 0 | 0 |
| d__Bacteria;k__norank_d__Bacteria;p__Patescibacteria;c__Dojkabacteria;o__norank_c__Dojkabacteria;f__norank_o__norank_c__Dojkabacteria;g__norank_f__norank_o__norank_c__Dojkabacteria | 2.62E-05 | 5.13E-05 | 0.000122612 | 0.000171541 | 6.79E-05 | 0.000110527 | 0.000235196 | 0.000129651 | 5.12E-05 | 2.40E-05 | 0.000247715 | 0.000128684 |
| d__Bacteria;k__norank_d__Bacteria;p__Firmicutes;c__Clostridia;o__Lachnospirales;f__Lachnospiraceae;g__Herbinix | 0.000183703 | 0.000487479 | 0.000318792 | 4.29E-05 | 0.000181077 | 0 | 0 | 0 | 0.000128005 | 2.40E-05 | 0 | 0 |
| d__Bacteria;k__norank_d__Bacteria;p__Chloroflexi;c__Chloroflexia;o__Chloroflexales;f__Chloroflexaceae;g__unclassified_f__Chloroflexaceae | 2.62E-05 | 0 | 2.45E-05 | 2.14E-05 | 6.79E-05 | 0.000132632 | 0 | 0 | 0 | 2.40E-05 | 0.000990859 | 7.72E-05 |
| d__Bacteria;k__norank_d__Bacteria;p__Acidobacteriota;c__Vicinamibacteria;o__Vicinamibacterales;f__Vicinamibacteraceae;g__Vicinamibacter | 2.62E-05 | 0 | 0 | 0 | 6.79E-05 | 4.42E-05 | 5.23E-05 | 0 | 0.000204808 | 0.000383325 | 0.0001734 | 0.000411787 |
| d__Bacteria;k__norank_d__Bacteria;p__Actinobacteriota;c__Acidimicrobiia;o__Microtrichales;f__Microtrichaceae;g__IMCC26207 | 0.000183703 | 0 | 9.81E-05 | 2.14E-05 | 4.53E-05 | 0.000176842 | 0.000209063 | 0.000207442 | 7.68E-05 | 0.000191663 | 2.48E-05 | 0.000128684 |
| d__Bacteria;k__norank_d__Bacteria;p__Actinobacteriota;c__Actinobacteria;o__Corynebacteriales;f__Nocardiaceae;g__Nocardia | 0.000104973 | 5.13E-05 | 4.90E-05 | 8.58E-05 | 0.000113173 | 0.000265264 | 0.000156797 | 5.19E-05 | 0.000102404 | 7.19E-05 | 2.48E-05 | 0.000283104 |
| d__Bacteria;k__norank_d__Bacteria;p__Elusimicrobiota;c__Lineage_IIb;o__norank_c__Lineage_IIb;f__norank_o__norank_c__Lineage_IIb;g__norank_f__norank_o__norank_c__Lineage_IIb | 0.000131216 | 0.000256568 | 0.000294269 | 0 | 0.000158443 | 0 | 0 | 0.000129651 | 7.68E-05 | 7.19E-05 | 9.91E-05 | 0.000128684 |
| d__Bacteria;k__norank_d__Bacteria;p__Acidobacteriota;c__Acidobacteriae;o__Acidobacteriales;f__Acidobacteriaceae_Subgroup_1;g__Acidipila | 0.00015746 | 0.000102627 | 0.000245224 | 4.29E-05 | 2.26E-05 | 0 | 0.000156797 | 0.000155581 | 0.000435217 | 2.40E-05 | 0 | 0 |
| d__Bacteria;k__norank_d__Bacteria;p__Verrucomicrobiota;c__Chlamydiae;o__Chlamydiales;f__Simkaniaceae;g__unclassified_f__Simkaniaceae | 0.000104973 | 0.000590107 | 0.000269747 | 8.58E-05 | 0.000135808 | 0 | 2.61E-05 | 5.19E-05 | 0 | 0 | 0 | 7.72E-05 |
| d__Bacteria;k__norank_d__Bacteria;p__Actinobacteriota;c__Actinobacteria;o__Streptosporangiales;f__Streptosporangiaceae;g__Thermopolyspora | 0 | 0 | 0 | 4.29E-05 | 0 | 0 | 0.00036586 | 0.000181512 | 0.000230409 | 0.00033541 | 0.000148629 | 2.57E-05 |
| d__Bacteria;k__norank_d__Bacteria;p__Proteobacteria;c__Gammaproteobacteria;o__Burkholderiales;f__Comamonadaceae;g__Ideonella | 0.000446136 | 0.000205255 | 0.000122612 | 0 | 6.79E-05 | 0 | 7.84E-05 | 0.000129651 | 7.68E-05 | 0 | 0.000148629 | 5.15E-05 |
| d__Bacteria;k__norank_d__Bacteria;p__Myxococcota;c__Polyangia;o__Polyangiales;f__Sandaracinaceae;g__Sandaracinus | 0.000131216 | 5.13E-05 | 0.000122612 | 2.14E-05 | 0.00033952 | 4.42E-05 | 0 | 0.000103721 | 2.56E-05 | 9.58E-05 | 0.000297258 | 7.72E-05 |
| d__Bacteria;k__norank_d__Bacteria;p__Firmicutes;c__Clostridia;o__Peptostreptococcales-Tissierellales;f__norank_o__Peptostreptococcales-Tissierellales;g__Alkaliphilus | 0.000236189 | 7.70E-05 | 9.81E-05 | 0.000192984 | 0.000135808 | 0 | 0.00018293 | 0.000103721 | 7.68E-05 | 0.000119789 | 4.95E-05 | 2.57E-05 |
| d__Bacteria;k__norank_d__Bacteria;p__Proteobacteria;c__Gammaproteobacteria;o__Steroidobacterales;f__Woeseiaceae;g__JTB255_marine_benthic_group | 7.87E-05 | 0.000179598 | 0.000294269 | 0 | 4.53E-05 | 6.63E-05 | 5.23E-05 | 0.000103721 | 2.56E-05 | 0.000191663 | 0.000123857 | 0.000128684 |
| d__Bacteria;k__norank_d__Bacteria;p__Firmicutes;c__Clostridia;o__Peptostreptococcales-Tissierellales;f__norank_o__Peptostreptococcales-Tissierellales;g__Tissierella | 0.000524866 | 7.70E-05 | 0.000318792 | 0.000128656 | 2.26E-05 | 0 | 7.84E-05 | 0.000103721 | 0 | 2.40E-05 | 0 | 0 |
| d__Bacteria;k__norank_d__Bacteria;p__Firmicutes;c__Bacilli;o__Bacillales;f__Bacillaceae;g__norank_f__Bacillaceae | 0.000236189 | 0.000153941 | 0.000245224 | 0.000150099 | 9.05E-05 | 2.21E-05 | 5.23E-05 | 0.000181512 | 7.68E-05 | 2.40E-05 | 0 | 2.57E-05 |
| d__Bacteria;k__norank_d__Bacteria;p__Proteobacteria;c__Gammaproteobacteria;o__WD260;f__norank_o__WD260;g__norank_f__norank_o__WD260 | 0.000131216 | 0.000128284 | 4.90E-05 | 0 | 0 | 0 | 7.84E-05 | 0.000285233 | 0.000358414 | 7.19E-05 | 0.000148629 | 0 |
| d__Bacteria;k__norank_d__Bacteria;p__Cyanobacteria;c__Cyanobacteriia;o__Cyanobacteriales;f__Cyanobacteriales_Incertae_Sedis;g__Phormidium_SAG_81.79 | 0 | 0 | 0 | 2.14E-05 | 9.05E-05 | 0.000198948 | 0 | 0 | 0 | 7.19E-05 | 0.000867002 | 0 |
| d__Bacteria;k__norank_d__Bacteria;p__Cyanobacteria;c__Vampirivibrionia;o__Vampirovibrionales;f__norank_o__Vampirovibrionales;g__norank_f__norank_o__Vampirovibrionales | 0.000288676 | 0.000179598 | 0.000147135 | 0.000107213 | 2.26E-05 | 6.63E-05 | 5.23E-05 | 5.19E-05 | 0.000153606 | 7.19E-05 | 0 | 0.000102947 |
| d__Bacteria;k__norank_d__Bacteria;p__Firmicutes;c__Clostridia;o__unclassified_c__Clostridia;f__unclassified_c__Clostridia;g__unclassified_c__Clostridia | 0.000288676 | 0.000179598 | 9.81E-05 | 0.000150099 | 4.53E-05 | 6.63E-05 | 0.000130664 | 0.000207442 | 7.68E-05 | 0 | 0 | 0 |
| d__Bacteria;k__norank_d__Bacteria;p__Bacteroidota;c__Bacteroidia;o__Cytophagales;f__Cyclobacteriaceae;g__Algoriphagus | 0.000314919 | 5.13E-05 | 0 | 0.000343083 | 6.79E-05 | 8.84E-05 | 5.23E-05 | 2.59E-05 | 0 | 9.58E-05 | 0.000123857 | 7.72E-05 |
| d__Bacteria;k__norank_d__Bacteria;p__Cyanobacteria;c__Cyanobacteriia;o__Cyanobacteriales;f__Nostocaceae;g__Cylindrospermum_PCC-7417 | 2.62E-05 | 0.000333539 | 7.36E-05 | 0.000128656 | 0.000158443 | 0 | 0.000209063 | 2.59E-05 | 0.000230409 | 0 | 2.48E-05 | 2.57E-05 |
| d__Bacteria;k__norank_d__Bacteria;p__Actinobacteriota;c__Actinobacteria;o__Micrococcales;f__Cellulomonadaceae;g__Actinotalea | 0 | 2.57E-05 | 0 | 2.14E-05 | 0 | 2.21E-05 | 0.00018293 | 0.000414884 | 0.000537621 | 0 | 0 | 2.57E-05 |
| d__Bacteria;k__norank_d__Bacteria;p__Actinobacteriota;c__Actinobacteria;o__Streptosporangiales;f__Streptosporangiaceae;g__unclassified_f__Streptosporangiaceae | 5.25E-05 | 2.57E-05 | 4.90E-05 | 2.14E-05 | 0 | 0 | 0.000209063 | 0.000259302 | 0.000486419 | 2.40E-05 | 0 | 0.000102947 |
| d__Bacteria;k__norank_d__Bacteria;p__Firmicutes;c__Bacilli;o__Bacillales;f__Sporolactobacillaceae;g__unclassified_f__Sporolactobacillaceae | 0.00015746 | 0.000410509 | 0.000147135 | 0.000214427 | 0 | 2.21E-05 | 0 | 5.19E-05 | 2.56E-05 | 0.000119789 | 2.48E-05 | 5.15E-05 |
| d__Bacteria;k__norank_d__Bacteria;p__Zixibacteria;c__norank_p__Zixibacteria;o__norank_c__norank_p__Zixibacteria;f__norank_o__norank_c__norank_p__Zixibacteria;g__norank_f__norank_o__norank_c__norank_p__Zixibacteria | 0.00015746 | 0.000102627 | 0.000171657 | 4.29E-05 | 6.79E-05 | 0 | 2.61E-05 | 0 | 0 | 2.40E-05 | 0.000470658 | 0.00015442 |
| d__Bacteria;k__norank_d__Bacteria;p__Bacteroidota;c__Bacteroidia;o__Sphingobacteriales;f__S15-21;g__norank_f__S15-21 | 0 | 0 | 0 | 0 | 0 | 0 | 0 | 0 | 0 | 0.000455199 | 0.000371572 | 0.000386051 |
| d__Bacteria;k__norank_d__Bacteria;p__Cyanobacteria;c__Cyanobacteriia;o__Cyanobacteriales;f__Nostocaceae;g__unclassified_f__Nostocaceae | 7.87E-05 | 0 | 4.90E-05 | 0.000257312 | 0.000316885 | 8.84E-05 | 7.84E-05 | 0.000181512 | 0.000128005 | 0 | 0 | 2.57E-05 |
| d__Bacteria;k__norank_d__Bacteria;p__Desulfobacterota;c__Desulfuromonadia;o__Geobacterales;f__Geobacteraceae;g__Geotalea | 0.00015746 | 0.000256568 | 0.000122612 | 0 | 6.79E-05 | 2.21E-05 | 0 | 0.000518605 | 2.56E-05 | 0 | 2.48E-05 | 0 |
| d__Bacteria;k__norank_d__Bacteria;p__Firmicutes;c__Clostridia;o__Clostridiales;f__Clostridiaceae;g__unclassified_f__Clostridiaceae | 0.000341163 | 0.000282225 | 9.81E-05 | 4.29E-05 | 0.000203712 | 2.21E-05 | 0 | 7.78E-05 | 7.68E-05 | 0 | 4.95E-05 | 0 |
| d__Bacteria;k__norank_d__Bacteria;p__Bacteroidota;c__Bacteroidia;o__Bacteroidales;f__Bacteroidetes_vadinHA17;g__norank_f__Bacteroidetes_vadinHA17 | 0.000603595 | 5.13E-05 | 0.000220702 | 0 | 0.000135808 | 6.63E-05 | 5.23E-05 | 2.59E-05 | 2.56E-05 | 0 | 0 | 0 |
| d__Bacteria;k__norank_d__Bacteria;p__Armatimonadota;c__Armatimonadia;o__Armatimonadales;f__norank_o__Armatimonadales;g__norank_f__norank_o__Armatimonadales | 0 | 0 | 9.81E-05 | 0.000150099 | 2.26E-05 | 0.000110527 | 0.00018293 | 2.59E-05 | 2.56E-05 | 0.000215621 | 0.000272486 | 7.72E-05 |
| d__Bacteria;k__norank_d__Bacteria;p__Proteobacteria;c__Alphaproteobacteria;o__Micavibrionales;f__norank_o__Micavibrionales;g__norank_f__norank_o__Micavibrionales | 2.62E-05 | 0 | 9.81E-05 | 6.43E-05 | 0 | 4.42E-05 | 0.000313594 | 2.59E-05 | 0.000230409 | 4.79E-05 | 0.000247715 | 7.72E-05 |
| d__Bacteria;k__norank_d__Bacteria;p__Spirochaetota;c__Spirochaetia;o__Spirochaetales;f__Spirochaetaceae;g__Spirochaeta | 0.000708568 | 7.70E-05 | 0.000245224 | 0 | 0 | 2.21E-05 | 2.61E-05 | 0 | 0 | 9.58E-05 | 0 | 0 |
| d__Bacteria;k__norank_d__Bacteria;p__Actinobacteriota;c__Actinobacteria;o__Micromonosporales;f__Micromonosporaceae;g__Catellatospora | 0.000419892 | 0.000102627 | 0 | 4.29E-05 | 2.26E-05 | 4.42E-05 | 7.84E-05 | 0.000155581 | 5.12E-05 | 9.58E-05 | 4.95E-05 | 0.000102947 |
| d__Bacteria;k__norank_d__Bacteria;p__Firmicutes;c__Clostridia;o__norank_c__Clostridia;f__Gracilibacteraceae;g__Gracilibacter | 0.000314919 | 0.000179598 | 0.000122612 | 0.000171541 | 9.05E-05 | 0.000110527 | 2.61E-05 | 2.59E-05 | 0 | 7.19E-05 | 2.48E-05 | 2.57E-05 |
| d__Bacteria;k__norank_d__Bacteria;p__Firmicutes;c__Negativicutes;o__Veillonellales-Selenomonadales;f__Sporomusaceae;g__Pelosinus | 2.62E-05 | 0.000102627 | 0.000122612 | 2.14E-05 | 6.79E-05 | 0.000154737 | 0.000418126 | 5.19E-05 | 5.12E-05 | 9.58E-05 | 0 | 5.15E-05 |
| d__Bacteria;k__norank_d__Bacteria;p__Myxococcota;c__Myxococcia;o__Myxococcales;f__Myxococcaceae;g__KD3-10 | 7.87E-05 | 0.000282225 | 0.000171657 | 0.000128656 | 9.05E-05 | 0.000132632 | 2.61E-05 | 0.000129651 | 2.56E-05 | 7.19E-05 | 0 | 2.57E-05 |
| d__Bacteria;k__norank_d__Bacteria;p__Proteobacteria;c__Gammaproteobacteria;o__Burkholderiales;f__Nitrosomonadaceae;g__Nitrosomonas | 0.000131216 | 0 | 4.90E-05 | 2.14E-05 | 0.000203712 | 2.21E-05 | 0.000130664 | 0.000129651 | 0.000128005 | 0.000143747 | 0.0001734 | 2.57E-05 |
| d__Bacteria;k__norank_d__Bacteria;p__Chloroflexi;c__Dehalococcoidia;o__661239;f__norank_o__661239;g__norank_f__norank_o__661239 | 0.000629839 | 0.000128284 | 0.000245224 | 8.58E-05 | 4.53E-05 | 2.21E-05 | 0 | 0 | 0 | 0 | 0 | 0 |
| d__Bacteria;k__norank_d__Bacteria;p__Firmicutes;c__Clostridia;o__Peptostreptococcales-Tissierellales;f__Sedimentibacteraceae;g__Sedimentibacter | 0.000209946 | 0.000128284 | 9.81E-05 | 0.000257312 | 9.05E-05 | 6.63E-05 | 2.61E-05 | 7.78E-05 | 2.56E-05 | 9.58E-05 | 7.43E-05 | 0 |
| d__Bacteria;k__norank_d__Bacteria;p__Proteobacteria;c__Gammaproteobacteria;o__Burkholderiales;f__Comamonadaceae;g__Piscinibacter | 5.25E-05 | 0.000102627 | 4.90E-05 | 0.000192984 | 6.79E-05 | 4.42E-05 | 0.00018293 | 7.78E-05 | 0.000102404 | 7.19E-05 | 0.000148629 | 5.15E-05 |
| d__Bacteria;k__norank_d__Bacteria;p__Proteobacteria;c__Gammaproteobacteria;o__Xanthomonadales;f__Rhodanobacteraceae;g__Luteibacter | 0.000209946 | 0.000102627 | 0 | 0 | 4.53E-05 | 6.63E-05 | 0.000261329 | 0.000129651 | 0.000230409 | 4.79E-05 | 2.48E-05 | 2.57E-05 |
| d__Bacteria;k__norank_d__Bacteria;p__Patescibacteria;c__Kazania;o__norank_c__Kazania;f__norank_o__norank_c__Kazania;g__norank_f__norank_o__norank_c__Kazania | 0.000314919 | 0.000102627 | 0.000171657 | 0 | 0.000181077 | 6.63E-05 | 0 | 5.19E-05 | 0 | 2.40E-05 | 0.000198172 | 2.57E-05 |
| d__Bacteria;k__norank_d__Bacteria;p__Actinobacteriota;c__Actinobacteria;o__Streptosporangiales;f__Thermomonosporaceae;g__Actinomadura | 0 | 5.13E-05 | 2.45E-05 | 6.43E-05 | 4.53E-05 | 0 | 0.000418126 | 0.000129651 | 7.68E-05 | 7.19E-05 | 0.000123857 | 0.000128684 |
| d__Bacteria;k__norank_d__Bacteria;p__Actinobacteriota;c__Actinobacteria;o__Micrococcales;f__Intrasporangiaceae;g__Intrasporangium | 7.87E-05 | 0 | 0 | 8.58E-05 | 2.26E-05 | 0.000110527 | 0.00036586 | 0.000207442 | 0.00025601 | 0 | 0 | 0 |
| d__Bacteria;k__norank_d__Bacteria;p__Patescibacteria;c__Gracilibacteria;o__norank_c__Gracilibacteria;f__norank_o__norank_c__Gracilibacteria;g__norank_f__norank_o__norank_c__Gracilibacteria | 0.00015746 | 0.000230911 | 0.000245224 | 0 | 6.79E-05 | 0 | 2.61E-05 | 0 | 2.56E-05 | 0.000167705 | 0.000123857 | 7.72E-05 |
| d__Bacteria;k__norank_d__Bacteria;p__Proteobacteria;c__Gammaproteobacteria;o__Burkholderiales;f__Comamonadaceae;g__Azohydromonas | 5.25E-05 | 0.000256568 | 4.90E-05 | 0 | 0 | 6.63E-05 | 0.000261329 | 0.000129651 | 0.000204808 | 4.79E-05 | 0 | 5.15E-05 |
| d__Bacteria;k__norank_d__Bacteria;p__Firmicutes;c__Clostridia;o__Clostridiales;f__Clostridiaceae;g__Clostridium_sensu_stricto_5 | 0.000314919 | 0.000102627 | 0 | 0.000235869 | 6.79E-05 | 2.21E-05 | 0.000235196 | 0.000103721 | 0 | 2.40E-05 | 0 | 0 |
| d__Bacteria;k__norank_d__Bacteria;p__Armatimonadota;c__DG-56;o__norank_c__DG-56;f__norank_o__norank_c__DG-56;g__norank_f__norank_o__norank_c__DG-56 | 0 | 0 | 4.90E-05 | 0.000128656 | 0.000158443 | 0.000221053 | 0.000418126 | 7.78E-05 | 5.12E-05 | 0 | 0 | 0 |
| d__Bacteria;k__norank_d__Bacteria;p__Actinobacteriota;c__Actinobacteria;o__Pseudonocardiales;f__Pseudonocardiaceae;g__unclassified_f__Pseudonocardiaceae | 0 | 0 | 0 | 6.43E-05 | 0.000362155 | 0.000353685 | 0 | 0 | 0 | 4.79E-05 | 0.000247715 | 2.57E-05 |
| d__Bacteria;k__norank_d__Bacteria;p__Firmicutes;c__Bacilli;o__Aneurinibacillales;f__Aneurinibacillaceae;g__Aneurinibacillus | 0.000262433 | 0.000102627 | 4.90E-05 | 8.58E-05 | 2.26E-05 | 4.42E-05 | 0 | 7.78E-05 | 0.000102404 | 0.000119789 | 0.000123857 | 0.000102947 |
| d__Bacteria;k__norank_d__Bacteria;p__Proteobacteria;c__Alphaproteobacteria;o__Rhodospirillales;f__Rhodospirillaceae;g__norank_f__Rhodospirillaceae | 0.000236189 | 7.70E-05 | 0.000343314 | 0 | 0.000135808 | 4.42E-05 | 7.84E-05 | 7.78E-05 | 2.56E-05 | 7.19E-05 | 0 | 0 |
| d__Bacteria;k__norank_d__Bacteria;p__Acidobacteriota;c__Acidobacteriae;o__PAUC26f;f__norank_o__PAUC26f;g__norank_f__norank_o__PAUC26f | 0.00015746 | 2.57E-05 | 0.000122612 | 2.14E-05 | 6.79E-05 | 6.63E-05 | 2.61E-05 | 7.78E-05 | 2.56E-05 | 0.000119789 | 0.000222943 | 0.00015442 |
| d__Bacteria;k__norank_d__Bacteria;p__Proteobacteria;c__Alphaproteobacteria;o__Rhizobiales;f__Beijerinckiaceae;g__Methylovirgula | 0.000209946 | 0.000102627 | 0.000171657 | 2.14E-05 | 2.26E-05 | 2.21E-05 | 0.000104531 | 0.000103721 | 0.000102404 | 0.000167705 | 0 | 5.15E-05 |
| d__Bacteria;k__norank_d__Bacteria;p__Proteobacteria;c__Gammaproteobacteria;o__Cellvibrionales;f__Spongiibacteraceae;g__BD1-7_clade | 0.000104973 | 0.000153941 | 0.000196179 | 0 | 0.000181077 | 8.84E-05 | 0 | 0 | 2.56E-05 | 9.58E-05 | 0.000123857 | 0.000102947 |
| d__Bacteria;k__norank_d__Bacteria;p__Planctomycetota;c__Phycisphaerae;o__Phycisphaerales;f__Phycisphaeraceae;g__norank_f__Phycisphaeraceae | 0.00015746 | 0.000179598 | 0.000196179 | 6.43E-05 | 6.79E-05 | 2.21E-05 | 5.23E-05 | 0 | 2.56E-05 | 0 | 0.000198172 | 0.000102947 |
| d__Bacteria;k__norank_d__Bacteria;p__Proteobacteria;c__Gammaproteobacteria;o__Oceanospirillales;f__Pseudohongiellaceae;g__BIyi10 | 5.25E-05 | 0 | 0 | 0.000107213 | 2.26E-05 | 0.000110527 | 0.000313594 | 7.78E-05 | 0.000332813 | 4.79E-05 | 0 | 0 |
| d__Bacteria;k__norank_d__Bacteria;p__Chloroflexi;c__Ktedonobacteria;o__Ktedonobacterales;f__Ktedonobacteraceae;g__HSB_OF53-F07 | 0.000104973 | 0.000230911 | 0.000416881 | 0 | 0 | 0 | 0.000130664 | 0.000181512 | 0 | 0 | 0 | 0 |
| d__Bacteria;k__norank_d__Bacteria;p__Acidobacteriota;c__Blastocatellia;o__Blastocatellales;f__Blastocatellaceae;g__Blastocatella | 5.25E-05 | 5.13E-05 | 4.90E-05 | 6.43E-05 | 0.000113173 | 2.21E-05 | 0 | 0.000207442 | 0.000204808 | 9.58E-05 | 7.43E-05 | 0.000128684 |
| d__Bacteria;k__norank_d__Bacteria;p__Proteobacteria;c__Gammaproteobacteria;o__Xanthomonadales;f__Rhodanobacteraceae;g__Ahniella | 2.62E-05 | 0.000179598 | 0.000147135 | 0 | 0.000181077 | 2.21E-05 | 7.84E-05 | 0 | 2.56E-05 | 4.79E-05 | 0.0001734 | 0.000180157 |
| d__Bacteria;k__norank_d__Bacteria;p__Proteobacteria;c__Gammaproteobacteria;o__Burkholderiales;f__Comamonadaceae;g__Caenimonas | 2.62E-05 | 0 | 0 | 8.58E-05 | 0.000113173 | 0.000243158 | 0.000156797 | 0.000129651 | 0.000204808 | 0 | 2.48E-05 | 7.72E-05 |
| d__Bacteria;k__norank_d__Bacteria;p__Myxococcota;c__Myxococcia;o__Myxococcales;f__27F-1492R;g__norank_f__27F-1492R | 0.00015746 | 2.57E-05 | 7.36E-05 | 4.29E-05 | 0.000135808 | 0.000221053 | 0 | 7.78E-05 | 0.000102404 | 4.79E-05 | 9.91E-05 | 7.72E-05 |
| d__Bacteria;k__norank_d__Bacteria;p__Bacteroidota;c__Bacteroidia;o__Chitinophagales;f__Chitinophagaceae;g__Pseudoflavitalea | 5.25E-05 | 2.57E-05 | 0.000196179 | 4.29E-05 | 6.79E-05 | 4.42E-05 | 0 | 0 | 0.000153606 | 0.000119789 | 0.000198172 | 0.00015442 |
| d__Bacteria;k__norank_d__Bacteria;p__Cyanobacteria;c__Cyanobacteriia;o__Phormidesmiales;f__Nodosilineaceae;g__Nodosilinea_PCC-7104 | 5.25E-05 | 7.70E-05 | 2.45E-05 | 0.000171541 | 0.000158443 | 0.000464212 | 0 | 0 | 0 | 0 | 9.91E-05 | 0 |
| d__Bacteria;k__norank_d__Bacteria;p__Gemmatimonadota;c__Gemmatimonadetes;o__Gemmatimonadales;f__Gemmatimonadaceae;g__Roseisolibacter | 7.87E-05 | 2.57E-05 | 4.90E-05 | 2.14E-05 | 0.000113173 | 0.000176842 | 5.23E-05 | 7.78E-05 | 5.12E-05 | 7.19E-05 | 0.000148629 | 0.000180157 |
| d__Bacteria;k__norank_d__Bacteria;p__Acidobacteriota;c__Acidobacteriae;o__Elev-16S-1166;f__norank_o__Elev-16S-1166;g__norank_f__norank_o__Elev-16S-1166 | 2.62E-05 | 0.000205255 | 0.000245224 | 0 | 0 | 2.21E-05 | 0 | 2.59E-05 | 0.000102404 | 0.000287494 | 0 | 0.000128684 |
| d__Bacteria;k__norank_d__Bacteria;p__Desulfobacterota;c__Desulfuromonadia;o__PB19;f__norank_o__PB19;g__norank_f__norank_o__PB19 | 7.87E-05 | 7.70E-05 | 0.000367836 | 0 | 4.53E-05 | 0 | 2.61E-05 | 0 | 0 | 0.000191663 | 0.000222943 | 2.57E-05 |
| d__Bacteria;k__norank_d__Bacteria;p__Proteobacteria;c__Gammaproteobacteria;o__Burkholderiales;f__Alcaligenaceae;g__Verticiella | 0.000708568 | 0.000179598 | 0.000122612 | 0 | 0 | 2.21E-05 | 0 | 0 | 0 | 0 | 0 | 0 |
| d__Bacteria;k__norank_d__Bacteria;p__Patescibacteria;c__unclassified_p__Patescibacteria;o__unclassified_p__Patescibacteria;f__unclassified_p__Patescibacteria;g__unclassified_p__Patescibacteria | 0.00015746 | 2.57E-05 | 7.36E-05 | 2.14E-05 | 4.53E-05 | 4.42E-05 | 2.61E-05 | 0.000129651 | 0.000153606 | 0.000287494 | 2.48E-05 | 2.57E-05 |
| d__Bacteria;k__norank_d__Bacteria;p__Proteobacteria;c__Alphaproteobacteria;o__Rhizobiales;f__Xanthobacteraceae;g__Ancylobacter | 0.000183703 | 5.13E-05 | 4.90E-05 | 0 | 2.26E-05 | 2.21E-05 | 0.000130664 | 0.000103721 | 7.68E-05 | 7.19E-05 | 0.000222943 | 7.72E-05 |
| d__Bacteria;k__norank_d__Bacteria;p__Firmicutes;c__Clostridia;o__Lachnospirales;f__Lachnospiraceae;g__norank_f__Lachnospiraceae | 0.000446136 | 0.000128284 | 0.000122612 | 0.000150099 | 6.79E-05 | 6.63E-05 | 2.61E-05 | 0 | 0 | 0 | 0 | 0 |
| d__Bacteria;k__norank_d__Bacteria;p__Firmicutes;c__Bacilli;o__Bacillales;f__Sporolactobacillaceae;g__Sporolactobacillus | 0.000262433 | 0.000179598 | 9.81E-05 | 0.000171541 | 6.79E-05 | 0 | 5.23E-05 | 7.78E-05 | 0 | 4.79E-05 | 4.95E-05 | 0 |
| d__Bacteria;k__norank_d__Bacteria;p__Actinobacteriota;c__Actinobacteria;o__Micrococcales;f__Intrasporangiaceae;g__Phycicoccus | 0 | 0 | 4.90E-05 | 0.000214427 | 4.53E-05 | 0.000110527 | 0.000156797 | 0.000155581 | 0.000102404 | 7.19E-05 | 7.43E-05 | 2.57E-05 |
| d__Bacteria;k__norank_d__Bacteria;p__Chloroflexi;c__Chloroflexia;o__Chloroflexales;f__Roseiflexaceae;g__unclassified_f__Roseiflexaceae | 7.87E-05 | 0.000153941 | 0.000147135 | 0 | 0 | 0 | 0.000156797 | 0.000466744 | 0 | 0 | 0 | 0 |
| d__Bacteria;k__norank_d__Bacteria;p__Firmicutes;c__Bacilli;o__Bacillales;f__Planococcaceae;g__Domibacillus | 7.87E-05 | 2.57E-05 | 9.81E-05 | 0.000128656 | 6.79E-05 | 6.63E-05 | 7.84E-05 | 0 | 0.000128005 | 9.58E-05 | 0.0001734 | 5.15E-05 |
| d__Bacteria;k__norank_d__Bacteria;p__Firmicutes;c__Clostridia;o__Lachnospirales;f__Lachnospiraceae;g__Anaerocolumna | 0.000288676 | 0.000153941 | 7.36E-05 | 0.000150099 | 2.26E-05 | 4.42E-05 | 0.000104531 | 5.19E-05 | 2.56E-05 | 4.79E-05 | 0 | 2.57E-05 |
| d__Bacteria;k__norank_d__Bacteria;p__Proteobacteria;c__Gammaproteobacteria;o__Burkholderiales;f__Gallionellaceae;g__Gallionella | 0.000209946 | 0.000153941 | 0.000588538 | 0 | 0 | 0 | 0 | 2.59E-05 | 0 | 0 | 0 | 0 |
| d__Bacteria;k__norank_d__Bacteria;p__Proteobacteria;c__Gammaproteobacteria;o__norank_c__Gammaproteobacteria;f__norank_o__norank_c__Gammaproteobacteria;g__norank_f__norank_o__norank_c__Gammaproteobacteria | 0.000104973 | 7.70E-05 | 0.000196179 | 0.000150099 | 0.000113173 | 2.21E-05 | 0 | 5.19E-05 | 0.000128005 | 7.19E-05 | 2.48E-05 | 2.57E-05 |
| d__Bacteria;k__norank_d__Bacteria;p__Chloroflexi;c__Ktedonobacteria;o__Ktedonobacterales;f__Ktedonobacteraceae;g__unclassified_f__Ktedonobacteraceae | 7.87E-05 | 0.000128284 | 0.000392359 | 8.58E-05 | 4.53E-05 | 2.21E-05 | 0.000156797 | 2.59E-05 | 2.56E-05 | 0 | 0 | 0 |
| d__Bacteria;k__norank_d__Bacteria;p__Chloroflexi;c__Ktedonobacteria;o__B12-WMSP1;f__norank_o__B12-WMSP1;g__norank_f__norank_o__B12-WMSP1 | 5.25E-05 | 7.70E-05 | 2.45E-05 | 0 | 0 | 0 | 0.000391993 | 0.000233372 | 0.000179207 | 0 | 0 | 0 |
| d__Bacteria;k__norank_d__Bacteria;p__Proteobacteria;c__Alphaproteobacteria;o__Paracaedibacterales;f__Paracaedibacteraceae;g__Candidatus_Paracaedibacter | 0.000104973 | 0.000153941 | 0.000318792 | 4.29E-05 | 0 | 0 | 0.000156797 | 5.19E-05 | 0 | 2.40E-05 | 7.43E-05 | 2.57E-05 |
| d__Bacteria;k__norank_d__Bacteria;p__Acidobacteriota;c__Acidobacteriae;o__Acidobacteriales;f__Acidobacteriaceae_Subgroup_1;g__Occallatibacter | 0.000131216 | 0.000128284 | 9.81E-05 | 0 | 0 | 0 | 0.00018293 | 0.000103721 | 0.000307212 | 0 | 0 | 0 |
| d__Bacteria;k__norank_d__Bacteria;p__Cyanobacteria;c__Cyanobacteriia;o__Cyanobacteriales;f__Phormidiaceae;g__norank_f__Phormidiaceae | 7.87E-05 | 0.000153941 | 0.000245224 | 2.14E-05 | 0.000113173 | 0.000265264 | 0 | 0 | 0 | 4.79E-05 | 2.48E-05 | 0 |
| d__Bacteria;k__norank_d__Bacteria;p__Patescibacteria;c__Berkelbacteria;o__norank_c__Berkelbacteria;f__norank_o__norank_c__Berkelbacteria;g__norank_f__norank_o__norank_c__Berkelbacteria | 0.000209946 | 0.000128284 | 9.81E-05 | 0 | 4.53E-05 | 6.63E-05 | 0 | 5.19E-05 | 0.000153606 | 0.000143747 | 4.95E-05 | 0 |
| d__Bacteria;k__norank_d__Bacteria;p__Actinobacteriota;c__Actinobacteria;o__Corynebacteriales;f__Nocardiaceae;g__unclassified_f__Nocardiaceae | 0.00015746 | 0 | 0 | 6.43E-05 | 4.53E-05 | 4.42E-05 | 2.61E-05 | 0.000181512 | 0.000102404 | 7.19E-05 | 0.000198172 | 5.15E-05 |
| d__Bacteria;k__norank_d__Bacteria;p__Firmicutes;c__Symbiobacteriia;o__Symbiobacteriales;f__Symbiobacteraceae;g__norank_f__Symbiobacteraceae | 0.000104973 | 0.000102627 | 2.45E-05 | 8.58E-05 | 4.53E-05 | 6.63E-05 | 5.23E-05 | 0.000181512 | 0 | 4.79E-05 | 0.0001734 | 5.15E-05 |
| d__Bacteria;k__norank_d__Bacteria;p__Chloroflexi;c__Chloroflexia;o__Chloroflexales;f__Chloroflexaceae;g__Oscillochloris | 5.25E-05 | 0 | 9.81E-05 | 0.000214427 | 0.000248981 | 0.000243158 | 0 | 0 | 0 | 2.40E-05 | 4.95E-05 | 0 |
| d__Bacteria;k__norank_d__Bacteria;p__Chloroflexi;c__SHA-26;o__norank_c__SHA-26;f__norank_o__norank_c__SHA-26;g__norank_f__norank_o__norank_c__SHA-26 | 0.000131216 | 0 | 9.81E-05 | 0.000257312 | 0.000113173 | 4.42E-05 | 5.23E-05 | 7.78E-05 | 0 | 0 | 0 | 0.00015442 |
| d__Bacteria;k__norank_d__Bacteria;p__Cyanobacteria;c__Cyanobacteriia;o__Cyanobacteriales;f__Coleofasciculaceae;g__Microcoleus_PCC-7113 | 2.62E-05 | 0.00056445 | 0.000171657 | 0 | 0 | 0.000110527 | 5.23E-05 | 0 | 0 | 0 | 0 | 0 |
| d__Bacteria;k__norank_d__Bacteria;p__Proteobacteria;c__Alphaproteobacteria;o__Rickettsiales;f__Mitochondria;g__norank_f__Mitochondria | 0.000183703 | 0.000153941 | 0.000196179 | 6.43E-05 | 0.000135808 | 6.63E-05 | 0 | 0 | 2.56E-05 | 2.40E-05 | 7.43E-05 | 0 |
| d__Bacteria;k__norank_d__Bacteria;p__Firmicutes;c__Bacilli;o__Bacillales;f__Bacillaceae;g__Virgibacillus | 7.87E-05 | 7.70E-05 | 0.000147135 | 0.000107213 | 9.05E-05 | 4.42E-05 | 5.23E-05 | 0.000103721 | 7.68E-05 | 7.19E-05 | 7.43E-05 | 0 |
| d__Bacteria;k__norank_d__Bacteria;p__Firmicutes;c__Clostridia;o__Oscillospirales;f__Ruminococcaceae;g__Ruminococcus | 0.000262433 | 7.70E-05 | 0.000196179 | 6.43E-05 | 0.000135808 | 0 | 7.84E-05 | 0 | 0 | 7.19E-05 | 0 | 2.57E-05 |
| d__Bacteria;k__norank_d__Bacteria;p__Cyanobacteria;c__Cyanobacteriia;o__unclassified_c__Cyanobacteriia;f__unclassified_c__Cyanobacteriia;g__unclassified_c__Cyanobacteriia | 0 | 5.13E-05 | 0 | 0 | 0 | 0 | 0 | 0 | 0 | 9.58E-05 | 0.000272486 | 0.000488998 |
| d__Bacteria;k__norank_d__Bacteria;p__Myxococcota;c__Polyangia;o__Polyangiales;f__Polyangiaceae;g__unclassified_f__Polyangiaceae | 0.000262433 | 0.000102627 | 2.45E-05 | 6.43E-05 | 2.26E-05 | 4.42E-05 | 0.000130664 | 0.000103721 | 7.68E-05 | 2.40E-05 | 2.48E-05 | 2.57E-05 |
| d__Bacteria;k__norank_d__Bacteria;p__Patescibacteria;c__Parcubacteria;o__Candidatus_Yanofskybacteria;f__norank_o__Candidatus_Yanofskybacteria;g__norank_f__norank_o__Candidatus_Yanofskybacteria | 0.000209946 | 0.000179598 | 0.000514971 | 0 | 0 | 0 | 0 | 0 | 0 | 0 | 0 | 0 |
| d__Bacteria;k__norank_d__Bacteria;p__Acidobacteriota;c__Vicinamibacteria;o__Vicinamibacterales;f__unclassified_o__Vicinamibacterales;g__unclassified_o__Vicinamibacterales | 0 | 0.000102627 | 2.45E-05 | 6.43E-05 | 2.26E-05 | 4.42E-05 | 0.00018293 | 7.78E-05 | 0.000128005 | 0 | 0.000123857 | 0.000128684 |
| d__Bacteria;k__norank_d__Bacteria;p__Proteobacteria;c__Alphaproteobacteria;o__Rhizobiales;f__Beijerinckiaceae;g__Methylobacterium-Methylorubrum | 0.000314919 | 0 | 7.36E-05 | 0 | 6.79E-05 | 0.000110527 | 7.84E-05 | 5.19E-05 | 7.68E-05 | 4.79E-05 | 2.48E-05 | 5.15E-05 |
| d__Bacteria;k__norank_d__Bacteria;p__Patescibacteria;c__Parcubacteria;o__Candidatus_Moranbacteria;f__norank_o__Candidatus_Moranbacteria;g__norank_f__norank_o__Candidatus_Moranbacteria | 0.000262433 | 0 | 0.000294269 | 8.58E-05 | 0.000203712 | 0 | 0 | 0 | 0 | 4.79E-05 | 0 | 0 |
| d__Bacteria;k__norank_d__Bacteria;p__Proteobacteria;c__Alphaproteobacteria;o__Rhizobiales;f__Beijerinckiaceae;g__Methylocella | 7.87E-05 | 0.000102627 | 0.000196179 | 4.29E-05 | 0.000135808 | 2.21E-05 | 0.000104531 | 0.000155581 | 0 | 0 | 0 | 5.15E-05 |
| d__Bacteria;k__norank_d__Bacteria;p__Patescibacteria;c__Parcubacteria;o__Candidatus_Jorgensenbacteria;f__norank_o__Candidatus_Jorgensenbacteria;g__norank_f__norank_o__Candidatus_Jorgensenbacteria | 0.000524866 | 0 | 0.000220702 | 0 | 9.05E-05 | 0 | 0 | 0 | 0 | 0 | 4.95E-05 | 0 |
| d__Bacteria;k__norank_d__Bacteria;p__Firmicutes;c__Clostridia;o__Oscillospirales;f__Oscillospiraceae;g__Sporobacter | 0.000367406 | 2.57E-05 | 7.36E-05 | 0.000128656 | 6.79E-05 | 2.21E-05 | 0 | 0 | 5.12E-05 | 7.19E-05 | 4.95E-05 | 2.57E-05 |
| d__Bacteria;k__norank_d__Bacteria;p__Proteobacteria;c__Gammaproteobacteria;o__Xanthomonadales;f__Xanthomonadaceae;g__unclassified_f__Xanthomonadaceae | 2.62E-05 | 0.000102627 | 0.000122612 | 0 | 2.26E-05 | 6.63E-05 | 0.000287461 | 7.78E-05 | 0 | 7.19E-05 | 0 | 0.000102947 |
| d__Bacteria;k__norank_d__Bacteria;p__Proteobacteria;c__Gammaproteobacteria;o__Burkholderiales;f__Hydrogenophilaceae;g__Thiobacillus | 0 | 5.13E-05 | 0.000171657 | 0 | 0.000656406 | 0 | 0 | 0 | 0 | 0 | 0 | 0 |
| d__Bacteria;k__norank_d__Bacteria;p__Proteobacteria;c__Alphaproteobacteria;o__Rhizobiales;f__Rhizobiaceae;g__Aureimonas | 0.000708568 | 0 | 0 | 6.43E-05 | 2.26E-05 | 2.21E-05 | 0 | 0 | 5.12E-05 | 0 | 0 | 0 |
| d__Bacteria;k__norank_d__Bacteria;p__Myxococcota;c__Myxococcia;o__Myxococcales;f__Myxococcaceae;g__Myxococcus | 0.000209946 | 5.13E-05 | 2.45E-05 | 0.000150099 | 6.79E-05 | 2.21E-05 | 0.000156797 | 0 | 2.56E-05 | 7.19E-05 | 2.48E-05 | 5.15E-05 |
| d__Bacteria;k__norank_d__Bacteria;p__Actinobacteriota;c__Thermoleophilia;o__Solirubrobacterales;f__unclassified_o__Solirubrobacterales;g__unclassified_o__Solirubrobacterales | 0.00015746 | 0.000153941 | 2.45E-05 | 0 | 4.53E-05 | 6.63E-05 | 0.000104531 | 7.78E-05 | 0 | 2.40E-05 | 0.000123857 | 7.72E-05 |
| d__Bacteria;k__norank_d__Bacteria;p__Proteobacteria;c__Gammaproteobacteria;o__Pseudomonadales;f__Moraxellaceae;g__Acinetobacter | 7.87E-05 | 2.57E-05 | 7.36E-05 | 4.29E-05 | 0 | 2.21E-05 | 7.84E-05 | 0.000155581 | 7.68E-05 | 9.58E-05 | 9.91E-05 | 0.000102947 |
| d__Bacteria;k__norank_d__Bacteria;p__Methylomirabilota;c__Methylomirabilia;o__Methylomirabilales;f__Methylomirabilaceae;g__Candidatus_Methylomirabilis | 0.000131216 | 0.000128284 | 4.90E-05 | 0 | 0.000135808 | 0.000132632 | 0 | 0 | 0 | 7.19E-05 | 9.91E-05 | 0.000102947 |
| d__Bacteria;k__norank_d__Bacteria;p__Proteobacteria;c__Gammaproteobacteria;o__Enterobacterales;f__Erwiniaceae;g__Pantoea | 0.000183703 | 0.000128284 | 0.000147135 | 0 | 0 | 6.63E-05 | 2.61E-05 | 2.59E-05 | 2.56E-05 | 9.58E-05 | 9.91E-05 | 5.15E-05 |
| d__Bacteria;k__norank_d__Bacteria;p__Firmicutes;c__Bacilli;o__Bacillales;f__Bacillaceae;g__Ureibacillus | 7.87E-05 | 0.000205255 | 2.45E-05 | 2.14E-05 | 2.26E-05 | 0.000132632 | 0.000104531 | 0.000129651 | 0 | 2.40E-05 | 0 | 0.000102947 |
| d__Bacteria;k__norank_d__Bacteria;p__Actinobacteriota;c__Actinobacteria;o__Micromonosporales;f__Micromonosporaceae;g__Longispora | 5.25E-05 | 0 | 2.45E-05 | 0.000107213 | 0.000203712 | 0.000154737 | 5.23E-05 | 5.19E-05 | 0 | 0.000119789 | 2.48E-05 | 5.15E-05 |
| d__Bacteria;k__norank_d__Bacteria;p__Proteobacteria;c__Gammaproteobacteria;o__Acidiferrobacterales;f__Acidiferrobacteraceae;g__Sulfurifustis | 0.00015746 | 7.70E-05 | 0.000245224 | 0 | 0.000135808 | 2.21E-05 | 0 | 2.59E-05 | 0 | 4.79E-05 | 0.000123857 | 0 |
| d__Bacteria;k__norank_d__Bacteria;p__Firmicutes;c__Clostridia;o__Lachnospirales;f__Lachnospiraceae;g__Lachnoclostridium | 0.000183703 | 0.000102627 | 7.36E-05 | 8.58E-05 | 6.79E-05 | 8.84E-05 | 0 | 2.59E-05 | 0.000153606 | 0 | 4.95E-05 | 0 |
| d__Bacteria;k__norank_d__Bacteria;p__Desulfobacterota;c__Syntrophobacteria;o__Syntrophobacterales;f__Syntrophobacteraceae;g__Syntrophobacter | 0.000236189 | 7.70E-05 | 0 | 6.43E-05 | 0.000203712 | 4.42E-05 | 0 | 0.000103721 | 0 | 9.58E-05 | 0 | 0 |
| d__Bacteria;k__norank_d__Bacteria;p__Chloroflexi;c__P2-11E;o__norank_c__P2-11E;f__norank_o__norank_c__P2-11E;g__norank_f__norank_o__norank_c__P2-11E | 5.25E-05 | 0.000205255 | 4.90E-05 | 6.43E-05 | 4.53E-05 | 0 | 0.000104531 | 0 | 0.000153606 | 2.40E-05 | 7.43E-05 | 5.15E-05 |
| d__Bacteria;k__norank_d__Bacteria;p__Proteobacteria;c__Gammaproteobacteria;o__Burkholderiales;f__Rhodocyclaceae;g__unclassified_f__Rhodocyclaceae | 0.000288676 | 0.000102627 | 2.45E-05 | 0 | 0 | 0 | 0 | 2.59E-05 | 2.56E-05 | 0.000167705 | 7.43E-05 | 0.000102947 |
| d__Bacteria;k__norank_d__Bacteria;p__Firmicutes;c__Bacilli;o__Bacillales;f__Bacillaceae;g__Aeribacillus | 0 | 2.57E-05 | 2.45E-05 | 0 | 0 | 0 | 0.000209063 | 2.59E-05 | 2.56E-05 | 0.000167705 | 9.91E-05 | 0.00023163 |
| d__Bacteria;k__norank_d__Bacteria;p__Verrucomicrobiota;c__Verrucomicrobiae;o__Pedosphaerales;f__Pedosphaeraceae;g__unclassified_f__Pedosphaeraceae | 5.25E-05 | 0.000102627 | 0.000343314 | 0 | 2.26E-05 | 0 | 0 | 0 | 0.000102404 | 0 | 0.000123857 | 5.15E-05 |
| d__Bacteria;k__norank_d__Bacteria;p__Firmicutes;c__Clostridia;o__Clostridiales;f__Oxobacteraceae;g__Oxobacter | 5.25E-05 | 0.000179598 | 2.45E-05 | 6.43E-05 | 2.26E-05 | 4.42E-05 | 7.84E-05 | 0.000155581 | 0.000102404 | 0 | 7.43E-05 | 0 |
| d__Bacteria;k__norank_d__Bacteria;p__Proteobacteria;c__Alphaproteobacteria;o__Rhodospirillales;f__Magnetospiraceae;g__norank_f__Magnetospiraceae | 5.25E-05 | 2.57E-05 | 0 | 0 | 0 | 2.21E-05 | 0.000287461 | 5.19E-05 | 0.000332813 | 0 | 2.48E-05 | 0 |
| d__Bacteria;k__norank_d__Bacteria;p__Chloroflexi;c__Anaerolineae;o__Ardenticatenales;f__Ardenticatenaceae;g__norank_f__Ardenticatenaceae | 2.62E-05 | 0 | 2.45E-05 | 0 | 6.79E-05 | 0.000221053 | 0 | 5.19E-05 | 0 | 4.79E-05 | 0.000297258 | 5.15E-05 |
| d__Bacteria;k__norank_d__Bacteria;p__Proteobacteria;c__Gammaproteobacteria;o__Steroidobacterales;f__Woeseiaceae;g__Woeseia | 2.62E-05 | 0 | 0.000269747 | 6.43E-05 | 0.000203712 | 2.21E-05 | 0 | 0 | 0 | 0 | 0.000148629 | 5.15E-05 |
| d__Bacteria;k__norank_d__Bacteria;p__Elusimicrobiota;c__Elusimicrobia;o__Lineage_IV;f__norank_o__Lineage_IV;g__norank_f__norank_o__Lineage_IV | 0.000104973 | 0.000333539 | 2.45E-05 | 2.14E-05 | 0 | 4.42E-05 | 7.84E-05 | 0 | 5.12E-05 | 2.40E-05 | 9.91E-05 | 0 |
| d__Bacteria;k__norank_d__Bacteria;p__Patescibacteria;c__Parcubacteria;o__unclassified_c__Parcubacteria;f__unclassified_c__Parcubacteria;g__unclassified_c__Parcubacteria | 0.000104973 | 0.000153941 | 0.000318792 | 0 | 0 | 0 | 0 | 5.19E-05 | 2.56E-05 | 0 | 0.000123857 | 0 |
| d__Bacteria;k__norank_d__Bacteria;p__Actinobacteriota;c__Actinobacteria;o__Micrococcales;f__Intrasporangiaceae;g__Ornithinibacter | 2.62E-05 | 0 | 0 | 0.000128656 | 0.000158443 | 0.000464212 | 0 | 0 | 0 | 0 | 0 | 0 |
| d__Bacteria;k__norank_d__Bacteria;p__Firmicutes;c__Desulfitobacteriia;o__Desulfitobacteriales;f__Heliobacteriaceae;g__norank_f__Heliobacteriaceae | 7.87E-05 | 0.000153941 | 7.36E-05 | 4.29E-05 | 2.26E-05 | 2.21E-05 | 5.23E-05 | 0.000207442 | 2.56E-05 | 2.40E-05 | 7.43E-05 | 0 |
| d__Bacteria;k__norank_d__Bacteria;p__Proteobacteria;c__Gammaproteobacteria;o__Burkholderiales;f__Comamonadaceae;g__Acidovorax | 0.00015746 | 0.000102627 | 0.000196179 | 0 | 9.05E-05 | 4.42E-05 | 7.84E-05 | 2.59E-05 | 2.56E-05 | 0 | 0 | 5.15E-05 |
| d__Bacteria;k__norank_d__Bacteria;p__Bacteroidota;c__Bacteroidia;o__Cytophagales;f__Microscillaceae;g__unclassified_f__Microscillaceae | 0 | 0.000230911 | 7.36E-05 | 6.43E-05 | 2.26E-05 | 2.21E-05 | 7.84E-05 | 0.000103721 | 7.68E-05 | 0 | 9.91E-05 | 0 |
| d__Bacteria;k__norank_d__Bacteria;p__Firmicutes;c__Clostridia;o__Christensenellales;f__Christensenellaceae;g__norank_f__Christensenellaceae | 0.000288676 | 7.70E-05 | 0.000122612 | 4.29E-05 | 4.53E-05 | 8.84E-05 | 0 | 2.59E-05 | 5.12E-05 | 0 | 2.48E-05 | 0 |
| d__Bacteria;k__norank_d__Bacteria;p__Proteobacteria;c__Gammaproteobacteria;o__Burkholderiales;f__Comamonadaceae;g__Leptothrix | 2.62E-05 | 2.57E-05 | 2.45E-05 | 0 | 0 | 4.42E-05 | 0.000287461 | 2.59E-05 | 0.000230409 | 0 | 9.91E-05 | 0 |
| d__Bacteria;k__norank_d__Bacteria;p__Firmicutes;c__Bacilli;o__Thermoactinomycetales;f__Thermoactinomycetaceae;g__norank_f__Thermoactinomycetaceae | 0.00015746 | 0.000153941 | 0.000147135 | 0 | 2.26E-05 | 2.21E-05 | 2.61E-05 | 0.000103721 | 0 | 0 | 9.91E-05 | 2.57E-05 |
| d__Bacteria;k__norank_d__Bacteria;p__Verrucomicrobiota;c__Verrucomicrobiae;o__Opitutales;f__Opitutaceae;g__Opitutus | 0.000104973 | 2.57E-05 | 0.000269747 | 0 | 0 | 2.21E-05 | 5.23E-05 | 0 | 7.68E-05 | 2.40E-05 | 7.43E-05 | 0.000102947 |
| d__Bacteria;k__norank_d__Bacteria;p__Firmicutes;c__Clostridia;o__Peptostreptococcales-Tissierellales;f__Peptostreptococcaceae;g__Sporacetigenium | 0.000104973 | 0.000102627 | 0.000171657 | 0.000150099 | 4.53E-05 | 0 | 0 | 0 | 0 | 7.19E-05 | 2.48E-05 | 7.72E-05 |
| d__Bacteria;k__norank_d__Bacteria;p__Firmicutes;c__Bacilli;o__Paenibacillales;f__Paenibacillaceae;g__unclassified_f__Paenibacillaceae | 0.00015746 | 2.57E-05 | 4.90E-05 | 0.000107213 | 6.79E-05 | 0 | 0.000104531 | 0.000155581 | 2.56E-05 | 0 | 4.95E-05 | 0 |
| d__Bacteria;k__norank_d__Bacteria;p__Proteobacteria;c__Alphaproteobacteria;o__Sphingomonadales;f__Sphingomonadaceae;g__Erythrobacter | 0 | 0 | 2.45E-05 | 0.000214427 | 0.000203712 | 0.000243158 | 0 | 0 | 2.56E-05 | 2.40E-05 | 0 | 0 |
| d__Bacteria;k__norank_d__Bacteria;p__Actinobacteriota;c__Actinobacteria;o__Frankiales;f__Sporichthyaceae;g__Longivirga | 0.000209946 | 0 | 4.90E-05 | 4.29E-05 | 0 | 2.21E-05 | 5.23E-05 | 0.000129651 | 0.000153606 | 0 | 7.43E-05 | 0 |
| d__Bacteria;k__norank_d__Bacteria;p__Proteobacteria;c__Alphaproteobacteria;o__Rhizobiales;f__Rhizobiales_Incertae_Sedis;g__Phreatobacter | 0 | 7.70E-05 | 0 | 0 | 0 | 0 | 5.23E-05 | 0.000207442 | 0 | 0.000119789 | 0.0001734 | 0.000102947 |
| d__Bacteria;k__norank_d__Bacteria;p__Firmicutes;c__Negativicutes;o__Veillonellales-Selenomonadales;f__Sporomusaceae;g__Anaerosinus | 2.62E-05 | 0.000205255 | 4.90E-05 | 2.14E-05 | 4.53E-05 | 0 | 0.000130664 | 2.59E-05 | 0.000102404 | 4.79E-05 | 2.48E-05 | 5.15E-05 |
| d__Bacteria;k__norank_d__Bacteria;p__Firmicutes;c__Bacilli;o__Haloplasmatales;f__Haloplasmataceae;g__Haloplasma | 0.000131216 | 0.000153941 | 7.36E-05 | 2.14E-05 | 9.05E-05 | 0 | 7.84E-05 | 0 | 0.000102404 | 0 | 0 | 7.72E-05 |
| d__Bacteria;k__norank_d__Bacteria;p__Elusimicrobiota;c__Elusimicrobia;o__MVP-88;f__norank_o__MVP-88;g__norank_f__norank_o__MVP-88 | 0 | 0.000153941 | 0.000220702 | 2.14E-05 | 0 | 0 | 2.61E-05 | 2.59E-05 | 0.000153606 | 0 | 7.43E-05 | 5.15E-05 |
| d__Bacteria;k__norank_d__Bacteria;p__Verrucomicrobiota;c__Verrucomicrobiae;o__Chthoniobacterales;f__Terrimicrobiaceae;g__Terrimicrobium | 2.62E-05 | 5.13E-05 | 9.81E-05 | 0 | 0 | 0 | 5.23E-05 | 0 | 0.000102404 | 7.19E-05 | 0.000272486 | 5.15E-05 |
| d__Bacteria;k__norank_d__Bacteria;p__Firmicutes;c__Clostridia;o__Lachnospirales;f__Lachnospiraceae;g__Epulopiscium | 0.000104973 | 5.13E-05 | 2.45E-05 | 2.14E-05 | 0.000294251 | 2.21E-05 | 0 | 0.000103721 | 5.12E-05 | 0 | 0 | 5.15E-05 |
| d__Bacteria;k__norank_d__Bacteria;p__Proteobacteria;c__Gammaproteobacteria;o__Methylococcales;f__Methylomonadaceae;g__Methylobacter | 0.000367406 | 0.000102627 | 9.81E-05 | 0.000128656 | 0 | 0 | 0 | 2.59E-05 | 0 | 0 | 0 | 0 |
| d__Bacteria;k__norank_d__Bacteria;p__Gemmatimonadota;c__Longimicrobia;o__Longimicrobiales;f__Longimicrobiaceae;g__YC-ZSS-LKJ147 | 0 | 0 | 0 | 4.29E-05 | 2.26E-05 | 0.000132632 | 0 | 2.59E-05 | 0 | 0.000119789 | 0.000247715 | 0.000128684 |
| d__Bacteria;k__norank_d__Bacteria;p__Actinobacteriota;c__Actinobacteria;o__Kineosporiales;f__Kineosporiaceae;g__Kineosporia | 0.000104973 | 7.70E-05 | 0 | 0.000107213 | 2.26E-05 | 2.21E-05 | 0.000156797 | 2.59E-05 | 0.000128005 | 2.40E-05 | 2.48E-05 | 2.57E-05 |
| d__Bacteria;k__norank_d__Bacteria;p__Bacteroidota;c__Rhodothermia;o__Rhodothermales;f__Rhodothermaceae;g__norank_f__Rhodothermaceae | 0 | 0 | 0 | 0.000192984 | 2.26E-05 | 8.84E-05 | 0.000130664 | 0.000103721 | 5.12E-05 | 0 | 0.000123857 | 0 |
| d__Bacteria;k__norank_d__Bacteria;p__Firmicutes;c__Bacilli;o__Bacillales;f__Bacillaceae;g__Terribacillus | 2.62E-05 | 5.13E-05 | 4.90E-05 | 4.29E-05 | 4.53E-05 | 0 | 0.000156797 | 0.000233372 | 0.000102404 | 0 | 0 | 0 |
| d__Bacteria;k__norank_d__Bacteria;p__Bacteroidota;c__Bacteroidia;o__Cytophagales;f__norank_o__Cytophagales;g__norank_f__norank_o__Cytophagales | 7.87E-05 | 5.13E-05 | 2.45E-05 | 0 | 0.000135808 | 0 | 0.000156797 | 0.000129651 | 7.68E-05 | 2.40E-05 | 2.48E-05 | 0 |
| d__Bacteria;k__norank_d__Bacteria;p__Verrucomicrobiota;c__Verrucomicrobiae;o__Opitutales;f__Opitutaceae;g__Lacunisphaera | 0.000183703 | 0.000102627 | 0.000220702 | 0 | 0 | 0 | 2.61E-05 | 0 | 0 | 4.79E-05 | 0 | 0.000102947 |
| d__Bacteria;k__norank_d__Bacteria;p__Bacteroidota;c__Bacteroidia;o__Cytophagales;f__Cytophagaceae;g__Cytophaga | 0.000104973 | 0.000153941 | 9.81E-05 | 0 | 2.26E-05 | 0 | 0.000130664 | 0 | 0 | 4.79E-05 | 0.000123857 | 0 |
| d__Bacteria;k__norank_d__Bacteria;p__Verrucomicrobiota;c__Verrucomicrobiae;o__Chthoniobacterales;f__Chthoniobacteraceae;g__unclassified_f__Chthoniobacteraceae | 2.62E-05 | 0.000179598 | 0.000220702 | 0 | 2.26E-05 | 0 | 0 | 0 | 0 | 2.40E-05 | 4.95E-05 | 0.00015442 |
| d__Bacteria;k__norank_d__Bacteria;p__Proteobacteria;c__Gammaproteobacteria;o__Burkholderiales;f__Alcaligenaceae;g__unclassified_f__Alcaligenaceae | 0 | 0.000153941 | 4.90E-05 | 0 | 2.26E-05 | 4.42E-05 | 7.84E-05 | 2.59E-05 | 5.12E-05 | 2.40E-05 | 9.91E-05 | 0.000128684 |
| d__Bacteria;k__norank_d__Bacteria;p__Desulfobacterota;c__Desulfobulbia;o__Desulfobulbales;f__Desulfocapsaceae;g__norank_f__Desulfocapsaceae | 2.62E-05 | 7.70E-05 | 0.000122612 | 0 | 0.000384789 | 6.63E-05 | 0 | 0 | 0 | 0 | 0 | 0 |
| d__Bacteria;k__norank_d__Bacteria;p__Actinobacteriota;c__Actinobacteria;o__Micrococcales;f__Microbacteriaceae;g__Humibacter | 0 | 0 | 0 | 0 | 0 | 0 | 0.000522657 | 2.59E-05 | 0.000128005 | 0 | 0 | 0 |
| d__Bacteria;k__norank_d__Bacteria;p__Cyanobacteria;c__Vampirivibrionia;o__Vampirovibrionales;f__Vampirovibrionaceae;g__norank_f__Vampirovibrionaceae | 5.25E-05 | 0.000179598 | 0.000220702 | 0 | 2.26E-05 | 0 | 2.61E-05 | 5.19E-05 | 5.12E-05 | 7.19E-05 | 0 | 0 |
| d__Bacteria;k__norank_d__Bacteria;p__Bacteroidota;c__Bacteroidia;o__Chitinophagales;f__Chitinophagaceae;g__Lacibacter | 2.62E-05 | 5.13E-05 | 2.45E-05 | 8.58E-05 | 0 | 0 | 0.000156797 | 0 | 5.12E-05 | 7.19E-05 | 2.48E-05 | 0.000180157 |
| d__Bacteria;k__norank_d__Bacteria;p__Firmicutes;c__Clostridia;o__norank_c__Clostridia;f__Hungateiclostridiaceae;g__Pseudoclostridium | 2.62E-05 | 2.57E-05 | 7.36E-05 | 0 | 0 | 0 | 0.000156797 | 0.000285233 | 5.12E-05 | 0 | 0 | 5.15E-05 |
| d__Bacteria;k__norank_d__Bacteria;p__Firmicutes;c__Bacilli;o__unclassified_c__Bacilli;f__unclassified_c__Bacilli;g__unclassified_c__Bacilli | 0.000131216 | 2.57E-05 | 7.36E-05 | 8.58E-05 | 0 | 2.21E-05 | 0 | 7.78E-05 | 0 | 4.79E-05 | 7.43E-05 | 0.000128684 |
| d__Bacteria;k__norank_d__Bacteria;p__Actinobacteriota;c__Actinobacteria;o__Pseudonocardiales;f__Pseudonocardiaceae;g__Saccharomonospora | 0 | 0 | 0 | 0 | 0 | 2.21E-05 | 0.00018293 | 0.000129651 | 0.000153606 | 9.58E-05 | 2.48E-05 | 5.15E-05 |
| d__Bacteria;k__norank_d__Bacteria;p__WS4;c__norank_p__WS4;o__norank_c__norank_p__WS4;f__norank_o__norank_c__norank_p__WS4;g__norank_f__norank_o__norank_c__norank_p__WS4 | 0.000183703 | 7.70E-05 | 4.90E-05 | 6.43E-05 | 0.000158443 | 2.21E-05 | 0 | 0 | 2.56E-05 | 2.40E-05 | 4.95E-05 | 0 |
| d__Bacteria;k__norank_d__Bacteria;p__Deferrisomatota;c__Defferrisomatia;o__Defferrisomatales;f__Defferrisomataceae;g__Deferrisoma | 0.000183703 | 0.000128284 | 2.45E-05 | 4.29E-05 | 0.000248981 | 2.21E-05 | 0 | 0 | 0 | 0 | 0 | 0 |
| d__Bacteria;k__norank_d__Bacteria;p__Firmicutes;c__Bacilli;o__Thermoactinomycetales;f__Thermoactinomycetaceae;g__Planifilum | 2.62E-05 | 7.70E-05 | 9.81E-05 | 0 | 0 | 4.42E-05 | 0 | 2.59E-05 | 0 | 0.000119789 | 0.000198172 | 5.15E-05 |
| d__Bacteria;k__norank_d__Bacteria;p__Firmicutes;c__Bacilli;o__Bacillales;f__Planococcaceae;g__Planomicrobium | 7.87E-05 | 2.57E-05 | 2.45E-05 | 0 | 0 | 2.21E-05 | 7.84E-05 | 0 | 0.000332813 | 4.79E-05 | 0 | 2.57E-05 |
| d__Bacteria;k__norank_d__Bacteria;p__Proteobacteria;c__Gammaproteobacteria;o__Xanthomonadales;f__Rhodanobacteraceae;g__Chiayiivirga | 0.000131216 | 0 | 0 | 0.000107213 | 0.000135808 | 0.000154737 | 0.000104531 | 0 | 0 | 0 | 0 | 0 |
| d__Bacteria;k__norank_d__Bacteria;p__Cyanobacteria;c__Cyanobacteriia;o__Cyanobacteriales;f__Coleofasciculaceae;g__unclassified_f__Coleofasciculaceae | 0.000131216 | 0.000256568 | 0.000245224 | 0 | 0 | 0 | 0 | 0 | 0 | 0 | 0 | 0 |
| d__Bacteria;k__norank_d__Bacteria;p__Proteobacteria;c__Gammaproteobacteria;o__EC3;f__norank_o__EC3;g__norank_f__norank_o__EC3 | 7.87E-05 | 0.000282225 | 9.81E-05 | 0 | 0 | 0 | 0 | 0 | 0 | 9.58E-05 | 0 | 7.72E-05 |
| d__Bacteria;k__norank_d__Bacteria;p__Firmicutes;c__Negativicutes;o__Veillonellales-Selenomonadales;f__Sporomusaceae;g__norank_f__Sporomusaceae | 2.62E-05 | 0.000128284 | 7.36E-05 | 6.43E-05 | 2.26E-05 | 2.21E-05 | 0.000104531 | 0.000181512 | 0 | 0 | 0 | 0 |
| d__Bacteria;k__norank_d__Bacteria;p__Proteobacteria;c__Gammaproteobacteria;o__Xanthomonadales;f__Rhodanobacteraceae;g__unclassified_f__Rhodanobacteraceae | 0.00015746 | 0.000153941 | 0.000196179 | 0 | 0 | 8.84E-05 | 2.61E-05 | 0 | 0 | 0 | 0 | 0 |
| d__Bacteria;k__norank_d__Bacteria;p__Firmicutes;c__Clostridia;o__Peptostreptococcales-Tissierellales;f__norank_o__Peptostreptococcales-Tissierellales;g__Tepidimicrobium | 0.000104973 | 2.57E-05 | 0.000122612 | 2.14E-05 | 6.79E-05 | 6.63E-05 | 5.23E-05 | 5.19E-05 | 2.56E-05 | 0 | 2.48E-05 | 5.15E-05 |
| d__Bacteria;k__norank_d__Bacteria;p__Proteobacteria;c__Gammaproteobacteria;o__Burkholderiales;f__Comamonadaceae;g__norank_f__Comamonadaceae | 0 | 0 | 4.90E-05 | 0 | 0 | 0 | 0.000156797 | 0.000181512 | 0.000128005 | 7.19E-05 | 2.48E-05 | 0 |
| d__Bacteria;k__norank_d__Bacteria;p__Proteobacteria;c__Gammaproteobacteria;o__Burkholderiales;f__Gallionellaceae;g__Candidatus_Nitrotoga | 0.000236189 | 5.13E-05 | 0.000220702 | 0 | 0 | 0 | 0 | 7.78E-05 | 2.56E-05 | 0 | 0 | 0 |
| d__Bacteria;k__norank_d__Bacteria;p__Firmicutes;c__Bacilli;o__Thermoactinomycetales;f__Thermoactinomycetaceae;g__Shimazuella | 0.000209946 | 0.000102627 | 0.000122612 | 4.29E-05 | 0 | 0 | 5.23E-05 | 2.59E-05 | 0 | 4.79E-05 | 0 | 0 |
| d__Bacteria;k__norank_d__Bacteria;p__Proteobacteria;c__Alphaproteobacteria;o__Rhizobiales;f__Devosiaceae;g__unclassified_f__Devosiaceae | 0 | 0 | 0 | 0.000171541 | 0.000113173 | 0.000110527 | 0.000104531 | 0.000103721 | 0 | 0 | 0 | 0 |
| d__Bacteria;k__norank_d__Bacteria;p__Verrucomicrobiota;c__Verrucomicrobiae;o__Pedosphaerales;f__Pedosphaeraceae;g__Ellin516 | 2.62E-05 | 0.000128284 | 0.000392359 | 0 | 2.26E-05 | 0 | 0 | 0 | 2.56E-05 | 0 | 0 | 0 |
| d__Bacteria;k__norank_d__Bacteria;p__Verrucomicrobiota;c__Verrucomicrobiae;o__Chthoniobacterales;f__Chthoniobacteraceae;g__LD29 | 7.87E-05 | 7.70E-05 | 0.000147135 | 6.43E-05 | 2.26E-05 | 0 | 0.000104531 | 5.19E-05 | 0 | 2.40E-05 | 2.48E-05 | 0 |
| d__Bacteria;k__norank_d__Bacteria;p__FCPU426;c__norank_p__FCPU426;o__norank_c__norank_p__FCPU426;f__norank_o__norank_c__norank_p__FCPU426;g__norank_f__norank_o__norank_c__norank_p__FCPU426 | 2.62E-05 | 7.70E-05 | 0.000343314 | 2.14E-05 | 0 | 0 | 0 | 2.59E-05 | 0 | 0 | 7.43E-05 | 2.57E-05 |
| d__Bacteria;k__norank_d__Bacteria;p__Cyanobacteria;c__Cyanobacteriia;o__Cyanobacteriales;f__Nostocaceae;g__Nostoc_PCC-7524 | 5.25E-05 | 5.13E-05 | 0 | 4.29E-05 | 0.000226347 | 4.42E-05 | 2.61E-05 | 0 | 5.12E-05 | 4.79E-05 | 2.48E-05 | 2.57E-05 |
| d__Bacteria;k__norank_d__Bacteria;p__Patescibacteria;c__Gracilibacteria;o__Candidatus_Peribacteria;f__norank_o__Candidatus_Peribacteria;g__norank_f__norank_o__Candidatus_Peribacteria | 5.25E-05 | 5.13E-05 | 0.000122612 | 0 | 6.79E-05 | 0 | 0 | 0 | 0 | 0.000143747 | 9.91E-05 | 5.15E-05 |
| d__Bacteria;k__norank_d__Bacteria;p__Proteobacteria;c__Gammaproteobacteria;o__Burkholderiales;f__Methylophilaceae;g__norank_f__Methylophilaceae | 0.000209946 | 0 | 9.81E-05 | 0 | 2.26E-05 | 0 | 2.61E-05 | 2.59E-05 | 0.000102404 | 4.79E-05 | 0 | 5.15E-05 |
| d__Bacteria;k__norank_d__Bacteria;p__Patescibacteria;c__ABY1;o__Candidatus_Magasanikbacteria;f__norank_o__Candidatus_Magasanikbacteria;g__norank_f__norank_o__Candidatus_Magasanikbacteria | 0.00015746 | 0.000230911 | 7.36E-05 | 0 | 6.79E-05 | 0 | 0 | 0 | 0 | 2.40E-05 | 2.48E-05 | 0 |
| d__Bacteria;k__norank_d__Bacteria;p__Planctomycetota;c__Phycisphaerae;o__Phycisphaerales;f__AKAU3564_sediment_group;g__norank_f__AKAU3564_sediment_group | 2.62E-05 | 0.000179598 | 0.000147135 | 4.29E-05 | 6.79E-05 | 8.84E-05 | 0 | 0 | 2.56E-05 | 0 | 0 | 0 |
| d__Bacteria;k__norank_d__Bacteria;p__Bacteroidota;c__Bacteroidia;o__Chitinophagales;f__unclassified_o__Chitinophagales;g__unclassified_o__Chitinophagales | 2.62E-05 | 7.70E-05 | 7.36E-05 | 0 | 0 | 0 | 5.23E-05 | 0 | 5.12E-05 | 0.000119789 | 7.43E-05 | 0.000102947 |
| d__Bacteria;k__norank_d__Bacteria;p__Bacteroidota;c__Bacteroidia;o__Sphingobacteriales;f__NS11-12_marine_group;g__norank_f__NS11-12_marine_group | 0.00015746 | 2.57E-05 | 0.000122612 | 0 | 4.53E-05 | 2.21E-05 | 5.23E-05 | 2.59E-05 | 5.12E-05 | 4.79E-05 | 0 | 2.57E-05 |
| d__Bacteria;k__norank_d__Bacteria;p__Nitrospinota;c__P9X2b3D02;o__norank_c__P9X2b3D02;f__norank_o__norank_c__P9X2b3D02;g__norank_f__norank_o__norank_c__P9X2b3D02 | 0.00015746 | 0.000102627 | 4.90E-05 | 8.58E-05 | 6.79E-05 | 8.84E-05 | 0 | 0 | 0 | 2.40E-05 | 0 | 0 |
| d__Bacteria;k__norank_d__Bacteria;p__Proteobacteria;c__Alphaproteobacteria;o__Sphingomonadales;f__Sphingomonadaceae;g__Rhizorhapis | 0 | 0.000102627 | 2.45E-05 | 0 | 0 | 4.42E-05 | 5.23E-05 | 0.000103721 | 7.68E-05 | 7.19E-05 | 9.91E-05 | 0 |
| d__Bacteria;k__norank_d__Bacteria;p__Actinobacteriota;c__Actinobacteria;o__Corynebacteriales;f__Nocardiaceae;g__Williamsia | 0.000104973 | 0.000102627 | 4.90E-05 | 6.43E-05 | 2.26E-05 | 0 | 7.84E-05 | 2.59E-05 | 5.12E-05 | 7.19E-05 | 0 | 0 |
| d__Bacteria;k__norank_d__Bacteria;p__Acidobacteriota;c__Subgroup_20;o__norank_c__Subgroup_20;f__norank_o__norank_c__Subgroup_20;g__norank_f__norank_o__norank_c__Subgroup_20 | 5.25E-05 | 0.000153941 | 7.36E-05 | 2.14E-05 | 4.53E-05 | 0 | 0 | 0 | 2.56E-05 | 4.79E-05 | 9.91E-05 | 5.15E-05 |
| d__Bacteria;k__norank_d__Bacteria;p__Proteobacteria;c__Alphaproteobacteria;o__Tistrellales;f__Geminicoccaceae;g__Geminicoccus | 2.62E-05 | 0 | 0 | 0 | 6.79E-05 | 2.21E-05 | 0 | 0.000129651 | 0 | 7.19E-05 | 0.000123857 | 0.000128684 |
| d__Bacteria;k__norank_d__Bacteria;p__Patescibacteria;c__Gracilibacteria;o__Candidatus_Abawacabacteria;f__norank_o__Candidatus_Abawacabacteria;g__norank_f__norank_o__Candidatus_Abawacabacteria | 7.87E-05 | 0.000153941 | 0.000122612 | 6.43E-05 | 0 | 0 | 0 | 0 | 0 | 0 | 0.000148629 | 0 |
| d__Bacteria;k__norank_d__Bacteria;p__Actinobacteriota;c__Actinobacteria;o__Pseudonocardiales;f__Pseudonocardiaceae;g__Amycolatopsis | 0 | 0.000128284 | 0.000122612 | 2.14E-05 | 2.26E-05 | 0 | 5.23E-05 | 2.59E-05 | 5.12E-05 | 0.000143747 | 0 | 0 |
| d__Bacteria;k__norank_d__Bacteria;p__Proteobacteria;c__Alphaproteobacteria;o__Rhodobacterales;f__Rhodobacteraceae;g__Cereibacter | 2.62E-05 | 0 | 0.000122612 | 4.29E-05 | 2.26E-05 | 2.21E-05 | 7.84E-05 | 2.59E-05 | 0 | 0 | 0.0001734 | 5.15E-05 |
| d__Bacteria;k__norank_d__Bacteria;p__Desulfobacterota;c__Desulfobulbia;o__Desulfobulbales;f__Desulfobulbaceae;g__Desulfobulbus | 7.87E-05 | 0.000102627 | 7.36E-05 | 0 | 0.000135808 | 6.63E-05 | 0 | 2.59E-05 | 7.68E-05 | 0 | 0 | 0 |
| d__Bacteria;k__norank_d__Bacteria;p__Proteobacteria;c__Gammaproteobacteria;o__Burkholderiales;f__Nitrosomonadaceae;g__DSSD61 | 2.62E-05 | 0 | 0.000122612 | 6.43E-05 | 6.79E-05 | 0.000154737 | 0 | 0 | 0 | 0 | 0.000123857 | 0 |
| d__Bacteria;k__norank_d__Bacteria;p__Bacteroidota;c__Bacteroidia;o__Chitinophagales;f__Chitinophagaceae;g__Niabella | 0.000104973 | 0 | 0 | 0.000364525 | 6.79E-05 | 2.21E-05 | 0 | 0 | 0 | 0 | 0 | 0 |
| d__Bacteria;k__norank_d__Bacteria;p__Myxococcota;c__Polyangia;o__Polyangiales;f__Polyangiaceae;g__Polyangium | 5.25E-05 | 0.000128284 | 0.000122612 | 0.000107213 | 2.26E-05 | 0 | 0 | 0 | 5.12E-05 | 2.40E-05 | 2.48E-05 | 2.57E-05 |
| d__Bacteria;k__norank_d__Bacteria;p__Firmicutes;c__Clostridia;o__norank_c__Clostridia;f__Hungateiclostridiaceae;g__Anaerobacterium | 0.000104973 | 7.70E-05 | 2.45E-05 | 6.43E-05 | 2.26E-05 | 8.84E-05 | 2.61E-05 | 0 | 5.12E-05 | 2.40E-05 | 7.43E-05 | 0 |
| d__Bacteria;k__norank_d__Bacteria;p__Bacteroidota;c__Bacteroidia;o__Chitinophagales;f__Saprospiraceae;g__Phaeodactylibacter | 2.62E-05 | 2.57E-05 | 0.000196179 | 2.14E-05 | 6.79E-05 | 2.21E-05 | 0 | 2.59E-05 | 0 | 7.19E-05 | 9.91E-05 | 0 |
| d__Bacteria;k__norank_d__Bacteria;p__Bacteroidota;c__Bacteroidia;o__Sphingobacteriales;f__KD3-93;g__norank_f__KD3-93 | 0.000209946 | 2.57E-05 | 2.45E-05 | 4.29E-05 | 0 | 2.21E-05 | 0 | 0.000129651 | 0 | 0 | 4.95E-05 | 5.15E-05 |
| d__Bacteria;k__norank_d__Bacteria;p__Armatimonadota;c__Fimbriimonadia;o__Fimbriimonadales;f__unclassified_o__Fimbriimonadales;g__unclassified_o__Fimbriimonadales | 0.000131216 | 0.000102627 | 4.90E-05 | 0 | 4.53E-05 | 0 | 2.61E-05 | 0 | 2.56E-05 | 0.000143747 | 2.48E-05 | 0 |
| d__Bacteria;k__norank_d__Bacteria;p__Actinobacteriota;c__Actinobacteria;o__Pseudonocardiales;f__Pseudonocardiaceae;g__Umezawaea | 0 | 0 | 0 | 2.14E-05 | 0.000248981 | 0.000176842 | 0 | 0 | 0 | 0 | 9.91E-05 | 0 |
| d__Bacteria;k__norank_d__Bacteria;p__Cyanobacteria;c__Cyanobacteriia;o__Oxyphotobacteria_Incertae_Sedis;f__unclassified_o__Oxyphotobacteria_Incertae_Sedis;g__Leptolyngbya_ANT.L52.2 | 0 | 2.57E-05 | 2.45E-05 | 2.14E-05 | 0 | 0 | 0 | 0 | 2.56E-05 | 4.79E-05 | 0.000371572 | 2.57E-05 |
| d__Bacteria;k__norank_d__Bacteria;p__Acidobacteriota;c__Holophagae;o__Holophagales;f__Holophagaceae;g__Holophaga | 0.000236189 | 0.000153941 | 0.000122612 | 0 | 0 | 0 | 2.61E-05 | 0 | 0 | 0 | 0 | 0 |
| d__Bacteria;k__norank_d__Bacteria;p__Cyanobacteria;c__Cyanobacteriia;o__Cyanobacteriales;f__Nostocaceae;g__Nostoc_PCC-8976 | 0 | 2.57E-05 | 4.90E-05 | 0.000171541 | 0.000135808 | 0.000154737 | 0 | 0 | 0 | 0 | 0 | 0 |
| d__Bacteria;k__norank_d__Bacteria;p__Firmicutes;c__Bacilli;o__Alicyclobacillales;f__Alicyclobacillaceae;g__Effusibacillus | 0.000183703 | 2.57E-05 | 0 | 2.14E-05 | 0 | 0.000154737 | 2.61E-05 | 0 | 0 | 0 | 0.000123857 | 0 |
| d__Bacteria;k__norank_d__Bacteria;p__Proteobacteria;c__Gammaproteobacteria;o__Burkholderiales;f__Comamonadaceae;g__Pelomonas | 2.62E-05 | 2.57E-05 | 0 | 0.000150099 | 4.53E-05 | 0 | 0.00018293 | 5.19E-05 | 2.56E-05 | 0 | 0 | 2.57E-05 |
| d__Bacteria;k__norank_d__Bacteria;p__Actinobacteriota;c__Actinobacteria;o__Pseudonocardiales;f__Pseudonocardiaceae;g__Saccharopolyspora | 0 | 0 | 0 | 2.14E-05 | 0 | 0 | 0.000130664 | 0.000181512 | 0.000102404 | 7.19E-05 | 2.48E-05 | 0 |
| d__Bacteria;k__norank_d__Bacteria;p__Firmicutes;c__unclassified_p__Firmicutes;o__unclassified_p__Firmicutes;f__unclassified_p__Firmicutes;g__unclassified_p__Firmicutes | 0 | 0.000230911 | 4.90E-05 | 0 | 0 | 0 | 0.000130664 | 0 | 0 | 7.19E-05 | 4.95E-05 | 0 |
| d__Bacteria;k__norank_d__Bacteria;p__Firmicutes;c__Clostridia;o__Caldicoprobacterales;f__Caldicoprobacteraceae;g__Caldicoprobacter | 0 | 7.70E-05 | 9.81E-05 | 4.29E-05 | 0.000135808 | 0 | 5.23E-05 | 2.59E-05 | 2.56E-05 | 4.79E-05 | 0 | 2.57E-05 |
| d__Bacteria;k__norank_d__Bacteria;p__Proteobacteria;c__Gammaproteobacteria;o__Burkholderiales;f__Gallionellaceae;g__Sideroxydans | 0.000131216 | 2.57E-05 | 0.000147135 | 0 | 0.000181077 | 4.42E-05 | 0 | 0 | 0 | 0 | 0 | 0 |
| d__Bacteria;k__norank_d__Bacteria;p__Proteobacteria;c__Gammaproteobacteria;o__Ga0077536;f__norank_o__Ga0077536;g__norank_f__norank_o__Ga0077536 | 7.87E-05 | 0.000256568 | 2.45E-05 | 6.43E-05 | 0 | 4.42E-05 | 0 | 0 | 0 | 2.40E-05 | 2.48E-05 | 0 |
| d__Bacteria;k__norank_d__Bacteria;p__Proteobacteria;c__Gammaproteobacteria;o__Aeromonadales;f__Aeromonadaceae;g__Aeromonas | 0.000288676 | 0 | 7.36E-05 | 0 | 0 | 0 | 5.23E-05 | 0 | 0 | 7.19E-05 | 0 | 2.57E-05 |
| d__Bacteria;k__norank_d__Bacteria;p__Myxococcota;c__Polyangia;o__Nannocystales;f__Nannocystaceae;g__norank_f__Nannocystaceae | 5.25E-05 | 0 | 0.000122612 | 0 | 2.26E-05 | 8.84E-05 | 0 | 2.59E-05 | 0 | 9.58E-05 | 7.43E-05 | 2.57E-05 |
| d__Bacteria;k__norank_d__Bacteria;p__Myxococcota;c__Polyangia;o__Polyangiales;f__unclassified_o__Polyangiales;g__unclassified_o__Polyangiales | 0.000209946 | 5.13E-05 | 7.36E-05 | 4.29E-05 | 0 | 0 | 0 | 0 | 5.12E-05 | 0 | 0 | 7.72E-05 |
| d__Bacteria;k__norank_d__Bacteria;p__Patescibacteria;c__Gracilibacteria;o__Candidatus_Peregrinibacteria;f__norank_o__Candidatus_Peregrinibacteria;g__norank_f__norank_o__Candidatus_Peregrinibacteria | 0 | 2.57E-05 | 0 | 2.14E-05 | 0.000113173 | 0 | 0 | 0 | 0 | 0.000167705 | 9.91E-05 | 7.72E-05 |
| d__Bacteria;k__norank_d__Bacteria;p__Proteobacteria;c__Alphaproteobacteria;o__Caulobacterales;f__Hyphomonadaceae;g__norank_f__Hyphomonadaceae | 0 | 5.13E-05 | 4.90E-05 | 0 | 4.53E-05 | 0.000176842 | 0.000104531 | 2.59E-05 | 2.56E-05 | 0 | 2.48E-05 | 0 |
| d__Bacteria;k__norank_d__Bacteria;p__Proteobacteria;c__Alphaproteobacteria;o__Rhodobacterales;f__Rhodobacteraceae;g__Flavimaricola | 0.000131216 | 0.000102627 | 7.36E-05 | 0 | 4.53E-05 | 2.21E-05 | 2.61E-05 | 2.59E-05 | 0 | 0 | 2.48E-05 | 5.15E-05 |
| d__Bacteria;k__norank_d__Bacteria;p__Planctomycetota;c__BD7-11;o__norank_c__BD7-11;f__norank_o__norank_c__BD7-11;g__norank_f__norank_o__norank_c__BD7-11 | 0 | 2.57E-05 | 2.45E-05 | 6.43E-05 | 9.05E-05 | 0 | 0 | 2.59E-05 | 0.000102404 | 0.000119789 | 4.95E-05 | 0 |
| d__Bacteria;k__norank_d__Bacteria;p__Proteobacteria;c__Alphaproteobacteria;o__Rhizobiales;f__Devosiaceae;g__Pelagibacterium | 0.00015746 | 0 | 7.36E-05 | 6.43E-05 | 0 | 2.21E-05 | 0.00018293 | 0 | 0 | 0 | 0 | 0 |
| d__Bacteria;k__norank_d__Bacteria;p__Acidobacteriota;c__Vicinamibacteria;o__Vicinamibacterales;f__Vicinamibacteraceae;g__unclassified_f__Vicinamibacteraceae | 0 | 2.57E-05 | 0 | 0 | 4.53E-05 | 4.42E-05 | 0 | 7.78E-05 | 7.68E-05 | 4.79E-05 | 2.48E-05 | 0.00015442 |
| d__Bacteria;k__norank_d__Bacteria;p__Firmicutes;c__Clostridia;o__Oscillospirales;f__UCG-010;g__norank_f__UCG-010 | 5.25E-05 | 5.13E-05 | 9.81E-05 | 0 | 0 | 6.63E-05 | 0 | 0.000129651 | 0 | 7.19E-05 | 0 | 2.57E-05 |
| d__Bacteria;k__norank_d__Bacteria;p__Bacteroidota;c__Bacteroidia;o__Sphingobacteriales;f__Sphingobacteriaceae;g__norank_f__Sphingobacteriaceae | 2.62E-05 | 5.13E-05 | 2.45E-05 | 0 | 6.79E-05 | 6.63E-05 | 0 | 0 | 0.000204808 | 0 | 4.95E-05 | 0 |
| d__Bacteria;k__norank_d__Bacteria;p__Proteobacteria;c__Gammaproteobacteria;o__Burkholderiales;f__Gallionellaceae;g__norank_f__Gallionellaceae | 7.87E-05 | 0.000128284 | 7.36E-05 | 0 | 0 | 0 | 0 | 0.000103721 | 0.000102404 | 0 | 0 | 0 |
| d__Bacteria;k__norank_d__Bacteria;p__Proteobacteria;c__Gammaproteobacteria;o__Diplorickettsiales;f__Diplorickettsiaceae;g__Rickettsiella | 5.25E-05 | 0 | 2.45E-05 | 4.29E-05 | 2.26E-05 | 0.000110527 | 5.23E-05 | 5.19E-05 | 0.000128005 | 0 | 0 | 0 |
| d__Bacteria;k__norank_d__Bacteria;p__Proteobacteria;c__Gammaproteobacteria;o__Enterobacterales;f__Yersiniaceae;g__unclassified_f__Yersiniaceae | 0.000236189 | 7.70E-05 | 0.000147135 | 0 | 2.26E-05 | 0 | 0 | 0 | 0 | 0 | 0 | 0 |
| d__Bacteria;k__norank_d__Bacteria;p__Actinobacteriota;c__Actinobacteria;o__Micromonosporales;f__Micromonosporaceae;g__Hamadaea | 5.25E-05 | 0 | 0 | 0 | 4.53E-05 | 0 | 0.000104531 | 0.000181512 | 2.56E-05 | 4.79E-05 | 2.48E-05 | 0 |
| d__Bacteria;k__norank_d__Bacteria;p__Actinobacteriota;c__Actinobacteria;o__Micrococcales;f__Intrasporangiaceae;g__Lapillicoccus | 2.62E-05 | 0 | 0 | 4.29E-05 | 4.53E-05 | 0.000110527 | 0 | 7.78E-05 | 2.56E-05 | 0 | 4.95E-05 | 0.000102947 |
| d__Bacteria;k__norank_d__Bacteria;p__Firmicutes;c__Clostridia;o__Lachnospirales;f__Lachnospiraceae;g__Lachnotalea | 0 | 5.13E-05 | 2.45E-05 | 2.14E-05 | 4.53E-05 | 0 | 7.84E-05 | 0.000207442 | 5.12E-05 | 0 | 0 | 0 |
| d__Bacteria;k__norank_d__Bacteria;p__Bacteroidota;c__Bacteroidia;o__Sphingobacteriales;f__Sphingobacteriaceae;g__Arcticibacter | 7.87E-05 | 0.000179598 | 0.000147135 | 0 | 2.26E-05 | 0 | 0 | 0 | 0 | 2.40E-05 | 0 | 2.57E-05 |
| d__Bacteria;k__norank_d__Bacteria;p__Bacteroidota;c__Bacteroidia;o__Bacteroidales;f__SB-5;g__norank_f__SB-5 | 0.000236189 | 0 | 0.000196179 | 0 | 0 | 4.42E-05 | 0 | 0 | 0 | 0 | 0 | 0 |
| d__Bacteria;k__norank_d__Bacteria;p__Proteobacteria;c__Gammaproteobacteria;o__Burkholderiales;f__Burkholderiaceae;g__Limnobacter | 2.62E-05 | 2.57E-05 | 0 | 2.14E-05 | 0.000113173 | 0.000287369 | 0 | 0 | 0 | 0 | 0 | 0 |
| d__Bacteria;k__norank_d__Bacteria;p__Proteobacteria;c__Alphaproteobacteria;o__Defluviicoccales;f__norank_o__Defluviicoccales;g__norank_f__norank_o__Defluviicoccales | 5.25E-05 | 0 | 7.36E-05 | 0 | 0 | 2.21E-05 | 0 | 0 | 0 | 7.19E-05 | 0.0001734 | 7.72E-05 |
| d__Bacteria;k__norank_d__Bacteria;p__Bacteroidota;c__Bacteroidia;o__Cytophagales;f__Cytophagaceae;g__Rhodocytophaga | 7.87E-05 | 0 | 2.45E-05 | 0 | 0.000113173 | 0.000176842 | 0 | 0 | 0 | 4.79E-05 | 2.48E-05 | 0 |
| d__Bacteria;k__norank_d__Bacteria;p__Patescibacteria;c__ABY1;o__unclassified_c__ABY1;f__unclassified_c__ABY1;g__unclassified_c__ABY1 | 2.62E-05 | 5.13E-05 | 0.000122612 | 0 | 0 | 4.42E-05 | 0 | 0 | 0 | 7.19E-05 | 0.000123857 | 2.57E-05 |
| d__Bacteria;k__norank_d__Bacteria;p__Firmicutes;c__BRH-c20a;o__norank_c__BRH-c20a;f__norank_o__norank_c__BRH-c20a;g__norank_f__norank_o__norank_c__BRH-c20a | 2.62E-05 | 0.000128284 | 4.90E-05 | 0.000128656 | 0 | 0 | 5.23E-05 | 5.19E-05 | 2.56E-05 | 0 | 0 | 0 |
| d__Bacteria;k__norank_d__Bacteria;p__Actinobacteriota;c__Thermoleophilia;o__Solirubrobacterales;f__Solirubrobacteraceae;g__unclassified_f__Solirubrobacteraceae | 5.25E-05 | 0 | 0 | 2.14E-05 | 0 | 0.000154737 | 5.23E-05 | 0.000103721 | 7.68E-05 | 0 | 0 | 0 |
| d__Bacteria;k__norank_d__Bacteria;p__Cyanobacteria;c__Cyanobacteriia;o__Oxyphotobacteria_Incertae_Sedis;f__unclassified_o__Oxyphotobacteria_Incertae_Sedis;g__EcFYyy-200 | 0 | 0 | 0 | 0 | 9.05E-05 | 0 | 0 | 0 | 0 | 0.000143747 | 0.000148629 | 7.72E-05 |
| d__Bacteria;k__norank_d__Bacteria;p__Actinobacteriota;c__Actinobacteria;o__Kineosporiales;f__Kineosporiaceae;g__unclassified_f__Kineosporiaceae | 5.25E-05 | 7.70E-05 | 4.90E-05 | 0 | 0 | 2.21E-05 | 2.61E-05 | 0.000129651 | 0.000102404 | 0 | 0 | 0 |
| d__Bacteria;k__norank_d__Bacteria;p__Proteobacteria;c__Gammaproteobacteria;o__Burkholderiales;f__Chromobacteriaceae;g__Pseudogulbenkiania | 7.87E-05 | 0.000128284 | 4.90E-05 | 0 | 0 | 0 | 2.61E-05 | 0 | 0 | 9.58E-05 | 7.43E-05 | 0 |
| d__Bacteria;k__norank_d__Bacteria;p__Planctomycetota;c__unclassified_p__Planctomycetota;o__unclassified_p__Planctomycetota;f__unclassified_p__Planctomycetota;g__unclassified_p__Planctomycetota | 0 | 5.13E-05 | 7.36E-05 | 0 | 0 | 4.42E-05 | 0 | 0 | 0 | 0 | 4.95E-05 | 0.00023163 |
| d__Bacteria;k__norank_d__Bacteria;p__Patescibacteria;c__ABY1;o__Candidatus_Kuenenbacteria;f__norank_o__Candidatus_Kuenenbacteria;g__norank_f__norank_o__Candidatus_Kuenenbacteria | 7.87E-05 | 0.000102627 | 2.45E-05 | 4.29E-05 | 9.05E-05 | 0.000110527 | 0 | 0 | 0 | 0 | 0 | 0 |
| d__Bacteria;k__norank_d__Bacteria;p__Bacteroidota;c__Bacteroidia;o__Sphingobacteriales;f__Lentimicrobiaceae;g__unclassified_f__Lentimicrobiaceae | 2.62E-05 | 7.70E-05 | 2.45E-05 | 0 | 0.000113173 | 0 | 0.000130664 | 7.78E-05 | 0 | 0 | 0 | 0 |
| d__Bacteria;k__norank_d__Bacteria;p__Proteobacteria;c__Gammaproteobacteria;o__Burkholderiales;f__Comamonadaceae;g__Rhodoferax | 7.87E-05 | 2.57E-05 | 7.36E-05 | 0 | 9.05E-05 | 2.21E-05 | 0 | 5.19E-05 | 5.12E-05 | 0 | 0 | 5.15E-05 |
| d__Bacteria;k__norank_d__Bacteria;p__Abditibacteriota;c__Abditibacteria;o__Abditibacteriales;f__Abditibacteriaceae;g__Abditibacterium | 0 | 5.13E-05 | 0.000122612 | 0 | 9.05E-05 | 0 | 7.84E-05 | 0 | 2.56E-05 | 0 | 7.43E-05 | 0 |
| d__Bacteria;k__norank_d__Bacteria;p__Planctomycetota;c__Planctomycetes;o__Isosphaerales;f__Isosphaeraceae;g__Tundrisphaera | 5.25E-05 | 2.57E-05 | 2.45E-05 | 0.000171541 | 4.53E-05 | 2.21E-05 | 2.61E-05 | 0 | 2.56E-05 | 2.40E-05 | 2.48E-05 | 0 |
| d__Bacteria;k__norank_d__Bacteria;p__Firmicutes;c__Bacilli;o__Bacillales;f__Bacillaceae;g__Caldibacillus | 0 | 0 | 0 | 0 | 0 | 0 | 0.000130664 | 0.000207442 | 7.68E-05 | 0 | 2.48E-05 | 0 |
| d__Bacteria;k__norank_d__Bacteria;p__Patescibacteria;c__Microgenomatia;o__Candidatus_Pacebacteria;f__norank_o__Candidatus_Pacebacteria;g__norank_f__norank_o__Candidatus_Pacebacteria | 0 | 5.13E-05 | 7.36E-05 | 0 | 6.79E-05 | 4.42E-05 | 7.84E-05 | 0 | 0 | 2.40E-05 | 7.43E-05 | 2.57E-05 |
| d__Bacteria;k__norank_d__Bacteria;p__Fibrobacterota;c__Fibrobacteria;o__Fibrobacterales;f__norank_o__Fibrobacterales;g__BBMC-4 | 7.87E-05 | 0.000128284 | 9.81E-05 | 4.29E-05 | 9.05E-05 | 0 | 0 | 0 | 0 | 0 | 0 | 0 |
| d__Bacteria;k__norank_d__Bacteria;p__Proteobacteria;c__Alphaproteobacteria;o__Rhodobacterales;f__Rhodobacteraceae;g__Tabrizicola | 0.000131216 | 2.57E-05 | 2.45E-05 | 0 | 4.53E-05 | 0 | 0 | 0.000155581 | 5.12E-05 | 0 | 0 | 0 |
| d__Bacteria;k__norank_d__Bacteria;p__Proteobacteria;c__Alphaproteobacteria;o__Rhodobacterales;f__Rhodobacteraceae;g__Defluviimonas | 2.62E-05 | 5.13E-05 | 0 | 2.14E-05 | 9.05E-05 | 4.42E-05 | 0 | 0 | 0 | 2.40E-05 | 0.000123857 | 5.15E-05 |
| d__Bacteria;k__norank_d__Bacteria;p__Chloroflexi;c__Chloroflexia;o__Thermomicrobiales;f__Thermomicrobiaceae;g__Sphaerobacter | 0 | 0.000102627 | 0 | 2.14E-05 | 0 | 0 | 0 | 0.000129651 | 0.000102404 | 0 | 4.95E-05 | 2.57E-05 |
| d__Bacteria;k__norank_d__Bacteria;p__Firmicutes;c__Bacilli;o__Bacillales;f__Planococcaceae;g__norank_f__Planococcaceae | 0.000131216 | 0 | 0 | 4.29E-05 | 0.000113173 | 2.21E-05 | 0 | 0 | 0 | 7.19E-05 | 4.95E-05 | 0 |
| d__Bacteria;k__norank_d__Bacteria;p__Proteobacteria;c__Alphaproteobacteria;o__Rhodobacterales;f__Rhodobacteraceae;g__norank_f__Rhodobacteraceae | 0 | 2.57E-05 | 2.45E-05 | 6.43E-05 | 4.53E-05 | 0.000221053 | 0 | 0 | 0 | 4.79E-05 | 0 | 0 |
| d__Bacteria;k__norank_d__Bacteria;p__Chloroflexi;c__Ktedonobacteria;o__Ktedonobacterales;f__Ktedonobacteraceae;g__JG30a-KF-32 | 0.000131216 | 0.000102627 | 0.000122612 | 0 | 0 | 4.42E-05 | 0 | 0 | 2.56E-05 | 0 | 0 | 0 |
| d__Bacteria;k__norank_d__Bacteria;p__Actinobacteriota;c__Actinobacteria;o__Micromonosporales;f__Micromonosporaceae;g__Virgisporangium | 5.25E-05 | 0 | 0 | 4.29E-05 | 0 | 0 | 2.61E-05 | 0 | 5.12E-05 | 0.000143747 | 9.91E-05 | 0 |
| d__Bacteria;k__norank_d__Bacteria;p__Bacteroidota;c__Bacteroidia;o__Flavobacteriales;f__Weeksellaceae;g__Moheibacter | 0.000209946 | 2.57E-05 | 0 | 0 | 0 | 0 | 2.61E-05 | 0 | 0.000153606 | 0 | 0 | 0 |
| d__Bacteria;k__norank_d__Bacteria;p__Firmicutes;c__Bacilli;o__Thermoactinomycetales;f__Thermoactinomycetaceae;g__Pasteuria | 0.000104973 | 7.70E-05 | 0 | 8.58E-05 | 0 | 2.21E-05 | 2.61E-05 | 0 | 0 | 4.79E-05 | 0 | 5.15E-05 |
| d__Bacteria;k__norank_d__Bacteria;p__Chloroflexi;c__Anaerolineae;o__SBR1031;f__A4b;g__OLB15 | 0 | 0 | 2.45E-05 | 0 | 0 | 0 | 0.000104531 | 5.19E-05 | 0.000230409 | 0 | 0 | 0 |
| d__Bacteria;k__norank_d__Bacteria;p__Patescibacteria;c__ABY1;o__Candidatus_Kerfeldbacteria;f__norank_o__Candidatus_Kerfeldbacteria;g__norank_f__norank_o__Candidatus_Kerfeldbacteria | 0.00015746 | 2.57E-05 | 0 | 0 | 0 | 0 | 0 | 0 | 0 | 2.40E-05 | 0.0001734 | 2.57E-05 |
| d__Bacteria;k__norank_d__Bacteria;p__Verrucomicrobiota;c__Omnitrophia;o__Omnitrophales;f__norank_o__Omnitrophales;g__norank_f__norank_o__Omnitrophales | 2.62E-05 | 0.000230911 | 0.000147135 | 0 | 0 | 0 | 0 | 0 | 0 | 0 | 0 | 0 |
| d__Bacteria;k__norank_d__Bacteria;p__Chloroflexi;c__Dehalococcoidia;o__unclassified_c__Dehalococcoidia;f__unclassified_c__Dehalococcoidia;g__unclassified_c__Dehalococcoidia | 0.000104973 | 2.57E-05 | 4.90E-05 | 0 | 6.79E-05 | 0 | 0.000104531 | 2.59E-05 | 2.56E-05 | 0 | 0 | 0 |
| d__Bacteria;k__norank_d__Bacteria;p__Chloroflexi;c__NLS2-31;o__norank_c__NLS2-31;f__norank_o__norank_c__NLS2-31;g__norank_f__norank_o__norank_c__NLS2-31 | 0.000183703 | 7.70E-05 | 9.81E-05 | 2.14E-05 | 0 | 2.21E-05 | 0 | 0 | 0 | 0 | 0 | 0 |
| d__Bacteria;k__norank_d__Bacteria;p__Firmicutes;c__Bacilli;o__Entomoplasmatales;f__type_III;g__norank_f__type_III | 5.25E-05 | 7.70E-05 | 0 | 0 | 0 | 4.42E-05 | 2.61E-05 | 5.19E-05 | 0.000102404 | 4.79E-05 | 0 | 0 |
| d__Bacteria;k__norank_d__Bacteria;p__Chloroflexi;c__Dehalococcoidia;o__FW22;f__norank_o__FW22;g__norank_f__norank_o__FW22 | 0.000104973 | 0 | 0.000147135 | 2.14E-05 | 0 | 0 | 0 | 2.59E-05 | 0.000102404 | 0 | 0 | 0 |
| d__Bacteria;k__norank_d__Bacteria;p__Sumerlaeota;c__Sumerlaeia;o__norank_c__Sumerlaeia;f__norank_o__norank_c__Sumerlaeia;g__norank_f__norank_o__norank_c__Sumerlaeia | 5.25E-05 | 7.70E-05 | 0 | 0 | 4.53E-05 | 0 | 0 | 0 | 0 | 7.19E-05 | 4.95E-05 | 0.000102947 |
| d__Bacteria;k__norank_d__Bacteria;p__Proteobacteria;c__Alphaproteobacteria;o__Sphingomonadales;f__Sphingomonadaceae;g__Sphingoaurantiacus | 0 | 0 | 0 | 6.43E-05 | 0.000113173 | 4.42E-05 | 2.61E-05 | 0 | 7.68E-05 | 0 | 7.43E-05 | 0 |
| d__Bacteria;k__norank_d__Bacteria;p__Firmicutes;c__Clostridia;o__Clostridiales;f__Caloramatoraceae;g__unclassified_f__Caloramatoraceae | 7.87E-05 | 0.000153941 | 4.90E-05 | 4.29E-05 | 0 | 2.21E-05 | 0 | 5.19E-05 | 0 | 0 | 0 | 0 |
| d__Bacteria;k__norank_d__Bacteria;p__Patescibacteria;c__Microgenomatia;o__Candidatus_Woesebacteria;f__norank_o__Candidatus_Woesebacteria;g__norank_f__norank_o__Candidatus_Woesebacteria | 5.25E-05 | 7.70E-05 | 9.81E-05 | 2.14E-05 | 0 | 0 | 0 | 0 | 0 | 7.19E-05 | 7.43E-05 | 0 |
| d__Bacteria;k__norank_d__Bacteria;p__Actinobacteriota;c__Thermoleophilia;o__Solirubrobacterales;f__Solirubrobacteraceae;g__Patulibacter | 0 | 0.000102627 | 2.45E-05 | 0 | 2.26E-05 | 8.84E-05 | 2.61E-05 | 0 | 5.12E-05 | 2.40E-05 | 0 | 5.15E-05 |
| d__Bacteria;k__norank_d__Bacteria;p__Firmicutes;c__Bacilli;o__Bacillales;f__Bacillaceae;g__Pseudogracilibacillus | 0 | 7.70E-05 | 0 | 8.58E-05 | 6.79E-05 | 0 | 0 | 0.000129651 | 0 | 2.40E-05 | 0 | 0 |
| d__Bacteria;k__norank_d__Bacteria;p__Bacteroidota;c__Bacteroidia;o__Chitinophagales;f__Chitinophagaceae;g__Sediminibacterium | 2.62E-05 | 2.57E-05 | 4.90E-05 | 0 | 0 | 0 | 0.000104531 | 2.59E-05 | 7.68E-05 | 2.40E-05 | 0 | 5.15E-05 |
| d__Bacteria;k__norank_d__Bacteria;p__Proteobacteria;c__Gammaproteobacteria;o__Salinisphaerales;f__Solimonadaceae;g__norank_f__Solimonadaceae | 2.62E-05 | 0 | 2.45E-05 | 0 | 2.26E-05 | 8.84E-05 | 0 | 0 | 0 | 7.19E-05 | 0.000123857 | 2.57E-05 |
| d__Bacteria;k__norank_d__Bacteria;p__Bacteroidota;c__Bacteroidia;o__Bacteroidales;f__Paludibacteraceae;g__norank_f__Paludibacteraceae | 0.000209946 | 0 | 0.000171657 | 0 | 0 | 0 | 0 | 0 | 0 | 0 | 0 | 0 |
| d__Bacteria;k__norank_d__Bacteria;p__Firmicutes;c__Bacilli;o__Bacillales;f__Salisediminibacteriaceae;g__Salipaludibacillus | 5.25E-05 | 0.000128284 | 9.81E-05 | 2.14E-05 | 0 | 0 | 0 | 0 | 0 | 0 | 0 | 7.72E-05 |
| d__Bacteria;k__norank_d__Bacteria;p__Fibrobacterota;c__Fibrobacteria;o__Fibrobacterales;f__Fibrobacteraceae;g__unclassified_f__Fibrobacteraceae | 7.87E-05 | 5.13E-05 | 2.45E-05 | 6.43E-05 | 0.000135808 | 2.21E-05 | 0 | 0 | 0 | 0 | 0 | 0 |
| d__Bacteria;k__norank_d__Bacteria;p__Patescibacteria;c__Microgenomatia;o__Candidatus_Levybacteria;f__norank_o__Candidatus_Levybacteria;g__norank_f__norank_o__Candidatus_Levybacteria | 0 | 0 | 2.45E-05 | 0.000128656 | 0 | 0 | 2.61E-05 | 0 | 0 | 0.000119789 | 0 | 7.72E-05 |
| d__Bacteria;k__norank_d__Bacteria;p__Proteobacteria;c__Gammaproteobacteria;o__Xanthomonadales;f__Rhodanobacteraceae;g__Pseudofulvimonas | 0 | 2.57E-05 | 0 | 0.000235869 | 0 | 8.84E-05 | 0 | 0 | 0 | 0 | 0 | 2.57E-05 |
| d__Bacteria;k__norank_d__Bacteria;p__Desulfobacterota;c__Desulfobulbia;o__Desulfobulbales;f__Desulfurivibrionaceae;g__Desulfurivibrio | 0.000183703 | 0.000153941 | 2.45E-05 | 0 | 0 | 0 | 0 | 0 | 0 | 0 | 0 | 0 |
| d__Bacteria;k__norank_d__Bacteria;p__Firmicutes;c__Clostridia;o__Eubacteriales;f__Alkalibacteraceae;g__Alkalibacter | 7.87E-05 | 5.13E-05 | 2.45E-05 | 2.14E-05 | 2.26E-05 | 0.000110527 | 2.61E-05 | 2.59E-05 | 0 | 0 | 0 | 0 |
| d__Bacteria;k__norank_d__Bacteria;p__Acidobacteriota;c__Subgroup_21;o__norank_c__Subgroup_21;f__norank_o__norank_c__Subgroup_21;g__norank_f__norank_o__norank_c__Subgroup_21 | 0.000104973 | 2.57E-05 | 0 | 0 | 0 | 0.000154737 | 2.61E-05 | 0 | 0 | 2.40E-05 | 0 | 2.57E-05 |
| d__Bacteria;k__norank_d__Bacteria;p__Actinobacteriota;c__Actinobacteria;o__Catenulisporales;f__Actinospicaceae;g__Actinospica | 0 | 0 | 0 | 0 | 0.000135808 | 2.21E-05 | 2.61E-05 | 5.19E-05 | 2.56E-05 | 4.79E-05 | 2.48E-05 | 2.57E-05 |
| d__Bacteria;k__norank_d__Bacteria;p__Cyanobacteria;c__Cyanobacteriia;o__Cyanobacteriales;f__norank_o__Cyanobacteriales;g__norank_f__norank_o__Cyanobacteriales | 0 | 0 | 0 | 2.14E-05 | 0.000226347 | 0.000110527 | 0 | 0 | 0 | 0 | 0 | 0 |
| d__Bacteria;k__norank_d__Bacteria;p__Desulfobacterota;c__Syntrophorhabdia;o__Syntrophorhabdales;f__Syntrophorhabdaceae;g__Syntrophorhabdus | 0.000209946 | 2.57E-05 | 7.36E-05 | 0 | 4.53E-05 | 0 | 0 | 0 | 0 | 0 | 0 | 0 |
| d__Bacteria;k__norank_d__Bacteria;p__Proteobacteria;c__Alphaproteobacteria;o__Caulobacterales;f__Parvularculaceae;g__Amphiplicatus | 0 | 2.57E-05 | 0.000147135 | 8.58E-05 | 0 | 6.63E-05 | 0 | 2.59E-05 | 0 | 0 | 0 | 0 |
| d__Bacteria;k__norank_d__Bacteria;p__Cyanobacteria;c__Cyanobacteriia;o__Leptolyngbyales;f__Leptolyngbyaceae;g__norank_f__Leptolyngbyaceae | 0 | 0 | 0 | 0 | 0 | 0 | 0 | 0 | 0 | 0 | 0.000346801 | 0 |
| d__Bacteria;k__norank_d__Bacteria;p__Firmicutes;c__Clostridia;o__norank_c__Clostridia;f__Hungateiclostridiaceae;g__Pseudobacteroides | 7.87E-05 | 0 | 7.36E-05 | 0 | 0 | 6.63E-05 | 2.61E-05 | 5.19E-05 | 2.56E-05 | 2.40E-05 | 0 | 0 |
| d__Bacteria;k__norank_d__Bacteria;p__Patescibacteria;c__ABY1;o__Candidatus_Komeilibacteria;f__norank_o__Candidatus_Komeilibacteria;g__norank_f__norank_o__Candidatus_Komeilibacteria | 2.62E-05 | 0 | 0.000147135 | 0 | 2.26E-05 | 0 | 0 | 0 | 0 | 0 | 0.000123857 | 2.57E-05 |
| d__Bacteria;k__norank_d__Bacteria;p__Halanaerobiaeota;c__Halanaerobiia;o__Halanaerobiales;f__Halanaerobiaceae;g__Halocella | 0.000104973 | 0 | 0 | 0 | 0.000113173 | 2.21E-05 | 0 | 0 | 0 | 0 | 0 | 0.000102947 |
| d__Bacteria;k__norank_d__Bacteria;p__Verrucomicrobiota;c__Verrucomicrobiae;o__Pedosphaerales;f__Pedosphaeraceae;g__Pedosphaera | 2.62E-05 | 2.57E-05 | 0.000245224 | 0 | 4.53E-05 | 0 | 0 | 0 | 0 | 0 | 0 | 0 |
| d__Bacteria;k__norank_d__Bacteria;p__Actinobacteriota;c__Actinobacteria;o__Micrococcales;f__Microbacteriaceae;g__Gryllotalpicola | 0 | 0 | 0 | 0 | 0 | 0 | 0.000156797 | 0.000129651 | 5.12E-05 | 0 | 0 | 0 |
| d__Bacteria;k__norank_d__Bacteria;p__Proteobacteria;c__Alphaproteobacteria;o__Micavibrionales;f__Micavibrionaceae;g__norank_f__Micavibrionaceae | 2.62E-05 | 0 | 0 | 6.43E-05 | 2.26E-05 | 0 | 0 | 0 | 2.56E-05 | 4.79E-05 | 9.91E-05 | 5.15E-05 |
| d__Bacteria;k__norank_d__Bacteria;p__Actinobacteriota;c__Actinobacteria;o__Micromonosporales;f__Micromonosporaceae;g__Stackebrandtia | 0 | 0 | 0 | 0 | 0 | 0 | 7.84E-05 | 2.59E-05 | 0.000230409 | 0 | 0 | 0 |
| d__Bacteria;k__norank_d__Bacteria;p__Bacteroidota;c__Bacteroidia;o__Cytophagales;f__Spirosomaceae;g__Emticicia | 0 | 0 | 0 | 0 | 0 | 0 | 2.61E-05 | 5.19E-05 | 0.000230409 | 0 | 0 | 2.57E-05 |
| d__Bacteria;k__norank_d__Bacteria;p__Bacteroidota;c__Bacteroidia;o__Chitinophagales;f__Chitinophagaceae;g__Filimonas | 0 | 7.70E-05 | 4.90E-05 | 0 | 0 | 0 | 5.23E-05 | 0.000129651 | 2.56E-05 | 0 | 0 | 0 |
| d__Bacteria;k__norank_d__Bacteria;p__Actinobacteriota;c__Actinobacteria;o__Propionibacteriales;f__Nocardioidaceae;g__Aeromicrobium | 0 | 0.000128284 | 0 | 2.14E-05 | 9.05E-05 | 6.63E-05 | 2.61E-05 | 0 | 0 | 0 | 0 | 0 |
| d__Bacteria;k__norank_d__Bacteria;p__Cyanobacteria;c__Cyanobacteriia;o__Cyanobacteriales;f__Coleofasciculaceae;g__norank_f__Coleofasciculaceae | 0 | 0.000307882 | 2.45E-05 | 0 | 0 | 0 | 0 | 0 | 0 | 0 | 0 | 0 |
| d__Bacteria;k__norank_d__Bacteria;p__Proteobacteria;c__Gammaproteobacteria;o__Burkholderiales;f__Methylophilaceae;g__unclassified_f__Methylophilaceae | 5.25E-05 | 7.70E-05 | 2.45E-05 | 0 | 0 | 0 | 5.23E-05 | 2.59E-05 | 2.56E-05 | 2.40E-05 | 2.48E-05 | 2.57E-05 |
| d__Bacteria;k__norank_d__Bacteria;p__Firmicutes;c__Clostridia;o__Clostridiales;f__norank_o__Clostridiales;g__norank_f__norank_o__Clostridiales | 0 | 0 | 0 | 0 | 2.26E-05 | 0 | 0 | 0.000285233 | 0 | 2.40E-05 | 0 | 0 |
| d__Bacteria;k__norank_d__Bacteria;p__Proteobacteria;c__Alphaproteobacteria;o__Elsterales;f__Elsteraceae;g__norank_f__Elsteraceae | 0 | 0 | 4.90E-05 | 0 | 2.26E-05 | 0 | 7.84E-05 | 0.000181512 | 0 | 0 | 0 | 0 |
| d__Bacteria;k__norank_d__Bacteria;p__Proteobacteria;c__Gammaproteobacteria;o__Pseudomonadales;f__Pseudomonadaceae;g__Azotobacter | 5.25E-05 | 7.70E-05 | 7.36E-05 | 0 | 0 | 0 | 0 | 2.59E-05 | 7.68E-05 | 0 | 0 | 2.57E-05 |
| d__Bacteria;k__norank_d__Bacteria;p__Firmicutes;c__Clostridia;o__Peptostreptococcales-Tissierellales;f__Proteiniboraceae;g__Proteiniborus | 5.25E-05 | 5.13E-05 | 2.45E-05 | 0 | 0 | 0 | 0 | 0 | 5.12E-05 | 0 | 9.91E-05 | 5.15E-05 |
| d__Bacteria;k__norank_d__Bacteria;p__Proteobacteria;c__Alphaproteobacteria;o__Rhizobiales;f__Methylopilaceae;g__Methylopila | 2.62E-05 | 0 | 0 | 0 | 0 | 4.42E-05 | 5.23E-05 | 7.78E-05 | 7.68E-05 | 0 | 4.95E-05 | 0 |
| d__Bacteria;k__norank_d__Bacteria;p__Proteobacteria;c__Gammaproteobacteria;o__Burkholderiales;f__Comamonadaceae;g__Pseudorhodoferax | 7.87E-05 | 0 | 0 | 4.29E-05 | 0.000113173 | 6.63E-05 | 0 | 0 | 2.56E-05 | 0 | 0 | 0 |
| d__Bacteria;k__norank_d__Bacteria;p__Verrucomicrobiota;c__Omnitrophia;o__Omnitrophales;f__Omnitrophaceae;g__Candidatus_Omnitrophus | 0.000104973 | 0 | 2.45E-05 | 0 | 4.53E-05 | 0 | 0 | 0 | 0 | 2.40E-05 | 2.48E-05 | 0.000102947 |
| d__Bacteria;k__norank_d__Bacteria;p__Firmicutes;c__Desulfotomaculia;o__Desulfotomaculales;f__norank_o__Desulfotomaculales;g__Cryptanaerobacter | 5.25E-05 | 5.13E-05 | 0 | 2.14E-05 | 4.53E-05 | 0 | 0.000104531 | 0 | 0 | 0 | 2.48E-05 | 2.57E-05 |
| d__Bacteria;k__norank_d__Bacteria;p__Acidobacteriota;c__Blastocatellia;o__unclassified_c__Blastocatellia;f__unclassified_c__Blastocatellia;g__unclassified_c__Blastocatellia | 0 | 0 | 0 | 0 | 0 | 0 | 0 | 0 | 5.12E-05 | 9.58E-05 | 4.95E-05 | 0.000128684 |
| d__Bacteria;k__norank_d__Bacteria;p__Proteobacteria;c__Alphaproteobacteria;o__Parvibaculales;f__Parvibaculaceae;g__Parvibaculum | 2.62E-05 | 5.13E-05 | 2.45E-05 | 0 | 2.26E-05 | 0 | 0 | 0 | 0 | 4.79E-05 | 9.91E-05 | 5.15E-05 |
| d__Bacteria;k__norank_d__Bacteria;p__Chloroflexi;c__Anaerolineae;o__SBR1031;f__A4b;g__unclassified_f__A4b | 0 | 5.13E-05 | 0 | 6.43E-05 | 0.000113173 | 4.42E-05 | 2.61E-05 | 0 | 0 | 2.40E-05 | 0 | 0 |
| d__Bacteria;k__norank_d__Bacteria;p__Planctomycetota;c__vadinHA49;o__norank_c__vadinHA49;f__norank_o__norank_c__vadinHA49;g__norank_f__norank_o__norank_c__vadinHA49 | 7.87E-05 | 5.13E-05 | 2.45E-05 | 4.29E-05 | 0 | 0 | 0 | 0 | 0 | 4.79E-05 | 4.95E-05 | 2.57E-05 |
| d__Bacteria;k__norank_d__Bacteria;p__Desulfobacterota;c__Desulfobulbia;o__Desulfobulbales;f__Desulfocapsaceae;g__Desulfobacterium_catecholicum_group | 7.87E-05 | 2.57E-05 | 4.90E-05 | 0 | 2.26E-05 | 6.63E-05 | 2.61E-05 | 2.59E-05 | 2.56E-05 | 0 | 0 | 0 |
| d__Bacteria;k__norank_d__Bacteria;p__Proteobacteria;c__Alphaproteobacteria;o__Rhizobiales;f__Rhizobiaceae;g__Nitratireductor | 2.62E-05 | 0 | 0 | 0 | 0.000181077 | 0.000110527 | 0 | 0 | 0 | 0 | 0 | 0 |
| d__Bacteria;k__norank_d__Bacteria;p__Firmicutes;c__Clostridia;o__Christensenellales;f__Christensenellaceae;g__Christensenellaceae_R-7_group | 7.87E-05 | 2.57E-05 | 7.36E-05 | 4.29E-05 | 0 | 2.21E-05 | 2.61E-05 | 0 | 0 | 2.40E-05 | 2.48E-05 | 0 |
| d__Bacteria;k__norank_d__Bacteria;p__Cyanobacteria;c__Cyanobacteriia;o__Oxyphotobacteria_Incertae_Sedis;f__unclassified_o__Oxyphotobacteria_Incertae_Sedis;g__norank_f__unclassified_o__Oxyphotobacteria_Incertae_Sedis | 2.62E-05 | 0 | 0 | 0 | 0 | 8.84E-05 | 0 | 0 | 0 | 0 | 0.000148629 | 5.15E-05 |
| d__Bacteria;k__norank_d__Bacteria;p__Acidobacteriota;c__Subgroup_19;o__norank_c__Subgroup_19;f__norank_o__norank_c__Subgroup_19;g__norank_f__norank_o__norank_c__Subgroup_19 | 0 | 0 | 0 | 0 | 4.53E-05 | 6.63E-05 | 0 | 0 | 5.12E-05 | 2.40E-05 | 4.95E-05 | 7.72E-05 |
| d__Bacteria;k__norank_d__Bacteria;p__Sva0485;c__norank_p__Sva0485;o__norank_c__norank_p__Sva0485;f__norank_o__norank_c__norank_p__Sva0485;g__norank_f__norank_o__norank_c__norank_p__Sva0485 | 5.25E-05 | 0 | 2.45E-05 | 2.14E-05 | 6.79E-05 | 0 | 2.61E-05 | 0 | 0 | 9.58E-05 | 2.48E-05 | 0 |
| d__Bacteria;k__norank_d__Bacteria;p__Proteobacteria;c__Alphaproteobacteria;o__Rhodobacterales;f__Rhodobacteraceae;g__Roseobacter_clade_CHAB-I-5_lineage | 2.62E-05 | 5.13E-05 | 0 | 4.29E-05 | 0 | 8.84E-05 | 2.61E-05 | 7.78E-05 | 0 | 0 | 0 | 0 |
| d__Bacteria;k__norank_d__Bacteria;p__Gemmatimonadota;c__Longimicrobia;o__Longimicrobiales;f__Longimicrobiaceae;g__Longimicrobium | 0 | 0 | 0 | 0 | 0 | 0 | 0.000104531 | 0.000103721 | 0.000102404 | 0 | 0 | 0 |
| d__Bacteria;k__norank_d__Bacteria;p__Proteobacteria;c__Alphaproteobacteria;o__Kiloniellales;f__Fodinicurvataceae;g__norank_f__Fodinicurvataceae | 0 | 2.57E-05 | 4.90E-05 | 0 | 4.53E-05 | 8.84E-05 | 0 | 0 | 7.68E-05 | 0 | 2.48E-05 | 0 |
| d__Bacteria;k__norank_d__Bacteria;p__Firmicutes;c__Clostridia;o__norank_c__Clostridia;f__norank_o__norank_c__Clostridia;g__norank_f__norank_o__norank_c__Clostridia | 2.62E-05 | 2.57E-05 | 2.45E-05 | 0 | 0 | 0.000132632 | 0 | 0 | 5.12E-05 | 0 | 4.95E-05 | 0 |
| d__Bacteria;k__norank_d__Bacteria;p__Proteobacteria;c__Alphaproteobacteria;o__Paracaedibacterales;f__Paracaedibacteraceae;g__norank_f__Paracaedibacteraceae | 0.000104973 | 7.70E-05 | 2.45E-05 | 0 | 0 | 0 | 5.23E-05 | 0 | 0 | 0 | 4.95E-05 | 0 |
| d__Bacteria;k__norank_d__Bacteria;p__Firmicutes;c__Clostridia;o__Clostridiales;f__Clostridiaceae;g__Clostridium_sensu_stricto_19 | 7.87E-05 | 2.57E-05 | 2.45E-05 | 0.000107213 | 0 | 4.42E-05 | 0 | 2.59E-05 | 0 | 0 | 0 | 0 |
| d__Bacteria;k__norank_d__Bacteria;p__Chloroflexi;c__Anaerolineae;o__C10-SB1A;f__norank_o__C10-SB1A;g__norank_f__norank_o__C10-SB1A | 2.62E-05 | 0 | 0 | 6.43E-05 | 6.79E-05 | 4.42E-05 | 2.61E-05 | 0 | 0 | 0 | 4.95E-05 | 2.57E-05 |
| d__Bacteria;k__norank_d__Bacteria;p__Firmicutes;c__Clostridia;o__norank_c__Clostridia;f__unclassified_o__norank_c__Clostridia;g__unclassified_o__norank_c__Clostridia | 0 | 2.57E-05 | 4.90E-05 | 2.14E-05 | 0 | 0 | 2.61E-05 | 0.000181512 | 0 | 0 | 0 | 0 |
| d__Bacteria;k__norank_d__Bacteria;p__Firmicutes;c__Negativicutes;o__norank_c__Negativicutes;f__norank_o__norank_c__Negativicutes;g__norank_f__norank_o__norank_c__Negativicutes | 0 | 2.57E-05 | 0 | 4.29E-05 | 0 | 0 | 0.00018293 | 0 | 0 | 0 | 0 | 5.15E-05 |
| d__Bacteria;k__norank_d__Bacteria;p__Firmicutes;c__Clostridia;o__Clostridiales;f__Clostridiaceae;g__Clostridium_sensu_stricto_11 | 5.25E-05 | 0 | 4.90E-05 | 4.29E-05 | 0 | 0 | 5.23E-05 | 2.59E-05 | 7.68E-05 | 0 | 0 | 0 |
| d__Bacteria;k__norank_d__Bacteria;p__Cyanobacteria;c__Cyanobacteriia;o__Cyanobacteriales;f__Nostocaceae;g__Tolypothrix | 0 | 7.70E-05 | 4.90E-05 | 0 | 2.26E-05 | 2.21E-05 | 0 | 2.59E-05 | 2.56E-05 | 2.40E-05 | 0 | 5.15E-05 |
| d__Bacteria;k__norank_d__Bacteria;p__Planctomycetota;c__Planctomycetes;o__Gemmatales;f__Gemmataceae;g__unclassified_f__Gemmataceae | 0 | 0 | 4.90E-05 | 0 | 4.53E-05 | 0 | 0 | 5.19E-05 | 5.12E-05 | 2.40E-05 | 2.48E-05 | 5.15E-05 |
| d__Bacteria;k__norank_d__Bacteria;p__TX1A-33;c__norank_p__TX1A-33;o__norank_c__norank;f__norank_o__norank;g__norank_f__norank | 7.87E-05 | 2.57E-05 | 4.90E-05 | 0 | 9.05E-05 | 0 | 0 | 0 | 0 | 0 | 0 | 5.15E-05 |
| d__Bacteria;k__norank_d__Bacteria;p__Proteobacteria;c__Alphaproteobacteria;o__Azospirillales;f__Inquilinaceae;g__Inquilinus | 7.87E-05 | 5.13E-05 | 4.90E-05 | 0 | 4.53E-05 | 4.42E-05 | 0 | 2.59E-05 | 0 | 0 | 0 | 0 |
| d__Bacteria;k__norank_d__Bacteria;p__Bacteroidota;c__Bacteroidia;o__Cytophagales;f__Microscillaceae;g__OLB12 | 5.25E-05 | 0.000102627 | 4.90E-05 | 0 | 4.53E-05 | 4.42E-05 | 0 | 0 | 0 | 0 | 0 | 0 |
| d__Bacteria;k__norank_d__Bacteria;p__Chloroflexi;c__Anaerolineae;o__Anaerolineales;f__Anaerolineaceae;g__UTCFX1 | 0 | 2.57E-05 | 7.36E-05 | 0 | 0.000113173 | 0 | 7.84E-05 | 0 | 0 | 0 | 0 | 0 |
| d__Bacteria;k__norank_d__Bacteria;p__Actinobacteriota;c__Actinobacteria;o__Micromonosporales;f__Micromonosporaceae;g__Rhizocola | 0 | 0 | 0.000122612 | 4.29E-05 | 0 | 0 | 0 | 0 | 0 | 7.19E-05 | 0 | 5.15E-05 |
| d__Bacteria;k__norank_d__Bacteria;p__Proteobacteria;c__Alphaproteobacteria;o__Rhodobacterales;f__Rhodobacteraceae;g__Actibacterium | 0 | 5.13E-05 | 0 | 0.000107213 | 0 | 0 | 5.23E-05 | 5.19E-05 | 2.56E-05 | 0 | 0 | 0 |
| d__Bacteria;k__norank_d__Bacteria;p__Firmicutes;c__Desulfotomaculia;o__Desulfotomaculales;f__Desulfallas-Sporotomaculum;g__norank_f__Desulfallas-Sporotomaculum | 2.62E-05 | 0 | 9.81E-05 | 4.29E-05 | 4.53E-05 | 0 | 0 | 0 | 2.56E-05 | 2.40E-05 | 0 | 2.57E-05 |
| d__Bacteria;k__norank_d__Bacteria;p__Proteobacteria;c__Gammaproteobacteria;o__Methylococcales;f__Methylomonadaceae;g__unclassified_f__Methylomonadaceae | 2.62E-05 | 7.70E-05 | 4.90E-05 | 0.000107213 | 0 | 0 | 2.61E-05 | 0 | 0 | 0 | 0 | 0 |
| d__Bacteria;k__norank_d__Bacteria;p__Firmicutes;c__norank_p__Firmicutes;o__norank_c__norank_p__Firmicutes;f__norank_o__norank_c__norank_p__Firmicutes;g__norank_f__norank_o__norank_c__norank_p__Firmicutes | 0 | 0 | 0 | 4.29E-05 | 9.05E-05 | 2.21E-05 | 2.61E-05 | 5.19E-05 | 0 | 0 | 4.95E-05 | 0 |
| d__Bacteria;k__norank_d__Bacteria;p__Proteobacteria;c__Gammaproteobacteria;o__Burkholderiales;f__Gallionellaceae;g__unclassified_f__Gallionellaceae | 7.87E-05 | 0.000153941 | 4.90E-05 | 0 | 0 | 0 | 0 | 0 | 0 | 0 | 0 | 0 |
| d__Bacteria;k__norank_d__Bacteria;p__Proteobacteria;c__Gammaproteobacteria;o__Diplorickettsiales;f__Diplorickettsiaceae;g__Diplorickettsia | 0 | 0.000102627 | 4.90E-05 | 0 | 0 | 0 | 5.23E-05 | 0 | 7.68E-05 | 0 | 0 | 0 |
| d__Bacteria;k__norank_d__Bacteria;p__Calditrichota;c__Calditrichia;o__Calditrichales;f__Calditrichaceae;g__JdFR-76 | 0.000131216 | 5.13E-05 | 0 | 4.29E-05 | 0 | 0 | 0 | 0 | 0 | 2.40E-05 | 2.48E-05 | 0 |
| d__Bacteria;k__norank_d__Bacteria;p__Verrucomicrobiota;c__Verrucomicrobiae;o__Verrucomicrobiales;f__Verrucomicrobiaceae;g__Verrucomicrobium | 0 | 0.000153941 | 2.45E-05 | 2.14E-05 | 2.26E-05 | 0 | 0 | 0 | 0 | 0 | 0 | 5.15E-05 |
| d__Bacteria;k__norank_d__Bacteria;p__Patescibacteria;c__Microgenomatia;o__Candidatus_Woykebacteria;f__norank_o__Candidatus_Woykebacteria;g__norank_f__norank_o__Candidatus_Woykebacteria | 0 | 0 | 0 | 0 | 0 | 4.42E-05 | 0 | 0 | 0 | 0 | 9.91E-05 | 0.000128684 |
| d__Bacteria;k__norank_d__Bacteria;p__Proteobacteria;c__Alphaproteobacteria;o__Micropepsales;f__Micropepsaceae;g__Micropepsis | 5.25E-05 | 2.57E-05 | 4.90E-05 | 0 | 0 | 6.63E-05 | 5.23E-05 | 2.59E-05 | 0 | 0 | 0 | 0 |
| d__Bacteria;k__norank_d__Bacteria;p__Actinobacteriota;c__Actinobacteria;o__Micrococcales;f__Beutenbergiaceae;g__Salana | 2.62E-05 | 0 | 0 | 2.14E-05 | 9.05E-05 | 0.000132632 | 0 | 0 | 0 | 0 | 0 | 0 |
| d__Bacteria;k__norank_d__Bacteria;p__Actinobacteriota;c__Actinobacteria;o__Micromonosporales;f__Micromonosporaceae;g__Catelliglobosispora | 0 | 0 | 0 | 0 | 0 | 2.21E-05 | 0 | 0 | 0 | 7.19E-05 | 0.000123857 | 5.15E-05 |
| d__Bacteria;k__norank_d__Bacteria;p__Bacteroidota;c__Bacteroidia;o__Chitinophagales;f__Chitinophagaceae;g__Segetibacter | 0 | 0 | 7.36E-05 | 0.000107213 | 0 | 8.84E-05 | 0 | 0 | 0 | 0 | 0 | 0 |
| d__Bacteria;k__norank_d__Bacteria;p__Planctomycetota;c__Pla3_lineage;o__norank_c__Pla3_lineage;f__norank_o__norank_c__Pla3_lineage;g__norank_f__norank_o__norank_c__Pla3_lineage | 5.25E-05 | 0 | 9.81E-05 | 2.14E-05 | 2.26E-05 | 0 | 0 | 0 | 0 | 7.19E-05 | 0 | 0 |
| d__Bacteria;k__norank_d__Bacteria;p__Cyanobacteria;c__Cyanobacteriia;o__Cyanobacteriales;f__Nostocaceae;g__Nostoc_PCC-7107 | 0 | 5.13E-05 | 0 | 4.29E-05 | 2.26E-05 | 4.42E-05 | 0 | 7.78E-05 | 2.56E-05 | 0 | 0 | 0 |
| d__Bacteria;k__norank_d__Bacteria;p__Proteobacteria;c__Alphaproteobacteria;o__Puniceispirillales;f__EF100-94H03;g__norank_f__EF100-94H03 | 0 | 0 | 0 | 0 | 9.05E-05 | 2.21E-05 | 0 | 0 | 0 | 2.40E-05 | 4.95E-05 | 7.72E-05 |
| d__Bacteria;k__norank_d__Bacteria;p__Actinobacteriota;c__Actinobacteria;o__Glycomycetales;f__Glycomycetaceae;g__Glycomyces | 0 | 0 | 0 | 0 | 0 | 0 | 0.000235196 | 2.59E-05 | 0 | 0 | 0 | 0 |
| d__Bacteria;k__norank_d__Bacteria;p__Chloroflexi;c__Ktedonobacteria;o__Ktedonobacterales;f__Ktedonobacteraceae;g__Thermosporothrix | 7.87E-05 | 0 | 0 | 4.29E-05 | 0.000113173 | 0 | 0 | 0 | 0 | 0 | 2.48E-05 | 0 |
| d__Bacteria;k__norank_d__Bacteria;p__Proteobacteria;c__Gammaproteobacteria;o__Burkholderiales;f__Alcaligenaceae;g__Advenella | 0.000209946 | 0 | 2.45E-05 | 0 | 2.26E-05 | 0 | 0 | 0 | 0 | 0 | 0 | 0 |
| d__Bacteria;k__norank_d__Bacteria;p__Actinobacteriota;c__Actinobacteria;o__Propionibacteriales;f__Propionibacteriaceae;g__Micropruina | 0 | 0 | 0 | 0 | 2.26E-05 | 0 | 0.000104531 | 0.000129651 | 0 | 0 | 0 | 0 |
| d__Bacteria;k__norank_d__Bacteria;p__Desulfobacterota;c__Desulfobacteria;o__Desulfobacterales;f__Desulfosarcinaceae;g__Sva0081_sediment_group | 0.000183703 | 2.57E-05 | 2.45E-05 | 0 | 0 | 2.21E-05 | 0 | 0 | 0 | 0 | 0 | 0 |
| d__Bacteria;k__norank_d__Bacteria;p__Proteobacteria;c__Alphaproteobacteria;o__Elsterales;f__unclassified_o__Elsterales;g__unclassified_o__Elsterales | 5.25E-05 | 0 | 7.36E-05 | 0 | 0 | 0 | 2.61E-05 | 5.19E-05 | 0 | 0 | 0 | 5.15E-05 |
| d__Bacteria;k__norank_d__Bacteria;p__Firmicutes;c__Bacilli;o__Thermoactinomycetales;f__Thermoactinomycetaceae;g__unclassified_f__Thermoactinomycetaceae | 0 | 0.000128284 | 0 | 0 | 0 | 0 | 0 | 0 | 0 | 2.40E-05 | 0 | 0.000102947 |
| d__Bacteria;k__norank_d__Bacteria;p__Firmicutes;c__Bacilli;o__Erysipelotrichales;f__Erysipelotrichaceae;g__ZOR0006 | 0.000104973 | 0 | 4.90E-05 | 2.14E-05 | 0 | 0 | 5.23E-05 | 0 | 0 | 0 | 2.48E-05 | 0 |
| d__Bacteria;k__norank_d__Bacteria;p__Chloroflexi;c__Ktedonobacteria;o__Ktedonobacterales;f__Ktedonobacteraceae;g__FCPS473 | 0 | 5.13E-05 | 0.000122612 | 0 | 0 | 0 | 5.23E-05 | 2.59E-05 | 0 | 0 | 0 | 0 |
| d__Bacteria;k__norank_d__Bacteria;p__Proteobacteria;c__Alphaproteobacteria;o__Rhizobiales;f__Rhizobiales_Incertae_Sedis;g__unclassified_f__Rhizobiales_Incertae_Sedis | 0.000104973 | 0 | 0 | 0 | 2.26E-05 | 2.21E-05 | 0 | 0 | 5.12E-05 | 0 | 2.48E-05 | 2.57E-05 |
| d__Bacteria;k__norank_d__Bacteria;p__Bacteroidota;c__Bacteroidia;o__Flavobacteriales;f__Flavobacteriaceae;g__unclassified_f__Flavobacteriaceae | 0 | 0 | 0 | 2.14E-05 | 4.53E-05 | 0.000132632 | 0 | 5.19E-05 | 0 | 0 | 0 | 0 |
| d__Bacteria;k__norank_d__Bacteria;p__Firmicutes;c__Syntrophomonadia;o__Syntrophomonadales;f__Syntrophomonadaceae;g__Syntrophomonas | 2.62E-05 | 5.13E-05 | 4.90E-05 | 0 | 0 | 0 | 0 | 0 | 5.12E-05 | 7.19E-05 | 0 | 0 |
| d__Bacteria;k__norank_d__Bacteria;p__Proteobacteria;c__Alphaproteobacteria;o__Rhizobiales;f__Beijerinckiaceae;g__Camelimonas | 5.25E-05 | 2.57E-05 | 0 | 0 | 2.26E-05 | 2.21E-05 | 0 | 5.19E-05 | 0 | 0 | 7.43E-05 | 0 |
| d__Bacteria;k__norank_d__Bacteria;p__Bacteroidota;c__Bacteroidia;o__unclassified_c__Bacteroidia;f__unclassified_c__Bacteroidia;g__unclassified_c__Bacteroidia | 0 | 0.000102627 | 0 | 0 | 0 | 0 | 0 | 0 | 0 | 7.19E-05 | 7.43E-05 | 0 |
| d__Bacteria;k__norank_d__Bacteria;p__Desulfobacterota;c__Syntrophia;o__Syntrophales;f__Syntrophaceae;g__Syntrophus | 7.87E-05 | 2.57E-05 | 4.90E-05 | 6.43E-05 | 0 | 0 | 2.61E-05 | 0 | 0 | 0 | 0 | 0 |
| d__Bacteria;k__norank_d__Bacteria;p__Desulfobacterota;c__Desulfobacteria;o__Desulfatiglandales;f__Desulfatiglandaceae;g__Desulfatiglans | 5.25E-05 | 0 | 2.45E-05 | 0 | 0 | 8.84E-05 | 0 | 5.19E-05 | 0 | 0 | 0 | 2.57E-05 |
| d__Bacteria;k__norank_d__Bacteria;p__Actinobacteriota;c__Actinobacteria;o__Propionibacteriales;f__Nocardioidaceae;g__Actinopolymorpha | 0 | 2.57E-05 | 0 | 0.000171541 | 4.53E-05 | 0 | 0 | 0 | 0 | 0 | 0 | 0 |
| d__Bacteria;k__norank_d__Bacteria;p__Firmicutes;c__D8A-2;o__norank_c__D8A-2;f__norank_o__norank_c__D8A-2;g__norank_f__norank_o__norank_c__D8A-2 | 0 | 2.57E-05 | 0 | 6.43E-05 | 0 | 2.21E-05 | 2.61E-05 | 0.000103721 | 0 | 0 | 0 | 0 |
| d__Bacteria;k__norank_d__Bacteria;p__Patescibacteria;c__Parcubacteria;o__Candidatus_Liptonbacteria;f__norank_o__Candidatus_Liptonbacteria;g__norank_f__norank_o__Candidatus_Liptonbacteria | 2.62E-05 | 5.13E-05 | 7.36E-05 | 4.29E-05 | 0 | 0 | 0 | 0 | 0 | 4.79E-05 | 0 | 0 |
| d__Bacteria;k__norank_d__Bacteria;p__Cyanobacteria;c__Cyanobacteriia;o__Cyanobacteriales;f__Nostocaceae;g__Sphaerospermopsis_BCCUSP55 | 0 | 0 | 0 | 0.000171541 | 6.79E-05 | 0 | 0 | 0 | 0 | 0 | 0 | 0 |
| d__Bacteria;k__norank_d__Bacteria;p__Halanaerobiaeota;c__Halanaerobiia;o__Halanaerobiales;f__Halobacteroidaceae;g__norank_f__Halobacteroidaceae | 2.62E-05 | 0 | 4.90E-05 | 6.43E-05 | 0 | 4.42E-05 | 0 | 5.19E-05 | 0 | 0 | 0 | 0 |
| d__Bacteria;k__norank_d__Bacteria;p__Bacteroidota;c__Bacteroidia;o__Bacteroidales;f__Prolixibacteraceae;g__WCHB1-32 | 5.25E-05 | 0 | 0 | 0 | 0 | 0 | 0 | 0.000181512 | 0 | 0 | 0 | 0 |
| d__Bacteria;k__norank_d__Bacteria;p__Proteobacteria;c__Alphaproteobacteria;o__Acetobacterales;f__Acetobacteraceae;g__Acidisoma | 5.25E-05 | 0 | 0 | 0 | 0 | 0 | 2.61E-05 | 5.19E-05 | 0.000102404 | 0 | 0 | 0 |
| d__Bacteria;k__norank_d__Bacteria;p__Proteobacteria;c__Alphaproteobacteria;o__Sphingomonadales;f__Sphingomonadaceae;g__Sphingosinicella | 0 | 7.70E-05 | 2.45E-05 | 0.000107213 | 2.26E-05 | 0 | 0 | 0 | 0 | 0 | 0 | 0 |
| d__Bacteria;k__norank_d__Bacteria;p__Proteobacteria;c__Alphaproteobacteria;o__Rickettsiales;f__AB1;g__norank_f__AB1 | 0 | 0 | 4.90E-05 | 0 | 0 | 0 | 0.000104531 | 0 | 5.12E-05 | 0 | 0 | 2.57E-05 |
| d__Bacteria;k__norank_d__Bacteria;p__Myxococcota;c__Polyangia;o__Polyangiales;f__Eel-36e1D6;g__norank_f__Eel-36e1D6 | 2.62E-05 | 2.57E-05 | 0 | 0.000128656 | 0 | 0 | 0 | 0 | 0 | 2.40E-05 | 0 | 2.57E-05 |
| d__Bacteria;k__norank_d__Bacteria;p__Proteobacteria;c__Gammaproteobacteria;o__Xanthomonadales;f__Rhodanobacteraceae;g__Mizugakiibacter | 0 | 0.000102627 | 7.36E-05 | 0 | 0 | 0 | 0 | 5.19E-05 | 0 | 0 | 0 | 0 |
| d__Bacteria;k__norank_d__Bacteria;p__Proteobacteria;c__Gammaproteobacteria;o__Burkholderiales;f__Nitrosomonadaceae;g__norank_f__Nitrosomonadaceae | 2.62E-05 | 2.57E-05 | 0.000122612 | 0 | 0 | 0 | 0 | 2.59E-05 | 2.56E-05 | 0 | 0 | 0 |
| d__Bacteria;k__norank_d__Bacteria;p__Desulfobacterota;c__Desulfobaccia;o__Desulfobaccales;f__Desulfobaccaceae;g__Desulfobacca | 5.25E-05 | 5.13E-05 | 4.90E-05 | 0 | 2.26E-05 | 0 | 2.61E-05 | 0 | 0 | 2.40E-05 | 0 | 0 |
| d__Bacteria;k__norank_d__Bacteria;p__Proteobacteria;c__Gammaproteobacteria;o__Enterobacterales;f__Enterobacteriaceae;g__unclassified_f__Enterobacteriaceae | 0 | 7.70E-05 | 0.000122612 | 0 | 0 | 0 | 0 | 0 | 2.56E-05 | 0 | 0 | 0 |
| d__Bacteria;k__norank_d__Bacteria;p__Proteobacteria;c__Gammaproteobacteria;o__Salinisphaerales;f__Solimonadaceae;g__Polycyclovorans | 7.87E-05 | 5.13E-05 | 0 | 0 | 2.26E-05 | 0 | 0 | 0 | 0 | 7.19E-05 | 0 | 0 |
| d__Bacteria;k__norank_d__Bacteria;p__Verrucomicrobiota;c__Verrucomicrobiae;o__Pedosphaerales;f__Pedosphaeraceae;g__SH3-11 | 0 | 5.13E-05 | 7.36E-05 | 0 | 2.26E-05 | 0 | 2.61E-05 | 0 | 0 | 0 | 2.48E-05 | 2.57E-05 |
| d__Bacteria;k__norank_d__Bacteria;p__Verrucomicrobiota;c__Verrucomicrobiae;o__S-BQ2-57_soil_group;f__norank_o__S-BQ2-57_soil_group;g__norank_f__norank_o__S-BQ2-57_soil_group | 0 | 2.57E-05 | 0.000171657 | 0 | 0 | 0 | 0 | 0 | 0 | 0 | 0 | 2.57E-05 |
| d__Bacteria;k__norank_d__Bacteria;p__Desulfobacterota;c__Syntrophobacteria;o__Syntrophobacterales;f__norank_o__Syntrophobacterales;g__norank_f__norank_o__Syntrophobacterales | 0.000104973 | 0 | 7.36E-05 | 2.14E-05 | 0 | 2.21E-05 | 0 | 0 | 0 | 0 | 0 | 0 |
| d__Bacteria;k__norank_d__Bacteria;p__Bdellovibrionota;c__Oligoflexia;o__Oligoflexales;f__norank_o__Oligoflexales;g__unclassified_f__norank_o__Oligoflexales | 0 | 2.57E-05 | 0.000147135 | 0 | 0 | 0 | 0 | 0 | 0 | 2.40E-05 | 2.48E-05 | 0 |
| d__Bacteria;k__norank_d__Bacteria;p__Actinobacteriota;c__Actinobacteria;o__Micrococcales;f__Dermabacteraceae;g__Brachybacterium | 2.62E-05 | 0 | 0 | 0.000192984 | 0 | 0 | 0 | 0 | 0 | 0 | 0 | 0 |
| d__Bacteria;k__norank_d__Bacteria;p__Firmicutes;c__Clostridia;o__Clostridiales;f__Clostridiaceae;g__Clostridium_sensu_stricto_7 | 2.62E-05 | 0 | 2.45E-05 | 4.29E-05 | 0 | 0 | 0 | 5.19E-05 | 2.56E-05 | 4.79E-05 | 0 | 0 |
| d__Bacteria;k__norank_d__Bacteria;p__Firmicutes;c__Clostridia;o__norank_c__Clostridia;f__Hungateiclostridiaceae;g__HN-HF0106 | 0 | 5.13E-05 | 7.36E-05 | 2.14E-05 | 2.26E-05 | 0 | 0 | 0 | 0 | 4.79E-05 | 0 | 0 |
| d__Bacteria;k__norank_d__Bacteria;p__Proteobacteria;c__Gammaproteobacteria;o__Burkholderiales;f__Sulfuricellaceae;g__Sulfuricella | 5.25E-05 | 0 | 4.90E-05 | 0 | 0 | 8.84E-05 | 0 | 0 | 0 | 0 | 0 | 2.57E-05 |
| d__Bacteria;k__norank_d__Bacteria;p__Actinobacteriota;c__Rubrobacteria;o__Rubrobacterales;f__Rubrobacteriaceae;g__Rubrobacter | 2.62E-05 | 0 | 2.45E-05 | 2.14E-05 | 0 | 4.42E-05 | 0 | 0 | 0 | 0 | 9.91E-05 | 0 |
| d__Bacteria;k__norank_d__Bacteria;p__Actinobacteriota;c__Thermoleophilia;o__Solirubrobacterales;f__Solirubrobacteraceae;g__JCM_18997 | 0 | 0 | 0 | 4.29E-05 | 0 | 6.63E-05 | 7.84E-05 | 2.59E-05 | 0 | 0 | 0 | 0 |
| d__Bacteria;k__norank_d__Bacteria;p__Proteobacteria;c__Alphaproteobacteria;o__Elsterales;f__URHD0088;g__norank_f__URHD0088 | 0.000104973 | 2.57E-05 | 0 | 0 | 0 | 0 | 0 | 7.78E-05 | 0 | 0 | 0 | 0 |
| d__Bacteria;k__norank_d__Bacteria;p__Acidobacteriota;c__Blastocatellia;o__Elev-16S-573;f__norank_o__Elev-16S-573;g__norank_f__norank_o__Elev-16S-573 | 0 | 0.000128284 | 0 | 0 | 0 | 0 | 5.23E-05 | 0 | 0 | 0 | 2.48E-05 | 0 |
| d__Bacteria;k__norank_d__Bacteria;p__Desulfobacterota;c__Desulfobacteria;o__Desulfobacterales;f__Desulfosarcinaceae;g__Desulfatirhabdium | 0.000131216 | 5.13E-05 | 0 | 0 | 0 | 2.21E-05 | 0 | 0 | 0 | 0 | 0 | 0 |
| d__Bacteria;k__norank_d__Bacteria;p__Proteobacteria;c__Alphaproteobacteria;o__Acetobacterales;f__Acetobacteraceae;g__Roseococcus | 5.25E-05 | 0 | 0 | 0 | 2.26E-05 | 0 | 2.61E-05 | 5.19E-05 | 0 | 0 | 0 | 5.15E-05 |
| d__Bacteria;k__norank_d__Bacteria;p__Proteobacteria;c__Alphaproteobacteria;o__Ferrovibrionales;f__norank_o__Ferrovibrionales;g__Taonella | 0 | 0 | 0 | 0 | 0 | 0 | 2.61E-05 | 0 | 0.000128005 | 0 | 4.95E-05 | 0 |
| d__Bacteria;k__norank_d__Bacteria;p__Chloroflexi;c__Chloroflexia;o__Elev-1554;f__norank_o__Elev-1554;g__norank_f__norank_o__Elev-1554 | 5.25E-05 | 0 | 0 | 2.14E-05 | 0 | 0 | 5.23E-05 | 2.59E-05 | 5.12E-05 | 0 | 0 | 0 |
| d__Bacteria;k__norank_d__Bacteria;p__Actinobacteriota;c__Actinobacteria;o__Pseudonocardiales;f__Pseudonocardiaceae;g__Crossiella | 2.62E-05 | 7.70E-05 | 7.36E-05 | 0 | 0 | 0 | 0 | 2.59E-05 | 0 | 0 | 0 | 0 |
| d__Bacteria;k__norank_d__Bacteria;p__Proteobacteria;c__Gammaproteobacteria;o__Beggiatoales;f__Beggiatoaceae;g__norank_f__Beggiatoaceae | 7.87E-05 | 0 | 0.000122612 | 0 | 0 | 0 | 0 | 0 | 0 | 0 | 0 | 0 |
| d__Bacteria;k__norank_d__Bacteria;p__Actinobacteriota;c__Actinobacteria;o__Streptomycetales;f__Streptomycetaceae;g__unclassified_f__Streptomycetaceae | 7.87E-05 | 0 | 0 | 4.29E-05 | 0 | 0 | 2.61E-05 | 5.19E-05 | 0 | 0 | 0 | 0 |
| d__Bacteria;k__norank_d__Bacteria;p__Proteobacteria;c__Gammaproteobacteria;o__Burkholderiales;f__Nitrosomonadaceae;g__unclassified_f__Nitrosomonadaceae | 0 | 0.000102627 | 4.90E-05 | 0 | 0 | 0 | 0 | 0 | 0 | 4.79E-05 | 0 | 0 |
| d__Bacteria;k__norank_d__Bacteria;p__Firmicutes;c__Bacilli;o__Bacillales;f__Bacillaceae;g__Oceanobacillus | 0.000104973 | 2.57E-05 | 0 | 0 | 4.53E-05 | 2.21E-05 | 0 | 0 | 0 | 0 | 0 | 0 |
| d__Bacteria;k__norank_d__Bacteria;p__Actinobacteriota;c__Actinobacteria;o__Micrococcales;f__Intrasporangiaceae;g__Aquipuribacter | 0 | 0 | 0 | 4.29E-05 | 0 | 0.000154737 | 0 | 0 | 0 | 0 | 0 | 0 |
| d__Bacteria;k__norank_d__Bacteria;p__Proteobacteria;c__Alphaproteobacteria;o__Rickettsiales;f__Rickettsiaceae;g__unclassified_f__Rickettsiaceae | 0 | 0 | 4.90E-05 | 0 | 4.53E-05 | 0 | 0 | 0 | 0 | 0 | 0 | 0.000102947 |
| d__Bacteria;k__norank_d__Bacteria;p__Actinobacteriota;c__Actinobacteria;o__Propionibacteriales;f__Propionibacteriaceae;g__norank_f__Propionibacteriaceae | 0 | 0 | 0 | 0 | 6.79E-05 | 0 | 5.23E-05 | 5.19E-05 | 0 | 0 | 2.48E-05 | 0 |
| d__Bacteria;k__norank_d__Bacteria;p__Firmicutes;c__Bacilli;o__Thermoactinomycetales;f__Thermoactinomycetaceae;g__Kroppenstedtia | 5.25E-05 | 0 | 0 | 0 | 6.79E-05 | 0 | 0 | 0 | 0 | 0 | 2.48E-05 | 5.15E-05 |
| d__Bacteria;k__norank_d__Bacteria;p__Acidobacteriota;c__Vicinamibacteria;o__unclassified_c__Vicinamibacteria;f__unclassified_c__Vicinamibacteria;g__unclassified_c__Vicinamibacteria | 0 | 2.57E-05 | 2.45E-05 | 0 | 2.26E-05 | 0 | 0 | 0 | 0 | 4.79E-05 | 7.43E-05 | 0 |
| d__Bacteria;k__norank_d__Bacteria;p__Firmicutes;c__Bacilli;o__Bacillales;f__Sporolactobacillaceae;g__Pullulanibacillus | 2.62E-05 | 0 | 2.45E-05 | 0 | 9.05E-05 | 0 | 0 | 2.59E-05 | 0 | 0 | 0 | 2.57E-05 |
| d__Bacteria;k__norank_d__Bacteria;p__Proteobacteria;c__Gammaproteobacteria;o__Xanthomonadales;f__Rhodanobacteraceae;g__Tahibacter | 0 | 5.13E-05 | 0 | 4.29E-05 | 0 | 0 | 0 | 0 | 0 | 2.40E-05 | 7.43E-05 | 0 |
| d__Bacteria;k__norank_d__Bacteria;p__Proteobacteria;c__Gammaproteobacteria;o__Burkholderiales;f__Rhodocyclaceae;g__Candidatus_Accumulibacter | 5.25E-05 | 0 | 4.90E-05 | 0 | 9.05E-05 | 0 | 0 | 0 | 0 | 0 | 0 | 0 |
| d__Bacteria;k__norank_d__Bacteria;p__Actinobacteriota;c__Actinobacteria;o__Propionibacteriales;f__Propionibacteriaceae;g__unclassified_f__Propionibacteriaceae | 0 | 0 | 0 | 0 | 2.26E-05 | 4.42E-05 | 0 | 0 | 5.12E-05 | 2.40E-05 | 4.95E-05 | 0 |
| d__Bacteria;k__norank_d__Bacteria;p__Firmicutes;c__Clostridia;o__Clostridiales;f__Clostridiaceae;g__Clostridium_sensu_stricto_18 | 0 | 0 | 4.90E-05 | 0 | 2.26E-05 | 6.63E-05 | 0 | 5.19E-05 | 0 | 0 | 0 | 0 |
| d__Bacteria;k__norank_d__Bacteria;p__Proteobacteria;c__Alphaproteobacteria;o__Rhizobiales;f__Hyphomicrobiaceae;g__norank_f__Hyphomicrobiaceae | 0 | 0 | 7.36E-05 | 4.29E-05 | 2.26E-05 | 0 | 2.61E-05 | 0 | 0 | 2.40E-05 | 0 | 0 |
| d__Bacteria;k__norank_d__Bacteria;p__Chloroflexi;c__Anaerolineae;o__Caldilineales;f__Caldilineaceae;g__unclassified_f__Caldilineaceae | 0 | 2.57E-05 | 0 | 6.43E-05 | 0 | 0 | 0 | 0 | 0 | 2.40E-05 | 7.43E-05 | 0 |
| d__Bacteria;k__norank_d__Bacteria;p__Proteobacteria;c__Gammaproteobacteria;o__Burkholderiales;f__Chromobacteriaceae;g__Vogesella | 0 | 0 | 0 | 6.43E-05 | 0 | 4.42E-05 | 5.23E-05 | 0 | 0 | 0 | 2.48E-05 | 0 |
| d__Bacteria;k__norank_d__Bacteria;p__Firmicutes;c__Bacilli;o__Exiguobacterales;f__Exiguobacteraceae;g__Exiguobacterium | 0 | 0 | 0 | 0 | 6.79E-05 | 6.63E-05 | 2.61E-05 | 0 | 0 | 2.40E-05 | 0 | 0 |
| d__Bacteria;k__norank_d__Bacteria;p__Proteobacteria;c__Alphaproteobacteria;o__Acetobacterales;f__Acetobacteraceae;g__Acidisphaera | 0 | 0 | 0 | 0 | 0 | 0 | 0.000156797 | 2.59E-05 | 0 | 0 | 0 | 0 |
| d__Bacteria;k__norank_d__Bacteria;p__Bacteroidota;c__Ignavibacteria;o__Ignavibacteriales;f__Ignavibacteriaceae;g__Ignavibacterium | 0.000104973 | 0 | 2.45E-05 | 0 | 0 | 0 | 5.23E-05 | 0 | 0 | 0 | 0 | 0 |
| d__Bacteria;k__norank_d__Bacteria;p__Bacteroidota;c__Bacteroidia;o__Chitinophagales;f__Chitinophagaceae;g__Niveitalea | 2.62E-05 | 0 | 2.45E-05 | 8.58E-05 | 0 | 4.42E-05 | 0 | 0 | 0 | 0 | 0 | 0 |
| d__Bacteria;k__norank_d__Bacteria;p__Firmicutes;c__Bacilli;o__Izemoplasmatales;f__norank_o__Izemoplasmatales;g__norank_f__norank_o__Izemoplasmatales | 0 | 5.13E-05 | 0 | 0 | 0 | 0 | 5.23E-05 | 0 | 5.12E-05 | 0 | 0 | 2.57E-05 |
| d__Bacteria;k__norank_d__Bacteria;p__Cyanobacteria;c__Cyanobacteriia;o__Cyanobacteriales;f__Chroococcidiopsaceae;g__norank_f__Chroococcidiopsaceae | 0 | 0 | 0 | 0.000107213 | 2.26E-05 | 0 | 0 | 0 | 0 | 0 | 4.95E-05 | 0 |
| d__Bacteria;k__norank_d__Bacteria;p__Verrucomicrobiota;c__Chlamydiae;o__Chlamydiales;f__Chlamydiaceae;g__norank_f__Chlamydiaceae | 0 | 7.70E-05 | 4.90E-05 | 0 | 0 | 0 | 5.23E-05 | 0 | 0 | 0 | 0 | 0 |
| d__Bacteria;k__norank_d__Bacteria;p__Bacteroidota;c__Bacteroidia;o__Cytophagales;f__Hymenobacteraceae;g__Pontibacter | 0 | 0 | 0 | 0 | 0 | 0 | 5.23E-05 | 2.59E-05 | 5.12E-05 | 2.40E-05 | 2.48E-05 | 0 |
| d__Bacteria;k__norank_d__Bacteria;p__Desulfobacterota;c__Desulfobulbia;o__Desulfobulbales;f__norank_o__Desulfobulbales;g__norank_f__norank_o__Desulfobulbales | 7.87E-05 | 2.57E-05 | 7.36E-05 | 0 | 0 | 0 | 0 | 0 | 0 | 0 | 0 | 0 |
| d__Bacteria;k__norank_d__Bacteria;p__Verrucomicrobiota;c__Verrucomicrobiae;o__Opitutales;f__Opitutaceae;g__Alterococcus | 0.000104973 | 2.57E-05 | 2.45E-05 | 0 | 0 | 2.21E-05 | 0 | 0 | 0 | 0 | 0 | 0 |
| d__Bacteria;k__norank_d__Bacteria;p__Actinobacteriota;c__Actinobacteria;o__Streptosporangiales;f__Nocardiopsaceae;g__Nocardiopsis | 0 | 0 | 2.45E-05 | 0 | 2.26E-05 | 0 | 7.84E-05 | 2.59E-05 | 2.56E-05 | 0 | 0 | 0 |
| d__Bacteria;k__norank_d__Bacteria;p__Patescibacteria;c__Parcubacteria;o__Candidatus_Adlerbacteria;f__norank_o__Candidatus_Adlerbacteria;g__norank_f__norank_o__Candidatus_Adlerbacteria | 0 | 0 | 2.45E-05 | 0 | 0 | 0 | 0 | 0 | 0 | 0 | 4.95E-05 | 0.000102947 |
| d__Bacteria;k__norank_d__Bacteria;p__Firmicutes;c__Clostridia;o__Oscillospirales;f__Ruminococcaceae;g__Caproiciproducens | 0 | 0.000102627 | 4.90E-05 | 0 | 0 | 0 | 0 | 0 | 0 | 2.40E-05 | 0 | 0 |
| d__Bacteria;k__norank_d__Bacteria;p__Proteobacteria;c__Alphaproteobacteria;o__Rickettsiales;f__Fokiniaceae;g__norank_f__Fokiniaceae | 0 | 0 | 0 | 0 | 0 | 0 | 5.23E-05 | 0 | 0 | 7.19E-05 | 0 | 5.15E-05 |
| d__Bacteria;k__norank_d__Bacteria;p__Proteobacteria;c__Alphaproteobacteria;o__Rhodobacterales;f__Rhodobacteraceae;g__Pseudorhodobacter | 2.62E-05 | 2.57E-05 | 4.90E-05 | 0 | 0 | 0 | 0 | 2.59E-05 | 0 | 4.79E-05 | 0 | 0 |
| d__Bacteria;k__norank_d__Bacteria;p__DTB120;c__norank_p__DTB120;o__norank_c__norank_p__DTB120;f__norank_o__norank_c__norank_p__DTB120;g__norank_f__norank_o__norank_c__norank_p__DTB120 | 0 | 2.57E-05 | 0.000122612 | 0 | 0 | 0 | 0 | 2.59E-05 | 0 | 0 | 0 | 0 |
| d__Bacteria;k__norank_d__Bacteria;p__Proteobacteria;c__Alphaproteobacteria;o__Rhodobacterales;f__Rhodobacteraceae;g__Limibaculum | 0 | 0 | 0 | 0 | 0 | 0 | 0 | 0 | 0 | 7.19E-05 | 2.48E-05 | 7.72E-05 |
| d__Bacteria;k__norank_d__Bacteria;p__Elusimicrobiota;c__Elusimicrobia;o__FCPU453;f__norank_o__FCPU453;g__norank_f__norank_o__FCPU453 | 5.25E-05 | 2.57E-05 | 7.36E-05 | 0 | 0 | 2.21E-05 | 0 | 0 | 0 | 0 | 0 | 0 |
| d__Bacteria;k__norank_d__Bacteria;p__Verrucomicrobiota;c__Verrucomicrobiae;o__Verrucomicrobiales;f__Verrucomicrobiaceae;g__Prosthecobacter | 5.25E-05 | 2.57E-05 | 7.36E-05 | 0 | 0 | 2.21E-05 | 0 | 0 | 0 | 0 | 0 | 0 |
| d__Bacteria;k__norank_d__Bacteria;p__Planctomycetota;c__Phycisphaerae;o__Phycisphaerales;f__Phycisphaeraceae;g__CL500-3 | 0 | 0 | 7.36E-05 | 0 | 0 | 0 | 0 | 0 | 0 | 4.79E-05 | 2.48E-05 | 2.57E-05 |
| d__Bacteria;k__norank_d__Bacteria;p__Firmicutes;c__Clostridia;o__Oscillospirales;f__Oscillospiraceae;g__Papillibacter | 0 | 2.57E-05 | 7.36E-05 | 0 | 0 | 0 | 0 | 0 | 0 | 4.79E-05 | 2.48E-05 | 0 |
| d__Bacteria;k__norank_d__Bacteria;p__Desulfobacterota;c__Desulfuromonadia;o__norank_c__Desulfuromonadia;f__unclassified_o__norank_c__Desulfuromonadia;g__unclassified_o__norank_c__Desulfuromonadia | 0 | 0 | 0.000171657 | 0 | 0 | 0 | 0 | 0 | 0 | 0 | 0 | 0 |
| d__Bacteria;k__norank_d__Bacteria;p__Proteobacteria;c__Alphaproteobacteria;o__Micavibrionales;f__unclassified_o__Micavibrionales;g__unclassified_o__Micavibrionales | 0 | 0 | 0.000171657 | 0 | 0 | 0 | 0 | 0 | 0 | 0 | 0 | 0 |
| d__Bacteria;k__norank_d__Bacteria;p__Firmicutes;c__Bacilli;o__Paenibacillales;f__Paenibacillaceae;g__Fontibacillus | 0 | 0 | 4.90E-05 | 4.29E-05 | 0 | 0 | 2.61E-05 | 0 | 2.56E-05 | 0 | 0 | 2.57E-05 |
| d__Bacteria;k__norank_d__Bacteria;p__Proteobacteria;c__Alphaproteobacteria;o__Rhodobacterales;f__Rhodobacteraceae;g__Rubellimicrobium | 2.62E-05 | 0 | 0 | 0 | 0 | 6.63E-05 | 0 | 2.59E-05 | 0 | 0 | 4.95E-05 | 0 |
| d__Bacteria;k__norank_d__Bacteria;p__Actinobacteriota;c__Actinobacteria;o__Streptosporangiales;f__Streptosporangiaceae;g__Sphaerisporangium | 0 | 0 | 0 | 2.14E-05 | 4.53E-05 | 0 | 0 | 2.59E-05 | 2.56E-05 | 0 | 4.95E-05 | 0 |
| d__Bacteria;k__norank_d__Bacteria;p__Proteobacteria;c__Alphaproteobacteria;o__Parvibaculales;f__Parvibaculaceae;g__norank_f__Parvibaculaceae | 0 | 2.57E-05 | 2.45E-05 | 0 | 4.53E-05 | 2.21E-05 | 0 | 2.59E-05 | 0 | 2.40E-05 | 0 | 0 |
| d__Bacteria;k__norank_d__Bacteria;p__Proteobacteria;c__Gammaproteobacteria;o__Burkholderiales;f__Comamonadaceae;g__Aquabacterium | 0 | 5.13E-05 | 0 | 6.43E-05 | 0 | 0 | 0 | 2.59E-05 | 2.56E-05 | 0 | 0 | 0 |
| d__Bacteria;k__norank_d__Bacteria;p__Planctomycetota;c__Pla4_lineage;o__norank_c__Pla4_lineage;f__norank_o__norank_c__Pla4_lineage;g__norank_f__norank_o__norank_c__Pla4_lineage | 0 | 0 | 4.90E-05 | 4.29E-05 | 0 | 0 | 0 | 0 | 0 | 2.40E-05 | 2.48E-05 | 2.57E-05 |
| d__Bacteria;k__norank_d__Bacteria;p__Proteobacteria;c__Gammaproteobacteria;o__Burkholderiales;f__Oxalobacteraceae;g__norank_f__Oxalobacteraceae | 0 | 2.57E-05 | 0 | 2.14E-05 | 2.26E-05 | 4.42E-05 | 0 | 0 | 5.12E-05 | 0 | 0 | 0 |
| d__Bacteria;k__norank_d__Bacteria;p__Bdellovibrionota;c__Oligoflexia;o__053A03-B-DI-P58;f__norank_o__053A03-B-DI-P58;g__norank_f__norank_o__053A03-B-DI-P58 | 0 | 0 | 0 | 6.43E-05 | 0 | 0 | 0 | 0 | 0 | 4.79E-05 | 4.95E-05 | 0 |
| d__Bacteria;k__norank_d__Bacteria;p__Desulfobacterota;c__Desulfobulbia;o__Desulfobulbales;f__Desulfocapsaceae;g__Desulfopila | 0 | 0 | 0 | 0 | 0.000158443 | 0 | 0 | 0 | 0 | 0 | 0 | 0 |
| d__Bacteria;k__norank_d__Bacteria;p__Proteobacteria;c__Alphaproteobacteria;o__Rhizobiales;f__Rhizobiaceae;g__Rhizobium_sphaerophysae_group | 0 | 0 | 0 | 0 | 9.05E-05 | 6.63E-05 | 0 | 0 | 0 | 0 | 0 | 0 |
| d__Bacteria;k__norank_d__Bacteria;p__Desulfobacterota;c__Desulfovibrionia;o__Desulfovibrionales;f__Desulfovibrionaceae;g__Desulfovibrio | 0.000104973 | 5.13E-05 | 0 | 0 | 0 | 0 | 0 | 0 | 0 | 0 | 0 | 0 |
| d__Bacteria;k__norank_d__Bacteria;p__Bacteroidota;c__Bacteroidia;o__Chitinophagales;f__Chitinophagaceae;g__Flaviaesturariibacter | 0 | 0 | 0 | 0 | 0 | 0 | 7.84E-05 | 5.19E-05 | 2.56E-05 | 0 | 0 | 0 |
| d__Bacteria;k__norank_d__Bacteria;p__Firmicutes;c__Clostridia;o__Peptostreptococcales-Tissierellales;f__norank_o__Peptostreptococcales-Tissierellales;g__Gottschalkia | 0 | 7.70E-05 | 0 | 0 | 0 | 0 | 7.84E-05 | 0 | 0 | 0 | 0 | 0 |
| d__Bacteria;k__norank_d__Bacteria;p__Myxococcota;c__Polyangia;o__Polyangiales;f__Polyangiaceae;g__Minicystis | 0 | 0 | 2.45E-05 | 0 | 0 | 0 | 0.000130664 | 0 | 0 | 0 | 0 | 0 |
| d__Bacteria;k__norank_d__Bacteria;p__Bacteroidota;c__Bacteroidia;o__Flavobacteriales;f__Flavobacteriaceae;g__Confluentibacter | 0.000104973 | 2.57E-05 | 2.45E-05 | 0 | 0 | 0 | 0 | 0 | 0 | 0 | 0 | 0 |
| d__Bacteria;k__norank_d__Bacteria;p__Verrucomicrobiota;c__Verrucomicrobiae;o__Verrucomicrobiales;f__Verrucomicrobiaceae;g__unclassified_f__Verrucomicrobiaceae | 0 | 2.57E-05 | 2.45E-05 | 0 | 0 | 0 | 0.000104531 | 0 | 0 | 0 | 0 | 0 |
| d__Bacteria;k__norank_d__Bacteria;p__Actinobacteriota;c__Thermoleophilia;o__Solirubrobacterales;f__Solirubrobacteraceae;g__Parviterribacter | 2.62E-05 | 2.57E-05 | 0 | 0 | 0 | 0 | 0 | 0 | 0.000102404 | 0 | 0 | 0 |
| d__Bacteria;k__norank_d__Bacteria;p__Firmicutes;c__Thermoanaerobacteria;o__Thermoanaerobacterales;f__SRB2;g__norank_f__SRB2 | 0 | 0 | 2.45E-05 | 0 | 0 | 0 | 0 | 0.000129651 | 0 | 0 | 0 | 0 |
| d__Bacteria;k__norank_d__Bacteria;p__Firmicutes;c__Clostridia;o__Peptostreptococcales-Tissierellales;f__Anaerovoracaceae;g__unclassified_f__Anaerovoracaceae | 2.62E-05 | 7.70E-05 | 2.45E-05 | 0 | 0 | 0 | 0 | 2.59E-05 | 0 | 0 | 0 | 0 |
| d__Bacteria;k__norank_d__Bacteria;p__Proteobacteria;c__Alphaproteobacteria;o__Rhodobacterales;f__Rhodobacteraceae;g__Gemmobacter | 2.62E-05 | 7.70E-05 | 2.45E-05 | 0 | 0 | 0 | 0 | 0 | 0 | 0 | 2.48E-05 | 0 |
| d__Bacteria;k__norank_d__Bacteria;p__Hydrogenedentes;c__Hydrogenedentia;o__Hydrogenedentiales;f__Hydrogenedensaceae;g__norank_f__Hydrogenedensaceae | 2.62E-05 | 0 | 7.36E-05 | 0 | 0 | 0 | 5.23E-05 | 0 | 0 | 0 | 0 | 0 |
| d__Bacteria;k__norank_d__Bacteria;p__Bdellovibrionota;c__Oligoflexia;o__Silvanigrellales;f__Silvanigrellaceae;g__Silvanigrella | 0 | 5.13E-05 | 2.45E-05 | 0 | 0 | 0 | 0 | 0 | 5.12E-05 | 0 | 2.48E-05 | 0 |
| d__Bacteria;k__norank_d__Bacteria;p__Chloroflexi;c__Anaerolineae;o__Anaerolineales;f__Anaerolineaceae;g__Leptolinea | 0 | 0.000102627 | 4.90E-05 | 0 | 0 | 0 | 0 | 0 | 0 | 0 | 0 | 0 |
| d__Bacteria;k__norank_d__Bacteria;p__Firmicutes;c__Bacilli;o__Bacillales;f__Bacillaceae;g__Aureibacillus | 2.62E-05 | 2.57E-05 | 7.36E-05 | 0 | 0 | 0 | 0 | 0 | 0 | 0 | 2.48E-05 | 0 |
| d__Bacteria;k__norank_d__Bacteria;p__Verrucomicrobiota;c__Verrucomicrobiae;o__Methylacidiphilales;f__Methylacidiphilaceae;g__norank_f__Methylacidiphilaceae | 0 | 2.57E-05 | 9.81E-05 | 0 | 0 | 0 | 2.61E-05 | 0 | 0 | 0 | 0 | 0 |
| d__Bacteria;k__norank_d__Bacteria;p__Desulfobacterota;c__Desulfomonilia;o__Desulfomonilales;f__Desulfomonilaceae;g__Desulfomonile | 7.87E-05 | 0 | 2.45E-05 | 0 | 2.26E-05 | 0 | 0 | 0 | 0 | 2.40E-05 | 0 | 0 |
| d__Bacteria;k__norank_d__Bacteria;p__Proteobacteria;c__Gammaproteobacteria;o__Gammaproteobacteria_Incertae_Sedis;f__unclassified_o__Gammaproteobacteria_Incertae_Sedis;g__unclassified_o__Gammaproteobacteria_Incertae_Sedis | 0 | 0 | 0 | 0 | 0 | 0 | 0 | 7.78E-05 | 0 | 7.19E-05 | 0 | 0 |
| d__Bacteria;k__norank_d__Bacteria;p__Proteobacteria;c__Gammaproteobacteria;o__Burkholderiales;f__Oxalobacteraceae;g__Duganella | 0 | 2.57E-05 | 4.90E-05 | 0 | 0 | 0 | 0 | 0 | 0 | 0 | 7.43E-05 | 0 |
| d__Bacteria;k__norank_d__Bacteria;p__Actinobacteriota;c__Actinobacteria;o__Streptosporangiales;f__Nocardiopsaceae;g__Thermobifida | 0 | 0 | 0 | 0 | 0 | 0 | 0 | 0 | 2.56E-05 | 4.79E-05 | 4.95E-05 | 2.57E-05 |
| d__Bacteria;k__norank_d__Bacteria;p__Firmicutes;c__Clostridia;o__Clostridiales;f__Clostridiaceae;g__Haloimpatiens | 7.87E-05 | 0 | 0 | 0 | 0 | 4.42E-05 | 0 | 0 | 0 | 0 | 0 | 2.57E-05 |
| d__Bacteria;k__norank_d__Bacteria;p__Actinobacteriota;c__Actinobacteria;o__Pseudonocardiales;f__Pseudonocardiaceae;g__Actinomycetospora | 2.62E-05 | 0 | 0 | 0 | 4.53E-05 | 0 | 0 | 0 | 7.68E-05 | 0 | 0 | 0 |
| d__Bacteria;k__norank_d__Bacteria;p__Proteobacteria;c__Alphaproteobacteria;o__Caedibacterales;f__Caedibacteraceae;g__Candidatus_Nucleicultrix | 0 | 0 | 0 | 0 | 2.26E-05 | 0 | 0 | 5.19E-05 | 0 | 4.79E-05 | 0 | 2.57E-05 |
| d__Bacteria;k__norank_d__Bacteria;p__Proteobacteria;c__Gammaproteobacteria;o__Cellvibrionales;f__Halieaceae;g__Haliea | 0 | 0 | 4.90E-05 | 0 | 0 | 0 | 0 | 0 | 0 | 4.79E-05 | 2.48E-05 | 2.57E-05 |
| d__Bacteria;k__norank_d__Bacteria;p__Planctomycetota;c__Planctomycetes;o__Isosphaerales;f__Isosphaeraceae;g__Paludisphaera | 0 | 0 | 2.45E-05 | 2.14E-05 | 2.26E-05 | 0 | 7.84E-05 | 0 | 0 | 0 | 0 | 0 |
| d__Bacteria;k__norank_d__Bacteria;p__Firmicutes;c__Bacilli;o__Erysipelotrichales;f__Erysipelatoclostridiaceae;g__Erysipelatoclostridium | 5.25E-05 | 2.57E-05 | 2.45E-05 | 0 | 0 | 4.42E-05 | 0 | 0 | 0 | 0 | 0 | 0 |
| d__Bacteria;k__norank_d__Bacteria;p__Verrucomicrobiota;c__Chlamydiae;o__Chlamydiales;f__Simkaniaceae;g__Ga0074140 | 0 | 7.70E-05 | 2.45E-05 | 0 | 2.26E-05 | 2.21E-05 | 0 | 0 | 0 | 0 | 0 | 0 |
| d__Bacteria;k__norank_d__Bacteria;p__Myxococcota;c__Myxococcia;o__Myxococcales;f__Myxococcaceae;g__Corallococcus | 0 | 0 | 0 | 0 | 0 | 4.42E-05 | 0 | 0 | 0 | 2.40E-05 | 0 | 7.72E-05 |
| d__Bacteria;k__norank_d__Bacteria;p__Proteobacteria;c__Gammaproteobacteria;o__Xanthomonadales;f__norank_o__Xanthomonadales;g__norank_f__norank_o__Xanthomonadales | 0 | 0 | 0 | 0 | 4.53E-05 | 0 | 0 | 0 | 0 | 0 | 7.43E-05 | 2.57E-05 |
| d__Bacteria;k__norank_d__Bacteria;p__Proteobacteria;c__Alphaproteobacteria;o__Rhodospirillales;f__Magnetospirillaceae;g__Magnetospirillum | 0 | 0 | 0 | 0 | 6.79E-05 | 0 | 0 | 0 | 0 | 0 | 0 | 7.72E-05 |
| d__Bacteria;k__norank_d__Bacteria;p__Firmicutes;c__Moorellia;o__Desulfitibacterales;f__Desulfitibacteraceae;g__Desulfitibacter | 2.62E-05 | 5.13E-05 | 0 | 4.29E-05 | 0 | 0 | 0 | 0 | 0 | 2.40E-05 | 0 | 0 |
| d__Bacteria;k__norank_d__Bacteria;p__Firmicutes;c__Desulfotomaculia;o__Desulfotomaculales;f__norank_o__Desulfotomaculales;g__unclassified_f__norank_o__Desulfotomaculales | 0 | 5.13E-05 | 4.90E-05 | 2.14E-05 | 0 | 2.21E-05 | 0 | 0 | 0 | 0 | 0 | 0 |
| d__Bacteria;k__norank_d__Bacteria;p__Proteobacteria;c__Alphaproteobacteria;o__Azospirillales;f__Azospirillales_Incertae_Sedis;g__Stella | 0 | 0 | 0 | 0 | 2.26E-05 | 2.21E-05 | 0 | 0 | 0 | 4.79E-05 | 2.48E-05 | 2.57E-05 |
| d__Bacteria;k__norank_d__Bacteria;p__Actinobacteriota;c__Actinobacteria;o__Frankiales;f__Cryptosporangiaceae;g__Cryptosporangium | 0 | 0 | 0 | 2.14E-05 | 0 | 4.42E-05 | 2.61E-05 | 0 | 2.56E-05 | 0 | 2.48E-05 | 0 |
| d__Bacteria;k__norank_d__Bacteria;p__Proteobacteria;c__Gammaproteobacteria;o__Burkholderiales;f__Rhodocyclaceae;g__norank_f__Rhodocyclaceae | 2.62E-05 | 0 | 2.45E-05 | 4.29E-05 | 2.26E-05 | 2.21E-05 | 0 | 0 | 0 | 0 | 0 | 0 |
| d__Bacteria;k__norank_d__Bacteria;p__Actinobacteriota;c__Actinobacteria;o__Euzebyales;f__Euzebyaceae;g__Euzebya | 0 | 0 | 0 | 0 | 0.000113173 | 0 | 0 | 0 | 0 | 0 | 2.48E-05 | 0 |
| d__Bacteria;k__norank_d__Bacteria;p__Firmicutes;c__Clostridia;o__Oscillospirales;f__Oscillospiraceae;g__unclassified_f__Oscillospiraceae | 2.62E-05 | 0 | 0 | 6.43E-05 | 4.53E-05 | 0 | 0 | 0 | 0 | 0 | 0 | 0 |
| d__Bacteria;k__norank_d__Bacteria;p__Firmicutes;c__Thermacetogenia;o__Thermacetogeniales;f__Thermacetogeniaceae;g__unclassified_f__Thermacetogeniaceae | 0 | 0 | 0 | 0 | 6.79E-05 | 6.63E-05 | 0 | 0 | 0 | 0 | 0 | 0 |
| d__Bacteria;k__norank_d__Bacteria;p__Bacteroidota;c__Bacteroidia;o__Sphingobacteriales;f__Sphingobacteriaceae;g__Pseudosphingobacterium | 0 | 0 | 0 | 2.14E-05 | 9.05E-05 | 2.21E-05 | 0 | 0 | 0 | 0 | 0 | 0 |
| d__Bacteria;k__norank_d__Bacteria;p__Firmicutes;c__Negativicutes;o__Veillonellales-Selenomonadales;f__norank_o__Veillonellales-Selenomonadales;g__norank_f__norank_o__Veillonellales-Selenomonadales | 0 | 0 | 0 | 8.58E-05 | 0 | 2.21E-05 | 0 | 0 | 2.56E-05 | 0 | 0 | 0 |
| d__Bacteria;k__norank_d__Bacteria;p__Actinobacteriota;c__Acidimicrobiia;o__Microtrichales;f__Ilumatobacteraceae;g__unclassified_f__Ilumatobacteraceae | 0.000131216 | 0 | 0 | 0 | 0 | 0 | 0 | 0 | 0 | 0 | 0 | 0 |
| d__Bacteria;k__norank_d__Bacteria;p__Cloacimonadota;c__Cloacimonadia;o__Cloacimonadales;f__norank_o__Cloacimonadales;g__norank_f__norank_o__Cloacimonadales | 0.000131216 | 0 | 0 | 0 | 0 | 0 | 0 | 0 | 0 | 0 | 0 | 0 |
| d__Bacteria;k__norank_d__Bacteria;p__Proteobacteria;c__Gammaproteobacteria;o__Salinisphaerales;f__Solimonadaceae;g__unclassified_f__Solimonadaceae | 0.000104973 | 0 | 0 | 0 | 0 | 0 | 0 | 0 | 0 | 0 | 0 | 2.57E-05 |
| d__Bacteria;k__norank_d__Bacteria;p__Proteobacteria;c__Gammaproteobacteria;o__Burkholderiales;f__Rhodocyclaceae;g__Sterolibacterium | 0 | 0 | 0 | 0 | 0 | 0 | 0.000104531 | 0 | 2.56E-05 | 0 | 0 | 0 |
| d__Bacteria;k__norank_d__Bacteria;p__Firmicutes;c__Clostridia;o__Monoglobales;f__Monoglobaceae;g__Monoglobus | 7.87E-05 | 0 | 0 | 0 | 0 | 0 | 2.61E-05 | 0 | 0 | 0 | 2.48E-05 | 0 |
| d__Bacteria;k__norank_d__Bacteria;p__Actinobacteriota;c__Actinobacteria;o__Propionibacteriales;f__Propionibacteriaceae;g__Aestuariimicrobium | 0 | 0 | 0 | 0 | 0 | 0 | 0 | 0.000103721 | 2.56E-05 | 0 | 0 | 0 |
| d__Bacteria;k__norank_d__Bacteria;p__Firmicutes;c__Bacilli;o__Bacillales;f__Sporolactobacillaceae;g__Tuberibacillus | 0 | 0 | 0 | 0 | 0 | 0 | 2.61E-05 | 0 | 5.12E-05 | 0 | 0 | 5.15E-05 |
| d__Bacteria;k__norank_d__Bacteria;p__Chloroflexi;c__Chloroflexia;o__Chloroflexales;f__unclassified_o__Chloroflexales;g__unclassified_o__Chloroflexales | 0 | 0 | 0 | 0 | 0 | 0 | 0 | 0 | 0 | 0 | 0 | 0.000128684 |
| d__Bacteria;k__norank_d__Bacteria;p__Proteobacteria;c__Alphaproteobacteria;o__Rhizobiales;f__Beijerinckiaceae;g__norank_f__Beijerinckiaceae | 0 | 0 | 0 | 0 | 0 | 0 | 0 | 5.19E-05 | 7.68E-05 | 0 | 0 | 0 |
| d__Bacteria;k__norank_d__Bacteria;p__Proteobacteria;c__Alphaproteobacteria;o__Azospirillales;f__Azospirillaceae;g__Azospirillum | 0 | 5.13E-05 | 0 | 0 | 0 | 0 | 0 | 2.59E-05 | 2.56E-05 | 0 | 0 | 2.57E-05 |
| d__Bacteria;k__norank_d__Bacteria;p__Proteobacteria;c__Gammaproteobacteria;o__211ds20;f__norank_o__211ds20;g__norank_f__norank_o__211ds20 | 0 | 0 | 0 | 0 | 0 | 0 | 0 | 2.59E-05 | 0.000102404 | 0 | 0 | 0 |
| d__Bacteria;k__norank_d__Bacteria;p__Firmicutes;c__Clostridia;o__Clostridiales;f__Clostridiaceae;g__Clostridium_sensu_stricto_2 | 5.25E-05 | 5.13E-05 | 2.45E-05 | 0 | 0 | 0 | 0 | 0 | 0 | 0 | 0 | 0 |
| d__Bacteria;k__norank_d__Bacteria;p__Cyanobacteria;c__Vampirivibrionia;o__Vampirovibrionales;f__Vampirovibrionaceae;g__unclassified_f__Vampirovibrionaceae | 0 | 0.000128284 | 0 | 0 | 0 | 0 | 0 | 0 | 0 | 0 | 0 | 0 |
| d__Bacteria;k__norank_d__Bacteria;p__Patescibacteria;c__Parcubacteria;o__Candidatus_Staskawiczbacteria;f__norank_o__Candidatus_Staskawiczbacteria;g__norank_f__norank_o__Candidatus_Staskawiczbacteria | 0 | 0.000128284 | 0 | 0 | 0 | 0 | 0 | 0 | 0 | 0 | 0 | 0 |
| d__Bacteria;k__norank_d__Bacteria;p__Bacteroidota;c__Bacteroidia;o__Cytophagales;f__Cyclobacteriaceae;g__unclassified_f__Cyclobacteriaceae | 0 | 0 | 0 | 0 | 0 | 0 | 0 | 0 | 0.000128005 | 0 | 0 | 0 |
| d__Bacteria;k__norank_d__Bacteria;p__Firmicutes;c__Clostridia;o__Lachnospirales;f__Lachnospiraceae;g__Roseburia | 2.62E-05 | 7.70E-05 | 2.45E-05 | 0 | 0 | 0 | 0 | 0 | 0 | 0 | 0 | 0 |
| d__Bacteria;k__norank_d__Bacteria;p__Latescibacterota;c__Latescibacteria;o__Latescibacterales;f__Latescibacteraceae;g__Candidatus_Latescibacter | 5.25E-05 | 2.57E-05 | 4.90E-05 | 0 | 0 | 0 | 0 | 0 | 0 | 0 | 0 | 0 |
| d__Bacteria;k__norank_d__Bacteria;p__Armatimonadota;c__Fimbriimonadia;o__Fimbriimonadales;f__norank_o__Fimbriimonadales;g__norank_f__norank_o__Fimbriimonadales | 0 | 0 | 0 | 0 | 2.26E-05 | 0 | 7.84E-05 | 0 | 0 | 0 | 0 | 2.57E-05 |
| d__Bacteria;k__norank_d__Bacteria;p__Proteobacteria;c__Alphaproteobacteria;o__Azospirillales;f__Azospirillaceae;g__norank_f__Azospirillaceae | 7.87E-05 | 0 | 0 | 0 | 0 | 2.21E-05 | 0 | 2.59E-05 | 0 | 0 | 0 | 0 |
| d__Bacteria;k__norank_d__Bacteria;p__Proteobacteria;c__Alphaproteobacteria;o__Rickettsiales;f__norank_o__Rickettsiales;g__Candidatus_Jidaibacter | 0 | 0 | 4.90E-05 | 0 | 0 | 0 | 2.61E-05 | 0 | 0 | 0 | 0 | 5.15E-05 |
| d__Bacteria;k__norank_d__Bacteria;p__Firmicutes;c__Clostridia;o__Oscillospirales;f__unclassified_o__Oscillospirales;g__unclassified_o__Oscillospirales | 5.25E-05 | 0 | 7.36E-05 | 0 | 0 | 0 | 0 | 0 | 0 | 0 | 0 | 0 |
| d__Bacteria;k__norank_d__Bacteria;p__Proteobacteria;c__Gammaproteobacteria;o__KF-JG30-C25;f__norank_o__KF-JG30-C25;g__norank_f__norank_o__KF-JG30-C25 | 0 | 7.70E-05 | 4.90E-05 | 0 | 0 | 0 | 0 | 0 | 0 | 0 | 0 | 0 |
| d__Bacteria;k__norank_d__Bacteria;p__Acidobacteriota;c__Acidobacteriae;o__Subgroup_12;f__norank_o__Subgroup_12;g__norank_f__norank_o__Subgroup_12 | 7.87E-05 | 0 | 2.45E-05 | 0 | 2.26E-05 | 0 | 0 | 0 | 0 | 0 | 0 | 0 |
| d__Bacteria;k__norank_d__Bacteria;p__Firmicutes;c__Negativicutes;o__Veillonellales-Selenomonadales;f__Sporomusaceae;g__unclassified_f__Sporomusaceae | 0 | 2.57E-05 | 0 | 0 | 0 | 2.21E-05 | 5.23E-05 | 0 | 0 | 0 | 0 | 2.57E-05 |
| d__Bacteria;k__norank_d__Bacteria;p__Proteobacteria;c__Alphaproteobacteria;o__Rickettsiales;f__Rickettsiaceae;g__norank_f__Rickettsiaceae | 2.62E-05 | 0 | 2.45E-05 | 0 | 2.26E-05 | 0 | 5.23E-05 | 0 | 0 | 0 | 0 | 0 |
| d__Bacteria;k__norank_d__Bacteria;p__Myxococcota;c__Polyangia;o__Polyangiales;f__Polyangiaceae;g__Labilithrix | 2.62E-05 | 2.57E-05 | 0 | 0 | 0 | 0 | 0 | 0 | 0 | 4.79E-05 | 0 | 2.57E-05 |
| d__Bacteria;k__norank_d__Bacteria;p__Spirochaetota;c__Leptospirae;o__Leptospirales;f__Leptospiraceae;g__Turneriella | 2.62E-05 | 2.57E-05 | 7.36E-05 | 0 | 0 | 0 | 0 | 0 | 0 | 0 | 0 | 0 |
| d__Bacteria;k__norank_d__Bacteria;p__Fibrobacterota;c__Chitinivibrionia;o__norank_c__Chitinivibrionia;f__norank_o__norank_c__Chitinivibrionia;g__norank_f__norank_o__norank_c__Chitinivibrionia | 5.25E-05 | 0 | 2.45E-05 | 0 | 0 | 0 | 0 | 0 | 0 | 4.79E-05 | 0 | 0 |
| d__Bacteria;k__norank_d__Bacteria;p__Proteobacteria;c__Gammaproteobacteria;o__Oceanospirillales;f__Halomonadaceae;g__Halomonas | 0 | 5.13E-05 | 0 | 0 | 0 | 2.21E-05 | 0 | 0 | 5.12E-05 | 0 | 0 | 0 |
| d__Bacteria;k__norank_d__Bacteria;p__Cyanobacteria;c__Cyanobacteriia;o__Oxyphotobacteria_Incertae_Sedis;f__unclassified_o__Oxyphotobacteria_Incertae_Sedis;g__Leptolyngbya_EcFYyyy-00 | 0 | 0 | 0 | 0 | 0 | 0 | 0 | 0 | 0 | 0 | 0.000123857 | 0 |
| d__Bacteria;k__norank_d__Bacteria;p__Proteobacteria;c__Alphaproteobacteria;o__Sphingomonadales;f__Sphingomonadaceae;g__Porphyrobacter | 2.62E-05 | 5.13E-05 | 0 | 2.14E-05 | 0 | 0 | 0 | 0 | 0 | 0 | 2.48E-05 | 0 |
| d__Bacteria;k__norank_d__Bacteria;p__Chloroflexi;c__Chloroflexia;o__Thermomicrobiales;f__Thermomicrobiaceae;g__unclassified_f__Thermomicrobiaceae | 0 | 0 | 0 | 0 | 2.26E-05 | 0 | 0 | 2.59E-05 | 5.12E-05 | 2.40E-05 | 0 | 0 |
| d__Bacteria;k__norank_d__Bacteria;p__Myxococcota;c__unclassified_p__Myxococcota;o__unclassified_p__Myxococcota;f__unclassified_p__Myxococcota;g__unclassified_p__Myxococcota | 5.25E-05 | 2.57E-05 | 0 | 0 | 4.53E-05 | 0 | 0 | 0 | 0 | 0 | 0 | 0 |
| d__Bacteria;k__norank_d__Bacteria;p__Verrucomicrobiota;c__unclassified_p__Verrucomicrobiota;o__unclassified_p__Verrucomicrobiota;f__unclassified_p__Verrucomicrobiota;g__unclassified_p__Verrucomicrobiota | 0 | 0 | 0.000122612 | 0 | 0 | 0 | 0 | 0 | 0 | 0 | 0 | 0 |
| d__Bacteria;k__norank_d__Bacteria;p__Proteobacteria;c__Alphaproteobacteria;o__Rickettsiales;f__Rickettsiaceae;g__Rickettsia | 0 | 0 | 4.90E-05 | 2.14E-05 | 0 | 0 | 0 | 5.19E-05 | 0 | 0 | 0 | 0 |
| d__Bacteria;k__norank_d__Bacteria;p__Bacteroidota;c__Bacteroidia;o__Bacteroidetes_VC2.1_Bac22;f__norank_o__Bacteroidetes_VC2.1_Bac22;g__norank_f__norank_o__Bacteroidetes_VC2.1_Bac22 | 0 | 0 | 2.45E-05 | 0 | 2.26E-05 | 0 | 0 | 0 | 2.56E-05 | 0 | 4.95E-05 | 0 |
| d__Bacteria;k__norank_d__Bacteria;p__Verrucomicrobiota;c__Chlamydiae;o__Chlamydiales;f__Criblamydiaceae;g__Estrella | 0 | 2.57E-05 | 7.36E-05 | 0 | 2.26E-05 | 0 | 0 | 0 | 0 | 0 | 0 | 0 |
| d__Bacteria;k__norank_d__Bacteria;p__Proteobacteria;c__Alphaproteobacteria;o__Sneathiellales;f__Sneathiellaceae;g__norank_f__Sneathiellaceae | 0 | 0 | 0 | 2.14E-05 | 2.26E-05 | 0 | 5.23E-05 | 0 | 0 | 0 | 2.48E-05 | 0 |
| d__Bacteria;k__norank_d__Bacteria;p__Firmicutes;c__Bacilli;o__Bacillales;f__Bacillaceae;g__Gracilibacillus | 0 | 0 | 0 | 2.14E-05 | 0 | 0 | 0 | 0 | 5.12E-05 | 4.79E-05 | 0 | 0 |
| d__Bacteria;k__norank_d__Bacteria;p__Proteobacteria;c__Gammaproteobacteria;o__Diplorickettsiales;f__Diplorickettsiaceae;g__unclassified_f__Diplorickettsiaceae | 0 | 0 | 4.90E-05 | 0 | 4.53E-05 | 0 | 2.61E-05 | 0 | 0 | 0 | 0 | 0 |
| d__Bacteria;k__norank_d__Bacteria;p__Cyanobacteria;c__Cyanobacteriia;o__Oxyphotobacteria_Incertae_Sedis;f__unclassified_o__Oxyphotobacteria_Incertae_Sedis;g__Phormidium_SAG_37.90 | 0 | 7.70E-05 | 0 | 4.29E-05 | 0 | 0 | 0 | 0 | 0 | 0 | 0 | 0 |
| d__Bacteria;k__norank_d__Bacteria;p__Firmicutes;c__Clostridia;o__Lachnospirales;f__Lachnospiraceae;g__Tyzzerella | 2.62E-05 | 0 | 0 | 0 | 4.53E-05 | 0 | 0 | 0 | 0 | 4.79E-05 | 0 | 0 |
| d__Bacteria;k__norank_d__Bacteria;p__Firmicutes;c__Negativicutes;o__Veillonellales-Selenomonadales;f__Sporomusaceae;g__Anaerospora | 0 | 0 | 0 | 0 | 2.26E-05 | 4.42E-05 | 5.23E-05 | 0 | 0 | 0 | 0 | 0 |
| d__Bacteria;k__norank_d__Bacteria;p__Bdellovibrionota;c__Oligoflexia;o__Silvanigrellales;f__Silvanigrellaceae;g__unclassified_f__Silvanigrellaceae | 0 | 0 | 4.90E-05 | 4.29E-05 | 0 | 0 | 2.61E-05 | 0 | 0 | 0 | 0 | 0 |
| d__Bacteria;k__norank_d__Bacteria;p__Proteobacteria;c__Gammaproteobacteria;o__Burkholderiales;f__Burkholderiaceae;g__Ralstonia | 0 | 0 | 0 | 0 | 0 | 6.63E-05 | 0 | 0 | 5.12E-05 | 0 | 0 | 0 |
| d__Bacteria;k__norank_d__Bacteria;p__Proteobacteria;c__Alphaproteobacteria;o__Ferrovibrionales;f__Ferrovibrionaceae;g__Ferrovibrio | 0 | 0 | 0 | 4.29E-05 | 0 | 2.21E-05 | 0 | 0 | 5.12E-05 | 0 | 0 | 0 |
| d__Bacteria;k__norank_d__Bacteria;p__Firmicutes;c__Clostridia;o__Peptostreptococcales-Tissierellales;f__Peptostreptococcaceae;g__Clostridioides | 0 | 0 | 0 | 0 | 4.53E-05 | 2.21E-05 | 0 | 0 | 0 | 4.79E-05 | 0 | 0 |
| d__Bacteria;k__norank_d__Bacteria;p__Cyanobacteria;c__Vampirivibrionia;o__Gastranaerophilales;f__norank_o__Gastranaerophilales;g__norank_f__norank_o__Gastranaerophilales | 2.62E-05 | 0 | 0 | 4.29E-05 | 4.53E-05 | 0 | 0 | 0 | 0 | 0 | 0 | 0 |
| d__Bacteria;k__norank_d__Bacteria;p__Bacteroidota;c__Ignavibacteria;o__Ignavibacteriales;f__BSV40;g__norank_f__BSV40 | 0 | 0 | 2.45E-05 | 0 | 4.53E-05 | 4.42E-05 | 0 | 0 | 0 | 0 | 0 | 0 |
| d__Bacteria;k__norank_d__Bacteria;p__Desulfobacterota;c__Desulfuromonadia;o__MA-28-I98C;f__norank_o__MA-28-I98C;g__norank_f__norank_o__MA-28-I98C | 0 | 0 | 0 | 0 | 0.000113173 | 0 | 0 | 0 | 0 | 0 | 0 | 0 |
| d__Bacteria;k__norank_d__Bacteria;p__Proteobacteria;c__Gammaproteobacteria;o__EPR3968-O8a-Bc78;f__norank_o__EPR3968-O8a-Bc78;g__norank_f__norank_o__EPR3968-O8a-Bc78 | 0 | 0 | 0 | 0 | 9.05E-05 | 2.21E-05 | 0 | 0 | 0 | 0 | 0 | 0 |
| d__Bacteria;k__norank_d__Bacteria;p__Chloroflexi;c__Chloroflexia;o__Chloroflexales;f__Chloroflexaceae;g__Chloronema | 0 | 0 | 0 | 8.58E-05 | 2.26E-05 | 0 | 0 | 0 | 0 | 0 | 0 | 0 |
| d__Bacteria;k__norank_d__Bacteria;p__Bacteroidota;c__Bacteroidia;o__Cytophagales;f__unclassified_o__Cytophagales;g__unclassified_o__Cytophagales | 0.000104973 | 0 | 0 | 0 | 0 | 0 | 0 | 0 | 0 | 0 | 0 | 0 |
| d__Bacteria;k__norank_d__Bacteria;p__Proteobacteria;c__Gammaproteobacteria;o__Cellvibrionales;f__Porticoccaceae;g__Porticoccus | 5.25E-05 | 0 | 0 | 0 | 0 | 0 | 5.23E-05 | 0 | 0 | 0 | 0 | 0 |
| d__Bacteria;k__norank_d__Bacteria;p__Acidobacteriota;c__Acidobacteriae;o__Acidobacteriales;f__Acidobacteriaceae_Subgroup_1;g__Terriglobus | 0 | 0 | 0 | 0 | 0 | 0 | 0.000104531 | 0 | 0 | 0 | 0 | 0 |
| d__Bacteria;k__norank_d__Bacteria;p__Proteobacteria;c__Gammaproteobacteria;o__HOC36;f__norank_o__HOC36;g__norank_f__norank_o__HOC36 | 7.87E-05 | 2.57E-05 | 0 | 0 | 0 | 0 | 0 | 0 | 0 | 0 | 0 | 0 |
| d__Bacteria;k__norank_d__Bacteria;p__Chloroflexi;c__Ktedonobacteria;o__B10-SB3A;f__norank_o__B10-SB3A;g__norank_f__norank_o__B10-SB3A | 2.62E-05 | 2.57E-05 | 0 | 0 | 0 | 0 | 0 | 5.19E-05 | 0 | 0 | 0 | 0 |
| d__Bacteria;k__norank_d__Bacteria;p__Actinobacteriota;c__Actinobacteria;o__Corynebacteriales;f__Nocardiaceae;g__Gordonia | 0 | 0 | 0 | 0 | 0 | 0 | 0 | 0.000103721 | 0 | 0 | 0 | 0 |
| d__Bacteria;k__norank_d__Bacteria;p__Cyanobacteria;c__Cyanobacteriia;o__Cyanobacteriales;f__Nostocaceae;g__Anabaena_PCC-7108 | 5.25E-05 | 0 | 0 | 0 | 0 | 0 | 0 | 0 | 5.12E-05 | 0 | 0 | 0 |
| d__Bacteria;k__norank_d__Bacteria;p__Actinobacteriota;c__Coriobacteriia;o__CG2-30-50-142;f__norank_o__CG2-30-50-142;g__norank_f__norank_o__CG2-30-50-142 | 7.87E-05 | 0 | 0 | 0 | 0 | 0 | 0 | 0 | 0 | 0 | 2.48E-05 | 0 |
| d__Bacteria;k__norank_d__Bacteria;p__Proteobacteria;c__Alphaproteobacteria;o__Rhizobiales;f__Beijerinckiaceae;g__Psychroglaciecola | 0 | 2.57E-05 | 0 | 0 | 0 | 0 | 2.61E-05 | 2.59E-05 | 2.56E-05 | 0 | 0 | 0 |
| d__Bacteria;k__norank_d__Bacteria;p__Firmicutes;c__Clostridia;o__Clostridiales;f__Clostridiaceae;g__Clostridium_sensu_stricto_16 | 5.25E-05 | 0 | 2.45E-05 | 0 | 0 | 0 | 2.61E-05 | 0 | 0 | 0 | 0 | 0 |
| d__Bacteria;k__norank_d__Bacteria;p__Verrucomicrobiota;c__Chlamydiae;o__Chlamydiales;f__Criblamydiaceae;g__Criblamydia | 0 | 0 | 0 | 0 | 0 | 0 | 0 | 0 | 5.12E-05 | 0 | 0 | 5.15E-05 |
| d__Bacteria;k__norank_d__Bacteria;p__Proteobacteria;c__Gammaproteobacteria;o__Burkholderiales;f__Neisseriaceae;g__norank_f__Neisseriaceae | 0 | 5.13E-05 | 0 | 0 | 0 | 0 | 0 | 0 | 5.12E-05 | 0 | 0 | 0 |
| d__Bacteria;k__norank_d__Bacteria;p__Gemmatimonadota;c__Longimicrobia;o__Longimicrobiales;f__Longimicrobiaceae;g__unclassified_f__Longimicrobiaceae | 0 | 0 | 0 | 0 | 0 | 0 | 2.61E-05 | 0 | 5.12E-05 | 0 | 2.48E-05 | 0 |
| d__Bacteria;k__norank_d__Bacteria;p__Firmicutes;c__Bacilli;o__Bacillales;f__Bacillaceae;g__Halobacillus | 2.62E-05 | 0 | 0 | 0 | 0 | 0 | 2.61E-05 | 0 | 0 | 0 | 4.95E-05 | 0 |
| d__Bacteria;k__norank_d__Bacteria;p__Chloroflexi;c__Ktedonobacteria;o__Ktedonobacterales;f__Ktedonobacteraceae;g__1921-2 | 0 | 2.57E-05 | 2.45E-05 | 0 | 0 | 0 | 0 | 0 | 5.12E-05 | 0 | 0 | 0 |
| d__Bacteria;k__norank_d__Bacteria;p__Proteobacteria;c__Alphaproteobacteria;o__Acetobacterales;f__Acetobacteraceae;g__Rhodovastum | 0 | 2.57E-05 | 0 | 0 | 0 | 0 | 2.61E-05 | 0 | 0 | 0 | 4.95E-05 | 0 |
| d__Bacteria;k__norank_d__Bacteria;p__Actinobacteriota;c__Actinobacteria;o__Streptosporangiales;f__Streptosporangiaceae;g__Herbidospora | 0 | 0 | 4.90E-05 | 0 | 0 | 0 | 5.23E-05 | 0 | 0 | 0 | 0 | 0 |
| d__Bacteria;k__norank_d__Bacteria;p__Bacteroidota;c__Bacteroidia;o__Flavobacteriales;f__Crocinitomicaceae;g__Fluviicola | 2.62E-05 | 0 | 0 | 0 | 0 | 0 | 0 | 0 | 0 | 0 | 7.43E-05 | 0 |
| d__Bacteria;k__norank_d__Bacteria;p__Verrucomicrobiota;c__Verrucomicrobiae;o__unclassified_c__Verrucomicrobiae;f__unclassified_c__Verrucomicrobiae;g__unclassified_c__Verrucomicrobiae | 0 | 5.13E-05 | 4.90E-05 | 0 | 0 | 0 | 0 | 0 | 0 | 0 | 0 | 0 |
| d__Bacteria;k__norank_d__Bacteria;p__Cyanobacteria;c__Cyanobacteriia;o__Oxyphotobacteria_Incertae_Sedis;f__unclassified_o__Oxyphotobacteria_Incertae_Sedis;g__Oscillatoria_SAG_1459-8 | 0 | 0 | 0 | 0 | 0 | 0 | 0 | 0 | 0 | 0 | 7.43E-05 | 2.57E-05 |
| d__Bacteria;k__norank_d__Bacteria;p__Cyanobacteria;c__Vampirivibrionia;o__Vampirovibrionales;f__unclassified_o__Vampirovibrionales;g__unclassified_o__Vampirovibrionales | 0 | 0 | 2.45E-05 | 0 | 0 | 0 | 0 | 0 | 2.56E-05 | 2.40E-05 | 0 | 2.57E-05 |
| d__Bacteria;k__norank_d__Bacteria;p__Proteobacteria;c__Gammaproteobacteria;o__Burkholderiales;f__Rhodocyclaceae;g__Dechloromonas | 0 | 5.13E-05 | 2.45E-05 | 0 | 0 | 0 | 0 | 0 | 0 | 2.40E-05 | 0 | 0 |
| d__Bacteria;k__norank_d__Bacteria;p__Elusimicrobiota;c__Lineage_IIc;o__norank_c__Lineage_IIc;f__norank_o__norank_c__Lineage_IIc;g__norank_f__norank_o__norank_c__Lineage_IIc | 2.62E-05 | 0 | 0 | 0 | 0 | 0 | 0 | 0 | 2.56E-05 | 4.79E-05 | 0 | 0 |
| d__Bacteria;k__norank_d__Bacteria;p__Patescibacteria;c__Microgenomatia;o__Candidatus_Collierbacteria;f__norank_o__Candidatus_Collierbacteria;g__norank_f__norank_o__Candidatus_Collierbacteria | 0 | 0 | 0 | 0 | 0 | 0 | 0 | 0 | 0 | 4.79E-05 | 0 | 5.15E-05 |
| d__Bacteria;k__norank_d__Bacteria;p__Verrucomicrobiota;c__Verrucomicrobiae;o__Pedosphaerales;f__Pedosphaeraceae;g__Oikopleura | 0 | 2.57E-05 | 7.36E-05 | 0 | 0 | 0 | 0 | 0 | 0 | 0 | 0 | 0 |
| d__Bacteria;k__norank_d__Bacteria;p__Planctomycetota;c__Planctomycetes;o__Pirellulales;f__Pirellulaceae;g__norank_f__Pirellulaceae | 2.62E-05 | 2.57E-05 | 2.45E-05 | 0 | 2.26E-05 | 0 | 0 | 0 | 0 | 0 | 0 | 0 |
| d__Bacteria;k__norank_d__Bacteria;p__Bacteroidota;c__Bacteroidia;o__Flavobacteriales;f__Flavobacteriaceae;g__Zeaxanthinibacter | 0 | 0 | 0 | 0 | 0 | 2.21E-05 | 0 | 0 | 7.68E-05 | 0 | 0 | 0 |
| d__Bacteria;k__norank_d__Bacteria;p__Deinococcota;c__Deinococci;o__Deinococcales;f__Deinococcaceae;g__Deinococcus | 0 | 0 | 0 | 0 | 2.26E-05 | 0 | 5.23E-05 | 0 | 0 | 2.40E-05 | 0 | 0 |
| d__Bacteria;k__norank_d__Bacteria;p__Chloroflexi;c__Dehalococcoidia;o__SAR202_clade;f__norank_o__SAR202_clade;g__norank_f__norank_o__SAR202_clade | 2.62E-05 | 2.57E-05 | 2.45E-05 | 0 | 0 | 2.21E-05 | 0 | 0 | 0 | 0 | 0 | 0 |
| d__Bacteria;k__norank_d__Bacteria;p__Proteobacteria;c__Alphaproteobacteria;o__Rhizobiales;f__Xanthobacteraceae;g__Starkeya | 2.62E-05 | 0 | 0 | 0 | 2.26E-05 | 0 | 0 | 0 | 2.56E-05 | 2.40E-05 | 0 | 0 |
| d__Bacteria;k__norank_d__Bacteria;p__Proteobacteria;c__Alphaproteobacteria;o__Thalassobaculales;f__norank_o__Thalassobaculales;g__norank_f__norank_o__Thalassobaculales | 0 | 0 | 0 | 0 | 2.26E-05 | 0 | 0 | 0 | 0 | 0 | 4.95E-05 | 2.57E-05 |
| d__Bacteria;k__norank_d__Bacteria;p__Actinobacteriota;c__Actinobacteria;o__Micrococcales;f__Micrococcaceae;g__Kocuria | 0 | 0 | 0 | 2.14E-05 | 0 | 0 | 0 | 0 | 2.56E-05 | 0 | 2.48E-05 | 2.57E-05 |
| d__Bacteria;k__norank_d__Bacteria;p__Desulfobacterota;c__Desulfobulbia;o__Desulfobulbales;f__Desulfocapsaceae;g__unclassified_f__Desulfocapsaceae | 5.25E-05 | 0 | 0 | 0 | 2.26E-05 | 2.21E-05 | 0 | 0 | 0 | 0 | 0 | 0 |
| d__Bacteria;k__norank_d__Bacteria;p__Myxococcota;c__Polyangia;o__Polyangiales;f__norank_o__Polyangiales;g__norank_f__norank_o__Polyangiales | 0 | 2.57E-05 | 0 | 0 | 4.53E-05 | 0 | 2.61E-05 | 0 | 0 | 0 | 0 | 0 |
| d__Bacteria;k__norank_d__Bacteria;p__Verrucomicrobiota;c__Kiritimatiellae;o__WCHB1-41;f__norank_o__WCHB1-41;g__norank_f__norank_o__WCHB1-41 | 0 | 0 | 4.90E-05 | 0 | 0 | 0 | 0 | 0 | 0 | 4.79E-05 | 0 | 0 |
| d__Bacteria;k__norank_d__Bacteria;p__Proteobacteria;c__Alphaproteobacteria;o__Acetobacterales;f__Acetobacteraceae;g__Craurococcus-Caldovatus | 0 | 0 | 0 | 0 | 0 | 0 | 0 | 0 | 0 | 7.19E-05 | 2.48E-05 | 0 |
| d__Bacteria;k__norank_d__Bacteria;p__Chloroflexi;c__Chloroflexia;o__Chloroflexales;f__Chloroflexaceae;g__Candidatus_Chloroploca | 0 | 0 | 0 | 0 | 0 | 4.42E-05 | 5.23E-05 | 0 | 0 | 0 | 0 | 0 |
| d__Bacteria;k__norank_d__Bacteria;p__Proteobacteria;c__Alphaproteobacteria;o__Zavarziniales;f__norank_o__Zavarziniales;g__norank_f__norank_o__Zavarziniales | 0 | 0 | 0 | 0 | 2.26E-05 | 2.21E-05 | 2.61E-05 | 0 | 2.56E-05 | 0 | 0 | 0 |
| d__Bacteria;k__norank_d__Bacteria;p__Proteobacteria;c__Gammaproteobacteria;o__Pseudomonadales;f__Moraxellaceae;g__Permianibacter | 0 | 0 | 0 | 0 | 0 | 0 | 0 | 0 | 0 | 9.58E-05 | 0 | 0 |
| d__Bacteria;k__norank_d__Bacteria;p__Nitrospirota;c__Leptospirillia;o__Leptospirillales;f__Leptospirillaceae;g__Leptospirillum | 0 | 0 | 4.90E-05 | 0 | 2.26E-05 | 0 | 0 | 0 | 0 | 2.40E-05 | 0 | 0 |
| d__Bacteria;k__norank_d__Bacteria;p__Desulfobacterota;c__Syntrophobacteria;o__Syntrophobacterales;f__Syntrophobacteraceae;g__unclassified_f__Syntrophobacteraceae | 0 | 0 | 7.36E-05 | 2.14E-05 | 0 | 0 | 0 | 0 | 0 | 0 | 0 | 0 |
| d__Bacteria;k__norank_d__Bacteria;p__Actinobacteriota;c__Actinobacteria;o__Frankiales;f__Geodermatophilaceae;g__Antricoccus | 0 | 0 | 0 | 0 | 6.79E-05 | 0 | 2.61E-05 | 0 | 0 | 0 | 0 | 0 |
| d__Bacteria;k__norank_d__Bacteria;p__Proteobacteria;c__Gammaproteobacteria;o__Burkholderiales;f__Rhodocyclaceae;g__Azospira | 0 | 0 | 0 | 0 | 4.53E-05 | 0 | 0 | 0 | 0 | 4.79E-05 | 0 | 0 |
| d__Bacteria;k__norank_d__Bacteria;p__Acidobacteriota;c__Blastocatellia;o__Blastocatellales;f__Blastocatellaceae;g__OLB17 | 0 | 0 | 0 | 2.14E-05 | 4.53E-05 | 0 | 0 | 0 | 2.56E-05 | 0 | 0 | 0 |
| d__Bacteria;k__norank_d__Bacteria;p__Proteobacteria;c__Gammaproteobacteria;o__Burkholderiales;f__Oxalobacteraceae;g__s3t2d-1089 | 0 | 0 | 0 | 0 | 0 | 6.63E-05 | 0 | 0 | 0 | 0 | 2.48E-05 | 0 |
| d__Bacteria;k__norank_d__Bacteria;p__Actinobacteriota;c__Actinobacteria;o__Pseudonocardiales;f__Pseudonocardiaceae;g__Kibdelosporangium | 0 | 0 | 0 | 0 | 9.05E-05 | 0 | 0 | 0 | 0 | 0 | 0 | 0 |
| d__Bacteria;k__norank_d__Bacteria;p__Chloroflexi;c__Anaerolineae;o__SBR1031;f__unclassified_o__SBR1031;g__unclassified_o__SBR1031 | 0 | 0 | 0 | 0 | 9.05E-05 | 0 | 0 | 0 | 0 | 0 | 0 | 0 |
| d__Bacteria;k__norank_d__Bacteria;p__Actinobacteriota;c__Actinobacteria;o__Propionibacteriales;f__Propionibacteriaceae;g__Friedmanniella | 0 | 0 | 0 | 2.14E-05 | 6.79E-05 | 0 | 0 | 0 | 0 | 0 | 0 | 0 |
| d__Bacteria;k__norank_d__Bacteria;p__Firmicutes;c__Clostridia;o__Peptostreptococcales-Tissierellales;f__Anaerovoracaceae;g__Family_XIII_AD3011_group | 0 | 0 | 0 | 4.29E-05 | 0 | 4.42E-05 | 0 | 0 | 0 | 0 | 0 | 0 |
| d__Bacteria;k__norank_d__Bacteria;p__Proteobacteria;c__Alphaproteobacteria;o__Caulobacterales;f__Hyphomonadaceae;g__Hyphomonas | 0 | 0 | 0 | 4.29E-05 | 0 | 4.42E-05 | 0 | 0 | 0 | 0 | 0 | 0 |
| d__Bacteria;k__norank_d__Bacteria;p__Firmicutes;c__Clostridia;o__Lachnospirales;f__Defluviitaleaceae;g__Defluviitalea | 7.87E-05 | 0 | 0 | 0 | 0 | 0 | 0 | 0 | 0 | 0 | 0 | 0 |
| d__Bacteria;k__norank_d__Bacteria;p__Proteobacteria;c__Gammaproteobacteria;o__Xanthomonadales;f__Rhodanobacteraceae;g__Rudaea | 7.87E-05 | 0 | 0 | 0 | 0 | 0 | 0 | 0 | 0 | 0 | 0 | 0 |
| d__Bacteria;k__norank_d__Bacteria;p__Proteobacteria;c__Gammaproteobacteria;o__Burkholderiales;f__Rhodocyclaceae;g__Propionivibrio | 5.25E-05 | 0 | 0 | 0 | 0 | 0 | 0 | 2.59E-05 | 0 | 0 | 0 | 0 |
| d__Bacteria;k__norank_d__Bacteria;p__Firmicutes;c__Bacilli;o__Thermicanales;f__Thermicanaceae;g__Thermicanus | 0 | 0 | 0 | 0 | 0 | 0 | 7.84E-05 | 0 | 0 | 0 | 0 | 0 |
| d__Bacteria;k__norank_d__Bacteria;p__Actinobacteriota;c__Actinobacteria;o__Streptomycetales;f__Streptomycetaceae;g__Kitasatospora | 0 | 0 | 0 | 0 | 0 | 0 | 2.61E-05 | 5.19E-05 | 0 | 0 | 0 | 0 |
| d__Bacteria;k__norank_d__Bacteria;p__Firmicutes;c__Bacilli;o__Lactobacillales;f__Streptococcaceae;g__Lactococcus | 0 | 0 | 0 | 0 | 0 | 0 | 0 | 7.78E-05 | 0 | 0 | 0 | 0 |
| d__Bacteria;k__norank_d__Bacteria;p__Firmicutes;c__Bacilli;o__Thermoactinomycetales;f__Thermoactinomycetaceae;g__Seinonella | 0 | 0 | 0 | 0 | 0 | 0 | 0 | 7.78E-05 | 0 | 0 | 0 | 0 |
| d__Bacteria;k__norank_d__Bacteria;p__Firmicutes;c__Desulfotomaculia;o__Desulfotomaculales;f__norank_o__Desulfotomaculales;g__Desulfurispora | 2.62E-05 | 5.13E-05 | 0 | 0 | 0 | 0 | 0 | 0 | 0 | 0 | 0 | 0 |
| d__Bacteria;k__norank_d__Bacteria;p__Proteobacteria;c__Alphaproteobacteria;o__Rickettsiales;f__Rickettsiaceae;g__Candidatus_Megaira | 0 | 0 | 0 | 0 | 0 | 0 | 0 | 2.59E-05 | 0 | 0 | 0 | 5.15E-05 |
| d__Bacteria;k__norank_d__Bacteria;p__Actinobacteriota;c__Actinobacteria;o__Streptosporangiales;f__Thermomonosporaceae;g__Thermostaphylospora | 0 | 0 | 0 | 0 | 0 | 0 | 0 | 0 | 0 | 0 | 0 | 7.72E-05 |
| d__Bacteria;k__norank_d__Bacteria;p__Cyanobacteria;c__Cyanobacteriia;o__Leptolyngbyales;f__Leptolyngbyaceae;g__Leptolyngbya_Es-Yyy1000 | 0 | 0 | 0 | 0 | 0 | 0 | 0 | 0 | 0 | 0 | 0 | 7.72E-05 |
| d__Bacteria;k__norank_d__Bacteria;p__Proteobacteria;c__Gammaproteobacteria;o__Methylococcales;f__Methylococcaceae;g__Methylocaldum | 0 | 0 | 0 | 0 | 0 | 0 | 0 | 0 | 0 | 0 | 0 | 7.72E-05 |
| d__Bacteria;k__norank_d__Bacteria;p__Proteobacteria;c__Alphaproteobacteria;o__Rhizobiales;f__Pleomorphomonadaceae;g__unclassified_f__Pleomorphomonadaceae | 0 | 0 | 0 | 0 | 0 | 0 | 0 | 2.59E-05 | 5.12E-05 | 0 | 0 | 0 |
| d__Bacteria;k__norank_d__Bacteria;p__Desulfobacterota;c__Desulfuromonadia;o__Geobacterales;f__unclassified_o__Geobacterales;g__unclassified_o__Geobacterales | 0 | 7.70E-05 | 0 | 0 | 0 | 0 | 0 | 0 | 0 | 0 | 0 | 0 |
| d__Bacteria;k__norank_d__Bacteria;p__Firmicutes;c__Clostridia;o__Oscillospirales;f__Ethanoligenenaceae;g__Acetanaerobacterium | 0 | 0 | 0 | 0 | 0 | 0 | 0 | 0 | 7.68E-05 | 0 | 0 | 0 |
| d__Bacteria;k__norank_d__Bacteria;p__Proteobacteria;c__Alphaproteobacteria;o__Rhizobiales;f__Xanthobacteraceae;g__Nitrobacter | 0 | 0 | 0 | 0 | 0 | 0 | 0 | 0 | 7.68E-05 | 0 | 0 | 0 |
| d__Bacteria;k__norank_d__Bacteria;p__Bacteroidota;c__Bacteroidia;o__Cytophagales;f__Amoebophilaceae;g__Candidatus_Amoebophilus | 0 | 0 | 2.45E-05 | 0 | 0 | 0 | 5.23E-05 | 0 | 0 | 0 | 0 | 0 |
| d__Bacteria;k__norank_d__Bacteria;p__Actinobacteriota;c__Actinobacteria;o__Micrococcales;f__Cellulomonadaceae;g__unclassified_f__Cellulomonadaceae | 0 | 0 | 0 | 0 | 0 | 0 | 0 | 0 | 0 | 0 | 2.48E-05 | 5.15E-05 |
| d__Bacteria;k__norank_d__Bacteria;p__Firmicutes;c__Clostridia;o__norank_c__Clostridia;f__Hungateiclostridiaceae;g__UCG-012 | 0 | 0 | 2.45E-05 | 0 | 0 | 0 | 0 | 2.59E-05 | 0 | 0 | 0 | 2.57E-05 |
| d__Bacteria;k__norank_d__Bacteria;p__Actinobacteriota;c__Actinobacteria;o__0319-7L14;f__norank_o__0319-7L14;g__norank_f__norank_o__0319-7L14 | 0 | 0 | 0 | 0 | 0 | 0 | 0 | 5.19E-05 | 0 | 2.40E-05 | 0 | 0 |
| d__Bacteria;k__norank_d__Bacteria;p__Firmicutes;c__Desulfotomaculia;o__Desulfotomaculales;f__norank_o__Desulfotomaculales;g__Desulfohalotomaculum | 0 | 0 | 0 | 0 | 0 | 0 | 0 | 2.59E-05 | 0 | 0 | 4.95E-05 | 0 |
| d__Bacteria;k__norank_d__Bacteria;p__Proteobacteria;c__Gammaproteobacteria;o__Burkholderiales;f__Comamonadaceae;g__Simplicispira | 0 | 0 | 0 | 0 | 0 | 0 | 0 | 2.59E-05 | 0 | 0 | 4.95E-05 | 0 |
| d__Bacteria;k__norank_d__Bacteria;p__Firmicutes;c__Desulfotomaculia;o__Desulfotomaculales;f__norank_o__Desulfotomaculales;g__Desulfotomaculum | 0 | 0 | 0 | 0 | 0 | 0 | 0 | 0 | 0 | 2.40E-05 | 0 | 5.15E-05 |
| d__Bacteria;k__norank_d__Bacteria;p__Bacteroidota;c__Bacteroidia;o__Sphingobacteriales;f__Lentimicrobiaceae;g__norank_f__Lentimicrobiaceae | 2.62E-05 | 0 | 4.90E-05 | 0 | 0 | 0 | 0 | 0 | 0 | 0 | 0 | 0 |
| d__Bacteria;k__norank_d__Bacteria;p__Firmicutes;c__Clostridia;o__Lachnospirales;f__unclassified_o__Lachnospirales;g__unclassified_o__Lachnospirales | 0 | 0 | 4.90E-05 | 0 | 0 | 0 | 0 | 2.59E-05 | 0 | 0 | 0 | 0 |
| d__Bacteria;k__norank_d__Bacteria;p__Proteobacteria;c__Alphaproteobacteria;o__Paracaedibacterales;f__Paracaedibacteraceae;g__Candidatus_Captivus | 0 | 0 | 4.90E-05 | 0 | 0 | 0 | 0 | 2.59E-05 | 0 | 0 | 0 | 0 |
| d__Bacteria;k__norank_d__Bacteria;p__Verrucomicrobiota;c__Verrucomicrobiae;o__Verrucomicrobiales;f__DEV007;g__norank_f__DEV007 | 0 | 0 | 4.90E-05 | 0 | 0 | 0 | 0 | 0 | 0 | 0 | 0 | 2.57E-05 |
| d__Bacteria;k__norank_d__Bacteria;p__Chloroflexi;c__Dehalococcoidia;o__Dehalococcoidales;f__Dehalococcoidaceae;g__Dehalogenimonas | 0 | 2.57E-05 | 4.90E-05 | 0 | 0 | 0 | 0 | 0 | 0 | 0 | 0 | 0 |
| d__Bacteria;k__norank_d__Bacteria;p__Desulfobacterota;c__Desulfuromonadia;o__unclassified_c__Desulfuromonadia;f__unclassified_c__Desulfuromonadia;g__unclassified_c__Desulfuromonadia | 0 | 2.57E-05 | 4.90E-05 | 0 | 0 | 0 | 0 | 0 | 0 | 0 | 0 | 0 |
| d__Bacteria;k__norank_d__Bacteria;p__Verrucomicrobiota;c__Verrucomicrobiae;o__Opitutales;f__Opitutaceae;g__norank_f__Opitutaceae | 0 | 2.57E-05 | 4.90E-05 | 0 | 0 | 0 | 0 | 0 | 0 | 0 | 0 | 0 |
| d__Bacteria;k__norank_d__Bacteria;p__Actinobacteriota;c__Actinobacteria;o__Nitriliruptorales;f__Nitriliruptoraceae;g__Egicoccus | 0 | 0 | 0 | 0 | 0 | 0 | 0 | 0 | 0 | 0 | 7.43E-05 | 0 |
| d__Bacteria;k__norank_d__Bacteria;p__Myxococcota;c__Polyangia;o__Polyangiales;f__Sandaracinaceae;g__unclassified_f__Sandaracinaceae | 0 | 0 | 0 | 0 | 0 | 0 | 0 | 0 | 0 | 0 | 7.43E-05 | 0 |
| d__Bacteria;k__norank_d__Bacteria;p__Proteobacteria;c__Alphaproteobacteria;o__Micavibrionales;f__Micavibrionaceae;g__unclassified_f__Micavibrionaceae | 0 | 0 | 0 | 0 | 0 | 0 | 0 | 0 | 0 | 0 | 7.43E-05 | 0 |
| d__Bacteria;k__norank_d__Bacteria;p__Verrucomicrobiota;c__Verrucomicrobiae;o__Opitutales;f__Opitutaceae;g__unclassified_f__Opitutaceae | 0 | 0 | 0 | 0 | 0 | 0 | 0 | 0 | 0 | 0 | 7.43E-05 | 0 |
| d__Bacteria;k__norank_d__Bacteria;p__Bacteroidota;c__Bacteroidia;o__Cytophagales;f__Hymenobacteraceae;g__Hymenobacter | 0 | 0 | 0 | 0 | 2.26E-05 | 0 | 0 | 2.59E-05 | 0 | 0 | 0 | 2.57E-05 |
| d__Bacteria;k__norank_d__Bacteria;p__Firmicutes;c__Bacilli;o__Caldalkalibacillales;f__Caldalkalibacillaceae;g__Caldalkalibacillus | 0 | 0 | 0 | 0 | 2.26E-05 | 0 | 0 | 2.59E-05 | 0 | 0 | 0 | 2.57E-05 |
| d__Bacteria;k__norank_d__Bacteria;p__Proteobacteria;c__Alphaproteobacteria;o__Sphingomonadales;f__Sphingomonadaceae;g__norank_f__Sphingomonadaceae | 0 | 0 | 0 | 0 | 0 | 2.21E-05 | 0 | 5.19E-05 | 0 | 0 | 0 | 0 |
| d__Bacteria;k__norank_d__Bacteria;p__Chloroflexi;c__Anaerolineae;o__ADurb.Bin180;f__norank_o__ADurb.Bin180;g__norank_f__norank_o__ADurb.Bin180 | 5.25E-05 | 0 | 0 | 2.14E-05 | 0 | 0 | 0 | 0 | 0 | 0 | 0 | 0 |
| d__Bacteria;k__norank_d__Bacteria;p__Actinobacteriota;c__Actinobacteria;o__Streptosporangiales;f__Thermomonosporaceae;g__norank_f__Thermomonosporaceae | 0 | 0 | 0 | 0 | 0 | 0 | 0 | 0 | 0 | 4.79E-05 | 0 | 2.57E-05 |
| d__Bacteria;k__norank_d__Bacteria;p__Bacteroidota;c__Bacteroidia;o__Cytophagales;f__Cytophagaceae;g__Siphonobacter | 0 | 0 | 7.36E-05 | 0 | 0 | 0 | 0 | 0 | 0 | 0 | 0 | 0 |
| d__Bacteria;k__norank_d__Bacteria;p__Firmicutes;c__Negativicutes;o__Veillonellales-Selenomonadales;f__Sporomusaceae;g__Dendrosporobacter | 0 | 0 | 7.36E-05 | 0 | 0 | 0 | 0 | 0 | 0 | 0 | 0 | 0 |
| d__Bacteria;k__norank_d__Bacteria;p__Patescibacteria;c__Parcubacteria;o__Candidatus_Giovannonibacteria;f__norank_o__Candidatus_Giovannonibacteria;g__norank_f__norank_o__Candidatus_Giovannonibacteria | 0 | 0 | 7.36E-05 | 0 | 0 | 0 | 0 | 0 | 0 | 0 | 0 | 0 |
| d__Bacteria;k__norank_d__Bacteria;p__Planctomycetota;c__Planctomycetes;o__Pirellulales;f__Pirellulaceae;g__unclassified_f__Pirellulaceae | 0 | 0 | 7.36E-05 | 0 | 0 | 0 | 0 | 0 | 0 | 0 | 0 | 0 |
| d__Bacteria;k__norank_d__Bacteria;p__Desulfobacterota;c__Syntrophobacteria;o__Syntrophobacterales;f__Syntrophobacteraceae;g__norank_f__Syntrophobacteraceae | 2.62E-05 | 2.57E-05 | 0 | 2.14E-05 | 0 | 0 | 0 | 0 | 0 | 0 | 0 | 0 |
| d__Bacteria;k__norank_d__Bacteria;p__Myxococcota;c__Myxococcia;o__Myxococcales;f__unclassified_o__Myxococcales;g__unclassified_o__Myxococcales | 2.62E-05 | 2.57E-05 | 0 | 2.14E-05 | 0 | 0 | 0 | 0 | 0 | 0 | 0 | 0 |
| d__Bacteria;k__norank_d__Bacteria;p__Chloroflexi;c__Anaerolineae;o__Anaerolineales;f__Anaerolineaceae;g__Levilinea | 0 | 0 | 4.90E-05 | 0 | 0 | 0 | 0 | 0 | 0 | 2.40E-05 | 0 | 0 |
| d__Bacteria;k__norank_d__Bacteria;p__Verrucomicrobiota;c__Verrucomicrobiae;o__Opitutales;f__unclassified_o__Opitutales;g__unclassified_o__Opitutales | 0 | 0 | 4.90E-05 | 0 | 0 | 0 | 0 | 0 | 0 | 2.40E-05 | 0 | 0 |
| d__Bacteria;k__norank_d__Bacteria;p__Proteobacteria;c__Alphaproteobacteria;o__AT-s3-44;f__norank_o__AT-s3-44;g__norank_f__norank_o__AT-s3-44 | 0 | 2.57E-05 | 2.45E-05 | 0 | 2.26E-05 | 0 | 0 | 0 | 0 | 0 | 0 | 0 |
| d__Bacteria;k__norank_d__Bacteria;p__Proteobacteria;c__Gammaproteobacteria;o__Enterobacterales;f__Enterobacteriaceae;g__Klebsiella | 0 | 0 | 2.45E-05 | 0 | 0 | 0 | 0 | 0 | 0 | 4.79E-05 | 0 | 0 |
| d__Bacteria;k__norank_d__Bacteria;p__Firmicutes;c__Bacilli;o__Thermoactinomycetales;f__Thermoactinomycetaceae;g__Risungbinella | 0 | 0 | 4.90E-05 | 0 | 0 | 2.21E-05 | 0 | 0 | 0 | 0 | 0 | 0 |
| d__Bacteria;k__norank_d__Bacteria;p__Chloroflexi;c__Chloroflexia;o__unclassified_c__Chloroflexia;f__unclassified_c__Chloroflexia;g__unclassified_c__Chloroflexia | 0 | 2.57E-05 | 0 | 0 | 4.53E-05 | 0 | 0 | 0 | 0 | 0 | 0 | 0 |
| d__Bacteria;k__norank_d__Bacteria;p__Bacteroidota;c__Bacteroidia;o__Flavobacteriales;f__Flavobacteriaceae;g__Subsaxibacter | 0 | 0 | 0 | 0 | 4.53E-05 | 0 | 0 | 0 | 2.56E-05 | 0 | 0 | 0 |
| d__Bacteria;k__norank_d__Bacteria;p__Proteobacteria;c__Gammaproteobacteria;o__Alteromonadales;f__Alteromonadaceae;g__Rheinheimera | 0 | 0 | 0 | 0 | 2.26E-05 | 2.21E-05 | 0 | 2.59E-05 | 0 | 0 | 0 | 0 |
| d__Bacteria;k__norank_d__Bacteria;p__Actinobacteriota;c__Acidimicrobiia;o__Acidimicrobiales;f__Acidimicrobiaceae;g__norank_f__Acidimicrobiaceae | 0 | 0 | 0 | 0 | 2.26E-05 | 0 | 0 | 0 | 0 | 4.79E-05 | 0 | 0 |
| d__Bacteria;k__norank_d__Bacteria;p__Bacteroidota;c__Bacteroidia;o__Flavobacteriales;f__Flavobacteriaceae;g__Arenibacter | 2.62E-05 | 0 | 0 | 0 | 0 | 4.42E-05 | 0 | 0 | 0 | 0 | 0 | 0 |
| d__Bacteria;k__norank_d__Bacteria;p__Actinobacteriota;c__Actinobacteria;o__Micrococcales;f__Intrasporangiaceae;g__Ornithinimicrobium | 0 | 2.57E-05 | 0 | 0 | 0 | 4.42E-05 | 0 | 0 | 0 | 0 | 0 | 0 |
| d__Bacteria;k__norank_d__Bacteria;p__Verrucomicrobiota;c__Chlamydiae;o__Chlamydiales;f__Criblamydiaceae;g__norank_f__Criblamydiaceae | 0 | 0 | 0 | 0 | 0 | 4.42E-05 | 0 | 0 | 2.56E-05 | 0 | 0 | 0 |
| d__Bacteria;k__norank_d__Bacteria;p__Proteobacteria;c__Gammaproteobacteria;o__Methylococcales;f__Methylomonadaceae;g__Methylomicrobium | 0 | 0 | 2.45E-05 | 0 | 4.53E-05 | 0 | 0 | 0 | 0 | 0 | 0 | 0 |
| d__Bacteria;k__norank_d__Bacteria;p__Actinobacteriota;c__Actinobacteria;o__Micrococcales;f__Bogoriellaceae;g__Georgenia | 0 | 0 | 2.45E-05 | 0 | 0 | 4.42E-05 | 0 | 0 | 0 | 0 | 0 | 0 |
| d__Bacteria;k__norank_d__Bacteria;p__Firmicutes;c__Bacilli;o__Lactobacillales;f__Enterococcaceae;g__Enterococcus | 0 | 0 | 0 | 0 | 6.79E-05 | 0 | 0 | 0 | 0 | 0 | 0 | 0 |
| d__Bacteria;k__norank_d__Bacteria;p__Proteobacteria;c__Alphaproteobacteria;o__Rhizobiales;f__Rhizobiaceae;g__Hoeflea | 0 | 0 | 0 | 0 | 6.79E-05 | 0 | 0 | 0 | 0 | 0 | 0 | 0 |
| d__Bacteria;k__norank_d__Bacteria;p__Bacteroidota;c__Bacteroidia;o__Chitinophagales;f__Saprospiraceae;g__Portibacter | 0 | 0 | 2.45E-05 | 4.29E-05 | 0 | 0 | 0 | 0 | 0 | 0 | 0 | 0 |
| d__Bacteria;k__norank_d__Bacteria;p__Firmicutes;c__Clostridia;o__Oscillospirales;f__Ruminococcaceae;g__unclassified_f__Ruminococcaceae | 0 | 0 | 2.45E-05 | 4.29E-05 | 0 | 0 | 0 | 0 | 0 | 0 | 0 | 0 |
| d__Bacteria;k__norank_d__Bacteria;p__Cyanobacteria;c__Cyanobacteriia;o__Thermosynechococcales;f__Thermosynechococcaceae;g__Synechococcus_IR11 | 0 | 0 | 0 | 0 | 0 | 6.63E-05 | 0 | 0 | 0 | 0 | 0 | 0 |
| d__Bacteria;k__norank_d__Bacteria;p__Firmicutes;c__Clostridia;o__Peptostreptococcales-Tissierellales;f__Peptostreptococcaceae;g__Paraclostridium | 0 | 0 | 0 | 0 | 0 | 6.63E-05 | 0 | 0 | 0 | 0 | 0 | 0 |
| d__Bacteria;k__norank_d__Bacteria;p__Actinobacteriota;c__Actinobacteria;o__Kineosporiales;f__Kineosporiaceae;g__Kineococcus | 0 | 0 | 0 | 6.43E-05 | 0 | 0 | 0 | 0 | 0 | 0 | 0 | 0 |
| d__Bacteria;k__norank_d__Bacteria;p__Firmicutes;c__Clostridia;o__Peptococcales;f__Peptococcaceae;g__unclassified_f__Peptococcaceae | 0 | 0 | 0 | 6.43E-05 | 0 | 0 | 0 | 0 | 0 | 0 | 0 | 0 |
| d__Bacteria;k__norank_d__Bacteria;p__Myxococcota;c__Polyangia;o__Nannocystales;f__Nannocystaceae;g__Pseudenhygromyxa | 0 | 0 | 0 | 6.43E-05 | 0 | 0 | 0 | 0 | 0 | 0 | 0 | 0 |
| d__Bacteria;k__norank_d__Bacteria;p__Planctomycetota;c__Brocadiae;o__Brocadiales;f__Brocadiaceae;g__Candidatus_Brocadia | 0 | 0 | 0 | 6.43E-05 | 0 | 0 | 0 | 0 | 0 | 0 | 0 | 0 |
| d__Bacteria;k__norank_d__Bacteria;p__Proteobacteria;c__Alphaproteobacteria;o__Caulobacterales;f__unclassified_o__Caulobacterales;g__unclassified_o__Caulobacterales | 0 | 0 | 0 | 6.43E-05 | 0 | 0 | 0 | 0 | 0 | 0 | 0 | 0 |
| d__Bacteria;k__norank_d__Bacteria;p__Proteobacteria;c__Alphaproteobacteria;o__Rhizobiales;f__Xanthobacteraceae;g__Pseudoxanthobacter | 0 | 0 | 0 | 6.43E-05 | 0 | 0 | 0 | 0 | 0 | 0 | 0 | 0 |
| d__Bacteria;k__norank_d__Bacteria;p__Bacteroidota;c__Bacteroidia;o__Cytophagales;f__Spirosomaceae;g__Fibrella | 5.25E-05 | 0 | 0 | 0 | 0 | 0 | 0 | 0 | 0 | 0 | 0 | 0 |
| d__Bacteria;k__norank_d__Bacteria;p__Firmicutes;c__Clostridia;o__Clostridia_vadinBB60_group;f__norank_o__Clostridia_vadinBB60_group;g__norank_f__norank_o__Clostridia_vadinBB60_group | 5.25E-05 | 0 | 0 | 0 | 0 | 0 | 0 | 0 | 0 | 0 | 0 | 0 |
| d__Bacteria;k__norank_d__Bacteria;p__Firmicutes;c__Dethiobacteria;o__Dethiobacterales;f__Dethiobacteraceae;g__norank_f__Dethiobacteraceae | 5.25E-05 | 0 | 0 | 0 | 0 | 0 | 0 | 0 | 0 | 0 | 0 | 0 |
| d__Bacteria;k__norank_d__Bacteria;p__Firmicutes;c__Limnochordia;o__MBA03;f__norank_o__MBA03;g__norank_f__norank_o__MBA03 | 5.25E-05 | 0 | 0 | 0 | 0 | 0 | 0 | 0 | 0 | 0 | 0 | 0 |
| d__Bacteria;k__norank_d__Bacteria;p__Planctomycetota;c__Planctomycetes;o__Gemmatales;f__Gemmataceae;g__Gemmata | 5.25E-05 | 0 | 0 | 0 | 0 | 0 | 0 | 0 | 0 | 0 | 0 | 0 |
| d__Bacteria;k__norank_d__Bacteria;p__Proteobacteria;c__Gammaproteobacteria;o__Burkholderiales;f__Alcaligenaceae;g__Pigmentiphaga | 5.25E-05 | 0 | 0 | 0 | 0 | 0 | 0 | 0 | 0 | 0 | 0 | 0 |
| d__Bacteria;k__norank_d__Bacteria;p__Proteobacteria;c__Gammaproteobacteria;o__Burkholderiales;f__Oxalobacteraceae;g__Collimonas | 5.25E-05 | 0 | 0 | 0 | 0 | 0 | 0 | 0 | 0 | 0 | 0 | 0 |
| d__Bacteria;k__norank_d__Bacteria;p__Actinobacteriota;c__Acidimicrobiia;o__Microtrichales;f__Microtrichaceae;g__Sva0996_marine_group | 0 | 0 | 0 | 0 | 0 | 0 | 5.23E-05 | 0 | 0 | 0 | 0 | 0 |
| d__Bacteria;k__norank_d__Bacteria;p__Bacteroidota;c__Bacteroidia;o__Flavobacteriales;f__Flavobacteriaceae;g__Vitellibacter | 0 | 0 | 0 | 0 | 0 | 0 | 5.23E-05 | 0 | 0 | 0 | 0 | 0 |
| d__Bacteria;k__norank_d__Bacteria;p__Proteobacteria;c__Gammaproteobacteria;o__Steroidobacterales;f__Steroidobacteraceae;g__unclassified_f__Steroidobacteraceae | 0 | 0 | 0 | 0 | 0 | 0 | 5.23E-05 | 0 | 0 | 0 | 0 | 0 |
| d__Bacteria;k__norank_d__Bacteria;p__Cyanobacteria;c__Cyanobacteriia;o__Cyanobacteriales;f__Oscillatoriaceae;g__Oxynema_BDU_92071 | 2.62E-05 | 2.57E-05 | 0 | 0 | 0 | 0 | 0 | 0 | 0 | 0 | 0 | 0 |
| d__Bacteria;k__norank_d__Bacteria;p__Firmicutes;c__Desulfitobacteriia;o__Desulfitobacteriales;f__unclassified_o__Desulfitobacteriales;g__unclassified_o__Desulfitobacteriales | 0 | 0 | 0 | 0 | 0 | 0 | 0 | 5.19E-05 | 0 | 0 | 0 | 0 |
| d__Bacteria;k__norank_d__Bacteria;p__Fibrobacterota;c__Fibrobacteria;o__Fibrobacterales;f__Fibrobacteraceae;g__norank_f__Fibrobacteraceae | 0 | 2.57E-05 | 0 | 0 | 0 | 0 | 2.61E-05 | 0 | 0 | 0 | 0 | 0 |
| d__Bacteria;k__norank_d__Bacteria;p__Firmicutes;c__Bacilli;o__Bacillales;f__Bacillaceae;g__Sinibacillus | 0 | 2.57E-05 | 0 | 0 | 0 | 0 | 2.61E-05 | 0 | 0 | 0 | 0 | 0 |
| d__Bacteria;k__norank_d__Bacteria;p__Actinobacteriota;c__Actinobacteria;o__Propionibacteriales;f__Propionibacteriaceae;g__Haloactinopolyspora | 0 | 0 | 0 | 0 | 0 | 0 | 2.61E-05 | 0 | 2.56E-05 | 0 | 0 | 0 |
| d__Bacteria;k__norank_d__Bacteria;p__Firmicutes;c__Limnochordia;o__Limnochordales;f__norank_o__Limnochordales;g__norank_f__norank_o__Limnochordales | 0 | 0 | 0 | 0 | 0 | 0 | 0 | 0 | 0 | 0 | 0 | 5.15E-05 |
| d__Bacteria;k__norank_d__Bacteria;p__Methylomirabilota;c__Methylomirabilia;o__Methylomirabilales;f__Methylomirabilaceae;g__wb1-A12 | 0 | 0 | 0 | 0 | 0 | 0 | 0 | 0 | 0 | 0 | 0 | 5.15E-05 |
| d__Bacteria;k__norank_d__Bacteria;p__Cyanobacteria;c__Cyanobacteriia;o__Cyanobacteriales;f__Phormidiaceae;g__Kamptonema_PCC-6407 | 0 | 5.13E-05 | 0 | 0 | 0 | 0 | 0 | 0 | 0 | 0 | 0 | 0 |
| d__Bacteria;k__norank_d__Bacteria;p__Cyanobacteria;c__Cyanobacteriia;o__Leptolyngbyales;f__Leptolyngbyaceae;g__Phormidesmis_ANT.L52.6 | 0 | 5.13E-05 | 0 | 0 | 0 | 0 | 0 | 0 | 0 | 0 | 0 | 0 |
| d__Bacteria;k__norank_d__Bacteria;p__Firmicutes;c__Clostridia;o__Peptostreptococcales-Tissierellales;f__norank_o__Peptostreptococcales-Tissierellales;g__unclassified_f__norank_o__Peptostreptococcales-Tissierellales | 0 | 5.13E-05 | 0 | 0 | 0 | 0 | 0 | 0 | 0 | 0 | 0 | 0 |
| d__Bacteria;k__norank_d__Bacteria;p__Firmicutes;c__Limnochordia;o__M55-D21;f__norank_o__M55-D21;g__norank_f__norank_o__M55-D21 | 0 | 5.13E-05 | 0 | 0 | 0 | 0 | 0 | 0 | 0 | 0 | 0 | 0 |
| d__Bacteria;k__norank_d__Bacteria;p__Firmicutes;c__Symbiobacteriia;o__Symbiobacteriales;f__norank_o__Symbiobacteriales;g__norank_f__norank_o__Symbiobacteriales | 0 | 5.13E-05 | 0 | 0 | 0 | 0 | 0 | 0 | 0 | 0 | 0 | 0 |
| d__Bacteria;k__norank_d__Bacteria;p__Proteobacteria;c__Gammaproteobacteria;o__BD72BR169;f__norank_o__BD72BR169;g__norank_f__norank_o__BD72BR169 | 0 | 5.13E-05 | 0 | 0 | 0 | 0 | 0 | 0 | 0 | 0 | 0 | 0 |
| d__Bacteria;k__norank_d__Bacteria;p__Proteobacteria;c__Alphaproteobacteria;o__Rhodospirillales;f__Magnetospirillaceae;g__norank_f__Magnetospirillaceae | 0 | 0 | 0 | 0 | 0 | 0 | 0 | 0 | 5.12E-05 | 0 | 0 | 0 |
| d__Bacteria;k__norank_d__Bacteria;p__Firmicutes;c__Bacilli;o__Entomoplasmatales;f__norank_o__Entomoplasmatales;g__Candidatus_Spiroplasma | 2.62E-05 | 0 | 2.45E-05 | 0 | 0 | 0 | 0 | 0 | 0 | 0 | 0 | 0 |
| d__Bacteria;k__norank_d__Bacteria;p__Firmicutes;c__Bacilli;o__Erysipelotrichales;f__unclassified_o__Erysipelotrichales;g__unclassified_o__Erysipelotrichales | 2.62E-05 | 0 | 2.45E-05 | 0 | 0 | 0 | 0 | 0 | 0 | 0 | 0 | 0 |
| d__Bacteria;k__norank_d__Bacteria;p__Firmicutes;c__Clostridia;o__Lachnospirales;f__Defluviitaleaceae;g__Defluviitaleaceae_UCG-011 | 2.62E-05 | 0 | 2.45E-05 | 0 | 0 | 0 | 0 | 0 | 0 | 0 | 0 | 0 |
| d__Bacteria;k__norank_d__Bacteria;p__Proteobacteria;c__Gammaproteobacteria;o__Burkholderiales;f__Rhodocyclaceae;g__Ferribacterium | 2.62E-05 | 0 | 2.45E-05 | 0 | 0 | 0 | 0 | 0 | 0 | 0 | 0 | 0 |
| d__Bacteria;k__norank_d__Bacteria;p__Proteobacteria;c__Alphaproteobacteria;o__Rhizobiales;f__Stappiaceae;g__Agaricicola | 0 | 0 | 2.45E-05 | 0 | 0 | 0 | 2.61E-05 | 0 | 0 | 0 | 0 | 0 |
| d__Bacteria;k__norank_d__Bacteria;p__Verrucomicrobiota;c__Verrucomicrobiae;o__Pedosphaerales;f__Pedosphaeraceae;g__DEV008 | 0 | 2.57E-05 | 0 | 0 | 0 | 0 | 0 | 0 | 0 | 0 | 2.48E-05 | 0 |
| d__Bacteria;k__norank_d__Bacteria;p__Proteobacteria;c__Gammaproteobacteria;o__Burkholderiales;f__Rhodocyclaceae;g__Georgfuchsia | 0 | 0 | 0 | 0 | 0 | 0 | 0 | 0 | 2.56E-05 | 0 | 2.48E-05 | 0 |
| d__Bacteria;k__norank_d__Bacteria;p__Firmicutes;c__Clostridia;o__Lachnospirales;f__norank_o__Lachnospirales;g__norank_f__norank_o__Lachnospirales | 0 | 2.57E-05 | 2.45E-05 | 0 | 0 | 0 | 0 | 0 | 0 | 0 | 0 | 0 |
| d__Bacteria;k__norank_d__Bacteria;p__Planctomycetota;c__Phycisphaerae;o__Tepidisphaerales;f__CPla-3_termite_group;g__norank_f__CPla-3_termite_group | 0 | 2.57E-05 | 2.45E-05 | 0 | 0 | 0 | 0 | 0 | 0 | 0 | 0 | 0 |
| d__Bacteria;k__norank_d__Bacteria;p__Cyanobacteria;c__Cyanobacteriia;o__Oxyphotobacteria_Incertae_Sedis;f__unclassified_o__Oxyphotobacteria_Incertae_Sedis;g__Phormidium_CYN64 | 0 | 0 | 0 | 0 | 0 | 0 | 0 | 0 | 0 | 2.40E-05 | 0 | 2.57E-05 |
| d__Bacteria;k__norank_d__Bacteria;p__Actinobacteriota;c__Actinobacteria;o__Pseudonocardiales;f__Pseudonocardiaceae;g__norank_f__Pseudonocardiaceae | 0 | 0 | 0 | 0 | 0 | 0 | 0 | 0 | 0 | 0 | 4.95E-05 | 0 |
| d__Bacteria;k__norank_d__Bacteria;p__Proteobacteria;c__Gammaproteobacteria;o__Burkholderiales;f__Methylophilaceae;g__Methylobacillus | 0 | 0 | 0 | 0 | 0 | 0 | 0 | 0 | 0 | 0 | 4.95E-05 | 0 |
| d__Bacteria;k__norank_d__Bacteria;p__Proteobacteria;c__Alphaproteobacteria;o__Rhizobiales;f__Rhizobiales_Incertae_Sedis;g__Alsobacter | 0 | 0 | 2.45E-05 | 0 | 0 | 0 | 0 | 0 | 0 | 0 | 2.48E-05 | 0 |
| d__Bacteria;k__norank_d__Bacteria;p__Desulfobacterota;c__Syntrophobacteria;o__Syntrophobacterales;f__Syntrophobacteraceae;g__Desulfovirga | 0 | 0 | 4.90E-05 | 0 | 0 | 0 | 0 | 0 | 0 | 0 | 0 | 0 |
| d__Bacteria;k__norank_d__Bacteria;p__Firmicutes;c__Desulfotomaculia;o__Desulfotomaculales;f__norank_o__Desulfotomaculales;g__Desulfofarcimen | 0 | 0 | 4.90E-05 | 0 | 0 | 0 | 0 | 0 | 0 | 0 | 0 | 0 |
| d__Bacteria;k__norank_d__Bacteria;p__Proteobacteria;c__Gammaproteobacteria;o__Burkholderiales;f__Rhodocyclaceae;g__Denitratisoma | 0 | 0 | 4.90E-05 | 0 | 0 | 0 | 0 | 0 | 0 | 0 | 0 | 0 |
| d__Bacteria;k__norank_d__Bacteria;p__Proteobacteria;c__Gammaproteobacteria;o__Methylococcales;f__Methylomonadaceae;g__Methyloglobulus | 0 | 0 | 4.90E-05 | 0 | 0 | 0 | 0 | 0 | 0 | 0 | 0 | 0 |
| d__Bacteria;k__norank_d__Bacteria;p__Spirochaetota;c__Leptospirae;o__Leptospirales;f__Leptospiraceae;g__Leptospira | 0 | 0 | 4.90E-05 | 0 | 0 | 0 | 0 | 0 | 0 | 0 | 0 | 0 |
| d__Bacteria;k__norank_d__Bacteria;p__Firmicutes;c__Clostridia;o__Clostridia_UCG-014;f__norank_o__Clostridia_UCG-014;g__norank_f__norank_o__Clostridia_UCG-014 | 2.62E-05 | 0 | 0 | 0 | 2.26E-05 | 0 | 0 | 0 | 0 | 0 | 0 | 0 |
| d__Bacteria;k__norank_d__Bacteria;p__Proteobacteria;c__Gammaproteobacteria;o__Burkholderiales;f__Burkholderiaceae;g__norank_f__Burkholderiaceae | 2.62E-05 | 0 | 0 | 0 | 2.26E-05 | 0 | 0 | 0 | 0 | 0 | 0 | 0 |
| d__Bacteria;k__norank_d__Bacteria;p__Entotheonellaeota;c__Entotheonellia;o__Entotheonellales;f__Entotheonellaceae;g__Candidatus_Entotheonella | 0 | 0 | 0 | 0 | 2.26E-05 | 0 | 2.61E-05 | 0 | 0 | 0 | 0 | 0 |
| d__Bacteria;k__norank_d__Bacteria;p__Proteobacteria;c__Gammaproteobacteria;o__Cellvibrionales;f__Cellvibrionaceae;g__norank_f__Cellvibrionaceae | 0 | 0 | 0 | 0 | 2.26E-05 | 0 | 2.61E-05 | 0 | 0 | 0 | 0 | 0 |
| d__Bacteria;k__norank_d__Bacteria;p__Proteobacteria;c__Alphaproteobacteria;o__Rickettsiales;f__bac2nit3;g__norank_f__bac2nit3 | 0 | 0 | 0 | 0 | 2.26E-05 | 0 | 0 | 2.59E-05 | 0 | 0 | 0 | 0 |
| d__Bacteria;k__norank_d__Bacteria;p__Verrucomicrobiota;c__Omnitrophia;o__Omnitrophales;f__unclassified_o__Omnitrophales;g__unclassified_o__Omnitrophales | 0 | 0 | 0 | 0 | 0 | 0 | 0 | 0 | 0 | 4.79E-05 | 0 | 0 |
| d__Bacteria;k__norank_d__Bacteria;p__Proteobacteria;c__Alphaproteobacteria;o__Parvibaculales;f__Parvibaculaceae;g__Rhodoligotrophos | 0 | 0 | 0 | 0 | 0 | 2.21E-05 | 0 | 0 | 2.56E-05 | 0 | 0 | 0 |
| d__Bacteria;k__norank_d__Bacteria;p__Firmicutes;c__Clostridia;o__Clostridiales;f__Caloramatoraceae;g__Fervidicella | 0 | 0 | 2.45E-05 | 0 | 2.26E-05 | 0 | 0 | 0 | 0 | 0 | 0 | 0 |
| d__Bacteria;k__norank_d__Bacteria;p__Verrucomicrobiota;c__Verrucomicrobiae;o__UA11;f__norank_o__UA11;g__norank_f__norank_o__UA11 | 0 | 0 | 2.45E-05 | 0 | 2.26E-05 | 0 | 0 | 0 | 0 | 0 | 0 | 0 |
| d__Bacteria;k__norank_d__Bacteria;p__Proteobacteria;c__Alphaproteobacteria;o__Sphingomonadales;f__Sphingomonadaceae;g__Sandaracinobacter | 0 | 0 | 0 | 0 | 4.53E-05 | 0 | 0 | 0 | 0 | 0 | 0 | 0 |
| d__Bacteria;k__norank_d__Bacteria;p__Verrucomicrobiota;c__Verrucomicrobiae;o__Chthoniobacterales;f__Terrimicrobiaceae;g__FukuN18_freshwater_group | 0 | 0 | 0 | 0 | 4.53E-05 | 0 | 0 | 0 | 0 | 0 | 0 | 0 |
| d__Bacteria;k__norank_d__Bacteria;p__Actinobacteriota;c__Actinobacteria;o__Micrococcales;f__Brevibacteriaceae;g__Brevibacterium | 0 | 0 | 0 | 0 | 0 | 4.42E-05 | 0 | 0 | 0 | 0 | 0 | 0 |
| d__Bacteria;k__norank_d__Bacteria;p__Bacteroidota;c__Bacteroidia;o__Chitinophagales;f__Saprospiraceae;g__unclassified_f__Saprospiraceae | 0 | 0 | 0 | 0 | 0 | 4.42E-05 | 0 | 0 | 0 | 0 | 0 | 0 |
| d__Bacteria;k__norank_d__Bacteria;p__Proteobacteria;c__Gammaproteobacteria;o__Legionellales;f__Legionellaceae;g__norank_f__Legionellaceae | 0 | 0 | 0 | 0 | 0 | 4.42E-05 | 0 | 0 | 0 | 0 | 0 | 0 |
| d__Bacteria;k__norank_d__Bacteria;p__Chloroflexi;c__Ktedonobacteria;o__Ktedonobacterales;f__Ktedonobacteraceae;g__Ktedonobacter | 0 | 0 | 0 | 2.14E-05 | 2.26E-05 | 0 | 0 | 0 | 0 | 0 | 0 | 0 |
| d__Bacteria;k__norank_d__Bacteria;p__Proteobacteria;c__Alphaproteobacteria;o__Rhizobiales;f__Pleomorphomonadaceae;g__Prosthecomicrobium | 0 | 0 | 0 | 2.14E-05 | 2.26E-05 | 0 | 0 | 0 | 0 | 0 | 0 | 0 |
| d__Bacteria;k__norank_d__Bacteria;p__Actinobacteriota;c__Actinobacteria;o__Propionibacteriales;f__Propionibacteriaceae;g__Propionicicella | 0 | 0 | 0 | 2.14E-05 | 0 | 2.21E-05 | 0 | 0 | 0 | 0 | 0 | 0 |
| d__Bacteria;k__norank_d__Bacteria;p__Cyanobacteria;c__Cyanobacteriia;o__Cyanobacteriales;f__Chroococcidiopsaceae;g__Aliterella | 0 | 0 | 0 | 2.14E-05 | 0 | 2.21E-05 | 0 | 0 | 0 | 0 | 0 | 0 |
| d__Bacteria;k__norank_d__Bacteria;p__Cyanobacteria;c__Cyanobacteriia;o__Cyanobacteriales;f__unclassified_o__Cyanobacteriales;g__unclassified_o__Cyanobacteriales | 0 | 0 | 0 | 4.29E-05 | 0 | 0 | 0 | 0 | 0 | 0 | 0 | 0 |
| d__Bacteria;k__norank_d__Bacteria;p__Actinobacteriota;c__Actinobacteria;o__Frankiales;f__Sporichthyaceae;g__hgcI_clade | 2.62E-05 | 0 | 0 | 0 | 0 | 0 | 0 | 0 | 0 | 0 | 0 | 0 |
| d__Bacteria;k__norank_d__Bacteria;p__Cyanobacteria;c__Cyanobacteriia;o__Cyanobacteriales;f__Cyanobacteriales_Incertae_Sedis;g__norank_f__Cyanobacteriales_Incertae_Sedis | 2.62E-05 | 0 | 0 | 0 | 0 | 0 | 0 | 0 | 0 | 0 | 0 | 0 |
| d__Bacteria;k__norank_d__Bacteria;p__Cyanobacteria;c__Cyanobacteriia;o__Cyanobacteriales;f__Nostocaceae;g__Fortiea_PCC-7126 | 2.62E-05 | 0 | 0 | 0 | 0 | 0 | 0 | 0 | 0 | 0 | 0 | 0 |
| d__Bacteria;k__norank_d__Bacteria;p__Firmicutes;c__Bacilli;o__Lactobacillales;f__Carnobacteriaceae;g__Trichococcus | 2.62E-05 | 0 | 0 | 0 | 0 | 0 | 0 | 0 | 0 | 0 | 0 | 0 |
| d__Bacteria;k__norank_d__Bacteria;p__Firmicutes;c__Clostridia;o__Lachnospirales;f__Lachnospiraceae;g__Lachnospiraceae_NK4A136_group | 2.62E-05 | 0 | 0 | 0 | 0 | 0 | 0 | 0 | 0 | 0 | 0 | 0 |
| d__Bacteria;k__norank_d__Bacteria;p__Firmicutes;c__Clostridia;o__Oscillospirales;f__Ruminococcaceae;g__Anaerotruncus | 2.62E-05 | 0 | 0 | 0 | 0 | 0 | 0 | 0 | 0 | 0 | 0 | 0 |
| d__Bacteria;k__norank_d__Bacteria;p__Firmicutes;c__Clostridia;o__Oscillospirales;f__Ruminococcaceae;g__Candidatus_Soleaferrea | 2.62E-05 | 0 | 0 | 0 | 0 | 0 | 0 | 0 | 0 | 0 | 0 | 0 |
| d__Bacteria;k__norank_d__Bacteria;p__Firmicutes;c__Syntrophomonadia;o__Syntrophomonadales;f__Syntrophomonadaceae;g__norank_f__Syntrophomonadaceae | 2.62E-05 | 0 | 0 | 0 | 0 | 0 | 0 | 0 | 0 | 0 | 0 | 0 |
| d__Bacteria;k__norank_d__Bacteria;p__Patescibacteria;c__CPR2;o__norank_c__CPR2;f__norank_o__norank_c__CPR2;g__norank_f__norank_o__norank_c__CPR2 | 2.62E-05 | 0 | 0 | 0 | 0 | 0 | 0 | 0 | 0 | 0 | 0 | 0 |
| d__Bacteria;k__norank_d__Bacteria;p__Patescibacteria;c__Parcubacteria;o__Candidatus_Doudnabacteria;f__norank_o__Candidatus_Doudnabacteria;g__norank_f__norank_o__Candidatus_Doudnabacteria | 2.62E-05 | 0 | 0 | 0 | 0 | 0 | 0 | 0 | 0 | 0 | 0 | 0 |
| d__Bacteria;k__norank_d__Bacteria;p__Planctomycetota;c__Phycisphaerae;o__Pla1_lineage;f__norank_o__Pla1_lineage;g__norank_f__norank_o__Pla1_lineage | 2.62E-05 | 0 | 0 | 0 | 0 | 0 | 0 | 0 | 0 | 0 | 0 | 0 |
| d__Bacteria;k__norank_d__Bacteria;p__Proteobacteria;c__Gammaproteobacteria;o__Burkholderiales;f__Comamonadaceae;g__Curvibacter | 2.62E-05 | 0 | 0 | 0 | 0 | 0 | 0 | 0 | 0 | 0 | 0 | 0 |
| d__Bacteria;k__norank_d__Bacteria;p__Proteobacteria;c__Gammaproteobacteria;o__Enterobacterales;f__Yersiniaceae;g__Rahnella1 | 2.62E-05 | 0 | 0 | 0 | 0 | 0 | 0 | 0 | 0 | 0 | 0 | 0 |
| d__Bacteria;k__norank_d__Bacteria;p__Verrucomicrobiota;c__Verrucomicrobiae;o__Opitutales;f__Opitutaceae;g__IMCC26134 | 2.62E-05 | 0 | 0 | 0 | 0 | 0 | 0 | 0 | 0 | 0 | 0 | 0 |
| d__Bacteria;k__norank_d__Bacteria;p__Bacteroidota;c__Bacteroidia;o__Cytophagales;f__Spirosomaceae;g__Larkinella | 0 | 0 | 0 | 0 | 0 | 0 | 2.61E-05 | 0 | 0 | 0 | 0 | 0 |
| d__Bacteria;k__norank_d__Bacteria;p__Bacteroidota;c__Bacteroidia;o__Sphingobacteriales;f__Sphingobacteriaceae;g__Parapedobacter | 0 | 0 | 0 | 0 | 0 | 0 | 2.61E-05 | 0 | 0 | 0 | 0 | 0 |
| d__Bacteria;k__norank_d__Bacteria;p__Firmicutes;c__Bacilli;o__Lactobacillales;f__Listeriaceae;g__Listeria | 0 | 0 | 0 | 0 | 0 | 0 | 2.61E-05 | 0 | 0 | 0 | 0 | 0 |
| d__Bacteria;k__norank_d__Bacteria;p__Proteobacteria;c__Alphaproteobacteria;o__Puniceispirillales;f__norank_o__Puniceispirillales;g__norank_f__norank_o__Puniceispirillales | 0 | 0 | 0 | 0 | 0 | 0 | 2.61E-05 | 0 | 0 | 0 | 0 | 0 |
| d__Bacteria;k__norank_d__Bacteria;p__Proteobacteria;c__Gammaproteobacteria;o__Burkholderiales;f__Chitinibacteraceae;g__Chitinibacter | 0 | 0 | 0 | 0 | 0 | 0 | 2.61E-05 | 0 | 0 | 0 | 0 | 0 |
| d__Bacteria;k__norank_d__Bacteria;p__Actinobacteriota;c__Actinobacteria;o__Frankiales;f__Sporichthyaceae;g__unclassified_f__Sporichthyaceae | 0 | 0 | 0 | 0 | 0 | 0 | 0 | 2.59E-05 | 0 | 0 | 0 | 0 |
| d__Bacteria;k__norank_d__Bacteria;p__Bacteroidota;c__Bacteroidia;o__Bacteroidales;f__unclassified_o__Bacteroidales;g__unclassified_o__Bacteroidales | 0 | 0 | 0 | 0 | 0 | 0 | 0 | 2.59E-05 | 0 | 0 | 0 | 0 |
| d__Bacteria;k__norank_d__Bacteria;p__Firmicutes;c__Clostridia;o__Clostridiales;f__Clostridiaceae;g__norank_f__Clostridiaceae | 0 | 0 | 0 | 0 | 0 | 0 | 0 | 2.59E-05 | 0 | 0 | 0 | 0 |
| d__Bacteria;k__norank_d__Bacteria;p__Firmicutes;c__Clostridia;o__Peptostreptococcales-Tissierellales;f__Peptostreptococcaceae;g__norank_f__Peptostreptococcaceae | 0 | 0 | 0 | 0 | 0 | 0 | 0 | 2.59E-05 | 0 | 0 | 0 | 0 |
| d__Bacteria;k__norank_d__Bacteria;p__Proteobacteria;c__Gammaproteobacteria;o__Burkholderiales;f__Rhodocyclaceae;g__Thauera | 0 | 0 | 0 | 0 | 0 | 0 | 0 | 2.59E-05 | 0 | 0 | 0 | 0 |
| d__Bacteria;k__norank_d__Bacteria;p__Actinobacteriota;c__Actinobacteria;o__Micrococcales;f__Dermacoccaceae;g__Yimella | 0 | 0 | 0 | 0 | 0 | 0 | 0 | 0 | 0 | 0 | 0 | 2.57E-05 |
| d__Bacteria;k__norank_d__Bacteria;p__Bacteroidota;c__Bacteroidia;o__Cytophagales;f__Cyclobacteriaceae;g__Imperialibacter | 0 | 0 | 0 | 0 | 0 | 0 | 0 | 0 | 0 | 0 | 0 | 2.57E-05 |
| d__Bacteria;k__norank_d__Bacteria;p__Cyanobacteria;c__Cyanobacteriia;o__Oxyphotobacteria_Incertae_Sedis;f__unclassified_o__Oxyphotobacteria_Incertae_Sedis;g__unclassified_o__Oxyphotobacteria_Incertae_Sedis | 0 | 0 | 0 | 0 | 0 | 0 | 0 | 0 | 0 | 0 | 0 | 2.57E-05 |
| d__Bacteria;k__norank_d__Bacteria;p__Dependentiae;c__Babeliae;o__Babeliales;f__UBA12411;g__norank_f__UBA12411 | 0 | 0 | 0 | 0 | 0 | 0 | 0 | 0 | 0 | 0 | 0 | 2.57E-05 |
| d__Bacteria;k__norank_d__Bacteria;p__Firmicutes;c__Bacilli;o__Thermoactinomycetales;f__Thermoactinomycetaceae;g__Novibacillus | 0 | 0 | 0 | 0 | 0 | 0 | 0 | 0 | 0 | 0 | 0 | 2.57E-05 |
| d__Bacteria;k__norank_d__Bacteria;p__Firmicutes;c__Clostridia;o__Clostridiales;f__Caloramatoraceae;g__norank_f__Caloramatoraceae | 0 | 0 | 0 | 0 | 0 | 0 | 0 | 0 | 0 | 0 | 0 | 2.57E-05 |
| d__Bacteria;k__norank_d__Bacteria;p__Firmicutes;c__Syntrophomonadia;o__Syntrophomonadales;f__Syntrophomonadaceae;g__unclassified_f__Syntrophomonadaceae | 0 | 0 | 0 | 0 | 0 | 0 | 0 | 0 | 0 | 0 | 0 | 2.57E-05 |
| d__Bacteria;k__norank_d__Bacteria;p__Proteobacteria;c__Gammaproteobacteria;o__Burkholderiales;f__Procabacteriaceae;g__Procabacter | 0 | 0 | 0 | 0 | 0 | 0 | 0 | 0 | 0 | 0 | 0 | 2.57E-05 |
| d__Bacteria;k__norank_d__Bacteria;p__Proteobacteria;c__Gammaproteobacteria;o__Oceanospirillales;f__Halomonadaceae;g__HdN1 | 0 | 0 | 0 | 0 | 0 | 0 | 0 | 0 | 0 | 0 | 0 | 2.57E-05 |
| d__Bacteria;k__norank_d__Bacteria;p__Actinobacteriota;c__Actinobacteria;o__Streptosporangiales;f__Thermomonosporaceae;g__Actinoallomurus | 0 | 2.57E-05 | 0 | 0 | 0 | 0 | 0 | 0 | 0 | 0 | 0 | 0 |
| d__Bacteria;k__norank_d__Bacteria;p__Bacteroidota;c__Bacteroidia;o__Flavobacteriales;f__Crocinitomicaceae;g__unclassified_f__Crocinitomicaceae | 0 | 2.57E-05 | 0 | 0 | 0 | 0 | 0 | 0 | 0 | 0 | 0 | 0 |
| d__Bacteria;k__norank_d__Bacteria;p__Bacteroidota;c__Ignavibacteria;o__Ignavibacteriales;f__unclassified_o__Ignavibacteriales;g__unclassified_o__Ignavibacteriales | 0 | 2.57E-05 | 0 | 0 | 0 | 0 | 0 | 0 | 0 | 0 | 0 | 0 |
| d__Bacteria;k__norank_d__Bacteria;p__Elusimicrobiota;c__Endomicrobia;o__Endomicrobiales;f__Endomicrobiaceae;g__Endomicrobium | 0 | 2.57E-05 | 0 | 0 | 0 | 0 | 0 | 0 | 0 | 0 | 0 | 0 |
| d__Bacteria;k__norank_d__Bacteria;p__Firmicutes;c__Clostridia;o__Clostridiales;f__Clostridiaceae;g__Proteiniclasticum | 0 | 2.57E-05 | 0 | 0 | 0 | 0 | 0 | 0 | 0 | 0 | 0 | 0 |
| d__Bacteria;k__norank_d__Bacteria;p__Firmicutes;c__Symbiobacteriia;o__Symbiobacteriales;f__Symbiobacteraceae;g__unclassified_f__Symbiobacteraceae | 0 | 2.57E-05 | 0 | 0 | 0 | 0 | 0 | 0 | 0 | 0 | 0 | 0 |
| d__Bacteria;k__norank_d__Bacteria;p__Proteobacteria;c__Gammaproteobacteria;o__Burkholderiales;f__Rhodocyclaceae;g__Sulfuritalea | 0 | 2.57E-05 | 0 | 0 | 0 | 0 | 0 | 0 | 0 | 0 | 0 | 0 |
| d__Bacteria;k__norank_d__Bacteria;p__Spirochaetota;c__Spirochaetia;o__Spirochaetales;f__Spirochaetaceae;g__Salinispira | 0 | 2.57E-05 | 0 | 0 | 0 | 0 | 0 | 0 | 0 | 0 | 0 | 0 |
| d__Bacteria;k__norank_d__Bacteria;p__Bacteroidota;c__Bacteroidia;o__Flavobacteriales;f__Flavobacteriaceae;g__Antarcticibacterium | 0 | 0 | 0 | 0 | 0 | 0 | 0 | 0 | 2.56E-05 | 0 | 0 | 0 |
| d__Bacteria;k__norank_d__Bacteria;p__Proteobacteria;c__Alphaproteobacteria;o__Rhizobiales;f__Xanthobacteraceae;g__Xanthobacter | 0 | 0 | 0 | 0 | 0 | 0 | 0 | 0 | 2.56E-05 | 0 | 0 | 0 |
| d__Bacteria;k__norank_d__Bacteria;p__Proteobacteria;c__Alphaproteobacteria;o__Sphingomonadales;f__Sphingomonadaceae;g__Croceicoccus | 0 | 0 | 0 | 0 | 0 | 0 | 0 | 0 | 2.56E-05 | 0 | 0 | 0 |
| d__Bacteria;k__norank_d__Bacteria;p__Proteobacteria;c__Gammaproteobacteria;o__Pseudomonadales;f__Moraxellaceae;g__Alkanindiges | 0 | 0 | 0 | 0 | 0 | 0 | 0 | 0 | 2.56E-05 | 0 | 0 | 0 |
| d__Bacteria;k__norank_d__Bacteria;p__Bacteroidota;c__Bacteroidia;o__Cytophagales;f__Spirosomaceae;g__norank_f__Spirosomaceae | 0 | 0 | 0 | 0 | 0 | 0 | 0 | 0 | 0 | 0 | 2.48E-05 | 0 |
| d__Bacteria;k__norank_d__Bacteria;p__Bdellovibrionota;c__Oligoflexia;o__Silvanigrellales;f__Silvanigrellaceae;g__norank_f__Silvanigrellaceae | 0 | 0 | 0 | 0 | 0 | 0 | 0 | 0 | 0 | 0 | 2.48E-05 | 0 |
| d__Bacteria;k__norank_d__Bacteria;p__Firmicutes;c__Bacilli;o__Paenibacillales;f__Paenibacillaceae;g__Ammoniibacillus | 0 | 0 | 0 | 0 | 0 | 0 | 0 | 0 | 0 | 0 | 2.48E-05 | 0 |
| d__Bacteria;k__norank_d__Bacteria;p__Patescibacteria;c__Parcubacteria;o__Candidatus_Terrybacteria;f__norank_o__Candidatus_Terrybacteria;g__norank_f__norank_o__Candidatus_Terrybacteria | 0 | 0 | 0 | 0 | 0 | 0 | 0 | 0 | 0 | 0 | 2.48E-05 | 0 |
| d__Bacteria;k__norank_d__Bacteria;p__Actinobacteriota;c__Actinobacteria;o__Corynebacteriales;f__Dietziaceae;g__Dietzia | 0 | 0 | 2.45E-05 | 0 | 0 | 0 | 0 | 0 | 0 | 0 | 0 | 0 |
| d__Bacteria;k__norank_d__Bacteria;p__Bacteroidota;c__Bacteroidia;o__Cytophagales;f__Spirosomaceae;g__Spirosoma | 0 | 0 | 2.45E-05 | 0 | 0 | 0 | 0 | 0 | 0 | 0 | 0 | 0 |
| d__Bacteria;k__norank_d__Bacteria;p__Firmicutes;c__Clostridia;o__Oscillospirales;f__Oscillospiraceae;g__norank_f__Oscillospiraceae | 0 | 0 | 2.45E-05 | 0 | 0 | 0 | 0 | 0 | 0 | 0 | 0 | 0 |
| d__Bacteria;k__norank_d__Bacteria;p__Patescibacteria;c__Parcubacteria;o__Candidatus_Zambryskibacteria;f__norank_o__Candidatus_Zambryskibacteria;g__norank_f__norank_o__Candidatus_Zambryskibacteria | 0 | 0 | 2.45E-05 | 0 | 0 | 0 | 0 | 0 | 0 | 0 | 0 | 0 |
| d__Bacteria;k__norank_d__Bacteria;p__Planctomycetota;c__Phycisphaerae;o__CCM11a;f__norank_o__CCM11a;g__norank_f__norank_o__CCM11a | 0 | 0 | 2.45E-05 | 0 | 0 | 0 | 0 | 0 | 0 | 0 | 0 | 0 |
| d__Bacteria;k__norank_d__Bacteria;p__Planctomycetota;c__Phycisphaerae;o__Tepidisphaerales;f__Tepidisphaeraceae;g__Tepidisphaera | 0 | 0 | 2.45E-05 | 0 | 0 | 0 | 0 | 0 | 0 | 0 | 0 | 0 |
| d__Bacteria;k__norank_d__Bacteria;p__Proteobacteria;c__Alphaproteobacteria;o__Rhizobiales;f__Beijerinckiaceae;g__Methylocystis | 0 | 0 | 2.45E-05 | 0 | 0 | 0 | 0 | 0 | 0 | 0 | 0 | 0 |
| d__Bacteria;k__norank_d__Bacteria;p__Proteobacteria;c__Gammaproteobacteria;o__Burkholderiales;f__Sulfuricellaceae;g__unclassified_f__Sulfuricellaceae | 0 | 0 | 2.45E-05 | 0 | 0 | 0 | 0 | 0 | 0 | 0 | 0 | 0 |
| d__Bacteria;k__norank_d__Bacteria;p__Bacteroidota;c__Bacteroidia;o__Cytophagales;f__Spirosomaceae;g__Lacihabitans | 0 | 0 | 0 | 0 | 0 | 0 | 0 | 0 | 0 | 2.40E-05 | 0 | 0 |
| d__Bacteria;k__norank_d__Bacteria;p__Bacteroidota;c__Bacteroidia;o__Flavobacteriales;f__Flavobacteriaceae;g__Pricia | 0 | 0 | 0 | 0 | 0 | 0 | 0 | 0 | 0 | 2.40E-05 | 0 | 0 |
| d__Bacteria;k__norank_d__Bacteria;p__Firmicutes;c__Bacilli;o__Alicyclobacillales;f__Alicyclobacillaceae;g__unclassified_f__Alicyclobacillaceae | 0 | 0 | 0 | 0 | 0 | 0 | 0 | 0 | 0 | 2.40E-05 | 0 | 0 |
| d__Bacteria;k__norank_d__Bacteria;p__Proteobacteria;c__Alphaproteobacteria;o__Paracaedibacterales;f__Paracaedibacteraceae;g__Candidatus_Finniella | 0 | 0 | 0 | 0 | 0 | 0 | 0 | 0 | 0 | 2.40E-05 | 0 | 0 |
| d__Bacteria;k__norank_d__Bacteria;p__Proteobacteria;c__Gammaproteobacteria;o__Burkholderiales;f__Chitinimonadaceae;g__Chitinimonas | 0 | 0 | 0 | 0 | 0 | 0 | 0 | 0 | 0 | 2.40E-05 | 0 | 0 |
| d__Bacteria;k__norank_d__Bacteria;p__Proteobacteria;c__Gammaproteobacteria;o__Xanthomonadales;f__Rhodanobacteraceae;g__Chujaibacter | 0 | 0 | 0 | 0 | 0 | 0 | 0 | 0 | 0 | 2.40E-05 | 0 | 0 |
| d__Bacteria;k__norank_d__Bacteria;p__Verrucomicrobiota;c__Verrucomicrobiae;o__Pedosphaerales;f__Pedosphaeraceae;g__DEV114 | 0 | 0 | 0 | 0 | 0 | 0 | 0 | 0 | 0 | 2.40E-05 | 0 | 0 |
| d__Bacteria;k__norank_d__Bacteria;p__Actinobacteriota;c__Actinobacteria;o__Micrococcales;f__Micrococcaceae;g__Paeniglutamicibacter | 0 | 0 | 0 | 0 | 2.26E-05 | 0 | 0 | 0 | 0 | 0 | 0 | 0 |
| d__Bacteria;k__norank_d__Bacteria;p__Actinobacteriota;c__RBG-16-55-12;o__norank_c__RBG-16-55-12;f__norank_o__norank_c__RBG-16-55-12;g__norank_f__norank_o__norank_c__RBG-16-55-12 | 0 | 0 | 0 | 0 | 2.26E-05 | 0 | 0 | 0 | 0 | 0 | 0 | 0 |
| d__Bacteria;k__norank_d__Bacteria;p__Bacteroidota;c__Bacteroidia;o__Cytophagales;f__Hymenobacteraceae;g__Rufibacter | 0 | 0 | 0 | 0 | 2.26E-05 | 0 | 0 | 0 | 0 | 0 | 0 | 0 |
| d__Bacteria;k__norank_d__Bacteria;p__Desulfobacterota;c__Desulfobulbia;o__Desulfobulbales;f__Desulfobulbaceae;g__norank_f__Desulfobulbaceae | 0 | 0 | 0 | 0 | 2.26E-05 | 0 | 0 | 0 | 0 | 0 | 0 | 0 |
| d__Bacteria;k__norank_d__Bacteria;p__Firmicutes;c__Thermacetogenia;o__Thermacetogeniales;f__Thermacetogeniaceae;g__Syntrophaceticus | 0 | 0 | 0 | 0 | 2.26E-05 | 0 | 0 | 0 | 0 | 0 | 0 | 0 |
| d__Bacteria;k__norank_d__Bacteria;p__Patescibacteria;c__Microgenomatia;o__Candidatus_Roizmanbacteria;f__norank_o__Candidatus_Roizmanbacteria;g__norank_f__norank_o__Candidatus_Roizmanbacteria | 0 | 0 | 0 | 0 | 2.26E-05 | 0 | 0 | 0 | 0 | 0 | 0 | 0 |
| d__Bacteria;k__norank_d__Bacteria;p__Proteobacteria;c__Alphaproteobacteria;o__Acetobacterales;f__Elioraeaceae;g__Elioraea | 0 | 0 | 0 | 0 | 2.26E-05 | 0 | 0 | 0 | 0 | 0 | 0 | 0 |
| d__Bacteria;k__norank_d__Bacteria;p__Proteobacteria;c__Alphaproteobacteria;o__Puniceispirillales;f__Puniceispirillales_Incertae_Sedis;g__Constrictibacter | 0 | 0 | 0 | 0 | 2.26E-05 | 0 | 0 | 0 | 0 | 0 | 0 | 0 |
| d__Bacteria;k__norank_d__Bacteria;p__Cyanobacteria;c__Cyanobacteriia;o__Cyanobacteriales;f__Coleofasciculaceae;g__Microcoleus_SAG_1449-1a | 0 | 0 | 0 | 0 | 0 | 2.21E-05 | 0 | 0 | 0 | 0 | 0 | 0 |
| d__Bacteria;k__norank_d__Bacteria;p__Planctomycetota;c__Phycisphaerae;o__Phycisphaerales;f__Phycisphaeraceae;g__unclassified_f__Phycisphaeraceae | 0 | 0 | 0 | 0 | 0 | 2.21E-05 | 0 | 0 | 0 | 0 | 0 | 0 |
| d__Bacteria;k__norank_d__Bacteria;p__Acidobacteriota;c__Aminicenantia;o__Aminicenantales;f__norank_o__Aminicenantales;g__norank_f__norank_o__Aminicenantales | 0 | 0 | 0 | 2.14E-05 | 0 | 0 | 0 | 0 | 0 | 0 | 0 | 0 |
| d__Bacteria;k__norank_d__Bacteria;p__Actinobacteriota;c__Actinobacteria;o__norank_c__Actinobacteria;f__norank_o__norank_c__Actinobacteria;g__norank_f__norank_o__norank_c__Actinobacteria | 0 | 0 | 0 | 2.14E-05 | 0 | 0 | 0 | 0 | 0 | 0 | 0 | 0 |
| d__Bacteria;k__norank_d__Bacteria;p__Firmicutes;c__Clostridia;o__Oscillospirales;f__Oscillospiraceae;g__UCG-002 | 0 | 0 | 0 | 2.14E-05 | 0 | 0 | 0 | 0 | 0 | 0 | 0 | 0 |
| d__Bacteria;k__norank_d__Bacteria;p__Firmicutes;c__Clostridia;o__Oscillospirales;f__Ruminococcaceae;g__norank_f__Ruminococcaceae | 0 | 0 | 0 | 2.14E-05 | 0 | 0 | 0 | 0 | 0 | 0 | 0 | 0 |
| d__Bacteria;k__norank_d__Bacteria;p__Firmicutes;c__Clostridia;o__Peptostreptococcales-Tissierellales;f__Thermotaleaceae;g__unclassified_f__Thermotaleaceae | 0 | 0 | 0 | 2.14E-05 | 0 | 0 | 0 | 0 | 0 | 0 | 0 | 0 |
| d__Bacteria;k__norank_d__Bacteria;p__Proteobacteria;c__Gammaproteobacteria;o__Enterobacterales;f__Enterobacteriaceae;g__Citrobacter | 0 | 0 | 0 | 2.14E-05 | 0 | 0 | 0 | 0 | 0 | 0 | 0 | 0 |
| d__Bacteria;k__norank_d__Bacteria;p__Verrucomicrobiota;c__Chlamydiae;o__Chlamydiales;f__Parachlamydiaceae;g__Candidatus_Metachlamydia | 0 | 0 | 0 | 2.14E-05 | 0 | 0 | 0 | 0 | 0 | 0 | 0 | 0 |
