# Supplementary material for: Soil microbial community succession and physicochemical property changes affect Ganoderma leucocontextum growth in the Dadu river basin
Source: Front Microbiol. 2026 Jan 7;16:1666459. doi: 10.3389/fmicb.2025.1666459 (PMC12819783; doi:10.3389/fmicb.2025.1666459)
Supplement: Supplementary file 3 [file Data_Sheet_3.doc]

Supplementary Table 3 Fungal abundance at phylum level

| #OTU ID | GCK_1 | GCK_2 | GCK_3 | G1p_1 | G1p_2 | G1p_3 | G1c_1 | G1c_2 | G1c_3 | G1m_1 | G1m_2 | G1m_3 |
| --- | --- | --- | --- | --- | --- | --- | --- | --- | --- | --- | --- | --- |
| p__Ascomycota | 0.646982011 | 0.663079452 | 0.68043767 | 0.840894252 | 0.835941194 | 0.851926064 | 0.851700925 | 0.86442128 | 0.846860436 | 0.210842695 | 0.269941689 | 0.303667515 |
| p__Basidiomycota | 0.078933742 | 0.067766846 | 0.067924443 | 0.06738411 | 0.057995812 | 0.070513542 | 0.064727469 | 0.050476169 | 0.058806313 | 0.739356553 | 0.65285814 | 0.61501227 |
| p__Mortierellomycota | 0.179863566 | 0.189071752 | 0.167278294 | 0.045793277 | 0.041267983 | 0.034783979 | 0.027376905 | 0.038071009 | 0.043631943 | 0.026521377 | 0.033905937 | 0.029898462 |
| p__unclassified_k__Fungi | 0.061665578 | 0.062678704 | 0.064142108 | 0.032532589 | 0.045680708 | 0.032577617 | 0.028907851 | 0.018393858 | 0.024450098 | 0.01805615 | 0.023279375 | 0.029087962 |
| p__Rozellomycota | 0.025283112 | 0.008532769 | 0.010919243 | 0.003962447 | 0.002116307 | 0.003894905 | 0.020847874 | 0.02532814 | 0.021883513 | 0.002634127 | 0.006641601 | 0.003016863 |
| p__Chytridiomycota | 0.003624738 | 0.004525294 | 0.006078754 | 0.004412725 | 0.010536506 | 0.002926807 | 0.004637864 | 0.002116307 | 0.00328703 | 0.001823626 | 0.011347007 | 0.017921066 |
| p__Zoopagomycota | 0.003444627 | 0.004165072 | 0.003084405 | 0.002386474 | 0.001936196 | 0.002521557 | 0.000360222 | 0.000157597 | 0.000202625 | 0.000225139 | 0.000585361 | 0.000630389 |
| p__Olpidiomycota | 2.25E-05 | 2.25E-05 | 0 | 0.001913682 | 0.000697931 | 0.000697931 | 0.000833014 | 0.0008105 | 0.000630389 | 0.00040525 | 0.000675417 | 0.000630389 |
| p__Glomeromycota | 0.00011257 | 2.25E-05 | 2.25E-05 | 0 | 0.00357971 | 2.25E-05 | 0.000180111 | 0.000135083 | 0.000157597 | 0 | 0.000675417 | 0 |
| p__Basidiobolomycota | 0 | 0 | 0 | 0.000450278 | 6.75E-05 | 9.01E-05 | 0.00011257 | 2.25E-05 | 0 | 2.25E-05 | 2.25E-05 | 2.25E-05 |
| p__Monoblepharomycota | 4.50E-05 | 0.00011257 | 6.75E-05 | 0.000135083 | 6.75E-05 | 0 | 0 | 4.50E-05 | 0 | 9.01E-05 | 6.75E-05 | 0.00011257 |
| p__Kickxellomycota | 0 | 0 | 4.50E-05 | 0 | 0.00011257 | 2.25E-05 | 0.000135083 | 0 | 2.25E-05 | 0 | 0 | 0 |
| p__Entorrhizomycota | 0 | 0 | 0 | 0 | 0 | 0 | 0.000180111 | 2.25E-05 | 6.75E-05 | 0 | 0 | 0 |
| p__Aphelidiomycota | 2.25E-05 | 0 | 0 | 9.01E-05 | 0 | 0 | 0 | 0 | 0 | 2.25E-05 | 0 | 0 |
| p__Mucoromycota | 0 | 2.25E-05 | 0 | 4.50E-05 | 0 | 2.25E-05 | 0 | 0 | 0 | 0 | 0 | 0 |
