# Supplementary material for: Soil microbial community succession and physicochemical property changes affect Ganoderma leucocontextum growth in the Dadu river basin
Source: Front Microbiol. 2026 Jan 7;16:1666459. doi: 10.3389/fmicb.2025.1666459 (PMC12819783; doi:10.3389/fmicb.2025.1666459)
Supplement: Supplementary file 4 [file Data_Sheet_4.doc]

Supplementary Table 4 Fungal abundance at genus level

| #OTU ID | GCK_1 | GCK_2 | GCK_3 | G1p_1 | G1p_2 | G1p_3 | G1c_1 | G1c_2 | G1c_3 | G1m_1 | G1m_2 | G1m_3 |
| --- | --- | --- | --- | --- | --- | --- | --- | --- | --- | --- | --- | --- |
| d__Eukaryota;k__Fungi;p__Basidiomycota;c__Agaricomycetes;o__Polyporales;f__Ganodermataceae;g__Ganoderma | 0.000945584 | 0.001418376 | 0.00144089 | 0.001170723 | 0.002138821 | 0.004727919 | 0.007362046 | 0.002544071 | 0.011347007 | 0.717585609 | 0.623477497 | 0.586869892 |
| d__Eukaryota;k__Fungi;p__Mortierellomycota;c__Mortierellomycetes;o__Mortierellales;f__Mortierellaceae;g__Mortierella | 0.179818538 | 0.188914154 | 0.167120697 | 0.045793277 | 0.041267983 | 0.034783979 | 0.027376905 | 0.037958439 | 0.043564401 | 0.026521377 | 0.033905937 | 0.029898462 |
| d__Eukaryota;k__Fungi;p__Ascomycota;c__Sordariomycetes;o__Hypocreales;f__Hypocreaceae;g__Trichoderma | 0.038341176 | 0.123601324 | 0.079766756 | 0.024877862 | 0.015939843 | 0.038521287 | 0.060202175 | 0.033320575 | 0.03744062 | 0.026183668 | 0.029290587 | 0.045207916 |
| d__Eukaryota;k__Fungi;p__Ascomycota;c__Sordariomycetes;o__Glomerellales;f__Plectosphaerellaceae;g__Gibellulopsis | 0.141252223 | 0.052840129 | 0.09876849 | 0.04050251 | 0.040997816 | 0.057928271 | 0.004007475 | 0.003602224 | 0.006123781 | 0.006011212 | 0.012607785 | 0.012787897 |
| d__Eukaryota;k__Fungi;p__unclassified_k__Fungi;c__unclassified_k__Fungi;o__unclassified_k__Fungi;f__unclassified_k__Fungi;g__unclassified_k__Fungi | 0.061665578 | 0.062678704 | 0.064142108 | 0.032532589 | 0.045680708 | 0.032577617 | 0.028907851 | 0.018393858 | 0.024450098 | 0.01805615 | 0.023279375 | 0.029087962 |
| d__Eukaryota;k__Fungi;p__Ascomycota;c__Leotiomycetes;o__Helotiales;f__Myxotrichaceae;g__Myxotrichum | 0 | 0 | 0 | 0.022739041 | 0.033072923 | 0.039106648 | 0.140441723 | 0.089402706 | 0.081860549 | 0.000202625 | 4.50E-05 | 0 |
| d__Eukaryota;k__Fungi;p__Ascomycota;c__Eurotiomycetes;o__Eurotiales;f__Aspergillaceae;g__Aspergillus | 0.000540334 | 2.25E-05 | 0.000135083 | 0.000427764 | 0.0008105 | 0.000787987 | 0.024630209 | 0.322804332 | 0.019474526 | 0.002836752 | 0.002904293 | 0.003962447 |
| d__Eukaryota;k__Fungi;p__Ascomycota;c__Sordariomycetes;o__Sordariales;f__Chaetomiaceae;g__Trichocladium | 0.015151856 | 0.022941666 | 0.037305536 | 0.030213657 | 0.038228606 | 0.04020983 | 0.008892991 | 0.014341356 | 0.008510255 | 0.01188734 | 0.028367517 | 0.027196794 |
| d__Eukaryota;k__Fungi;p__Ascomycota;c__Leotiomycetes;o__Helotiales;f__Helotiaceae;g__Scytalidium | 0 | 0 | 0 | 0 | 0 | 0 | 0.06859986 | 0.036450008 | 0.156133913 | 0.003332058 | 0.001350834 | 0.003894905 |
| d__Eukaryota;k__Fungi;p__Ascomycota;c__Leotiomycetes;o__Helotiales;f__unclassified_o__Helotiales;g__unclassified_o__Helotiales | 0.030708963 | 0.038566315 | 0.027264336 | 0.071526668 | 0.033725826 | 0.032892811 | 0.005921156 | 0.004637864 | 0.004075016 | 0.00450278 | 0.004570322 | 0.007992435 |
| d__Eukaryota;k__Fungi;p__Ascomycota;c__Sordariomycetes;o__Hypocreales;f__Nectriaceae;g__Fusicolla | 0.012382646 | 0.013868564 | 0.038273634 | 0.025665849 | 0.035189229 | 0.080914965 | 0.005808587 | 0.006258865 | 0.007587185 | 0.006506518 | 0.008105005 | 0.006438976 |
| d__Eukaryota;k__Fungi;p__Ascomycota;c__Leotiomycetes;o__Helotiales;f__Helotiaceae;g__unclassified_f__Helotiaceae | 0.000225139 | 0.000270167 | 6.75E-05 | 0.075669226 | 0.157799941 | 0.000337709 | 0.000180111 | 0.000225139 | 0.000157597 | 4.50E-05 | 4.50E-05 | 4.50E-05 |
| d__Eukaryota;k__Fungi;p__Basidiomycota;c__Tremellomycetes;o__Filobasidiales;f__Piskurozymaceae;g__Solicoccozyma | 0.055609339 | 0.047414278 | 0.048967738 | 0.010513992 | 0.014183758 | 0.010018687 | 0.004480267 | 0.006956796 | 0.005358309 | 0.005515906 | 0.006416462 | 0.008645338 |
| d__Eukaryota;k__Fungi;p__Ascomycota;c__Dothideomycetes;o__Capnodiales;f__Cladosporiaceae;g__Cladosporium | 0.037755814 | 0.051917059 | 0.071684265 | 0.012247563 | 0.011347007 | 0.016029898 | 0.000225139 | 0.000472792 | 0.000202625 | 0.00040525 | 0.00051782 | 0.000292681 |
| d__Eukaryota;k__Fungi;p__Ascomycota;c__Sordariomycetes;o__Hypocreales;f__Clavicipitaceae;g__Collarina | 0.001643515 | 0.00040525 | 0.000202625 | 0.000585361 | 0.001485918 | 0.001395862 | 0.067631763 | 0.046018416 | 0.046221041 | 0.000157597 | 0 | 0.000247653 |
| d__Eukaryota;k__Fungi;p__Basidiomycota;c__Tremellomycetes;o__Tremellales;f__Trimorphomycetaceae;g__Saitozyma | 0 | 0 | 0 | 0.028570142 | 0.023414458 | 0.034783979 | 0.023842223 | 0.01897922 | 0.019677151 | 0.003016863 | 0.010063714 | 0.003647252 |
| d__Eukaryota;k__Fungi;p__Ascomycota;c__Sordariomycetes;o__Glomerellales;f__Plectosphaerellaceae;g__Verticillium | 0.013283202 | 0.035549452 | 0.036179841 | 0.039556926 | 0.016795371 | 0.021545805 | 0 | 4.50E-05 | 6.75E-05 | 0 | 0.000180111 | 0.000135083 |
| d__Eukaryota;k__Fungi;p__Ascomycota;c__unclassified_p__Ascomycota;o__unclassified_p__Ascomycota;f__unclassified_p__Ascomycota;g__unclassified_p__Ascomycota | 0.004908031 | 0.002476529 | 0.003219488 | 0.005696017 | 0.009343269 | 0.009523381 | 0.0050206 | 0.003151946 | 0.088389581 | 0.005493392 | 0.01753833 | 0.008014949 |
| d__Eukaryota;k__Fungi;p__Ascomycota;c__Sordariomycetes;o__Hypocreales;f__Nectriaceae;g__Cosmospora | 0.01096427 | 0.008847964 | 0.016277551 | 0.017921066 | 0.022446361 | 0.02717428 | 0.00513317 | 0.00317446 | 0.003061891 | 0.010491478 | 0.014228786 | 0.019226873 |
| d__Eukaryota;k__Fungi;p__Ascomycota;c__Sordariomycetes;o__Glomerellales;f__Plectosphaerellaceae;g__Plectosphaerella | 0.01148209 | 0.015399509 | 0.018191233 | 0.021770944 | 0.0276921 | 0.035819619 | 0.006236351 | 0.002994349 | 0.006033726 | 0.002071279 | 0.006236351 | 0.003669766 |
| d__Eukaryota;k__Fungi;p__Ascomycota;c__Sordariomycetes;o__Hypocreales;f__Hypocreaceae;g__Hypomyces | 0.00173357 | 0.001688543 | 0.001193237 | 0.031857172 | 0.086408357 | 0.019452012 | 0.000697931 | 0.000472792 | 0.000720445 | 0.00450278 | 0.004300155 | 0.002566585 |
| d__Eukaryota;k__Fungi;p__Ascomycota;c__Leotiomycetes;o__Thelebolales;f__Pseudeurotiaceae;g__Pseudeurotium | 0.004953059 | 0.008645338 | 0.018326317 | 0.046378639 | 0.007632213 | 0.008802936 | 0.015061801 | 0.019114303 | 0.008802936 | 0.005268253 | 0.0042101 | 0.006776685 |
| d__Eukaryota;k__Fungi;p__Ascomycota;c__Sordariomycetes;o__Chaetosphaeriales;f__Chaetosphaeriaceae;g__Chaetosphaeria | 2.25E-05 | 0 | 2.25E-05 | 0 | 0 | 2.25E-05 | 0.052434879 | 0.037688273 | 0.053087782 | 0 | 6.75E-05 | 0 |
| d__Eukaryota;k__Fungi;p__Ascomycota;c__Sordariomycetes;o__unclassified_c__Sordariomycetes;f__unclassified_c__Sordariomycetes;g__unclassified_c__Sordariomycetes | 0.027827183 | 0.011459576 | 0.011504604 | 0.010468965 | 0.007609699 | 0.011392035 | 0.018506428 | 0.010266339 | 0.017110566 | 0.003827363 | 0.005245739 | 0.006574059 |
| d__Eukaryota;k__Fungi;p__Ascomycota;c__Eurotiomycetes;o__Eurotiales;f__Trichocomaceae;g__Talaromyces | 0.003467141 | 0.001215751 | 0.00132832 | 0.051399239 | 0.030123601 | 0.040412455 | 0.00317446 | 0.00195871 | 0.002949321 | 0.001418376 | 0.000382736 | 0.000742959 |
| d__Eukaryota;k__Fungi;p__Ascomycota;c__Sordariomycetes;o__Sordariales;f__Lasiosphaeriaceae;g__Schizothecium | 0.002454015 | 0.001913682 | 0.002071279 | 0.012900466 | 0.01713308 | 0.016705316 | 0.018641511 | 0.01373348 | 0.021860999 | 0.008127519 | 0.012652813 | 0.006303893 |
| d__Eukaryota;k__Fungi;p__Ascomycota;c__Sordariomycetes;o__Hypocreales;f__unclassified_o__Hypocreales;g__unclassified_o__Hypocreales | 0.002116307 | 0.004592836 | 0.002589099 | 0.0042101 | 0.003039377 | 0.006033726 | 0.036652633 | 0.022806583 | 0.031992255 | 0.00092307 | 0.001508431 | 0.000675417 |
| d__Eukaryota;k__Fungi;p__Ascomycota;c__Eurotiomycetes;o__Chaetothyriales;f__Herpotrichiellaceae;g__Exophiala | 0.016412635 | 0.010513992 | 0.013395772 | 0.009883603 | 0.011437062 | 0.015534593 | 0.006101268 | 0.003016863 | 0.007136907 | 0.003804849 | 0.006078754 | 0.00513317 |
| d__Eukaryota;k__Fungi;p__Ascomycota;c__Sordariomycetes;o__Xylariales;f__Bartaliniaceae;g__Truncatella | 0.004367697 | 0.00409753 | 0.005876129 | 0.001080667 | 0.001485918 | 0.083008758 | 4.50E-05 | 0.00011257 | 2.25E-05 | 0.00103564 | 0.00173357 | 0.000855528 |
| d__Eukaryota;k__Fungi;p__Ascomycota;c__Sordariomycetes;o__Sordariales;f__Lasiosphaeriaceae;g__Cercophora | 0.018821622 | 0.023639597 | 0.00513317 | 0.001621001 | 0.003624738 | 0.001643515 | 0.019947317 | 0.006754171 | 0.014431411 | 0.002904293 | 0.001080667 | 0.00195871 |
| d__Eukaryota;k__Fungi;p__Ascomycota;c__Sordariomycetes;o__Hypocreales;f__Clavicipitaceae;g__Metarhizium | 0.012787897 | 0.038386203 | 0.014521467 | 0.002589099 | 0.002791724 | 0.003039377 | 0.012900466 | 0.00317446 | 0.005605962 | 0.001530945 | 0.000945584 | 0.001215751 |
| d__Eukaryota;k__Fungi;p__Ascomycota;c__Sordariomycetes;o__Hypocreales;f__Nectriaceae;g__Fusarium | 0.010874215 | 0.005831101 | 0.008915505 | 0.021545805 | 0.005673503 | 0.00513317 | 0.009095617 | 0.009005561 | 0.008870478 | 0.002814238 | 0.002476529 | 0.004232614 |
| d__Eukaryota;k__Fungi;p__Ascomycota;c__Sordariomycetes;o__Microascales;f__Microascaceae;g__Cephalotrichum | 0.000202625 | 0.00040525 | 0.000472792 | 0.00686674 | 0.008059977 | 0.009793548 | 0.014949231 | 0.00542585 | 0.00553842 | 0.010919243 | 0.00974852 | 0.019992345 |
| d__Eukaryota;k__Fungi;p__Ascomycota;c__Sordariomycetes;o__Sordariales;f__unclassified_o__Sordariales;g__unclassified_o__Sordariales | 0.002206362 | 0.004142558 | 0.002949321 | 0.009320756 | 0.003669766 | 0.003422113 | 0.015602134 | 0.011054326 | 0.022311277 | 0.000675417 | 0.00051782 | 0.00092307 |
| d__Eukaryota;k__Fungi;p__Ascomycota;c__Sordariomycetes;o__Hypocreales;f__Bionectriaceae;g__Clonostachys | 0.013891078 | 0.002904293 | 0.001013126 | 0.028232434 | 0.009613436 | 0.006574059 | 0.004863003 | 0.002566585 | 0.004052502 | 0.000652903 | 0.000675417 | 0.000382736 |
| d__Eukaryota;k__Fungi;p__Ascomycota;c__Sordariomycetes;o__Chaetosphaeriales;f__Chaetosphaeriaceae;g__Gonytrichum | 0.000495306 | 0.000630389 | 0.000472792 | 0 | 0.00011257 | 0.000202625 | 0.01292298 | 0.014679064 | 0.008150033 | 0.009343269 | 0.008577797 | 0.017560844 |
| d__Eukaryota;k__Fungi;p__Ascomycota;c__Sordariomycetes;o__Hypocreales;f__Hypocreales_fam_Incertae_sedis;g__Acremonium | 0.021433235 | 0.008892991 | 0.009906117 | 0.005831101 | 0.006213837 | 0.008780422 | 0.002408988 | 0.001621001 | 0.00103564 | 0.00011257 | 0.000720445 | 0.000157597 |
| d__Eukaryota;k__Fungi;p__Rozellomycota;c__unclassified_p__Rozellomycota;o__unclassified_p__Rozellomycota;f__unclassified_p__Rozellomycota;g__unclassified_p__Rozellomycota | 0.00963595 | 0.00328703 | 0.005403337 | 0.003219488 | 0.001530945 | 0.002971835 | 0.012225049 | 0.007001824 | 0.009906117 | 0.002431501 | 0.006101268 | 0.002926807 |
| d__Eukaryota;k__Fungi;p__Ascomycota;c__Leotiomycetes;o__Thelebolales;f__Pseudeurotiaceae;g__Pseudogymnoascus | 0.006799199 | 0.019992345 | 0.01067159 | 0.003422113 | 0.01107684 | 0.003984961 | 0.002408988 | 0.000675417 | 0.002836752 | 0.001080667 | 0.001688543 | 0.001305806 |
| d__Eukaryota;k__Fungi;p__Ascomycota;c__Sordariomycetes;o__Hypocreales;f__Nectriaceae;g__unclassified_f__Nectriaceae | 0.003129432 | 0.008938019 | 0.00830763 | 0.002814238 | 0.009793548 | 0.003894905 | 0.006123781 | 0.004547808 | 0.005515906 | 0.007834838 | 0.00132832 | 0.001553459 |
| d__Eukaryota;k__Fungi;p__Ascomycota;c__Sordariomycetes;o__Hypocreales;f__Nectriaceae;g__Neonectria | 0.006236351 | 0.005605962 | 0.00646149 | 0.005650989 | 0.007542157 | 0.026498863 | 0.000855528 | 0.000427764 | 0.000225139 | 0.000968098 | 0.001778598 | 0.001170723 |
| d__Eukaryota;k__Fungi;p__Ascomycota;c__Sordariomycetes;o__Microascales;f__Microascaceae;g__Lophotrichus | 0.002476529 | 0.002273904 | 0.004435239 | 0.035189229 | 0.002296418 | 0.002499043 | 0.001238265 | 0.002161335 | 0.001260779 | 0.001913682 | 0.002566585 | 0.003377085 |
| d__Eukaryota;k__Fungi;p__Ascomycota;c__Sordariomycetes;o__Hypocreales;f__Hypocreales_fam_Incertae_sedis;g__Sarocladium | 0.007069365 | 0.008082491 | 0.007362046 | 0.011392035 | 0.010446451 | 0.011301979 | 0.001688543 | 0.000720445 | 0.001238265 | 0.00051782 | 0.000427764 | 0.000697931 |
| d__Eukaryota;k__Fungi;p__Ascomycota;c__Eurotiomycetes;o__Eurotiales;f__Aspergillaceae;g__Penicillium | 0.00317446 | 0.001395862 | 0.001305806 | 0.00542585 | 0.00871288 | 0.023121778 | 0.005088142 | 0.002273904 | 0.003219488 | 0.001621001 | 0.001756084 | 0.001373348 |
| d__Eukaryota;k__Fungi;p__Chytridiomycota;c__unclassified_p__Chytridiomycota;o__unclassified_p__Chytridiomycota;f__unclassified_p__Chytridiomycota;g__unclassified_p__Chytridiomycota | 0.002566585 | 0.002544071 | 0.004592836 | 0.003467141 | 0.010288853 | 0.001485918 | 0.002701668 | 0.001238265 | 0.002093793 | 0.000900556 | 0.008600311 | 0.015759732 |
| d__Eukaryota;k__Fungi;p__Rozellomycota;c__Rozellomycotina_cls_Incertae_sedis;o__GS11;f__unclassified_o__GS11;g__unclassified_o__GS11 | 0.009253214 | 0.003016863 | 0.003737308 | 0.000382736 | 0.000180111 | 0.000382736 | 0.007947408 | 0.017268163 | 0.01159466 | 9.01E-05 | 0.000157597 | 9.01E-05 |
| d__Eukaryota;k__Fungi;p__Ascomycota;c__Sordariomycetes;o__Sordariales;f__Chaetomiaceae;g__unclassified_f__Chaetomiaceae | 0.010266339 | 0.008352658 | 0.009726006 | 0.003849877 | 0.005358309 | 0.005628476 | 0.000472792 | 0.001373348 | 0.001530945 | 0.001418376 | 0.001621001 | 0.00265664 |
| d__Eukaryota;k__Fungi;p__Ascomycota;c__Sordariomycetes;o__Hypocreales;f__Hypocreales_fam_Incertae_sedis;g__Emericellopsis | 0.004390211 | 0.004052502 | 0.002949321 | 0.009838575 | 0.00911813 | 0.002431501 | 0.001058153 | 0.001283292 | 0.000945584 | 0.003849877 | 0.004885517 | 0.007181935 |
| d__Eukaryota;k__Fungi;p__Ascomycota;c__Pezizomycetes;o__Pezizales;f__Pyronemataceae;g__Cheilymenia | 0.025665849 | 0.000450278 | 0.000630389 | 0.003129432 | 0.007249477 | 0.008059977 | 0 | 9.01E-05 | 9.01E-05 | 0.001193237 | 0.000607875 | 0.001823626 |
| d__Eukaryota;k__Fungi;p__Ascomycota;c__Dothideomycetes;o__Pleosporales;f__Didymosphaeriaceae;g__Paraphaeosphaeria | 0.011662201 | 0.007181935 | 0.010356395 | 0.000585361 | 0.000833014 | 0.000585361 | 0.007159421 | 0.004863003 | 0.005200711 | 4.50E-05 | 0.000157597 | 6.75E-05 |
| d__Eukaryota;k__Fungi;p__Ascomycota;c__Sordariomycetes;o__Hypocreales;f__Nectriaceae;g__Neocosmospora | 0.007609699 | 0.000292681 | 0.001215751 | 0.007632213 | 0.00276921 | 0.001936196 | 0.008645338 | 0.00871288 | 0.007339532 | 0.001080667 | 0.00051782 | 0.000945584 |
| d__Eukaryota;k__Fungi;p__Ascomycota;c__Dothideomycetes;o__Tubeufiales;f__Tubeufiaceae;g__Titaea | 0.00963595 | 0.0134408 | 0.007632213 | 0.002138821 | 0.002003737 | 0.002318932 | 0.000427764 | 0.000225139 | 0.000270167 | 0.001913682 | 0.003962447 | 0.002859266 |
| d__Eukaryota;k__Fungi;p__Ascomycota;c__Sordariomycetes;o__Sordariales;f__Sordariales_fam_Incertae_sedis;g__Staphylotrichum | 0 | 0 | 0 | 0.002273904 | 0.00173357 | 0.002296418 | 0.004277641 | 0.003782336 | 0.008645338 | 0.002611613 | 0.010784159 | 0.004953059 |
| d__Eukaryota;k__Fungi;p__Ascomycota;c__Dothideomycetes;o__Dothideomycetes_ord_Incertae_sedis;f__Eremomycetaceae;g__Arthrographis | 6.75E-05 | 2.25E-05 | 0.000225139 | 0.000900556 | 0.000540334 | 0.007249477 | 0.011256951 | 0.009523381 | 0.004187586 | 0.001058153 | 0.004322669 | 0.001823626 |
| d__Eukaryota;k__Fungi;p__Ascomycota;c__Eurotiomycetes;o__Onygenales;f__Onygenales_fam_Incertae_sedis;g__Chrysosporium | 0.002206362 | 0.000562848 | 0.000945584 | 0.002454015 | 0.002386474 | 0.008397686 | 0.011752257 | 0.0042101 | 0.006213837 | 9.01E-05 | 0.00011257 | 0.000562848 |
| d__Eukaryota;k__Fungi;p__Ascomycota;c__Leotiomycetes;o__Helotiales;f__Helotiales_fam_Incertae_sedis;g__Mycoarthris | 0.005290767 | 0.016817885 | 0.006011212 | 0.001666029 | 0.001463404 | 0.004953059 | 0.000202625 | 0.00011257 | 0.000382736 | 0.00132832 | 0.000585361 | 0.001013126 |
| d__Eukaryota;k__Fungi;p__Ascomycota;c__Dothideomycetes;o__Pleosporales;f__Didymellaceae;g__Phoma | 0.004345183 | 0.001598487 | 0.002611613 | 0.002228876 | 0.004727919 | 0.005650989 | 0.0008105 | 0.000607875 | 0.000742959 | 0.002296418 | 0.004637864 | 0.004750433 |
| d__Eukaryota;k__Fungi;p__Basidiomycota;c__Tremellomycetes;o__Cystofilobasidiales;f__Mrakiaceae;g__Tausonia | 0.010874215 | 0.00144089 | 0.001621001 | 6.75E-05 | 0.000855528 | 0.00173357 | 0.000270167 | 0.000202625 | 0.000225139 | 0.002454015 | 0.004930545 | 0.008938019 |
| d__Eukaryota;k__Fungi;p__Ascomycota;c__Leotiomycetes;o__Helotiales;f__Myxotrichaceae;g__Oidiodendron | 0.000540334 | 0.001058153 | 0.001125695 | 0.005808587 | 0.004705406 | 0.004075016 | 0.002341446 | 0.005515906 | 0.002093793 | 0.002926807 | 0.000990612 | 0.002273904 |
| d__Eukaryota;k__Fungi;p__Ascomycota;c__Eurotiomycetes;o__Eurotiales;f__Trichocomaceae;g__Thermomyces | 0 | 2.25E-05 | 0 | 6.75E-05 | 0 | 2.25E-05 | 0.011437062 | 0.005110656 | 0.014116217 | 0.000540334 | 0.000878042 | 0.000585361 |
| d__Eukaryota;k__Fungi;p__Ascomycota;c__Sordariomycetes;o__Microascales;f__Microascaceae;g__Parascedosporium | 0 | 2.25E-05 | 0 | 2.25E-05 | 0.000135083 | 0 | 0.015084315 | 0.007519643 | 0.00974852 | 0 | 0 | 0 |
| d__Eukaryota;k__Fungi;p__Ascomycota;c__Dothideomycetes;o__Pleosporales;f__Didymellaceae;g__Epicoccum | 0.010378909 | 0.010513992 | 0.001981223 | 0.006146295 | 0.000382736 | 0.00144089 | 0.000585361 | 9.01E-05 | 0.00011257 | 0.000337709 | 0.000247653 | 0.000180111 |
| d__Eukaryota;k__Fungi;p__Ascomycota;c__Sordariomycetes;o__Coniochaetales;f__Coniochaetaceae;g__Coniochaeta | 0.001891168 | 0.004817975 | 0.004525294 | 0.003196974 | 0.001305806 | 0.001418376 | 0.001418376 | 0.00409753 | 0.002318932 | 0.001868654 | 0.001868654 | 0.00225139 |
| d__Eukaryota;k__Fungi;p__Ascomycota;c__Dothideomycetes;o__Pleosporales;f__unclassified_o__Pleosporales;g__unclassified_o__Pleosporales | 0.001711057 | 0.000697931 | 0.006574059 | 0.005245739 | 0.004660378 | 0.001913682 | 0.000675417 | 0.000787987 | 0.000720445 | 0.001103181 | 0.000900556 | 0.000697931 |
| d__Eukaryota;k__Fungi;p__Ascomycota;c__Sordariomycetes;o__Sordariales;f__Chaetomiaceae;g__Chaetomium | 0.000180111 | 0.000495306 | 0.000247653 | 0.001801112 | 0.001463404 | 0.000900556 | 0.009928631 | 0.004277641 | 0.004998086 | 0.00051782 | 0.000180111 | 0.000540334 |
| d__Eukaryota;k__Fungi;p__Ascomycota;c__Sordariomycetes;o__Hypocreales;f__Nectriaceae;g__Acremoniopsis | 0 | 0 | 0 | 0 | 0 | 0 | 0.009861089 | 0.004975572 | 0.009410811 | 0 | 0 | 0 |
| d__Eukaryota;k__Fungi;p__Ascomycota;c__Dothideomycetes;o__Pleosporales;f__Didymellaceae;g__Boeremia | 0.000247653 | 0.000180111 | 0.00011257 | 0.001666029 | 0.018889164 | 0.001463404 | 0.000562848 | 0.000382736 | 0.000337709 | 0.00011257 | 4.50E-05 | 0 |
| d__Eukaryota;k__Fungi;p__Ascomycota;c__Sordariomycetes;o__Glomerellales;f__Plectosphaerellaceae;g__Chordomyces | 0.004412725 | 0.003084405 | 0.004727919 | 0.001395862 | 0.00144089 | 0.002746696 | 0.000360222 | 0.000427764 | 0.001283292 | 0.000202625 | 0.000787987 | 0.001125695 |
| d__Eukaryota;k__Fungi;p__Ascomycota;c__Sordariomycetes;o__Hypocreales;f__Nectriaceae;g__Gibberella | 0.000900556 | 0.000607875 | 0.0008105 | 0.00195871 | 0.001801112 | 0.004592836 | 0.001215751 | 0.000878042 | 0.00144089 | 0.001508431 | 0.003106919 | 0.002499043 |
| d__Eukaryota;k__Fungi;p__Ascomycota;c__Sordariomycetes;o__Hypocreales;f__Nectriaceae;g__Dactylonectria | 0.00236396 | 0.002003737 | 0.002926807 | 0.003557197 | 0.003894905 | 0.002679154 | 0.000495306 | 0.000315195 | 0.000360222 | 0.00040525 | 0.000495306 | 0.000607875 |
| d__Eukaryota;k__Fungi;p__Ascomycota;c__Sordariomycetes;o__Chaetosphaeriales;f__Chaetosphaeriaceae;g__unclassified_f__Chaetosphaeriaceae | 6.75E-05 | 4.50E-05 | 6.75E-05 | 6.75E-05 | 4.50E-05 | 0.000427764 | 0.008622825 | 0.004120044 | 0.00605624 | 0.00011257 | 0.000202625 | 0.000157597 |
| d__Eukaryota;k__Fungi;p__Basidiomycota;c__Tremellomycetes;o__Tremellales;f__Bulleribasidiaceae;g__Vishniacozyma | 0.003084405 | 0.004930545 | 0.003129432 | 0.001485918 | 0.001598487 | 0.004457753 | 0 | 4.50E-05 | 4.50E-05 | 0 | 0.000135083 | 6.75E-05 |
| d__Eukaryota;k__Fungi;p__Basidiomycota;c__Dacrymycetes;o__Dacrymycetales;f__Dacrymycetaceae;g__Calocera | 0 | 0 | 0 | 0 | 0 | 0 | 0.008735394 | 0.00697931 | 0.003106919 | 0 | 0 | 0 |
| d__Eukaryota;k__Fungi;p__Zoopagomycota;c__Zoopagomycetes;o__Zoopagales;f__Piptocephalidaceae;g__Syncephalis | 0.003354571 | 0.003984961 | 0.002949321 | 0.001598487 | 0.001463404 | 0.00184614 | 0.000247653 | 0.000135083 | 4.50E-05 | 0.000225139 | 0.000472792 | 0.000472792 |
| d__Eukaryota;k__Fungi;p__Ascomycota;c__Leotiomycetes;o__Helotiales;f__Helotiales_fam_Incertae_sedis;g__Cadophora | 0.005403337 | 0.001621001 | 0.001058153 | 0.001305806 | 0.001530945 | 0.001936196 | 0.001530945 | 0.000990612 | 0.0008105 | 0.00011257 | 0.00011257 | 0.000135083 |
| d__Eukaryota;k__Fungi;p__Ascomycota;c__Leotiomycetes;o__Helotiales;f__Helotiaceae;g__Tetracladium | 0.006911768 | 0.002048765 | 0.004277641 | 0.000607875 | 0.000315195 | 0.000652903 | 0.000135083 | 0.000202625 | 4.50E-05 | 9.01E-05 | 0.000270167 | 0.000202625 |
| d__Eukaryota;k__Fungi;p__Ascomycota;c__Leotiomycetes;o__Helotiales;f__Sclerotiniaceae;g__Botrytis | 0.002634127 | 0.004390211 | 0.004998086 | 0.00103564 | 0.000990612 | 0.00103564 | 0.000135083 | 6.75E-05 | 2.25E-05 | 2.25E-05 | 0.000157597 | 0 |
| d__Eukaryota;k__Fungi;p__Ascomycota;c__Sordariomycetes;o__Hypocreales;f__Nectriaceae;g__Mariannaea | 0.000968098 | 0.001463404 | 0.007497129 | 0.000180111 | 0.000135083 | 9.01E-05 | 0.002026251 | 0.000337709 | 0.000968098 | 0.00103564 | 0.000157597 | 0.00011257 |
| d__Eukaryota;k__Fungi;p__Ascomycota;c__Dothideomycetes;o__Pleosporales;f__Didymellaceae;g__Didymella | 0.000270167 | 0.000427764 | 0.000315195 | 0.006281379 | 0.001936196 | 0.002408988 | 0.001013126 | 0.000360222 | 0.000450278 | 0.000382736 | 0.000495306 | 0.000630389 |
| d__Eukaryota;k__Fungi;p__Ascomycota;c__Sordariomycetes;o__Microascales;f__Microascaceae;g__Microascus | 2.25E-05 | 2.25E-05 | 0 | 0.00051782 | 0.00103564 | 0.000382736 | 0.005786073 | 0.00173357 | 0.005200711 | 0 | 6.75E-05 | 0 |
| d__Eukaryota;k__Fungi;p__Ascomycota;c__Dothideomycetes;o__Capnodiales;f__unclassified_o__Capnodiales;g__unclassified_o__Capnodiales | 0.001643515 | 0.001170723 | 0.00225139 | 0.003264516 | 0.002161335 | 0.001058153 | 0.00092307 | 0.000833014 | 0.000562848 | 0.000135083 | 0.000270167 | 0.000247653 |
| d__Eukaryota;k__Fungi;p__Ascomycota;c__Sordariomycetes;o__Sordariales;f__Lasiosphaeriaceae;g__Cladorrhinum | 2.25E-05 | 2.25E-05 | 0.000202625 | 0.002589099 | 0.001868654 | 0.000878042 | 0.000787987 | 0.000472792 | 0.000675417 | 0.002791724 | 0.000742959 | 0.003332058 |
| d__Eukaryota;k__Fungi;p__Basidiomycota;c__Tremellomycetes;o__Trichosporonales;f__Trichosporonaceae;g__Apiotrichum | 0 | 0 | 0 | 0.000337709 | 0.000630389 | 0.001350834 | 0.003894905 | 0.00265664 | 0.003264516 | 0.000382736 | 0.000157597 | 0.000607875 |
| d__Eukaryota;k__Fungi;p__Ascomycota;c__Dothideomycetes;o__Pleosporales;f__Phaeosphaeriaceae;g__Paraphoma | 0.000427764 | 0.000742959 | 0.000157597 | 0.001756084 | 0.000585361 | 0.007181935 | 0.000472792 | 0.000202625 | 0.000787987 | 4.50E-05 | 0.000472792 | 0.000225139 |
| d__Eukaryota;k__Fungi;p__Ascomycota;c__Sordariomycetes;o__Hypocreales;f__Nectriaceae;g__Cylindrocarpon | 0.000990612 | 0.000878042 | 0.001553459 | 0.001530945 | 0.000833014 | 0.001103181 | 0.000765473 | 0.000472792 | 0.000765473 | 0.00092307 | 0.001305806 | 0.001643515 |
| d__Eukaryota;k__Fungi;p__Rozellomycota;c__Rozellomycotina_cls_Incertae_sedis;o__GS05;f__unclassified_o__GS05;g__unclassified_o__GS05 | 0.006371434 | 0.002206362 | 0.001756084 | 0.000270167 | 0.000337709 | 0.000540334 | 0.000135083 | 0.000562848 | 0.000157597 | 0.00011257 | 0.000135083 | 0 |
| d__Eukaryota;k__Fungi;p__Basidiomycota;c__Agaricomycetes;o__Agaricales;f__Psathyrellaceae;g__Coprinopsis | 2.25E-05 | 4.50E-05 | 9.01E-05 | 0.00092307 | 0.004435239 | 0.000833014 | 0.00040525 | 0.00040525 | 0.000337709 | 0.001485918 | 0.002138821 | 0.00103564 |
| d__Eukaryota;k__Fungi;p__Ascomycota;c__Eurotiomycetes;o__Onygenales;f__Arachnomycetaceae;g__Arachnomyces | 0.000270167 | 0 | 0.000337709 | 9.01E-05 | 0.001125695 | 0.000472792 | 0.002746696 | 0.002341446 | 0.003151946 | 6.75E-05 | 0.00011257 | 0 |
| d__Eukaryota;k__Fungi;p__Basidiomycota;c__Tremellomycetes;o__Tremellales;f__unclassified_o__Tremellales;g__unclassified_o__Tremellales | 0.001373348 | 0.002476529 | 0.001643515 | 0.002026251 | 0.000990612 | 0.001756084 | 0 | 0 | 0 | 0 | 0 | 6.75E-05 |
| d__Eukaryota;k__Fungi;p__Ascomycota;c__Sordariomycetes;o__Hypocreales;f__Cordycipitaceae;g__Simplicillium | 0.000270167 | 0.00040525 | 0.002228876 | 0.002634127 | 0.001283292 | 0.003377085 | 0 | 0 | 0 | 0 | 0 | 0 |
| d__Eukaryota;k__Fungi;p__Basidiomycota;c__unclassified_p__Basidiomycota;o__unclassified_p__Basidiomycota;f__unclassified_p__Basidiomycota;g__unclassified_p__Basidiomycota | 0.001395862 | 0.00103564 | 0.001103181 | 0.001148209 | 0.001058153 | 0.000990612 | 0.000607875 | 0.000562848 | 0.000540334 | 0.000652903 | 0.000495306 | 0.000562848 |
| d__Eukaryota;k__Fungi;p__Ascomycota;c__Sordariomycetes;o__Hypocreales;f__Bionectriaceae;g__Bionectria | 0 | 0 | 0 | 0.001553459 | 0.003399599 | 0.004975572 | 2.25E-05 | 2.25E-05 | 0 | 0 | 0 | 0 |
| d__Eukaryota;k__Fungi;p__Ascomycota;c__Dothideomycetes;o__unclassified_c__Dothideomycetes;f__unclassified_c__Dothideomycetes;g__unclassified_c__Dothideomycetes | 0.000157597 | 9.01E-05 | 4.50E-05 | 0.000720445 | 0.007249477 | 0.000562848 | 0.00040525 | 0.000382736 | 0.000225139 | 6.75E-05 | 0 | 0 |
| d__Eukaryota;k__Fungi;p__Ascomycota;c__Eurotiomycetes;o__Chaetothyriales;f__Herpotrichiellaceae;g__Phialophora | 0 | 0 | 6.75E-05 | 6.75E-05 | 0 | 0 | 0.007249477 | 0.00051782 | 0.001575973 | 0 | 0 | 0 |
| d__Eukaryota;k__Fungi;p__Ascomycota;c__Dothideomycetes;o__Pleosporales;f__Cucurbitariaceae;g__Pyrenochaetopsis | 0 | 9.01E-05 | 4.50E-05 | 0.001485918 | 0.000540334 | 0.000157597 | 0.001688543 | 0.002183849 | 0.002971835 | 9.01E-05 | 2.25E-05 | 0.000135083 |
| d__Eukaryota;k__Fungi;p__Ascomycota;c__Sordariomycetes;o__Microascales;f__Microascaceae;g__Acaulium | 2.25E-05 | 0.000270167 | 0.00011257 | 0.003084405 | 0.001621001 | 0.000765473 | 0.000765473 | 0.000720445 | 0.000833014 | 0.000585361 | 0.000337709 | 0.000225139 |
| d__Eukaryota;k__Fungi;p__Ascomycota;c__Eurotiomycetes;o__Chaetothyriales;f__Cyphellophoraceae;g__Cyphellophora | 0.000495306 | 0.000427764 | 0.000742959 | 0.00092307 | 0.000900556 | 0.001598487 | 0.000180111 | 0.000180111 | 0.003512169 | 0 | 0.000360222 | 0 |
| d__Eukaryota;k__Fungi;p__Ascomycota;c__Sordariomycetes;o__Ophiostomatales;f__Ophiostomataceae;g__Sporothrix | 0.000157597 | 0.000270167 | 6.75E-05 | 0.000427764 | 0.0008105 | 0.000787987 | 0.001125695 | 0.00040525 | 0.001058153 | 0.002701668 | 0.000360222 | 0.001148209 |
| d__Eukaryota;k__Fungi;p__Ascomycota;c__Sordariomycetes;o__Sordariales;f__Lasiosphaeriaceae;g__unclassified_f__Lasiosphaeriaceae | 0.000180111 | 0.000675417 | 9.01E-05 | 0.001080667 | 0.00092307 | 0.00195871 | 0.00040525 | 0.000697931 | 0.000945584 | 0.000765473 | 0.000990612 | 0.000427764 |
| d__Eukaryota;k__Fungi;p__Ascomycota;c__Dothideomycetes;o__Pleosporales;f__Pleosporales_fam_Incertae_sedis;g__Monodictys | 0.000202625 | 0.000427764 | 0.001913682 | 0.002408988 | 0.001891168 | 0.000945584 | 0.000472792 | 6.75E-05 | 9.01E-05 | 0.000135083 | 0.000225139 | 0.000292681 |
| d__Eukaryota;k__Fungi;p__Ascomycota;c__Sordariomycetes;o__Sordariales;f__Sordariales_fam_Incertae_sedis;g__Ramophialophora | 0.00011257 | 9.01E-05 | 2.25E-05 | 0.000315195 | 2.25E-05 | 0 | 0.002206362 | 0.001125695 | 0.001418376 | 0.000427764 | 0.002341446 | 0.000855528 |
| d__Eukaryota;k__Fungi;p__Basidiomycota;c__Tremellomycetes;o__Trichosporonales;f__Trichosporonaceae;g__Cutaneotrichosporon | 0 | 0 | 0 | 2.25E-05 | 0 | 4.50E-05 | 0.003557197 | 0.003039377 | 0.002206362 | 0 | 0 | 0 |
| d__Eukaryota;k__Fungi;p__Ascomycota;c__Pezizomycetes;o__Pezizales;f__Pezizaceae;g__Peziza | 0.001238265 | 0.000495306 | 0.000270167 | 6.75E-05 | 0.000270167 | 0.000787987 | 0.000157597 | 9.01E-05 | 4.50E-05 | 0.000450278 | 0.000292681 | 0.004525294 |
| d__Eukaryota;k__Fungi;p__Ascomycota;c__Pezizomycetes;o__Pezizales;f__Pyronemataceae;g__Tricharina | 0.001373348 | 0.001530945 | 0.00040525 | 0.001756084 | 0.001080667 | 0.002228876 | 0 | 0 | 0.000157597 | 0 | 4.50E-05 | 0 |
| d__Eukaryota;k__Fungi;p__Ascomycota;c__Sordariomycetes;o__Sordariales;f__Chaetomiaceae;g__Myceliophthora | 0 | 0 | 4.50E-05 | 0 | 0 | 0 | 0.003984961 | 0.001801112 | 0.002431501 | 4.50E-05 | 0 | 0.000247653 |
| d__Eukaryota;k__Fungi;p__Ascomycota;c__Dothideomycetes;o__Pleosporales;f__Cucurbitariaceae;g__Pyrenochaeta | 0 | 0 | 0 | 0 | 0 | 4.50E-05 | 4.50E-05 | 2.25E-05 | 2.25E-05 | 0.005110656 | 0.000427764 | 0.002071279 |
| d__Eukaryota;k__Fungi;p__Ascomycota;c__Dothideomycetes;o__Pleosporales;f__Periconiaceae;g__Periconia | 0.000180111 | 0.000787987 | 6.75E-05 | 0.000945584 | 0.002926807 | 0.00195871 | 0.000157597 | 0.000180111 | 0.000202625 | 2.25E-05 | 4.50E-05 | 9.01E-05 |
| d__Eukaryota;k__Fungi;p__Ascomycota;c__Pezizomycetes;o__Pezizales;f__Pyronemataceae;g__Pseudaleuria | 0.000990612 | 0.000630389 | 0 | 0.000292681 | 0 | 0 | 0 | 0.001350834 | 0.000900556 | 0.001013126 | 0.001350834 | 0.000720445 |
| d__Eukaryota;k__Fungi;p__Ascomycota;c__Pezizomycetes;o__Pezizales;f__Pyronemataceae;g__unclassified_f__Pyronemataceae | 0.000427764 | 0.000135083 | 0.000135083 | 0.000135083 | 0.00011257 | 0.000720445 | 0.000270167 | 0.000225139 | 0.000157597 | 0 | 0.000787987 | 0.004007475 |
| d__Eukaryota;k__Fungi;p__Ascomycota;c__Eurotiomycetes;o__Chaetothyriales;f__Herpotrichiellaceae;g__Cladophialophora | 0.000315195 | 0.000697931 | 0.000427764 | 0 | 0 | 6.75E-05 | 0.001711057 | 0.000765473 | 0.003084405 | 0 | 0 | 0 |
| d__Eukaryota;k__Fungi;p__Ascomycota;c__Sordariomycetes;o__Microascales;f__Microascaceae;g__Pseudallescheria | 0.000247653 | 2.25E-05 | 2.25E-05 | 0.000630389 | 0.00011257 | 2.25E-05 | 0.000225139 | 0.004750433 | 0.000157597 | 0.000450278 | 6.75E-05 | 0.000247653 |
| d__Eukaryota;k__Fungi;p__Ascomycota;c__Sordariomycetes;o__Microascales;f__unclassified_o__Microascales;g__unclassified_o__Microascales | 0.003714794 | 0.000247653 | 0.000247653 | 0.000878042 | 0 | 0.001485918 | 0.00011257 | 4.50E-05 | 4.50E-05 | 2.25E-05 | 4.50E-05 | 9.01E-05 |
| d__Eukaryota;k__Fungi;p__Ascomycota;c__Sordariomycetes;o__Glomerellales;f__Plectosphaerellaceae;g__Acrostalagmus | 0.00011257 | 0.000135083 | 0.000247653 | 9.01E-05 | 0.000652903 | 0.000337709 | 0.002431501 | 0.001080667 | 0.001508431 | 9.01E-05 | 6.75E-05 | 6.75E-05 |
| d__Eukaryota;k__Fungi;p__Chytridiomycota;c__Rhizophydiomycetes;o__Rhizophydiales;f__Alphamycetaceae;g__unclassified_f__Alphamycetaceae | 0 | 0 | 0.000652903 | 0.000742959 | 0.000225139 | 0.00011257 | 2.25E-05 | 0 | 0.000247653 | 0.000855528 | 0.001913682 | 0.001756084 |
| d__Eukaryota;k__Fungi;p__Ascomycota;c__Pezizomycetes;o__Pezizales;f__Pyronemataceae;g__Pseudombrophila | 0 | 0 | 0 | 0 | 0 | 0 | 0.000202625 | 2.25E-05 | 0.000630389 | 0.001080667 | 0.000990612 | 0.00357971 |
| d__Eukaryota;k__Fungi;p__Ascomycota;c__Pezizomycetes;o__Pezizales;f__Pyronemataceae;g__Scutellinia | 0.000382736 | 0.000337709 | 0.000315195 | 0.002341446 | 0.001418376 | 0.000765473 | 2.25E-05 | 0.000562848 | 0.00011257 | 0 | 6.75E-05 | 4.50E-05 |
| d__Eukaryota;k__Fungi;p__Ascomycota;c__Dothideomycetes;o__Pleosporales;f__Melanommataceae;g__unclassified_f__Melanommataceae | 0.000878042 | 0.000315195 | 0.00040525 | 0 | 0 | 0 | 0.00040525 | 0.003827363 | 0.000540334 | 0 | 0 | 0 |
| d__Eukaryota;k__Fungi;p__Ascomycota;c__Sordariomycetes;o__Hypocreales;f__Nectriaceae;g__Ilyonectria | 0.001463404 | 0.000787987 | 0.00092307 | 0.00144089 | 0.000360222 | 0.000135083 | 0.000472792 | 0.000225139 | 0.00051782 | 2.25E-05 | 0 | 0 |
| d__Eukaryota;k__Fungi;p__Ascomycota;c__Saccharomycetes;o__Saccharomycetales;f__Dipodascaceae;g__Geotrichum | 0 | 0 | 0 | 0.000225139 | 0.000135083 | 0 | 0.005786073 | 0 | 0 | 0 | 0 | 0 |
| d__Eukaryota;k__Fungi;p__Basidiomycota;c__Agaricomycetes;o__Agaricales;f__Psathyrellaceae;g__Coprinellus | 0.000270167 | 0.00011257 | 0.000135083 | 0.001395862 | 0.000720445 | 0.001148209 | 0.000427764 | 0.000697931 | 0.000427764 | 0.000202625 | 0.000337709 | 0.000270167 |
| d__Eukaryota;k__Fungi;p__Ascomycota;c__Sordariomycetes;o__Hypocreales;f__Nectriaceae;g__Calcarisporium | 0.000720445 | 0.000945584 | 0.000878042 | 0.00184614 | 0.000697931 | 0.000585361 | 0 | 2.25E-05 | 0 | 0 | 9.01E-05 | 0.000247653 |
| d__Eukaryota;k__Fungi;p__Ascomycota;c__Eurotiomycetes;o__Chaetothyriales;f__Herpotrichiellaceae;g__unclassified_f__Herpotrichiellaceae | 0.000675417 | 0.000427764 | 0.000450278 | 0.003669766 | 0 | 0.000427764 | 0.00011257 | 4.50E-05 | 0.000135083 | 4.50E-05 | 0 | 2.25E-05 |
| d__Eukaryota;k__Fungi;p__Ascomycota;c__Sordariomycetes;o__Hypocreales;f__Nectriaceae;g__Volutella | 0.000720445 | 0.000180111 | 0.000247653 | 0.000315195 | 0.00184614 | 0.000720445 | 0.000247653 | 0.000382736 | 0.000292681 | 0.000540334 | 0.000247653 | 0.000225139 |
| d__Eukaryota;k__Fungi;p__Ascomycota;c__Sordariomycetes;o__Hypocreales;f__Nectriaceae;g__Lasionectria | 0 | 0 | 0 | 0 | 2.25E-05 | 0 | 9.01E-05 | 0.000135083 | 0.000787987 | 0.000247653 | 0.004457753 | 0.000180111 |
| d__Eukaryota;k__Fungi;p__Ascomycota;c__Eurotiomycetes;o__Eurotiales;f__Trichocomaceae;g__Sagenomella | 0.000382736 | 0.000585361 | 0.000337709 | 0.000540334 | 0.000247653 | 0.00040525 | 0.000652903 | 0.000585361 | 0.000135083 | 0.000720445 | 0.000720445 | 0.000495306 |
| d__Eukaryota;k__Fungi;p__Ascomycota;c__Sordariomycetes;o__Sordariales;f__Sordariales_fam_Incertae_sedis;g__Remersonia | 0.000202625 | 2.25E-05 | 9.01E-05 | 0.002003737 | 0.000630389 | 0.00011257 | 0.000382736 | 0.000270167 | 0.000585361 | 0.000675417 | 0.000157597 | 0.000630389 |
| d__Eukaryota;k__Fungi;p__Ascomycota;c__Sordariomycetes;o__Hypocreales;f__Cordycipitaceae;g__Beauveria | 0.001103181 | 0.000945584 | 0.000697931 | 0.000225139 | 0.000585361 | 0.000945584 | 0.000270167 | 0.000157597 | 6.75E-05 | 0.000337709 | 6.75E-05 | 4.50E-05 |
| d__Eukaryota;k__Fungi;p__Ascomycota;c__Sordariomycetes;o__Sordariales;f__Chaetomiaceae;g__Chaetomidium | 0 | 0 | 0 | 0.001013126 | 2.25E-05 | 2.25E-05 | 0.000607875 | 0.00011257 | 0.001103181 | 0.000652903 | 0.000292681 | 0.001575973 |
| d__Eukaryota;k__Fungi;p__Basidiomycota;c__Tremellomycetes;o__Filobasidiales;f__Filobasidiaceae;g__Naganishia | 0.000945584 | 0.000720445 | 0.000630389 | 0.000495306 | 0.000472792 | 4.50E-05 | 0.001125695 | 0.000135083 | 0.000472792 | 0 | 4.50E-05 | 0.000247653 |
| d__Eukaryota;k__Fungi;p__Ascomycota;c__Dothideomycetes;o__Pleosporales;f__Didymellaceae;g__Chaetasbolisia | 4.50E-05 | 4.50E-05 | 0.000135083 | 0 | 0.000225139 | 0.000472792 | 0.00103564 | 0.00132832 | 0.001485918 | 0.000315195 | 0 | 0.000180111 |
| d__Eukaryota;k__Fungi;p__Olpidiomycota;c__Olpidiomycetes;o__Olpidiales;f__Olpidiaceae;g__Olpidium | 2.25E-05 | 2.25E-05 | 0 | 0.001891168 | 0.00051782 | 0.000607875 | 0.000833014 | 0.000180111 | 0.000157597 | 0.000337709 | 0.000292681 | 0.00040525 |
| d__Eukaryota;k__Fungi;p__Ascomycota;c__Eurotiomycetes;o__Onygenales;f__Arthrodermataceae;g__Arthroderma | 0.000225139 | 0 | 0 | 0 | 0 | 0.004480267 | 0.000135083 | 2.25E-05 | 0.000360222 | 0 | 0 | 0 |
| d__Eukaryota;k__Fungi;p__Ascomycota;c__Sordariomycetes;o__Coniochaetales;f__unclassified_o__Coniochaetales;g__unclassified_o__Coniochaetales | 0 | 0 | 0 | 0.001125695 | 0.000495306 | 0.001283292 | 0.000382736 | 0.000180111 | 0.000337709 | 0.00051782 | 0.000292681 | 0.000472792 |
| d__Eukaryota;k__Fungi;p__Basidiomycota;c__Agaricomycetes;o__Auriculariales;f__unclassified_o__Auriculariales;g__unclassified_o__Auriculariales | 0.000157597 | 0.000337709 | 0.002183849 | 0.000135083 | 0.000157597 | 0 | 4.50E-05 | 0 | 0.000247653 | 0.000855528 | 0.000495306 | 0.000225139 |
| d__Eukaryota;k__Fungi;p__Ascomycota;c__Sordariomycetes;o__Hypocreales;f__Hypocreales_fam_Incertae_sedis;g__Chlamydocillium | 0.000360222 | 0.000225139 | 0.000135083 | 0.001170723 | 0.000540334 | 0.001778598 | 0.000135083 | 6.75E-05 | 4.50E-05 | 9.01E-05 | 6.75E-05 | 0.000157597 |
| d__Eukaryota;k__Fungi;p__Chytridiomycota;c__Rhizophydiomycetes;o__Rhizophydiales;f__Rhizophydiales_fam_Incertae_sedis;g__Operculomyces | 0 | 0.00092307 | 0.000495306 | 4.50E-05 | 2.25E-05 | 0.00132832 | 0.000878042 | 0.000270167 | 0.000495306 | 0 | 0.000180111 | 0 |
| d__Eukaryota;k__Fungi;p__Ascomycota;c__Sordariomycetes;o__Microascales;f__Microascaceae;g__Scopulariopsis | 0.000270167 | 0.000180111 | 2.25E-05 | 0.000180111 | 0.000225139 | 9.01E-05 | 0.001756084 | 0.000855528 | 0.000990612 | 0 | 2.25E-05 | 0 |
| d__Eukaryota;k__Fungi;p__Basidiomycota;c__Agaricomycetes;o__Sebacinales;f__Serendipitaceae;g__Serendipita | 0.000292681 | 0.000630389 | 0.000945584 | 0.000180111 | 0.000157597 | 0.001170723 | 0.000472792 | 0.000180111 | 0.000292681 | 4.50E-05 | 0.000180111 | 4.50E-05 |
| d__Eukaryota;k__Fungi;p__Ascomycota;c__Sordariomycetes;o__Myrmecridiales;f__Myrmecridiaceae;g__Myrmecridium | 0.000180111 | 2.25E-05 | 9.01E-05 | 0.000270167 | 0.000540334 | 0 | 0.000787987 | 0.000495306 | 0.001125695 | 0.000202625 | 0.000607875 | 0.000270167 |
| d__Eukaryota;k__Fungi;p__Basidiomycota;c__Microbotryomycetes;o__Microbotryomycetes_ord_Incertae_sedis;f__Chrysozymaceae;g__Slooffia | 0.000540334 | 0.000878042 | 0.001148209 | 0.000630389 | 2.25E-05 | 0.000337709 | 0.000157597 | 6.75E-05 | 0.00011257 | 0.000225139 | 0.000292681 | 0.000157597 |
| d__Eukaryota;k__Fungi;p__Basidiomycota;c__Tremellomycetes;o__Holtermanniales;f__Holtermanniales_fam_Incertae_sedis;g__Holtermanniella | 0 | 0 | 0 | 0.000135083 | 0.000968098 | 0 | 0.000562848 | 0.000765473 | 0.000472792 | 0.000742959 | 0.000562848 | 0.000315195 |
| d__Eukaryota;k__Fungi;p__Ascomycota;c__Sordariomycetes;o__Diaporthales;f__Diaporthaceae;g__Diaporthe | 0 | 0.004345183 | 0 | 0 | 0 | 0 | 2.25E-05 | 0 | 0 | 0 | 0 | 0 |
| d__Eukaryota;k__Fungi;p__Ascomycota;c__Sordariomycetes;o__Hypocreales;f__Stachybotryaceae;g__Paramyrothecium | 6.75E-05 | 2.25E-05 | 0.000180111 | 9.01E-05 | 0.000270167 | 0.000720445 | 0.000247653 | 0.00011257 | 0.000247653 | 0.000427764 | 0.00051782 | 0.001305806 |
| d__Eukaryota;k__Fungi;p__Ascomycota;c__Sordariomycetes;o__Ophiostomatales;f__Ophiostomataceae;g__unclassified_f__Ophiostomataceae | 0 | 0 | 0 | 0 | 2.25E-05 | 0 | 0.001913682 | 0.001801112 | 0.000360222 | 0 | 0 | 0 |
| d__Eukaryota;k__Fungi;p__Ascomycota;c__Dothideomycetes;o__Acrospermales;f__Acrospermales_fam_Incertae_sedis;g__Leptodiscella | 0.000135083 | 0.000360222 | 0.000315195 | 0.000427764 | 0.00011257 | 9.01E-05 | 0.00092307 | 0.00040525 | 0.000292681 | 0.000180111 | 0.000270167 | 0.000315195 |
| d__Eukaryota;k__Fungi;p__Basidiomycota;c__Tremellomycetes;o__Tremellales;f__Tremellales_fam_Incertae_sedis;g__Cuniculitrema | 0 | 0 | 0 | 0 | 0 | 0 | 0 | 0 | 0 | 0.00328703 | 0.000180111 | 0.000360222 |
| d__Eukaryota;k__Fungi;p__Basidiomycota;c__Agaricomycetes;o__Cantharellales;f__Ceratobasidiaceae;g__unclassified_f__Ceratobasidiaceae | 0 | 0 | 0.000180111 | 0.000382736 | 0 | 2.25E-05 | 0 | 0 | 0.002881779 | 0 | 0.000247653 | 4.50E-05 |
| d__Eukaryota;k__Fungi;p__Ascomycota;c__Sordariomycetes;o__Sordariales;f__Chaetomiaceae;g__Dichotomopilus | 0 | 0 | 0 | 0 | 0 | 0 | 0.003039377 | 0.000337709 | 0.000315195 | 0 | 0 | 0 |
| d__Eukaryota;k__Fungi;p__Ascomycota;c__Sordariomycetes;o__Sordariales;f__Lasiosphaeriaceae;g__Podospora | 4.50E-05 | 6.75E-05 | 0.000225139 | 0.00040525 | 0.000180111 | 0.000900556 | 0.000270167 | 0.000225139 | 0.001103181 | 2.25E-05 | 9.01E-05 | 0 |
| d__Eukaryota;k__Fungi;p__Glomeromycota;c__Glomeromycetes;o__Glomerales;f__Glomeraceae;g__Funneliformis | 0 | 0 | 0 | 0 | 0.003512169 | 0 | 0 | 0 | 0 | 0 | 0 | 0 |
| d__Eukaryota;k__Fungi;p__Basidiomycota;c__Tremellomycetes;o__Tremellales;f__Tremellaceae;g__Tremella | 0 | 0 | 0 | 0 | 0 | 0 | 0.001621001 | 0.000787987 | 0.001080667 | 0 | 0 | 0 |
| d__Eukaryota;k__Fungi;p__Ascomycota;c__Sordariomycetes;o__Glomerellales;f__Plectosphaerellaceae;g__unclassified_f__Plectosphaerellaceae | 0.000472792 | 0.000135083 | 0.0008105 | 0.000360222 | 0.000968098 | 9.01E-05 | 0 | 0.00011257 | 6.75E-05 | 9.01E-05 | 0.000135083 | 4.50E-05 |
| d__Eukaryota;k__Fungi;p__Ascomycota;c__Sordariomycetes;o__Hypocreales;f__Cordycipitaceae;g__Lecanicillium | 0.000247653 | 9.01E-05 | 0.000360222 | 0.000292681 | 0.00040525 | 0.000540334 | 0 | 0.000135083 | 0.000337709 | 6.75E-05 | 0.000765473 | 0 |
| d__Eukaryota;k__Fungi;p__Ascomycota;c__Dothideomycetes;o__Pleosporales;f__Lentitheciaceae;g__unclassified_f__Lentitheciaceae | 0.000157597 | 0 | 0.00011257 | 6.75E-05 | 4.50E-05 | 0.000292681 | 0.001058153 | 0.000180111 | 0.000652903 | 0 | 0.000540334 | 2.25E-05 |
| d__Eukaryota;k__Fungi;p__Ascomycota;c__Dothideomycetes;o__Venturiales;f__Sympoventuriaceae;g__Ochroconis | 0.000247653 | 0.00011257 | 0.000157597 | 0.000360222 | 0.000225139 | 0.000180111 | 0.000427764 | 0.000315195 | 0.000247653 | 0.000225139 | 0.00011257 | 0.000450278 |
| d__Eukaryota;k__Fungi;p__Basidiomycota;c__Agaricomycetes;o__Agaricales;f__Stephanosporaceae;g__unclassified_f__Stephanosporaceae | 0 | 0 | 0 | 0 | 0 | 2.25E-05 | 0.00011257 | 0.0008105 | 0.002048765 | 0 | 0 | 2.25E-05 |
| d__Eukaryota;k__Fungi;p__Ascomycota;c__Dothideomycetes;o__Pleosporales;f__Phaeosphaeriaceae;g__unclassified_f__Phaeosphaeriaceae | 9.01E-05 | 0.000720445 | 0.000427764 | 0.0008105 | 0.000202625 | 0.000202625 | 4.50E-05 | 0 | 2.25E-05 | 6.75E-05 | 0.00040525 | 0 |
| d__Eukaryota;k__Fungi;p__Basidiomycota;c__Agaricomycetes;o__Polyporales;f__Ganodermataceae;g__unclassified_f__Ganodermataceae | 2.25E-05 | 0.00011257 | 0 | 0.000945584 | 0.000292681 | 0.000135083 | 0.000225139 | 0.000202625 | 0.000225139 | 0.000337709 | 0.000270167 | 0.000180111 |
| d__Eukaryota;k__Fungi;p__Ascomycota;c__Sordariomycetes;o__Microascales;f__Microascaceae;g__unclassified_f__Microascaceae | 0.00011257 | 6.75E-05 | 0 | 0.001013126 | 0.000337709 | 0.000427764 | 0.000382736 | 0.000360222 | 0.000202625 | 4.50E-05 | 0 | 0 |
| d__Eukaryota;k__Fungi;p__Ascomycota;c__Sordariomycetes;o__Trichosphaeriales;f__Trichosphaeriaceae;g__Nigrospora | 0.000157597 | 6.75E-05 | 0.001373348 | 0 | 0.000315195 | 0.000607875 | 2.25E-05 | 0.000202625 | 4.50E-05 | 0 | 6.75E-05 | 6.75E-05 |
| d__Eukaryota;k__Fungi;p__Ascomycota;c__Sordariomycetes;o__Sordariales;f__Chaetomiaceae;g__Thielavia | 0.000675417 | 0.000180111 | 0.000157597 | 0.000225139 | 4.50E-05 | 0.00011257 | 0.000225139 | 0.000315195 | 9.01E-05 | 0.000562848 | 0.000247653 | 2.25E-05 |
| d__Eukaryota;k__Fungi;p__Ascomycota;c__Dothideomycetes;o__Pleosporales;f__Melanommataceae;g__Pleotrichocladium | 0.000360222 | 0.000225139 | 0.000337709 | 6.75E-05 | 0 | 0.000360222 | 0.000540334 | 0.000292681 | 0.000585361 | 0 | 6.75E-05 | 2.25E-05 |
| d__Eukaryota;k__Fungi;p__Ascomycota;c__Sordariomycetes;o__Sordariales;f__Lasiosphaeriaceae;g__Apiosordaria | 0.000427764 | 4.50E-05 | 0 | 0.000540334 | 0.000472792 | 0 | 0.000247653 | 0.00011257 | 0.00011257 | 0.000382736 | 0.000157597 | 0.000337709 |
| d__Eukaryota;k__Fungi;p__Basidiomycota;c__Agaricomycetes;o__Thelephorales;f__Thelephoraceae;g__Tomentella | 0.000292681 | 0.000247653 | 0.000135083 | 9.01E-05 | 0.000157597 | 0.000585361 | 9.01E-05 | 0.000495306 | 0.000202625 | 0.000202625 | 0.000135083 | 0.000180111 |
| d__Eukaryota;k__Fungi;p__Basidiomycota;c__Agaricomycetes;o__Agaricales;f__unclassified_o__Agaricales;g__unclassified_o__Agaricales | 0.000382736 | 0.000180111 | 0.000180111 | 0.000450278 | 0.000315195 | 0 | 0.000247653 | 0.00051782 | 4.50E-05 | 0.00011257 | 0.000135083 | 0.000135083 |
| d__Eukaryota;k__Fungi;p__Basidiomycota;c__Agaricomycetes;o__unclassified_c__Agaricomycetes;f__unclassified_c__Agaricomycetes;g__unclassified_c__Agaricomycetes | 0.000157597 | 0.000180111 | 0.000247653 | 0.001148209 | 4.50E-05 | 0.000225139 | 0 | 9.01E-05 | 0.000292681 | 0.000247653 | 2.25E-05 | 0 |
| d__Eukaryota;k__Fungi;p__Ascomycota;c__Sordariomycetes;o__Coniochaetales;f__Coniochaetaceae;g__Lecythophora | 0 | 4.50E-05 | 0 | 0.000652903 | 0.000135083 | 0.000675417 | 0.00011257 | 4.50E-05 | 0.000135083 | 0.000247653 | 0.000225139 | 0.000360222 |
| d__Eukaryota;k__Fungi;p__Ascomycota;c__Dothideomycetes;o__Pleosporales;f__Sporormiaceae;g__Preussia | 2.25E-05 | 0.00011257 | 4.50E-05 | 0.000157597 | 0.000315195 | 0.000427764 | 0.000225139 | 6.75E-05 | 0.000180111 | 6.75E-05 | 0.000697931 | 0.000292681 |
| d__Eukaryota;k__Fungi;p__Ascomycota;c__Saccharomycetes;o__Saccharomycetales;f__Phaffomycetaceae;g__Cyberlindnera | 0.000833014 | 0.00040525 | 0.000607875 | 2.25E-05 | 2.25E-05 | 0.00011257 | 0.000315195 | 4.50E-05 | 0.000135083 | 6.75E-05 | 0 | 2.25E-05 |
| d__Eukaryota;k__Fungi;p__Ascomycota;c__Sordariomycetes;o__Sordariales;f__Chaetomiaceae;g__Mycothermus | 0 | 0 | 6.75E-05 | 0 | 0 | 0 | 0.001013126 | 0.000562848 | 0.000607875 | 0.000337709 | 0 | 0 |
| d__Eukaryota;k__Fungi;p__Ascomycota;c__Sordariomycetes;o__Hypocreales;f__Clavicipitaceae;g__Metapochonia | 0.000135083 | 9.01E-05 | 4.50E-05 | 0.000900556 | 0.000135083 | 0.000180111 | 0.000225139 | 4.50E-05 | 0.000202625 | 4.50E-05 | 0.000157597 | 0.000382736 |
| d__Eukaryota;k__Fungi;p__Ascomycota;c__Leotiomycetes;o__Helotiales;f__Leotiaceae;g__Halenospora | 0.000360222 | 0.000270167 | 0.00011257 | 0 | 0.000697931 | 2.25E-05 | 0.000180111 | 0.000450278 | 9.01E-05 | 0.000157597 | 6.75E-05 | 0.000135083 |
| d__Eukaryota;k__Fungi;p__Ascomycota;c__Sordariomycetes;o__Hypocreales;f__Hypocreales_fam_Incertae_sedis;g__Stilbella | 0.001013126 | 0.000157597 | 4.50E-05 | 0.000157597 | 0.000675417 | 9.01E-05 | 4.50E-05 | 0 | 0.000247653 | 0 | 2.25E-05 | 2.25E-05 |
| d__Eukaryota;k__Fungi;p__Ascomycota;c__Sordariomycetes;o__Sordariales;f__Chaetomiaceae;g__Zopfiella | 0 | 0.000675417 | 4.50E-05 | 0.000630389 | 0.00011257 | 6.75E-05 | 0.000135083 | 9.01E-05 | 0.000225139 | 0 | 0.00040525 | 0 |
| d__Eukaryota;k__Fungi;p__Ascomycota;c__Leotiomycetes;o__Helotiales;f__Helotiaceae;g__Mycofalcella | 0.000607875 | 0.000270167 | 2.25E-05 | 0.00092307 | 0.000450278 | 6.75E-05 | 0 | 0 | 0 | 0 | 0 | 0 |
| d__Eukaryota;k__Fungi;p__Ascomycota;c__Sordariomycetes;o__Boliniales;f__Boliniales_fam_Incertae_sedis;g__Paramicrothyrium | 0.000270167 | 0.000360222 | 0.00011257 | 9.01E-05 | 0.000292681 | 0.000878042 | 2.25E-05 | 0.00011257 | 4.50E-05 | 6.75E-05 | 4.50E-05 | 4.50E-05 |
| d__Eukaryota;k__Fungi;p__Ascomycota;c__Sordariomycetes;o__Microascales;f__Microascaceae;g__Kernia | 0.000247653 | 2.25E-05 | 2.25E-05 | 0.00051782 | 0.000135083 | 0 | 0.00040525 | 0.00011257 | 0.000202625 | 0.000157597 | 0.000337709 | 0.000157597 |
| d__Eukaryota;k__Fungi;p__Basidiomycota;c__Agaricomycetes;o__Russulales;f__Peniophoraceae;g__Peniophora | 2.25E-05 | 0.00011257 | 0 | 0.000742959 | 0.000360222 | 0.000652903 | 4.50E-05 | 0.00011257 | 2.25E-05 | 2.25E-05 | 0.00011257 | 0.00011257 |
| d__Eukaryota;k__Fungi;p__Basidiomycota;c__Agaricomycetes;o__Agaricales;f__Psathyrellaceae;g__Hormographiella | 0 | 0 | 0 | 0 | 0 | 0 | 0.001170723 | 0.000315195 | 0.000765473 | 0 | 6.75E-05 | 0 |
| d__Eukaryota;k__Fungi;p__Ascomycota;c__Pezizomycetes;o__Pezizales;f__Ascobolaceae;g__Ascobolus | 0.000247653 | 0.00092307 | 0.000247653 | 0.000540334 | 0.000225139 | 6.75E-05 | 4.50E-05 | 0 | 0 | 0 | 0 | 0 |
| d__Eukaryota;k__Fungi;p__Ascomycota;c__Sordariomycetes;o__Hypocreales;f__Cordycipitaceae;g__Isaria | 0 | 0 | 2.25E-05 | 0 | 0 | 0 | 0.000968098 | 0.00040525 | 0.000878042 | 0 | 0 | 0 |
| d__Eukaryota;k__Fungi;p__Ascomycota;c__Sordariomycetes;o__Hypocreales;f__Nectriaceae;g__Thelonectria | 0.000135083 | 0.000247653 | 0.000742959 | 0.000765473 | 0.000157597 | 0 | 9.01E-05 | 4.50E-05 | 6.75E-05 | 0 | 0 | 0 |
| d__Eukaryota;k__Fungi;p__Ascomycota;c__Sordariomycetes;o__Hypocreales;f__Nectriaceae;g__Rectifusarium | 0 | 0 | 0 | 0.000225139 | 6.75E-05 | 0 | 0.000787987 | 0.000157597 | 0.000675417 | 6.75E-05 | 6.75E-05 | 0.000202625 |
| d__Eukaryota;k__Fungi;p__Ascomycota;c__Orbiliomycetes;o__Orbiliales;f__Orbiliaceae;g__Arthrobotrys | 2.25E-05 | 6.75E-05 | 9.01E-05 | 0 | 0 | 0.00011257 | 0.00011257 | 2.25E-05 | 0.000157597 | 0.000360222 | 0.000540334 | 0.000742959 |
| d__Eukaryota;k__Fungi;p__Ascomycota;c__Sordariomycetes;o__Glomerellales;f__Plectosphaerellaceae;g__Lectera | 0 | 0 | 0 | 4.50E-05 | 0 | 0 | 0.000427764 | 0.000337709 | 0.001373348 | 0 | 2.25E-05 | 0 |
| d__Eukaryota;k__Fungi;p__Basidiomycota;c__Agaricomycetes;o__Agaricales;f__Tricholomataceae;g__Calyptella | 0 | 0.002138821 | 0 | 0 | 0 | 0 | 0 | 0 | 0 | 0 | 0 | 0 |
| d__Eukaryota;k__Fungi;p__Ascomycota;c__Leotiomycetes;o__Helotiales;f__Vibrisseaceae;g__Phialocephala | 0 | 0 | 0 | 0 | 0.000135083 | 2.25E-05 | 0.00040525 | 0.000562848 | 0.000607875 | 6.75E-05 | 0.000247653 | 9.01E-05 |
| d__Eukaryota;k__Fungi;p__Olpidiomycota;c__Olpidiomycetes;o__Olpidiales;f__Olpidiaceae;g__unclassified_f__Olpidiaceae | 0 | 0 | 0 | 2.25E-05 | 0.000180111 | 9.01E-05 | 0 | 0.000630389 | 0.000472792 | 6.75E-05 | 0.000382736 | 0.000225139 |
| d__Eukaryota;k__Fungi;p__Zoopagomycota;c__Zoopagomycetes;o__Zoopagales;f__unclassified_o__Zoopagales;g__unclassified_o__Zoopagales | 6.75E-05 | 0.000135083 | 4.50E-05 | 0.000765473 | 0.000427764 | 0.000315195 | 0 | 0 | 4.50E-05 | 0 | 0.00011257 | 0.000157597 |
| d__Eukaryota;k__Fungi;p__Ascomycota;c__Dothideomycetes;o__Pleosporales;f__Didymellaceae;g__unclassified_f__Didymellaceae | 0 | 0.000878042 | 0 | 0.00051782 | 0.000315195 | 0.000180111 | 4.50E-05 | 0 | 4.50E-05 | 0 | 0 | 0 |
| d__Eukaryota;k__Fungi;p__Basidiomycota;c__Tremellomycetes;o__Tremellales;f__Rhynchogastremataceae;g__Papiliotrema | 0 | 0 | 0 | 0 | 0.001215751 | 4.50E-05 | 0.000135083 | 0.000202625 | 2.25E-05 | 0 | 9.01E-05 | 0.000270167 |
| d__Eukaryota;k__Fungi;p__Basidiomycota;c__Agaricomycetes;o__Agaricales;f__Entolomataceae;g__Entoloma | 0.000180111 | 0 | 9.01E-05 | 0.000247653 | 2.25E-05 | 0.000225139 | 0.000585361 | 0.000247653 | 0.000292681 | 0 | 4.50E-05 | 2.25E-05 |
| d__Eukaryota;k__Fungi;p__Basidiomycota;c__Agaricomycetes;o__Trechisporales;f__Hydnodontaceae;g__Trechispora | 9.01E-05 | 0 | 0 | 0.000540334 | 0.000135083 | 0.000495306 | 6.75E-05 | 0.00011257 | 9.01E-05 | 0.000247653 | 4.50E-05 | 9.01E-05 |
| d__Eukaryota;k__Fungi;p__Ascomycota;c__Leotiomycetes;o__Helotiales;f__Helotiales_fam_Incertae_sedis;g__Chalara | 0 | 6.75E-05 | 0.000202625 | 0 | 0 | 6.75E-05 | 2.25E-05 | 0.000833014 | 6.75E-05 | 0.000315195 | 9.01E-05 | 0.000180111 |
| d__Eukaryota;k__Fungi;p__Ascomycota;c__Leotiomycetes;o__Helotiales;f__Helotiaceae;g__Glarea | 0.000157597 | 0.000562848 | 0.000382736 | 0 | 0 | 0 | 0.000225139 | 0.000225139 | 0.000135083 | 0 | 0.000135083 | 0 |
| d__Eukaryota;k__Fungi;p__Basidiomycota;c__Agaricomycetes;o__Auriculariales;f__Exidiaceae;g__Exidia | 0 | 0 | 2.25E-05 | 0.000855528 | 0.000382736 | 0.000472792 | 4.50E-05 | 2.25E-05 | 0 | 0 | 0 | 0 |
| d__Eukaryota;k__Fungi;p__Ascomycota;c__Eurotiomycetes;o__Onygenales;f__Onygenaceae;g__Aphanoascus | 0 | 0 | 6.75E-05 | 0.000360222 | 0.000180111 | 0.00040525 | 0.000202625 | 0.000202625 | 0.000180111 | 0.000135083 | 0 | 6.75E-05 |
| d__Eukaryota;k__Fungi;p__Ascomycota;c__Dothideomycetes;o__Pleosporales;f__Torulaceae;g__Torula | 4.50E-05 | 2.25E-05 | 4.50E-05 | 4.50E-05 | 0.000225139 | 0.000382736 | 0.000247653 | 6.75E-05 | 0.00011257 | 4.50E-05 | 2.25E-05 | 0.000495306 |
| d__Eukaryota;k__Fungi;p__Ascomycota;c__Sordariomycetes;o__Chaetosphaeriales;f__Chaetosphaeriaceae;g__Chloridium | 0 | 0 | 0 | 0 | 0 | 0 | 0.000157597 | 0.00011257 | 9.01E-05 | 0.001103181 | 0.000180111 | 9.01E-05 |
| d__Eukaryota;k__Fungi;p__Ascomycota;c__Dothideomycetes;o__Pleosporales;f__Arthopyreniaceae;g__unclassified_f__Arthopyreniaceae | 0 | 0 | 0 | 0 | 0.000157597 | 0 | 0.000337709 | 0.000202625 | 0.000833014 | 6.75E-05 | 6.75E-05 | 0 |
| d__Eukaryota;k__Fungi;p__Ascomycota;c__Sordariomycetes;o__Hypocreales;f__Ophiocordycipitaceae;g__Hirsutella | 0 | 0.001508431 | 4.50E-05 | 0 | 0 | 0 | 0 | 4.50E-05 | 0 | 0 | 2.25E-05 | 0 |
| d__Eukaryota;k__Fungi;p__Basidiomycota;c__Agaricomycetes;o__Agaricales;f__Psathyrellaceae;g__Psathyrella | 2.25E-05 | 4.50E-05 | 0.000202625 | 0.000495306 | 0.000180111 | 0.000202625 | 0.000180111 | 0 | 4.50E-05 | 0 | 0.000157597 | 6.75E-05 |
| d__Eukaryota;k__Fungi;p__Ascomycota;c__Dothideomycetes;o__Venturiales;f__Sympoventuriaceae;g__Scolecobasidium | 0.000337709 | 0.00011257 | 0.000157597 | 0 | 0.00011257 | 0.000495306 | 9.01E-05 | 0.00011257 | 0.00011257 | 0 | 6.75E-05 | 0 |
| d__Eukaryota;k__Fungi;p__Basidiomycota;c__Agaricomycetes;o__Polyporales;f__Meruliaceae;g__Bjerkandera | 0 | 4.50E-05 | 0 | 0.001080667 | 2.25E-05 | 0.000315195 | 2.25E-05 | 0 | 0 | 6.75E-05 | 0 | 0 |
| d__Eukaryota;k__Fungi;p__Ascomycota;c__Sordariomycetes;o__Hypocreales;f__Tilachlidiaceae;g__Tilachlidium | 0.000180111 | 0.000337709 | 0.000270167 | 0 | 0.000292681 | 0.000360222 | 0 | 4.50E-05 | 0 | 2.25E-05 | 2.25E-05 | 2.25E-05 |
| d__Eukaryota;k__Fungi;p__Ascomycota;c__Dothideomycetes;o__Pleosporales;f__Torulaceae;g__Dendryphion | 0.00011257 | 0.000180111 | 9.01E-05 | 0 | 0 | 0.000180111 | 0.000180111 | 0.000135083 | 0.000202625 | 9.01E-05 | 0.000180111 | 0.000180111 |
| d__Eukaryota;k__Fungi;p__Ascomycota;c__Leotiomycetes;o__Helotiales;f__Leotiaceae;g__Gorgomyces | 0.000315195 | 0.000202625 | 0.000675417 | 0 | 0 | 0 | 0.000135083 | 2.25E-05 | 0.000180111 | 0 | 0 | 0 |
| d__Eukaryota;k__Fungi;p__Basidiomycota;c__Agaricomycetes;o__Russulales;f__Peniophoraceae;g__Subulicystidium | 0 | 0 | 0 | 0.000787987 | 0 | 6.75E-05 | 0.000292681 | 0 | 0.000292681 | 0 | 0 | 6.75E-05 |
| d__Eukaryota;k__Fungi;p__Ascomycota;c__Eurotiomycetes;o__Onygenales;f__Gymnoascaceae;g__Leucothecium | 9.01E-05 | 0 | 0.00011257 | 0.000225139 | 0.000135083 | 0.000360222 | 0.00011257 | 0.000135083 | 0.000337709 | 0 | 0 | 0 |
| d__Eukaryota;k__Fungi;p__Ascomycota;c__Dothideomycetes;o__Pleosporales;f__Leptosphaeriaceae;g__Neophaeosphaeria | 0.000202625 | 9.01E-05 | 6.75E-05 | 0 | 0 | 0 | 0.000337709 | 0.000472792 | 0.000292681 | 0 | 2.25E-05 | 0 |
| d__Eukaryota;k__Fungi;p__Basidiomycota;c__Agaricomycetes;o__Agaricales;f__Schizophyllaceae;g__Schizophyllum | 0 | 0 | 9.01E-05 | 0.00092307 | 0 | 0.000135083 | 0.00011257 | 4.50E-05 | 9.01E-05 | 0 | 4.50E-05 | 4.50E-05 |
| d__Eukaryota;k__Fungi;p__Rozellomycota;c__Rozellomycotina_cls_Incertae_sedis;o__Rozellomycotina_ord_Incertae_sedis;f__Rozellomycotina_fam_Incertae_sedis;g__Paramicrosporidium | 0 | 0 | 0 | 0 | 6.75E-05 | 0 | 0.00051782 | 0.00040525 | 0.000225139 | 0 | 0.000247653 | 0 |
| d__Eukaryota;k__Fungi;p__Basidiomycota;c__Agaricomycetes;o__Agaricales;f__Bolbitiaceae;g__Conocybe | 6.75E-05 | 0.000337709 | 4.50E-05 | 0.000337709 | 0.000225139 | 0.000202625 | 0 | 0.000135083 | 2.25E-05 | 0 | 2.25E-05 | 0 |
| d__Eukaryota;k__Fungi;p__Ascomycota;c__Leotiomycetes;o__Helotiales;f__Helotiaceae;g__Hymenoscyphus | 0 | 4.50E-05 | 0.000135083 | 0.000180111 | 0.000157597 | 2.25E-05 | 0.000270167 | 0.000450278 | 0.00011257 | 0 | 0 | 0 |
| d__Eukaryota;k__Fungi;p__Chytridiomycota;c__Rhizophydiomycetes;o__Rhizophydiales;f__unclassified_o__Rhizophydiales;g__unclassified_o__Rhizophydiales | 0.000652903 | 0.00051782 | 0.00011257 | 0 | 0 | 0 | 2.25E-05 | 6.75E-05 | 0 | 0 | 0 | 0 |
| d__Eukaryota;k__Fungi;p__Ascomycota;c__Sordariomycetes;o__Xylariales;f__unclassified_o__Xylariales;g__unclassified_o__Xylariales | 9.01E-05 | 0.000135083 | 2.25E-05 | 6.75E-05 | 0.000135083 | 0.00011257 | 0.000247653 | 0.000225139 | 0.000225139 | 0 | 2.25E-05 | 6.75E-05 |
| d__Eukaryota;k__Fungi;p__Ascomycota;c__Dothideomycetes;o__Pleosporales;f__Pleosporaceae;g__Alternaria | 0.000247653 | 0.000225139 | 0.00011257 | 0.000427764 | 0 | 0 | 2.25E-05 | 2.25E-05 | 4.50E-05 | 0.000202625 | 4.50E-05 | 0 |
| d__Eukaryota;k__Fungi;p__Ascomycota;c__Sordariomycetes;o__Hypocreales;f__Ophiocordycipitaceae;g__Tolypocladium | 0.001013126 | 6.75E-05 | 6.75E-05 | 0 | 0 | 9.01E-05 | 4.50E-05 | 4.50E-05 | 0 | 0 | 0 | 0 |
| d__Eukaryota;k__Fungi;p__Ascomycota;c__Sordariomycetes;o__Microascales;f__Microascaceae;g__Scedosporium | 0 | 0 | 6.75E-05 | 0.000360222 | 4.50E-05 | 0.000180111 | 0.000135083 | 4.50E-05 | 2.25E-05 | 0.000180111 | 0.000247653 | 2.25E-05 |
| d__Eukaryota;k__Fungi;p__Basidiomycota;c__Agaricomycetes;o__Auriculariales;f__Exidiaceae;g__Heterochaete | 0 | 0 | 2.25E-05 | 0.000450278 | 0.000157597 | 0 | 0.000135083 | 0 | 4.50E-05 | 6.75E-05 | 0 | 0.000382736 |
| d__Eukaryota;k__Fungi;p__Basidiomycota;c__Agaricomycetes;o__Agaricales;f__Pleurotaceae;g__Nematoctonus | 9.01E-05 | 0.000495306 | 0.000675417 | 0 | 0 | 0 | 0 | 0 | 0 | 0 | 0 | 0 |
| d__Eukaryota;k__Fungi;p__Basidiomycota;c__Agaricomycetes;o__Polyporales;f__Meruliaceae;g__Phlebia | 0 | 0 | 0 | 0.000968098 | 6.75E-05 | 0.00011257 | 0 | 4.50E-05 | 4.50E-05 | 0 | 2.25E-05 | 0 |
| d__Eukaryota;k__Fungi;p__Basidiomycota;c__Agaricomycetes;o__Sebacinales;f__unclassified_o__Sebacinales;g__unclassified_o__Sebacinales | 9.01E-05 | 0.000225139 | 0.000135083 | 0 | 0 | 4.50E-05 | 0.000157597 | 2.25E-05 | 0.00011257 | 0.000202625 | 0.000135083 | 0.000135083 |
| d__Eukaryota;k__Fungi;p__Chytridiomycota;c__Rhizophlyctidomycetes;o__Rhizophlyctidales;f__Rhizophlyctidaceae;g__Rhizophlyctis | 4.50E-05 | 0.000135083 | 0 | 0 | 0 | 0 | 0.000697931 | 0.000360222 | 0 | 0 | 0 | 0 |
| d__Eukaryota;k__Fungi;p__Ascomycota;c__Sordariomycetes;o__Hypocreales;f__Tilachlidiaceae;g__Septofusidium | 0.000225139 | 0.000247653 | 0.000472792 | 0 | 0 | 6.75E-05 | 4.50E-05 | 0 | 0 | 9.01E-05 | 4.50E-05 | 0 |
| d__Eukaryota;k__Fungi;p__Basidiomycota;c__Microbotryomycetes;o__Kriegeriales;f__Kriegeriaceae;g__unclassified_f__Kriegeriaceae | 0 | 6.75E-05 | 2.25E-05 | 0 | 0.000787987 | 0.000247653 | 0 | 0 | 0 | 6.75E-05 | 0 | 0 |
| d__Eukaryota;k__Fungi;p__Basidiomycota;c__Tremellomycetes;o__Tremellales;f__Cuniculitremaceae;g__Kockovaella | 0 | 0 | 0 | 0.000675417 | 6.75E-05 | 0.000202625 | 6.75E-05 | 0 | 0.00011257 | 0 | 0 | 0 |
| d__Eukaryota;k__Fungi;p__Ascomycota;c__Sordariomycetes;o__Hypocreales;f__Niessliaceae;g__Eucasphaeria | 0.000315195 | 0 | 9.01E-05 | 0.000180111 | 0 | 0.000225139 | 0 | 2.25E-05 | 0 | 0.000157597 | 2.25E-05 | 9.01E-05 |
| d__Eukaryota;k__Fungi;p__Ascomycota;c__Sordariomycetes;o__Hypocreales;f__Stachybotryaceae;g__Stachybotrys | 0 | 0 | 0.000157597 | 0 | 0 | 9.01E-05 | 0 | 2.25E-05 | 0 | 0.00040525 | 0.000180111 | 0.000247653 |
| d__Eukaryota;k__Fungi;p__Basidiomycota;c__Microbotryomycetes;o__unclassified_c__Microbotryomycetes;f__unclassified_c__Microbotryomycetes;g__unclassified_c__Microbotryomycetes | 4.50E-05 | 0.000607875 | 6.75E-05 | 0.00011257 | 0 | 0 | 2.25E-05 | 0 | 0 | 0 | 0.00011257 | 6.75E-05 |
| d__Eukaryota;k__Fungi;p__Ascomycota;c__Sordariomycetes;o__Sordariales;f__Sordariaceae;g__unclassified_f__Sordariaceae | 0 | 0.00011257 | 0 | 9.01E-05 | 0 | 0 | 0 | 0 | 0 | 0.00011257 | 0.000675417 | 0 |
| d__Eukaryota;k__Fungi;p__Ascomycota;c__Dothideomycetes;o__Pleosporales;f__Lophiotremataceae;g__Lophiotrema | 0 | 0 | 0 | 0 | 0.000270167 | 0 | 0 | 9.01E-05 | 0 | 9.01E-05 | 0.000157597 | 0.000337709 |
| d__Eukaryota;k__Fungi;p__Ascomycota;c__Eurotiomycetes;o__Chaetothyriales;f__Herpotrichiellaceae;g__Minimelanolocus | 9.01E-05 | 0.000315195 | 9.01E-05 | 4.50E-05 | 4.50E-05 | 0.000247653 | 2.25E-05 | 2.25E-05 | 0 | 2.25E-05 | 0 | 2.25E-05 |
| d__Eukaryota;k__Fungi;p__Ascomycota;c__Leotiomycetes;o__Thelebolales;f__Thelebolaceae;g__Thelebolus | 0.000157597 | 0.000157597 | 0 | 0 | 0 | 0 | 4.50E-05 | 0 | 0 | 0.000360222 | 9.01E-05 | 0.00011257 |
| d__Eukaryota;k__Fungi;p__Ascomycota;c__Eurotiomycetes;o__Onygenales;f__Onygenales_fam_Incertae_sedis;g__Malbranchea | 0 | 0 | 0 | 0 | 0 | 0 | 0.000360222 | 4.50E-05 | 0.000382736 | 0.00011257 | 0 | 2.25E-05 |
| d__Eukaryota;k__Fungi;p__Ascomycota;c__Sordariomycetes;o__Hypocreales;f__Nectriaceae;g__Stephanonectria | 0 | 0 | 0 | 6.75E-05 | 0.000270167 | 0.00011257 | 0.000315195 | 2.25E-05 | 4.50E-05 | 0 | 2.25E-05 | 6.75E-05 |
| d__Eukaryota;k__Fungi;p__Basidiomycota;c__Agaricomycetes;o__Polyporales;f__Meruliaceae;g__Phanerochaete | 0 | 0 | 0 | 0.000607875 | 0 | 0.000157597 | 0.000135083 | 0 | 0 | 0 | 0 | 0 |
| d__Eukaryota;k__Fungi;p__Basidiomycota;c__Agaricomycetes;o__Polyporales;f__Coriolaceae;g__Trametes | 0 | 0 | 0 | 0.000742959 | 0 | 4.50E-05 | 4.50E-05 | 2.25E-05 | 0 | 0 | 0 | 4.50E-05 |
| d__Eukaryota;k__Fungi;p__Ascomycota;c__Dothideomycetes;o__Pleosporales;f__Dictyosporiaceae;g__Pseudocoleophoma | 0 | 0 | 0 | 9.01E-05 | 0.000292681 | 0 | 2.25E-05 | 0.000135083 | 0.000360222 | 0 | 0 | 0 |
| d__Eukaryota;k__Fungi;p__Ascomycota;c__Sordariomycetes;o__Sordariales;f__Chaetomiaceae;g__Melanocarpus | 0 | 2.25E-05 | 0 | 2.25E-05 | 0 | 0 | 0.000157597 | 0.000135083 | 0.000247653 | 2.25E-05 | 0.000157597 | 0.00011257 |
| d__Eukaryota;k__Fungi;p__Chytridiomycota;c__Rhizophydiomycetes;o__Rhizophydiales;f__Alphamycetaceae;g__Betamyces | 0 | 0 | 0 | 0 | 0 | 0 | 0 | 0 | 0 | 6.75E-05 | 0.000450278 | 0.000360222 |
| d__Eukaryota;k__Fungi;p__Ascomycota;c__Sordariomycetes;o__Hypocreales;f__Nectriaceae;g__Pseudocosmospora | 0 | 0.000180111 | 0.000135083 | 0 | 0.000270167 | 9.01E-05 | 6.75E-05 | 2.25E-05 | 0.00011257 | 0 | 0 | 0 |
| d__Eukaryota;k__Fungi;p__Ascomycota;c__Eurotiomycetes;o__Chaetothyriales;f__Cyphellophoraceae;g__Anthopsis | 0 | 0 | 0 | 0 | 0 | 0 | 0.000202625 | 0.000225139 | 0.000382736 | 0 | 2.25E-05 | 2.25E-05 |
| d__Eukaryota;k__Fungi;p__Ascomycota;c__Eurotiomycetes;o__unclassified_c__Eurotiomycetes;f__unclassified_c__Eurotiomycetes;g__unclassified_c__Eurotiomycetes | 0.00011257 | 0 | 0.000202625 | 0.000382736 | 0 | 0 | 2.25E-05 | 2.25E-05 | 9.01E-05 | 2.25E-05 | 0 | 0 |
| d__Eukaryota;k__Fungi;p__Ascomycota;c__Eurotiomycetes;o__Eurotiales;f__Aspergillaceae;g__Phialosimplex | 0 | 4.50E-05 | 0 | 9.01E-05 | 0.000360222 | 0 | 2.25E-05 | 6.75E-05 | 4.50E-05 | 0.00011257 | 0 | 0.00011257 |
| d__Eukaryota;k__Fungi;p__Glomeromycota;c__Glomeromycetes;o__Diversisporales;f__Diversisporales_fam_Incertae_sedis;g__Entrophospora | 6.75E-05 | 0 | 0 | 0 | 0 | 0 | 6.75E-05 | 0 | 2.25E-05 | 0 | 0.000675417 | 0 |
| d__Eukaryota;k__Fungi;p__Ascomycota;c__Sordariomycetes;o__Hypocreales;f__Clavicipitaceae;g__unclassified_f__Clavicipitaceae | 2.25E-05 | 9.01E-05 | 2.25E-05 | 0.000315195 | 0.000180111 | 0.000135083 | 0 | 0 | 4.50E-05 | 0 | 0 | 0 |
| d__Eukaryota;k__Fungi;p__Basidiomycota;c__Pucciniomycetes;o__Platygloeales;f__Eocronartiaceae;g__Eocronartium | 0.000180111 | 0.000292681 | 0.000337709 | 0 | 0 | 0 | 0 | 0 | 0 | 0 | 0 | 0 |
| d__Eukaryota;k__Fungi;p__Basidiomycota;c__Microbotryomycetes;o__Microbotryomycetes_ord_Incertae_sedis;f__Chrysozymaceae;g__Sampaiozyma | 2.25E-05 | 9.01E-05 | 0.000180111 | 0.000225139 | 0 | 0.00011257 | 0.000135083 | 0 | 0 | 4.50E-05 | 0 | 0 |
| d__Eukaryota;k__Fungi;p__Ascomycota;c__Leotiomycetes;o__Helotiales;f__Hyaloscyphaceae;g__Cistella | 0.00011257 | 2.25E-05 | 9.01E-05 | 2.25E-05 | 6.75E-05 | 0.00011257 | 6.75E-05 | 9.01E-05 | 0.000180111 | 0 | 0 | 4.50E-05 |
| d__Eukaryota;k__Fungi;p__Basidiomycota;c__Agaricomycetes;o__Agaricales;f__Cortinariaceae;g__Cortinarius | 0 | 0 | 0.000247653 | 0.000337709 | 0 | 2.25E-05 | 0 | 2.25E-05 | 0 | 2.25E-05 | 4.50E-05 | 0.00011257 |
| d__Eukaryota;k__Fungi;p__Basidiomycota;c__Agaricomycetes;o__Polyporales;f__Steccherinaceae;g__Steccherinum | 2.25E-05 | 0 | 0 | 0.000540334 | 0 | 0 | 0 | 4.50E-05 | 6.75E-05 | 0.00011257 | 0 | 0 |
| d__Eukaryota;k__Fungi;p__Basidiomycota;c__Tremellomycetes;o__unclassified_c__Tremellomycetes;f__unclassified_c__Tremellomycetes;g__unclassified_c__Tremellomycetes | 0 | 0 | 0 | 2.25E-05 | 0 | 4.50E-05 | 0.000157597 | 0.000382736 | 0.000157597 | 0 | 0 | 0 |
| d__Eukaryota;k__Fungi;p__Ascomycota;c__Orbiliomycetes;o__Orbiliales;f__Orbiliaceae;g__Retiarius | 6.75E-05 | 0 | 0 | 0.000675417 | 0 | 2.25E-05 | 0 | 0 | 0 | 0 | 0 | 0 |
| d__Eukaryota;k__Fungi;p__Ascomycota;c__Eurotiomycetes;o__Onygenales;f__unclassified_o__Onygenales;g__unclassified_o__Onygenales | 0.00011257 | 0.00011257 | 4.50E-05 | 0.000135083 | 0.000157597 | 4.50E-05 | 2.25E-05 | 0 | 0.000135083 | 0 | 0 | 0 |
| d__Eukaryota;k__Fungi;p__Ascomycota;c__Leotiomycetes;o__Helotiales;f__Dermateaceae;g__Mollisia | 0.000135083 | 0 | 0.00011257 | 0.000157597 | 0 | 0 | 0 | 4.50E-05 | 0 | 4.50E-05 | 9.01E-05 | 0.000157597 |
| d__Eukaryota;k__Fungi;p__Basidiomycota;c__Pucciniomycetes;o__Platygloeales;f__unclassified_o__Platygloeales;g__unclassified_o__Platygloeales | 4.50E-05 | 6.75E-05 | 0.000292681 | 0 | 0.000135083 | 0 | 0 | 0 | 2.25E-05 | 0.000135083 | 2.25E-05 | 2.25E-05 |
| d__Eukaryota;k__Fungi;p__Basidiomycota;c__Agaricomycetes;o__Agaricales;f__Strophariaceae;g__Deconica | 0 | 2.25E-05 | 0 | 0.000360222 | 0 | 4.50E-05 | 2.25E-05 | 6.75E-05 | 4.50E-05 | 6.75E-05 | 0 | 0.00011257 |
| d__Eukaryota;k__Fungi;p__Ascomycota;c__Sordariomycetes;o__Hypocreales;f__Hypocreaceae;g__Monocillium | 0.000247653 | 0 | 0.000202625 | 0 | 4.50E-05 | 0.000135083 | 0 | 2.25E-05 | 0 | 6.75E-05 | 0 | 0 |
| d__Eukaryota;k__Fungi;p__Ascomycota;c__Orbiliomycetes;o__Orbiliales;f__Orbiliaceae;g__Monacrosporium | 2.25E-05 | 0 | 2.25E-05 | 0 | 0 | 0 | 0.000135083 | 4.50E-05 | 0.000337709 | 0 | 0.000135083 | 2.25E-05 |
| d__Eukaryota;k__Fungi;p__Ascomycota;c__Sordariomycetes;o__Microascales;f__Microascaceae;g__Petriella | 0 | 0 | 0 | 0.000180111 | 6.75E-05 | 0 | 6.75E-05 | 0 | 6.75E-05 | 0.000315195 | 0 | 0 |
| d__Eukaryota;k__Fungi;p__Ascomycota;c__Sordariomycetes;o__Hypocreales;f__Nectriaceae;g__Xenoacremonium | 0 | 0 | 0 | 0 | 0 | 0 | 0.000450278 | 9.01E-05 | 0.000157597 | 0 | 0 | 0 |
| d__Eukaryota;k__Fungi;p__Ascomycota;c__Sordariomycetes;o__Ophiostomatales;f__Ophiostomataceae;g__Hawksworthiomyces | 0 | 0 | 0 | 0 | 0 | 0 | 0.000607875 | 6.75E-05 | 2.25E-05 | 0 | 0 | 0 |
| d__Eukaryota;k__Fungi;p__Ascomycota;c__Sordariomycetes;o__Hypocreales;f__Tilachlidiaceae;g__unclassified_f__Tilachlidiaceae | 9.01E-05 | 4.50E-05 | 0.000135083 | 0 | 0 | 0.000135083 | 2.25E-05 | 6.75E-05 | 0.000180111 | 0 | 0 | 2.25E-05 |
| d__Eukaryota;k__Fungi;p__Ascomycota;c__Eurotiomycetes;o__Verrucariales;f__Verrucariaceae;g__unclassified_f__Verrucariaceae | 4.50E-05 | 0 | 0 | 0.000585361 | 6.75E-05 | 0 | 0 | 0 | 0 | 0 | 0 | 0 |
| d__Eukaryota;k__Fungi;p__Ascomycota;c__Sordariomycetes;o__Glomerellales;f__Plectosphaerellaceae;g__Stachylidium | 4.50E-05 | 0 | 0 | 0 | 0 | 0 | 0.000315195 | 6.75E-05 | 0.000135083 | 0 | 0 | 0.000135083 |
| d__Eukaryota;k__Fungi;p__Basidiomycota;c__Wallemiomycetes;o__Wallemiales;f__Wallemiaceae;g__Wallemia | 0 | 0 | 0 | 0.000562848 | 4.50E-05 | 9.01E-05 | 0 | 0 | 0 | 0 | 0 | 0 |
| d__Eukaryota;k__Fungi;p__Basidiomycota;c__Agaricostilbomycetes;o__Agaricostilbales;f__Kondoaceae;g__Kondoa | 0 | 0 | 0 | 0.000585361 | 0.00011257 | 0 | 0 | 0 | 0 | 0 | 0 | 0 |
| d__Eukaryota;k__Fungi;p__Ascomycota;c__Sordariomycetes;o__Hypocreales;f__Stachybotryaceae;g__Striatibotrys | 0.000202625 | 0 | 2.25E-05 | 0 | 0 | 6.75E-05 | 0.000180111 | 0.00011257 | 6.75E-05 | 0 | 0 | 2.25E-05 |
| d__Eukaryota;k__Fungi;p__Chytridiomycota;c__Chytridiomycetes;o__Chytridiales;f__Chytridiaceae;g__Dendrochytridium | 4.50E-05 | 0.000225139 | 0 | 0 | 0 | 0 | 0.000270167 | 0.00011257 | 2.25E-05 | 0 | 0 | 0 |
| d__Eukaryota;k__Fungi;p__Basidiomycota;c__Agaricomycetes;o__Polyporales;f__Meruliaceae;g__Scopuloides | 0 | 2.25E-05 | 2.25E-05 | 0.000225139 | 0 | 6.75E-05 | 4.50E-05 | 0 | 0 | 0.000225139 | 0 | 4.50E-05 |
| d__Eukaryota;k__Fungi;p__Basidiobolomycota;c__Basidiobolomycetes;o__Basidiobolales;f__unclassified_o__Basidiobolales;g__unclassified_o__Basidiobolales | 0 | 0 | 0 | 0.000450278 | 4.50E-05 | 0 | 9.01E-05 | 2.25E-05 | 0 | 2.25E-05 | 0 | 2.25E-05 |
| d__Eukaryota;k__Fungi;p__Ascomycota;c__Orbiliomycetes;o__Orbiliales;f__Orbiliaceae;g__Dactylaria | 0.000180111 | 0.000382736 | 6.75E-05 | 0 | 0 | 0 | 0 | 0 | 0 | 0 | 0 | 0 |
| d__Eukaryota;k__Fungi;p__Ascomycota;c__Eurotiomycetes;o__Onygenales;f__Gymnoascaceae;g__Gymnoascus | 9.01E-05 | 0 | 0 | 0.000225139 | 0.000180111 | 6.75E-05 | 0 | 0 | 0 | 0 | 2.25E-05 | 4.50E-05 |
| d__Eukaryota;k__Fungi;p__Basidiomycota;c__Cystobasidiomycetes;o__Cystobasidiales;f__Cystobasidiaceae;g__Occultifur | 0 | 0 | 0 | 0 | 0.000135083 | 0 | 0.000315195 | 0.000157597 | 2.25E-05 | 0 | 0 | 0 |
| d__Eukaryota;k__Fungi;p__Ascomycota;c__Leotiomycetes;o__Helotiales;f__Leotiaceae;g__Neobulgaria | 2.25E-05 | 0.000450278 | 9.01E-05 | 6.75E-05 | 0 | 0 | 0 | 0 | 0 | 0 | 0 | 0 |
| d__Eukaryota;k__Fungi;p__Basidiomycota;c__Microbotryomycetes;o__Leucosporidiales;f__Leucosporidiaceae;g__Leucosporidium | 0.000225139 | 0.000135083 | 0.000135083 | 0 | 4.50E-05 | 2.25E-05 | 4.50E-05 | 2.25E-05 | 0 | 0 | 0 | 0 |
| d__Eukaryota;k__Fungi;p__Ascomycota;c__Eurotiomycetes;o__Chaetothyriales;f__Herpotrichiellaceae;g__Fonsecaea | 0 | 0 | 0 | 0 | 0 | 0 | 0.000337709 | 0.000157597 | 0.000135083 | 0 | 0 | 0 |
| d__Eukaryota;k__Fungi;p__Monoblepharomycota;c__Sanchytriomycetes;o__Sanchytriales;f__Sanchytriaceae;g__unclassified_f__Sanchytriaceae | 4.50E-05 | 0.00011257 | 6.75E-05 | 6.75E-05 | 6.75E-05 | 0 | 0 | 0 | 0 | 9.01E-05 | 6.75E-05 | 0.00011257 |
| d__Eukaryota;k__Fungi;p__Ascomycota;c__Eurotiomycetes;o__Eurotiales;f__unclassified_o__Eurotiales;g__unclassified_o__Eurotiales | 0 | 2.25E-05 | 0 | 0 | 9.01E-05 | 0 | 0.00011257 | 0 | 0.000382736 | 0 | 0 | 0 |
| d__Eukaryota;k__Fungi;p__Ascomycota;c__Sordariomycetes;o__Microascales;f__Microascales_fam_Incertae_sedis;g__Wardomyces | 2.25E-05 | 0 | 0 | 0.000225139 | 9.01E-05 | 6.75E-05 | 2.25E-05 | 0 | 2.25E-05 | 2.25E-05 | 9.01E-05 | 2.25E-05 |
| d__Eukaryota;k__Fungi;p__Basidiomycota;c__Agaricomycetes;o__Agaricales;f__Strophariaceae;g__Galerina | 0 | 0 | 0 | 0 | 0 | 0 | 0.000270167 | 9.01E-05 | 0.000135083 | 9.01E-05 | 0 | 0 |
| d__Eukaryota;k__Fungi;p__Zoopagomycota;c__Zoopagomycetes;o__Zoopagales;f__Piptocephalidaceae;g__unclassified_f__Piptocephalidaceae | 2.25E-05 | 4.50E-05 | 0 | 2.25E-05 | 0 | 0.000292681 | 0.00011257 | 2.25E-05 | 6.75E-05 | 0 | 0 | 0 |
| d__Eukaryota;k__Fungi;p__Ascomycota;c__Sordariomycetes;o__Xylariales;f__Apiosporaceae;g__Arthrinium | 0 | 0.00011257 | 0 | 0.00011257 | 0 | 0 | 0 | 0 | 0 | 0.000157597 | 9.01E-05 | 0.00011257 |
| d__Eukaryota;k__Fungi;p__Ascomycota;c__Leotiomycetes;o__Helotiales;f__Leotiaceae;g__Flagellospora | 0 | 0 | 0 | 9.01E-05 | 0 | 4.50E-05 | 4.50E-05 | 0.000202625 | 0.000180111 | 0 | 0 | 0 |
| d__Eukaryota;k__Fungi;p__Ascomycota;c__Eurotiomycetes;o__Chaetothyriales;f__unclassified_o__Chaetothyriales;g__unclassified_o__Chaetothyriales | 2.25E-05 | 0 | 0 | 0 | 0.000225139 | 0.000157597 | 0 | 6.75E-05 | 2.25E-05 | 6.75E-05 | 0 | 0 |
| d__Eukaryota;k__Fungi;p__Basidiomycota;c__Agaricomycetes;o__Thelephorales;f__Thelephoraceae;g__unclassified_f__Thelephoraceae | 0.000157597 | 0.000180111 | 0.00011257 | 0 | 0 | 6.75E-05 | 2.25E-05 | 2.25E-05 | 0 | 0 | 0 | 0 |
| d__Eukaryota;k__Fungi;p__Ascomycota;c__Eurotiomycetes;o__Chaetothyriales;f__Herpotrichiellaceae;g__Argopericonia | 0 | 0 | 0 | 0 | 0 | 0 | 0.00011257 | 9.01E-05 | 0.000360222 | 0 | 0 | 0 |
| d__Eukaryota;k__Fungi;p__Ascomycota;c__Dothideomycetes;o__Pleosporales;f__Sporormiaceae;g__Sporormiella | 0.000270167 | 0 | 9.01E-05 | 0 | 9.01E-05 | 0 | 0 | 0.00011257 | 0 | 0 | 0 | 0 |
| d__Eukaryota;k__Fungi;p__Ascomycota;c__Dothideomycetes;o__Pleosporales;f__Massarinaceae;g__Stagonospora | 0 | 0 | 0 | 0.000180111 | 6.75E-05 | 0.000202625 | 2.25E-05 | 0 | 6.75E-05 | 0 | 0 | 0 |
| d__Eukaryota;k__Fungi;p__Ascomycota;c__Sordariomycetes;o__Sordariales;f__Sordariales_fam_Incertae_sedis;g__Conlarium | 2.25E-05 | 0.000202625 | 0.000247653 | 0 | 0 | 0 | 0 | 0 | 2.25E-05 | 2.25E-05 | 0 | 2.25E-05 |
| d__Eukaryota;k__Fungi;p__Basidiomycota;c__Agaricomycetes;o__Agaricales;f__Clavariaceae;g__Clavulinopsis | 6.75E-05 | 2.25E-05 | 0 | 0 | 0 | 0 | 2.25E-05 | 0.000427764 | 0 | 0 | 0 | 0 |
| d__Eukaryota;k__Fungi;p__Mortierellomycota;c__unclassified_p__Mortierellomycota;o__unclassified_p__Mortierellomycota;f__unclassified_p__Mortierellomycota;g__unclassified_p__Mortierellomycota | 4.50E-05 | 0.000157597 | 0.000157597 | 0 | 0 | 0 | 0 | 0.00011257 | 6.75E-05 | 0 | 0 | 0 |
| d__Eukaryota;k__Fungi;p__Basidiomycota;c__Microbotryomycetes;o__Sporidiobolales;f__Sporidiobolaceae;g__Rhodotorula | 0 | 9.01E-05 | 0.000247653 | 0 | 0 | 0 | 4.50E-05 | 4.50E-05 | 9.01E-05 | 0 | 0 | 0 |
| d__Eukaryota;k__Fungi;p__Ascomycota;c__Dothideomycetes;o__Pleosporales;f__Sporormiaceae;g__Westerdykella | 0 | 4.50E-05 | 0 | 2.25E-05 | 0 | 0 | 0 | 0 | 0 | 0.000180111 | 0.000135083 | 0.000135083 |
| d__Eukaryota;k__Fungi;p__Ascomycota;c__Pezizomycetes;o__Pezizales;f__Pyronemataceae;g__Trichophaeopsis | 0 | 0 | 0 | 0.000495306 | 0 | 0 | 0 | 2.25E-05 | 0 | 0 | 0 | 0 |
| d__Eukaryota;k__Fungi;p__Basidiomycota;c__Tremellomycetes;o__Cystofilobasidiales;f__unclassified_o__Cystofilobasidiales;g__unclassified_o__Cystofilobasidiales | 0.000180111 | 0 | 0.000157597 | 0 | 0 | 0 | 4.50E-05 | 6.75E-05 | 0 | 0 | 0 | 4.50E-05 |
| d__Eukaryota;k__Fungi;p__Basidiomycota;c__Agaricomycetes;o__Agaricales;f__Hymenogastraceae;g__Naucoria | 2.25E-05 | 4.50E-05 | 4.50E-05 | 0 | 0 | 0.000292681 | 0 | 0 | 0 | 0 | 9.01E-05 | 0 |
| d__Eukaryota;k__Fungi;p__Ascomycota;c__Sordariomycetes;o__Sordariales;f__Cephalothecaceae;g__Phialemonium | 0.000157597 | 9.01E-05 | 0.000135083 | 6.75E-05 | 4.50E-05 | 0 | 0 | 0 | 0 | 0 | 0 | 0 |
| d__Eukaryota;k__Fungi;p__Ascomycota;c__Sordariomycetes;o__Sordariales;f__Chaetomiaceae;g__Collariella | 6.75E-05 | 6.75E-05 | 0.000247653 | 6.75E-05 | 0 | 0 | 0 | 2.25E-05 | 0 | 0 | 0 | 0 |
| d__Eukaryota;k__Fungi;p__Ascomycota;c__Pezizomycotina_cls_Incertae_sedis;o__Pezizomycotina_ord_Incertae_sedis;f__Pezizomycotina_fam_Incertae_sedis;g__Hormiactis | 6.75E-05 | 4.50E-05 | 0.000247653 | 6.75E-05 | 0 | 0 | 2.25E-05 | 0 | 0 | 0 | 0 | 2.25E-05 |
| d__Eukaryota;k__Fungi;p__Ascomycota;c__Eurotiomycetes;o__Chaetothyriales;f__Herpotrichiellaceae;g__Rhinocladiella | 0 | 0 | 0 | 0 | 0 | 0 | 0.000315195 | 4.50E-05 | 0.00011257 | 0 | 0 | 0 |
| d__Eukaryota;k__Fungi;p__Ascomycota;c__Sordariomycetes;o__Hypocreales;f__Cordycipitaceae;g__Cordyceps | 0 | 0.000157597 | 4.50E-05 | 0 | 0.000135083 | 0.000135083 | 0 | 0 | 0 | 0 | 0 | 0 |
| d__Eukaryota;k__Fungi;p__Basidiomycota;c__Microbotryomycetes;o__Microbotryales;f__Ustilentylomataceae;g__Microbotryozyma | 0.000135083 | 0.000157597 | 0.00011257 | 0 | 0 | 0 | 0 | 4.50E-05 | 2.25E-05 | 0 | 0 | 0 |
| d__Eukaryota;k__Fungi;p__Ascomycota;c__Dothideomycetes;o__Capnodiales;f__Mycosphaerellaceae;g__Ramularia | 0 | 0.00011257 | 2.25E-05 | 0.000202625 | 0 | 0.00011257 | 0 | 0 | 0 | 0 | 2.25E-05 | 0 |
| d__Eukaryota;k__Fungi;p__Ascomycota;c__Sordariomycetes;o__Hypocreales;f__Nectriaceae;g__Atractium | 2.25E-05 | 0.00011257 | 0 | 0 | 0 | 0 | 0.000247653 | 6.75E-05 | 0 | 0 | 0 | 0 |
| d__Eukaryota;k__Fungi;p__Ascomycota;c__Sordariomycetes;o__Hypocreales;f__Bionectriaceae;g__Gliomastix | 9.01E-05 | 0 | 0.000270167 | 0 | 0 | 0 | 0 | 0 | 0 | 2.25E-05 | 4.50E-05 | 2.25E-05 |
| d__Eukaryota;k__Fungi;p__Ascomycota;c__Sordariomycetes;o__Xylariales;f__Microdochiaceae;g__Microdochium | 6.75E-05 | 0 | 0 | 0 | 2.25E-05 | 0 | 2.25E-05 | 0 | 6.75E-05 | 2.25E-05 | 0.000135083 | 0.00011257 |
| d__Eukaryota;k__Fungi;p__Ascomycota;c__Pezizomycetes;o__Pezizales;f__Ascodesmidaceae;g__Eleutherascus | 2.25E-05 | 0.000135083 | 0.00011257 | 0 | 4.50E-05 | 0 | 0 | 0 | 6.75E-05 | 0 | 6.75E-05 | 0 |
| d__Eukaryota;k__Fungi;p__Ascomycota;c__Dothideomycetes;o__Capnodiales;f__Mycosphaerellaceae;g__Cercospora | 0.000180111 | 0 | 2.25E-05 | 0 | 0.000225139 | 0 | 0 | 0 | 0 | 0 | 0 | 0 |
| d__Eukaryota;k__Fungi;p__Basidiomycota;c__Agaricomycetes;o__Agaricales;f__Agaricaceae;g__Coprinus | 0 | 0 | 0 | 0 | 0 | 0 | 0.000247653 | 4.50E-05 | 0.000135083 | 0 | 0 | 0 |
| d__Eukaryota;k__Fungi;p__Ascomycota;c__Sordariomycetes;o__Microascales;f__Microascaceae;g__Yunnania | 0 | 0 | 0 | 0 | 0.000292681 | 6.75E-05 | 0 | 0 | 0 | 4.50E-05 | 0 | 2.25E-05 |
| d__Eukaryota;k__Fungi;p__Basidiomycota;c__Agaricomycetes;o__Cantharellales;f__Cantharellales_fam_Incertae_sedis;g__Burgoa | 0 | 0 | 0 | 0 | 0 | 0 | 0.000315195 | 0 | 0.00011257 | 0 | 0 | 0 |
| d__Eukaryota;k__Fungi;p__Basidiomycota;c__Cystobasidiomycetes;o__unclassified_c__Cystobasidiomycetes;f__unclassified_c__Cystobasidiomycetes;g__unclassified_c__Cystobasidiomycetes | 0 | 0 | 0 | 2.25E-05 | 0 | 9.01E-05 | 0.000202625 | 2.25E-05 | 9.01E-05 | 0 | 0 | 0 |
| d__Eukaryota;k__Fungi;p__Ascomycota;c__Sordariomycetes;o__Sordariales;f__Chaetomiaceae;g__Crassicarpon | 0 | 0 | 0 | 0 | 0 | 0 | 4.50E-05 | 0.000337709 | 0 | 4.50E-05 | 0 | 0 |
| d__Eukaryota;k__Fungi;p__Ascomycota;c__Eurotiomycetes;o__Chaetothyriales;f__Herpotrichiellaceae;g__Capronia | 0 | 0 | 0 | 0 | 0 | 0 | 0.00040525 | 0 | 2.25E-05 | 0 | 0 | 0 |
| d__Eukaryota;k__Fungi;p__Ascomycota;c__Xylonomycetes;o__GS34;f__unclassified_o__GS34;g__unclassified_o__GS34 | 0 | 0 | 0 | 0 | 0 | 0 | 0.000247653 | 6.75E-05 | 9.01E-05 | 0 | 0 | 0 |
| d__Eukaryota;k__Fungi;p__Basidiomycota;c__Agaricomycetes;o__Boletales;f__Paxillaceae;g__Paxillus | 2.25E-05 | 0 | 0 | 0 | 0 | 0 | 0 | 4.50E-05 | 0 | 2.25E-05 | 0.000135083 | 0.000180111 |
| d__Eukaryota;k__Fungi;p__Basidiomycota;c__Agaricomycetes;o__Agaricales;f__Pluteaceae;g__Pluteus | 0 | 0 | 0 | 0 | 0 | 0 | 0.000315195 | 4.50E-05 | 2.25E-05 | 0 | 0 | 2.25E-05 |
| d__Eukaryota;k__Fungi;p__Ascomycota;c__Orbiliomycetes;o__Orbiliales;f__Orbiliales_fam_Incertae_sedis;g__Vermispora | 0 | 6.75E-05 | 0 | 0.000157597 | 0 | 0 | 0 | 4.50E-05 | 0.000135083 | 0 | 0 | 0 |
| d__Eukaryota;k__Fungi;p__Basidiomycota;c__Agaricomycetes;o__Auriculariales;f__Auriculariales_fam_Incertae_sedis;g__Oliveonia | 4.50E-05 | 2.25E-05 | 4.50E-05 | 0.000180111 | 0 | 0.00011257 | 0 | 0 | 0 | 0 | 0 | 0 |
| d__Eukaryota;k__Fungi;p__Basidiomycota;c__Agaricomycetes;o__Agaricales;f__Lycoperdaceae;g__Lycoperdon | 0 | 4.50E-05 | 0 | 0 | 0.000270167 | 0 | 0 | 0 | 0 | 0 | 2.25E-05 | 6.75E-05 |
| d__Eukaryota;k__Fungi;p__Ascomycota;c__Saccharomycetes;o__Saccharomycetales;f__Saccharomycetaceae;g__Vanderwaltozyma | 2.25E-05 | 4.50E-05 | 0.000337709 | 0 | 0 | 0 | 0 | 0 | 0 | 0 | 0 | 0 |
| d__Eukaryota;k__Fungi;p__Ascomycota;c__Sordariomycetes;o__Hypocreales;f__Hypocreales_fam_Incertae_sedis;g__unclassified_f__Hypocreales_fam_Incertae_sedis | 4.50E-05 | 4.50E-05 | 0 | 0 | 4.50E-05 | 0 | 4.50E-05 | 4.50E-05 | 0.00011257 | 0 | 2.25E-05 | 4.50E-05 |
| d__Eukaryota;k__Fungi;p__Ascomycota;c__Eurotiomycetes;o__Chaetothyriales;f__Trichomeriaceae;g__Bradymyces | 0 | 0 | 0 | 0.00011257 | 0.000292681 | 0 | 0 | 0 | 0 | 0 | 0 | 0 |
| d__Eukaryota;k__Fungi;p__Ascomycota;c__Leotiomycetes;o__Helotiales;f__Leotiaceae;g__Pezoloma | 0 | 2.25E-05 | 2.25E-05 | 0 | 0 | 4.50E-05 | 4.50E-05 | 0.000180111 | 6.75E-05 | 0 | 0 | 0 |
| d__Eukaryota;k__Fungi;p__Basidiomycota;c__Agaricomycetes;o__Polyporales;f__Meruliaceae;g__Porostereum | 0 | 0 | 9.01E-05 | 0 | 9.01E-05 | 0.000180111 | 0 | 2.25E-05 | 0 | 0 | 0 | 0 |
| d__Eukaryota;k__Fungi;p__Basidiomycota;c__Agaricomycetes;o__Cantharellales;f__Cantharellales_fam_Incertae_sedis;g__Minimedusa | 0 | 0 | 0 | 0 | 0 | 0 | 0.000382736 | 0 | 0 | 0 | 0 | 0 |
| d__Eukaryota;k__Fungi;p__Ascomycota;c__Sordariomycetes;o__Conioscyphales;f__Conioscyphaceae;g__Conioscypha | 0 | 0 | 0 | 0 | 0 | 0 | 9.01E-05 | 0 | 0.000292681 | 0 | 0 | 0 |
| d__Eukaryota;k__Fungi;p__Ascomycota;c__Sordariomycetes;o__Hypocreales;f__Nectriaceae;g__Sarcopodium | 0 | 0 | 0 | 2.25E-05 | 0.000292681 | 6.75E-05 | 0 | 0 | 0 | 0 | 0 | 0 |
| d__Eukaryota;k__Fungi;p__Ascomycota;c__Pezizomycetes;o__Pezizales;f__Morchellaceae;g__Morchella | 0 | 0 | 0 | 0 | 0 | 2.25E-05 | 0 | 0 | 0 | 0 | 0.000360222 | 0 |
| d__Eukaryota;k__Fungi;p__Ascomycota;c__Dothideomycetes;o__Pleosporales;f__Pleosporaceae;g__unclassified_f__Pleosporaceae | 0 | 0 | 0.000360222 | 0 | 0 | 0 | 0 | 0 | 0 | 0 | 0 | 2.25E-05 |
| d__Eukaryota;k__Fungi;p__Ascomycota;c__Sordariomycetes;o__Hypocreales;f__Stachybotryaceae;g__Alfaria | 0 | 0 | 0 | 0 | 0 | 4.50E-05 | 0.00011257 | 2.25E-05 | 0.000202625 | 0 | 0 | 0 |
| d__Eukaryota;k__Fungi;p__Ascomycota;c__Sordariomycetes;o__Phomatosporales;f__Phomatosporaceae;g__Phomatospora | 0 | 4.50E-05 | 0 | 0 | 0 | 0 | 0 | 0 | 2.25E-05 | 9.01E-05 | 4.50E-05 | 0.000157597 |
| d__Eukaryota;k__Fungi;p__Ascomycota;c__Sordariomycetes;o__Hypocreales;f__Nectriaceae;g__Rodentomyces | 0 | 0 | 0 | 0 | 2.25E-05 | 9.01E-05 | 6.75E-05 | 2.25E-05 | 0 | 0 | 6.75E-05 | 9.01E-05 |
| d__Eukaryota;k__Fungi;p__Ascomycota;c__Leotiomycetes;o__Helotiales;f__Hyaloscyphaceae;g__Venturiocistella | 2.25E-05 | 0 | 0 | 0.000225139 | 0 | 0 | 2.25E-05 | 0 | 0 | 0 | 9.01E-05 | 0 |
| d__Eukaryota;k__Fungi;p__Ascomycota;c__Dothideomycetes;o__Dothideomycetes_ord_Incertae_sedis;f__Eremomycetaceae;g__unclassified_f__Eremomycetaceae | 0 | 0 | 0 | 0 | 0 | 0 | 0.000270167 | 6.75E-05 | 2.25E-05 | 0 | 0 | 0 |
| d__Eukaryota;k__Fungi;p__Ascomycota;c__Saccharomycetes;o__Saccharomycetales;f__Saccharomycetales_fam_Incertae_sedis;g__Candida | 0 | 0 | 0 | 6.75E-05 | 6.75E-05 | 0 | 9.01E-05 | 0.00011257 | 2.25E-05 | 0 | 0 | 0 |
| d__Eukaryota;k__Fungi;p__Ascomycota;c__Eurotiomycetes;o__Chaetothyriales;f__Trichomeriaceae;g__unclassified_f__Trichomeriaceae | 4.50E-05 | 0 | 0 | 0 | 6.75E-05 | 0.000180111 | 0 | 0 | 0 | 0 | 4.50E-05 | 0 |
| d__Eukaryota;k__Fungi;p__Ascomycota;c__Sordariomycetes;o__Chaetosphaeriales;f__Chaetosphaeriaceae;g__Dinemasporium | 0 | 0 | 0 | 0 | 0 | 0 | 2.25E-05 | 0.000247653 | 6.75E-05 | 0 | 0 | 0 |
| d__Eukaryota;k__Fungi;p__Ascomycota;c__Leotiomycetes;o__unclassified_c__Leotiomycetes;f__unclassified_c__Leotiomycetes;g__unclassified_c__Leotiomycetes | 0 | 0 | 6.75E-05 | 0 | 0 | 0 | 0.000225139 | 0 | 0 | 4.50E-05 | 0 | 0 |
| d__Eukaryota;k__Fungi;p__Ascomycota;c__Sordariomycetes;o__Xylariales;f__Amphisphaeriaceae;g__Seimatosporium | 0 | 0 | 0 | 0.000292681 | 0 | 0 | 4.50E-05 | 0 | 0 | 0 | 0 | 0 |
| d__Eukaryota;k__Fungi;p__Basidiomycota;c__Agaricomycetes;o__Agaricales;f__Physalacriaceae;g__Cylindrobasidium | 0 | 0 | 0 | 0.000337709 | 0 | 0 | 0 | 0 | 0 | 0 | 0 | 0 |
| d__Eukaryota;k__Fungi;p__Basidiomycota;c__Agaricomycetes;o__Polyporales;f__Steccherinaceae;g__unclassified_f__Steccherinaceae | 2.25E-05 | 0 | 0 | 0.000180111 | 0 | 0 | 0 | 0 | 2.25E-05 | 2.25E-05 | 6.75E-05 | 0 |
| d__Eukaryota;k__Fungi;p__Ascomycota;c__Dothideomycetes;o__Pleosporales;f__Pleomassariaceae;g__unclassified_f__Pleomassariaceae | 0 | 0.000315195 | 0 | 0 | 0 | 0 | 0 | 0 | 0 | 0 | 0 | 0 |
| d__Eukaryota;k__Fungi;p__Ascomycota;c__Orbiliomycetes;o__Orbiliales;f__Orbiliaceae;g__Dactylella | 0 | 0 | 0.000180111 | 0 | 0 | 0 | 0 | 0 | 0 | 0 | 0 | 0.000135083 |
| d__Eukaryota;k__Fungi;p__Ascomycota;c__Dothideomycetes;o__Capnodiales;f__Mycosphaerellaceae;g__Septoria | 0 | 0 | 0 | 0 | 2.25E-05 | 0 | 6.75E-05 | 0.000225139 | 0 | 0 | 0 | 0 |
| d__Eukaryota;k__Fungi;p__Basidiomycota;c__Agaricomycetes;o__Agaricales;f__Tricholomataceae;g__Arrhenia | 0 | 0 | 0 | 0 | 0 | 0 | 0 | 0 | 0 | 2.25E-05 | 0.000292681 | 0 |
| d__Eukaryota;k__Fungi;p__Basidiomycota;c__Tremellomycetes;o__Tremellales;f__Cuniculitremaceae;g__Fellomyces | 0 | 0 | 0 | 0 | 0 | 0 | 0.000292681 | 0 | 2.25E-05 | 0 | 0 | 0 |
| d__Eukaryota;k__Fungi;p__Ascomycota;c__Dothideomycetes;o__Venturiales;f__Sympoventuriaceae;g__unclassified_f__Sympoventuriaceae | 0 | 0 | 0 | 0 | 0 | 0 | 0 | 2.25E-05 | 0 | 9.01E-05 | 0.000135083 | 6.75E-05 |
| d__Eukaryota;k__Fungi;p__Chytridiomycota;c__Lobulomycetes;o__unclassified_c__Lobulomycetes;f__unclassified_c__Lobulomycetes;g__unclassified_c__Lobulomycetes | 0 | 0 | 0 | 0 | 0 | 0 | 0 | 6.75E-05 | 4.50E-05 | 0 | 0.000180111 | 0 |
| d__Eukaryota;k__Fungi;p__Ascomycota;c__Dothideomycetes;o__Pleosporales;f__Lophiostomataceae;g__Lophiostoma | 0 | 0 | 0 | 0.000247653 | 0 | 0 | 0 | 0 | 2.25E-05 | 2.25E-05 | 0 | 0 |
| d__Eukaryota;k__Fungi;p__Ascomycota;c__Sordariomycetes;o__Hypoceales;f__Catabotrydaceae;g__Calostilbe | 0 | 0 | 0 | 9.01E-05 | 0 | 9.01E-05 | 0 | 0 | 0 | 4.50E-05 | 2.25E-05 | 4.50E-05 |
| d__Eukaryota;k__Fungi;p__Basidiomycota;c__Tremellomycetes;o__Cystofilobasidiales;f__Mrakiaceae;g__Mrakia | 4.50E-05 | 4.50E-05 | 0 | 0 | 0 | 0 | 9.01E-05 | 4.50E-05 | 6.75E-05 | 0 | 0 | 0 |
| d__Eukaryota;k__Fungi;p__Basidiomycota;c__Agaricomycetes;o__Auriculariales;f__Exidiaceae;g__unclassified_f__Exidiaceae | 0 | 0 | 0 | 0.000292681 | 0 | 0 | 0 | 0 | 0 | 0 | 0 | 0 |
| d__Eukaryota;k__Fungi;p__Basidiomycota;c__Dacrymycetes;o__Dacrymycetales;f__Dacrymycetaceae;g__Dacrymyces | 0 | 0 | 0 | 0.000292681 | 0 | 0 | 0 | 0 | 0 | 0 | 0 | 0 |
| d__Eukaryota;k__Fungi;p__Chytridiomycota;c__Chytridiomycetes;o__Chytridiales;f__Chytridiaceae;g__Phlyctochytrium | 0.000202625 | 0 | 0 | 0 | 0 | 0 | 4.50E-05 | 0 | 0 | 0 | 0 | 4.50E-05 |
| d__Eukaryota;k__Fungi;p__Ascomycota;c__Dothideomycetes;o__Minutisphaerales;f__Minutisphaeraceae;g__Minutisphaera | 2.25E-05 | 0.00011257 | 0 | 4.50E-05 | 4.50E-05 | 2.25E-05 | 0 | 2.25E-05 | 2.25E-05 | 0 | 0 | 0 |
| d__Eukaryota;k__Fungi;p__Ascomycota;c__Saccharomycetes;o__Saccharomycetales;f__Phaffomycetaceae;g__Barnettozyma | 0.000180111 | 0 | 4.50E-05 | 0 | 0 | 4.50E-05 | 0 | 0 | 0 | 0 | 0 | 0 |
| d__Eukaryota;k__Fungi;p__Entorrhizomycota;c__Entorrhizomycetes;o__Entorrhizales;f__Entorrhizaceae;g__Entorrhiza | 0 | 0 | 0 | 0 | 0 | 0 | 0.000180111 | 2.25E-05 | 6.75E-05 | 0 | 0 | 0 |
| d__Eukaryota;k__Fungi;p__Basidiomycota;c__Agaricomycetes;o__Boletales;f__Sclerodermataceae;g__Scleroderma | 0 | 0 | 0 | 0.000157597 | 0 | 2.25E-05 | 9.01E-05 | 0 | 0 | 0 | 0 | 0 |
| d__Eukaryota;k__Fungi;p__Ascomycota;c__Dothideomycetes;o__Pleosporales;f__Phaeosphaeriaceae;g__Setophoma | 2.25E-05 | 4.50E-05 | 0 | 0 | 6.75E-05 | 4.50E-05 | 0 | 6.75E-05 | 0 | 0 | 2.25E-05 | 0 |
| d__Eukaryota;k__Fungi;p__Ascomycota;c__Orbiliomycetes;o__Orbiliales;f__Orbiliaceae;g__unclassified_f__Orbiliaceae | 0 | 4.50E-05 | 0 | 0 | 0 | 0 | 0 | 0 | 0 | 6.75E-05 | 2.25E-05 | 0.000135083 |
| d__Eukaryota;k__Fungi;p__Ascomycota;c__Sordariomycetes;o__Hypocreales;f__Ophiocordycipitaceae;g__Purpureocillium | 0 | 0 | 0 | 6.75E-05 | 0 | 0 | 0.000135083 | 0 | 0 | 2.25E-05 | 2.25E-05 | 2.25E-05 |
| d__Eukaryota;k__Fungi;p__Kickxellomycota;c__Kickxellomycetes;o__Kickxellales;f__Kickxellaceae;g__Coemansia | 0 | 0 | 4.50E-05 | 0 | 0.00011257 | 2.25E-05 | 6.75E-05 | 0 | 2.25E-05 | 0 | 0 | 0 |
| d__Eukaryota;k__Fungi;p__Ascomycota;c__Lecanoromycetes;o__Lecanorales;f__Ramalinaceae;g__Bacidia | 0 | 0 | 0 | 0 | 0 | 0.000247653 | 0 | 0 | 0 | 0 | 0 | 0 |
| d__Eukaryota;k__Fungi;p__Ascomycota;c__Sordariomycetes;o__Xylariales;f__Xylariaceae;g__Daldinia | 0 | 0 | 0 | 0 | 0.000247653 | 0 | 0 | 0 | 0 | 0 | 0 | 0 |
| d__Eukaryota;k__Fungi;p__Ascomycota;c__Sordariomycetes;o__Hypocreales;f__Stachybotryaceae;g__Albifimbria | 0 | 2.25E-05 | 0 | 0 | 0 | 0 | 0 | 4.50E-05 | 2.25E-05 | 0 | 0 | 0.000157597 |
| d__Eukaryota;k__Fungi;p__Ascomycota;c__Sordariomycetes;o__Hypocreales;f__Hypocreales_fam_Incertae_sedis;g__Hapsidospora | 0 | 6.75E-05 | 6.75E-05 | 0 | 0 | 0 | 6.75E-05 | 2.25E-05 | 2.25E-05 | 0 | 0 | 0 |
| d__Eukaryota;k__Fungi;p__Ascomycota;c__Sordariomycetes;o__Microascales;f__Graphiaceae;g__Graphium | 0 | 0 | 0 | 0 | 0 | 0 | 4.50E-05 | 0 | 4.50E-05 | 2.25E-05 | 6.75E-05 | 6.75E-05 |
| d__Eukaryota;k__Fungi;p__Zoopagomycota;c__unclassified_p__Zoopagomycota;o__unclassified_p__Zoopagomycota;f__unclassified_p__Zoopagomycota;g__unclassified_p__Zoopagomycota | 0 | 0 | 9.01E-05 | 0 | 4.50E-05 | 6.75E-05 | 0 | 0 | 4.50E-05 | 0 | 0 | 0 |
| d__Eukaryota;k__Fungi;p__Basidiomycota;c__Tremellomycetes;o__Tremellales;f__Trimorphomycetaceae;g__Carlosrosaea | 0 | 0 | 0 | 0 | 2.25E-05 | 0.000225139 | 0 | 0 | 0 | 0 | 0 | 0 |
| d__Eukaryota;k__Fungi;p__Ascomycota;c__Eurotiomycetes;o__Eurotiales;f__Thermoascaceae;g__Byssochlamys | 0 | 0 | 0 | 0 | 0 | 0 | 0 | 0 | 2.25E-05 | 0.000135083 | 9.01E-05 | 0 |
| d__Eukaryota;k__Fungi;p__Ascomycota;c__Leotiomycetes;o__Helotiales;f__Chaetomellaceae;g__Pilidium | 0 | 0 | 0 | 0 | 0 | 0.000135083 | 9.01E-05 | 0 | 2.25E-05 | 0 | 0 | 0 |
| d__Eukaryota;k__Fungi;p__Ascomycota;c__Dothideomycetes;o__Capnodiales;f__Mycosphaerellaceae;g__unclassified_f__Mycosphaerellaceae | 0 | 0 | 0 | 0 | 4.50E-05 | 0.000202625 | 0 | 0 | 0 | 0 | 0 | 0 |
| d__Eukaryota;k__Fungi;p__Ascomycota;c__Taphrinomycetes;o__Taphrinales;f__Taphrinaceae;g__Taphrina | 0 | 0 | 4.50E-05 | 0.000202625 | 0 | 0 | 0 | 0 | 0 | 0 | 0 | 0 |
| d__Eukaryota;k__Fungi;p__Basidiomycota;c__Agaricomycetes;o__Polyporales;f__Steccherinaceae;g__Fibricium | 4.50E-05 | 0 | 0 | 0.000202625 | 0 | 0 | 0 | 0 | 0 | 0 | 0 | 0 |
| d__Eukaryota;k__Fungi;p__Basidiomycota;c__Agaricomycetes;o__Polyporales;f__Hyphodermataceae;g__Hyphoderma | 0 | 0 | 0 | 0.000180111 | 4.50E-05 | 0 | 0 | 0 | 0 | 0 | 0 | 0 |
| d__Eukaryota;k__Fungi;p__Ascomycota;c__Sordariomycetes;o__Hypocreales;f__Bionectriaceae;g__Nectriella | 0 | 0 | 0 | 0 | 0.000157597 | 6.75E-05 | 0 | 0 | 0 | 0 | 0 | 0 |
| d__Eukaryota;k__Fungi;p__Basidiomycota;c__Agaricomycetes;o__Trechisporales;f__Hydnodontaceae;g__Brevicellicium | 0 | 0 | 0 | 4.50E-05 | 0 | 0.000157597 | 0 | 0 | 0 | 0 | 0 | 2.25E-05 |
| d__Eukaryota;k__Fungi;p__Ascomycota;c__Eurotiomycetes;o__Onygenales;f__Onygenaceae;g__Keratinophyton | 0 | 0 | 0 | 0 | 0.000225139 | 0 | 0 | 0 | 0 | 0 | 0 | 0 |
| d__Eukaryota;k__Fungi;p__Ascomycota;c__Sordariomycetes;o__Hypocreales;f__Hypocreales_fam_Incertae_sedis;g__Leucosphaerina | 0 | 0 | 0 | 0.000225139 | 0 | 0 | 0 | 0 | 0 | 0 | 0 | 0 |
| d__Eukaryota;k__Fungi;p__Basidiomycota;c__Agaricomycetes;o__Agaricales;f__Strophariaceae;g__Hypholoma | 6.75E-05 | 0 | 2.25E-05 | 0 | 0 | 0 | 0 | 2.25E-05 | 2.25E-05 | 9.01E-05 | 0 | 0 |
| d__Eukaryota;k__Fungi;p__Chytridiomycota;c__Spizellomycetes;o__Spizellomycetales;f__Spizellomycetaceae;g__Spizellomyces | 0 | 0 | 0 | 0 | 0 | 0 | 0 | 0 | 0.000225139 | 0 | 0 | 0 |
| d__Eukaryota;k__Fungi;p__Chytridiomycota;c__Synchytriomycetes;o__Synchytriales;f__Synchytriaceae;g__unclassified_f__Synchytriaceae | 2.25E-05 | 4.50E-05 | 0.000135083 | 0 | 0 | 0 | 0 | 0 | 0 | 0 | 2.25E-05 | 0 |
| d__Eukaryota;k__Fungi;p__Ascomycota;c__Dothideomycetes;o__Pleosporales;f__Phaeosphaeriaceae;g__Setophaeosphaeria | 0 | 2.25E-05 | 0 | 0.000202625 | 0 | 0 | 0 | 0 | 0 | 0 | 0 | 0 |
| d__Eukaryota;k__Fungi;p__Ascomycota;c__Sordariomycetes;o__Myrmecridiales;f__Myrmecridiales_fam_Incertae_sedis;g__Atractospora | 0 | 0 | 0 | 0 | 0 | 0 | 0 | 0 | 2.25E-05 | 0.000202625 | 0 | 0 |
| d__Eukaryota;k__Fungi;p__Ascomycota;c__Sordariomycetes;o__Hypocreales;f__Stachybotryaceae;g__unclassified_f__Stachybotryaceae | 0 | 0 | 0.00011257 | 0 | 0 | 0 | 0 | 0 | 0 | 0 | 2.25E-05 | 9.01E-05 |
| d__Eukaryota;k__Fungi;p__Ascomycota;c__Dothideomycetes;o__Pleosporales;f__Phaeosphaeriaceae;g__Phaeosphaeria | 0 | 0 | 0 | 0.00011257 | 0 | 0.00011257 | 0 | 0 | 0 | 0 | 0 | 0 |
| d__Eukaryota;k__Fungi;p__Ascomycota;c__Dothideomycetes;o__Pleosporales;f__Sporormiaceae;g__Chaetopreussia | 0 | 0 | 0 | 0 | 0 | 0 | 0 | 0.00011257 | 0.00011257 | 0 | 0 | 0 |
| d__Eukaryota;k__Fungi;p__Ascomycota;c__Orbiliomycetes;o__unclassified_c__Orbiliomycetes;f__unclassified_c__Orbiliomycetes;g__unclassified_c__Orbiliomycetes | 0 | 0 | 0 | 0 | 0 | 0 | 0 | 4.50E-05 | 0.000157597 | 0 | 0 | 0 |
| d__Eukaryota;k__Fungi;p__Ascomycota;c__Dothideomycetes;o__Pleosporales;f__Biatriosporaceae;g__Biatriospora | 0 | 6.75E-05 | 0 | 0 | 0 | 6.75E-05 | 2.25E-05 | 2.25E-05 | 2.25E-05 | 0 | 0 | 0 |
| d__Eukaryota;k__Fungi;p__Ascomycota;c__Sordariomycetes;o__Sordariales;f__Helminthosphaeriaceae;g__Endophragmiella | 0 | 6.75E-05 | 2.25E-05 | 0 | 0 | 0 | 0 | 0 | 0 | 4.50E-05 | 0 | 6.75E-05 |
| d__Eukaryota;k__Fungi;p__Glomeromycota;c__Archaeosporomycetes;o__Archaeosporales;f__unclassified_o__Archaeosporales;g__unclassified_o__Archaeosporales | 2.25E-05 | 2.25E-05 | 0 | 0 | 0 | 0 | 2.25E-05 | 0 | 0.000135083 | 0 | 0 | 0 |
| d__Eukaryota;k__Fungi;p__Basidiomycota;c__Agaricomycetes;o__Agaricales;f__Bolbitiaceae;g__unclassified_f__Bolbitiaceae | 0.000202625 | 0 | 0 | 0 | 0 | 0 | 0 | 0 | 0 | 0 | 0 | 0 |
| d__Eukaryota;k__Fungi;p__Basidiomycota;c__Agaricomycetes;o__Agaricales;f__Pterulaceae;g__Pterula | 0 | 0 | 0 | 0 | 0.000202625 | 0 | 0 | 0 | 0 | 0 | 0 | 0 |
| d__Eukaryota;k__Fungi;p__Basidiomycota;c__Agaricomycetes;o__Polyporales;f__Fomitopsidaceae;g__Postia | 0 | 0 | 0 | 0 | 0 | 0 | 0 | 0 | 0 | 0 | 0 | 0.000202625 |
| d__Eukaryota;k__Fungi;p__Basidiomycota;c__Agaricomycetes;o__Sebacinales;f__Serendipitaceae;g__unclassified_f__Serendipitaceae | 0 | 0 | 0 | 0 | 0 | 0 | 0 | 0 | 0.000202625 | 0 | 0 | 0 |
| d__Eukaryota;k__Fungi;p__Basidiomycota;c__Microbotryomycetes;o__Sporidiobolales;f__Sporidiobolaceae;g__Sporobolomyces | 0 | 0 | 0 | 0 | 0 | 0 | 0 | 0 | 0 | 0.000202625 | 0 | 0 |
| d__Eukaryota;k__Fungi;p__Basidiomycota;c__Tremellomycetes;o__Filobasidiales;f__Piskurozymaceae;g__Piskurozyma | 0 | 0 | 0.000202625 | 0 | 0 | 0 | 0 | 0 | 0 | 0 | 0 | 0 |
| d__Eukaryota;k__Fungi;p__Ascomycota;c__Dothideomycetes;o__Pleosporales;f__Didymosphaeriaceae;g__Pseudopithomyces | 2.25E-05 | 0 | 0 | 0 | 0 | 0 | 0 | 0 | 0 | 4.50E-05 | 0.00011257 | 2.25E-05 |
| d__Eukaryota;k__Fungi;p__Basidiomycota;c__Agaricostilbomycetes;o__Agaricostilbales;f__Chionosphaeraceae;g__unclassified_f__Chionosphaeraceae | 2.25E-05 | 0 | 4.50E-05 | 2.25E-05 | 0.00011257 | 0 | 0 | 0 | 0 | 0 | 0 | 0 |
| d__Eukaryota;k__Fungi;p__Ascomycota;c__Leotiomycetes;o__Helotiales;f__Helotiaceae;g__Culicidospora | 0 | 0 | 0.000180111 | 0 | 0 | 0 | 0 | 0 | 0 | 0 | 0 | 0 |
| d__Eukaryota;k__Fungi;p__Ascomycota;c__Dothideomycetes;o__Pleosporales;f__Lentitheciaceae;g__Poaceascoma | 2.25E-05 | 0 | 0 | 0 | 0 | 0 | 0 | 2.25E-05 | 4.50E-05 | 0 | 9.01E-05 | 0 |
| d__Eukaryota;k__Fungi;p__Ascomycota;c__Leotiomycetes;o__Helotiales;f__Helotiaceae;g__Dimorphospora | 0 | 0 | 6.75E-05 | 0 | 0 | 0 | 9.01E-05 | 0 | 2.25E-05 | 0 | 0 | 0 |
| d__Eukaryota;k__Fungi;p__Ascomycota;c__Sordariomycetes;o__Xylariales;f__Xylariaceae;g__Hypoxylon | 2.25E-05 | 0 | 0 | 9.01E-05 | 0 | 0 | 4.50E-05 | 0 | 2.25E-05 | 0 | 0 | 0 |
| d__Eukaryota;k__Fungi;p__Glomeromycota;c__Paraglomeromycetes;o__Paraglomerales;f__Paraglomeraceae;g__Paraglomus | 0 | 0 | 0 | 0 | 0 | 0 | 9.01E-05 | 9.01E-05 | 0 | 0 | 0 | 0 |
| d__Eukaryota;k__Fungi;p__Ascomycota;c__Sordariomycetes;o__Hypocreales;f__Cordycipitaceae;g__unclassified_f__Cordycipitaceae | 0 | 0 | 0 | 0 | 0 | 0 | 2.25E-05 | 0 | 0 | 0 | 0.000135083 | 2.25E-05 |
| d__Eukaryota;k__Fungi;p__Ascomycota;c__Eurotiomycetes;o__Verrucariales;f__Verrucariaceae;g__Verrucaria | 0 | 0 | 0 | 6.75E-05 | 0.00011257 | 0 | 0 | 0 | 0 | 0 | 0 | 0 |
| d__Eukaryota;k__Fungi;p__Ascomycota;c__Sordariomycetes;o__Xylariales;f__Xylariaceae;g__Nemania | 6.75E-05 | 0 | 0 | 0.00011257 | 0 | 0 | 0 | 0 | 0 | 0 | 0 | 0 |
| d__Eukaryota;k__Fungi;p__Ascomycota;c__Dothideomycetes;o__Capnodiales;f__Capnodiaceae;g__Leptoxyphium | 0 | 0 | 0 | 0 | 0.000157597 | 0 | 0 | 0 | 0 | 0 | 0 | 0 |
| d__Eukaryota;k__Fungi;p__Basidiomycota;c__Tremellomycetes;o__Tremellales;f__Bulleribasidiaceae;g__Hannaella | 0 | 0 | 0 | 0.000157597 | 0 | 0 | 0 | 0 | 0 | 0 | 0 | 0 |
| d__Eukaryota;k__Fungi;p__Basidiobolomycota;c__unclassified_p__Basidiobolomycota;o__unclassified_p__Basidiobolomycota;f__unclassified_p__Basidiobolomycota;g__unclassified_p__Basidiobolomycota | 0 | 0 | 0 | 0 | 2.25E-05 | 9.01E-05 | 2.25E-05 | 0 | 0 | 0 | 2.25E-05 | 0 |
| d__Eukaryota;k__Fungi;p__Ascomycota;c__Sordariomycetes;o__Hypocreales;f__Niessliaceae;g__Niesslia | 0 | 4.50E-05 | 6.75E-05 | 0 | 0 | 4.50E-05 | 0 | 0 | 0 | 0 | 0 | 0 |
| d__Eukaryota;k__Fungi;p__Basidiomycota;c__Agaricomycetes;o__Agaricales;f__Psathyrellaceae;g__Lacrymaria | 2.25E-05 | 0 | 4.50E-05 | 0 | 0 | 0 | 0 | 2.25E-05 | 2.25E-05 | 4.50E-05 | 0 | 0 |
| d__Eukaryota;k__Fungi;p__Basidiomycota;c__Agaricomycetes;o__Hymenochaetales;f__Schizoporaceae;g__Oxyporus | 0 | 0 | 0 | 0 | 0 | 0 | 0 | 0 | 6.75E-05 | 0 | 9.01E-05 | 0 |
| d__Eukaryota;k__Fungi;p__Basidiomycota;c__Agaricomycetes;o__Thelephorales;f__Thelephoraceae;g__Thelephora | 0 | 0 | 0 | 0 | 0 | 0 | 0 | 2.25E-05 | 4.50E-05 | 0 | 9.01E-05 | 0 |
| d__Eukaryota;k__Fungi;p__Chytridiomycota;c__Rhizophydiomycetes;o__Rhizophydiales;f__Rhizophydiaceae;g__Rhizophydium | 0 | 2.25E-05 | 0 | 9.01E-05 | 0 | 0 | 0 | 0 | 4.50E-05 | 0 | 0 | 0 |
| d__Eukaryota;k__Fungi;p__Ascomycota;c__Sordariomycetes;o__Sordariales;f__Chaetomiaceae;g__Acrophialophora | 0 | 0 | 0 | 0 | 0 | 0 | 4.50E-05 | 6.75E-05 | 4.50E-05 | 0 | 0 | 0 |
| d__Eukaryota;k__Fungi;p__Ascomycota;c__Leotiomycetes;o__Thelebolales;f__Pseudeurotiaceae;g__Leuconeurospora | 2.25E-05 | 0 | 0.000135083 | 0 | 0 | 0 | 0 | 0 | 0 | 0 | 0 | 0 |
| d__Eukaryota;k__Fungi;p__Ascomycota;c__Dothideomycetes;o__Botryosphaeriales;f__Botryosphaeriaceae;g__Microdiplodia | 0 | 0 | 2.25E-05 | 0 | 0 | 6.75E-05 | 2.25E-05 | 2.25E-05 | 0 | 0 | 0 | 0 |
| d__Eukaryota;k__Fungi;p__Ascomycota;c__Dothideomycetes;o__Capnodiales;f__Mycosphaerellaceae;g__Acrodontium | 0 | 2.25E-05 | 0 | 0 | 4.50E-05 | 6.75E-05 | 0 | 0 | 0 | 0 | 0 | 0 |
| d__Eukaryota;k__Fungi;p__Ascomycota;c__Dothideomycetes;o__Pleosporales;f__Cucurbitariaceae;g__unclassified_f__Cucurbitariaceae | 4.50E-05 | 2.25E-05 | 0 | 0 | 0 | 6.75E-05 | 0 | 0 | 0 | 0 | 0 | 0 |
| d__Eukaryota;k__Fungi;p__Ascomycota;c__Dothideomycetes;o__Pleosporales;f__Dictyosporiaceae;g__unclassified_f__Dictyosporiaceae | 0 | 0 | 0 | 0 | 0 | 0 | 0 | 0 | 2.25E-05 | 0 | 9.01E-05 | 2.25E-05 |
| d__Eukaryota;k__Fungi;p__Ascomycota;c__Dothideomycetes;o__Pleosporales;f__Didymosphaeriaceae;g__Tremateia | 0 | 0 | 0 | 0 | 0 | 0 | 6.75E-05 | 2.25E-05 | 0 | 0 | 4.50E-05 | 0 |
| d__Eukaryota;k__Fungi;p__Ascomycota;c__Sordariomycetes;o__Glomerellales;f__Plectosphaerellaceae;g__Sodiomyces | 0 | 2.25E-05 | 4.50E-05 | 0 | 0 | 0 | 0 | 2.25E-05 | 0 | 0 | 4.50E-05 | 0 |
| d__Eukaryota;k__Fungi;p__Ascomycota;c__Sordariomycetes;o__Xylariales;f__Apiosporaceae;g__Apiospora | 0 | 0 | 0 | 0 | 9.01E-05 | 0 | 0 | 0 | 2.25E-05 | 0 | 0 | 2.25E-05 |
| d__Eukaryota;k__Fungi;p__Ascomycota;c__Sordariomycetes;o__Xylariales;f__Sporocadaceae;g__Neopestalotiopsis | 0 | 4.50E-05 | 0 | 0 | 0 | 0 | 0 | 6.75E-05 | 0 | 0 | 2.25E-05 | 0 |
| d__Eukaryota;k__Fungi;p__Basidiomycota;c__Agaricomycetes;o__Agaricales;f__Inocybaceae;g__Inocybe | 0 | 0 | 4.50E-05 | 0 | 2.25E-05 | 0 | 0 | 0 | 2.25E-05 | 0 | 0 | 4.50E-05 |
| d__Eukaryota;k__Fungi;p__Basidiomycota;c__Agaricomycetes;o__Polyporales;f__Xenasmataceae;g__Xenasmatella | 0 | 0 | 0 | 0 | 0 | 0 | 6.75E-05 | 0 | 0 | 6.75E-05 | 0 | 0 |
| d__Eukaryota;k__Fungi;p__Basidiomycota;c__Cystobasidiomycetes;o__Cystobasidiomycetes_ord_Incertae_sedis;f__Symmetrosporaceae;g__Symmetrospora | 0 | 0 | 6.75E-05 | 0 | 0 | 6.75E-05 | 0 | 0 | 0 | 0 | 0 | 0 |
| d__Eukaryota;k__Fungi;p__Chytridiomycota;c__Rhizophydiomycetes;o__Rhizophydiales;f__Angulomycetaceae;g__Angulomyces | 4.50E-05 | 4.50E-05 | 0 | 0 | 0 | 0 | 0 | 0 | 4.50E-05 | 0 | 0 | 0 |
| d__Eukaryota;k__Fungi;p__Ascomycota;c__Archaeorhizomycetes;o__Archaeorhizomycetales;f__Archaeorhizomycetaceae;g__Archaeorhizomyces | 0 | 0 | 0 | 0 | 0 | 0 | 6.75E-05 | 2.25E-05 | 4.50E-05 | 0 | 0 | 0 |
| d__Eukaryota;k__Fungi;p__Ascomycota;c__Laboulbeniomycetes;o__Pyxidiophorales;f__unclassified_o__Pyxidiophorales;g__unclassified_o__Pyxidiophorales | 9.01E-05 | 4.50E-05 | 0 | 0 | 0 | 0 | 0 | 0 | 0 | 0 | 0 | 0 |
| d__Eukaryota;k__Fungi;p__Ascomycota;c__Leotiomycetes;o__Thelebolales;f__Pseudeurotiaceae;g__unclassified_f__Pseudeurotiaceae | 0 | 6.75E-05 | 6.75E-05 | 0 | 0 | 0 | 0 | 0 | 0 | 0 | 0 | 0 |
| d__Eukaryota;k__Fungi;p__Basidiomycota;c__Agaricomycetes;o__Polyporales;f__Podoscyphaceae;g__Cotylidia | 4.50E-05 | 6.75E-05 | 2.25E-05 | 0 | 0 | 0 | 0 | 0 | 0 | 0 | 0 | 0 |
| d__Eukaryota;k__Fungi;p__Ascomycota;c__Lecanoromycetes;o__Ostropales;f__Stictidaceae;g__unclassified_f__Stictidaceae | 0 | 0 | 0 | 0.000135083 | 0 | 0 | 0 | 0 | 0 | 0 | 0 | 0 |
| d__Eukaryota;k__Fungi;p__Ascomycota;c__Sordariomycetes;o__Xylariales;f__Xylariaceae;g__Rosellinia | 0 | 0 | 0 | 0 | 0 | 0 | 0 | 0 | 0.000135083 | 0 | 0 | 0 |
| d__Eukaryota;k__Fungi;p__Basidiomycota;c__Agaricomycetes;o__Polyporales;f__Meruliaceae;g__Efibula | 0 | 0 | 0 | 0.000135083 | 0 | 0 | 0 | 0 | 0 | 0 | 0 | 0 |
| d__Eukaryota;k__Fungi;p__Ascomycota;c__Sordariomycetes;o__Sordariales;f__Lasiosphaeriaceae;g__Jugulospora | 0 | 0.00011257 | 0 | 0 | 0 | 0 | 0 | 0 | 0 | 0 | 2.25E-05 | 0 |
| d__Eukaryota;k__Fungi;p__Basidiomycota;c__Agaricomycetes;o__Agaricales;f__Agaricaceae;g__Agaricus | 0 | 0 | 0 | 0 | 0 | 0.00011257 | 0 | 0 | 0 | 2.25E-05 | 0 | 0 |
| d__Eukaryota;k__Fungi;p__Ascomycota;c__Eurotiomycetes;o__Onygenales;f__Arachnomycetaceae;g__Onychocola | 0 | 0 | 0 | 0 | 0 | 0 | 0 | 2.25E-05 | 9.01E-05 | 0 | 0 | 0 |
| d__Eukaryota;k__Fungi;p__Ascomycota;c__Sordariomycetes;o__Hypocreales;f__Hypocreaceae;g__unclassified_f__Hypocreaceae | 4.50E-05 | 0 | 0 | 0 | 2.25E-05 | 0 | 0 | 0 | 2.25E-05 | 0 | 0 | 2.25E-05 |
| d__Eukaryota;k__Fungi;p__Basidiomycota;c__Tremellomycetes;o__Cystofilobasidiales;f__Cystofilobasidiaceae;g__Cystofilobasidium | 0 | 0 | 2.25E-05 | 0 | 0 | 4.50E-05 | 2.25E-05 | 0 | 2.25E-05 | 0 | 0 | 0 |
| d__Eukaryota;k__Fungi;p__Rozellomycota;c__Rozellomycotina_cls_Incertae_sedis;o__Branch02;f__unclassified_o__Branch02;g__unclassified_o__Branch02 | 0 | 0 | 0 | 0 | 0 | 0 | 2.25E-05 | 9.01E-05 | 0 | 0 | 0 | 0 |
| d__Eukaryota;k__Fungi;p__Aphelidiomycota;c__Aphelidiomycetes;o__GS16;f__unclassified_o__GS16;g__unclassified_o__GS16 | 2.25E-05 | 0 | 0 | 6.75E-05 | 0 | 0 | 0 | 0 | 0 | 2.25E-05 | 0 | 0 |
| d__Eukaryota;k__Fungi;p__Ascomycota;c__Dothideomycetes;o__Capnodiales;f__Mycosphaerellaceae;g__Zymoseptoria | 0 | 9.01E-05 | 0 | 0 | 2.25E-05 | 0 | 0 | 0 | 0 | 0 | 0 | 0 |
| d__Eukaryota;k__Fungi;p__Ascomycota;c__Dothideomycetes;o__Pleosporales;f__Dictyosporiaceae;g__Dictyosporium | 0 | 0 | 0 | 0 | 4.50E-05 | 0 | 0 | 0 | 0 | 0 | 0 | 6.75E-05 |
| d__Eukaryota;k__Fungi;p__Ascomycota;c__Dothideomycetes;o__Pleosporales;f__Didymellaceae;g__Leptosphaerulina | 0 | 0 | 9.01E-05 | 0 | 0 | 0 | 0 | 0 | 2.25E-05 | 0 | 0 | 0 |
| d__Eukaryota;k__Fungi;p__Ascomycota;c__Dothideomycetes;o__Pleosporales;f__Phaeosphaeriaceae;g__Leptospora | 0 | 2.25E-05 | 0 | 9.01E-05 | 0 | 0 | 0 | 0 | 0 | 0 | 0 | 0 |
| d__Eukaryota;k__Fungi;p__Ascomycota;c__Eurotiomycetes;o__Chaetothyriales;f__Herpotrichiellaceae;g__Melanoctona | 0 | 0 | 0 | 0 | 0 | 0 | 4.50E-05 | 0 | 6.75E-05 | 0 | 0 | 0 |
| d__Eukaryota;k__Fungi;p__Ascomycota;c__Sordariomycetes;o__Melanosporales;f__Ceratostomataceae;g__Harzia | 0 | 0 | 0 | 0 | 4.50E-05 | 0 | 0 | 0 | 6.75E-05 | 0 | 0 | 0 |
| d__Eukaryota;k__Fungi;p__Ascomycota;c__Sordariomycetes;o__Microascales;f__Microascales_fam_Incertae_sedis;g__Cephalotrichiella | 2.25E-05 | 2.25E-05 | 4.50E-05 | 0 | 0 | 0 | 0 | 0 | 2.25E-05 | 0 | 0 | 0 |
| d__Eukaryota;k__Fungi;p__Ascomycota;c__Sordariomycetes;o__Xylariales;f__Xylariaceae;g__Xylaria | 0 | 6.75E-05 | 0 | 0 | 4.50E-05 | 0 | 0 | 0 | 0 | 0 | 0 | 0 |
| d__Eukaryota;k__Fungi;p__Basidiomycota;c__Agaricomycetes;o__Agaricales;f__Strophariaceae;g__Pholiota | 0 | 0 | 0 | 9.01E-05 | 0 | 0 | 2.25E-05 | 0 | 0 | 0 | 0 | 0 |
| d__Eukaryota;k__Fungi;p__Basidiomycota;c__Agaricomycetes;o__Atheliales;f__Atheliaceae;g__Amphinema | 6.75E-05 | 0 | 0 | 0 | 0 | 0 | 0 | 0 | 0 | 0 | 0 | 4.50E-05 |
| d__Eukaryota;k__Fungi;p__Basidiomycota;c__Agaricomycetes;o__Corticiales;f__Corticiaceae;g__Galzinia | 4.50E-05 | 2.25E-05 | 0 | 0 | 0 | 0 | 0 | 0 | 0 | 0 | 0 | 4.50E-05 |
| d__Eukaryota;k__Fungi;p__Basidiomycota;c__Agaricomycetes;o__Hymenochaetales;f__Hymenochaetales_fam_Incertae_sedis;g__Resinicium | 0 | 0 | 0 | 6.75E-05 | 0 | 4.50E-05 | 0 | 0 | 0 | 0 | 0 | 0 |
| d__Eukaryota;k__Fungi;p__Basidiomycota;c__Agaricomycetes;o__Polyporales;f__Cerrenaceae;g__Panus | 0 | 0 | 0 | 0 | 6.75E-05 | 4.50E-05 | 0 | 0 | 0 | 0 | 0 | 0 |
| d__Eukaryota;k__Fungi;p__Basidiomycota;c__Agaricostilbomycetes;o__Agaricostilbales;f__Chionosphaeraceae;g__Kurtzmanomyces | 0 | 0 | 0 | 0 | 2.25E-05 | 6.75E-05 | 0 | 2.25E-05 | 0 | 0 | 0 | 0 |
| d__Eukaryota;k__Fungi;p__Chytridiomycota;c__Spizellomycetes;o__Spizellomycetales;f__Powellomycetaceae;g__unclassified_f__Powellomycetaceae | 4.50E-05 | 0 | 0 | 6.75E-05 | 0 | 0 | 0 | 0 | 0 | 0 | 0 | 0 |
| d__Eukaryota;k__Fungi;p__Ascomycota;c__Archaeorhizomycetes;o__unclassified_c__Archaeorhizomycetes;f__unclassified_c__Archaeorhizomycetes;g__unclassified_c__Archaeorhizomycetes | 0 | 0 | 0 | 0.00011257 | 0 | 0 | 0 | 0 | 0 | 0 | 0 | 0 |
| d__Eukaryota;k__Fungi;p__Ascomycota;c__Sordariomycetes;o__Conioscyphales;f__unclassified_o__Conioscyphales;g__unclassified_o__Conioscyphales | 0 | 0 | 0 | 0 | 0 | 0 | 0.00011257 | 0 | 0 | 0 | 0 | 0 |
| d__Eukaryota;k__Fungi;p__Ascomycota;c__Sordariomycetes;o__Hypocreales;f__Cordycipitaceae;g__Leptobacillium | 0 | 0 | 0 | 0 | 0.00011257 | 0 | 0 | 0 | 0 | 0 | 0 | 0 |
| d__Eukaryota;k__Fungi;p__Ascomycota;c__Sordariomycetes;o__Melanosporales;f__Melanosporaceae;g__Melanospora | 0 | 0 | 0 | 0 | 0 | 0 | 0 | 0 | 0.00011257 | 0 | 0 | 0 |
| d__Eukaryota;k__Fungi;p__Basidiomycota;c__Agaricomycetes;o__Agaricales;f__Entolomataceae;g__Clitopilus | 0 | 0 | 0 | 0 | 0 | 0 | 0 | 0 | 0.00011257 | 0 | 0 | 0 |
| d__Eukaryota;k__Fungi;p__Basidiomycota;c__Agaricomycetes;o__Polyporales;f__Polyporaceae;g__Echinochaete | 0 | 0 | 0 | 0 | 0 | 0.00011257 | 0 | 0 | 0 | 0 | 0 | 0 |
| d__Eukaryota;k__Fungi;p__Basidiomycota;c__Pucciniomycetes;o__Platygloeales;f__Platygloeaceae;g__unclassified_f__Platygloeaceae | 0 | 0 | 0.00011257 | 0 | 0 | 0 | 0 | 0 | 0 | 0 | 0 | 0 |
| d__Eukaryota;k__Fungi;p__Ascomycota;c__Dothideomycetes;o__Dothideales;f__Aureobasidiaceae;g__Aureobasidium | 0 | 0 | 0 | 4.50E-05 | 0 | 0 | 0 | 4.50E-05 | 0 | 0 | 0 | 0 |
| d__Eukaryota;k__Fungi;p__Ascomycota;c__Dothideomycetes;o__Pleosporales;f__Lentitheciaceae;g__Keissleriella | 0 | 0 | 0 | 0 | 0 | 2.25E-05 | 2.25E-05 | 0 | 0 | 0 | 2.25E-05 | 2.25E-05 |
| d__Eukaryota;k__Fungi;p__Ascomycota;c__Dothideomycetes;o__Pleosporales;f__Phaeosphaeriaceae;g__Ophiosphaerella | 0 | 0 | 4.50E-05 | 0 | 0 | 0 | 0 | 0 | 4.50E-05 | 0 | 0 | 0 |
| d__Eukaryota;k__Fungi;p__Ascomycota;c__Dothideomycetes;o__Venturiales;f__Sympoventuriaceae;g__Pseudosigmoidea | 0 | 0 | 0 | 0 | 0 | 4.50E-05 | 2.25E-05 | 0 | 2.25E-05 | 0 | 0 | 0 |
| d__Eukaryota;k__Fungi;p__Ascomycota;c__Sordariomycetes;o__Xylariales;f__Diatrypaceae;g__unclassified_f__Diatrypaceae | 0 | 0 | 0 | 0 | 0 | 4.50E-05 | 0 | 0 | 4.50E-05 | 0 | 0 | 0 |
| d__Eukaryota;k__Fungi;p__Ascomycota;c__Taphrinomycetes;o__unclassified_c__Taphrinomycetes;f__unclassified_c__Taphrinomycetes;g__unclassified_c__Taphrinomycetes | 0 | 2.25E-05 | 2.25E-05 | 0 | 0 | 4.50E-05 | 0 | 0 | 0 | 0 | 0 | 0 |
| d__Eukaryota;k__Fungi;p__Ascomycota;c__Dothideomycetes;o__Capnodiales;f__Teratosphaeriaceae;g__Devriesia | 9.01E-05 | 0 | 0 | 0 | 0 | 0 | 0 | 0 | 0 | 0 | 0 | 0 |
| d__Eukaryota;k__Fungi;p__Ascomycota;c__Dothideomycetes;o__Capnodiales;f__Teratosphaeriaceae;g__unclassified_f__Teratosphaeriaceae | 0 | 0 | 0 | 0 | 9.01E-05 | 0 | 0 | 0 | 0 | 0 | 0 | 0 |
| d__Eukaryota;k__Fungi;p__Ascomycota;c__Dothideomycetes;o__Pleosporales;f__Lophiostomataceae;g__Biappendiculispora | 0 | 0 | 0 | 0 | 0 | 0 | 0 | 9.01E-05 | 0 | 0 | 0 | 0 |
| d__Eukaryota;k__Fungi;p__Ascomycota;c__Dothideomycetes;o__Pleosporales;f__Massarinaceae;g__Helminthosporium | 0 | 0 | 0 | 0 | 0 | 0 | 0 | 0 | 0 | 0 | 2.25E-05 | 6.75E-05 |
| d__Eukaryota;k__Fungi;p__Ascomycota;c__Eurotiomycetes;o__Eurotiales;f__Aspergillaceae;g__unclassified_f__Aspergillaceae | 0 | 0 | 0 | 0 | 0 | 9.01E-05 | 0 | 0 | 0 | 0 | 0 | 0 |
| d__Eukaryota;k__Fungi;p__Ascomycota;c__Pezizomycetes;o__Pezizales;f__Pyronemataceae;g__Sphaerosporella | 0 | 0 | 0 | 0 | 9.01E-05 | 0 | 0 | 0 | 0 | 0 | 0 | 0 |
| d__Eukaryota;k__Fungi;p__Ascomycota;c__Sordariomycetes;o__Hypoceales;f__Catabotrydaceae;g__Botryosporium | 0 | 0 | 0 | 0 | 2.25E-05 | 6.75E-05 | 0 | 0 | 0 | 0 | 0 | 0 |
| d__Eukaryota;k__Fungi;p__Ascomycota;c__Sordariomycetes;o__Hypocreales;f__Bionectriaceae;g__unclassified_f__Bionectriaceae | 0 | 0 | 0 | 0 | 0 | 9.01E-05 | 0 | 0 | 0 | 0 | 0 | 0 |
| d__Eukaryota;k__Fungi;p__Ascomycota;c__Sordariomycetes;o__Melanosporales;f__Ceratostomataceae;g__Microthecium | 0 | 0 | 0 | 0 | 0 | 0 | 0 | 0 | 9.01E-05 | 0 | 0 | 0 |
| d__Eukaryota;k__Fungi;p__Ascomycota;c__Sordariomycetes;o__Sordariales;f__Lasiosphaeriaceae;g__Immersiella | 0 | 0 | 0 | 0 | 0 | 0 | 4.50E-05 | 4.50E-05 | 0 | 0 | 0 | 0 |
| d__Eukaryota;k__Fungi;p__Basidiomycota;c__Agaricomycetes;o__Agaricales;f__Tricholomataceae;g__Macrocystidia | 0 | 0 | 0 | 0 | 0 | 0 | 0 | 0 | 0 | 2.25E-05 | 6.75E-05 | 0 |
| d__Eukaryota;k__Fungi;p__Basidiomycota;c__Agaricomycetes;o__Hymenochaetales;f__Schizoporaceae;g__unclassified_f__Schizoporaceae | 0 | 0 | 0 | 0 | 0 | 9.01E-05 | 0 | 0 | 0 | 0 | 0 | 0 |
| d__Eukaryota;k__Fungi;p__Basidiomycota;c__Agaricomycetes;o__Polyporales;f__Podoscyphaceae;g__Hypochnicium | 0 | 0 | 0 | 0 | 0 | 0 | 0 | 0 | 0 | 0 | 9.01E-05 | 0 |
| d__Eukaryota;k__Fungi;p__Basidiomycota;c__Agaricomycetes;o__Sebacinales;f__Sebacinaceae;g__Efibulobasidium | 0 | 0 | 0 | 0 | 9.01E-05 | 0 | 0 | 0 | 0 | 0 | 0 | 0 |
| d__Eukaryota;k__Fungi;p__Rozellomycota;c__Rozellomycotina_cls_Incertae_sedis;o__GS10;f__unclassified_o__GS10;g__unclassified_o__GS10 | 0 | 0 | 0 | 9.01E-05 | 0 | 0 | 0 | 0 | 0 | 0 | 0 | 0 |
| d__Eukaryota;k__Fungi;p__Ascomycota;c__Dothideomycetes;o__Myriangiales;f__Myriangiaceae;g__Myriangium | 0 | 0 | 0 | 6.75E-05 | 0 | 0 | 0 | 0 | 0 | 0 | 2.25E-05 | 0 |
| d__Eukaryota;k__Fungi;p__Ascomycota;c__Dothideomycetes;o__Pleosporales;f__Coniothyriaceae;g__unclassified_f__Coniothyriaceae | 2.25E-05 | 0 | 0 | 0 | 6.75E-05 | 0 | 0 | 0 | 0 | 0 | 0 | 0 |
| d__Eukaryota;k__Fungi;p__Ascomycota;c__Pezizomycotina_cls_Incertae_sedis;o__Pezizomycotina_ord_Incertae_sedis;f__Pezizomycotina_fam_Incertae_sedis;g__Arcuadendron | 0 | 0 | 0 | 0 | 0 | 6.75E-05 | 0 | 0 | 2.25E-05 | 0 | 0 | 0 |
| d__Eukaryota;k__Fungi;p__Basidiomycota;c__Agaricomycetes;o__Polyporales;f__Polyporaceae;g__Lentinus | 0 | 2.25E-05 | 0 | 6.75E-05 | 0 | 0 | 0 | 0 | 0 | 0 | 0 | 0 |
| d__Eukaryota;k__Fungi;p__Basidiomycota;c__Agaricomycetes;o__Polyporales;f__unclassified_o__Polyporales;g__unclassified_o__Polyporales | 0 | 0 | 0 | 0 | 0 | 2.25E-05 | 0 | 0 | 6.75E-05 | 0 | 0 | 0 |
| d__Eukaryota;k__Fungi;p__Basidiomycota;c__Agaricomycetes;o__Trechisporales;f__unclassified_o__Trechisporales;g__unclassified_o__Trechisporales | 4.50E-05 | 2.25E-05 | 0 | 2.25E-05 | 0 | 0 | 0 | 0 | 0 | 0 | 0 | 0 |
| d__Eukaryota;k__Fungi;p__Basidiomycota;c__Tremellomycetes;o__Filobasidiales;f__Filobasidiaceae;g__Filobasidium | 0 | 0 | 0 | 6.75E-05 | 0 | 0 | 0 | 0 | 0 | 0 | 2.25E-05 | 0 |
| d__Eukaryota;k__Fungi;p__Ascomycota;c__Dothideomycetes;o__Capnodiales;f__Cladosporiaceae;g__Rachicladosporium | 0 | 2.25E-05 | 0 | 0 | 0 | 0 | 0 | 0 | 4.50E-05 | 0 | 0 | 0 |
| d__Eukaryota;k__Fungi;p__Ascomycota;c__Leotiomycetes;o__Helotiales;f__Helotiaceae;g__Articulospora | 0 | 0 | 0 | 4.50E-05 | 0 | 0 | 0 | 0 | 2.25E-05 | 0 | 0 | 0 |
| d__Eukaryota;k__Fungi;p__Ascomycota;c__Saccharomycetes;o__Saccharomycetales;f__Debaryomycetaceae;g__Debaryomyces | 0 | 0 | 2.25E-05 | 4.50E-05 | 0 | 0 | 0 | 0 | 0 | 0 | 0 | 0 |
| d__Eukaryota;k__Fungi;p__Ascomycota;c__Sordariomycetes;o__Glomerellales;f__unclassified_o__Glomerellales;g__unclassified_o__Glomerellales | 0 | 0 | 2.25E-05 | 0 | 0 | 4.50E-05 | 0 | 0 | 0 | 0 | 0 | 0 |
| d__Eukaryota;k__Fungi;p__Ascomycota;c__Sordariomycetes;o__Xylariales;f__Diatrypaceae;g__Peroneutypa | 0 | 0 | 0 | 0 | 0 | 0 | 2.25E-05 | 0 | 0 | 2.25E-05 | 2.25E-05 | 0 |
| d__Eukaryota;k__Fungi;p__Basidiomycota;c__Agaricomycetes;o__Agaricales;f__Cortinariaceae;g__Gymnopilus | 0 | 0 | 0 | 0 | 0 | 2.25E-05 | 0 | 0 | 2.25E-05 | 0 | 2.25E-05 | 0 |
| d__Eukaryota;k__Fungi;p__Basidiomycota;c__Agaricomycetes;o__Boletales;f__Gyrodontaceae;g__Gyrodon | 0 | 0 | 0 | 0 | 0 | 0 | 0 | 4.50E-05 | 0 | 2.25E-05 | 0 | 0 |
| d__Eukaryota;k__Fungi;p__Basidiomycota;c__Tremellomycetes;o__Cystofilobasidiales;f__Mrakiaceae;g__Itersonilia | 0 | 2.25E-05 | 0 | 2.25E-05 | 0 | 2.25E-05 | 0 | 0 | 0 | 0 | 0 | 0 |
| d__Eukaryota;k__Fungi;p__Basidiomycota;c__Tremellomycetes;o__Tremellales;f__Bulleraceae;g__Bullera | 0 | 4.50E-05 | 0 | 0 | 2.25E-05 | 0 | 0 | 0 | 0 | 0 | 0 | 0 |
| d__Eukaryota;k__Fungi;p__Basidiomycota;c__Tremellomycetes;o__Tremellales;f__Tremellaceae;g__Cryptococcus_f__Tremellaceae | 0 | 0 | 0 | 0 | 2.25E-05 | 0 | 0 | 0 | 4.50E-05 | 0 | 0 | 0 |
| d__Eukaryota;k__Fungi;p__Ascomycota;c__Dothideomycetes;o__Botryosphaeriales;f__Botryosphaeriaceae;g__Sardiniella | 0 | 0 | 0 | 6.75E-05 | 0 | 0 | 0 | 0 | 0 | 0 | 0 | 0 |
| d__Eukaryota;k__Fungi;p__Ascomycota;c__Dothideomycetes;o__Capnodiales;f__Mycosphaerellaceae;g__Mycodiella | 0 | 0 | 0 | 6.75E-05 | 0 | 0 | 0 | 0 | 0 | 0 | 0 | 0 |
| d__Eukaryota;k__Fungi;p__Ascomycota;c__Dothideomycetes;o__Capnodiales;f__Neodevriesiaceae;g__Neodevriesia | 0 | 0 | 0 | 6.75E-05 | 0 | 0 | 0 | 0 | 0 | 0 | 0 | 0 |
| d__Eukaryota;k__Fungi;p__Ascomycota;c__Dothideomycetes;o__Pleosporales;f__Didymellaceae;g__Neoascochyta | 0 | 0 | 0 | 0 | 0 | 0 | 6.75E-05 | 0 | 0 | 0 | 0 | 0 |
| d__Eukaryota;k__Fungi;p__Ascomycota;c__Dothideomycetes;o__Pleosporales;f__Didymosphaeriaceae;g__Montagnula | 0 | 0 | 0 | 0 | 0 | 0 | 6.75E-05 | 0 | 0 | 0 | 0 | 0 |
| d__Eukaryota;k__Fungi;p__Ascomycota;c__Eurotiomycetes;o__Onygenales;f__Onygenaceae;g__Auxarthron | 6.75E-05 | 0 | 0 | 0 | 0 | 0 | 0 | 0 | 0 | 0 | 0 | 0 |
| d__Eukaryota;k__Fungi;p__Ascomycota;c__Eurotiomycetes;o__Sclerococcales;f__Dactylosporaceae;g__Dactylospora | 0 | 0 | 0 | 0 | 0 | 0 | 0 | 0 | 0 | 0 | 6.75E-05 | 0 |
| d__Eukaryota;k__Fungi;p__Ascomycota;c__Leotiomycetes;o__Helotiales;f__Dermateaceae;g__Dermea | 2.25E-05 | 0 | 4.50E-05 | 0 | 0 | 0 | 0 | 0 | 0 | 0 | 0 | 0 |
| d__Eukaryota;k__Fungi;p__Ascomycota;c__Leotiomycetes;o__Helotiales;f__Hyaloscyphaceae;g__Lachnum | 0 | 0 | 0 | 6.75E-05 | 0 | 0 | 0 | 0 | 0 | 0 | 0 | 0 |
| d__Eukaryota;k__Fungi;p__Ascomycota;c__Leotiomycetes;o__Helotiales;f__Sclerotiniaceae;g__Sclerotinia | 0 | 0 | 0 | 6.75E-05 | 0 | 0 | 0 | 0 | 0 | 0 | 0 | 0 |
| d__Eukaryota;k__Fungi;p__Ascomycota;c__Pezizomycetes;o__Pezizales;f__Pyronemataceae;g__Lasiobolidium | 0 | 0 | 0 | 0 | 0 | 0 | 0 | 0 | 0 | 0 | 6.75E-05 | 0 |
| d__Eukaryota;k__Fungi;p__Ascomycota;c__Saccharomycetes;o__Saccharomycetales;f__Saccharomycetaceae;g__Issatchenkia | 0 | 0 | 0 | 0 | 0 | 0 | 0 | 6.75E-05 | 0 | 0 | 0 | 0 |
| d__Eukaryota;k__Fungi;p__Ascomycota;c__Sordariomycetes;o__Chaetosphaeriales;f__Chaetosphaeriaceae;g__Codinaea | 0 | 0 | 0 | 0 | 0 | 6.75E-05 | 0 | 0 | 0 | 0 | 0 | 0 |
| d__Eukaryota;k__Fungi;p__Ascomycota;c__Sordariomycetes;o__Xylariales;f__Amphisphaeriaceae;g__Monochaetia | 0 | 0 | 0 | 0 | 0 | 6.75E-05 | 0 | 0 | 0 | 0 | 0 | 0 |
| d__Eukaryota;k__Fungi;p__Ascomycota;c__Sordariomycetes;o__Xylariales;f__Xylariaceae;g__unclassified_f__Xylariaceae | 0 | 0 | 0 | 0 | 4.50E-05 | 2.25E-05 | 0 | 0 | 0 | 0 | 0 | 0 |
| d__Eukaryota;k__Fungi;p__Basidiomycota;c__Agaricomycetes;o__Agaricales;f__Bolbitiaceae;g__Pholiotina | 0 | 0 | 0 | 0 | 6.75E-05 | 0 | 0 | 0 | 0 | 0 | 0 | 0 |
| d__Eukaryota;k__Fungi;p__Basidiomycota;c__Agaricomycetes;o__Agaricales;f__Lycoperdaceae;g__unclassified_f__Lycoperdaceae | 0 | 0 | 0 | 0 | 0 | 0 | 0 | 2.25E-05 | 4.50E-05 | 0 | 0 | 0 |
| d__Eukaryota;k__Fungi;p__Basidiomycota;c__Agaricomycetes;o__Cantharellales;f__unclassified_o__Cantharellales;g__unclassified_o__Cantharellales | 6.75E-05 | 0 | 0 | 0 | 0 | 0 | 0 | 0 | 0 | 0 | 0 | 0 |
| d__Eukaryota;k__Fungi;p__Basidiomycota;c__Agaricomycetes;o__Hymenochaetales;f__Hymenochaetaceae;g__Fulvifomes | 0 | 0 | 0 | 0 | 6.75E-05 | 0 | 0 | 0 | 0 | 0 | 0 | 0 |
| d__Eukaryota;k__Fungi;p__Basidiomycota;c__Agaricomycetes;o__Hymenochaetales;f__Hymenochaetaceae;g__Phellinus | 0 | 0 | 0 | 0 | 4.50E-05 | 2.25E-05 | 0 | 0 | 0 | 0 | 0 | 0 |
| d__Eukaryota;k__Fungi;p__Basidiomycota;c__Agaricomycetes;o__Hymenochaetales;f__Hymenochaetales_fam_Incertae_sedis;g__Peniophorella | 0 | 0 | 0 | 0 | 6.75E-05 | 0 | 0 | 0 | 0 | 0 | 0 | 0 |
| d__Eukaryota;k__Fungi;p__Basidiomycota;c__Agaricomycetes;o__Polyporales;f__Meruliaceae;g__Phlebiopsis | 0 | 0 | 0 | 6.75E-05 | 0 | 0 | 0 | 0 | 0 | 0 | 0 | 0 |
| d__Eukaryota;k__Fungi;p__Basidiomycota;c__Agaricomycetes;o__Polyporales;f__Polyporaceae;g__Polyporus | 0 | 0 | 6.75E-05 | 0 | 0 | 0 | 0 | 0 | 0 | 0 | 0 | 0 |
| d__Eukaryota;k__Fungi;p__Basidiomycota;c__Agaricomycetes;o__Russulales;f__Gloeocystidiellaceae;g__Gloeocystidiellum | 0 | 0 | 0 | 0 | 0 | 6.75E-05 | 0 | 0 | 0 | 0 | 0 | 0 |
| d__Eukaryota;k__Fungi;p__Basidiomycota;c__Cystobasidiomycetes;o__Cystobasidiales;f__Cystobasidiaceae;g__Cystobasidium | 0 | 0 | 0 | 0 | 0 | 6.75E-05 | 0 | 0 | 0 | 0 | 0 | 0 |
| d__Eukaryota;k__Fungi;p__Basidiomycota;c__Cystobasidiomycetes;o__Naohideales;f__unclassified_o__Naohideales;g__unclassified_o__Naohideales | 0 | 0 | 6.75E-05 | 0 | 0 | 0 | 0 | 0 | 0 | 0 | 0 | 0 |
| d__Eukaryota;k__Fungi;p__Basidiomycota;c__Exobasidiomycetes;o__Entylomatales;f__Entylomatales_fam_Incertae_sedis;g__Tilletiopsis | 6.75E-05 | 0 | 0 | 0 | 0 | 0 | 0 | 0 | 0 | 0 | 0 | 0 |
| d__Eukaryota;k__Fungi;p__Basidiomycota;c__Tremellomycetes;o__Cystofilobasidiales;f__Mrakiaceae;g__Udeniomyces | 6.75E-05 | 0 | 0 | 0 | 0 | 0 | 0 | 0 | 0 | 0 | 0 | 0 |
| d__Eukaryota;k__Fungi;p__Chytridiomycota;c__Synchytriomycetes;o__Synchytriales;f__Synchytriaceae;g__Synchytrium | 0 | 2.25E-05 | 4.50E-05 | 0 | 0 | 0 | 0 | 0 | 0 | 0 | 0 | 0 |
| d__Eukaryota;k__Fungi;p__Glomeromycota;c__Glomeromycetes;o__Diversisporales;f__Acaulosporaceae;g__unclassified_f__Acaulosporaceae | 0 | 0 | 0 | 0 | 6.75E-05 | 0 | 0 | 0 | 0 | 0 | 0 | 0 |
| d__Eukaryota;k__Fungi;p__Kickxellomycota;c__unclassified_p__Kickxellomycota;o__unclassified_p__Kickxellomycota;f__unclassified_p__Kickxellomycota;g__unclassified_p__Kickxellomycota | 0 | 0 | 0 | 0 | 0 | 0 | 6.75E-05 | 0 | 0 | 0 | 0 | 0 |
| d__Eukaryota;k__Fungi;p__Monoblepharomycota;c__Monoblepharidomycetes;o__unclassified_c__Monoblepharidomycetes;f__unclassified_c__Monoblepharidomycetes;g__unclassified_c__Monoblepharidomycetes | 0 | 0 | 0 | 6.75E-05 | 0 | 0 | 0 | 0 | 0 | 0 | 0 | 0 |
| d__Eukaryota;k__Fungi;p__Rozellomycota;c__Rozellomycotina_cls_Incertae_sedis;o__GS08;f__unclassified_o__GS08;g__unclassified_o__GS08 | 2.25E-05 | 2.25E-05 | 2.25E-05 | 0 | 0 | 0 | 0 | 0 | 0 | 0 | 0 | 0 |
| d__Eukaryota;k__Fungi;p__Ascomycota;c__Dothideomycetes;o__Pleosporales;f__Leptosphaeriaceae;g__Plenodomus | 0 | 0 | 0 | 2.25E-05 | 0 | 0 | 0 | 2.25E-05 | 0 | 0 | 0 | 0 |
| d__Eukaryota;k__Fungi;p__Ascomycota;c__Pezizomycetes;o__Pezizales;f__Ascodesmidaceae;g__Cephaliophora | 0 | 0 | 0 | 0 | 0 | 0 | 0 | 2.25E-05 | 0 | 0 | 2.25E-05 | 0 |
| d__Eukaryota;k__Fungi;p__Ascomycota;c__Sordariomycetes;o__Melanosporales;f__unclassified_o__Melanosporales;g__unclassified_o__Melanosporales | 2.25E-05 | 0 | 0 | 0 | 0 | 0 | 0 | 0 | 2.25E-05 | 0 | 0 | 0 |
| d__Eukaryota;k__Fungi;p__Basidiomycota;c__Agaricomycetes;o__Agaricales;f__Crepidotaceae;g__Crepidotus | 0 | 0 | 0 | 2.25E-05 | 0 | 0 | 2.25E-05 | 0 | 0 | 0 | 0 | 0 |
| d__Eukaryota;k__Fungi;p__Basidiomycota;c__Agaricomycetes;o__Agaricales;f__Strophariaceae;g__Stropharia | 0 | 0 | 0 | 0 | 0 | 0 | 0 | 2.25E-05 | 0 | 2.25E-05 | 0 | 0 |
| d__Eukaryota;k__Fungi;p__Basidiomycota;c__Agaricomycetes;o__Agaricales;f__Strophariaceae;g__unclassified_f__Strophariaceae | 0 | 0 | 0 | 0 | 2.25E-05 | 0 | 0 | 0 | 2.25E-05 | 0 | 0 | 0 |
| d__Eukaryota;k__Fungi;p__Basidiomycota;c__Agaricomycetes;o__Cantharellales;f__Ceratobasidiaceae;g__Rhizoctonia | 0 | 0 | 0 | 0 | 0 | 0 | 0 | 0 | 2.25E-05 | 0 | 0 | 2.25E-05 |
| d__Eukaryota;k__Fungi;p__Basidiomycota;c__Agaricomycetes;o__Polyporales;f__Meruliaceae;g__Phaeophlebiopsis | 0 | 0 | 0 | 0 | 0 | 2.25E-05 | 0 | 0 | 2.25E-05 | 0 | 0 | 0 |
| d__Eukaryota;k__Fungi;p__Basidiomycota;c__Tremellomycetes;o__Filobasidiales;f__Filobasidiaceae;g__Goffeauzyma | 0 | 0 | 0 | 0 | 0 | 0 | 0 | 0 | 2.25E-05 | 0 | 2.25E-05 | 0 |
| d__Eukaryota;k__Fungi;p__Basidiomycota;c__Tremellomycetes;o__Trichosporonales;f__Trichosporonaceae;g__Trichosporon | 0 | 0 | 0 | 0 | 0 | 2.25E-05 | 0 | 0 | 2.25E-05 | 0 | 0 | 0 |
| d__Eukaryota;k__Fungi;p__Mucoromycota;c__Mucoromycetes;o__Mucorales;f__Mucoraceae;g__Mucor | 0 | 2.25E-05 | 0 | 0 | 0 | 2.25E-05 | 0 | 0 | 0 | 0 | 0 | 0 |
| d__Eukaryota;k__Fungi;p__Ascomycota;c__Dothideomycetes;o__Pleosporales;f__Didymosphaeriaceae;g__Paraconiothyrium | 0 | 0 | 0 | 0 | 4.50E-05 | 0 | 0 | 0 | 0 | 0 | 0 | 0 |
| d__Eukaryota;k__Fungi;p__Ascomycota;c__Dothideomycetes;o__Pleosporales;f__Didymosphaeriaceae;g__unclassified_f__Didymosphaeriaceae | 0 | 0 | 0 | 0 | 0 | 4.50E-05 | 0 | 0 | 0 | 0 | 0 | 0 |
| d__Eukaryota;k__Fungi;p__Ascomycota;c__Dothideomycetes;o__Pleosporales;f__Morosphaeriaceae;g__Acrocalymma | 0 | 0 | 0 | 4.50E-05 | 0 | 0 | 0 | 0 | 0 | 0 | 0 | 0 |
| d__Eukaryota;k__Fungi;p__Ascomycota;c__Dothideomycetes;o__Pleosporales;f__Phaeosphaeriaceae;g__Neosetophoma | 0 | 0 | 0 | 0 | 0 | 4.50E-05 | 0 | 0 | 0 | 0 | 0 | 0 |
| d__Eukaryota;k__Fungi;p__Ascomycota;c__Dothideomycetes;o__Pleosporales;f__Phaeosphaeriaceae;g__Setomelanomma | 0 | 0 | 0 | 0 | 0 | 4.50E-05 | 0 | 0 | 0 | 0 | 0 | 0 |
| d__Eukaryota;k__Fungi;p__Ascomycota;c__Dothideomycetes;o__Pleosporales;f__Pleosporales_fam_Incertae_sedis;g__Pseudorobillarda | 0 | 0 | 0 | 0 | 0 | 0 | 0 | 4.50E-05 | 0 | 0 | 0 | 0 |
| d__Eukaryota;k__Fungi;p__Ascomycota;c__Dothideomycetes;o__Pleosporales;f__Tetraplosphaeriaceae;g__Tetraplosphaeria | 0 | 0 | 0 | 0 | 0 | 0 | 0 | 0 | 4.50E-05 | 0 | 0 | 0 |
| d__Eukaryota;k__Fungi;p__Ascomycota;c__Dothideomycetes;o__Pleosporales;f__Thyridariaceae;g__Roussoella | 0 | 0 | 0 | 0 | 0 | 0 | 2.25E-05 | 2.25E-05 | 0 | 0 | 0 | 0 |
| d__Eukaryota;k__Fungi;p__Ascomycota;c__Eurotiomycetes;o__Chaetothyriales;f__Chaetothyriales_fam_Incertae_sedis;g__Strelitziana | 0 | 0 | 0 | 0 | 4.50E-05 | 0 | 0 | 0 | 0 | 0 | 0 | 0 |
| d__Eukaryota;k__Fungi;p__Ascomycota;c__Lecanoromycetes;o__Acarosporales;f__Acarosporaceae;g__Sarcogyne | 0 | 0 | 0 | 0 | 0 | 0 | 0 | 0 | 0 | 4.50E-05 | 0 | 0 |
| d__Eukaryota;k__Fungi;p__Ascomycota;c__Lecanoromycetes;o__Acarosporales;f__Acarosporaceae;g__unclassified_f__Acarosporaceae | 0 | 0 | 0 | 0 | 0 | 4.50E-05 | 0 | 0 | 0 | 0 | 0 | 0 |
| d__Eukaryota;k__Fungi;p__Ascomycota;c__Lecanoromycetes;o__Caliciales;f__Physciaceae;g__Physcia | 0 | 0 | 0 | 0 | 0 | 4.50E-05 | 0 | 0 | 0 | 0 | 0 | 0 |
| d__Eukaryota;k__Fungi;p__Ascomycota;c__Leotiomycetes;o__Helotiales;f__Leotiaceae;g__Alatospora | 0 | 4.50E-05 | 0 | 0 | 0 | 0 | 0 | 0 | 0 | 0 | 0 | 0 |
| d__Eukaryota;k__Fungi;p__Ascomycota;c__Leotiomycetes;o__Helotiales;f__Sclerotiniaceae;g__unclassified_f__Sclerotiniaceae | 0 | 0 | 0 | 0 | 0 | 0 | 4.50E-05 | 0 | 0 | 0 | 0 | 0 |
| d__Eukaryota;k__Fungi;p__Ascomycota;c__Leotiomycetes;o__Thelebolales;f__Pseudeurotiaceae;g__Geomyces | 0 | 0 | 0 | 0 | 0 | 0 | 0 | 0 | 4.50E-05 | 0 | 0 | 0 |
| d__Eukaryota;k__Fungi;p__Ascomycota;c__Orbiliomycetes;o__Orbiliales;f__Orbiliaceae;g__Orbilia | 0 | 0 | 0 | 0 | 0 | 0 | 0 | 0 | 4.50E-05 | 0 | 0 | 0 |
| d__Eukaryota;k__Fungi;p__Ascomycota;c__Pezizomycetes;o__Pezizales;f__Pyronemataceae;g__Kotlabaea | 0 | 2.25E-05 | 2.25E-05 | 0 | 0 | 0 | 0 | 0 | 0 | 0 | 0 | 0 |
| d__Eukaryota;k__Fungi;p__Ascomycota;c__Sordariomycetes;o__Branch06;f__unclassified_o__Branch06;g__unclassified_o__Branch06 | 0 | 0 | 0 | 0 | 0 | 0 | 4.50E-05 | 0 | 0 | 0 | 0 | 0 |
| d__Eukaryota;k__Fungi;p__Ascomycota;c__Sordariomycetes;o__Glomerellales;f__Plectosphaerellaceae;g__Musicillium | 0 | 0 | 0 | 0 | 0 | 0 | 0 | 0 | 0 | 0 | 4.50E-05 | 0 |
| d__Eukaryota;k__Fungi;p__Ascomycota;c__Sordariomycetes;o__Hypocreales;f__Clavicipitaceae;g__Pochonia | 0 | 0 | 0 | 4.50E-05 | 0 | 0 | 0 | 0 | 0 | 0 | 0 | 0 |
| d__Eukaryota;k__Fungi;p__Ascomycota;c__Sordariomycetes;o__Hypocreales;f__Hypocreales_fam_Incertae_sedis;g__Chlamydomyces | 0 | 0 | 0 | 0 | 0 | 0 | 0 | 0 | 4.50E-05 | 0 | 0 | 0 |
| d__Eukaryota;k__Fungi;p__Ascomycota;c__Sordariomycetes;o__Hypocreales;f__Nectriaceae;g__Stylonectria | 0 | 0 | 0 | 0 | 0 | 0 | 0 | 0 | 0 | 0 | 4.50E-05 | 0 |
| d__Eukaryota;k__Fungi;p__Ascomycota;c__Sordariomycetes;o__Hypocreales;f__Ophiocordycipitaceae;g__Harposporium | 0 | 0 | 0 | 0 | 4.50E-05 | 0 | 0 | 0 | 0 | 0 | 0 | 0 |
| d__Eukaryota;k__Fungi;p__Ascomycota;c__Sordariomycetes;o__Hypocreales;f__Stachybotryaceae;g__Myxospora | 0 | 4.50E-05 | 0 | 0 | 0 | 0 | 0 | 0 | 0 | 0 | 0 | 0 |
| d__Eukaryota;k__Fungi;p__Ascomycota;c__Sordariomycetes;o__Sordariales;f__Sordariales_fam_Incertae_sedis;g__Cordana | 0 | 0 | 0 | 4.50E-05 | 0 | 0 | 0 | 0 | 0 | 0 | 0 | 0 |
| d__Eukaryota;k__Fungi;p__Ascomycota;c__Sordariomycetes;o__Xylariales;f__Amphisphaeriaceae;g__unclassified_f__Amphisphaeriaceae | 0 | 0 | 0 | 0 | 0 | 0 | 0 | 0 | 0 | 0 | 4.50E-05 | 0 |
| d__Eukaryota;k__Fungi;p__Ascomycota;c__Sordariomycetes;o__Xylariales;f__Sporocadaceae;g__Pestalotiopsis | 0 | 0 | 0 | 0 | 4.50E-05 | 0 | 0 | 0 | 0 | 0 | 0 | 0 |
| d__Eukaryota;k__Fungi;p__Basidiomycota;c__Agaricomycetes;o__Agaricales;f__Bolbitiaceae;g__Panaeolus | 0 | 0 | 0 | 0 | 0 | 4.50E-05 | 0 | 0 | 0 | 0 | 0 | 0 |
| d__Eukaryota;k__Fungi;p__Basidiomycota;c__Agaricomycetes;o__Agaricales;f__Lyophyllaceae;g__Tephrocybe | 0 | 0 | 0 | 0 | 0 | 0 | 0 | 0 | 0 | 0 | 4.50E-05 | 0 |
| d__Eukaryota;k__Fungi;p__Basidiomycota;c__Agaricomycetes;o__Boletales;f__Astraeaceae;g__Astraeus | 0 | 0 | 0 | 0 | 0 | 0 | 0 | 4.50E-05 | 0 | 0 | 0 | 0 |
| d__Eukaryota;k__Fungi;p__Basidiomycota;c__Agaricomycetes;o__Boletales;f__Boletaceae;g__Butyriboletus | 0 | 0 | 0 | 0 | 0 | 0 | 0 | 0 | 0 | 0 | 0 | 4.50E-05 |
| d__Eukaryota;k__Fungi;p__Basidiomycota;c__Agaricomycetes;o__Cantharellales;f__Ceratobasidiaceae;g__Thanatephorus | 0 | 4.50E-05 | 0 | 0 | 0 | 0 | 0 | 0 | 0 | 0 | 0 | 0 |
| d__Eukaryota;k__Fungi;p__Basidiomycota;c__Agaricomycetes;o__Corticiales;f__Corticiaceae;g__Limonomyces | 0 | 0 | 0 | 0 | 0 | 4.50E-05 | 0 | 0 | 0 | 0 | 0 | 0 |
| d__Eukaryota;k__Fungi;p__Basidiomycota;c__Agaricomycetes;o__Corticiales;f__unclassified_o__Corticiales;g__unclassified_o__Corticiales | 0 | 0 | 0 | 0 | 0 | 0 | 0 | 0 | 0 | 4.50E-05 | 0 | 0 |
| d__Eukaryota;k__Fungi;p__Basidiomycota;c__Agaricomycetes;o__Hymenochaetales;f__Hymenochaetaceae;g__unclassified_f__Hymenochaetaceae | 0 | 0 | 0 | 4.50E-05 | 0 | 0 | 0 | 0 | 0 | 0 | 0 | 0 |
| d__Eukaryota;k__Fungi;p__Basidiomycota;c__Agaricomycetes;o__Hymenochaetales;f__Tubulicrinaceae;g__Tubulicrinis | 0 | 0 | 0 | 0 | 0 | 0 | 4.50E-05 | 0 | 0 | 0 | 0 | 0 |
| d__Eukaryota;k__Fungi;p__Basidiomycota;c__Agaricomycetes;o__Polyporales;f__Fomitopsidaceae;g__Frantisekia | 0 | 0 | 0 | 0 | 0 | 0 | 0 | 4.50E-05 | 0 | 0 | 0 | 0 |
| d__Eukaryota;k__Fungi;p__Basidiomycota;c__Agaricomycetes;o__Polyporales;f__Meruliaceae;g__Rhizochaete | 0 | 0 | 0 | 0 | 0 | 0 | 0 | 0 | 0 | 0 | 4.50E-05 | 0 |
| d__Eukaryota;k__Fungi;p__Basidiomycota;c__Cystobasidiomycetes;o__Cystobasidiomycetes_ord_Incertae_sedis;f__Buckleyzymaceae;g__Buckleyzyma | 0 | 2.25E-05 | 2.25E-05 | 0 | 0 | 0 | 0 | 0 | 0 | 0 | 0 | 0 |
| d__Eukaryota;k__Fungi;p__Basidiomycota;c__Cystobasidiomycetes;o__Erythrobasidiales;f__Erythrobasidiaceae;g__Erythrobasidium | 0 | 0 | 0 | 0 | 0 | 0 | 0 | 0 | 0 | 0 | 4.50E-05 | 0 |
| d__Eukaryota;k__Fungi;p__Basidiomycota;c__Tremellomycetes;o__Trichosporonales;f__Trichosporonaceae;g__Vanrija | 0 | 0 | 0 | 0 | 0 | 0 | 0 | 4.50E-05 | 0 | 0 | 0 | 0 |
| d__Eukaryota;k__Fungi;p__Chytridiomycota;c__Chytridiomycetes;o__Chytridiales;f__Chytridiaceae;g__unclassified_f__Chytridiaceae | 0 | 4.50E-05 | 0 | 0 | 0 | 0 | 0 | 0 | 0 | 0 | 0 | 0 |
| d__Eukaryota;k__Fungi;p__Chytridiomycota;c__Rhizophydiomycetes;o__Rhizophydiales;f__Halomycetaceae;g__Paranamyces | 0 | 0 | 4.50E-05 | 0 | 0 | 0 | 0 | 0 | 0 | 0 | 0 | 0 |
| d__Eukaryota;k__Fungi;p__Chytridiomycota;c__Spizellomycetes;o__Spizellomycetales;f__Powellomycetaceae;g__Powellomyces | 0 | 0 | 0 | 0 | 0 | 0 | 0 | 0 | 4.50E-05 | 0 | 0 | 0 |
| d__Eukaryota;k__Fungi;p__Glomeromycota;c__Paraglomeromycetes;o__Paraglomerales;f__unclassified_o__Paraglomerales;g__unclassified_o__Paraglomerales | 0 | 0 | 0 | 0 | 0 | 0 | 0 | 4.50E-05 | 0 | 0 | 0 | 0 |
| d__Eukaryota;k__Fungi;p__Monoblepharomycota;c__Hyaloraphidiomycetes;o__Hyaloraphidiales;f__Hyaloraphidiales_fam_Incertae_sedis;g__Hyaloraphidium | 0 | 0 | 0 | 0 | 0 | 0 | 0 | 4.50E-05 | 0 | 0 | 0 | 0 |
| d__Eukaryota;k__Fungi;p__Mucoromycota;c__Endogonomycetes;o__GS21;f__unclassified_o__GS21;g__unclassified_o__GS21 | 0 | 0 | 0 | 4.50E-05 | 0 | 0 | 0 | 0 | 0 | 0 | 0 | 0 |
| d__Eukaryota;k__Fungi;p__Aphelidiomycota;c__unclassified_p__Aphelidiomycota;o__unclassified_p__Aphelidiomycota;f__unclassified_p__Aphelidiomycota;g__unclassified_p__Aphelidiomycota | 0 | 0 | 0 | 2.25E-05 | 0 | 0 | 0 | 0 | 0 | 0 | 0 | 0 |
| d__Eukaryota;k__Fungi;p__Ascomycota;c__Dothideomycetes;o__Capnodiales;f__Capnodiaceae;g__Antennariella | 0 | 0 | 0 | 0 | 0 | 0 | 0 | 0 | 0 | 0 | 0 | 2.25E-05 |
| d__Eukaryota;k__Fungi;p__Ascomycota;c__Dothideomycetes;o__Mytilinidales;f__Kirschsteiniotheliaceae;g__Taeniolella | 0 | 2.25E-05 | 0 | 0 | 0 | 0 | 0 | 0 | 0 | 0 | 0 | 0 |
| d__Eukaryota;k__Fungi;p__Ascomycota;c__Dothideomycetes;o__Pleosporales;f__Didymellaceae;g__Paraboeremia | 0 | 0 | 0 | 0 | 0 | 0 | 0 | 0 | 0 | 2.25E-05 | 0 | 0 |
| d__Eukaryota;k__Fungi;p__Ascomycota;c__Dothideomycetes;o__Pleosporales;f__Lophiostomataceae;g__unclassified_f__Lophiostomataceae | 0 | 0 | 0 | 0 | 0 | 0 | 2.25E-05 | 0 | 0 | 0 | 0 | 0 |
| d__Eukaryota;k__Fungi;p__Ascomycota;c__Dothideomycetes;o__Pleosporales;f__Montagnulaceae;g__unclassified_f__Montagnulaceae | 0 | 0 | 2.25E-05 | 0 | 0 | 0 | 0 | 0 | 0 | 0 | 0 | 0 |
| d__Eukaryota;k__Fungi;p__Ascomycota;c__Dothideomycetes;o__Pleosporales;f__Phaeosphaeriaceae;g__Phaeosphaeriopsis | 0 | 0 | 0 | 0 | 0 | 0 | 2.25E-05 | 0 | 0 | 0 | 0 | 0 |
| d__Eukaryota;k__Fungi;p__Ascomycota;c__Dothideomycetes;o__Pleosporales;f__Thyridariaceae;g__Parathyridaria | 0 | 0 | 0 | 0 | 2.25E-05 | 0 | 0 | 0 | 0 | 0 | 0 | 0 |
| d__Eukaryota;k__Fungi;p__Ascomycota;c__Dothideomycetes;o__Tubeufiales;f__Tubeufiaceae;g__unclassified_f__Tubeufiaceae | 0 | 0 | 0 | 0 | 0 | 0 | 0 | 0 | 2.25E-05 | 0 | 0 | 0 |
| d__Eukaryota;k__Fungi;p__Ascomycota;c__Dothideomycetes;o__Valsariales;f__Valsariaceae;g__Myrmaecium | 0 | 0 | 0 | 0 | 0 | 0 | 2.25E-05 | 0 | 0 | 0 | 0 | 0 |
| d__Eukaryota;k__Fungi;p__Ascomycota;c__Dothideomycetes;o__Venturiales;f__Venturiaceae;g__Venturia | 0 | 0 | 0 | 2.25E-05 | 0 | 0 | 0 | 0 | 0 | 0 | 0 | 0 |
| d__Eukaryota;k__Fungi;p__Ascomycota;c__Eurotiomycetes;o__Chaetothyriales;f__Herpotrichiellaceae;g__Melanchlenus | 0 | 0 | 0 | 0 | 0 | 0 | 0 | 0 | 2.25E-05 | 0 | 0 | 0 |
| d__Eukaryota;k__Fungi;p__Ascomycota;c__Eurotiomycetes;o__Chaetothyriales;f__Trichomeriaceae;g__Knufia | 0 | 0 | 0 | 0 | 0 | 0 | 0 | 0 | 2.25E-05 | 0 | 0 | 0 |
| d__Eukaryota;k__Fungi;p__Ascomycota;c__Eurotiomycetes;o__Eurotiales;f__Aspergillaceae;g__Hamigera | 0 | 0 | 0 | 0 | 0 | 0 | 0 | 0 | 0 | 0 | 2.25E-05 | 0 |
| d__Eukaryota;k__Fungi;p__Ascomycota;c__Eurotiomycetes;o__Eurotiales;f__Thermoascaceae;g__Thermoascus | 0 | 0 | 0 | 0 | 0 | 0 | 0 | 0 | 0 | 0 | 0 | 2.25E-05 |
| d__Eukaryota;k__Fungi;p__Ascomycota;c__Eurotiomycetes;o__Eurotiales;f__Trichocomaceae;g__Rasamsonia | 0 | 0 | 0 | 0 | 0 | 0 | 0 | 2.25E-05 | 0 | 0 | 0 | 0 |
| d__Eukaryota;k__Fungi;p__Ascomycota;c__Eurotiomycetes;o__Onygenales;f__Arthrodermataceae;g__Trichophyton | 2.25E-05 | 0 | 0 | 0 | 0 | 0 | 0 | 0 | 0 | 0 | 0 | 0 |
| d__Eukaryota;k__Fungi;p__Ascomycota;c__Eurotiomycetes;o__Onygenales;f__Gymnoascaceae;g__Arachniotus | 0 | 0 | 0 | 0 | 0 | 0 | 2.25E-05 | 0 | 0 | 0 | 0 | 0 |
| d__Eukaryota;k__Fungi;p__Ascomycota;c__Laboulbeniomycetes;o__Pyxidiophorales;f__Pyxidiophoraceae;g__Pyxidiophora | 0 | 0 | 0 | 0 | 0 | 0 | 0 | 0 | 2.25E-05 | 0 | 0 | 0 |
| d__Eukaryota;k__Fungi;p__Ascomycota;c__Leotiomycetes;o__Erysiphales;f__Erysiphaceae;g__Golovinomyces | 0 | 0 | 0 | 0 | 0 | 0 | 0 | 0 | 2.25E-05 | 0 | 0 | 0 |
| d__Eukaryota;k__Fungi;p__Ascomycota;c__Leotiomycetes;o__Helotiales;f__Dermateaceae;g__Neofabraea | 0 | 0 | 2.25E-05 | 0 | 0 | 0 | 0 | 0 | 0 | 0 | 0 | 0 |
| d__Eukaryota;k__Fungi;p__Ascomycota;c__Leotiomycetes;o__Helotiales;f__Dermateaceae;g__Patinella | 2.25E-05 | 0 | 0 | 0 | 0 | 0 | 0 | 0 | 0 | 0 | 0 | 0 |
| d__Eukaryota;k__Fungi;p__Ascomycota;c__Leotiomycetes;o__Helotiales;f__Helotiales_fam_Incertae_sedis;g__Acephala | 0 | 0 | 0 | 0 | 0 | 0 | 0 | 2.25E-05 | 0 | 0 | 0 | 0 |
| d__Eukaryota;k__Fungi;p__Ascomycota;c__Leotiomycetes;o__Helotiales;f__Helotiales_fam_Incertae_sedis;g__unclassified_f__Helotiales_fam_Incertae_sedis | 0 | 0 | 0 | 0 | 0 | 0 | 2.25E-05 | 0 | 0 | 0 | 0 | 0 |
| d__Eukaryota;k__Fungi;p__Ascomycota;c__Leotiomycetes;o__Helotiales;f__Sclerotiniaceae;g__Scleromitrula | 0 | 0 | 0 | 0 | 2.25E-05 | 0 | 0 | 0 | 0 | 0 | 0 | 0 |
| d__Eukaryota;k__Fungi;p__Ascomycota;c__Leotiomycetes;o__Phacidiales;f__Bulgariaceae;g__Bulgaria | 0 | 0 | 0 | 0 | 0 | 2.25E-05 | 0 | 0 | 0 | 0 | 0 | 0 |
| d__Eukaryota;k__Fungi;p__Ascomycota;c__Pezizomycetes;o__Pezizales;f__Pezizaceae;g__Pachyella | 0 | 0 | 0 | 2.25E-05 | 0 | 0 | 0 | 0 | 0 | 0 | 0 | 0 |
| d__Eukaryota;k__Fungi;p__Ascomycota;c__Sordariomycetes;o__Boliniales;f__Boliniaceae;g__unclassified_f__Boliniaceae | 0 | 0 | 0 | 0 | 0 | 0 | 0 | 0 | 2.25E-05 | 0 | 0 | 0 |
| d__Eukaryota;k__Fungi;p__Ascomycota;c__Sordariomycetes;o__Diaporthales;f__Sydowiellaceae;g__Breviappendix | 0 | 0 | 0 | 0 | 0 | 0 | 0 | 2.25E-05 | 0 | 0 | 0 | 0 |
| d__Eukaryota;k__Fungi;p__Ascomycota;c__Sordariomycetes;o__Hypocreales;f__Hypocreales_fam_Incertae_sedis;g__Fusariella | 0 | 0 | 0 | 0 | 0 | 0 | 0 | 0 | 0 | 0 | 2.25E-05 | 0 |
| d__Eukaryota;k__Fungi;p__Ascomycota;c__Sordariomycetes;o__Hypocreales;f__Hypocreales_fam_Incertae_sedis;g__Ustilaginoidea | 0 | 0 | 0 | 0 | 0 | 0 | 0 | 0 | 0 | 2.25E-05 | 0 | 0 |
| d__Eukaryota;k__Fungi;p__Ascomycota;c__Sordariomycetes;o__Hypocreales;f__Nectriaceae;g__Haematonectria | 0 | 0 | 0 | 0 | 0 | 0 | 0 | 0 | 2.25E-05 | 0 | 0 | 0 |
| d__Eukaryota;k__Fungi;p__Ascomycota;c__Sordariomycetes;o__Hypocreales;f__Nectriaceae;g__Nectria | 0 | 0 | 2.25E-05 | 0 | 0 | 0 | 0 | 0 | 0 | 0 | 0 | 0 |
| d__Eukaryota;k__Fungi;p__Ascomycota;c__Sordariomycetes;o__Hypocreales;f__Ophiocordycipitaceae;g__Ophiocordyceps | 0 | 0 | 2.25E-05 | 0 | 0 | 0 | 0 | 0 | 0 | 0 | 0 | 0 |
| d__Eukaryota;k__Fungi;p__Ascomycota;c__Sordariomycetes;o__Microascales;f__Microascales_fam_Incertae_sedis;g__unclassified_f__Microascales_fam_Incertae_sedis | 0 | 0 | 0 | 0 | 0 | 0 | 0 | 2.25E-05 | 0 | 0 | 0 | 0 |
| d__Eukaryota;k__Fungi;p__Ascomycota;c__Sordariomycetes;o__Pleurotheciales;f__Pleurotheciaceae;g__unclassified_f__Pleurotheciaceae | 0 | 0 | 0 | 0 | 0 | 0 | 0 | 0 | 2.25E-05 | 0 | 0 | 0 |
| d__Eukaryota;k__Fungi;p__Ascomycota;c__Sordariomycetes;o__Sordariales;f__Cephalothecaceae;g__Cephalotheca | 0 | 0 | 0 | 0 | 0 | 0 | 2.25E-05 | 0 | 0 | 0 | 0 | 0 |
| d__Eukaryota;k__Fungi;p__Ascomycota;c__Sordariomycetes;o__Xylariales;f__Xylariaceae;g__Annulohypoxylon | 0 | 0 | 0 | 0 | 0 | 0 | 0 | 0 | 0 | 2.25E-05 | 0 | 0 |
| d__Eukaryota;k__Fungi;p__Ascomycota;c__Sordariomycetes;o__Xylariales;f__Xylariaceae;g__Creosphaeria | 0 | 0 | 2.25E-05 | 0 | 0 | 0 | 0 | 0 | 0 | 0 | 0 | 0 |
| d__Eukaryota;k__Fungi;p__Ascomycota;c__Sordariomycetes;o__Xylariales;f__Xylariales_fam_Incertae_sedis;g__Robillarda | 0 | 0 | 0 | 0 | 0 | 0 | 0 | 0 | 2.25E-05 | 0 | 0 | 0 |
| d__Eukaryota;k__Fungi;p__Basidiomycota;c__Agaricomycetes;o__Agaricales;f__Chromocyphellaceae;g__unclassified_f__Chromocyphellaceae | 0 | 0 | 0 | 0 | 0 | 0 | 0 | 2.25E-05 | 0 | 0 | 0 | 0 |
| d__Eukaryota;k__Fungi;p__Basidiomycota;c__Agaricomycetes;o__Agaricales;f__Crepidotaceae;g__Simocybe | 0 | 0 | 0 | 0 | 0 | 0 | 0 | 0 | 0 | 0 | 2.25E-05 | 0 |
| d__Eukaryota;k__Fungi;p__Basidiomycota;c__Agaricomycetes;o__Agaricales;f__Hymenogastraceae;g__Hebeloma | 0 | 0 | 0 | 0 | 0 | 0 | 0 | 2.25E-05 | 0 | 0 | 0 | 0 |
| d__Eukaryota;k__Fungi;p__Basidiomycota;c__Agaricomycetes;o__Agaricales;f__Lycoperdaceae;g__Bovista | 0 | 2.25E-05 | 0 | 0 | 0 | 0 | 0 | 0 | 0 | 0 | 0 | 0 |
| d__Eukaryota;k__Fungi;p__Basidiomycota;c__Agaricomycetes;o__Agaricales;f__Physalacriaceae;g__Flammulina | 0 | 0 | 0 | 0 | 0 | 0 | 0 | 0 | 2.25E-05 | 0 | 0 | 0 |
| d__Eukaryota;k__Fungi;p__Basidiomycota;c__Agaricomycetes;o__Agaricales;f__Porotheleaceae;g__Porotheleum | 0 | 0 | 0 | 0 | 0 | 0 | 0 | 0 | 2.25E-05 | 0 | 0 | 0 |
| d__Eukaryota;k__Fungi;p__Basidiomycota;c__Agaricomycetes;o__Agaricales;f__Psathyrellaceae;g__Parasola | 0 | 0 | 0 | 0 | 0 | 0 | 0 | 0 | 0 | 0 | 2.25E-05 | 0 |
| d__Eukaryota;k__Fungi;p__Basidiomycota;c__Agaricomycetes;o__Agaricales;f__Tricholomataceae;g__Dictyolus | 0 | 0 | 0 | 0 | 0 | 0 | 0 | 2.25E-05 | 0 | 0 | 0 | 0 |
| d__Eukaryota;k__Fungi;p__Basidiomycota;c__Agaricomycetes;o__Agaricales;f__Tricholomataceae;g__Lepista | 0 | 0 | 0 | 0 | 0 | 0 | 0 | 0 | 0 | 0 | 0 | 2.25E-05 |
| d__Eukaryota;k__Fungi;p__Basidiomycota;c__Agaricomycetes;o__Agaricales;f__Tricholomataceae;g__Omphalina | 0 | 0 | 0 | 0 | 0 | 0 | 0 | 0 | 2.25E-05 | 0 | 0 | 0 |
| d__Eukaryota;k__Fungi;p__Basidiomycota;c__Agaricomycetes;o__Cantharellales;f__Cantharellales_fam_Incertae_sedis;g__Sistotrema | 0 | 0 | 0 | 0 | 0 | 0 | 0 | 2.25E-05 | 0 | 0 | 0 | 0 |
| d__Eukaryota;k__Fungi;p__Basidiomycota;c__Agaricomycetes;o__Hymenochaetales;f__Hymenochaetaceae;g__Phylloporia | 0 | 0 | 0 | 0 | 0 | 0 | 0 | 0 | 0 | 0 | 2.25E-05 | 0 |
| d__Eukaryota;k__Fungi;p__Basidiomycota;c__Agaricomycetes;o__Polyporales;f__Fomitopsidaceae;g__Tyromyces | 0 | 0 | 0 | 0 | 0 | 0 | 0 | 0 | 0 | 0 | 0 | 2.25E-05 |
| d__Eukaryota;k__Fungi;p__Basidiomycota;c__Agaricomycetes;o__Polyporales;f__Ganodermataceae;g__Perenniporia | 0 | 0 | 0 | 0 | 0 | 0 | 0 | 2.25E-05 | 0 | 0 | 0 | 0 |
| d__Eukaryota;k__Fungi;p__Basidiomycota;c__Agaricomycetes;o__Polyporales;f__Ganodermataceae;g__Yuchengia | 0 | 0 | 0 | 0 | 0 | 0 | 0 | 2.25E-05 | 0 | 0 | 0 | 0 |
| d__Eukaryota;k__Fungi;p__Basidiomycota;c__Agaricomycetes;o__Polyporales;f__Meruliaceae;g__Irpex | 0 | 0 | 0 | 0 | 0 | 0 | 0 | 0 | 2.25E-05 | 0 | 0 | 0 |
| d__Eukaryota;k__Fungi;p__Basidiomycota;c__Agaricomycetes;o__Polyporales;f__Steccherinaceae;g__Ceriporiopsis | 0 | 0 | 0 | 2.25E-05 | 0 | 0 | 0 | 0 | 0 | 0 | 0 | 0 |
| d__Eukaryota;k__Fungi;p__Basidiomycota;c__Agaricomycetes;o__Russulales;f__Auriscalpiaceae;g__Auriscalpium | 0 | 0 | 0 | 0 | 0 | 0 | 2.25E-05 | 0 | 0 | 0 | 0 | 0 |
| d__Eukaryota;k__Fungi;p__Basidiomycota;c__Agaricomycetes;o__Russulales;f__Peniophoraceae;g__unclassified_f__Peniophoraceae | 0 | 0 | 0 | 0 | 2.25E-05 | 0 | 0 | 0 | 0 | 0 | 0 | 0 |
| d__Eukaryota;k__Fungi;p__Basidiomycota;c__Tremellomycetes;o__Tremellales;f__Phaeotremellaceae;g__Phaeotremella | 0 | 0 | 0 | 0 | 0 | 0 | 0 | 0 | 0 | 2.25E-05 | 0 | 0 |
| d__Eukaryota;k__Fungi;p__Chytridiomycota;c__Spizellomycetes;o__Spizellomycetales;f__Spizellomycetaceae;g__unclassified_f__Spizellomycetaceae | 0 | 0 | 0 | 0 | 0 | 0 | 0 | 0 | 2.25E-05 | 0 | 0 | 0 |
| d__Eukaryota;k__Fungi;p__Glomeromycota;c__Archaeosporomycetes;o__Archaeosporales;f__Archaeosporaceae;g__unclassified_f__Archaeosporaceae | 0 | 0 | 2.25E-05 | 0 | 0 | 0 | 0 | 0 | 0 | 0 | 0 | 0 |
| d__Eukaryota;k__Fungi;p__Glomeromycota;c__Glomeromycetes;o__Diversisporales;f__Diversisporaceae;g__unclassified_f__Diversisporaceae | 0 | 0 | 0 | 0 | 0 | 2.25E-05 | 0 | 0 | 0 | 0 | 0 | 0 |
| d__Eukaryota;k__Fungi;p__Glomeromycota;c__unclassified_p__Glomeromycota;o__unclassified_p__Glomeromycota;f__unclassified_p__Glomeromycota;g__unclassified_p__Glomeromycota | 2.25E-05 | 0 | 0 | 0 | 0 | 0 | 0 | 0 | 0 | 0 | 0 | 0 |
