# Supplementary material for: Soil microbial community succession and physicochemical property changes affect Ganoderma leucocontextum growth in the Dadu river basin
Source: Front Microbiol. 2026 Jan 7;16:1666459. doi: 10.3389/fmicb.2025.1666459 (PMC12819783; doi:10.3389/fmicb.2025.1666459)
Supplement: Supplementary file 5 [file Data_Sheet_5.doc]

Supplementary Table 5 Bacterial alpha diversity indices

| Sample\Estimators | ACE | Chao | Shannon | Simpson | Pielou_e |
| --- | --- | --- | --- | --- | --- |
| GCK_1 | 6266.731368 | 5998.085453 | 7.098652 | 0.003835 | 0.837876289 |
| GCK_2 | 6389.785448 | 6120.308594 | 6.992541 | 0.007861 | 0.823299843 |
| GCK_3 | 6648.506091 | 6363.936782 | 7.098433 | 0.006514 | 0.832548167 |
| G1c_1 | 5397.579755 | 5211.337678 | 6.829542 | 0.006126 | 0.81828479 |
| G1c_2 | 5131.766878 | 4957.666667 | 6.785602 | 0.005851 | 0.819296488 |
| G1c_3 | 5003.469631 | 4827.440977 | 6.835097 | 0.005002 | 0.827082469 |
| G1m_1 | 5682.779177 | 5430.206897 | 6.212838 | 0.046029 | 0.743343567 |
| G1m_2 | 5453.880576 | 5214.113074 | 6.480161 | 0.026563 | 0.777647632 |
| G1m_3 | 5469.219934 | 5210.558324 | 6.367947 | 0.034164 | 0.763501059 |
| G1p_1 | 4912.88514 | 4722.426471 | 6.250477 | 0.011785 | 0.764454213 |
| G1p_2 | 5495.085812 | 5267.340881 | 6.648029 | 0.010508 | 0.799939869 |
| G1p_3 | 5056.396492 | 4946.941704 | 6.315219 | 0.013695 | 0.769173473 |

NOTE: Different lower-case and upper-case letters showed significant difference (P < 0.05) in the indices between the different growth stages of *G. leucocontextum*.
