# Supplementary material for: Soil microbial community succession and physicochemical property changes affect Ganoderma leucocontextum growth in the Dadu river basin
Source: Front Microbiol. 2026 Jan 7;16:1666459. doi: 10.3389/fmicb.2025.1666459 (PMC12819783; doi:10.3389/fmicb.2025.1666459)
Supplement: Supplementary file 6 [file Data_Sheet_6.doc]

Supplementary Table 6 Fungal alpha diversity indices

| Sample\Estimators | ACE | Chao | Shannon | Simpson | Pielou_e |
| --- | --- | --- | --- | --- | --- |
| G1c_1 | 1291.338927 | 1321.496241 | 4.6542 | 0.032643 | 0.670706107 |
| G1c_2 | 1185.239109 | 1202 | 3.785554 | 0.117748 | 0.55339935 |
| G1c_3 | 1454.492525 | 1421.939227 | 4.485365 | 0.037251 | 0.6395772 |
| G1m_1 | 692.252923 | 677.83908 | 2.003852 | 0.512255 | 0.316135089 |
| G1m_2 | 760.939166 | 754.28972 | 2.504383 | 0.377327 | 0.386293619 |
| G1m_3 | 743.93436 | 790.347826 | 2.598206 | 0.342589 | 0.40553374 |
| G1p_1 | 904.7144 | 909.578947 | 4.646968 | 0.02262 | 0.69161466 |
| G1p_2 | 945.608589 | 942.421053 | 4.395865 | 0.04266 | 0.653423948 |
| G1p_3 | 894.689563 | 883.622951 | 4.515282 | 0.02829 | 0.673355953 |
| GCK_1 | 1056.687578 | 1041.888112 | 4.333783 | 0.04236 | 0.638781108 |
| GCK_2 | 1102.390099 | 1070.15 | 4.326616 | 0.029377 | 0.639555221 |
| GCK_3 | 1164.747583 | 1153.892617 | 4.351875 | 0.031981 | 0.635200341 |

NOTE: Different lower-case and upper-case letters showed significant difference (P < 0.05) in the indices between the different growth stages of *G. leucocontextum*.
